# High throughput approaches reveal splicing of primary microRNA transcripts and tissue specific expression of mature microRNAs in *Vitis vinifera*

**DOI:** 10.1186/1471-2164-10-558

**Published:** 2009-11-25

**Authors:** Erica Mica, Viviana Piccolo, Massimo Delledonne, Alberto Ferrarini, Mario Pezzotti, Cesare Casati, Cristian Del Fabbro, Giorgio Valle, Alberto Policriti, Michele Morgante, Graziano Pesole, M Enrico Pè, David S Horner

**Affiliations:** 1 Dipartimento di Scienze Biomolecolari e Biotecnologie, Università degli Studi di Milano, Milano, Italy; 2 Scuola Superiore Sant'Anna, Pisa, Italy; 3 Dipartimento di Biotecnologie, Università degli Studi di Verona, Verona, Italy; 4 Dipartimento di Scienze Tecnologie e Mercati della Vite e Vino, Università degli Studi di Verona, Verona, Italy; 5 Dipartimento di Scienze Agrarie ed Ambientali, Università degli Studi di Udine, Udine, Italy; 6 CRIBI Biotechnology Centre, Dipartimento di Biologia, Università degli Studi di Padova, Padova, Italy; 7 Istituto di Genomica Applicata, Udine, Italy; 8 Istituto Tecnologie Biomediche, Consiglio Nazionale delle Ricerche, Bari, Italy; 9 Dipartimento di Biochimica e Biologia Molecolare "E. Quagliariello", Università di Bari, Bari, Italy

## Abstract

**Background:**

MicroRNAs are short (~21 base) single stranded RNAs that, in plants, are generally coded by specific genes and cleaved specifically from hairpin precursors. MicroRNAs are critical for the regulation of multiple developmental, stress related and other physiological processes in plants. The recent annotation of the genome of the grapevine (*Vitis vinifera *L.) allowed the identification of many putative conserved microRNA precursors, grouped into multiple gene families.

**Results:**

Here we use oligonucleotide arrays to provide the first indication that many of these microRNAs show differential expression patterns between tissues and during the maturation of fruit in the grapevine. Furthermore we demonstrate that whole transcriptome sequencing and deep-sequencing of small RNA fractions can be used both to identify which microRNA precursors are expressed in different tissues and to estimate genomic coordinates and patterns of splicing and alternative splicing for many primary miRNA transcripts.

**Conclusion:**

Our results show that many microRNAs are differentially expressed in different tissues and during fruit maturation in the grapevine. Furthermore, the demonstration that whole transcriptome sequencing can be used to identify candidate splicing events and approximate primary microRNA transcript coordinates represents a significant step towards the large-scale elucidation of mechanisms regulating the expression of microRNAs at the transcriptional and post-transcriptional levels.

## Background

MicroRNAs (miRNAs) are small (19-24 nt) non-coding RNAs that play important roles in the regulation of various cellular processes by inhibiting gene expression at the post-transcriptional level [1-3]. Many miRNAs interact with target mRNAs, leading to degradation or sequestration from the translational apparatus [4,5]; some miRNAs target other non-coding transcripts and are required for the generation of trans-acting small interfering RNAs (ta-siRNAs) [6]. miRNAs have been implicated in the regulation of key developmental, stress response and other physiological processes. While in animals many miRNAs are derived from introns or untranslated regions of coding messages, plant miRNAs are typically specified by dedicated MIR genes. These genes are, at least for the most part, transcribed by RNA polymerase II to yield capped and polyadenylated primary transcripts (pri-miRNA) [7]. The RNAse III enzyme Dicer-like-1 (DCL1) mediates the specific excision of mature miRNAs from the pri-miRNA via the initial generation of imperfect hairpin precursors (pre-miRNAs) and the subsequent excision of a duplex consisting of the mature microRNA and its complementary region (miRNA*) [8,9]. Most, if not all plant miRNAs then undergo methylation of the 2' hydroxyl group at the 3' ends of this duplex [10] and are subsequently exported to the cytosol, where one strand, the mature miRNA, is selectively incorporated into the RNA Induced Silencing Complex (RISC) which mediates interactions with target mRNAs [11].

The same, or highly similar mature miRNAs are often specified by different genomic loci within a species, and many, but by no means all, miRNAs show broad phylogenetic conservation - similar miRNAs are grouped into families. Several computational methods have been developed to identify putative pre-miRNAs by evaluating the capacity of the genomic context of sequences similar to known mature miRNAs to form hairpin structures exhibiting structural and thermodynamic features consistent with known pre-miRNAs (e.g. [12]). Purely *ab-initio *approaches to the prediction of non-conserved (lineage-specific) miRNAs have also yielded some notable successes (e.g. [13]), although such approaches are often plagued by an excess of false positive results. The most reliable method to identify putative novel pre-miRNAs remains the sequencing of small RNA fractions [10,14-20] coupled with the identification of plausible hairpin structures in flanking genomic sequences (e.g. [21]).

Once mature miRNA sequences have been identified, their expression in different tissues, developmental stages or environmental conditions can be studied through experimental approaches such as northern blotting, oligonucleotide arrays or deep-sequencing of isolated small RNA fractions. However, the fact that identical or highly similar mature miRNAs can derive from multiple loci within a single genome limits the capacity of such approaches to determine which genomic precursor loci are truly expressed.

Meaningful insight into the regulation of miRNA expression at the transcriptional and other levels is undoubtedly desirable in the context of post-genomic and systems biology initiatives. However, large scale and *in-silico *studies of the regulation of transcription of miRNAs require accurate definition of primary transcript coordinates on a genomic sequence, or at the very least, fairly accurate estimates of transcriptional start sites. Owing to their typically short physiological half-life, relatively few pri-miRNAs have been found in EST and large-scale full-length cDNA sequencing projects in plants. For the same reasons, manual cloning and characterization of primary miRNAs is a laborious process, and has been performed for relatively few plant miRNAs [7,22-24].

To date, large-scale analysis of expression of plant miRNAs and associated prediction of precursor sequences have been restricted to relatively few species and the paucity of complete plant genome sequences limits the possibilities for studies incorporating extensive genomic information. The recently published genome sequence of the grapevine (*Vitis vinifera *L.) [25] provides the first opportunity to study the potential roles of miRNAs in fruit maturation and other physiological processes of a commercially important species in the context of a complete genome sequence.

We have previously used comparative methods to predict 140 putative pre-miRNAs (representing 28 conserved miRNA families) in the grapevine genome [25]. Here we present experimental validation of a large number of these predictions using several high throughput methodologies. Oligonucleotide arrays reveal that several of these families show significant changes in expression levels in different tissues and during fruit maturation. Small RNA deep-sequencing allowed the precise definition of boundaries of mature miRNA sequences where comparative predictions left some ambiguity.

Additionally, we demonstrate that deep sequencing of the polyA^+ ^transcriptome permits the precise identification of which candidate precursors are expressed in different tissues and shows that, in many cases, fairly precise estimates of primary transcript coordinates may be inferred from such data. Finally, we show that patterns of splicing and alternative splicing of pri-miRNAs may be elucidated from whole transcriptome deep sequencing data and confirm that a significant proportion of grapevine pri-miRNAs are subjected to such processes, consistent with the suggestion that post-transcriptional regulation might play a widespread role in the regulation of plant miRNA maturation.

## Results and Discussion

In the previous description of 8.4 fold coverage assembly of the grapevine (*Vitis vinifera *L.) genome [25] we identified 164 candidate conserved miRNAs. Here we present a comprehensive characterization of conserved miRNAs in grapevine including a refinement of mature miRNA sequences and important information regarding pattern of expression of both mature miRNAs and precursors. Three complementary approaches were followed to characterize the expression pattern of both the mature miRNAs and their precursors. To allow a comparison between methods, all technologies were applied to leaf tissue. Whole transcriptome deep sequencing was performed on all tissues available from the highly homozygote sequenced clone PN40024. To maximize the coverage of tissues studied, microarray and 454 transcriptomic analyses were performed on berries - organs of particular agronomic importance - from other clones as they cannot be easily obtained from the very weak PN40024 clone.

### Comparative prediction of microRNA precursors

We previously used the MicroHarvester software [12] with all plant miRNAs present in release 9.1 of miRBase [26] to identify 164 candidate conserved miRNAs and their precursors in the 8.4 fold coverage assembly of the genome of the grapevine (*Vitis vinifera *L.) [25]. Manual refinement of these predictions provided 140 high-confidence candidate pre-miRNAs classified in 28 conserved families (79 unique predicted mature microRNA sequences).

For the most part, we confirm existing patterns of miRNA family conservation with respect to completely sequenced plant genomes for which extensive analyses of miRNAs have been performed (*Arabidopsis thaliana*, *Populus trichocarpa*, *Oryza sativa *and *Physcomitrella patens*). Of the 28 families for which we identified putative precursor sequences in *Vitis vinifera *(Table 1), 9 are represented in all four of these species and a further 10 have been characterized in all three magnoliophytes. One family (miR403) is present in both of the previously sequenced Dicots, while two previously Arabidopsis-specific families (miR828 and miR845) were predicted in grapevine (suggesting their loss or - as yet - undetected presence in poplar), while miR479 and miR482 (annotated only in poplar and grapevine) are likely to have evolved in a common ancestor of these organisms after its divergence from the Arabidopsis lineage [25]. miR477 precursors have been characterized only in poplar, grapevine and *P. patens*, while a series of miR535 precursors represent the first members of this family to be identified in core eudicots, having been identified only in *P. patens*, rice and more recently in the California poppy (*Eschscholzia californica*) [27]. The only families tested for which members have been identified in poplar and at least one other of the aforementioned genomes but for which microHarvester failed to identify candidate precursors in the grapevine genome were miR472, miR530 and miR827.

**Table 1:** Expression of mature- and pre-miRNAs in *Vitis vinifera*

**miRNA**	**Sequence**	**Expression data**				**miRNA**	**Sequence**	**Expression data**			
	
		**A**	**B**	**C**	**D**			**A**	**B**	**C**	**D**
MIR156A	TGACAGAAGAGAGGGAGCAC	2229	____	N	__I___	MIR171G	TTGAGCCGAACCAATATCACC	0	__RC	N	R*L*I*___

MIR156B	TGACAGAAGAGAGTGAGCAC	835876	___C	N	______	MIR172A	AGAATCTTGATGATGCTGCAT	3450	_S_C	N	______

MIR156C	TGACAGAAGAGAGTGAGCAC	835876	____	N	______	MIR172B	AGAATCTTGATGATGCTGCAT	73450	____	N	______

MIR156D	TGACAGAAGAGAGTGAGCAC	835876	LSRC	N	______	MIR172D	AGAATCTTGATGATGCTGCAT	73450	____	N	_LIBgBv_

MIR156E	TGACAGAGGAGAGTGAGCAC	251	____	N	______	MIR172C	GAATCTTGATGATGCTGCAG	1	____	N	_LIBgBv_

MIR156F	TTGACAGAAGATAGAGAGCAC	30	_S__	N	RLIBgBvBm	MIR319B	TTGGACTGAAGGGAGCTCCCT	1	____	N	RLIBgBvBm

MIR156G	TTGACAGAAGATAGAGAGCAC	30	LS_C	N	RLIBgBvBm*	MIR319C	TTGGACTGAAGGGAGCTCCCT	1	_S__	N	RLIBgBvBm

MIR156I	TTGACAGAAGATAGAGAGCAC	30	_S__	N	RLIBg*BvBm	MIR319E	TTTGGACTGAAGGGAGCTC	1	_S_C	Y	RLIBgBvBm

MIR156H	TGACAGAAGAGAGAGAGCAT	69	LS__	Y	______	MIR319G	ATTGGACTGAAGGGAGCTCCC	0	___C	N	RLIBgBvBm

MIR159A	CTTGGAGTGAAGGGAGCTCTC	0	____	N	RLIBgBvBm	MIR319F	TTGGATTGAAGGGAGCTCCCT	1	_S__	N	RLI*BgBv*Bm*

MIR159B	CTTGGAGTGAAGGGAGCTCTC	0	____	N	RLIBgBvBm	MIR390	AAGCTCAGGAGGGATAGCGCC	141	_S__	N	RLIBgBvBm

MIR159C	TTTGGATTGAAGGGAGCTCT	124	LSRC	N	RLIBgBvBm	MIR393A	TTCCAAAGGGATCGCATTGAT	14983	____	Y	RLIBgBvBm

MIR160A	TGCCTGGCTCCCTGAATGCCA	276	____	N	RLI*BgBv*Bm*	MIR393B	TCCAAAGGGATCGCATTGATC	398	LS__	Y	RLIBgBvBm

MIR160B	TGCCTGGCTCCCTGAATGCCA	276	____	Y	RLIBgBvBm	MIR394A	TTGGCATTCTGTCCACCTCC	748	_S__	N	______

MIR160E	TGCCTGGCTCCCTGAATGCCA	276	____	Y	RLIBgBvBm	MIR394B	TTGGCATTCTGTCCACCTCC	748	LS_C	N	______

MIR160C	TGCCTGGCTCCCTGTATGCCA	130	_SRC	N	RLIBgBvBm*	MIR394C	TTGGCATTCTGTCCACCTCC	748	____	N	______

MIR160D	TGCCTGGCTCCCTGTATGCCA	130	____	N	RLIBgBvBm	MIR395A	TGAAGTGTTTGGGGGAACTC	198	____	N	RLIBgBvBm

MIR160F	TGCCTGGCTCCCTGTATGCCA	130	____	N	RL*IBg*Bv*Bm	MIR395B	TGAAGTGTTTGGGGGAACTC	198	____	N	RLIBgBvBm

MIR162	TCGATAAACCTCTGCATCCAG	1	LS_C	Y	RLIBgBvBm	MIR395C	TGAAGTGTTTGGGGGAACTC	198	____	N	RLI*BgBvBm

MIR164A	TGGAGAAGCAGGGCACGTGCA	13841	____	N	RLIBg__	MIR395D	TGAAGTGTTTGGGGGAACTC	198	____	N	RLIBgBvBm

MIR164C	TGGAGAAGCAGGGCACGTGCA	13841	L___	N	RLIBg__	MIR395E	TGAAGTGTTTGGGGGAACTC	198	____	N	RLIBgBvBm

MIR164D	TGGAGAAGCAGGGCACGTGCA	13841	____	N	RLIBg__	MIR395F	TGAAGTGTTTGGGGGAACTC	198	____	N	RLIBgBvBm

MIR164B	TGGAGAAGCAGGGCACATGCT	1	____	N	_LI*Bg__	MIR395L	TGAAGTGTTTGGGGGAACTC	198	____	N	RLIBgBvBm

MIR166A	TCGGACCAGGCTTCATTCCT	181	LSRC	Y	RLIBgBvBm	MIR395 M	TGAAGTGTTTGGGGGAACTC	198	____	N	RLIBgBvBm

MIR166B	TCGGACCAGGCTTCATTCCT	181	LS__	N	RLIBgBvBm	MIR395G	TGAAGTGTTTGGGGGAACTC	198	____	N	RLIBgBvBm

MIR166C	TCGGACCAGGCTTCATTCCCC	219273	LSRC	N	RLIBgBvBm	MIR395H	TGAAGTGTTTGGGGGAACTC	198	____	N	RLIBgBvBm

MIR166D	TCGGACCAGGCTTCATTCCCC	219273	_S__	N	RLIBgBvBm	MIR395I	TGAAGTGTTTGGGGGAACTC	198	____	N	RLIBgBvBm

MIR166E	TCGGACCAGGCTTCATTCCCC	219273	_SRC	N	R*LI*Bg*Bv*Bm*	MIR395J	TGAAGTGTTTGGGGGAACTC	198	_S__	N	RLIBgBvBm

MIR166F	TCGGACCAGGCTTCATTCCCC	219273	____	N	RLIBgBvBm	MIR395K	TGAAGTGTTTGGGGGAACTC	198	____	N	RLIBgBvBm

MIR166G	TCGGACCAGGCTTCATTCCCC	219273	____	N	RLIBgBvBm*	MIR395N	CTGAAGAGTCTGGAGGAACTC	0	____	N	_L____

MIR166H	TCGGACCAGGCTTCATTCCCC	219273	_S_C	N	RLIBgBvBm	MIR396B	TTCCACAGCTTTCTTGAACTT	2204	LS_C	N	RL_Bg__

MIR167A	TGAAGCTGCCAGCATGATCTG	166	L_RC	N	RLIBgBvBm	MIR396A	TTCCACAGCTTTCTTGAAC	810	_S__	N	RL_Bg__

MIR167B	TGAAGCTGCCAGCATGATCTA	4965	L__C	Y	RL*IBg*BvBm	MIR396C	TTCCACAGCTTTCTTGAAC	810	____	N	RL_Bg__

MIR167C	TGAAGCTGCCAGCATGATCT	89	____	N	RLIBgBvBm	MIR396D	TTCCACAGCTTTCTTGAAC	810	_S_C	N	RL_Bg__

MIR167D	TGAAGCTGCCAGCATGATCTA	4965	LS__	N	RLIBgBvBm	MIR397A	ATTGAGTGCAGCGTTGATGAA	714	LS_C	N	R__Bg__

MIR167E	TGAAGCTGCCAGCATGATCTA	4965	____	N	RLIBgBvBm	MIR397B	ATTGAGTGCAGCGTTGATGAA	714	____	N	R__Bg__

MIR168	TCGCTTGGTGCAGGTCGGGAA	5	LSRC	Y	RLIBgBvBm	MIR398A	TTCTCAGGTCACCCCTTTGGG	13	_SRC	N	RL_Bg__

MIR169B	TGAGCCAAGGATGGCTTGCCG	0	____	N	_L__BvBm	MIR398B	CTCATGTGTTCTCAGGTCGCC	15	LSRC	N	R__Bg__

MIR169H	TGAGCCAAGGATGGCTTGCCG	0	____	N	_L__BvBm	MIR398C	CTCATGTGTTCTCAGGTCGCC	15	LSRC	N	R__Bg__

MIR169A	CAGCCAAGGATGACTTGCCGG	675	____	N	_LIBgBvBm	MIR399A	TGCCAAAGGAGAATTGCCCTG	106	L___	N	R_I___

MIR169C	CAGCCAAGGATGACTTGCCGG	675	____	N	_LIBgBvBm	MIR399H	TGCCAAAGGAGAATTGCCCTG	106	L___	N	R_I___

MIR169J	CAGCCAAGGATGACTTGCCGG	675	____	N	_LIBgBvBm	MIR399B	TGCCAAAGGAGAGTTGCCCTG	34	____	N	R__Bg_Bm

MIR169K	CAGCCAAGGATGACTTGCCGG	675	____	N	_LIBgBvBm	MIR399C	TGCCAAAGGAGAGTTGCCCTG	34	____	N	R__Bg_Bm

MIR169S	CAGCCAAGGATGACTTGCCGG	675	____	N	_LI*BgBvBm	MIR399I	CAAAGGAGAGTTGCCCTG	1	L_RC	N	R__Bg_Bm

MIR169W	CAGCCAAGGATGACTTGCCGG	675	____	N	_LIBgBvBm	MIR399D	TGCCAAAGGAGATTTGCTCGT	0	____	N	______

MIR169L	GAGCCAAGGATGACTTGCCGT	0	____	N	_LIBgBvBm	MIR399E	TGCCAAAGGAGATTTGCCCGG	0	___C	N	______

MIR169 M	AGCCAAGGATGACTTGCCGGC	16	____	N	_LI*BgBv*Bm*	MIR399F	TGCCGAAGGAGATTTGTCCTG	0	____	N	______

MIR169N	AGCCAAGGATGACTTGCCGGC	16	____	N	_L*I*Bg*Bv*Bm*	MIR399G	TGCCAAAGGAGATTTGCCCCT	0	____	N	R_I__Bm

MIR169O	GAGCCAAGGATGACTTGCCGC	0	____	N	_LIBgBvBm	MIR403A	TTAGATTCACGCACAAACTCG	0	____	N	RLIBgBvBm

MIR169P	AGCCAAGGATGACTTGCCG	16	____	N	_LIBgBvBm	MIR403B	TTAGATTCACGCACAAACTCG	0	____	N	RLIBgBvBm

MIR169Q	AGCCAAGGATGACTTGCCG	16	____	N	_LIBgBvBm	MIR403C	TTAGATTCACGCACAAACTCG	0	___C	N	RLI*BgBvBm*

MIR169E	TAGCCAAGGATGACTTGCCTG	8	L___	N	_LIBgBvBm	MIR403D	TTAGATTCACGCACAAACTCG	0	____	N	RLIBgBvBm

MIR169F	CAGCCAAGGATGACTTGCCGA	317	___C	N	_LIBgBvBm*	MIR403E	TTAGATTCACGCACAAACTCG	0	____	N	RLIBgBvBm

MIR169G	CAGCCAAGGATGACTTGCCGA	317	_S__	N	_LIBgBvBm	MIR403F	TTAGATTCACGCACAAACTCG	0	_SRC	N	RLIBgBvBm

MIR169R	TGAGTCAAGGATGACTTGCCG	0	____	N	_LI*BgBv*Bm*	MIR408	TGCACTGCCTCTTCCCTGGC	131	LSRC	Y	RLIBgBvBm

MIR169T	TGAGTCAAGGATGACTTGCCG	0	____	N	_L*I*Bg*Bv*Bm*	MIR477A	ATCTCCCTCAAAGGCTTCCAA	0	____	N	___BgBvBm

MIR169U	TGAGTCAAGGATGACTTGCCG	0	____	N	_L*I*Bg*Bv*Bm*	MIR479	TGTGGTATTGGTTCGGCTCATC	2	____	N	______

MIR169V	AAGCCAAGGATGAATTGCCGG	0	____	N	__IBg__	MIR482	CCTACTCCTCCCATTCC	0	LSRC	Y	______

MIR169X	TAGCCAAGGATGACTTGCCTA	1	____	Y	_LIBgBvBm	MIR535A	TGACAACGAGAGAGAGCACGCT	0	____	Y	RLIBgBvBm

MIR169Y	TAGCGAAGGATGACTTGCCTA	0	____	N	__I___	MIR535B	TGACAACGAGAGAGAGCACGCT	0	____	Y	RLIBgBvBm

MIR169I	GAGCCAAGGATGACTGGCCGT	0	____	N	_L_Bg__	MIR535C	TGACAACGAGAGAGAGCACGCT	0	____	Y	RLI*BgBv*Bm

MIR169D	CAGCCAAGAATGATTTGCCGG	0	____	N	______	MIR535D	TGACAACGAGAGAGAGCACGCT	0	____	Y	RLIBgBvBm

MIR171B	TGATTGAGCCGCGTCAATATC	0	____	N	R_____	MIR535E	TGACAACGAGAGAGAGCACGCT	0	____	Y	RLIBgBvBm

MIR171C	TGATTGAGCCGTGCCAATATC	637	____	N	RLIBg__	MIR828A	TCTTGCTCAAATGAGTATTCCA	0	____	N	______

MIR171D	TGATTGAGCCGTGCCAATATC	637	____	N	RLIBg__	MIR828B	TCTTGCTCAAATGAGTGTTCCA	0	____	N	______

MIR171A	TGATTGAGCCGTGCCAATATC	637	_SRC	Y	RLIBg__	MIR845A	TAGCTCTGATACCAATTGATA	0	____	N	______

MIR171I	TGATTGAGCCGTGCCAATATC	637	____	N	RLIBg__	MIR845B	TAGCTCTGATACCAATTGATA	0	____	N	______

MIR171E	TGATTGAGCCGCGCCAATATC	53	___C	N	RLI*BgBv*Bm*	MIR845C	AGGCTCTGATACCAATTGATG	0	____	N	______

MIR171H	TGGTTGAGCCGCGCCAATATC	0	____	N	RLIBgBvBm	MIR845D	TGGCTCTGATACCAATTGATGG	0	____	N	______

MIR171F	TTGAGCCGCGCCAATATCACT	0	_S__	N	RLIBgBv_	MIR845E	TGGCTCTGATACCAATTGATGG	0	____	N	______

For each predicted pre-miRNA the table reports: the mature sequence, the number of perfect matching short RNA reads observed in leaf (column A), tissues in which significant expression of the precursor was observed by Illumina whole transcriptome sequencing (column B, where L = leaf, R = root, S = stem, C = callus), the presence of 454 reads including the precursor sequence in leaf (column C, where Y = yes, N = no), and tissues where the Combimatrix oligoarray showed significant expression of the mature sequence (column D, where L = leaf, I = inflorescence, R = root, Bg = immature berry, Bv = veraison, Bm = mature berry). Asterisks indicate signal detected for precursor in that tissue. Mature miRNAs are ordered to reflect expected cases of crosshybridization for oligonucleotide arrays. For all microRNAs, chromosome, strand and coordinates of the precursor miRNA are provided (scaffold coordinates indicate that the miRNA was situated on a scaffold not incorporated into the 8.4× genome assembly).

With respect to the reference annotation of protein coding genes in the *Vitis vinifera *genome, 127 putative pre-miRNAs were intergenic in location (17 overlapped with annotated genes but on the non-coding strand). Four precursor predictions fell within or overlapped annotated coding or UTR exons although homology searches and transcriptomics data generated subsequently to the initial annotations call into question the validity of all but two of these exon annotations. miRNA 156 h is probably an incorrect prediction derived from a coincidentally plausible hairpin structure formed by the opposite strand to the presumed target (a *Squamosa-promoter Binding Protein *(*SBP*) box gene). A similar situation is observed for miR171 g which falls on the opposite strand to to a GRAS domain transcription factor gene. Nine precursor predictions were apparently intronic in location. Manual checks of the automated annotations suggested that all of the introns putatively containing pre-miRNAs were likely to be erroneous predictions, being atypically long (over 13 kb) and interrupting putative retroelement derived genes or obvious fusion gene predictions (not shown).

### Deep sequencing of small RNAs from grapevine leaf tissue

We generated 37,219,323 reads with Illumina sequencing of small RNA isolated from *Vitis vinifera *L. clone PN40024 leaves. 15,261,352 individual small RNA reads of 19-24 bases (41% of the total reads generated) representing 2,626,822 unique sequences were mapped to the draft genome after removal of adapter sequences. Over 40% of the total mapped sequences were of length 21 bases and accounted for 26.3% of the genomic loci represented by the mapped data (mean redundancy of 9.25 reads/locus). 34% of loci represented were of length 24 (13.4% of tags sequenced) with a mean redundancy of 2.27 reads/locus, suggesting, in accord with other studies [28,29], that miRNAs in our sample tend to be expressed at higher levels or processed more specifically than the more heterogeneous 24 base small RNAs [see Additional file 1: Supplemental Table S1].

**Figure 1:**
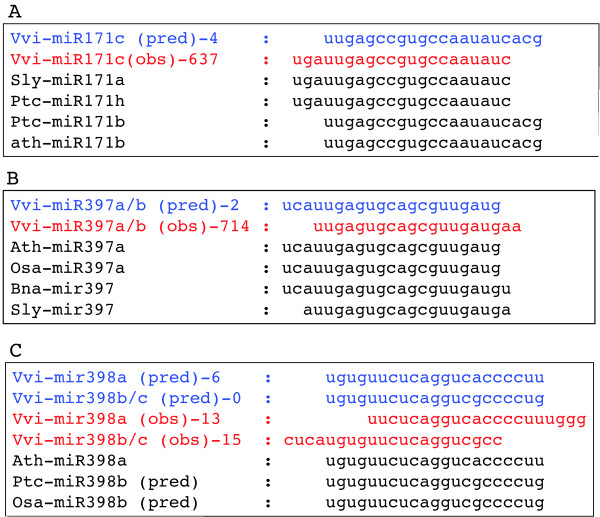
**Alignments of predicted and observed mature miRNA sequences for three miRNAs**. For each miRNA, the sequence predicted by comparative analyses and the number of corresponding small RNA reads are shown in blue, while the most frequent small RNA derived from the genomic locus (and the corresponding number of observed reads) are shown in red. Homologous miRNAs from other plant species are shown for reference. A: Vvi-miR171c, B: Vvi-miR397a/b, C: Vvi-miR398a/b and c.

Mapping of the short tags onto the genome sequence revealed that of the 28 families predicted by our comparative analysis, 22 showed at least one sequence tag either in exact or very close correspondence to the position of one of the predicted mature sequences (the exceptions being miR403, miR477, miR535, miR828, miR482 and miR845). For some families, thousands or even hundreds of thousands of short RNA sequences were recovered (miR156, miR164, miR166, miR167, miR172, miR393, miR396), while less than 1000 sequences corresponded to each of the remaining represented families. In some cases, the most commonly observed sequences were identical to at least one of the predicted mature sequences (notably: miR156, miR160, miR164, miR167, miR169, miR172, miR394, miR399) while for other families, the predominant mature miRNA sequenced exhibited small variations (shifts or differences of length of one or two bases) with respect to the predicted mature sequences (e.g. miR166, miR393, miR395, miR396, miR408). This finding was not unexpected given the variation in mature miRNA lengths within families observed in other plant species and the nature of the comparative method used to generate the initial predictions. For three families (miR171, miR397, mir398) the predominant species sequenced showed greater shifts with respect to the prediction. While in the case of miR171 and miR397, shifts of up to three bases within or between organisms are registered in miRBase (Figure 1A, B), the grapevine miR398 family presented several mature sequences that varied by up to 8 bases with respect to one another (Figure 1C). For other predicted precursors for which matching small RNA reads were recovered, the vast majority of reads conform to the sequences indicated in Table 1 [see also Additional File 1: Supplemental figure S3], consistent with the primary requirement for the annotation of plant miRNA sequences [30].

We recovered a number of reads that include an additional 3' base that does not correspond to any genomic locus, this tendency has been observed in other species (e.g. [28]). Furthermore, a low but notable proportion of mapped small RNA sequences show mismatches to the genomic sequence while preferentially mapping only to putative miRNA precursor loci. This is probably due to errors in sequencing or during reverse transcription or amplification. Some families (notably miR168) show a greatly exaggerated tendency towards sequence errors with 13,477 short sequences showing a single substitution (G in the genomic sequence, A in the reads recovered) from the predicted sequence and not exhibiting satisfactory alternative map positions on the grapevine genome (not shown). This is possibly due to known increased error rate of the Illumina sequencing strategy in GC rich sequences [31] although the intriguing possibility of RNA editing cannot be excluded. The expected miR482 mature sequence was not observed but the miRNA* sequence was present, albeit at low frequency. During the course of these analyses it became clear that two precursors (miR172a and 172b) were likely to derive from the opposite strand from that initially predicted. Where appropriate, corrected mature sequences have been deposited in miRBase.

Due to identity or similarity among mature miRNAs belonging to the same family, deep sequencing of small RNAs does not allow consistent unambiguous assignment of mature miRNAs to their genomic loci of origin. Nevertheless our analysis provided indications of the presence and abundance of 47 distinct mature miRNAs from conserved families in leaves - corresponding to up to 97 distinct precursor loci (see Table 1 for summary and Additional File 1: Supplemental figure S3 for detailed maps of all small RNA reads mapping to predicted precursors). The error prone nature of the small RNA deep sequencing data likely gives rise to a certain number of false positive matches. In fact in cases where hundreds of thousands of reads represent a single family, sequence and reverse transcription errors lead to the coincidental generation of mature sequences that perfectly match other precursor sequences. For example, over 800,000 reads perfectly matched miR156b/c/d, while over 2000 reads matched miR156a (whose sequence differs from the miR156b/c/d mature sequence by a single base). The mean Illumina base call quality score for this position in the miR156a matches was 11.7 versus 30.6 for the same position in the miR156b/c/d matching tags (37.4 and 35.1 for the flanking positions in the miR156a matched sequences). A similar pattern was observed for several other mature/precursor clusters and we conclude that, particularly when mature sequences differ by only a single base, it is difficult to reliably differentiate between precursors of origin with current small RNA deep-sequencing data.

### Oligonucleotide arrays

A 12K CombiMatrix custom array was developed to validate our *in-silico *miRNA predictions and to profile miRNAs expression in different tissues.

Slides were hybridized with low molecular weight RNA (LMW-RNA), extracted from six grapevine *(V. vinifera *L. cv Corvina) tissues: ripening berries (three stages analyzed), roots, leaves and young inflorescences. Each hybridization and LMW-RNA extraction was performed twice.

**Figure 2:**
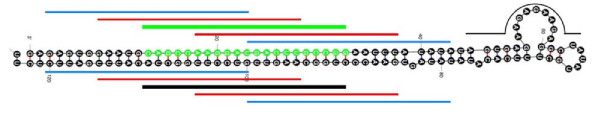
**Oligonucleotide design strategy for Combimatrix custom oligonucleotide array**. Probes were designed complementary to the predicted mature miRNA (green line) and miRNA* (thick black line) sequences. Additional probes were designed to the loop region (thin black line) as well as probes shifted 5 nucleotides (red lines) and 10 nucleotides (blue lines) with respect to the miRNA and miRNA* sequences.

In addition to the mature miRNA sequences, the probe set included probes shifted 5 or 10 bases 3' or 5' with respect to the central base of the corresponding mature miRNAs as well as probes derived from regions of the stem not predicted to overlap with the mature miRNA sequence and controls containing maximally destabilizing substitutions with respect to probe sequences (Figure 2). Except for probes shifted 5 nucleotides towards the 5' end of the miRNA precursor, for more than 90% of the probes a signal drop-off greater than 90% was observed - indicating no significant hybridization for these probes occurred. On the contrary, for probes shifted 5 nucleotide towards the 5' end of the miRNA precursor the lack of signal drop-off might be due to the fact that probes were synthesized with their 3' termini towards the slide, and that no "spacer" oligonucleotide was used (according to CombiMatrix protocols). As a consequence, steric effects might reduce the specificity determined by the 3'-most five bases of the probes.

Other than for 26 out of 140 pre-miRNAs (Table 1), no detectable signals were recorded for the probes designed on the precursor loop regions - likely due to size fractionation of RNA samples and the relatively short half-life of pre-miRNAs. We conclude that our miRNA expression data are principally derived from mature miRNAs molecules, without appreciable pre-miRNA contamination.

Finally, it should be noted that recent studies have demonstrated appreciable levels of cross-hybridization between closely related miRNAs and probes differing by only one or two bases [32]. It is therefore difficult to exclude the possibility that cross-hybridization within miRNA families causes a distortion of quantitative estimates of expression levels of some individual mature miRNA sequences.

### Microarray analysis of miRNA expression

Of the mature miRNA sequences considered, 56 (corresponding to 23 different families), showed significant expression in at least one tissue tested (Table 1 and Additional File 1: Supplemental Figure S2), and another 6 showed a borderline signal. Specifically, 41 different miRNAs showed significant signal in roots, 47 in leaves, 49 in young inflorescences, 53 in green berries, 42 in berries at veraison (the point where growth ends and maturation begins) and 40 in mature berries.

**Figure 3:**
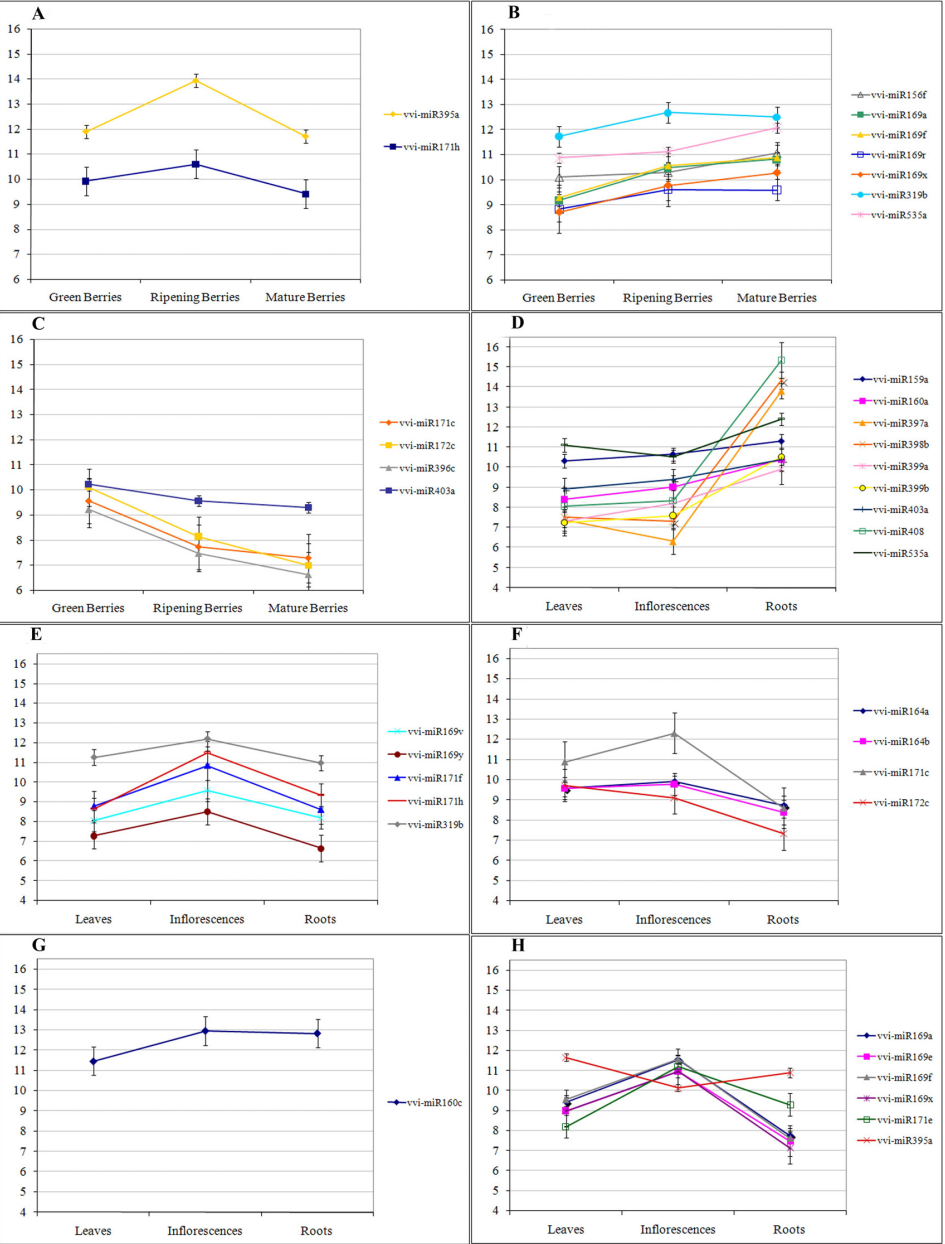
**Differential expression of mature miRNAs by tissue**. miRNAs showing significant changes in expression by tissue are reported. Panels A-C: miRNAs differentially expressed in one stage of berry ripening A: at veraison, B: in green berries, C: in mature berries. Panel D: miRNAs more highly expressed in roots, Panel E: miRNAs more highly expressed in inflorescences, Panel F: miRNAs less expressed in roots, Panel G: miRNAs less expressed in leaves, Panel H: miRNAs showing significant differences in all tissues tested. Error bars indicate confidence intervals. For all panels, the Y axis shows Log2 of the normalized median of spot intensities.

To evaluate the statistical significance of the differential expression of mature miRNAs in the six tissue considered, we set up two distinct comparisons: one among the three developmental stages of the ripening berries and the other one among leaves, roots and inflorescences. ANOVA analyses were performed with a P-value threshold of 0.05 and subsequently a Scheffè test was used to assess which of the three tissues showed significant differences. Thirteen different mature miRNAs showed a statistically significant change in signal between the ripening stages of the berry (Figure 3A-C), and 27 miRNAs showed significant changes in their expression when comparing three different tissues (leaves, roots and inflorescences)(Figure 3D-H). miR395a and miR171 h show a distinctive pattern of expression - being highly expressed at veraison with respect to the other two stages (4.4 and 2.3 fold changes of expression level respectively) (Figure 3A). Seven miRNAs (miR156f, miR169a, miR169f, miR169r, miR169x, miR319b and miR535a) are more expressed in mature berries than in green berries (Figure 3B). Four miRNAs (miR171c, miR172c, miR396c, miR403a) are, on the contrary, more expressed in green berries, their expression decreasing during ripening (Figure 3C).

Clear patterns also emerge from analyses of differential expression between roots, leaves and young inflorescences. Thirteen miRNAs are significantly differentially expressed in roots, showing a similar expression in the other tissues. In particular miR397a, miR398b and miR408 all show at least 100 fold higher expression in root than either leaf or early inflorescences, while miR159a, miR160a, miR399a, miR399b, miR403a and miR535 show more modest, but still significant, changes in the same comparisons (Figure 3D). On the contrary miR164a, miR164b, miR171c and miR172c show a significantly lower level of expression in roots (Figure 3F).

Five miRNAs (miR169v, miR169y, miR171f, miR171 h and miR319b) yield significantly higher signals in young inflorescences than both leaves and roots (between 2 and 7.2 fold higher levels in this tissue)(Figure 3E). Only one miRNA, miR160c, shows a leaf-specific expression profile (2.5 fold lower level in leaves with respect to other tissues) (Figure 3G). Finally, six miRNAs (miR169a, miR169e, miR169f, miR169x, miR171e and miR395a) exhibit significant differences in expression levels in all comparisons between leaf, root and inflorescences (Figure 3H). Five of these miRNAs (169a, 169e, 169f, 169x and 171e) show the highest expression in young inflorescences and the lowest in roots.

Following the widespread assumption that many miRNA/target interactions are conserved between related species [1,2], our data regarding differential expression of mature miRNA sequences raise some intriguing possibilities particularly with respect to the potential importance of miRNA in the regulation of fruit maturation.

Li et al. [33] recently showed that the transcription factor NFYA5 is targeted by miR169 and that overexpression of miR169 leads to excessive water loss through leaves and hypersensitivity to drought stress in *A. thaliana*. In this light, the preponderance of miR169 family members in the group of miRNAs upregulated in mature berries is striking and might reflect a mechanism to protect maturing fruit from dehydration. We also note that miR535 family, identified so far only in *O. sativa *and *P. patens *[34] is upregulated during berry maturation. This is a first indication of a possible function of miR535 for which no information was previously available. miR396c shows 6 fold decrease in expression during ripening. The mir396 family targets seven *Growth Regulating Factor *(*GRF*) genes in Arabidopsis [13]. *GRF *genes encode putative transcription factors associated with cell expansion in leaf and other tissues in *A. thaliana *and *O. sativa *[35,36]. A potential role for miR396 in the regulation of cell expansion during fruit maturation is an intriguing hypothesis. In addition, recent data also link miR396 to responses to abiotic stresses including drought [37], again suggesting the importance of water homeostasis during berry ripening. miR172, downregulated during berry maturation, targets *Apetala 2 *(AP2) -like transcription factors, regulators of flowering time, organ identity and of vegetative phase change [38]. In grapevine, genes related to AP2 are upregulated at veraison, being involved in berry maturation [39] and putatively connected with abiotic and biotic stress resistance. This evidence fits well with our findings. The sharp up-regulation of miR395 at veraison suggests a further role for miRNAs in an agronomically important aspect of grape maturation. miR395 is known to contribute to the regulation of sulfur metabolism, targeting both sulfate transporters and ATP sulphurylase genes. A direct connection between ATP sulfurylases and berry maturation has not been demonstrated, but it is known that a Glutathione S-transferase is strongly connected with berry ripening and in particular with coloration during berry development [39].

miR397a, miR398b and miR408 which are extremely highly expressed in root tissues target various copper proteins: plantacyanin, laccases and a superoxide dismutase, all putatively involved in stress responses and lignification [13,15,40,41]. These miRNAs have also been shown to be coexpressed in Arabidopsis under conditions of copper deprivation [42]. Moreover some *laccase *genes in Arabidopsis are root specific (for example *AtLAC15*) or mostly expressed in roots [43] and are involved in root elongation and lignification [44]. Given that grapevine roots are much more lignified than those of Arabidopsis, it is plausible that regulation of laccase expression is vital in the grapevine. It is interesting to note that the laccase family is, along with other polyphenol oxidase gene families, massively expanded in grapevine with respect to Arabidopsis (>60 genes in *V. vinifera*, 17 in Arabidopsis).

The complementary approaches utilized in the current work allowed us to uncover relevant shortcomings of both oligoarray and small RNA deep sequencing in the characterization of miRNA expression patterns. In particular, oligonucleotide arrays, for the most part, do not yield sufficient specificity to discriminate between family members. Our custom array, in which sequences of various length outside the predicted mature miRNA were included, clearly indicates that cross-hybridization is likely to present a confounding factor. For small RNA sequencing by Illumina technology, sequence features and/or post-transcriptional modification may be important features causing unforeseen biases determining an underestimate of abundance of specific miRNA families. This is suggested by the fact that some families, such as miR162, miR535, mirR403, miR482 generated few or no leaf small

RNA sequence reads, even if they can be clearly detected by northern blotting (miR162, miR482, not shown) or oligonucleotide arrays (miR162, mir403, miR535) in the same tissues. Our data suggest that this anomaly is not caused by the presence of unprocessed precursors in the samples used for oligonucleotide array analyses, although we cannot exclude the possibility of environmentally influenced or clone-specific expression patterns of these microRNAs influencing our results. However, it is interesting to note that some of these sequences (e.g. miR535 which contains several repeats of the motif 'GAG') have characteristics known to be associated with high levels of sequencing errors with Illumina technology [31].

### Whole transcriptome sequencing and differential expression of precursors

The majority of plant miRNA genes are transcribed by RNA polymerase II and result in the production of polyadenylated primary transcripts [7]. A strict correlation between expression levels of individual precursors and levels of mature miRNAs should not be expected. Mature miRNAs are likely to be, in general, more stable than their corresponding primary transcripts and may derive from more than one genomic locus. Furthermore, recent data in plants [45] and animals [46] suggest that a variety of mechanisms, including alternative splicing and the specific binding of protein factors, can regulate the efficiency with which pri- or pre-miRNAs are processed. High levels of primary transcript can thus be associated with low levels of mature miRNAs and vice-versa. These considerations notwithstanding, it is reasonable to presume that sequences derived from highly expressed pri-miRNA transcripts should be represented in whole transcriptome "deep sequencing" experiments.

To investigate this hypothesis, we have analyzed whole polyA^+ ^transcriptome data generated with the Illumina Solexa technology [47] and Roche 454 next generation sequencing platforms.

A total of 135,047,735 Illumina sequences (33-35 bases in length) derived from polyA^+ ^RNA isolated from 4 tissues (*in vitro *cultivated juvenile leaf (29,829,113 sequences), *in vitro *cultivated juvenile stem (30,785,175 sequences), *in vitro *cultivated juvenile root (29,254,635 sequences) and embryonic callus (45,178,812 sequences) were mapped to the grapevine genome and coordinates compared to those for predicted pre-miRNAs.

The statistical significance of the number of reads mapping within a predicted pre-miRNA was evaluated (see Materials and Methods) and 52 predicted precursors show significant expression in at least one tissue (25 in leaf, 38 in stem, 17 in root, 33 in callus)(Table 1). Many predicted precursors show a wide expression (miR156d, miR159c, miR166a and c, miR168, miR171a, miR398a, miR398b and c, miR408, miR482). In some families, when expressed, precursors show overlapping patterns. For example, miR319c, miR319e and miR319f are all expressed in stem, while miR319c and miR319 g are expressed in callus, no expression of miR319 was detected in leaf or root. A similar situation is observed for the miR396 family. In other cases, different precursors seem to be predominantly expressed in different tissues. For example miR171e transcripts are detected only in callus, miR171f is only transcribed in stem while miR171 g is observed in callus and root - a similar situation can be observed for several families including miR166, miR167 and miR169). These data suggest that tissue specific expression of different precursors within single families is widespread in the grapevine.

454 sequencing generated 613,098 and 581,655 reads respectively from leaf and berry polyA^+ ^RNA. The expression of 15 unique predicted precursor sequences received ulterior support from these data (Table 1). With the exception of miR160b and the miR535 family the expression of all precursors detected by 454 sequencing in leaf was also strongly supported by Illumina data. Interestingly, given the lack of detectable expression of the mature sequence in leaf or berry, miR482 precursors were detected at high levels both by Illumina and 454 sequencing, suggesting post-transcriptional regulation of processing of this transcript.

### Estimation of primary microRNA transcripts and splice sites

For a number of predicted microRNAs the density of coverage of the corresponding genomic loci was sufficient to attempt to estimate primary transcript coordinates as well as patterns of splicing and alternative splicing.

We constructed Position Specific Scoring Matrices (PSSMs) of experimentally validated grapevine canonical splice donor and acceptor contexts (French-Italian Consortium for Characterization of the Grapevine Genome, unpublished data) and used these matrices to evaluate all possible canonical splice donors and acceptors from 3 kb upstream to 3 kb downstream of predicted microRNA precursors showing extensive coverage by Illumina RNA-Seq reads. The positions and flanking exonic sequences of all possible splice donor/acceptor pairs were used to combinatorially generate possible splice junctions. RNA-Seq reads which did not map perfectly to the genome sequence were compared to these computationally generated splice junctions and pairs providing perfect matches (with at least 8 bases on either side of the splice junction) were recorded along with the tissue distribution of reads supporting each splice event. Additionally, for each supported splice event, we recorded the ratio of base coverage by RNA-Seq of the flanking 40 putatively exonic bases and the coverage of flanking 40 putatively intronic bases (not including reads previously identified as covering the putative splice junction). Three fold greater coverage of exonic regions was considered as additional support for the presence of a functional splice junction. Introns inferred from mapping of 454 transcriptome reads were also recorded.

Visual examination of RNA-Seq coverage of regions upstream and downstream of miRNA precursor loci was used to provide initial estimates of transcript start and end positions. This step was complicated by the known propensity of RNA-Seq to provide uneven coverage of transcript termini - presumably due to the dynamics the nebulization step in sample preparation and to issues associated with differential recovery of fragments during sample preparation. Accordingly, we subjected the 6 kb interval centered on predicted precursors to promoter prediction analysis by TSSP-TCM [48] in order to attempt to provide support for manually identified transcription start sites. Estimated transcript coordinates, putative intron coordinates quality scores for each donor/acceptor, frequencies of splice junction-covering reads, and TSS proposed by TSSP-TCM are reported in Additional File 1: Supplemental Table S4.

**Figure 4:**
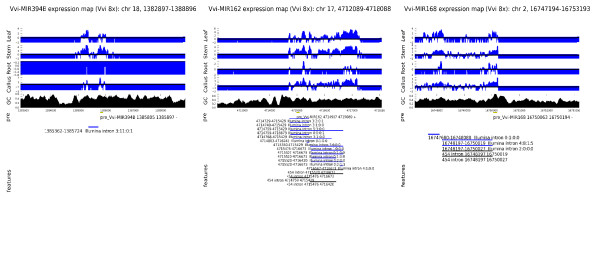
**Transcription and splicing of pri-miRNAs in *Vitis vinifera***. A summary of transcription of genomic loci containing predicted pre-miRNAs is provided. Illumina whole transcriptome reads per base are reported for four tissues as log(number of reads/expected number of reads under random distribution of reads). Local GC content, position and strand of predicted pre-miRNA as also shown along with coordinates of: canonical introns inferred from non-contiguous mapping of Illumina reads (blue bars), 454 reads (black bars) and assembled 454 sequence contigs (green bars). Predicted genes where present are represented by red bars. Panel A refers to miR394B, panel B to miR162 and panel C to miR168.

Figure 4 shows the transcriptional landscape for the genomic region from 3 Kbp upstream to 3 Kbp downstream of three exemplar predicted miRNA precursors, including introns inferred from 454 and Illumina sequence data, the concordance of splicing events identified by 454 and Illumina reads is notable and consistent with the reliability of the Illumina data to infer splicing events. Detailed genomic alignments of all reads supporting splices indicated in Figure 4 are available in Additional File 1: Supplemental Figure S5. We note that relative numbers of tags representing different regions of putative primary miRNA transcripts vary but tend to be consistent between different tissues. The GC content of 100 base windows centered on each genomic position are also shown and illustrate, within the proposed primary transcripts, an apparent correspondence between depth of coverage and GC content [31].

Figure 4A shows the transcriptional context of miR394b and the presence of a canonical intron supported by 14 Illumina reads (7 distinct sequences). This intron was also easily detectable through RACE experiments (see Additional File 1: Supplemental Table S4). We note that the position of the intron corresponds well to a region of low, or undetectable levels of Illumina transcriptome coverage, and that tissue specific differences in Illumina reads mapping to this region are quite apparent. These data suggest that our approach is capable of identifying introns in pri-miRNA transcripts and differences in steady state levels of pri-miRNA transcripts between tissues. Additionally, 3' RACE experiments indicated a transcript 3' end within 20 bp of the position predicted from RNA-Seq read coverage (see Additional File 1: Supplemental Table S4).

The miR162 precursor (Figure 4B) is of particular interest in that it covers a region including several potential canonical introns that are supported by multiple Illumina and 454 reads. All tissues indicate that the transcriptional start site falls between positions 4,714,680 - 4,714,687 on chromosome 17. The postulated canonical introns imply alternative splicing of the nascent primary transcript from this locus, as does the coverage of the region by 454 contigs (whose map positions are also consistent with the Illumina data with respect to the overall coordinates of the nascent primary transcript). Several of these introns, and the alternative splicing of this transcript were also supported by preliminary RACE experiments (see Additional File 1: Supplemental Table S4). Indeed, while the boundaries of proposed introns correspond to "shoulders" of falling transcript coverage, significant levels of reads mapping within the introns are observed. Interestingly, Hirsch et al. [45] recently demonstrated that the primary miR162a transcript of Arabidopsis is subjected to complex pattern of alternative splicing, similar to that proposed for the grapevine miR162 transcript. In Arabidopsis, only unspliced isoforms are capable of yielding mature miRNAs. Our findings therefore suggest conservation of alternative splicing as a key regulatory mechanism in miR162 expression and indicate that Illumina and 454 transcript data can also be used to identify alternatively spliced plant pri-miRNAs.

Figure 4C shows evidence for expression of the miR168 locus. Analogously to miR162, our data suggest alternative splicing of the pri-mRNA, while the distribution of 454 contigs is highly consistent with the Illumina data. Vaucheret et al. [49] showed that AGO1, the target of miR168 is involved in the regulation of miR168 stability. Our data may hint at yet another mechanism of regulation of this intriguing miRNA.

Of 25 precursor loci chosen on the basis of extensive RNA-Seq coverage (see Additional File 1: Supplemental Table S4), 18 showed evidence of transcript splicing and 8 of alternative splicing, suggesting that post-transcriptional modification of miRNA transcripts is likely to be widespread. It is possible that some splicing events frequently identified by deep sequencing approaches could be associated with regulation of downstream processing of transcripts as has been shown for the miR162 transcript of Arabidopsis [45]. For miR162 and miR168, this hypothesis might be consistent with the low levels of mature microRNA observed by deep-sequencing, in contrast to the apparently high spliced transcript levels. For several pre/pri-miRNA loci (notably miR162 and miR168) we infer several closely related canonical introns (shared splice donors with splice acceptor sites shifted by a few tens of bases or vice-versa). We speculate that this phenomenon might be due, in part, to the incapacity of the Nonsense Mediated Decay pathway (which is dependent on ribosomal scanning of mRNAs [50] to monitor "erroneous" splicing of non-coding transcripts.

The estimation of primary transcript coordinates, and in particular transcription start sites is a critical step towards the elucidation of specific mechanisms regulating the expression of miRNAs at the transcriptional level. Our Illumina transcript reads are non-directional - it is not possible to establish from which strand of the genome a transcript is derived. However, we show elsewhere that both concomitant transcription of both genomic strands at single loci and transcription of intergenic regions are rare in grapevine (French-Italian Consortium for Characterization of the Grapevine Genome, unpublished data). Thus, evidence of transcription of intergenic pre-miRNAs can reasonably be considered as validation of transcription of the precursor.

The finding that relative depth of coverage of different regions of primary transcripts is consistent between tissues suggest the presence of systematic biases in either the procedure used to fragment the cDNA, in amplification of fragments for sequencing, or in sequencing efficiency. Dohm et al. [31] observed a strong relationship between local GC content and depth of coverage with Illumina genome resequencing. Indeed, we observe a relationship between local GC content and depth of coverage - even within regions that show contiguous coverage and are unlikely to represent introns (correlation between log coverage for positions represented by at least one sequence and GC content of 100 base window centered on that position for all bases within 3 kb of a predicted precursor is >0.25 for all tissues, p = 0). However, grapevine introns between both coding and non-coding exons show a low GC content (34.7 and 32.3% respectively) with respect to coding and non-coding exons (44 and 37.3% respectively) [25]. Thus, it may be difficult to differentiate between introns and regions where low coverage is a result of low GC content in exonic regions on the basis of Illumina transcriptome data - particularly where levels of template are likely to be low and *a-priori *gene models are not available. However, the discovery that putative splice junctions for pri-miRNAs can be identified by discontiguous mapping of illumina reads may help to ameliorate this problem for plant pri-miRNAs. The fact that we recovered evidence of alternative splicing of miR162, is consistent with data from *A. thaliana *[45] and validates our basic approach. Indeed, other putative pri-miRNAs, including miR394b show evidence of splicing from both transcript coverage and discontiguous mapping of whole transcriptome reads.

## Conclusion

We have used a combination of high throughput approaches to show highly tissue specific expression of mature miRNAs in the grapevine *Vitis vinifera *including the first evidence of differential expression of miRNAs during fruit maturation in this species. We have shown that, for plants at least, whole transcriptome sequence data can be applied to the detection of differential transcription of putative precursor miRNA loci and to the detection and definition of pri-miRNAs as well as to the tentative definition of patterns of splicing in such precursors. It is probable that similar analyses performed in lines carrying mutations in genes involved in miRNA processing (in particular DCL1- plants) will allow more extensive and accurate definition of miRNA transcripts on a large scale, eventually facilitating detailed analyses of promoter sequences and a deeper understanding of mechanisms of transcriptional regulation of miRNA genes. Our analyses also suggest that splicing (and alternative splicing) of pri-miRNAs may be widespread and might constitute a general mechanism for the regulation of miRNAs.

## Methods

### Plant materials

*Grapevine (Vitis vinifera *L.) clone PN40024 plants and callus tissue were cultivated *in vitro *under standard conditions. For oligonucleotide array analyses, fresh tissues, with exception of roots (where *in vitro *cultivated plants were used), were collected from field-grown *V. vinifera *L. cv Corvina. Grape berries were harvested 5, 9 and 15 weeks after flowering, while leaves were collected from pre-flowering plants, inflorescence samples were collected 3 weeks before flowering.

### RNA extraction and deep sequencing

For Illumina deep sequencing, total RNA from PN40024 was extracted with the Spectrum Plant Total RNA Kit (SIGMA) as directed by the manufacturer. RNA was DNase treated with RQ1 RNAse-Free DNAse (Promega) and RNA integrity was checked using an Agilent Technologies 2100 Bioanalyzer.

Total RNA samples from leaf, root, stem and callus were processed using proprietary kits at Illumina, Inc. in Hayward (CA, USA). Briefly, PolyA^+ ^RNA was isolated from total RNA fragmented using Ambion RNA fragmentation buffer. cDNA synthesis was performed with Invitrogen random hexamer primers and cDNA was purified using QIAquick PCR spin column (Qiagen). Ends were blunted and 3' A overhangs introduced using T4 DNA polymerase and *E. coli *DNA polymerase I Klenow fragment. cDNAs were ligated to adapters with a single 'T' base overhang. After selection of 150-200 bp fragments from 2% low-range agarose gel, samples were amplified by 18 PCR cycles to enrich cDNAs with correctly ligated adapters and to amplify the amount of DNA in the library. Samples were loaded on Cluster Station to create flow cells of CSMA (Clonal Single Molecular Array) and sequenced on the Illumina platform. RNA-Seq data are available from http://www.genoscope.cns.fr/externe/gmorse/raw_data/.

Small RNAs (20-30 nt) were isolated from leaf total RNA by a denaturing PAGE gel. Samples were prepared for sequencing using proprietary kits at Illumina, Inc (CA, USA). Briefly, 5' and 3'-adapters were ligated to small RNAs. After reverse transcription, a low number of PCR cycles were used to create a sufficient amount of cDNA constructs. cDNA sample was then loaded on the Cluster Station and sequenced at ultra-high throughput on the Illumina platform at Illumina. Small RNA sequencing data are available from the Short Reads Archive (SRA) under accession number SRS005164.

For 454 transcriptome analysis, polyA^+ ^RNA was isolated from *V. vinifera *L. cv Corvina leaf and berry tissues by according to Rezaian and Krake [51]. After reverse transcription using an oligo(dT)-adapter primer for first strand synthesis, cDNAs were amplified with 18 (leaf) and 17 (berry) cycles of LA-PCR [52]. Normalization was carried out by one cycle of denaturation and reassociation of the cDNA. Reassociated ds-cDNA was separated from the remaining ss-cDNA (normalized cDNA) by passing the mixture over a hydroxylapatite column. After hydroxylapatite chromatography, the ss-cDNA was amplified with 9 LA-PCR cycles using phosphorylated primers. cDNAs were finally purified using the NucleoSpin ExtractII kit from Macherey & Nagel and subjected to sequencing on the ROCHE 454 GS FLX platform according to manufacturer's instructions.

For oligonucleotide array analyses, total RNA was extracted from *V. vinifera *L. cv Corvina tissues and size fractioned, following the procedure [53] with minor modifications. Before extracting RNA from berries, seeds were separated from the rest of the fruit. Low molecular weight (LMW) RNA was checked for quality and quantity using the NanoDrop Spectrometer (ND 1000, Celbio SpA) and the Agilent 2100 Bioanalyazer.

### Oligonucleotide arrays

Predicted grapevine miRNA precursor sequences have been published elsewhere (Jaillon et al., 2007). A CombiMatrix 12K CustomArray was synthesized with 1947 miRNA-specific probes synthesized to test grapevine miRNA expression profiles.

For each grapevine miRNA precursor, we designed a set of 20-22 nt probes specific for the mature miRNA, the miRNA* and their complementary sequences, as well as a probe specific for the non mature microRNA stem/loop region and probes designed on the miRNA and miRNA* sequences but shifted of five or ten nt, forward and backward in order to test probe specificity. snRNA U6 and four grapevine tRNA probes were used as positive controls. Fourteen distinct negative and mRNA degradation control probes were included. Additionally, for each specific probe, a mismatch control with 2 maximally destabilizing substitutions was included. Each probe was present on the final array in three replicates. All probe sequences are available in Additional File 2: Supplemental Table S6.

Slides were hybridized with 3 g of LMW RNA labeled with Cy5 (Mirus LabelIT miRNA labeling Kit (Mirus Bio Corp.)). Hybridization and washing were performed as indicated by CombiMatrix. Slides were scanned with a Perkin Elmer Scanarray 4000 XL raw data was extracted with Scanarray Express 4.0 and Microarray Imager (CombiMatrix) software. After each hybridization, slides were stripped according to manufacturer's instructions and re-used 5 to 6 times.

Two hybridizations were performed with independently extracted LMW RNAs, for each sample. Background level was defined as the average signal of the negative and degradation controls plus two times their standard deviation. The ratio between intensities of the perfect match probe and its mismatch probe (referred to as PM/MM) was also used to estimate the reliability of each signal. Probes with a median signal higher than background and with PM/MM value higher than 1.2 were called as present. The normalization between arrays was performed using the quantile normalization method [54] using the BLIST software, provided by Combimatrix.

Normalized signals were Log2 transformed and probes with a low PM/MM ratio (<1.2) were discarded. Differentially expressed genes in various tissues were identified with a one-way ANOVA test (*p*-value < 0.05). Significant results were further investigated with Scheffè test, a *post hoc *test to define which tissues showed significant differences.

The use of short RNA probes has not proven to be effective to distinguish between miRNAs that have few differences, in particular at the first or last nucleotide [32], therefore microarray data from closely related miRNAs have been treated as replicated data. Thus different miRNA precursors that give rise to almost identical mature products have been clustered as single entities.

Complete oligonucleotide array experimental design and data are available from the Gene Expression Omnibus [GEO: GSE13801].

### RACE experiments

Total RNA was extracted from *V. vinifera *L. cv Corvina leaves, using a rapid CTAB method, as described [55], with minor modifications, and DNAse treated (DNase I from Sigma). The FirstChoice RLM-RACE kit (Ambion) was used to perform classic 3' and 5' RACE protocols, following manufacturer's instructions. PCR products, obtained with gene specific primers (miR482-5'-Rout-CGGCATAGGATCTGAGTCCAC, miR482-5'-Rinn-GAAATCCCCGAAAACAATAGGA, miR482-3'-Fout-CGGTTTTCAGATTGGGTTATGA, miR482-3'-Finn-AGGAAGAATGGTGGATTCATTA, miR394b-5'-Rout-CCTCTTTTGTGGCTGTGAGATG, miR394b-5'-Rinn-TGAAAGAGGCAAAGAGGAGGAG, miR394b-3'-Fout-CAATCTCTCTCGCTCTTCCACT, miR394b-3'-Finn-ACATCTCACAGCCACAAAAGAG, miR162-5'-Rout-GAATTTGGCGTTGTGATGCTAC, miR162-5'-Rinn-AGAAGAACACAGGGCGGATCT, miR162-3'-Fout-AGACTCTGGTAGCATCACAACG, miR162-3'-Finn-GGTTTATCTTCCGATGGAGAAC), were subsequently cloned in pGEM-T Easy vector (Promega) and sequenced.

### Bioinformatics methods

For small RNA deep sequencing, initial reads of length 33 bases were scanned for the presence of the 5' part of the 3' linker sequence 5'-TCGTATGCCGTCTTCTGCTTG-3' allowing 2 mismatches. Raw sequences whose last 9-14 bases represented the first 9-14 bases of the 3' cloning primer were mapped to the draft genome sequence after removal of adaptor sequences using the software SOAP [56]. Whole transcriptome Illumina reads were mapped to the *Vitis vinifera *genome using the software SOAP.

Counts of reads mapping to defined genomic loci and all statistical analyses of cluster densities were performed using custom scripts written in PYTHON.

For Illumina transcriptome data, we estimate the probability that at least the observed number of reads should be clustered in the genomic interval defined by the precursor using the Poisson distribution. Thus, we exclude all reads mapping to predicted genes, and search for significant violations (at the 1% confidence interval) of the null-hypothesis that remaining reads should be distributed randomly among intergenic regions. We consider only reads mapping uniquely to a single genomic locus. Given the expected short half-life of most primary miRNA transcripts, we believe that these criteria constitute an extremely conservative test of precursor expression.

For the genomic regions containing predicted miRNA precursors, we used ad-hoc PYTHON scripts exploiting the MatPlotLib library to plot, for each base the log of the coverage (normalized to the expected coverage under a null model of random distribution). Thus only values above zero reflect higher than expected numbers of transcript-derived matches.

Python scripts were used to generate PSSMs for U2 splice donors and acceptors (3 exonic bases, 14 intronic bases) and aggregate log scores were used to assign scores to all possible canonical splice donors and acceptors. Custom PYTHON scripts were used to combinatorially generate putative splice junctions and SOAP was employed to map RNA-Seq reads that did not provide contiguous perfect matches to the genome sequence to such junctions.

454 and RACE reads were mapped to the *Vitis vinifera *L. genome sequence using preliminary Blast searches and fine mapping of splice junctions was performed using SPIDEY [57] with default settings for plant sequences. Only reads where over 85% of the read length was aligned at over 95% identity were considered.

## Authors' contributions

EM Extracted RNA for oligonucleotide arrays, performed oligonucleotide array experiments and analyses of oligonucleotide array data and contributed to drafting the manuscript. VP designed the oligonucleotide array, performed analyses of deep sequencing data and contributed to drafting the manuscript. MEP conceived the project, provided technical support and contributed to drafting of the manuscript, MD and AF performed oligonucleotide array experiments and data extraction, MP provided plant biomass, CC performed analyses of oligonucleotide data, GV provided 454 sequence data, CDF and AP provided software for mapping of splice junctions, MM provided Illumina short sequence data, GP supervised the project, provided technical support and contributed to drafting the manuscript. DSH provided software for data analyses, performed data analyses, supervised the project and contributed to drafting the manuscript. All authors have read and approved the final manuscript.

## Supplementary Material

Additional file 1 **Expression data for *Vitis vinifera *miRNAs**. **Supplemental Table S1**: Frequencies of mapped small RNA reads by size and locus. **Supplemental Figure S2**: Log expression levels of all predicted miRNAs in all tissues as detected by oligonucleotide array. **Supplemental Figure S3**: Detailed alignments and frequencies of all small RNA reads mapping to miRNA precursors. **Supplemental Table S4**: transcript data for 25 *Vitis vinifera *miRNAs. **Supplemental Figure S5**: Splice junction read coverage for Vvi-miR394b, Vvi-miR162 and Vvi-miR168. Click here for file

Additional file 2 **Oligonucleotide Array probes for analysis of Vitis vinifera miRNA expression**. **Supplemental Table S6**: All oligonucleotide array probe sequences. Click here for file
