# Supplementary material for: High throughput approaches reveal splicing of primary microRNA transcripts and tissue specific expression of mature microRNAs in Vitis vinifera
Source: BMC Genomics. 2009 Nov 25;10:558. doi: 10.1186/1471-2164-10-558 (PMC2822795; doi:10.1186/1471-2164-10-558)
Supplement: Additional file 1 — Expression data for Vitis vinifera miRNAs. Supplemental Table S1: Frequencies of mapped small RNA reads by size and locus. Supplemental Figure S2: Log expression levels of all predicted miRNAs in all tissues as detected by oligonucleotide array. Supplemental Figure S3: Detailed alignments and frequencies of all small RNA reads mapping to miRNA precursors. Supplemental Table S4: transcript data for 25 Vitis vinifera miRNAs. Supplemental Figure S5: Splice junction read coverage for Vvi-miR394b, Vvi-miR162 and Vvi-miR168. [file 1471-2164-10-558-S1.pdf]

Supplemental Table S1: Frequencies of mapped small RNA reads by size and locus.

length	# loci	# reads	mean reads/locus
19	146612	1466320	10,001364
20	212166	2464169	11,614344
21	664229	6143363	9,248863
22	376958	2474858	6,565341
23	330553	674605	2,040838
24	896304	2038037	2,273823

Supplemental Figure S2: Log Expression levels of all predicted miRNAs in all tissues tested by oligonucleotide array. Error bars represent confidence intervals. Y axis represents Log2 of the normalized median of spot intensities

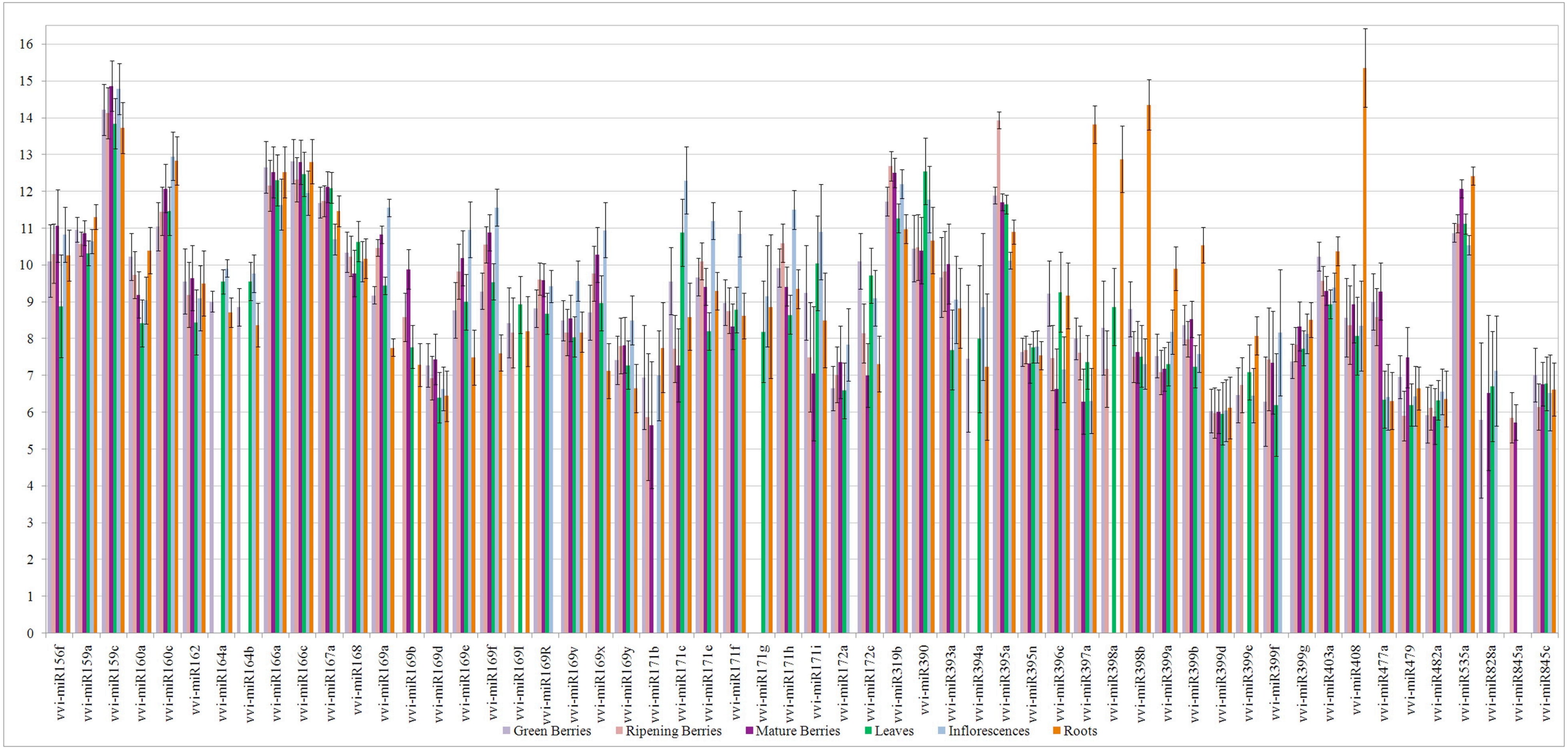

Supplemental Figure S3: Alignments of all small reads mapping to predicted pre-miRNAs and their putative secondary structures: Scaffold, and Chromosome coordinates as well as predicted secondary structures are shown for all putative microRNA precursors considered in this work. Frequencies of matching smallRNA reads aligned to precursors are shown. Intitial mature sequences predicted are shown in red, preferred designations of mature sequences are shown as underlined text.

```
__________________________________
>vvi-MIR156a [sc:66 - 769283 769386] [chr:19 - 8702104 8702207]
......(((.((((((((((((((((((((.(((((.(..(((.((((...)))))))..).)))))..))))))))))).))))))))).)))..........  (-46.90)
AGAAATATGTTGACAGAAGAGAGGGAGCACAACCCAGCATCAGCTAAAGACTCTTTGCTTTTCTTGGGAGTGTGCTCTCTCTTCTTCTGTCATCATCACACTCC
          TGACAGAAGAGAGGGAGCAC     2229
         TTGACAGAAGAGAGGGAGCAC     1119
           GACAGAAGAGAGGGAGCAC     19
          TGACAGAAGAGAGGGAGCACA    11
          TGACAGAAGAGAGGGAGCA      7
          TGACAGAAGAGAGGGAGC       5
         TTGACAGAAGAGAGGGAGC       3
         TTGACAGAAGAGAGGGAGCACAA   3
         TTGACAGAAGAGAGGGAGCA      2
           GACAGAAGAGAGGGAGCACA    2
            ACAGAAGAGAGGGAGCAC     1

__________________________________
>vvi-MIR156b [sc:6 - 5342190 5342291] [chr:4 - 5351258 5351359]
(((((.((.(((((((((((((((((((((.(((((((((..(((((....)))))..))))))))).))))))).))))).))))))))).)).)))))..    (-53.30)
CATTGAAAACTGACAGAAGAGAGTGAGCACACAGAGGCAATTGTATAAGTTTATGCTTTTGCTTTTGCGTGCTCATTTCTCTTTCTGTCAGCTTCCAGTGCC
          TGACAGAAGAGAGTGAGCAC     835876
         CTGACAGAAGAGAGTGAGCAC     9068
          TGACAGAAGAGAGTGAGCACA    4751
          TGACAGAAGAGAGTGAGCA      3092
          TGACAGAAGAGAGTGAGC       1409
           GACAGAAGAGAGTGAGCAC     475
            ACAGAAGAGAGTGAGCAC     162
         CTGACAGAAGAGAGTGAGCA      102
          TGACAGAAGAGAGTGAG        97
           GACAGAAGAGAGTGAGCACA    90
            ACAGAAGAGAGTGAGCACA    42
            ACAGAAGAGAGTGAGCACAC   30
         CTGACAGAAGAGAGTGAGC       17
       AACTGACAGAAGAGAGTGAGCAC     15
      AAACTGACAGAAGAGAGTGAGCAC     9
             CAGAAGAGAGTGAGCAC     9
        ACTGACAGAAGAGAGTGAGCAC     7
            ACAGAAGAGAGTGAGCACACA  6
```

```
         CTGACAGAAGAGAGTGAGCACA      4
          TGACAGAAGAGAGTGAGCACAC     4
         CTGACAGAAGAGAGTGA           3
         CTGACAGAAGAGAGTGAG          3
            ACAGAAGAGAGTGAGCA        2
        ACTGACAGAAGAGAGTGAGC         1
           GACAGAAGAGAGTGAGC         1
        ACTGACAGAAGAGAGTGAGCA        1
          TGACAGAAGAGAGTGAGCACACA    1
```

```
__________________________________
>vvi-MIR156c [sc:6 - 841232 841335] [chr:4 - 850300 850403]
.((((.(((((((((((((((((((((((.((.(((...(((..(((((....)))))..)))))).)).))))))))))))))).))))))))))))))....  (-60.20)
AAGGAATAGGTGACAGAAGAGAGTGAGCACACATGGTCTGTTCTTGCATGGGTCCATGCTTGAAGCTCTGCGTGCTCACTCTCTATCTGTCATCTGTCCTCTCT
          TGACAGAAGAGAGTGAGCAC       835876
          TGACAGAAGAGAGTGAGCACA      4751
          TGACAGAAGAGAGTGAGCA        3092
          TGACAGAAGAGAGTGAGC         1409
           GACAGAAGAGAGTGAGCAC       475
            ACAGAAGAGAGTGAGCAC       162
         GTGACAGAAGAGAGTGAGCAC       112
          TGACAGAAGAGAGTGAG          97
         GTGACAGAAGAGAGTGAGCA        95
           GACAGAAGAGAGTGAGCACA      90
            ACAGAAGAGAGTGAGCACA      42
            ACAGAAGAGAGTGAGCACAC     30
             CAGAAGAGAGTGAGCAC       9
        GGTGACAGAAGAGAGTGAGCAC       8
            ACAGAAGAGAGTGAGCACACA    6
          TGACAGAAGAGAGTGAGCACAC     4
         GTGACAGAAGAGAGTGAGC         2
            ACAGAAGAGAGTGAGCA        2
           GACAGAAGAGAGTGAGC         1
        GGTGACAGAAGAGAGTGAGCA        1
          TGACAGAAGAGAGTGAGCACACA    1
          TGACAGAAGAGAGTGAGCACACATGG1
```

```
__________________________________
>vvi-MIR156d [sc:14 + 5411513 5411615] [chr:11 - 5633893 5633995]
.....((..((((((((((((((((((((..((.((((....(((((....)))))...)))).))..))))))).))))).)))))))))..)).......   (-47.50)
AGAGAGAGACTGACAGAAGAGAGTGAGCACATGCAGGCAATTTGTATAAGGGTATACCTATGCCGGTGCGTGCTCACCTCTCTTTCTGTCAGCTTCAGGTGCT
          TGACAGAAGAGAGTGAGCAC       835876
         CTGACAGAAGAGAGTGAGCAC       9068
          TGACAGAAGAGAGTGAGCACA      4751
          TGACAGAAGAGAGTGAGCA        3092
          TGACAGAAGAGAGTGAGC         1409
           GACAGAAGAGAGTGAGCAC       475
            ACAGAAGAGAGTGAGCAC       162
         CTGACAGAAGAGAGTGAGCA        102
          TGACAGAAGAGAGTGAG          97
           GACAGAAGAGAGTGAGCACA      90
            ACAGAAGAGAGTGAGCACA      42
         CTGACAGAAGAGAGTGAGC         17
          TGACAGAAGAGAGTGAGCACAT     12
             CAGAAGAGAGTGAGCAC       9
        ACTGACAGAAGAGAGTGAGCAC       7
            ACAGAAGAGAGTGAGCACAT     6
      AGACTGACAGAAGAGAGTGAGCAC       5
         CTGACAGAAGAGAGTGAGCACA      4
         CTGACAGAAGAGAGTGAG          3
         CTGACAGAAGAGAGTGA           3
```

```
             ACAGAAGAGAGTGAGCA       2
            GACAGAAGAGAGTGAGC        1
           TGACAGAAGAGAGTGAGCACATGC  1
         ACTGACAGAAGAGAGTGAGCA       1
         ACTGACAGAAGAGAGTGAGC        1
        GACTGACAGAAGAGAGTGAGCAC      1


__________________________________
>vvi-MIR156e [sc:13 - 1481673 1481780] [chr:11 - 1481673 1481780]
((.(((..((((((((((.(((((((((.((.(((....(((..(((((.......)))))..)))))).)).))))))))))))..))))))))).))).))..          (-48.20)
GTATGGTAGGTGACAGAGGAGAGTGAGCACTCATGGTTCTTTTCTTGCATGAAGTTTCTATGCTTGAAGCTCTGTGTGCTTACTCCCTATCTGTCACCCCTCAAGCAT
          TGACAGAGGAGAGTGAGCAC      251
          TGACAGAGGAGAGTGAGCA       2
           GACAGAGGAGAGTGAGCAC      1


__________________________________
>vvi-MIR156f [sc:21 + 498862 498966] [chr:14 + 15664264 15664368]
....((((((((((((.(((.((((((.(((((((..((.((.((((....)))).)).)).)..))))..))).))))))..))).))))))))))))))......  (-45.40)
TTATGTTCTGTTGACAGAAGATAGAGAGCACAACTGAACATACCCAAGAGGGCTTGTGTTTGAGCAGTCTTGTCCTCTCTCTTCTCCTGTCAACAGAACTTGCTG
          TTGACAGAAGATAGAGAGCAC     30
           TGACAGAAGATAGAGAGCAC     7
           TGACAGAAGATAGAGAGCACA    2


__________________________________
>vvi-MIR156g [sc:16 + 2308172 2308274] [chr:17 - 3037544 3037646]
(((((......((((((((.(((((((((((((.(((((.(((((.......))))).))).)).)))))))))))))..)))))))))))))))........   (-50.00)
GGTGATGTTGTTGACAGAAGATAGAGAGCACAGATGATGATGTGCATGGAGTTTGCATCTCACTCCTTTGTGCTCTCTAGACTTCTGTCATCACCTCAGCCCC
          TTGACAGAAGATAGAGAGCAC     30
           TGACAGAAGATAGAGAGCAC     7
           TGACAGAAGATAGAGAGCACA    2




__________________________________
>vvi-MIR156h [sc:18 - 820816 821043] [chr:12 + 4070523 4070750]
.((..((((((((.((..((((((((((......((((.......((((((((((((((....))))..)))).).)))))))..((((.....(..(((....)))..)....))))..............))))))............(((.((((((((......(((....)))....))))..)))).))))))))))))).)..)).))))))))....)).         (-
59.90)
TGCCTCACAATGACAGAAGAGAGAGAGCATGCTGGTGGGAAAACAATTACAACTTTTGATCATCTGATCTGGAAATGCTTGTAAGCGGCATTCTCTTGGATTGTAATCTGAATTCTGCCTCTATCATCAACCTGCCCACAAACGATTTCCTTCAACTGAGTGCCTTTCCGGCTTGAGCCTTCTGCATGATCAGCTGAGTTCTTTCTGCGCCTTTCATTGTGTCCTGCC
          TGACAGAAGAGAGAGAGCAT      69
          TGACAGAAGAGAGAGAGCA       11
          TGACAGAAGAGAGAGAGC        9


__________________________________
>vvi-MIR156i [sc:167 + 235850 235952] [chr:Un_random + 36776839 36776941]
(((((......((((((((((((((((((((((.(((((.((((.........)))).))).)).)))))))))))))).))))))))))))))))........   (-49.00)
GGTGACGTTGTTGACAGAAGATAGAGAGCACAGATGATGAAATGCAAGGAGCCAGCATCTCACTCCTTTGTGCTCTCTATTCTTCTGTCATCACCTTAAGGCC
          TTGACAGAAGATAGAGAGCAC     30
           TGACAGAAGATAGAGAGCAC     7
           TGACAGAAGATAGAGAGCACA    2


__________________________________
>vvi-MIR159a [sc:40 + 1876099 1876292] [chr:15_random + 3461857 3462050]
((((.((((((((((((((.((((((((..((..(((((......((((.((((..((.(.(((.((((..((((((((((.((((((..((((......))))..))))))..)))))))))))..)))))))..).)).)..)))).)))).....))))).)).)..)))))))))))))))))))))))))))))...       (-93.20)
GGGTTTATGGGAGCTCCTTTACGCTCCAGTTCTGAAAGGAGATGATGGTATCCACAGCTGCTGGTTCATGAGTACCTATGGCTGCACAATATATATAGCAATGTTGTGTGCAAACTATGGGTGTGCATGACCTCGGAGAAGTGGTTGCCTTGATCTTTCTGGCCTTGGAGTGAAGGGAGCTCTCATAACCCTTC
                                                                                                                                                                             CTTGGAGTGAAGGGAGCTCTC     0


__________________________________
>vvi-MIR159b [sc:40 + 1873406 1873590] [chr:15_random + 3459164 3459348]
.((.(((((((((((((((.((((((((...((((((.(((((((.((.(.((((((((.((((((((((.....((........)))..((((....)))))))))))))))).)))))))).).)).))))))).)))))).(......)...)))))))))))))))))))))))))))))....    (-75.20)
GGGCTTGTGGGAGCTTCTTTACACTCCAGAACTGAAAGGAGATACTACTGCTGGTTCATGAAAACCTATGGCTGCATTCTCTATACTGAAATTTTGTATACAAACTATGGGTTTGCATGAACTTGGAGAAGTGTTTGCTTTTATCTTTTCTGGCCTTGGAGTGAAGGGAGCTCTCATAACCGATC
                                                                                                                                                                    CTTGGAGTGAAGGGAGCTCTC   0


__________________________________
>vvi-MIR159c [sc:16 + 2735981 2736170] [chr:17 - 2609648 2609837]
....((.((((((((((((..(((((((..((((((((.(((((((((..((((((.((((..((((.(..((((((.(((((((.(((.......)))...))))))).)))))).).)))).))))..)))).)))).)))))))).))).)..)))))))).)))..)))))))..)))))))..)))))))))))).)).......            (-92.40)
TTTTGGAGTGGAGCTCCTTGAAGTCCAATAGAGGGTCTTACTGGGTAGATTGAGCTGCTGACTTATGGATCCCACAGCCCTATCCCGTCAATGGGGGGCATTGGATAGGCTTGTGGCTTGCATATCTCAGGAGCTGCATTATCCAAGTTTAGATCCTTGTTTGGATTGAAGGGAGCTCTACACCTCTCTC
                                                                                                                                                                 TTTGGATTGAAGGGAGC              2305
                                                                                                                                                                 TTTGGATTGAAGGGAGCT             646
                                                                                                                                                                 TTTGGATTGAAGGGAGCTC            518
                                                                                                                                                                 TTTGGATTGAAGGGAGCTCT           124
                                                                                                                                                                 TTTGGATTGAAGGGAGCTCTA          30
                                                                                                                                                                  TTGGATTGAAGGGAGCTC            17
                                                                                                                                                                  TTGGATTGAAGGGAGCTCT           12
                                                                                                                                                                  TTGGATTGAAGGGAGCTCTA          4
                                                                                                                                                                   TGGATTGAAGGGAGCTC            1
                                                                                                                                                                  TTGGATTGAAGGGAGCT             1
                                                                                                                                                                 TTTGGATTGAAGGGAGCTCTACA        1
                                                                                                                                                                 TTTGGATTGAAGGGAGCTCTAC         1
                                                                                                                                                                GTTTGGATTGAAGGGAGCTCT           1
                                                                                                                                                                GTTTGGATTGAAGGGAGCTC            1
                                                                                                                                                               TGTTTGGATTGAAGGGAGC              1
                                                  AGCTGCTGACTTATGGATCC          1

__________________________________
```

```
>vvi-MIR160a [sc:47 + 660403 660505] [chr:12 + 8539030 8539132]
((((....((((.(((((((((((.(((((.(((.((((...(((((.....)))))...)))).))).))))))))).))))))).)))))))))......    (-52.30)
GGGGTTGATCTGCCTGGCTCCCTGAATGCCATCTAAGAAGCTTGTCAAGGTTGTTGACTCTCTTCCTAGTTGGCATCAGAGGAGTCATGCAGGCCCCAACATT
          TGCCTGGCTCCCTGAATGCCA    276
          TGCCTGGCTCCCTGAATGC      4
             CTGGCTCCCTGAATGCCA    2

__________________________________
>vvi-MIR160b [sc:383 + 36508 36611] [chr:Un_random + 78660926 78661029]
....((((.((((.(((((((((..((((((..((.((((.(((((((......))))))).)))).))..))))))..))))))))).))))))))).......  (-47.00)
AATATTGTTCTGCCTGGCTCCCTGAATGCCATCTAAGAAGCTTGTTAAAGAGTGTTGACAACCTTCCTATTTGGCATGAGGGGAGTCAAGCAGGCGAAACACTT
          TGCCTGGCTCCCTGAATGCCA    276
          TGCCTGGCTCCCTGAATGC      4
             CTGGCTCCCTGAATGCCA    2

__________________________________
>vvi-MIR160c [sc:76 + 752851 752956] [chr:10 - 8894085 8894190]
...(((((.((((.(((((((.((((((((((.((((((..(((.(((.......))).)))...)))))).)))))))))).))))))).)))))))))......(-58.30)
AAATGGGTTGTGCCTGGCTCCCTGTATGCCACACGTAGAGACCACTCCCTTTCCCGGATTGGCTATTTGCGGGTGGCGTGCGAGGAGCCAAGCATACCCACCATTC
          TGCCTGGCTCCCTGTATGCCA    130
             CTGGCTCCCTGTATGCCA    1

__________________________________
>vvi-MIR160d [sc:29 - 1349764 1349865] [chr:8 + 11902361 11902462]
........(((((.(((((((.((((((((((((((.(((.(.(((........))).).))).)))))))))))))).))))))).))))).........    (-49.30)
CATACTTACATGCCTGGCTCCCTGTATGCCATTTGCAAAGTCCACCCGAGGATTGGTAGCCTTCGCGGATGGCGTGCGAGGAGCCATGCATGCATCTTGCTT
          TGCCTGGCTCCCTGTATGCCA    130
         ATGCCTGGCTCCCTGTATGCCA    2
             CTGGCTCCCTGTATGCCA    1
          TGCCTGGCTCCCTGTATGCCAT   1



__________________________________
>vvi-MIR160e [sc:1809 - 11818 11921] [scaffold not incorporated in genome assembly]
....((((.((((.(((((((((..((((((..((.((((.(((((((......))))))).)))).))..))))))..))))))))).))))))))).......  (-47.00)
AATATTGTTCTGCCTGGCTCCCTGAATGCCATCTAAGAAGCTTGTTAAAGAGTGTTGACAACCTTCCTATTTGGCATGAGGGGAGTCAAGCAGGCGAAACACTT
          TGCCTGGCTCCCTGAATGCCA    276
          TGCCTGGCTCCCTGAATGC      4
             CTGGCTCCCTGAATGCCA    2

__________________________________
>vvi-MIR160f [sc:17 - 2195098 2195199] [chr:13 + 5553097 5553198]
...(((((..(((.(((((((..((((((((((..(..((.(((.(((....))).))).))..)..))))))))))..))))))).))))))))).......    (-51.10)
TAAGCATATATGCCTGGCTCCCTGTATGCCATTTGCAGAGCCCACCGGCACATCGATGGCCTTCGTGGATGGCGTATGAGGAGCCATGCATATGCCCCATCT
          TGCCTGGCTCCCTGTATGCCA    130
         ATGCCTGGCTCCCTGTATGCCA    2
             CTGGCTCCCTGTATGCCA    1
          TGCCTGGCTCCCTGTATGCCAT   1

__________________________________
>vvi-MIR162 [sc:16 - 630729 630831] [chr:17 + 4714987 4715089]
((((..(.((((((((((.((((.((((((((..(((.(((..(((((........))))).))).)))..)))))))).)))).)))))))))))).))))))....    (-41.70)
TGAAGTCACTGGATGCAGCGGTTCATCGATCTCTTCCTGAAATTGTTGTGAAAAAAGCAGATCAAGAATCGGTCGATAAACCTCTGCATCCAGCGTTCACTCC
                                                                          TCGATAAACCTCTGCATCCAG  1

__________________________________
>vvi-MIR164a [sc:42 - 451600 451720] [chr:7 - 3651395 3651515]
(((.((.((((((((((((..((((((((((.((......))...................................(((....)))..))))))))))..))))))))))))).)))))...         (-45.00)
AGCTCCTTGTTGGAGAAGCAGGGCACGTGCAGATTTGCCTCACTTTTCCCCCTTTTTTCTTCTTTACTCCCACCACCGCCCACAGGCTTGCACGTGCTCCCCTTCTCCAACATGGGTTCCT
          TGGAGAAGCAGGGCACGTGCA    13841
          TGGAGAAGCAGGGCACGTGC     99
          TGGAGAAGCAGGGCACGTG      19
          TGGAGAAGCAGGGCACG        13
           GGAGAAGCAGGGCACGTGCA    8
          TGGAGAAGCAGGGCACGT       5
        GTTGGAGAAGCAGGGCACGTGCA    1
            GAGAAGCAGGGCACGTGCA    1

__________________________________
>vvi-MIR164b [sc:7 - 511008 511100] [chr:9 - 511008 511100]
....(((.(..(((((..((((((((((((..(((((..((((.........))))))))))..))))))))))))..)))))..).)))......  (-38.80)
TAAACCATGCTGGAGAAGCAGGGCACATGCTGGATCAATCAGCACCTGGATCTGAGATTTTACATGTGCCCTGGCTCTCCCACTTGGATCCTA
          TGGAGAAGCAGGGCACATGC     1
          TGGAGAAGCAGGGCACATGCT    1

__________________________________
>vvi-MIR164c [sc:88 + 655651 655747] [chr:8 + 8981162 8981258]
...((((((..((((..(((.(...).)))....(((((((..(((...................)))..))))))).))))..))))))).......          (-25.61)
TTGAGCAAGATGGAGAAGCAGGGCACGTGCATTACTAGCTCATGCACCACAAACCAATAATCTTTTGCTCGAGTTAGACTCTTGCTTGCTGCTTTGA
          TGGAGAAGCAGGGCACGTGCA    13841
          TGGAGAAGCAGGGCACGTGC     99
          TGGAGAAGCAGGGCACGTGCAT   20
          TGGAGAAGCAGGGCACGTG      19
```

```
          TGGAGAAGCAGGGCACG        13
           GGAGAAGCAGGGCACGTGCA    8
          TGGAGAAGCAGGGCACGT       5
         ATGGAGAAGCAGGGCACGTGCA    3
           GGAGAAGCAGGGCACGTGCAT   2
            GAGAAGCAGGGCACGTGCA    1

>vvi-MIR164d [sc:27 - 1404604 1404721] [chr:14 - 1404604 1404721]
.((((.((.((((((((..((((((((((((..((............((..((((....))))..))............))..))))))))))))..)))))))).)).)))))...            (-48.95)
AAGCTCTTGATGGAGAAGCAGGGCACGTGCAGTTCACAAATTCTAATCTGCTCTGCACGTGCAGTTCACAAATTCTAATCTGCTCTGCACGTGCTCCCCTTCTCCAACATGGGTTCCT
          TGGAGAAGCAGGGCACGTGCA    13841
          TGGAGAAGCAGGGCACGTGC     99
          TGGAGAAGCAGGGCACGTG      19
          TGGAGAAGCAGGGCACG        13
           GGAGAAGCAGGGCACGTGCA    8
          TGGAGAAGCAGGGCACGT       5
         ATGGAGAAGCAGGGCACGTGCA    3
            GAGAAGCAGGGCACGTGCA    1

>vvi-MIR166a [sc:41 - 494693 494822] [chr:8 - 3340277 3340406]
((.((((((.(((((((((((((.(((.(((((............................((((((.((((.........)))).))))))).))))).))).)))))))))))))).)))))))..)).            (-45.41)
GCGGGTGTTTGGAATGAGGTTTGATCCAAGATCCTTCCTTTTTCTCCATTAATTCCTTCAGTACTGGTCATGATTTAGTTACTTCATAATCGGTTGATCTCGGACCAGGCTTCATTCCTGACACCAAGCT
                                                                                                   TCGGACCAGGCTTCATTCC            1496
                                                                                                   TCGGACCAGGCTTCATTC             529
                                                                                                   TCGGACCAGGCTTCATTCCT           181
                                                                                                   TCGGACCAGGCTTCATT              118
                                                                                                 TCTCGGACCAGGCTTCATTCC            16
                                                                                                     GGACCAGGCTTCATTCC            8
                                                                                                   TCGGACCAGGCTTCATTCCTG          8
                                                                                                    CGGACCAGGCTTCATTCC            2
                                                                                                  CTCGGACCAGGCTTCATTC             1
                                                                                                    CGGACCAGGCTTCATTC             1
                                                                                                  CTCGGACCAGGCTTCATTCC            1
                                                                                                     GGACCAGGCTTCATTCCTG          1

>vvi-MIR166b [sc:38 - 1125312 1125410] [chr:12 + 15726184 15726282]
((..(((((((((((((..(.((((..(((.((.(((((...................))))).)).)))..)))).)..)))))))))))))...)).       (-42.41)
GTTTTTTGAGGGGAATGTTGGCTGGCTCGAGGCATTCACATAAGAAGAAGATATATAAGTGAAGGTGTCGGACCAGGCTTCATTCCTCTCAAATATACA
                                                                   TCGGACCAGGCTTCATTCC        1496
                                                                   TCGGACCAGGCTTCATTC         529
                                                                   TCGGACCAGGCTTCATTCCT       181
                                                                   TCGGACCAGGCTTCATTCCTC      165
                                                                   TCGGACCAGGCTTCATT          118
                                                                     GGACCAGGCTTCATTCC        8
                                                                    CGGACCAGGCTTCATTCC        2
                                                                    CGGACCAGGCTTCATTC         1
                                                                     GGACCAGGCTTCATTCCTC      1
                                                                   TCGGACCAGGCTTCATTCCTCT     1

>vvi-MIR166c [sc:37 + 128461 128620] [chr:15 - 7564994 7565153]
.....(((.((((((((..((((((..(((.((((((...........((((..(..((....))..)..)))))).....((((((((......((((........))))...)))))))).))))))).)))..))))))..)))))))).))).......     (-52.60)
CTATTTTGAGGGGAATGTTGTCTGGCTCGAGGACACTTACCTTAACTTTGATCCGTTTCTCTAGATCCATATCACCTATATATATGTATTATTGTATTTTTATTTGTACTGAGCATGTGTTGGTGTCGTCGGACCAGGCTTCATTCCCCCAATTATCGC
                                                                                                                                 TCGGACCAGGCTTCATTCCCC               219273
                                                                                                                                 TCGGACCAGGCTTCATTCC                 1496
                                                                                                                                 TCGGACCAGGCTTCATTCCC                1281
                                                                                                                                   GGACCAGGCTTCATTCCCC               791
                                                                                                                                 TCGGACCAGGCTTCATTC                  529
                                                                                                                                  CGGACCAGGCTTCATTCCCC               128
                                                                                                                                 TCGGACCAGGCTTCATT                   118
                                                                                                                                   GGACCAGGCTTCATTCCC                103
                                                                                                                                 TCGGACCAGGCTTCATTCCCCC              89
    TTTGAGGGGAATGTTGTCTGG          51
                                                                                                                                  CGGACCAGGCTTCATTCCC                10
                                                                                                                                   GGACCAGGCTTCATTCC                 8
                                                                                                                                GTCGGACCAGGCTTCATTCCC                5
                                                                                                                                  CGGACCAGGCTTCATTCC                 2
                                                                                                                                 TCGGACCAGGCTTCATTCCCCCC             2
```

```
                                                                                                       GACCAGGCTTCATTCCCC                1
                                                                                                     CGGACCAGGCTTCATTC1
                                                                                                    TCGGACCAGGCTTCATTCCCCCCAAT           1
                                                                                                    TCGGACCAGGCTTCATTCCCCCCA             1
          GGAATGTTGTCTGGCTCGAGG   1
       AGGGGAATGTTGTCTGGCTC       1
      GAGGGGAATGTTGTCTGGCTC       1
```

```
__________________________________
>vvi-MIR166d [sc:86 - 899379 899538] [chr:16 - 7520920 7521079]
.....((((((((.((..((((((..(((((.(((.((((...(((((.....))))).....(((((((....................................))))))).).))).))).))))).)))))).)).))))))))))).......     (-50.99)
CTGCTTTGAGGGGATTGTTGTCTGGCTCGAGGCCACCAACCATCTTTGATTGATTCAAATACTTTGATCAGCTTCAACCCATCTTCTCCTTCAATTCAATTCCACCCTCTGATTATGTGTTAGTGCCCTCGGACCAGGCTTCATTCCCCTCAATCACTGC
                                                                                                    TCGGACCAGGCTTCATTCCCC    219273
                                                                                                    TCGGACCAGGCTTCATTCC      1496
                                                                                                    TCGGACCAGGCTTCATTCCC     1281
                                                                                                      GGACCAGGCTTCATTCCCC    791
                                                                                                    TCGGACCAGGCTTCATTC       529
                                                                                                    TCGGACCAGGCTTCATTCCCCT   180
                                                                                                     CGGACCAGGCTTCATTCCCC    128
                                                                                                    TCGGACCAGGCTTCATT        118
                                                                                                   CTCGGACCAGGCTTCATTCCCC    118
                                                                                                      GGACCAGGCTTCATTCCC     103
                                                                                                   CTCGGACCAGGCTTCATTCCC     38
                                                                                                     CGGACCAGGCTTCATTCCC     10
                                                                                                      GGACCAGGCTTCATTCC      8
                                                                                                     CGGACCAGGCTTCATTCCCCT   7
                                                                                                  CCTCGGACCAGGCTTCATTCC      5
                                                                                                     CGGACCAGGCTTCATTCC      2
                                                                                                  CCTCGGACCAGGCTTCATTCCCC    2
                                                                                                   CTCGGACCAGGCTTCATTC       1
                                                                                                     CGGACCAGGCTTCATTC       1
                                                                                                   CTCGGACCAGGCTTCATTCC      1
                                                                                                       GACCAGGCTTCATTCCCC    1
                                                                                                      GGACCAGGCTTCATTCCCCT   1
```

```
__________________________________
>vvi-MIR166e [sc:105 - 1099967 1100132] [chr:2 + 2180274 2180439]
(((..(((.((((((((..((((((..(((.(((((((((.....((((((((.(((......)))........(((((.(((..((((((......))))))..))))))))...)))))))))))))))))).)))..))))))..)))))))).)))..)))..           (-71.80)
GTGTTTTGAGGGGAATGTTGTCTGGCTCGAGGACACCAACTAGATCTATGATCTGCGTGTAATTGTGAATGGGTGATCGTCTCTCAGTTTTGAAGGAGAGATTTTGAGTGATCTTTGATCATGGGTTGGTGTCGTCGGACCAGGCTTCATTCCCCCCAATTTATTG

                                                                                                           TCGGACCAGGCTTCATTCCCC         219273
                                                                                                           TCGGACCAGGCTTCATTCC           1496
                                                                                                           TCGGACCAGGCTTCATTCCC          1281
                                                                                                             GGACCAGGCTTCATTCCCC         791
                                                                                                           TCGGACCAGGCTTCATTC            529
                                                                                                            CGGACCAGGCTTCATTCCCC         128
                                                                                                           TCGGACCAGGCTTCATT             118
                                                                                                             GGACCAGGCTTCATTCCC          103
                                                                                                           TCGGACCAGGCTTCATTCCCCC        89
    TTTGAGGGGAATGTTGTCTGG          51

                                                                                                            CGGACCAGGCTTCATTCCC          10
                                                                                                             GGACCAGGCTTCATTCC           8
                                                                                                          GTCGGACCAGGCTTCATTCCC          5
                                                                                                            CGGACCAGGCTTCATTCC           2
                                                                                                           TCGGACCAGGCTTCATTCCCCCC       2
      GAGGGGAATGTTGTCTGGCTC       1
       AGGGGAATGTTGTCTGGCTC       1
          GGAATGTTGTCTGGCTCGAGG   1

                                                                                                            CGGACCAGGCTTCATTC            1
                                                                                                              GACCAGGCTTCATTCCCC         1
                                                                                                           TCGGACCAGGCTTCATTCCCCCCA      1
                                                                                                           TCGGACCAGGCTTCATTCCCCCCAAT    1
```

```
__________________________________
>vvi-MIR166f [sc:20 - 663678 663777] [chr:7 + 14569971 14570070]
(((.((((.((((((((..(.((((..(((((((((.(((((..((.......))..))))))))))))))..)))).)..)))))))).)))))))...      (-49.50)
TGTAGTTGTGGGGAATGTTGGCTGGCTCGAGGCTTTCTTGAGTGATTTTATGTATACCTCAAAAAGCTTCGGACCAGGCTTCATTCCCCTCAACACACTT
                                                                     TCGGACCAGGCTTCATTCCCC      219273
                                                                    TTCGGACCAGGCTTCATTCCC       44209
                                                                     TCGGACCAGGCTTCATTCC        1496
                                                                     TCGGACCAGGCTTCATTCCC       1281
                                                                       GGACCAGGCTTCATTCCCC      791
                                                                     TCGGACCAGGCTTCATTC         529
                                                                    TTCGGACCAGGCTTCATTCC        237
                                                                     TCGGACCAGGCTTCATTCCCCT     180
                                                                    TTCGGACCAGGCTTCATTC         170
                                                                      CGGACCAGGCTTCATTCCCC      128
                                                                     TCGGACCAGGCTTCATT          118
                                                                       GGACCAGGCTTCATTCCC       103
                                                                    TTCGGACCAGGCTTCAT           47
                                                                    TTCGGACCAGGCTTCATT          27
                                                                   CTTCGGACCAGGCTTCATTCCC       22
                                                                      CGGACCAGGCTTCATTCCC       10
                                                                    TTCGGACCAGGCTTCATTCCCC      9
                                                                       GGACCAGGCTTCATTCC        8
```

```
                                                                     CGGACCAGGCTTCATTCCCCT    7
                                                                  CTTCGGACCAGGCTTCATTCC       3
                                                                     CGGACCAGGCTTCATTCC       2
                                                                     CGGACCAGGCTTCATTC        1
                                                                  CTTCGGACCAGGCTTCATTCCCC     1
                                                                       GACCAGGCTTCATTCCCC     1
                                                                      GGACCAGGCTTCATTCCCCT    1
                                                                    TCGGACCAGGCTTCATTCCCCTC   1
```

```
__________________________________
>vvi-MIR166g [sc:31 - 2931810 2931909] [chr:7 + 257887 257986]
....((((((((((((..((((((((((((((((.((((.(((..((....))..))).)))).))))))))))))))))..))))))))))))......      (-57.00)
AACAGTTGAGGGGAATGTTGTCTGGTTCGAGATCCTTCGTATGAAGCTCAAGCAGCGTTTGAATGATTTCGGACCAGGCTTCATTCCCCTCAACCCAAAA
                                                                    TCGGACCAGGCTTCATTCCCC     219273
                                                                   TTCGGACCAGGCTTCATTCCC      44209
                                                                    TCGGACCAGGCTTCATTCC       1496
                                                                    TCGGACCAGGCTTCATTCCC      1281
                                                                      GGACCAGGCTTCATTCCCC     791
                                                                    TCGGACCAGGCTTCATTC        529
                                                                   TTCGGACCAGGCTTCATTCC       237
                                                                    TCGGACCAGGCTTCATTCCCCT    180
                                                                   TTCGGACCAGGCTTCATTC        170
                                                                     CGGACCAGGCTTCATTCCCC     128
                                                                  TTTCGGACCAGGCTTCATTCC       122
                                                                    TCGGACCAGGCTTCATT         118
                                                                      GGACCAGGCTTCATTCCC      103
                                                                   TTCGGACCAGGCTTCAT          47
                                                                   TTCGGACCAGGCTTCATT         27
                                                                     CGGACCAGGCTTCATTCCC      10
                                                                   TTCGGACCAGGCTTCATTCCCC     9
                                                                      GGACCAGGCTTCATTCC       8
                                                                     CGGACCAGGCTTCATTCCCCT    7
                                                                  TTTCGGACCAGGCTTCATT         2
                                                                     CGGACCAGGCTTCATTCC       2
                                                                  TTTCGGACCAGGCTTCATTCCC      2
                                                                  TTTCGGACCAGGCTTCAT          1
                                                                  TTTCGGACCAGGCTTCATTC        1
                                                                     CGGACCAGGCTTCATTC        1
                                                                       GACCAGGCTTCATTCCCC     1
                                                                      GGACCAGGCTTCATTCCCCT    1
                                                                    TCGGACCAGGCTTCATTCCCCTC   1
```

```
__________________________________
>vvi-MIR166h [sc:2 - 4521489 4521588] [chr:5 - 6593621 6593720]
...(((((.((((((.(..(((((((((((((.((...((.....)).((((.....))))..)).)))))))))))))..).)))))).)))))..... (-42.50)
AACAGTTGAGGGGAACGCTGTCTGGTTCGAGACCACTCGCTTCAAGTACAGACATAATCTGAATGATCTCGGACCAGGCTTCATTCCCCCCAACTCAACA
                                                                    TCGGACCAGGCTTCATTCCCC     219273
                                                                    TCGGACCAGGCTTCATTCC       1496
                                                                    TCGGACCAGGCTTCATTCCC      1281
                                                                      GGACCAGGCTTCATTCCCC     791
                                                                    TCGGACCAGGCTTCATTC        529
                                                                     CGGACCAGGCTTCATTCCCC     128
                                                                    TCGGACCAGGCTTCATT         118
                                                                   CTCGGACCAGGCTTCATTCCCC     118
                                                                      GGACCAGGCTTCATTCCC      103
                                                                    TCGGACCAGGCTTCATTCCCCC    89
                                                                   CTCGGACCAGGCTTCATTCCC      38
                                                                  TCTCGGACCAGGCTTCATTCC       16
                                                                     CGGACCAGGCTTCATTCCC      10
                                                                      GGACCAGGCTTCATTCC       8
```

```
                                                     ATCTCGGACCAGGCTTCATTCCCC      5
                                                         CGGACCAGGCTTCATTCC        2
                                                       TCGGACCAGGCTTCATTCCCCCC     2
                                                      CTCGGACCAGGCTTCATTC          1
                                                         CGGACCAGGCTTCATTC         1
                                                      CTCGGACCAGGCTTCATTCC         1
                                                           GACCAGGCTTCATTCCCC      1
                                                       TCGGACCAGGCTTCATTCCCCCCA    1
                                                       TCGGACCAGGCTTCATTCCCCCCAAC  1

__________________________________
>vvi-MIR167a [sc:5 + 1563504 1563854] [chr:1 + 1563504 1563854]
..((((.((((((((((((.((.((((((((((.(.......(((..(((...((..((.((((((.....((((((.(....).))))))).....(((((((..(((((((((.((((((..(((((((((.........)))).....)))))).....))))))....(((.......))))......)))))))))...(((....))).))))))).................
..)))))))..))...))..)))..)))......................((((.(((....))).))))......).)))))))))))).)))))))))))).)))).....     (-112.70)
TTTGAGAGGTTGAAGCTGCCAGCATGATCTGGTGAAACAAACACCATCTCTTTCTTCTCTAACCCCATGTCTGGATTCGTCCACCGATCCATTATTATAGACCAGGCCGCCCGTTTCCCATGTAGTGATCGATAATTAGGCTCGGGGTTTTCACTTTTTAGTGGGATCTAATCCTTAGGATGGATGTTTGTATGGGTGGTATATATCATGGTGAGGTCTGTTTTCTATTTTAATTCTA
ACGGGGTTTTGATTTAGCTGAGGGGGTATAATTCATAGCCTAATTCCAAAACCTAACTCCATAGAGATAGGGTTCCATGATCAGGTCATCTTGCAGCTTCAATCACTCACTCA
          TGAAGCTGCCAGCATGATC       256
          TGAAGCTGCCAGCATGA         205
          TGAAGCTGCCAGCATGATCTG     166
          TGAAGCTGCCAGCATGAT        137
          TGAAGCTGCCAGCATGATCT      89
          TGAAGCTGCCAGCATGATCTGG    50

__________________________________
>vvi-MIR167b [sc:54 + 1827200 1827298] [chr:14 + 6802135 6802233]
((.((((((.((((((((((.(((((((((..((((((((................))))))))..))))))))))))))))))))).)))))).))..        (-47.69)
CAATAGCAGTTGAAGCTGCCAGCATGATCTAAGCTTTTCTGTTGCCCACCCTTTCTCCAGGAAAGACTAGATCATGTGGCAGTTTCACCTGTTGATGGA
          TGAAGCTGCCAGCATGATCTA     4965
          TGAAGCTGCCAGCATGATC       256
          TGAAGCTGCCAGCATGA         205
          TGAAGCTGCCAGCATGAT        137
          TGAAGCTGCCAGCATGATCT      89
          TGAAGCTGCCAGCATGATCTAA    49
           GAAGCTGCCAGCATGATCTAA    1
           GAAGCTGCCAGCATGATCTA     1

__________________________________
>vvi-MIR167c [sc:31 + 2349952 2350042] [chr:7 - 839754 839844]
((.((((((.(((.(((((((.((((((((...(((.(((..........))).)))...))))))))))))))).))).)))))).))..    (-37.00)
CAGTAGCAGTTGAAGCTGCCAGCATGATCTCAACTTCCCTATACAAGTCAAGGAAAGATCAGATCATGTGGTAGCCTCACCTGTTGATGGG
          TGAAGCTGCCAGCATGATC       256
          TGAAGCTGCCAGCATGA         205
          TGAAGCTGCCAGCATGAT        137
          TGAAGCTGCCAGCATGATCT      89
          TGAAGCTGCCAGCATGATCTCA    77
          TGAAGCTGCCAGCATGATCTC     19
          TGAAGCTGCCAGCATGATCTCAA   2
           GAAGCTGCCAGCATGATCTCA    1

__________________________________
>vvi-MIR167d [sc:31 + 2344759 2344873] [chr:7 - 844923 845037]
((((((..(((((.((((.(((((((((((((((((((((...(((................)))..))))))....)))))))))))))..)).)))).)))))..)))))).. (-46.79)
AGGGAATAAGTGAAGCTGCCAGCATGATCTAGCTTTGGCTAGGGATACAGAGAAAGAGAGAGATCAGAGCTAACCCTAGCTAGGTCATGCCCTGACAGCCTCACTCCTTCCTTCT
          TGAAGCTGCCAGCATGATCTA     4965
          TGAAGCTGCCAGCATGATC       256
          TGAAGCTGCCAGCATGA         205
          TGAAGCTGCCAGCATGAT        137
          TGAAGCTGCCAGCATGATCT      89
           GAAGCTGCCAGCATGATCTA     1

__________________________________
>vvi-MIR167e [sc:2 + 3637629 3637718] [chr:5 + 5709761 5709850]
.((((.(((.((((((((((((.((((((((............(((((....)))))...)))))))))))))))))))).))).....))))     (-36.06)
CACTATCAGTTGAAGCTGCCAGCATGATCTAAACTTGCTTCCCTTTGTGAACAGAGATCAGATCATGTGGCAGTTTCACCTGTTGTTGGT
          TGAAGCTGCCAGCATGATCTA     4965
          TGAAGCTGCCAGCATGATC       256
          TGAAGCTGCCAGCATGA         205
          TGAAGCTGCCAGCATGAT        137
          TGAAGCTGCCAGCATGATCT      89
          TGAAGCTGCCAGCATGATCTAA    49
          TGAAGCTGCCAGCATGATCTAAA   32
           GAAGCTGCCAGCATGATCTAA    1
           GAAGCTGCCAGCATGATCTA     1
```

```
>vvi-MIR168 [sc:113 + 853207 853338] [chr:2 - 16750063 16750194]
.(((((..(((((.((((((((((.(((((.(.((.(((((..(((((((...)))))))).....((((((((.......)))))))).))))).)).).))))).)))))))))).))))).)))))...          (-72.40)
GGTCTCTAATTCGCTTGGTGCAGGTCGGGAACCGACTTCGCCGCTCCGGCAGCGCCGGAGGCACGCGGCGGCCTACGATTGGTTGCTGAGCGAATTCCGATCCCGCCTTGCATCAACTGAATCGGAGACGGC
          TCGCTTGGTGCAGGTCGGGAA     5
          TCGCTTGGTGCAGGTCGGGA      2

>vvi-MIR169a [sc:118 - 461597 461720] [chr:11_random - 1209766 1209889]
((((((.(((((.(((((((((..(((((((((..(((((((((((((((.........(((.....)))))))).))))).))))))..)))))))))..)))))))))).))))).)))))))..      (-61.10)
GAGAGTGGAATGCAGCCAAGGATGACTTGCCGGAATTTATATATAGTGAGAAGAGAAGAGGCTATATGCTTTCACATATATAGTGAACCCTGGCAAGTTGTCCTTGGCTACATTTGATTCTCTT
            CAGCCAAGGATGACTTGCCGG   675
            CAGCCAAGGATGACTTGCCG    26
                                                                                             GGCAAGTTGTCCTTGGCTAC       15
 AGAGTGGAATGCAGCCAAGGA              13
             AGCCAAGGATGACTTGCCGG   8
            CAGCCAAGGATGACTTGCCGGAA 8
            CAGCCAAGGATGACTTG       2
            CAGCCAAGGATGACTTGCCGGA  2
            CAGCCAAGGATGACTTGC      1
                                                                                             GGCAAGTTGTCCTTGGCTA        1
                                                                                              GCAAGTTGTCCTTGGCTAC       1
                                                                                             GGCAAGTTGTCCTTGGCTACA      1

>vvi-MIR169b [sc:118 - 310296 310400] [chr:11_random - 1058465 1058569]
((((((((((.(((((((((((((((.((((((((.(((((.((((((((...)))))))..))))).))).))))).)))))))))))))))))))))))))...  (-61.20)
GGGGTCGAATTGAGCCAAGGATGGCTTGCCGTCATCTGCAGCAAGAGTTGGAGACTTTTAACTGTGCGTGCACGGTTAGTCATCCTTGGCTCATTTGGCCCTTCT
          TGAGCCAAGGATGGCTTGCCG     0

>vvi-MIR169c [sc:6 - 2254966 2255055] [chr:4 - 2264034 2264123]
((((((..((((.(((((((((..((((((((((((((((((....))))))))..))))))))))..))))))))).)))))..)))))))..     (-60.00)
GGGAGTAGAATGCAGCCAAGGATGACTTGCCGGAGATGGGGCATTCCTCGTTAATCCGGCAAGTTGTCCTTGGCTACATTGGGCTCTCTT
            CAGCCAAGGATGACTTGCCGG   675
            CAGCCAAGGATGACTTGCCG    26
                                                          GGCAAGTTGTCCTTGGCTAC      15
             AGCCAAGGATGACTTGCCGG   8
            CAGCCAAGGATGACTTG       2
            CAGCCAAGGATGACTTGCCGGA  2
            CAGCCAAGGATGACTTGC      1
                                                         CGGCAAGTTGTCCTTGGCTA       1
                                                          GGCAAGTTGTCCTTGGCTA       1
                                                           GCAAGTTGTCCTTGGCTAC      1
                                                          GGCAAGTTGTCCTTGGCTACA     1

>vvi-MIR169d [sc:118 - 468088 468207] [chr:11_random - 1216257 1216376]
((((.(((((.(((((((.(..(((((((((..(((((((((((((((.........(((.....)))))))).))))).))))))..)))))))))..).))))))).))))).))))..          (-47.30)
GAGTGGAATGCAGCCAAGAATGATTTGCCGGAATTTATATATAGTGAGAAGAGAAGAGGCTATAGGCTTTCACATATATAGTGAACCCTGGCAAGTTGTCCTTGGCTACATTTGATTCTC
        __CAGCCAAGAATGATTTGCCGG     0
                                                                                           GGCAAGTTGTCCTTGGCTAC         15
                                                                                           GGCAAGTTGTCCTTGGCTA          1
                                                                                            GCAAGTTGTCCTTGGCTAC         1
                                                                                           GGCAAGTTGTCCTTGGCTACA        1

>vvi-MIR169e [sc:9 + 870068 870216] [chr:14 - 14285187 14285335]
((((.((..((((((((((((.(((((.(((((.((((...(((.(((((((....((..(((......))).)).....((((((.....)))))))).))))).))).)..)))))...))))))))))))))))))).)))..)))))))...     (-49.90)
GTCTTGTCTGATAGCCAAGGATGACTTGCCTGCCCTATCCCAAGTGGGTCAATTTCTTTCTTTTTTCCAAGTAGTGTTTGATATTTGATATATCATGAATCCGGTTGTTTGGGTTCCAGGCAGTCACCTTGGCTAATTTTACAGGCTCT
           TAGCCAAGGATGACTTGCCTG    8
           TAGCCAAGGATGACTTGCC      6
          ATAGCCAAGGATGACTTGCCTG    2
           TAGCCAAGGATGACTTGCCT     1

>vvi-MIR169f [sc:84 - 316911 317094] [chr:1_random - 1379004 1379187]
((((.((((....(((((((.(..(((((((.........((((((.(((((.....(((.................)))....))).)).))))))).......(((((........((((((......)))))).)))))..........)))))))..).))))))).)))).)))))....     (-47.59)
GATATTATGGTGCAGCCAAGGATGACTTGCCGAAAATAATTTGCTCGATCGTTAATGTGCTTCTCCAGTTTCTATTGCAATTTATGCATAGGGCAATATATATATCCTTTATAGCTTGTTAATTCAGATATTGACTGAGGAATCAATAATGGGCAAGTTGTGTTTGGCTACATGTTTATCTCAT
            CAGCCAAGGATGACTTGCCGA               317
            CAGCCAAGGATGACTTGCCG                26
             AGCCAAGGATGACTTGCCGA               5
      ATGGTGCAGCCAAGGATGACT                     2
            CAGCCAAGGATGACTTG                   2
            CAGCCAAGGATGACTTGCCGAAA             2
            CAGCCAAGGATGACTTGC                  1

>vvi-MIR169g [sc:34 + 1507120 1507240] [chr:8 + 20118758 20118878]
(((((....(((.(((((((((..((((((((.((.((...((((....((((...((((....))))...))))..)))).)))).)))))))))..))))))))).)))....)))))..         (-52.20)
GAAAGTATGGTGCAGCCAAGGATGACTTGCCGACTCTCTACTTAGCCATCTGCCTTTCTCCAAGGAGGCTTGCAGTACTGATGAAGCCGGCAAGTTGTCTTTGGCTACATGCTTCTTTCTG
```

```
            CAGCCAAGGATGACTTGCCGA   317
            CAGCCAAGGATGACTTGCCG   26
             AGCCAAGGATGACTTGCCGA   5
            CAGCCAAGGATGACTTG       2
      ATGGTGCAGCCAAGGATGACT         2
            CAGCCAAGGATGACTTGC      1

             CGGCAAGTTGTCTTTGGCTAC          197
              GGCAAGTTGTCTTTGGCTAC          91
              GGCAAGTTGTCTTTGGCTACA         49
              GGCAAGTTGTCTTTGGCTA           7
             CGGCAAGTTGTCTTTGGCTACA         7
            CCGGCAAGTTGTCTTTGGCTAC          4
             CGGCAAGTTGTCTTTGGCTA           3
               GCAAGTTGTCTTTGGCTAC          2
             CGGCAAGTTGTCTTTGGCTACAT        2
              GGCAAGTTGTCTTTGGCTACAT        1
              GGCAAGTTGTCTTTGGCT1
             CGGCAAGTTGTCTTTGGC  1

__________________________________
>vvi-MIR169h [sc:118 - 423404 423504] [chr:11_random - 1171573 1171673]
.((((..((..(((.(((((((((((..((((....(((.((((.((((....)))).)))).))).))))..))))))))))).)))..)).))))....      (-34.90)
AGGGTGGAATTGAGCCAAGGATGGCTTGCCGTCCTTTGTCACTATTTGAGGCATTAACTGGTCACGCACGGAGGGTTATCCTTGACTCCTTTAGCTCCTCT
          TGAGCCAAGGATGGCTTGCCG     0

__________________________________
>vvi-MIR169i [sc:118 - 417231 417335] [chr:11_random - 1165400 1165504]
.((((..((..(((.((.((((((((..((((((((...((((((((..........))))))))..))).)))))..)))))))).)).)))..)).))))....  (-40.60)
AGGGTGGAATTGAGCCAAGGATGACTGGCCGTCATTTTTCAGTTGGTATTTGAGGCTTTAACTGGTCATGCACGGCTGGTTATCCCTGTCTCCTTTAGCTCCTCT
           GAGCCAAGGATGACTGGCCGT    0

__________________________________
>vvi-MIR169j [sc:118 - 472655 472776] [chr:11_random - 1220824 1220945]
((((((((((.(.(((((((((..(((((((((....(((.......((((((.(((((.........)))))))).))).)))...)))))))))..))))))))).).)))))))))))..        (-63.90)
GAGAGTGGAGTGCAGCCAAGGATGACTTGCCGGAATTCACATATAGAGTGGAATGAGGCAATAGACCGGCCTCTTCTCATGGTGTCCCTGGCAGGTTGTCCTTGGCTACCTTTCGCTCTCTT
            CAGCCAAGGATGACTTGCCGG               675
            CAGCCAAGGATGACTTGCCG                26
             AGCCAAGGATGACTTGCCGG               8
            CAGCCAAGGATGACTTGCCGGAA             8
            CAGCCAAGGATGACTTG                   2
            CAGCCAAGGATGACTTGCCGGA              2
            CAGCCAAGGATGACTTGC                  1

__________________________________
>vvi-MIR169k [sc:118 - 466033 466154] [chr:11_random - 1214202 1214323]
((((((((((.(.(((((((((..(((((((((..(((((.......)))))....((.((((((....)))))).)).........)))))))))..))))))))).).)))))))))))..        (-61.10)
GAGAGTGGAGTGCAGCCAAGGATGACTTGCCGGAATTCACATATAGAGTGGAATGAGGCAAGAGGCCGGCCTCTTCCCATGGTGTCCCTGGCAGGTTGTCCTTGGCTACCTTTCGTTCTCTT
            CAGCCAAGGATGACTTGCCGG               675
            CAGCCAAGGATGACTTGCCG                26
             AGCCAAGGATGACTTGCCGG               8
            CAGCCAAGGATGACTTGCCGGAA             8
            CAGCCAAGGATGACTTG                   2
            CAGCCAAGGATGACTTGCCGGA              2
            CAGCCAAGGATGACTTGC                  1

__________________________________
>vvi-MIR169l [sc:118 - 389957 390059] [chr:11_random - 1138126 1138228]
.((((.((((.(((((((((((((((.(((((.(..(((.....)))..)..((((.....))))....))))).)))))))))))))))))))).))))....    (-50.10)
AGGGTGGAATTGAGCCAAGGATGACTTGCCGTCCTTTGCATCAAGCATTGAAGCTTTAACTGGGCATGCACGGCTAGTTATTCTTGGCTCATTTGGCCCCTCT
           GAGCCAAGGATGACTTGCCGT    0

__________________________________
>vvi-MIR169m [sc:118 - 214491 214592] [chr:11_random - 962660 962761]
((.((((((..(((((..(((((.((((((((..((....))..(((....((......)).)))....))))))))))))))..)))))..)))))).))..    (-46.20)
AGAGTGGAAGTGAGCCAAGGATGACTTGCCGGCAGCTGCAGCAAGGCATTAAGGTTTAACTGGCCATAACTGGCAAGCATCCGAGGCTCTGTTTCACCCTCT
           GAGCCAAGGATGACTTGCCGG    0
            AGCCAAGGATGACTTGCCGGC   16
            AGCCAAGGATGACTTGCCGG    8

__________________________________
>vvi-MIR169n [sc:118 - 195487 195587] [chr:11_random - 943656 943756]
...((((((((((((..(((((.((((((.((....)).....(((.(((.......))))))......)))))))))))..)))))))))))))).....      (-45.00)
TGAGTGGAATAGAGCCAAGGATGACTTGCCGGCAACTGCAGCAAGGCATAGGGTTTAACTGGTCATAACTGGCAAGCATCTGAGGCTCTATTTCACCCTCT
           GAGCCAAGGATGACTTGCCGG    0
            AGCCAAGGATGACTTGCCGGC   16
            AGCCAAGGATGACTTGCCGG    8

__________________________________
>vvi-MIR169o [sc:118 - 384917 385019] [chr:11_random - 1133086 1133188]
.((((.(...((((((((((((((((((.((((((..(((.((((......))))...))).).)))).).))))))))))))))))))))..).))))....    (-51.70)
AGGGTGGATTTGAGCCAAGGATGACTTGCCGCCATCAGCAGCAAGCATTGAAGCTTTAACTGGGCATGGACAGCGAGTTATTCTTGGCTCATGCGGCCCCTCT
```

```
          GAGCCAAGGATGACTTGCCGC    0

___________________________________
>vvi-MIR169p [sc:118 - 227917 228018] [chr:11_random - 976086 976187]
((.(((((((.(((((..(((((.((((((((..((....))..(((....((......)).)))....)))))))))))))..))))).))))))).))..    (-45.20)
AGTGTGGAATTGAGCCAAGGATGACTTGCCGGCAGCTGCAGCAAGGCATTAAGGTTTAACTGGCTATAACTGGCAAGCATCCGAGGCTCTGTTTCACCCTCT
          GAGCCAAGGATGACTTGCCGG    0
           AGCCAAGGATGACTTGCCGGC   16
           AGCCAAGGATGACTTGCCGG    8

___________________________________
>vvi-MIR169q [sc:118 - 191247 191343] [chr:11_random - 939416 939512]
((((..((((((((((.((((.((.(((((((..((.((((.((((....))).)))).))..))))))).)).)))))))))))))))..)))).. 	(-48.90)
AGGGTGGAATAGAGCCAAGGATGACTTGCCGGCATTTGCAGTAAGTCTATATTAACTGGAACAGCCGGCATGTAATCCTGGCTCTATTTGGTCCTCT
          GAGCCAAGGATGACTTGCCGG    0
           AGCCAAGGATGACTTGCCGGC   16
           AGCCAAGGATGACTTGCCGG    8

___________________________________
>vvi-MIR169r [sc:118 - 160588 160696] [chr:11_random - 908757 908865]
(((((.((((((((((((...(((((((((.(((((((..((.((((((........)))))).)).)))))))..)))))))))..)))))))))))).))))).. 	(-56.10)
AGGGTGGAATTGAGTCAAGGATGACTTGCCGATATATATTTGCAGAAGGCATGCAGGGGCTTTTAGCTATGTGTAACCGGCAAGTTGACTTGACTCAGTTTGGCCCTCT
         TGAGTCAAGGATGACTTGCCG     0

___________________________________
>vvi-MIR169s [sc:118 - 137852 137950] [chr:11_random - 886021 886119]
((.((..((.((.(((((.(((..((((((((((......((.....))......((......))))))))))))..))).))))).)).))..)).)).. 	(-40.50)
GAAAGTGGATTGCAGCCAAGGATGACTTGCCGGCACTTGGCATTAGGCACCATCGATCATCTTCCCGGCAAGTTGTTCCTGGCTACATTCTGCTCTCTT
           CAGCCAAGGATGACTTGCCGG   675
           CAGCCAAGGATGACTTGCCG    26
            AGCCAAGGATGACTTGCCGGC  16
           CAGCCAAGGATGACTTGCCGGC  14
            AGCCAAGGATGACTTGCCGG   8
           CAGCCAAGGATGACTTG       2
           CAGCCAAGGATGACTTGC      1

___________________________________
>vvi-MIR169t [sc:118 - 176151 176260] [chr:11_random - 924320 924429]
(((((.((((.((((((((...(((((((((.(((((((..((.(((((.(((....)))))))).)).)))))))..)))))))))..)))))))).)))).))))).. 	(-53.10)
AGGGTGGAATCGAGTCAAGGATGACTTGCCGATATATATTTGCGGAAGGACTTGCATGGGCCTTTAGCTATGTGTAACCGGCAAGTTGACTTGACTCAGTTTGGCCCTCT
         CGAGTCAAGGATGACTTGCCG     0

___________________________________
>vvi-MIR169T [sc:118 - 166317 166426] [chr:11_random - 914486 914595]
(((((.((((.((((((((...(((((((((..((((((..((.(((((.(.......).))))).)).))))))...)))))))))..)))))))).)))).))))).. 	(-52.80)
AGGGTGGAATTGAGTCAAGGATGACTTGCCGTTATATATTTGCAGAAGGGCACGCAGGGGCCTTTAGCTATGTGTTACCGGCAAGTTGACTTGACTCTGTTTGGCCCTCT
         TGAGTCAAGGATGACTTGCCG     0

___________________________________
>vvi-MIR169v [sc:118 - 109284 109379] [chr:11_random - 857453 857548]
((((((.((((.((((((((((((((((((((((.((((((((.....)))))...))).).)))))))))))).))))))))).))))...)))))) 	(-42.20)
AGGGAGGAACAAAGCCAAGGATGAATTGCCGGCGATGAAAGTAGGCAGCTTTGTCCATGGATCGGCAATTTATTTCTTGGCTATGTTGGGCTCTCT
          AAGCCAAGGATGAATTGCCGG    0

___________________________________
>vvi-MIR169w [sc:21 + 3743273 3743415] [chr:14 + 18908675 18908817]
(((...(..(((.(((.((((((((((((((((....((.(((.(((((((.(((.((((((((((.......).)))))))))))...))).))))))).))..))))))))))))))))).))).))).)..).)))....   	(-57.00)
GGTTATGTGGTGCAGCCAAGGATGACTTGCCGGCAACTCCCTTTATTGTACTCATGCATGCTCATACTTATACTGTTGTGGGCATCATTTAAGTGGCAAAAATGGTGGTCGGCGAGTCATTCTTAGCTACATTTCTGCCTCAT
           CAGCCAAGGATGACTTGCCGG   675
           CAGCCAAGGATGACTTGCCG    26
            AGCCAAGGATGACTTGCCGGC  16
           CAGCCAAGGATGACTTGCCGGC  14
            AGCCAAGGATGACTTGCCGG   8
           CAGCCAAGGATGACTTG       2
           CAGCCAAGGATGACTTGC      1

___________________________________
>vvi-MIR169x [sc:16 + 4993946 4994070] [chr:17 - 351748 351872]
(((..(((...((((((((((((((.(((((((.((((((((((....))).)))..(((........)))..........))))..))))))))))))).))))).))))).....))).))).. 	(-42.70)
GTCTCGTCTGGTAGCCAAGGATGACTTGCCTAAATCCACCAGGTTTCAAAACACTGAATGTAATTATCCACAAAGCGATTCCGTGGTCTTTAGGCAGTCATTCCTTCGGCTAAACTGACCGGCTC
          TAGCCAAGGATGACTTGCC      6
          TAGCCAAGGATGACTTGCCTA    1
```

```
           TAGCCAAGGATGACTTGCCT     1

__________________________________
>vvi-MIR169y [sc:180 - 169908 170155] [chr:1 + 14661042 14661289]
.((..((.(((.((.(((((.((((((((...((((.(.((((((((........(((((((((((...(((.((((....)))).)))................(((.......))).((((........((((((.....)))))).........)))).((((((....))))))....)))).....))))))))))).))).).))))...))))))))))))).)))))
.))..))...  (-73.77)
CGTTGTTTGGTAGCGAAGGATGACTTGCCTACAGCCTCCTTAAGGTTTCAAAATACCGAGCTTGTCTACCTTTGATCAAATGATTCAAGAAACAAAAGAAAAGAAAGGCCATGGCAGCCATCCATCAGAGTCAACATGAGAATCATGTTCTATAGAAATTGGAGTCATTCTTGAGAATGAACACGACACTATTAGCTTGGAATCTATAATGTGGTTTATGGCAAGTCTCCTTGGCACC
TGATGGCCTC
          TAGCGAAGGATGACTTGCCTA     0


__________________________________
>vvi-MIR171a [sc:9 + 461317 461415] [chr:14 - 14693988 14694086]
..(((...((((((((((((.((((((((((((....((((..((((.....))))..))))....)))))))))))).)))))))))))))...)))         (-47.80)
TGGTGGGAAGCGTGATGTTGGGACGGCTCAATCAAACCAAAGATCTCAATGGTTGAGTCCTTTAATCTGATTGAGCCGTGCCAATATCATGTTCAATAC
                                                                      TTGAGCCGTGCCAATATCATG    0
                                                                   TGATTGAGCCGTGCCAATATC       637
                                                                   TGATTGAGCCGTGCCAATA         43
                                                                   TGATTGAGCCGTGCCAATAT        4
                                                                   TGATTGAGCCGTGCCAAT          4
                                                                   TGATTGAGCCGTGCCAA           1
                                                                  CTGATTGAGCCGTGCCAATATC       1


__________________________________
>vvi-MIR171b [sc:36 - 591211 591309] [chr:12 + 7277319 7277417]
(..((((((((((((((((.(((((((.((..((((((((((((......)).))))))))))..)).))))))).)))))))))))))))))..)..         (-46.90)
TGGGGGGAGGTATTGGCGTGCCTCAATTTAAAGACATGGTTAGATGAAGAGATATTAGCCATGTGATTTGATTGAGCCGCGTCAATATCTCCTTTTGCA
                                                                    TGATTGAGCCGCGTCAATATC      0


__________________________________
>vvi-MIR171c [sc:36 - 536559 536661] [chr:12 + 7331967 7332069]
.((((.((((.((((((((..(((((((((.(((((.(((.(((((.((....)))))))).))).))))).)))))))))..)))))))).))))....))))    (-48.00)
TGGTATTACGGGATATTGGTGCGGTTCAATAAGAAAGCAGTGCTCGCAATCTTTTTGAGTTCTGGTTTTTGATTGAGCCGTGCCAATATCACGTGTCATTGCT
                                                                      TGATTGAGCCGTGCCAATATC     637
                                                                      TGATTGAGCCGTGCCAATA       43
                                                                      TGATTGAGCCGTGCCAAT        4
                                                                      TGATTGAGCCGTGCCAATAT      4
                                                                         TTGAGCCGTGCCAATATCACG  4
                                                                         TTGAGCCGTGCCAATATCAC   2
                                                                      TGATTGAGCCGTGCCAA         1


__________________________________
>vvi-MIR171d [sc:795 - 2155 2254] [scaffold not incorporated in genome assembly]
.......(((.(((((((.((((((((((((((((.(((.(.(((((......))))).).))).))))))))))))))).))))))).)))..........       (-38.60)
TAGATACACGAGATATTGATACGGTTCAATTAGAAAGCTGTGTTCTAAGTTAAGAACTCTGTTGTTTGATTGAGCCGTGCCAATATCACGTCCTACTTCT
                                                                   TGATTGAGCCGTGCCAATATC       637
                                                                   TGATTGAGCCGTGCCAATA         43
                                                                   TGATTGAGCCGTGCCAAT          4
                                                                   TGATTGAGCCGTGCCAATAT        4
                                                                      TTGAGCCGTGCCAATATCACG    4
                                                                      TTGAGCCGTGCCAATATCAC     2
                                                                   TGATTGAGCCGTGCCAA           1


__________________________________
>vvi-MIR171e [sc:13 + 5162582 5162684] [chr:11 + 5162582 5162684]
((((((....(((((((((.((((((((.((.((((..((((((......))))))...)).)))).))))))))).))))))))))))))))......    (-44.70)
GGAAAGTAAGCGATGTTGGTGAGGTTCAATCCGAAGACGGATTTACGAAGAGCCGTAAAGAACGATCTCTGATTGAGCCGCGCCAATATCACTTTCTGCTAAG
                                                                      TGATTGAGCCGCGCCAATATC     53
                                                                      TGATTGAGCCGCGCCAATA       4
                                                                      TGATTGAGCCGCGCCAA         1


__________________________________
>vvi-MIR171f [sc:104 - 699397 699499] [chr:9 + 7000493 7000595]
.....(((((.((((((((((.((((((((((((.((((..(((.(((......))).)))..)))))))))))))))).)))))))))).))))).......    (-45.90)
TTGAAGAAAGCGATGTTGGTGAGGTTCAATCTGAAGATTGATTTATGCTTGAATGTAAAAACTGATCTCAGATTGAGCCGCGCCAATATCACTTTTCTGTGGC
                                                                         TTGAGCCGCGCCAATATCACT  0


__________________________________
>vvi-MIR171g [sc:1 - 10508015 10508090] [chr:18 + 3212869 3212944]
....(.(((...((((((((((..((((.((((........))))))))...))))))))))).....))).)....       (-21.70)
GCCAGCCTCTCCATGTTGGTTCCATCGGTGGGGGACACCAACTCCTTGAGCCGAACCAATATCACCCGAGCCAAAT
                                                 TTGAGCCGAACCAATATCACC     0


__________________________________
>vvi-MIR171h [sc:16 + 3531871 3531966] [chr:17 - 1813852 1813947]
....(((.((((((((((((((((((((.(((..(((.(((.(((....))).))).)))..))).)))))))))))))))))))).))).......           (-47.30)
TTTCACGAGATGTTGGTGCGGTTCAACCTAACTGTAGTGTCCATGAATGTATCGACTCTGATGTTTGGTTGAGCCGCGCCAATATCCCGTCTATCT
                                                                TGGTTGAGCCGCGCCAATATC         0
```

```
>vvi-MIR171i [sc:16 - 4463645 4463747] [chr:17 + 882071 882173]
...(((.(((..((((((((.((((((((((((.((.(((...((((((...))))))...))).)).)))))))))))).))))))))..))).)))).....   (-44.90)
GAAGAAGGTGGGGATGTTGGAATGGCTCAATCAAATCAAATCTCCCAAGGGTTTTGGGTCCTTTAATCTGATTGAGCCGTGCCAATATCATCATGTTCACTTC
                                                                          TTGAGCCGTGCCAATATCATC   0
                                                                       TGATTGAGCCGTGCCAATATC      637
                                                                       TGATTGAGCCGTGCCAATA        43
                                                                       TGATTGAGCCGTGCCAAT         4
                                                                       TGATTGAGCCGTGCCAATAT       4
                                                                       TGATTGAGCCGTGCCAA          1
                                                                      CTGATTGAGCCGTGCCAATATC      1
```

```
>vvi-MIR172a [sc:25 + 2163229 2163338] [chr: 6: - 21821956 21822065]
....((((.(((((((((((((((((((((.(((((....))..((..(.(((...))).)..))............))).))))))))))))))))))))).))))))..        (-46.50)
TTGTTTGCGCATGTAGCATCATCAAGATTCACATGCAAATGCACGGTGGTGATGTTATCTCTGCCAAACTTTTCAAGGATGAGAATCTTGATGATGCTGCATCGGCAATA
                                                                                  AGAATCTTGATGATGCTGCAT                73450
                                                                                  AGAATCTTGATGATGCTGCA                 839
                                                                                 GAGAATCTTGATGATGCTGCAT                34
                                                                                   GAATCTTGATGATGCTGCAT                33
                                                                                  AGAATCTTGATGATGCTGC                  32
                                                                                 GAGAATCTTGATGATGCTGCA                 23
                                                                                    AATCTTGATGATGCTGCAT                9
                                                                                      TCTTGATGATGCTGCAT                4
                                                                                  AGAATCTTGATGATGCT                    4
                                                                                     ATCTTGATGATGCTGCAT                3
                                                                                  AGAATCTTGATGATGCTGCATC               2
                                                                                  AGAATCTTGATGATGCTG                   2
                                                                                  AGAATCTTGATGATGCTGCATCG              1
                                                                                TGAGAATCTTGATGATGCTGCAT                1
```

Wrong strand prediction:

TGAATCTTGATGATGCTACAT   0

```
>vvi-MIR172b [sc:17 + 1425044 1425161] [chr: 13 - 6323135 6323252]
....((((.((((((((((((((((((((((.((..(....)(((((.((((((((((........))))))))....)).))))))..)).))))))))))))))))))))))).)))))..            (-62.20)
TTGTTTGCAGGTGTAGCATCATCAAGATTCACATGCGAAAGCCCGGCGCGGCAGCGATGCCATCTTCGCTGCCTCAAGTTTTGGGGTTGAGAATCTTGATGATGCTGCATCGGCAATA
                                                                                           AGAATCTTGATGATGCTGCAT                    73450
                                                                                           AGAATCTTGATGATGCTGCA                     839
                                                                                            GAGAATCTTGATGATGCTGCAT                  34
                                                                                              GAATCTTGATGATGCTGCAT                  33
                                                                                             AGAATCTTGATGATGCTGC                    32
                                                                                              GAGAATCTTGATGATGCTGCA                 23
                                                                                                  AATCTTGATGATGCTGCAT               9
                                                                                                    TCTTGATGATGCTGCAT               4
                                                                                               AGAATCTTGATGATGCT                    4
                                                                                                   ATCTTGATGATGCTGCAT               3
                                                                                            AGAATCTTGATGATGCTGCATC                  2
                                                                                            AGAATCTTGATGATGCTG                      2
                                                                                            AGAATCTTGATGATGCTGCATCG                 1
                                                                                          TGAGAATCTTGATGATGCTGCAT                   1
```

Wrong strand prediction:

TGAATCTTGATGATGCTACAC   0

```
>vvi-MIR172c [sc:17 - 4437525 4437662] [chr:13 - 3310634 3310771]
..((((.(.((.(((((((((((((((((.((.((...((((..((...((..(((..((.((.((((.....)))))).))...)))..)).))..)))).)).)).))))))))))))))))).)).).)))).....   (-57.90)
GTTTGCGGATGGAGCATCATCAAGATTCACAAGTATTGAGACTCAGTGCGTGGTGGTGATGGTGACTTTTGTGGTCCCTTCCTACACTCCGATGGCTCTTTGATGTGGGAATCTTGATGATGCTGCAGCGGCAATAAA
                                                                                                                GGAATCTTGATGATGCTGCA   1
                                                                                                                GGAATCTTGATGATGCTGCAG  1
```

```
>vvi-MIR172d [sc:29 - 1692538 1692646] [chr:8 - 11559580 11559688]
....((.((((((((((((((((((((((.((.((((......(((((.(((...))).)))))).......))).).)).))))))))))))))))))))))).)).......         (-52.22)
GTTAGCTGATGCAGCATCATCAAGATTCACACCCAATGGAAGGGCAGTGATGCAATCTCTGCCAAAGATTTTGAGATGAGAATCTTGATGATGCTGCATTCGCAGTGAA
                                                                               AGAATCTTGATGATGCTGCAT        73450
                                                                               AGAATCTTGATGATGCTGCA         839
                                                                               AGAATCTTGATGATGCTGCATT       80
                                                                                GAATCTTGATGATGCTGCAT        66
                                                                              GAGAATCTTGATGATGCTGCAT        34
                                                                               AGAATCTTGATGATGCTGC          32
                                                                              GAGAATCTTGATGATGCTGCA         23
                                                                                 AATCTTGATGATGCTGCAT        18
                                                                                   TCTTGATGATGCTGCAT        8
                                                                                  ATCTTGATGATGCTGCAT        6
                                                                               AGAATCTTGATGATGCT            4
                                                                               AGAATCTTGATGATGCTG           2
                                                                             TGAGAATCTTGATGATGCTGCAT        1
                                                                               AGAATCTTGATGATGCTGCATTCG     1
```

```
>vvi-MIR319b [sc:5 + 4170832 4171025] [chr:1 + 4170832 4171025]
.((((((...(((((((((((((((((..((.(((((((....((((((.(((..(((.(((..((((((..(((((((..((((.(((.............)))))))..)))))))..))))))..))).)))..)))))))))..))))))).))..)))))))))))))))))))......)))))).       (-102.82)
TGTGGAAGAAGGAGCTCTCTTTAGTCCAGTCCGAGACAGCTTCAAGGCTGATACGTGGCTGCTGACTCGTTGCTTCAAGAACTCATCACATAGGCCTGGAAAAGATGGATGGTTTTTTGATCCAACGATGCAGGAGCTGCGTTCAGTTATGGCTGTCACGTCTTGGACTGAAGGGAGCTCCCTTGTATTCCACA
                                                                                                                                                                                    CTTGGACTGAAGGGAGCTCCC       0
                                                                                                                                                                                     TTGGACTGAAGGGAGCTCCCT      1

>vvi-MIR319c [sc:132 + 439049 439230] [chr:2 - 661750 661931]
...((.(((((.(((((..((((((((((..((((((..((..(((((((.((((.(((.(((..(((((((..((..(((((.((........)).)))))..))..))))))))..))).)).).)))).))))))).))..))))))..))))))))))..))))).))))).)).....       (-83.40)
TTACATTGAAGAGAGCTTTCTTCAGTCCACTCATGGGTGGCAGTAGGATTGAATTAGCTGCCGACTCATTCATCCAAATACTGTGTTAAGGACTACCCAGTAAATGATTGAATGATGCGGGAGACAAATTGGATCTTAAGCTCCCTGTGCTTGGACTGAAGGGAGCTCCCTTCACTGCAATC
                                                                                                                                                                CTTGGACTGAAGGGAGCTCCC       0
                                                  AGCTGCCGACTCATTCATCCA       3
                                                                                                                                                                 TTGGACTGAAGGGAGCTCCCT      1

>vvi-MIR319e [sc:13 + 4274222 4274328] [chr:11 + 4274222 4274328]
.(.((((.((((((((((((.(((.((((((((.......((((((...........))))).))...))))))))).)))))))))))))))).)))).)...            (-40.40)
TGCAGAAATGGGGGTTCCTTTGCAGCCCAAAACAACTCCATCGCTGAAGAAGATGATGAACTTCATGCTCCTTGTTTTGGACTGAAGGGAGCTCCTAGTTCTTCTCT
                                                                            TTTGGACTGAAGGGAGCTCCT           0
                                                                            TTTGGACTGAAGGGAGCT              1

>vvi-MIR319f [sc:3 - 409791 409983] [chr:6 + 9069150 9069342]
(((((...(((.(((((((((((((((((..((((((..((..((((((..((.(((.((.(((..((((((((.((..((((((((....(((......))).))))))))..)).))))))))..))).)).))).)).))..))))))..))..))))))..))))))))))))))))).)))))))).....        (-88.70)
AGTGGTTTAAGAGAGCTTCCTTCAGTCCACTCATGGATGGGTTAGGGGTTTGGATTAGCTGCCGACTCATTCATTCAAACACAGTAGAATGGAAATGGAGTTTACTACTGTGAATGTGTGAATGATGCGGAGGTAAATTTCATCCTTTTCTTGTCTGTGCTTGGACTGAAGGGAGCTCCCTTCTACTGTTTT
                                                                                                                                                                                   CTTGGACTGAAGGGAGCTCCC       0
                                                                                                                                                                                    TTGGACTGAAGGGAGCTCCCT      1

>vvi-MIR319g [sc:16 + 1677842 1678063] [chr:17 - 3667755 3667976]
.....(((...((((((((((((((((((..((.((((.(((.(((((..((.(((.((((.(((((((...(((((.(((((.((.((((.((..(((((((...))))).))..)).)))).))......))))).))))).)...)))))))).)))).))).)).)...)))).).))).)))).)).))))))))))))))))))....))).....  (-96.00)
GAACAGCTGAAGAGCTCCTTTAGTCCAATAAAGAGGGCTGAGGGGCGGCCAGAGCTGCCATCTCATGCACTTGGCTATGCTTATTCTTTTCTCAATCAATTCGGTTCGAGTATGGCTGTGGAGTGACAAATGCTAAGTTTAGCCTGATGCATGACATGGGAGCAACTCCTCCGCACGCTTTGCCCACTCATTGGACTGAAGGGAGCTCCCAGGGCAGATCT
                                                                                                                                                                                                                   ATTGGACTGAAGGGAGCTCCC  0

>vvi-MIR390 [sc:3 - 1385305 1385444] [chr:6 + 8093689 8093828]
((((((((.((((((((((.(((((((((((((.((.(((((((...(((....((((((((((....)))).))))))....)))..)))))))...))...))))))))))))).)))))))))).)))))))))..  (-62.30)
AGAATCTGTTAAGCTCAGGAGGGATAGCGCCATGAGCCATGATGAAAATGATTGTTGAGTTGAAAATTTTTTCTAGCTTGTTTTTTGGGTCATCATTTTGTGTGTGTGGCGCTATCTATCCTGAGTTTCACGGGTTCTTC
          AAGCTCAGGAGGGATAGCGCC    141
          AAGCTCAGGAGGGATAGCGCCA   5
          AAGCTCAGGAGGGATAGCGC     4
           AGCTCAGGAGGGATAGCGC     1
           AGCTCAGGAGGGATAGCGCC    1
              TCAGGAGGGATAGCGCC    1

>vvi-MIR393a [sc:222 - 104163 104288] [chr:Un_random - 54807498 54807623]
((((((.((((((.(((((((.((((.(((((.((((..(...............(((((((..........)))))))).....)..)))).))))))))).))))))).)))))).))))))..    (-53.90)
GGTGGACAGTTCCAAAGGGATCGCATTGATCCCATGGTTATCTTCCACCTCTCTTACCAGACTATCATAACAAAGTCTGGCCTTTTTCCCATTGGATCATGCTATCCCTTAGGAACTCTCCATCAG
          TCCAAAGGGATCGCATTGATCC    0
         TTCCAAAGGGATCGCATTGAT      14983
          TCCAAAGGGATCGCATTGATC     398
         TTCCAAAGGGATCGCATTGA       136
         TTCCAAAGGGATCGCATTGATC     127
            CAAAGGGATCGCATTGAT      46
          TCCAAAGGGATCGCATTGAT      30
          TCCAAAGGGATCGCATTGA       19
            CAAAGGGATCGCATTGATC     7
         TTCCAAAGGGATCGCATT         5
         TTCCAAAGGGATCGCAT          4
         TTCCAAAGGGATCGCATTG        4
             AAAGGGATCGCATTGAT      2
          TCCAAAGGGATCGCATTG        1
            CAAAGGGATCGCATTGA       1

>vvi-MIR393b [sc:17 - 3375224 3375306] [chr:13 + 4372990 4373072]
((((((((.((((((((((((.((((.(((((.(((..(.....)..))))))))))))).))))))))))))).))))))))..            (-45.70)
GGAGGAGGCATCCAAAGGGATCGCATTGATCCCAAACTCTCCATGTCTTTGGATCATGCTATCCCTTTGGATTCCTCCTTTGG
          TCCAAAGGGATCGCATTGATCC    0
          TCCAAAGGGATCGCATTGATC     398
            CAAAGGGATCGCATTGAT      46
          TCCAAAGGGATCGCATTGAT      30
          TCCAAAGGGATCGCATTGA       19
            CAAAGGGATCGCATTGATC     7
         ATCCAAAGGGATCGCATTGAT      4
             AAAGGGATCGCATTGAT      2
          TCCAAAGGGATCGCATTG        1
            CAAAGGGATCGCATTGA       1

>vvi-MIR394a [sc:38 + 1925563 1925651] [chr:12 - 14925943 14926031]
```

```
((((((((((((((((.(((.(((((((((........................))))))))).))).)))))))))))))))))..      (-51.01)
CAGAGCCATTTTGGCATTCTGTCCACCTCCATGCACATCATGGGTCTATATTCTTTTGGAGGTGGCCAGCATGCCAAAGTGGCTCTGTA
          TTGGCATTCTGTCCACCTCC      748
          TTGGCATTCTGTCCACCTCCA     9
            GGCATTCTGTCCACCTCC      3
          TTGGCATTCTGTCCACCTC       3
          TTGGCATTCTGTCCACCT        2
          TTGGCATTCTGTCCACC         1

__________________________________
>vvi-MIR394b [sc:1 + 12335062 12335154] [chr:18 - 1385805 1385897]
((((((((.((((((.(.((((((((((((......(((((......)))))....)).))))))))))).).))))))..))))))))).  (-42.60)
ACAGAGTTTATTGGCATTCTGTCCACCTCCCATCTCTTGAAAATCTCTCTTTTCTCTCTGTGGAGGTGGGCATACTGCCAACCAAGCTCTGTT
          TTGGCATTCTGTCCACCTCC      748
            GGCATTCTGTCCACCTCC      3
          TTGGCATTCTGTCCACCTC       3
          TTGGCATTCTGTCCACCT        2
          TTGGCATTCTGTCCACC         1
         ATTGGCATTCTGTCCACCTCC      1

__________________________________
>vvi-MIR394c [sc:1 + 10217177 10217278] [chr:18 - 3503681 3503782]
((((((((.((((((((((((.((.((((((......(((((((....)))))))(((......)))..)))))).)).))))))))))))).))))))))..    (-59.50)
CAGAGCCATTTTGGCATTCTGTCCACCTCCATATATACCAATTCTTCTGAATTGGCGCCCACAAGCGTTTGGAGGCGGCCAGGATGCCAAATTGGCTCTGTA
          TTGGCATTCTGTCCACCTCC      748
          TTGGCATTCTGTCCACCTCCA     9
            GGCATTCTGTCCACCTCC      3
          TTGGCATTCTGTCCACCTC       3
          TTGGCATTCTGTCCACCT        2
          TTGGCATTCTGTCCACC         1

__________________________________
>vvi-MIR395a [sc:5 + 6669509 6669600] [chr:1 + 6669509 6669600]
....((..(((((((((..(.(((((((.((((((((...............).))))))).))))))).)..)))))))))..))......  (-40.86)
GTCCCCTAGAGTTCCCTTGATCACTTCACTAGGGAGCTTCGCTAGTTTTAATGACTTCCTACTGAAGTGTTTGGGGGAACTCCTGGTACCGA
                                                              TGAAGTGTTTGGGGGAACTC 198
                                                              TGAAGTGTTTGGGGGAAC   3
                                                             CTGAAGTGTTTGGGGGAACTC 2
                                                                AAGTGTTTGGGGGAACTC 1
                                                               GAAGTGTTTGGGGGAACTC 1
                                                              TGAAGTGTTTGGGGGAACT  1

__________________________________
>vvi-MIR395b [sc:5 + 6643441 6643530] [chr:1 + 6643441 6643530]
....((..((((((((((...(((((((...(((...(((......)))..)))......)))))))...))))))))))..))......    (-34.90)
GTCCCCTAGAGTTCCCCTTACCACTTCACTGGGGATCTTCTCTAATGAATGCCTACGTACTGAAGTGTTTGGGGGAACTCCTGGTGCCAT
                                                            TGAAGTGTTTGGGGGAACTC   198
                                                            TGAAGTGTTTGGGGGAAC     3
                                                           CTGAAGTGTTTGGGGGAACTC   2
                                                              AAGTGTTTGGGGGAACTC   1
                                                             GAAGTGTTTGGGGGAACTC   1
                                                            TGAAGTGTTTGGGGGAACT    1

__________________________________
>vvi-MIR395c [sc:5 - 6640691 6640782] [chr:1 - 6640691 6640782]
....((..(((((((((..(.(((((((.((((((...((..((....))..)).)))))).))))))).)..)))))))))..))......  (-37.80)
GTCCCCTAGAGTTCCCTTGACCACTTCACTGGGGACCTTCTCTAGTTATAATGACTTCCTACTGAAGTGTTTGGGGGAACTCCTGGTGCCAT
                                                              TGAAGTGTTTGGGGGAACTC 198
                                                              TGAAGTGTTTGGGGGAAC   3
                                                             CTGAAGTGTTTGGGGGAACTC 2
                                                              TGAAGTGTTTGGGGGAACT  1
                                                               GAAGTGTTTGGGGGAACTC 1
                                                                AAGTGTTTGGGGGAACTC 1

__________________________________
>vvi-MIR395d [sc:5 + 6653540 6653625] [chr:1 + 6653540 6653625]
....((..(((((((((..(.(((((((.((((((((.........)).)))))).))))))).)..)))))))))..))......        (-40.80)
GCCCCCTAGAGTTCCCCTGACCACTTCATTGGGGATCTTCTCTAATGACTTCCTACTGAAGTGTTTGGGGGAACTCCTGGTGTCAT
                                                        TGAAGTGTTTGGGGGAACTC      198
                                                        TGAAGTGTTTGGGGGAAC        3
                                                       CTGAAGTGTTTGGGGGAACTC      2
                                                          AAGTGTTTGGGGGAACTC      1
                                                         GAAGTGTTTGGGGGAACTC      1
                                                        TGAAGTGTTTGGGGGAACT       1

__________________________________
>vvi-MIR395e [sc:5 + 6646025 6646116] [chr:1 + 6646025 6646116]
....((..(((((((((..(.(((((((.((((((...((............)).)))))).))))))).)..)))))))))..))......  (-37.30)
CTCCCCTAGAGTTCCCTTGACCACTTCACTGGGGACCTTCTCTAATTATAATGACTTCCTACTGAAGTGTTTGGGGGAACTCCTGGTGCCAT
                                                              TGAAGTGTTTGGGGGAACTC 198
                                                              TGAAGTGTTTGGGGGAAC   3
                                                             CTGAAGTGTTTGGGGGAACTC 2
```

```
                                                    AAGTGTTTGGGGGAACTC 1
                                                   GAAGTGTTTGGGGGAACTC 1
                                                  TGAAGTGTTTGGGGGAACT  1

__________________________________
>vvi-MIR395f [sc:5 + 6630319 6630404] [chr:1 + 6630319 6630404]
(.((((..(((((((((..(.(((((((.((((((((.........)).)))))).))))))).)..)))))))))..)).)))..        (-44.20)
GTACCCTAGAGTTCCCCTGACCACTTCACTGGGGATCTTCTCTAATGACTTCCCACTGAAGTGTTTGGGGGAACTCCTGGTGTCAT
                                                        TGAAGTGTTTGGGGGAACTC      198
                                                        TGAAGTGTTTGGGGGAAC        3
                                                       CTGAAGTGTTTGGGGGAACTC      2
                                                          AAGTGTTTGGGGGAACTC      1
                                                         GAAGTGTTTGGGGGAACTC      1
                                                        TGAAGTGTTTGGGGGAACT       1

__________________________________
>vvi-MIR395g [sc:5 + 6622890 6622975] [chr:1 + 6622890 6622975]
(.(.((..(((((((((..(((((((((.(((((((...........))))))).)))))))))..)))))))))..)).).)..        (-44.40)
GTCCCCTAGAGTTCCCCTGAGCACTTCATTGGGGATCCTTAGTTTTCAATTCCTACTGAAGTGTTTGGGGGAACTCCCGGTGTCAT
                                                        TGAAGTGTTTGGGGGAACTC      198
                                                        TGAAGTGTTTGGGGGAAC        3
                                                       CTGAAGTGTTTGGGGGAACTC      2
                                                          AAGTGTTTGGGGGAACTC      1
                                                         GAAGTGTTTGGGGGAACTC      1
                                                        TGAAGTGTTTGGGGGAACT       1

__________________________________
>vvi-MIR395h [sc:5 + 6706656 6706747] [chr:1 + 6706656 6706747]
(..((...(((((((((..(.(((((((.((((((...((...........)).)))))).))))))).)..)))))))))..))..)...  (-37.50)
GTCCCCTAGAGTTCCCTTGACCACTTCACTGGGGACCTTCTCTAATTATAATGACTTCCTACTGAAGTGTTTGGGGGAACTCCTGGTGCTAT
                                                              TGAAGTGTTTGGGGGAACTC 198
                                                              TGAAGTGTTTGGGGGAAC   3
                                                             CTGAAGTGTTTGGGGGAACTC 2
                                                                AAGTGTTTGGGGGAACTC 1
                                                               GAAGTGTTTGGGGGAACTC 1
                                                              TGAAGTGTTTGGGGGAACT  1

__________________________________
>vvi-MIR395i [sc:5 + 6702646 6702731] [chr:1 + 6702646 6702731]
....((..(((((((((..(.(((((((.((((((((.........)).)))))).))))))).)..)))))))))..))......        (-40.80)
GCCCCCTAGAGTTCCCCTGACCACTTCACTGGGGATCTTCTGTAATGACTTCCTACTGAAGTGTTTGGGGGAACTCCTGGTGTCAT
                                                        TGAAGTGTTTGGGGGAACTC      198
                                                        TGAAGTGTTTGGGGGAAC        3
                                                       CTGAAGTGTTTGGGGGAACTC      2
                                                          AAGTGTTTGGGGGAACTC      1
                                                         GAAGTGTTTGGGGGAACTC      1
                                                        TGAAGTGTTTGGGGGAACT       1

__________________________________
>vvi-MIR395j [sc:5 + 6693030 6693115] [chr:1 + 6693030 6693115]
....((..(((((((((..(.(((((((.((((((((.........)).)))))).))))))).)..)))))))))..))......        (-40.80)
GCCCCCTAGAGTTCCCCTGACCACTTCACTGGGGATCTTCTTTAATGACTTCCTACTGAAGTGTTTGGGGGAACTCCTGGTGTCAT
                                                        TGAAGTGTTTGGGGGAACTC      198
                                                        TGAAGTGTTTGGGGGAAC        3
                                                       CTGAAGTGTTTGGGGGAACTC      2
                                                          AAGTGTTTGGGGGAACTC      1
                                                         GAAGTGTTTGGGGGAACTC      1
                                                        TGAAGTGTTTGGGGGAACT       1

__________________________________
>vvi-MIR395k [sc:5 + 6678361 6678452] [chr:1 + 6678361 6678452]
...((..(((((((((..(.(((((((.((((((...((...........)).)))))).))))))).)..)))))))))..)).......  (-37.30)
TCCCCTAGAGTTCCCTTGACCACTTCACTGGGGACCTTCTCTAATTATAATGACTTCCTACTGAAGTGTTTGGGGGAACTCCTGGTGCCATT
                                                             TGAAGTGTTTGGGGGAACTC  198
                                                             TGAAGTGTTTGGGGGAAC    3
                                                            CTGAAGTGTTTGGGGGAACTC  2
                                                               AAGTGTTTGGGGGAACTC  1
                                                              GAAGTGTTTGGGGGAACTC  1
                                                             TGAAGTGTTTGGGGGAACT   1

__________________________________
>vvi-MIR395l [sc:5 + 6699102 6699188] [chr:1 + 6699102 6699188]
....((..(((((((((..(.(((((((.(((((..((.........))..))))).))))))).)..)))))))))..))......       (-41.10)
GCCCCCTAGAGTTCCCCTGACCACTTCACTGGGGGATCTTCTGTAATGACTTCCTACTGAAGTGTTTGGGGGAACTCCTGGTGTCAT
                                                         TGAAGTGTTTGGGGGAACTC     198
                                                         TGAAGTGTTTGGGGGAAC       3
                                                        CTGAAGTGTTTGGGGGAACTC     2
```

```
                                                                        AAGTGTTTGGGGGAACTC      1
                                                                       GAAGTGTTTGGGGGAACTC      1
                                                                      TGAAGTGTTTGGGGGAACT       1

__________________________________
>vvi-MIR395m [sc:5 + 6697815 6697906] [chr:1 + 6697815 6697906]
....((..((((((((..((((((((.((((((...((..((....))..)).)))))).)))))))))..)))))))))..))......   (-42.00)
GTCCCCTAGAGTTCCCTTGAACACTTCACTGGGGACCTTCTCTAGTTATAATGACTTCCTACTGAAGTGTTTGGGGGAACTCCTGGTGCCAT
                                                              TGAAGTGTTTGGGGGAACTC 198
                                                              TGAAGTGTTTGGGGGAAC   3
                                                             CTGAAGTGTTTGGGGGAACTC 2
                                                                AAGTGTTTGGGGGAACTC 1
                                                               GAAGTGTTTGGGGGAACTC 1
                                                              TGAAGTGTTTGGGGGAACT  1

__________________________________
>vvi-MIR395n [sc:101 - 1067989 1068125] [chr:17 - 6465770 6465906]
(((.(..(((((((.(((..(.((.(((((......................(((((.....)))))....................((((.....)))).))))).)).)..))).))))))))..).)))..     (-37.00)
GGCCTTGAGAGTTCCCCCAACCCCTCCAGTATGACCATCTCTCTTCTTCTTTTAATTTGTTCATCAAATCTTCTTCTACTATCTATTTCCAGGGTGATTTCCTCTACTGAAGAGTCTGGAGGAACTCTTGGTGCCAC
                                                                                                           CTGAAGAGTCTGGAGGAACTC    0

__________________________________
>vvi-MIR396a [sc:104 + 339535 339650] [chr:9 - 7360342 7360457]
...(((((((..(((((((((((((((((.((.((((((((.((.(((.....((((......)))).))))))).)))).)).)).)))))))))))))))))..))))))).  (-51.30)
TCCTGTCATGCTTTTCCACAGCTTTCTTGAACTACTTTTCCATCTACCTGTTTGATGATCTTCTTCATAAGGAGTGTGAAATAGAAGCTCAAGAAAGCTGTGGGAGGACATGGCAA
             TTCCACAGCTTTCTTGAACTA  0
             TTCCACAGCTTTCTTGAAC    810
             TTCCACAGCTTTCTTGAA     132
             TTCCACAGCTTTCTTGAACT   44
             TTCCACAGCTTTCTTGA      18
                                                                                         CTCAAGAAAGCTGTGGGAGG           3
              TCCACAGCTTTCTTGAAC    1

__________________________________
>vvi-MIR396b [sc:13 + 5206054 5206161] [chr:11 + 5206054 5206161]
....((((((.(((((((((((((((((((((((...........((((..................))))))))))))))))))))))))))))).))))))..           (-52.67)
TCCTGCCATGCTTTTCCACAGCTTTCTTGAACTTCTTCTCCCTGTTTGGTGGTCTCCTACGTTTACCCATCCACTGAAGTTCAAGAAAGCTGTGGGAAACCATGGCTT
             TTCCACAGCTTTCTTGAACTT  2204
             TTCCACAGCTTTCTTGAAC    810
             TTCCACAGCTTTCTTGAA     132
                CACAGCTTTCTTGAACTT  44
             TTCCACAGCTTTCTTGAACT   44
             TTCCACAGCTTTCTTGA      18
              TCCACAGCTTTCTTGAAC    1
             TTCCACAGCTTTCTTGAACTTC 1

__________________________________
>vvi-MIR396c [sc:6 - 5105253 5105360] [chr:4 - 5114321 5114428]
(((.(((((.((((((((((((((((.((((((((((..(((.(((...........))))))..)))))..))))).)))))))))))))))).))))).)))..           (-48.00)
TTCTGTGATCTTCCACAGCTTTCTTGAACTGTTTTGGAAGCCTTCCACTTCCATGCAATGGAGGAGTTCAACCAACAGCTCAGGAAAGTTGTGGAACATTACTGAGAG
          TTCCACAGCTTTCTTGAAC       810
          TTCCACAGCTTTCTTGAA        132
          TTCCACAGCTTTCTTGAACTG     83
          TTCCACAGCTTTCTTGAACT      44
          TTCCACAGCTTTCTTGA         18
           TCCACAGCTTTCTTGAACTG     12
           TCCACAGCTTTCTTGAAC       1
             CACAGCTTTCTTGAACTG     1

__________________________________
>vvi-MIR396d [sc:13 - 5212374 5212493] [chr:11 - 5212374 5212493]
....((((..(((((((((((.((((((((((.....((((((...(((((((......)))))))...))))..)).......))))))))).))))))))))))..))))......          (-47.80)
CTTTGTATTCTTCCACAGCTTTCTTGAACTGCATTTTTCAGACTACTGGTGTGTTTTTGTATGCACTCAAAGTCATTGTAACAGTTGCGGTTCAATAAAGCTGTGGGAAGATACCAACAG
          TTCCACAGCTTTCTTGAAC       810
          TTCCACAGCTTTCTTGAA        132
          TTCCACAGCTTTCTTGAACTG     83
          TTCCACAGCTTTCTTGAACT      44
          TTCCACAGCTTTCTTGA         18
           TCCACAGCTTTCTTGAACTG     12
           TCCACAGCTTTCTTGAAC       1
             CACAGCTTTCTTGAACTG     1
            CCACAGCTTTCTTGAACTGCA   1
                                                                                            TCAATAAAGCTGTGGGAAG        1

__________________________________
>vvi-MIR397a [sc:448 + 59654 59771] [chr:Un_random + 85971396 85971513]
.......((((.((((((((((((((..(((.((((..((...((((((((.(.(((((...)))))...).))))))))..))))))).....)))..))))))))))))))).)))).            (-46.40)
GAAGAAAACATCATTGAGTGCAGCGTTGATGAAACTGAAGTATTCCATTTTTCAGCTTCTTTGAAGTCCGGCAAGATGGGTTCTGGTTGATTCCATTGGCGCTGCACTCAATCATGTC
            ATTGAGTGCAGCGTTGATGAA   714
             TTGAGTGCAGCGTTGATGAA   96
             TTGAGTGCAGCGTTGATGAAA  88
             TTGAGTGCAGCGTTGATGA    15
             TTGAGTGCAGCGTTGATGAAAC 13
```

```
                AGTGCAGCGTTGATGAA   6
            ATTGAGTGCAGCGTTGATGA    6
             TTGAGTGCAGCGTTGATG     3
          TCATTGAGTGCAGCGTTGATG     2
             TTGAGTGCAGCGTTGAT      1
            ATTGAGTGCAGCGTTGATGAAA  1
            ATTGAGTGCAGCGTTGATG     1
            ATTGAGTGCAGCGTTGA       1
           CATTGAGTGCAGCGTTGATGA    1

___________________________________
>vvi-MIR397b [sc:1477 - 246 363] [chr:Un_random - 146568042 146568159]
.......((((.(((((((((((((..(((.((((..((...((((((((.(.(((((...)))))...).)))))))))..))))))).....)))..)))))))))))))))).)))).            (-46.40)
GAAGAAAACATCATTGAGTGCAGCGTTGATGAAACTGAAGTATTCCATTTTTCAGCTTCTTTGAAGTCCGGCAAGATGGGTTCTGGTTGATTCCATTGGCGCTGCACTCAATCATGTC
            ATTGAGTGCAGCGTTGATGAA   714
             TTGAGTGCAGCGTTGATGAA   96
             TTGAGTGCAGCGTTGATGAAA  88
             TTGAGTGCAGCGTTGATGA    15
             TTGAGTGCAGCGTTGATGAAAC 13
            ATTGAGTGCAGCGTTGATGA    6
                AGTGCAGCGTTGATGAA   6
             TTGAGTGCAGCGTTGATG     3
          TCATTGAGTGCAGCGTTGATG     2
            ATTGAGTGCAGCGTTGA       1
             TTGAGTGCAGCGTTGAT      1
            ATTGAGTGCAGCGTTGATG     1
           CATTGAGTGCAGCGTTGATGA    1
            ATTGAGTGCAGCGTTGATGAAA  1

___________________________________
>vvi-MIR398a [sc:5 + 717225 717327] [chr:1 + 717225 717327]
...(((((((((.(((..((((((((((((..(.((((((((((.((........))))))))))))).)..))))))))))))))).))))))))).....   (-55.60)
ACACCCCAAGGGAGTGGCACCTGAGAACACCGGTTGTGTTGGCTGCGCCAAGCTCTGCTAGCTAATGCATTTTGTGTTCTCAGGTCACCCCTTTGGGGCACCA
                                                                                  TTCTCAGGTCACCCCTTTGGG          13
                                                                              TGTGTTCTCAGGTCACCCCTT              6
                                                                              TGTGTTCTCAGGTCACC                  1
                                                                             TTGTGTTCTCAGGTCACCC                 1

___________________________________
>vvi-MIR398b [sc:25 + 3324549 3324636] [chr:6 - 20660658 20660745]
..((((..((((.(((((((((((((.((((((...(((...........)))...)))))))))))))))))).)))))..)))))....       (-44.70)
GTGTCCTACAGGTGTGACCTGAGAATCACATGCCCGCACCACCCATCTTTGCTCTCATGTGTTCTCAGGTCGCCCCTGCTGGACTCCC
                                                         TGTGTTCTCAGGTCGCCCCTG     0
                                                     CTCATGTGTTCTCAGGTCGCC         15
                                                         TGTGTTCTCAGGTCGCCC        5
                                                          GTGTTCTCAGGTCGCCCCTGC    1
                                                         TGTGTTCTCAGGTCGCCCC       1
                                                        ATGTGTTCTCAGGTCGCCCC       1
                                                       CATGTGTTCTCAGGTCGCCCC       1
                                                      TCATGTGTTCTCAGGTCGCCC        1

___________________________________
>vvi-MIR398c [sc:15 - 78481 78568] [chr:6 + 14851882 14851969]
..((((..((((.(((((((((((((.((((((...(((...........)))...)))))))))))))))))).)))))..)))))....       (-45.80)
GTGTCCTACAGGAGTGACCTGAGAATCACATGCCCGCATCACCCATCTTTGCTCTCATGTGTTCTCAGGTCGCCCCTGCTGGACTCCC
                                                         TGTGTTCTCAGGTCGCCCCTG     0
                                                     CTCATGTGTTCTCAGGTCGCC         15
                                                         TGTGTTCTCAGGTCGCCC        5
                                                      TCATGTGTTCTCAGGTCGCCC        1
                                                       CATGTGTTCTCAGGTCGCCCC       1
                                                        ATGTGTTCTCAGGTCGCCCC       1
                                                         TGTGTTCTCAGGTCGCCCC       1
                                                          GTGTTCTCAGGTCGCCCCTGC    1

___________________________________
>vvi-MIR399a [sc:81 - 819715 819811] [chr:10 + 1853461 1853557]
..(((((((((.(..((((((((((((((((((.....(((...........)))......))))))))))))))))))..).)))))))))....          (-51.32)
GAGAATAACAGTGTGATTCTCCTTTGGCAGAGATATCACGCATGGAGTAGAAGGCGGTAGCTGCTCTGCCAAAGGAGAATTGCCCTGTTATTCAATT
                                                                   TGCCAAAGGAGAATTGCCCTG       106
                                                                   TGCCAAAGGAGAATTGCCC         4
                                                                   TGCCAAAGGAGAATTGCCCTGTT     3
                                                                   TGCCAAAGGAGAATTGCC          2
                                                                      CAAAGGAGAATTGCCCTG       1
                                                                   TGCCAAAGGAGAATTGCCCTGT      1
                                                                       AAAGGAGAATTGCCCTGT      1
                                                                       AAAGGAGAATTGCCCTGTT     1

___________________________________
>vvi-MIR399b [sc:376 + 84186 84302] [chr:Un_random + 77999653 77999769]
..((.((((((((((.(((((..(((((((((((..((..(((..((((......))))...)))..))...........))).))))))))..))))).)))))))))).))).... (-48.22)
```

```
ATCAATCATAGGGCACCTCTTTCTTGGCAGGCACTGGCTCCTATATATGAATATACATAGCTTAGCTGCAGTAAAAAGATGTGACTTGCCAAAGGAGAGTTGCCCTGTGACTGCTTC
                                                                                         TGCCAAAGGAGAGTTGCCCTG           34
                                                                                            CAAAGGAGAGTTGCCCTG           1
                                                                                         TGCCAAAGGAGAGTTGCCCTGT          1

__________________________________
>vvi-MIR399c [sc:37 - 1888940 1889021] [chr:15 + 5804593 5804674]
(((((.(.((((((..(((((..(((((((((.((((....)))..).)))))))))..)))))..)))))).).))))).. (-39.40)
ACCGGTCTCAGGGCCTCTTTCACTTGGTAGGTGACATAGATGTGAATGATCTGCCAAAGGAGAGTTGCCCTGTGTCTGGTTA
                                                     TGCCAAAGGAGAGTTGCCCTG           34
                                                        CAAAGGAGAGTTGCCCTG           1
                                                     TGCCAAAGGAGAGTTGCCCTGT          1

__________________________________
>vvi-MIR399d [sc:81 + 821136 821240] [chr:10 - 1852032 1852136]
.((((((...(((((((((((((((((((((((((........)))...(..(((((......)))))..)))))))))))))))))))))))...))))))... (-49.20)
GTAAATTATAGAGCAGATTTCTTTTGGCAGATGGCGATCACAAGCCAATGTGCTCAAAGGGCATTGAGTGTGTCTGCCAAAGGAGATTTGCTCGTGAATTTAACT
                                                                              TGCCAAAGGAGATTTGCTCGT           0

__________________________________
>vvi-MIR399e [sc:81 + 816930 817041] [chr:10 - 1856231 1856342]
..........((((((((.((((((((((((..((((((..((((....((........))..))))..)))).))..)))))))))))))))))))))............      (-42.10)
GCATATTACAGGGCAAATTATCTTTTGGCAGGCAGCCACTTAGAGACACAGCCAAGCCATGCATTCTTGTAGTGTGCCCTCTGCCAAAGGAGATTTGCCCGGCAATTCTTCT
                                                                                     TGCCAAAGGAGATTTGCCCGG    0

__________________________________
>vvi-MIR399f [sc:81 + 813348 813454] [chr:10 - 1859818 1859924]
((((((.....((((.((((((.((((((((.((((...(((..((((((........))))))))))))....)))))))).)))))))))))))))).......           (-38.60)
GTTGCATTAGAGGGCCAAATCTGCTTTGGCATGAAGCCCATTTAGCAGGCTCGTCTGTGTCTGCAGTCTTCTCGCTGCCGAAGGAGATTTGTCCTGCAATTCTTCTG
                                                                               TGCCGAAGGAGATTTGTCCTG          0

__________________________________
>vvi-MIR399g [sc:81 + 827902 828014] [chr:10 - 1845258 1845370]
.(((((((..((((((..((((.((((((((.(((...(((((((....)))))))).......(((.....)))..))).)))))))).))))..))))))..))))))...     (-48.80)
ATGAATTGCTGGGCAATACTCCATTGGCAGTTGGCCACTCGGCTGACCGCGGTTGACTTCACAAGTAGCAGACTAAGCTCACTGCCAAAGGAGATTTGCCCCTCAATTCAGCT
                                                                                      TGCCAAAGGAGATTTGCCCCT   0

__________________________________
>vvi-MIR399h [sc:81 - 825627 825716] [chr:10 + 1847556 1847645]
..((((..(((.(((((.((((((((((((((((..((((....))))...))).))))))))))))))).))))).)))..)))).....     (-41.10)
AGGAATAACAGTGCAATCCTCCTTTGGCAGAAAGATCATGCACATGCATACTTCTGTTTTGCCAAAGGAGAATTGCCCTGCCATTCGCTC
                                                           TGCCAAAGGAGAATTGCCCTG   106
                                                           TGCCAAAGGAGAATTGCCC     4
                                                           TGCCAAAGGAGAATTGCC      2
                                                              CAAAGGAGAATTGCCCTG   1

__________________________________
>vvi-MIR399i [sc:112 - 503240 503361] [chr:2 + 4040803 4040924]
((((((..((((((..((((((((.((((.(((((((((.(((((......))))).)).))))))))(((((((.........)))).))).)))))))))))))..))))))..)))))).        (-57.90)
AGTAGTTGTAGGGCTTCTCTCCTTCTGGCAGGAGATGGCAATAGATTATCCTTTGTGGCTTATCTCCGGTGTTGATTAACTTCCAATGACCCGCCAAAGGAGAGTTGCCCTGTGACTACTTC
                                                                                             CGCCAAAGGAGAGTTGCCCTG       0
                                                                                                CAAAGGAGAGTTGCCCTG       1

__________________________________
>vvi-MIR403a [sc:64 - 1835262 1835376] [chr:5 - 1835262 1835376]
.((...((((((((((((((((((((.(((.((.(((.(..(((((.((((....))))........)))))..)))).)).))).)))))))))))))))))..))))...)).   (-48.30)
GCACATCTCAAGTTTGTGCGTGAATCAAACCCCATCGTAGCCGTCCATATCTTCGGGTACTAATCACGGGGCGATTCGACGGCGTTAGATTCACGCACAAACTCGTGAGTTCTGA
                                                                                        TTAGATTCACGCACAAACTCG  0

__________________________________
>vvi-MIR403b [sc:64 - 1304545 1304630] [chr:5 - 1304545 1304630]
......((((((((((((((.(((((.((((((((..............)))))))).))))).))))))))))))))........         (-43.44)
ACAAACCTCGAGTTTGTGCGCGAATCCAACGCCTCGATCTTCTTTCAAAGGGGTGTTAGATTCACGCACAAACTCGGGATCTGTCT
                                                        TTAGATTCACGCACAAACTCG       0

__________________________________
>vvi-MIR403c [sc:64 - 1302086 1302200] [chr:5 - 1302086 1302200]
(((.(((.(.((((((((((((((((.(((..(.(((.(..(((((.((((....))))........)))))..)))).)..))).)))))))))))))))).).))).)))..   (-45.20)
GCACATCTCAAGTTTGTGCGTGAATCAAACCCCATCGTAGCCGTCCATATCTTCGGGTACTAATCACGGGGCGATTCGACGCCGTTAGATTCACGCACAAACTCGTGATCTGTCC
                                                                                        TTAGATTCACGCACAAACTCG  0

__________________________________
>vvi-MIR403d [sc:64 - 1740161 1740246] [chr:5 - 1740161 1740246]
......((((((((((((((.(((((..((((((..............))))))....))))).))))))))))))))........         (-40.24)
ACAAACCTCGAGTTTGTGCGCGAATCCAACCCCTCAATCTTCTTTCAAAGGGGTGTTAGATTCACGCACAAACTCGGGATCTGTCT
                                                        TTAGATTCACGCACAAACTCG       0
```

```
>vvi-MIR403e [sc:64 - 1738515 1738629] [chr:5 - 1738515 1738629]
(((.(((.(.((((((((((((((((.(((..(.(((.(..(((((.......(((.......)))))))))..)))).)..))).))))))))))))))).).))).)))..   (-44.31)
GCACATCTCAAGTTTGTGCGTGAATCAAACCCCATCGTAGCCGTCCATATCTTCGTGTACTAATCACGGGGCGATTCGACGCCGTTAGATTCACGCACAAACTCGCGATCTGTCC
                                                                                     TTAGATTCACGCACAAACTCG  0

>vvi-MIR403f [sc:42 - 1340023 1340130] [chr:7 - 4539818 4539925]
..((((.(.((((((((((((((.(((((...(((...(((.............))))))..(((........))).))))).))))))))))))))).).)))))....           (-36.92)
TGAGATGAGGAGTTTGTGCGTGACTCTAAAACCGCCTGATCTAAACCTTTTCCCGATGCGATACCTTTTCTATGGTCTTAGATTCACGCACAAACTCGTAATCTGTCT
                                                                              TTAGATTCACGCACAAACTCG         0

>vvi-MIR408 [sc:42 + 2173285 2173391] [chr:7 + 5373080 5373186]
.((((..((((((((((.((((((((((((((.((((.(....(((((...........)))))...).)))).)))))))))))))).))))))..)))..))))).            (-51.90)
AAGAGGAAGACGGGGACGAGGTAGTGCATGGATGGAACTATTAACAGAAGAATGTTAAGCTGTTTTTGCTCTACCCATGCACTGCCTCTTCCCTGGCTCTGTCTCTC
                                                                              TGCACTGCCTCTTCCCTGGC          118
                                                                              TGCACTGCCTCTTCCCTGGCT         4
                                                                               GCACTGCCTCTTCCCTGGC          2
                                                                              TGCACTGCCTCTTCCCTGGCTC        2
                                                                                ACTGCCTCTTCCCTGGC           1
                                                                              TGCACTGCCTCTTCCCTGG           1
                                                                             ATGCACTGCCTCTTCCCTGGC          1

>vvi-MIR477 [sc:166 + 518573 518666] [chr:1 + 15382675 15382768]
.(((((..(((..(((((((....(((((((((((((.(.((........)).).))))))))))))))....)))))))..)))..))))))... (-40.30)
GAAGTTGTCAATCTCCCTCAAAGGCTTCCAATTTTCCTGGCTGATTATCAAGGTTGGAAAGTTGGAAGACAGTGGGGGACCTTGGAAGCTTACC
          ATCTCCCTCAAAGGCTTCCAA     0

>vvi-MIR479 [sc:86 + 1068353 1068446] [chr:16 + 7689894 7689987]
(((.(((.((.((((((((((((((((((.((((.......................)))).)))))))))))))))))).))))).)))... (-38.56)
GTAGACATGGTGTGGTATTGGTTCGGCTCATCTTCTTTCATTCATTTTCATCGGCTCAAAGACGAGCCGAACCAATATCACTCTTGTATGCTTC
          TGTGGTATTGGTTCGGCTCATC    2

>vvi-MIR482 [sc:101 + 181595 181711] [chr:17 + 5579376 5579492]
......(((((((.((((((((((((((.(((((..((((..((((((((((........))))))....))))..))))))))).)))))))))).)))).))))))))........ (-51.80)
AATGTTTGGGAATTGGAGAGTAGGAAAGCTTAGCCATCTATTCCCTTCATGGTTTCCTCTCCATGGATTGGGGGTCTAGAGCTAGTCTTTCCTACTCCTCCCATTCCTATTGTTTTC
                                                                                                  CCTACTCCTCCCATTCC            0

          AATTGGAGAGTAGGAAAGCTT     2

>vvi-MIR535a [sc:392 - 4679 4779] [chr:Un_random - 82149759 82149859]
....((((..(((((.((((((((((((((((.((..(((.(((((.......))))).)))..)).)))))))))))))))).))))).)))).......     (-51.40)
TGTAATTGCTTGACAACGAGAGAGAGCACGCTAGTCAGCATTCATGCGAGGAACATGACTGTGTACAGGCGTGCTCTCTCTCGCTGTCATACAATCTTCTC
          TGACAACGAGAGAGAGCACGCT    0

>vvi-MIR535b [sc:392 - 4670 4770] [chr:Un_random - 88067433 88067533]
....((((..(((((.((((((((((((((((.((..(((.(((((.......))))).)))..)).)))))))))))))))).))))).)))).......     (-51.40)
TGTAATTGCTTGACAACGAGAGAGAGCACGCTAGTCAGCATTCATGCGAGGAACATGACTGTGTACAGGCGTGCTCTCTCTCGCTGTCATACAATCTTCTC
          TGACAACGAGAGAGAGCACGCT    0

>vvi-MIR535c [sc:1309 - 299 399] [chr:Un_random - 143826712 143826812]
....((((..(((((.((((((((((((((((.((..(((.(((((.......))))).)))..)).)))))))))))))))).))))).)))).......     (-51.40)
TGTAATTGCTTGACAACGAGAGAGAGCACGCTAGTCAGCATTCATGCGAGGAACATGACTGTGTACAGGCGTGCTCTCTCTCGCTGTCATACAATCTTCTC
          TGACAACGAGAGAGAGCACGCT    0

>vvi-MIR535d [sc:1190 - 13719 13819] [chr:Un_random - 103652414 103652514]
....((((..(((((.(((.((((((((((((.((..(((.(((((.......))))).)))..)).))))))))))))).))).))))).)))).......     (-47.80)
TGTAATTGCTTGACAACGAGAGAGAGCACGCTAGTCTGCATTCATGCGAGGAACATGACTGTGTACAGGCGTGCTCTCTGTCGCTGTCATACAATCTTCTC
          TGACAACGAGAGAGAGCACGCT    0

>vvi-MIR535e [sc:1190 - 13719 13819] [chr:Un_random + 105371228 105371328]
....((((..(((((.(((.((((((((((((.((..(((.(((((.......))))).)))..)).))))))))))))).))).))))).)))).......     (-47.80)
TGTAATTGCTTGACAACGAGAGAGAGCACGCTAGTCTGCATTCATGCGAGGAACATGACTGTGTACAGGCGTGCTCTCTGTCGCTGTCATACAATCTTCTC
          TGACAACGAGAGAGAGCACGCT    0

>vvi-MIR828a [sc:86 + 1219681 1219802] [chr:16 + 7841222 7841343]
..(((.(((.(((.((((((((((((((..(((.((((((((((((((..(.(((((....))))).)..)).)))...)).)))))))))..))))))))))))).))).))).)))......        (-42.20)
```

TATAAAGTTTCTTGCTCAAATGAGTATTCCAAACAACAGCTTGTGAAGCAATGATTACTGTCATAGTCTCTGCATGTGGATTGTTGTTGAGATGCTCATTTGAGGAAGCAACCTTAAAAATG
TCTTGCTCAAATGAGTATTCCA 0

____
>vvi-MIR828b [sc:5 + 2945837 2945908] [chr:1 + 2945837 2945908]
...((((((((..(((..((((((.(((.(((........)))))).)))))))...)))))))))))..... (-21.00)
GCTCTTGGTTTCTTGCTCAAATGAGTGTTCCACATCAACCTGGATAGTCATTTCCTGCAAAACCAGGTTTCT
TCTTGCTCAAATGAGTGTTCCA 0

____
>vvi-MIR845a [sc:86 + 3156 3597] [chr:16 + 6624697 6625138]
.....(((.(((((((...(((.((((((((((.............(((.(((...((((......(((.((((......)))))))).....))))...((.....)).))).)))...(((((...(((((((..((((((..((((((((((((((.((((((((((...((((.(((((.((.......(.(((((((.((((((...(((....((((((((.....)))))).
..))))))..)))))).)))))))).)...)).)))))).....(..(((.(((.....))).)))..)..))))..)))))))))).......((((..(((........)))..)))))))))))))))))))))))))))))..))..))))))...)))))))...)))))....))))......)))))))))....)))))))......))).. (-108.60)
TTTCACTTGATTAGTTCCTTCATGAGTTATTGCAAGTAATCTCCAAATTTCCTTAGCGGATTTACATTGACATATTCTATTATATTCATTCCTATCCATAGCACATTGCAAAGTAAAAACGACTTTAGCATTTAATTGAAAATTTCTTCTATCAAGCTCATTCCATTCTTGCTTGGGTTTTGGAACCAAAATTCCATCAACTAATTTAGTGGGTAAAGTTGGGCCATCTTCAATGGCA
TCCCATACATCTAAATCAGTTGATTGTAAGTACCAAGTCATTCTAGCTTTCCAATAGGGATAGTCGGTTCCCGTAAAGAATGGAGCTCTATGTTTTGAACAATTTTCAGTTTGAGATGAGCTTGATGGAATAGCCATTTTCCTCTTAGATGATTAAGTCTTAAGCAAGAGGTCTAGCTCTGATACCAATTGATAAAACAAAGGC

TAGCTCTGATACCAATTGATA 0

____
>vvi-MIR845b [sc:216 + 313861 314302] [chr:Un_random + 53496712 53497153]
.....(((.(((((((...(((.((((((((((..((.....................(((((..(((.((((......)))))))............((.....))...)))))...(((((...(((((((..((((((((.((((((((((((.(((((((((((((((((((((((....)))))))).(((((((.((((((((((...((((((.....))))))....
)))))....))))))).))))))))...))))......................((((........))))...))))))))))).......((((..(((........)))..)))))))))))))))))))))))))))))).))..))))))...)))))))...)))))....))..)))))..))))))))....)))))))......))).. (-106.10)
TTTCACTTGATTAGTTCCTTCATGAGTTATTTCAAGCAATCTCCAAATTTCCTTAGCAGATTTACATTGGCATATTCTATTATATTCATTCCTATCCATAGCACATTGCAAAGTAAAAACGACTTTAGCATTTAATTGAAAATTTCTTCTATCAAGCTCATTCCATTCTTGCTTGAGTTTTGGAACCAAAACTCCATCAACTAATTTAGTGGGAAAAGTTGGGCCATCTTCAATGACA
TCCCATACATCTAAATCAGTTGATTGTAAGTACCAAGTCATTCTAACTTTCCAATAGGGATAGTCGGTTCCCGTAAAGAATGGAGCTCTATGTTTTGAACAATTTTCAGTTTGAGATGAGCTTGATGGAACAACCATTTTCCTCTTAGATGATTAAGTCTTAAGCAAGAGGTCTAGCTCTGATACCAATTGATAAAACAAAGGC

TAGCTCTGATACCAATTGATA 0

____
>vvi-MIR845c [sc:31 - 2686178 2686267] [chr:7 + 503529 503618]
...((((((((((((((.(((.((((((((((((...((((.......)))).)))))))))))))).))))))))..))))))))))..... (-30.30)
CAATTATGTGTTTTGGTTGTCTGAGTTTCATGTTGGCAATTTCTTAATGATTAAACATGAGGCTCTGATACCAATTGATGCATAAACCAT
AGGCTCTGATACCAATTGATG 0

____
>vvi-MIR845d [sc:30 - 551951 552081] [chr:Un_random - 108642758 108642888]
....((..((((((((.....(((((((((((..............(((..(((((..((((......))))..)))))...))).........)))))))))))).....))))))))))........ (-32.25)
ATAACCAACATTAATTATCCCTAGGGCCATGGGATAAACAAAAACTCTGCAAAACCAAGTTAGCACACCCTAGAATGGTTCTAGCACAATTACTAACCTATGGCTCTGATACCAATTGATGGGAAAAACCT
TGGCTCTGATACCAATTGATG 0

____
>vvi-MIR845e [sc:168 + 268220 268350] [chr:Un_random + 37572078 37572208]
........((((((((.....((((((((((((............((((......))))..........(((((.....)))))............)))))))))))).....)))))))).......... (-31.30)
AGAACGCACATTAATTATCCCTAGGGCCATGGGATAAACAAAAACCTTGAAAAAACAAGTTAGCACACCCTAGAATGGTTCTAGCACGATTACTAACCTATGGCTCTGATACCAATTGATGGGAAAAACTC
TGGCTCTGATACCAATTGATG 0

Supplemental Figure S4: predicted transcript and intron coordinates for 25 *Vitis vinifera* pri-miRNAs. For each miRNA genomic locus showing extensive coverage with Illumina RNAseq reads, Transcription start and (where possible) stop coordinates were estimated from read coverage. Introns were predicted as explained in the main text. The table shows transcript coordinates estimated from different tissues, frequencies of support for introns(by tissue), splice site scores, promoter coordinates estimated by TSS-TCM, and notes showing additional support obtained for intronsand transcrtipt coordinates

croRNA	Chr	strand	TSS(TSSP-TCM)	TATA(TSSP-TCM)	Leaf start	Stem start	Root start	Callus start	Leaf Stop	Stem stop	Root stop	Callus stop	donor	donor score	donor support	acceptor	acceptor score	acceptor support	leaf	stem	root	callus	f reads	Additional Info (support for introns, RACE start/stop
iR156d	chr11	-	5634584	TATA-less	5634380	5634415	5634364	5634353	5631341	5631328	5633903	nd												
													5633555	0.571000		5633474	0.330000		0	2	0	0	2	
													5633512	0.389000		5631712	0.164000	leaf callus	2	0	0	0	1	
													5633184	0.370000	stem	5631712	0.164000	leaf callus	1	0	0	0	1	
													5632979	0.593000		5632856	0.392000	stem	0	7	0	0	3	
													5632655	0.606000		5632529	0.426000	leaf stem	2	17	0	1	10	
													5631801	0.328000		5631712	0.164000	leaf callus	1	0	0	0	1	
iR156g	chr17	-	No pred	no	3038168	3037793	nd	3038273	3036995	3037004	nd	3037013												
													3038152	0.355000		3037280	0.490000	stem callus	2	0	0	0	1	
													3037895	0.524000		3037280	0.490000	stem callus	1	0	0	1	2	454 leaf
													3037783	0.430000	stem callus	3037280	0.490000	stem callus	3	1	0	0	2	
													3037711	0.555000		3037280	0.490000	stem callus	12	3	0	0	8	
													3037596	0.401000	stem callus	3037280	0.490000	stem callus	1	0	0	0	1	
													3037392	0.503000	stem callus	3037280	0.490000	stem callus	0	4	0	1	4	
miR156i	chrUn_random	+	36776661	36776634	36776678	36776678	36776742	36776743	nd	nd	nd	nd												
iR159c	chr17	-	No pred	no	2610336	2610326	2610317	2610317	2609123	2609131	nd	2609196												
													2610173	0.508000		2609343	0.374000	leaf stem root	22	8	0	2	8	
iR160c	chr10	-	8894350	8894374	8894318	8894386	8894350	8894371	nd	nd	nd	nd												
													8893990	0.577000		8893881	0.505000	leaf stem root callus	0	3	1	0	4	
miR162	chr17	+	4714318	TATA-less	4714680	4714686	4714687	4714687	4717308	4717340	4717273	4717307												RACE STOP: 4717237/4715594/4715751/4717045/4716530
													4716567	0.501000		4716673	0.490000		4	1	0	0	2	
													4715520	0.504000		4716673	0.490000		0	2	1	1	3	454 berry/RACE
													4715520	0.504000		4716420	0.366000		0	2	0	0	2	
													4715510	0.471000		4716673	0.490000		0	1	0	0	1	RACE
													4715507	0.501000		4716673	0.490000		0	1	0	0	1	
													4715476	0.440000		4716673	0.490000		1	0	0	0	1	454 leaf
													4715350	0.373000		4715429	0.481000		3	0	0	0	1	
													4714863	0.390000		4716240	0.244000		0	1	0	0	1	
													4714768	0.407000		4715429	0.481000		3	1	0	0	2	
													4714759	0.377000		4716673	0.490000		0	0	0	1	1	
													4714759	0.377000		4715429	0.481000		5	3	0	0	4	454 leaf
													4714740	0.470000		4715429	0.481000		3	1	0	0	3	
													4714729	0.426000		4715429	0.481000		3	2	0	1	6	
iR167a	chr1	+	No pred	no	1563294	nd	1563320	1563291	1563916	nd	1563933	1563912												
iR167d	chr7	-	845566	845597	845542	845545	nd	nd	844270	844250	nd	nd												
													844837	0.489000	leaf	844632	0.370000	stem	0	1	0	0	1	
miR168	chr2	-	16750258	16750282	16750255	16750240	16750225	16750239	16747427	16747408	nd	16747419												
													16750027	0.467000		16748197	0.506000	leaf root	2	0	0	0	1	454 berry
													16750019	0.448000		16748197	0.506000	leaf root	4	8	1	5	6	454 berry
													16748088	0.482000		16747680	0.448000	leaf stem callus	0	1	0	0	1	
iR169g	chr8	+	No pred	no	20118544	nd	nd	nd	20119875	nd	nd	nd												
iR171a	chr14	-	No pred	no	14694238	14694267	14694292	14694236	nd	14693362	nd	nd												
iR319c	chr2	-	No pred	no	nd	662167	nd	nd	nd	660898	nd	nd												
iR393b	chr13	+	No pred	no	4372918	4372919	nd	nd	4373963	4373978	nd	nd												
													4373255	0.551000		4373664	0.402000		1	0	0	0	1	
iR394b	chr18	-	1386133	TATA-less	1386084	1386013	nd	nd	1385071	1384873	nd	nd												RACE STOP 1385081/1385085/1385121/1385102/1385108
													1385724	0.510000		1385362	0.467000	leaf stem callus	3	11	0	1	7	RACE
iR396b	chr11	+	No pred	no	5205873	5205921	5205886	5205883	5208124	5208135	5208011	5208094												
													5206245	0.545000		5206387	0.369000	stem callus	3	1	1	0	5	
													5206451	0.542000		5207749	0.404000		0	1	0	1	2	
													5206513	0.439000		5207086	0.171000		0	1	0	0	1	
													5207688	0.409000	leaf	5207749	0.404000		0	0	0	1	1	
													5207750	0.538000		5207816	0.363000	leaf	0	1	0	0	1	
iR397a	chrUn_random	+	No pred	no	85971353	85971349	85971353	85971342	nd	nd	nd	nd												
													85971560	0.435000		85971702	0.385000		0	1	0	0	1	
													85972847	0.571000		85972917	0.439000	stem root callus	0	5	0	40	11	
													85973070	0.598000		85973212	0.454000	stem callus	0	2	0	0	2	
iR397b	chrUn_random	-	No pred	no	146568205	146568210	146568207	146568217	nd	nd	nd	nd												
													146567996	0.435000		146567862	0.385000		0	1	0	2	2	
iR398a	chr1	+	No pred	no	717331	717187	717264	717248	719184	717198	719188	719200												
													717390	0.499000		717577	0.314000	leaf stem root callus	0	2	1	1	4	
													717390	0.499000		717687	0.351000		0	1	0	0	1	
													717390	0.499000		718148	0.353000	leaf stem root callus	0	1	0	0	1	
													717855	0.510000		718148	0.353000	leaf stem root callus	2	15	2	4	11	
													718282	0.533000		718370	0.390000	leaf stem root callus	2	8	0	4	8	
													718500	0.479000		719049	0.363000	leaf stem root callus	0	0	0	1	1	
iR398b	chr6	-	No pred	no	20660773	20660783	20660768	20660767	20660459	20660448	nd	20660443												
iR398c	chr6	+	No pred	no	14851851	14851857	14851856	14851858	14852180	14852215	nd	14852186												
iR403a	chr5	-	1835411	1835441	nd	1835395	1835420	1835389	nd	nd	nd	nd												
													1834959	0.568000		1834086	0.411000		0	4	0	0	2	454 leaf
													1835273	0.431000		1835195	0.349000		0	2	0	0	2	
iR403c	chr5	-	1302236	1302265	1302234	1302219	1302219	1302232	1301756	1301756	1301760	1301760												
													1302097	0.365000		1302012	0.349000		0	2	0	0	2	
iR403e	chr5	-	No pred	no	1738240	1738247	1738248	1738231	nd	nd	nd	nd												
													1737325	0.632000		1737240	0.404000		1	2	0	8	8	
													1738206	0.568000		1737402	0.303000		1	1	1	0	3	
miR408	chr7	+	No pred	no	5372904	5372907	5372907	5372896	5374243	5374226	5374255	5374207												
													5373063	0.555000		5373688	0.179000	leaf root	2	1	0	0	2	
													5373063	0.555000		5373709	0.261000	root	0	0	0	1	1	
													5373063	0.555000		5373745	0.303000		0	2	0	0	2	
													5373063	0.555000		5373858	0.327000	root callus	208	353	143	195	31	
iR482a	chr17	+	No pred	no	5579315	5579315	5579315	5579315	5580798	5580493	5580438	5581255												RACE START: 5579321 RACE STOP: 5580180
													5579322	0.514000	leaf stem root callus	5579599	0.482000	leaf stem root callus	1	0	0	0	1	
													5579346	0.367000	leaf stem root callus	5579599	0.482000	leaf stem root callus	5	0	0	0	2	
													5579360	0.390000	stem callus	5579599	0.482000	leaf stem root callus	0	1	0	0	1	

Supplemental Figure S5: RNAseq reads Supporting predicted splice junctions for *Vitis vinifera* pri-miRNAs shown in Figure 4

```
Vvi-MIR162      chr17   +      4716567                          4716673
CCCCAGGCAGCAAAATTTAGTGTTTCCACAGGTTGCATTTTTG ........ TTTCTTGAGCAGGTATCTGGAATCGGAAAGTTGTTTCTTGTTT
          CAAAATTTAGTGTTTCCACAG                                  GTATCTGGA                    3
         GCAAAATTTAGTGTTTCCACAG                                  GTATCTGG                     2

Vvi-MIR162      chr17   +      4715520                          4716673
GCCAAATTCTTGCTGAATGTAGTAATTTCCAGTAAATTTTAAT ........ TTTCTTGAGCAGGTATCTGGAATCGGAAAGTTGTTTCTTGTTT
        CTTGCTGAATGTAGTAATTTCCA                                  GTATCTGGA                    1
              GAATGTAGTAATTTCCA                                  GTATCTGGAATCG                1
           GCTGAATGTAGTAATTTCCA                                  GTATCTGGAA                   2

Vvi-MIR162      chr17   +      4715520                         4716420
GCCAAATTCTTGCTGAATGTAGTAATTTCCAGTAAATTTTAAT ........ GTTCTTGAGTAGGGGGGATAAGGCTGCTGGTTTTGCGAAGTGC
       TCTTGCTGAATGTAGTAATTTCCA                                  GGGGGA                       1
               AATGTAGTAATTTCCA                                  GGGGGATAAGGCTG               1

Vvi-MIR162      chr17   +      4715510                          4716673
GCATCACAACGCCAAATTCTTGCTGAATGTAGTAATTTCCAGT ........ TTTCTTGAGCAGGTATCTGGAATCGGAAAGTTGTTTCTTGTTT
                 TCTTGCTGAATGTA                                  GTATCTGGAATCGGAA             1

Vvi-MIR162      chr17   +      4715507                          4716673
GTAGCATCACAACGCCAAATTCTTGCTGAATGTAGTAATTTCC ........ TTTCTTGAGCAGGTATCTGGAATCGGAAAGTTGTTTCTTGTTT
       CACAACGCCAAATTCTTGCTGAAT                                  GTATCT                       1

Vvi-MIR162      chr17   +      4715476                          4716673
CTGTGTTCTTCTGTGTTTCGAACAGACTCTGGTAGCATCACAA ........ TTTCTTGAGCAGGTATCTGGAATCGGAAAGTTGTTTCTTGTTT
                      CAGACTCTG                                  GTATCTGGAATCGGAAAGTTG        1

Vvi-MIR162      chr17   +      4715350                          4715429
AGACCATGTTCACAAATAGTCTTGTAAAGCTGTAACAGCCTGA ........ AGTTTATTGCAGGGAAGGAGATCCGCCCTGTGTTCTTCTGTGT
              AATAGTCTTGTAAAGCT                                  GGAAGGAGATCCG                3
```

```
Vvi-MIR162      chr17   +      4714863                          4716240
TCATTTGGTCAGATCTGTGGTTTTTGATTTTGTGTTTTTGAAA ........ ACACTCCATAAGGTTTTTTAATTGGGTTAACTTCTATTCTCAT
       GTCAGATCTGTGGTTTTTGATTTT                                  GTTTTT                       1

Vvi-MIR162      chr17   +      4714768                          4715429
ATGGTGACCCTTCAGATTCCTGGTTCACGCTGTTACTCTTTCT ........ AGTTTATTGCAGGGAAGGAGATCCGCCCTGTGTTCTTCTGTGT
                 TCCTGGTTCACGCT                                  GGAAGGAGATCCGCCC             1
              GATTCCTGGTTCACGCT                                  GGAAGGAGATCCG                3

Vvi-MIR162      chr17   +      4714759                          4716673
CGTACGGCAATGGTGACCCTTCAGATTCCTGGTTCACGCTGTT ........ TTTCTTGAGCAGGTATCTGGAATCGGAAAGTTGTTTCTTGTTT
                    TCAGATTCCTG                                  GTATCTGGAATCGGAAAGTTG        1

Vvi-MIR162      chr17   +      4714759                          4715429
CGTACGGCAATGGTGACCCTTCAGATTCCTGGTTCACGCTGTT ........ AGTTTATTGCAGGGAAGGAGATCCGCCCTGTGTTCTTCTGTGT
                     CAGATTCCTG                                  GGAAGGAGATCCGCCCTGTG         2
              GACCCTTCAGATTCCTG                                  GGAAGGAGATCCG                3
            GTGACCCTTCAGATTCCTG                                  GGAAGGAGATC                  1
             TGACCCTTCAGATTCCTG                                  GGAAGGAGATCC                 2

Vvi-MIR162      chr17   +      4714740                          4715429
AGAGAGAGAGGGAGAAAAACGTACGGCAATGGTGACCCTTCAG ........ AGTTTATTGCAGGGAAGGAGATCCGCCCTGTGTTCTTCTGTGT
             GAAAAACGTACGGCAATG                                  GGAAGGAGATCC                 1
                   CGTACGGCAATG                                  GGAAGGAGATCCGCCCTG           1
            AGAAAAACGTACGGCAATG                                  GGAAGGAGATC                  2

Vvi-MIR162      chr17   +      4714729                          4715429
ATAGAGAAGGGAGAGAGAGAGGGAGAAAAACGTACGGCAATGG ........ AGTTTATTGCAGGGAAGGAGATCCGCCCTGTGTTCTTCTGTGT
        GGGAGAGAGAGAGGGAGAAAAAC                                  GGAAGGAGA                    1
                        GAAAAAC                                  GGAAGGAGATCCGCCCTGTGTTC      1
                GAGAGGGAGAAAAAC                                  GGAAGGAGATCCGCC              1
             AGAGAGAGGGAGAAAAAC                                  GGAAGGAGATCC                 1
              GAGAGAGGGAGAAAAAC                                  GGAAGGAGATCCG                1
            GAGAGAGAGGGAGAAAAAC                                  GGAAGGAGATC                  1
```

```
Vvi-MIR168      chr2    -      16750027                         16748197
ATGTGATGATGAAAGACTACTTCGATCTCAGGTTTCTAGGTTG ........ GCTTGTTTTCAGGTGCGGGGGCTCAACAAATTTGTTGCAGGGC
          GAAAGACTACTTCGATCTCAG                                  GTGCGGGGG                    2


Vvi-MIR168      chr2    -      16750019                         16748197
ATGAAAGACTACTTCGATCTCAGGTTTCTAGGTTGGAAAAATT ........ GCTTGTTTTCAGGTGCGGGGGCTCAACAAATTTGTTGCAGGGC
           CTTCGATCTCAGGTTTCTAG                                  GTGCGGGGGC                   5
                       GTTTCTAG                                  GTGCGGGGGCTCAACAAATTTG       3
        CTACTTCGATCTCAGGTTTCTAG                                  GTGCGGG                      4
                ATCTCAGGTTTCTAG                                  GTGCGGGGGCTCAAC              1
               GATCTCAGGTTTCTAG                                  GTGCGGGGGCTCAACA             2
                     AGGTTTCTAG                                  GTGCGGGGGCTCAACAAATT         3

Vvi-MIR168      chr2    -      16748088                         16747680
TCAACCCTAACAATTATTGTCACATGCCCAGGTTTCTTGGTAA ........ TTTATTCTGTAGATCATTGCATGATTGGCCCATTCTCCTCTCT
                      CATGCCCAG                                  ATCATTGCATGATTGGCCCAT   1

Vvi-MIR394B     chr18   -      1385724                          1385362
CTCTCTCGCTCTTCCACTCTAGAGCATCAAGGTGAAAACCCCA ........ CTTGTGTTGCAGGGGTTTCATCAACTCCTCCTCTTTGCCTCTT
                  CTAGAGCATCAAG                                  GGGTTTCATCAACTCCT            1
         TCTTCCACTCTAGAGCATCAAG                                  GGGTTTCATC                   1
                     GAGCATCAAG                                  GGGTTTCATCAACTCCTCCT         2
            TCCACTCTAGAGCATCAAG                                  GGGTTTCATCA                  1
                        CATCAAG                                  GGGTTTCATCAACTCCTCCTCTT      2
           TTCCACTCTAGAGCATCAAG                                  GGGTTTCATC                   5
                    AGAGCATCAAG                                  GGGTTTCATCAACTCCTCC          3
```
