# Supplementary material for: High throughput approaches reveal splicing of primary microRNA transcripts and tissue specific expression of mature microRNAs in Vitis vinifera
Source: BMC Genomics. 2009 Nov 25;10:558. doi: 10.1186/1471-2164-10-558 (PMC2822795; doi:10.1186/1471-2164-10-558)
Supplement: Additional file 2 — Oligonucleotide Array probes for analysis of Vitis vinifera miRNA expression. Supplemental Table S6: All oligonucleotide array probe sequences. [file 1471-2164-10-558-S2.pdf]

Supplemental Table S6. Combimatrix microarray spotted probes list.

Probe Name	Probe Sequence 5’-3'	Probe Type
vvi|tRNA-Ala(Chlor)_12_32|PosCtrl|as|	tcagttggtagagctccgctc	Positive control
vvi|tRNA-Phe(Chlor)_48_68|PosCtrl|as|	ccagttcaaatctggttcctg	Positive control
vvi|tRNA-Phe(Chlor)_37_57|PosCtrl|as|	tcctcgtgtcaccagttcaaa	Positive control
vvi|tRNA-Lys(Chlor)_2_23|PosCtrl|as|	ggttgctaactcaatggtagag	Positive control
vvi|tRNA-His(Chlor)_52_72|PosCtrl|as|	cgggttcaattcccgtcgttc	Positive control
vvi|tRNA-His(Chlor)_19_39|PosCtrl|as|	gatcaaggcagtggattgtga	Positive control
vvi|tRNA-Ser(Chlor)_51_71|PosCtrl|as|	agttattcgtaccgagggttc	Positive control
vvi|tRNA-Ser(Chlor)_38_59|PosCtrl|as|	aatccgttgtacgagttattcg	Positive control
vvi|tRNA-Tyr(Chlor)_43_63|PosCtrl|as|	gttggcaatatgtctacgctg	Positive control
vvi|tRNA-Tyr(Chlor)_4_24|PosCtrl|as|	tcgatgcccgagcggctaatg	Positive control
vvi|tRNA-Met(Chlor)_48_68|PosCtrl|as|	cacgggttcaaatcctgtctc	Positive control
vvi|tRNA-Met(Chlor)_33_53|PosCtrl|as|	tcataaccttgaggtcacggg	Positive control
vvi|tRNA-Ile(Chlor)_10_30|PosCtrl|as|	ctctggttcaagtccaggatg	Positive control
vvi|UBQ(AY684128)_74_94|DegradCtrl|as|	acttggtcctccgcctgagag	Degradation control
vvi|Act(AY847627)_170_190|DegradCtrl|as|	ttgtgcttagtggtgggtcga	Degradation control
vvi|Act(AY847627)_144_165|DegradCtrl|as|	tatcaggaaggacctctatggc	Degradation control
vvi|Hsp101(AY764155)_2888_2908|DegradCtrl|as|	gcagtgacctgtaattggtct	Degradation control
vvi|Hsp101(AY764155)_2660_2680|DegradCtrl|as|	tcagacgtgttgattcggatc	Degradation control
vvi|GDBrPK(AF178575)_1085_1106|DegradCtrl|as|	gtcagtggcattgattatcaga	Degradation control
vvi|GDBrPK(AF178575)_1072_1092|DegradCtrl|as|	cagtgcgttcatggtcagtgg	Degradation control
neg|Bio B Spike_394_418|NegCtrl|as|	gcgcctgagattcactcaacgtgcc	Negative control
neg|Bio C Spike_113_137|NegCtrl|as|	acgtgggtgtatttacgctgtggaa	Negative control
neg|Lambda Spike 12_609_633|NegCtrl|as|	cttccggcaatactcgtaaaccata	Negative control
neg|Lambda Spike 5_753_777|NegCtrl|as|	gaacaggttatcgaaatcagccaca	Negative control
neg|Lambda Spike 6_497_521|NegCtrl|as|	tacaaccggaatgttgaccttgcct	Negative control
neg|Lambda Spike 8_329_353|NegCtrl|as|	gtcacctttatctgccgccactcat	Negative control
neg|Lambda Spike 9_1167_1191|NegCtrl|as|	gatgtttgcagacgtaatggtgcgg	Negative control
QC-oligoB-AS-3	gagttgctgttacatcagcgaatgtgcatggaacacactt	Quality control
QC-3prime	tgactgactggactgtgggtgtgcgatacgtgtcc	Quality control

QC-5prime	tgggtgtgcgatacgtgtccgactgactgactgac	Quality control
QC-LLD-1	agatttcttcggtttcccgtggttggcgtctga	Quality control
QC-LLD-2	ggagtatgttcggctgcgcgagagctatgatgccg	Quality control
QC-LLD-3	gtggcgatgttaacctcggcttatggcgtcaccgg	Quality control
QC-LLD-4	cgaacatcccagggtaacacgca	Quality control
NC-SP-phage-03-3	caccgtaattactgtgagccatcatgacgccgatg	Generic negative control
NC-SP-phage-03-7	gacggtgatcgcatcggtgctgttaacaagttcat	Generic negative control
NC-SP-phage-03-8	agtggctgaaacagttgttgattgagtctctttgg	Generic negative control
NC-SP-phage-05-2	ccatgattacgccagttgtacggacacgaactcat	Generic negative control
NC-SP-phage-05-1	ggagacgggcaatcagttcatctttcgtcatggac	Generic negative control
NC-SP-phage-05-16	cctgtccggcatagctgatattttcagggtcatca	Generic negative control
NC-SP-phage-06-1	gactctccggattaactggcggtgacggtaatttc	Generic negative control
NC-SP-phage-06-8	tcatacataccagacagccggtacaccacgttttc	Generic negative control
NC-SP-phage-06-14	gtcacaccgttatgttcaaatgattcggttttcag	Generic negative control
NC-SP-phage-08-9	gtgaccataccgtacaggttagaaaccgtcagcgt	Generic negative control
NC-SP-phage-08-10	ggatcggcgtaactgtttccgttgacgaagttcac	Generic negative control
NC-SP-phage-08-13	ggacagaaagcgtcacgctgtacgttttcaggttg	Generic negative control
NC-SP-phage-09-4	cgaccaaatcgttgtaaatccccgtaaaggcagat	Generic negative control
NC-SP-phage-09-7	catcaggcagagtctcatgtaactgcgccgttaac	Generic negative control
NC-SP-phage-09-11	ctctgggaacaatatgaattacagcgccatcaggc	Generic negative control
NC-SP-phage-12-6	gttacggttgatttcgagttgggtccacttatcgc	Generic negative control
NC-SP-phage-12-14	ccagtaactggctgaacacaccgttgatgatctgc	Generic negative control
NC-SP-phage-12-15	catgttctgatacaggttggtaaccagccagtagt	Generic negative control
NC-bacteria-10-7	gaagaagaatcagggcgacacggtctccatttttg	Generic negative control
NC-bacteria-10-2	cgagcttcaacagcgtccagaataacggtgaacaa	Generic negative control
NC-bacteria-10-5	catgtatcttgcaaacggcaatcgacaccagtctg	Generic negative control
NC-bacteria-9-4	aaagggagctatgtcgagatttcttcggtttcccg	Generic negative control
NC-bacteria-9-9	gaagctgtcgctaactattcagtcaaagggagcta	Generic negative control
NC-bacteria-9-11	gaagtcttcgatgatggatttacgacggtatttca	Generic negative control
NC-bacteria-5-4	ataacgatggtggcgatgttaacctcggcttatgg	Generic negative control
NC-bacteria-5-11	gtaaggagcatcaaaagactgcggtcatctttggg	Generic negative control
NC-bacteria-5-12	aaatgtacaggcgaccagcttgttaacgatgacca	Generic negative control
NC-plant-1	cctaccgtaggtcgacaatatagatattttgttgg	Generic negative control
NC-plant-2	gcggaagaagttgtcggagcaatcgtcatgggaa	Generic negative control

NC-plant-3	tggtaaacagagttcctctaattgtcacagggaac	Generic negative control
NC-plant-4	agcagatctcaaagattgtaggagacatggacgtc	Generic negative control
NC-plant-5	cagcaagctctagaagttcagaggaggcaggagag	Generic negative control
NC-plant-6	ccaactagcgagtagaatcaactatcagcaatggc	Generic negative control
NC-plant-7	ggaagaagaaatacgcacaagtttgtgtcacaatg	Generic negative control
NC-plant-8	acattcgcaaggaaccgtgagctagaagttatcca	Generic negative control
vvi|snRNAU6|nat|as|	tgcgtgtcatccttgcgcagg	Custom Positive Control
vvi|snRNAU6|2mut|as|	tgcgtgtcattcttgcccagg	Custom Positive Control
scaffold_2358|+|1257|1330|10-30|tRNA|nat|as|	cactgccttgatccacttgg	Custom Positive Control
scaffold_2358|+|1257|1330|10-30|tRNA|2mut|as|	cactgtcttgatcaacttgg	Custom Positive Control
scaffold_105|-|365541|365614|10-30|tRNA|nat|as|	tagcgtgataaccaactaca	Custom Positive Control
scaffold_105|-|365541|365614|10-30|tRNA|2mut|as|	tagcctgataacaaactaca	Custom Positive Control
scaffold_111|+|86247|86318|10-30|tRNA|nat|as|	cccaccacgcttccgctgcg	Custom Positive Control
scaffold_111|+|86247|86318|10-30|tRNA|2mut|as|	cccaccacccttcccctgcg	Custom Positive Control
scaffold_834|-|23630|23701|10-30|tRNA|nat|as|	tcagacgcgctaccattgcg	Custom Positive Control
scaffold_834|-|23630|23701|10-30|tRNA|2mut|as|	tcagacgccctacaattgcg	Custom Positive Control
vvi|miR-1|nat|as|	gtgctccctctcttctgtca	vvi-miR156a_perfect match probe
vvi|miR-1|2mut|as|	gtgctctctctcttccgtca	vvi-miR156a_mismatch probe
vvi|miR-2|nat|as|	gtgctcactctcttctgtca	vvi-miR156b_perfect match probe
vvi|miR-2|2mut|as|	gtgctccctctcttccgtca	vvi-miR156b_mismatch probe
vvi|miR-3|nat|as|	gtgctcactctcctctgtca	vvi-miR156e_perfect match probe
vvi|miR-3|2mut|as|	gtgctccctcttctctgtca	vvi-miR156e_mismatch probe
vvi|miR-4|nat|as|	gtgctctctatcttctgtcaa	vvi-miR156f_perfect match probe
vvi|miR-4|2mut|as|	gtgctccctatcttctctcaa	vvi-miR156f_mismatch probe
vvi|miR-5|nat|as|	atgctctctctcttctgtca	vvi-miR156h_perfect match probe
vvi|miR-5|2mut|as|	atgctctccctcttccgtca	vvi-miR156h_mismatch probe
vvi|miR-6|nat|as|	gtgctctctatcttctgtca	vvi-miR156i_perfect match probe
vvi|miR-6|2mut|as|	gtgctccctatcttccgtca	vvi-miR156i_mismatch probe
vvi|miR-7|nat|as|	gagagctcccttcactccaag	vvi-miR159a_perfect match probe
vvi|miR-7|2mut|as|	gagagctctcttcacttcaag	vvi-miR159a_mismatch probe
vvi|miR-8|nat|as|	tgagagctcccttcactccaag	vvi-miR159b_perfect match probe
vvi|miR-8|2mut|as|	tgagagctctcttcacttcaag	vvi-miR159b_mismatch probe
vvi|miR-9|nat|as|	tagagctcccttcaatccaaa	vvi-miR159c_perfect match probe
vvi|miR-9|2mut|as|	tagagctctcttcaattcaaa	vvi-miR159c_mismatch probe

vvi|miR-10|nat|as|	tggcattcagggagccaggca	vvi-miR160a_perfect match probe
vvi|miR-10|2mut|as|	tggcattcagtgagtcaggca	vvi-miR160a_mismatch probe
vvi|miR-11|nat|as|	tggcatacagggagccaggca	vvi-miR160c_perfect match probe
vvi|miR-11|2mut|as|	tggcatacagtgagtcaggca	vvi-miR160c_mismatch probe
vvi|miR-12|nat|as|	ctggatgcagaggtttatcga	vvi-miR162_perfect match probe
vvi|miR-12|2mut|as|	ctggatgaagagctttatcga	vvi-miR162_mismatch probe
vvi|miR-13|nat|as|	tgcacgtgccctgcttctcca	vvi-miR164a_perfect match probe
vvi|miR-13|2mut|as|	tgcacgtgtcctccttctcca	vvi-miR164a_mismatch probe
vvi|miR-14|nat|as|	agcatgtgccctgcttctcca	vvi-miR164b_perfect match probe
vvi|miR-14|2mut|as|	agcatgtgtcctccttctcca	vvi-miR164b_mismatch probe
vvi|miR-15|nat|as|	gggggaatgaagcctggtccga	vvi-miR166c_perfect match probe
vvi|miR-15|2mut|as|	ggggaaatgaagtctggtccga	vvi-miR166c_mismatch probe
vvi|miR-16|nat|as|	aggggaatgaagcctggtccga	vvi-miR166d_perfect match probe
vvi|miR-16|2mut|as|	agggaaatgaagtctggtccga	vvi-miR166d_mismatch probe
vvi|miR-17|nat|as|	caggaatgaagcctggtccga	vvi-miR166a_perfect match probe
vvi|miR-17|2mut|as|	caggaatgaactctggtccga	vvi-miR166a_mismatch probe
vvi|miR-18|nat|as|	gaggaatgaagcctggtccga	vvi-miR166b_perfect match probe
vvi|miR-18|2mut|as|	gaggaatgaactctggtccga	vvi-miR166b_mismatch probe
vvi|miR-19|nat|as|	cagatcatgctggcagcttca	vvi-miR167a_perfect match probe
vvi|miR-19|2mut|as|	cagatcatcctgccagcttca	vvi-miR167a_mismatch probe
vvi|miR-20|nat|as|	tagatcatgctggcagcttca	vvi-miR167b_perfect match probe
vvi|miR-20|2mut|as|	tagatcatcctgccagcttca	vvi-miR167b_mismatch probe
vvi|miR-21|nat|as|	agatcatgctggcagcttca	vvi-miR167c_perfect match probe
vvi|miR-21|2mut|as|	agatcatcctgccagcttca	vvi-miR167c_mismatch probe
vvi|miR-22|nat|as|	ttcccgacctgcaccaagcga	vvi-miR168_perfect match probe
vvi|miR-22|2mut|as|	ttccagacctgaaccaagcga	vvi-miR168_mismatch probe
vvi|miR-23|nat|as|	ccggcaagtcatccttggctg	vvi-miR169a_perfect match probe
vvi|miR-23|2mut|as|	ccggaaagtcatccttcgctg	vvi-miR169a_mismatch probe
vvi|miR-24|nat|as|	taggcaagtcatccttcgcta	vvi-miR169y_perfect match probe
vvi|miR-24|2mut|as|	taggaaagtcatccttagcta	vvi-miR169y_mismatch probe
vvi|miR-25|nat|as|	cggcaagccatccttggctca	vvi-miR169b_perfect match probe
vvi|miR-25|2mut|as|	cggcaagtcatccttgcctca	vvi-miR169b_mismatch probe
vvi|miR-26|nat|as|	ccggcaaatcattcttggctg	vvi-miR169d_perfect match probe
vvi|miR-26|2mut|as|	ccggaaaatcattcttcgctg	vvi-miR169d_mismatch probe

vvi|miR-27|nat|as|	caggcaagtcatccttggcta	vvi-miR169e_perfect match probe
vvi|miR-27|2mut|as|	caggaaagtcatccttcgcta	vvi-miR169e_mismatch probe
vvi|miR-28|nat|as|	tcggcaagtcatccttggctg	vvi-miR169f_perfect match probe
vvi|miR-28|2mut|as|	tcggaaagtcatccttcgctg	vvi-miR169f_mismatch probe
vvi|miR-29|nat|as|	ggcaagtcatccttggctg	vvi-miR169g_perfect match probe
vvi|miR-29|2mut|as|	ggcaagccatccttcgctg	vvi-miR169g_mismatch probe
vvi|miR-30|nat|as|	acggccagtcatccttggctc	vvi-miR169i_perfect match probe
vvi|miR-30|2mut|as|	acggtcagtcatccttcgctc	vvi-miR169i_mismatch probe
vvi|miR-31|nat|as|	acggcaagtcatccttggctc	vvi-miR169l_perfect match probe
vvi|miR-31|2mut|as|	acggaaagtcatccttcgctc	vvi-miR169l_mismatch probe
vvi|miR-32|nat|as|	ccggcaagtcatccttggctc	vvi-miR169m_perfect match probe
vvi|miR-32|2mut|as|	ccggaaagtcatccttcgctc	vvi-miR169m_mismatch probe
vvi|miR-33|nat|as|	gcggcaagtcatccttggctc	vvi-miR169o_perfect match probe
vvi|miR-33|2mut|as|	gcggaaagtcatccttcgctc	vvi-miR169o_mismatch probe
vvi|miR-34|nat|as|	cggcaagtcatccttgactca	vvi-miR169r_perfect match probe
vvi|miR-34|2mut|as|	cggcaagccattcttgactca	vvi-miR169r_mismatch probe
vvi|miR-35|nat|as|	cggcaagtcatccttgactcg	vvi-miR169s_perfect match probe
vvi|miR-35|2mut|as|	cggcaagccattcttgactcg	vvi-miR169s_mismatch probe
vvi|miR-36|nat|as|	ccggcaattcatccttggctt	vvi-miR169v_perfect match probe
vvi|miR-36|2mut|as|	ccggaaattcatccttcgctt	vvi-miR169v_mismatch probe
vvi|miR-37|nat|as|	taggcaagtcatccttggcta	vvi-miR169x_perfect match probe
vvi|miR-37|2mut|as|	taggaaagtcatccttcgcta	vvi-miR169x_mismatch probe
vvi|miR-38|nat|as|	cgtgatgttgggacggctcaa	vvi-miR171a_perfect match probe
vvi|miR-38|2mut|as|	cgtgatgttgtgacgcctcaa	vvi-miR171a_mismatch probe
vvi|miR-39|nat|as|	gatattgacgcggctcaatca	vvi-miR171b_perfect match probe
vvi|miR-39|2mut|as|	gatattgacgagcctcaatca	vvi-miR171b_mismatch probe
vvi|miR-40|nat|as|	cgtgatattggcacggctcaa	vvi-miR171c_perfect match probe
vvi|miR-40|2mut|as|	cgtgatattgccacgcctcaa	vvi-miR171c_mismatch probe
vvi|miR-41|nat|as|	gatattggcgcggctcaatca	vvi-miR171e_perfect match probe
vvi|miR-41|2mut|as|	gatattggcgagcctcaatca	vvi-miR171e_mismatch probe
vvi|miR-42|nat|as|	agtgatattggcgcggctcaa	vvi-miR171f_perfect match probe
vvi|miR-42|2mut|as|	agtgatattgccgaggctcaa	vvi-miR171f_mismatch probe
vvi|miR-43|nat|as|	ggtgatattggttcggctcaa	vvi-miR171g_perfect match probe
vvi|miR-43|2mut|as|	ggtgatattcgttcgcctcaa	vvi-miR171g_mismatch probe

vvi|miR-44|nat|as|	gatattggcgcggctcaacca	vvi-miR171h_perfect match probe
vvi|miR-44|2mut|as|	gatattggcgagcctcaacca	vvi-miR171h_mismatch probe
vvi|miR-45|nat|as|	gatgatattggcacggctcaa	vvi-miR171i_perfect match probe
vvi|miR-45|2mut|as|	gatgatattgccacgcctcaa	vvi-miR171i_mismatch probe
vvi|miR-46|nat|as|	atgtagcatcatcaagattca	vvi-miR172a_perfect match probe
vvi|miR-46|2mut|as|	atgtagaatcataaagattca	vvi-miR172a_mismatch probe
vvi|miR-47|nat|as|	gtgtagcatcatcaagattca	vvi-miR172b_perfect match probe
vvi|miR-47|2mut|as|	gtgtagaatcataaagattca	vvi-miR172b_mismatch probe
vvi|miR-48|nat|as|	ctgcagcatcatcaagattcc	vvi-miR172c_perfect match probe
vvi|miR-48|2mut|as|	ctgcagaatcataaagattcc	vvi-miR172c_mismatch probe
vvi|miR-49|nat|as|	atgcagcatcatcaagattct	vvi-miR172d_perfect match probe
vvi|miR-49|2mut|as|	atgcagaatcataaagattct	vvi-miR172d_mismatch probe
vvi|miR-50|nat|as|	gggagctcccttcagtccaag	vvi-miR319b_perfect match probe
vvi|miR-50|2mut|as|	gggagctctcttcagttcaag	vvi-miR319b_mismatch probe
vvi|miR-51|nat|as|	aggagctcccttcagtccaaa	vvi-miR319e_perfect match probe
vvi|miR-51|2mut|as|	aggagctctcttcagttcaaa	vvi-miR319e_mismatch probe
vvi|miR-52|nat|as|	gggagctcccttcagtccaat	vvi-miR319g_perfect match probe
vvi|miR-52|2mut|as|	gggagctctcttcagttcaat	vvi-miR319g_mismatch probe
vvi|miR-53|nat|as|	ggcgctatccctcctgagctt	vvi-miR390_perfect match probe
vvi|miR-53|2mut|as|	ggcgctatctcttctgagctt	vvi-miR390_mismatch probe
vvi|miR-54|nat|as|	ggatcaatgcgatccctttgga	vvi-miR393a_perfect match probe
vvi|miR-54|2mut|as|	ggatcaatgagatctctttgga	vvi-miR393a_mismatch probe
vvi|miR-55|nat|as|	ggaggtggacagaatgccaa	vvi-miR394a_perfect match probe
vvi|miR-55|2mut|as|	ggaggtcgacagaatcccaa	vvi-miR394a_mismatch probe
vvi|miR-56|nat|as|	gagttcccccaaacacttcag	vvi-miR395a_perfect match probe
vvi|miR-56|2mut|as|	gagttccctcaaacccttcag	vvi-miR395a_mismatch probe
vvi|miR-57|nat|as|	gagttcctccagactcttcag	vvi-miR395n_perfect match probe
vvi|miR-57|2mut|as|	gagttcctcaagcctcttcag	vvi-miR395n_mismatch probe
vvi|miR-58|nat|as|	aagctcaagaaagctgtgggag	vvi-miR396a_perfect match probe
vvi|miR-58|2mut|as|	aagctaaagaaagttgtgggag	vvi-miR396a_mismatch probe
vvi|miR-59|nat|as|	aagttcaagaaagctgtgggaa	vvi-miR396b_perfect match probe
vvi|miR-59|2mut|as|	aagttaaagaaagttgtgggaa	vvi-miR396b_mismatch probe
vvi|miR-60|nat|as|	cagttcaagaaagctgtggaa	vvi-miR396c_perfect match probe
vvi|miR-60|2mut|as|	cagttaaagaaagttgtggaa	vvi-miR396c_mismatch probe

vvi|miR-61|nat|as|	catcaacgctgcactcaatga	vvi-miR397a_perfect match probe
vvi|miR-61|2mut|as|	catcaaccctgaactcaatga	vvi-miR397a_mismatch probe
vvi|miR-62|nat|as|	aaggggtgacctgagaacaca	vvi-miR398a_perfect match probe
vvi|miR-62|2mut|as|	aaggtgtgacccgagaacaca	vvi-miR398a_mismatch probe
vvi|miR-63|nat|as|	caggggcgacctgagaacaca	vvi-miR398b_perfect match probe
vvi|miR-63|2mut|as|	caggggagacccgagaacaca	vvi-miR398b_mismatch probe
vvi|miR-64|nat|as|	cagggcaattctcctttggca	vvi-miR399a_perfect match probe
vvi|miR-64|2mut|as|	caggccaattcttctttggca	vvi-miR399a_mismatch probe
vvi|miR-65|nat|as|	cagggcaactctcctttggca	vvi-miR399b_perfect match probe
vvi|miR-65|2mut|as|	caggccaactcttctttggca	vvi-miR399b_mismatch probe
vvi|miR-66|nat|as|	acgagcaaatctcctttggca	vvi-miR399d_perfect match probe
vvi|miR-66|2mut|as|	acgagaaaatcttctttggca	vvi-miR399d_mismatch probe
vvi|miR-67|nat|as|	ccgggcaaatctcctttggca	vvi-miR399e_perfect match probe
vvi|miR-67|2mut|as|	ccggccaaatcttctttggca	vvi-miR399e_mismatch probe
vvi|miR-68|nat|as|	caggacaaatctccttcggca	vvi-miR399f_perfect match probe
vvi|miR-68|2mut|as|	caggaaaaatctccttaggca	vvi-miR399f_mismatch probe
vvi|miR-69|nat|as|	aggggcaaatctcctttggca	vvi-miR399g_perfect match probe
vvi|miR-69|2mut|as|	agggccaaatcttctttggca	vvi-miR399g_mismatch probe
vvi|miR-70|nat|as|	cagggcaactctcctttggcg	vvi-miR399i_perfect match probe
vvi|miR-70|2mut|as|	caggccaactcttctttggcg	vvi-miR399i_mismatch probe
vvi|miR-71|nat|as|	cgagtttgtgcgtgaatctaa	vvi-miR403a_perfect match probe
vvi|miR-71|2mut|as|	cgagtttgtgactgaatctaa	vvi-miR403a_mismatch probe
vvi|miR-72|nat|as|	gccagggaagaggcagtgcat	vvi-miR408_perfect match probe
vvi|miR-72|2mut|as|	gccagtgaagagccagtgcat	vvi-miR408_mismatch probe
vvi|miR-73|nat|as|	ttggaagcctttgagggagat	vvi-miR477a_perfect match probe
vvi|miR-73|2mut|as|	ttggaagtctttgagtgagat	vvi-miR477a_mismatch probe
vvi|miR-74|nat|as|	gatgagccgaaccaataccaca	vvi-miR479_perfect match probe
vvi|miR-74|2mut|as|	gatgagtcgaacaaataccaca	vvi-miR479_mismatch probe
vvi|miR-75|nat|as|	ggaatgggaggagtagg	vvi-miR482a_perfect match probe
vvi|miR-75|2mut|as|	ggaatgtgagaagtagg	vvi-miR482a_mismatch probe
vvi|miR-76|nat|as|	agcgtgctctctctcgttgtca	vvi-miR535a_perfect match probe
vvi|miR-76|2mut|as|	agcgtcctctctctccttgtca	vvi-miR535a_mismatch probe
vvi|miR-77|nat|as|	tggaatactcatttgagcaaga	vvi-miR828a_perfect match probe
vvi|miR-77|2mut|as|	tggaatactaatttgagaaaga	vvi-miR828a_mismatch probe

vvi|miR-78|nat|as|	tggaacactcatttgagcaaga	vvi-miR828b_perfect match probe
vvi|miR-78|2mut|as|	tggaaccctcatttgagaaaga	vvi-miR828b_mismatch probe
vvi|miR-79|nat|as|	tatcaattggtatcagagcta	vvi-miR845a_perfect match probe
vvi|miR-79|2mut|as|	tatcaattcgtatccgagcta	vvi-miR845a_mismatch probe
vvi|miR-80|nat|as|	catcaattggtatcagagcct	vvi-miR845c_perfect match probe
vvi|miR-80|2mut|as|	catcaattcgtatccgagcct	vvi-miR845c_mismatch probe
vvi|miR-81|nat|as|	catcaattggtatcagagcca	vvi-miR845d_perfect match probe
vvi|miR-81|2mut|as|	catcaattcgtatccgagcca	vvi-miR845d_mismatch probe
vvi|miR-82|nat|as|	ccctctcttctgtcaacata	vvi-miR156a|-5_perfect match probe
vvi|miR-82|2mut|as|	ccctcccttctctcaacata	vvi-miR156a|-5_mismatch probe
vvi|miR-83|nat|as|	gggttgtgctccctctcttc	vvi-miR156a|+5_perfect match probe
vvi|miR-83|2mut|as|	gggttgtcctctctctcttc	vvi-miR156a|+5_mismatch probe
vvi|miR-84|nat|as|	cactctcttctgtcagtttt	vvi-miR156b|-5_perfect match probe
vvi|miR-84|2mut|as|	cactcccttctctcagtttt	vvi-miR156b|-5_mismatch probe
vvi|miR-85|nat|as|	tctgtgtgctcactctcttc	vvi-miR156b|+5_perfect match probe
vvi|miR-85|2mut|as|	tctgtgtcctccctctcttc	vvi-miR156b|+5_mismatch probe
vvi|miR-86|nat|as|	cactctcttctgtcacctat	vvi-miR156c|-5_perfect match probe
vvi|miR-86|2mut|as|	cactcccttctgtcatctat	vvi-miR156c|-5_mismatch probe
vvi|miR-87|nat|as|	catgtgtgctcactctcttc	vvi-miR156c|+5_perfect match probe
vvi|miR-87|2mut|as|	catgtgtcctccctctcttc	vvi-miR156c|+5_mismatch probe
vvi|miR-88|nat|as|	cactctcttctgtcagtctc	vvi-miR156d|-5_perfect match probe
vvi|miR-88|2mut|as|	cactcccttctctcagtctc	vvi-miR156d|-5_mismatch probe
vvi|miR-89|nat|as|	tgcatgtgctcactctcttc	vvi-miR156d|+5_perfect match probe
vvi|miR-89|2mut|as|	tgcatgtcctccctctcttc	vvi-miR156d|+5_mismatch probe
vvi|miR-90|nat|as|	cactctcctctgtcacctac	vvi-miR156e|-5_perfect match probe
vvi|miR-90|2mut|as|	cactcttctctgtcatctac	vvi-miR156e|-5_mismatch probe
vvi|miR-91|nat|as|	catgagtgctcactctcctc	vvi-miR156e|+5_perfect match probe
vvi|miR-91|2mut|as|	catgagtcctccctctcctc	vvi-miR156e|+5_mismatch probe
vvi|miR-92|nat|as|	tctatcttctgtcaacagaa	vvi-miR156f|-5_perfect match probe
vvi|miR-92|2mut|as|	tctatcttccgtcaaaagaa	vvi-miR156f|-5_mismatch probe
vvi|miR-93|nat|as|	cagttgtgctctctatcttc	vvi-miR156f|+5_perfect match probe
vvi|miR-93|2mut|as|	cagttgtcctccctatcttc	vvi-miR156f|+5_mismatch probe
vvi|miR-94|nat|as|	tctatcttctgtcaacaaca	vvi-miR156g|-5_perfect match probe
vvi|miR-94|2mut|as|	tctatcttccgtcaaaaaca	vvi-miR156g|-5_mismatch probe

vvi|miR-95|nat|as|	catctgtgctctctatcttc	vvi-miR156g|+5_perfect match probe
vvi|miR-95|2mut|as|	catctgtcctccctatcttc	vvi-miR156g|+5_mismatch probe
vvi|miR-96|nat|as|	ctctctcttctgtcattgtg	vvi-miR156h|-5_perfect match probe
vvi|miR-96|2mut|as|	ctctcccttctctcattgtg	vvi-miR156h|-5_mismatch probe
vvi|miR-97|nat|as|	ccagcatgctctctctcttc	vvi-miR156h|+5_perfect match probe
vvi|miR-97|2mut|as|	ccagcatcctctctcccttc	vvi-miR156h|+5_mismatch probe
vvi|miR-98|nat|as|	ctctatcttctgtcaacaac	vvi-miR156i|-5_perfect match probe
vvi|miR-98|2mut|as|	ctctatcttttctcaacaac	vvi-miR156i|-5_mismatch probe
vvi|miR-99|nat|as|	catctgtgctctctatcttc	vvi-miR156i|+5_perfect match probe
vvi|miR-99|2mut|as|	catctgtcctccctatcttc	vvi-miR156i|+5_mismatch probe
vvi|miR-100|nat|as|	tcccttcactccaaggccag	vvi-miR159a|-5_perfect match probe
vvi|miR-100|2mut|as|	tcccttccctccaagcccag	vvi-miR159a|-5_mismatch probe
vvi|miR-101|nat|as|	gttatgagagctcccttcac	vvi-miR159a|+5_perfect match probe
vvi|miR-101|2mut|as|	gttatgagacctctcttcac	vvi-miR159a|+5_mismatch probe
vvi|miR-102|nat|as|	tcccttcactccaaggccag	vvi-miR159b|-5_perfect match probe
vvi|miR-102|2mut|as|	tcccttccctccaagcccag	vvi-miR159b|-5_mismatch probe
vvi|miR-103|nat|as|	ggttatgagagctcccttca	vvi-miR159b|+5_perfect match probe
vvi|miR-103|2mut|as|	ggttataagagctctcttca	vvi-miR159b|+5_mismatch probe
vvi|miR-104|nat|as|	tcccttcaatccaaacaagg	vvi-miR159c|-5_perfect match probe
vvi|miR-104|2mut|as|	tcccttaaatcaaaacaagg	vvi-miR159c|-5_mismatch probe
vvi|miR-105|nat|as|	aggtgtagagctcccttcaa	vvi-miR159c|+5_perfect match probe
vvi|miR-105|2mut|as|	aggtgtagacctctcttcaa	vvi-miR159c|+5_mismatch probe
vvi|miR-106|nat|as|	tcagggagccaggcagatca	vvi-miR160a|-5_perfect match probe
vvi|miR-106|2mut|as|	tcagggagtcagccagatca	vvi-miR160a|-5_mismatch probe
vvi|miR-107|nat|as|	ttagatggcattcagggagc	vvi-miR160a|+5_perfect match probe
vvi|miR-107|2mut|as|	ttagatgccattcagtgagc	vvi-miR160a|+5_mismatch probe
vvi|miR-108|nat|as|	tcagggagccaggcagaaca	vvi-miR160b|-5_perfect match probe
vvi|miR-108|2mut|as|	tcagggagtcagccagaaca	vvi-miR160b|-5_mismatch probe
vvi|miR-109|nat|as|	ttagatggcattcagggagc	vvi-miR160b|+5_perfect match probe
vvi|miR-109|2mut|as|	ttagatgccattcagtgagc	vvi-miR160b|+5_mismatch probe
vvi|miR-110|nat|as|	acagggagccaggcacaacc	vvi-miR160c|-5_perfect match probe
vvi|miR-110|2mut|as|	acagggagtcagccacaacc	vvi-miR160c|-5_mismatch probe
vvi|miR-111|nat|as|	acgtgtggcatacagggagc	vvi-miR160c|+5_perfect match probe
vvi|miR-111|2mut|as|	acgtgtgccatacagtgagc	vvi-miR160c|+5_mismatch probe

vvi|miR-112|nat|as|	acagggagccaggcatgtaa	vvi-miR160d|-5_perfect match probe
vvi|miR-112|2mut|as|	acagggagtcagccatgtaa	vvi-miR160d|-5_mismatch probe
vvi|miR-113|nat|as|	gcaaatggcatacagggagc	vvi-miR160d|+5_perfect match probe
vvi|miR-113|2mut|as|	gcaaatgccatacagtgagc	vvi-miR160d|+5_mismatch probe
vvi|miR-114|nat|as|	tcagggagccaggcagaaca	vvi-miR160e|-5_perfect match probe
vvi|miR-114|2mut|as|	tcagggagtcagccagaaca	vvi-miR160e|-5_mismatch probe
vvi|miR-115|nat|as|	ttagatggcattcagggagc	vvi-miR160e|+5_perfect match probe
vvi|miR-115|2mut|as|	ttagatgccattcagtgagc	vvi-miR160e|+5_mismatch probe
vvi|miR-116|nat|as|	acagggagccaggcatatat	vvi-miR160f|-5_perfect match probe
vvi|miR-116|2mut|as|	acagggagtcagccatatat	vvi-miR160f|-5_mismatch probe
vvi|miR-117|nat|as|	gcaaatggcatacagggagc	vvi-miR160f|+5_perfect match probe
vvi|miR-117|2mut|as|	gcaaatgccatacagtgagc	vvi-miR160f|+5_mismatch probe
vvi|miR-118|nat|as|	gcagaggtttatcgaccgat	vvi-miR162|-5_perfect match probe
vvi|miR-118|2mut|as|	gcagagctttatagaccgat	vvi-miR162|-5_mismatch probe
vvi|miR-119|nat|as|	gaacgctggatgcagaggtt	vvi-miR162|+5_perfect match probe
vvi|miR-119|2mut|as|	gaaccctggatgaagaggtt	vvi-miR162|+5_mismatch probe
vvi|miR-120|nat|as|	tgccctgcttctccaacaag	vvi-miR164a|-5_perfect match probe
vvi|miR-120|2mut|as|	tgccctccttctcaaacaag	vvi-miR164a|-5_mismatch probe
vvi|miR-121|nat|as|	aaatctgcacgtgccctgct	vvi-miR164a|+5_perfect match probe
vvi|miR-121|2mut|as|	aaatctgaacgtgtcctgct	vvi-miR164a|+5_mismatch probe
vvi|miR-122|nat|as|	tgccctgcttctccagcatg	vvi-miR164b|-5_perfect match probe
vvi|miR-122|2mut|as|	tgccctccttctccaccatg	vvi-miR164b|-5_mismatch probe
vvi|miR-123|nat|as|	gatccagcatgtgccctgct	vvi-miR164b|+5_perfect match probe
vvi|miR-123|2mut|as|	gatccagaatgtgtcctgct	vvi-miR164b|+5_mismatch probe
vvi|miR-124|nat|as|	tgccctgcttctccatcaag	vvi-miR164d|-5_perfect match probe
vvi|miR-124|2mut|as|	tgccctccttctcaatcaag	vvi-miR164d|-5_mismatch probe
vvi|miR-125|nat|as|	tgaactgcacgtgccctgct	vvi-miR164d|+5_perfect match probe
vvi|miR-125|2mut|as|	tgaactgaacgtgtcctgct	vvi-miR164d|+5_mismatch probe
vvi|miR-126|nat|as|	tgaagcctggtccgacgaca	vvi-miR166c|-5_perfect match probe
vvi|miR-126|2mut|as|	tgaagtctggtcagacgaca	vvi-miR166c|-5_mismatch probe
vvi|miR-127|nat|as|	aattgggggggaatgaagcct	vvi-miR166c|+5_perfect match probe
vvi|miR-127|2mut|as|	aattggggtgaataaagcct	vvi-miR166c|+5_mismatch probe
vvi|miR-128|nat|as|	tgaagcctggtccgagggca	vvi-miR166d|-5_perfect match probe
vvi|miR-128|2mut|as|	tgaagtctggtcagagggca	vvi-miR166d|-5_mismatch probe

vvi|miR-129|nat|as|	gattgaggggaatgaagcct	vvi-miR166d|+5_perfect match probe
vvi|miR-129|2mut|as|	gattgaggtgaataaagcct	vvi-miR166d|+5_mismatch probe
vvi|miR-130|nat|as|	tgaagcctggtccgacgaca	vvi-miR166e|-5_perfect match probe
vvi|miR-130|2mut|as|	tgaagtctggtcagacgaca	vvi-miR166e|-5_mismatch probe
vvi|miR-131|nat|as|	aattgggggaatgaagcct	vvi-miR166e|+5_perfect match probe
vvi|miR-131|2mut|as|	aattggggtgaataaagcct	vvi-miR166e|+5_mismatch probe
vvi|miR-132|nat|as|	tgaagcctggtccgaagctt	vvi-miR166f|-5_perfect match probe
vvi|miR-132|2mut|as|	tgaagtctggtcagaagctt	vvi-miR166f|-5_mismatch probe
vvi|miR-133|nat|as|	tgttgaggggaatgaagcct	vvi-miR166f|+5_perfect match probe
vvi|miR-133|2mut|as|	tgttgaggtgaataaagcct	vvi-miR166f|+5_mismatch probe
vvi|miR-134|nat|as|	tgaagcctggtccgaaatca	vvi-miR166g|-5_perfect match probe
vvi|miR-134|2mut|as|	tgaagtctggtcagaaatca	vvi-miR166g|-5_mismatch probe
vvi|miR-135|nat|as|	ggttgaggggaatgaagcct	vvi-miR166g|+5_perfect match probe
vvi|miR-135|2mut|as|	ggttgaggtgaataaagcct	vvi-miR166g|+5_mismatch probe
vvi|miR-136|nat|as|	tgaagcctggtccgagatca	vvi-miR166h|-5_perfect match probe
vvi|miR-136|2mut|as|	tgaagtctggtcagagatca	vvi-miR166h|-5_mismatch probe
vvi|miR-137|nat|as|	agttgggggaatgaagcct	vvi-miR166h|+5_perfect match probe
vvi|miR-137|2mut|as|	agttggggtgaataaagcct	vvi-miR166h|+5_mismatch probe
vvi|miR-138|nat|as|	tgaagcctggtccgagatca	vvi-miR166a|-5_perfect match probe
vvi|miR-138|2mut|as|	tgaagtctggtcagagatca	vvi-miR166a|-5_mismatch probe
vvi|miR-139|nat|as|	ggtgtcaggaatgaagcctg	vvi-miR166a|+5_perfect match probe
vvi|miR-139|2mut|as|	ggtgtcagaaatgaaccctg	vvi-miR166a|+5_mismatch probe
vvi|miR-140|nat|as|	tgaagcctggtccgacacct	vvi-miR166b|-5_perfect match probe
vvi|miR-140|2mut|as|	tgaagtctggtcagacacct	vvi-miR166b|-5_mismatch probe
vvi|miR-141|nat|as|	tttgagaggaatgaagcctg	vvi-miR166b|+5_perfect match probe
vvi|miR-141|2mut|as|	tttgagagaaatgaaccctg	vvi-miR166b|+5_mismatch probe
vvi|miR-142|nat|as|	atgctggcagcttcaacctc	vvi-miR167a|-5_perfect match probe
vvi|miR-142|2mut|as|	atgctgccagtttcaacctc	vvi-miR167a|-5_mismatch probe
vvi|miR-143|nat|as|	ttcaccagatcatgctggca	vvi-miR167a|+5_perfect match probe
vvi|miR-143|2mut|as|	ttcacaagatcatcctggca	vvi-miR167a|+5_mismatch probe
vvi|miR-144|nat|as|	atgctggcagcttcaactgc	vvi-miR167b|-5_perfect match probe
vvi|miR-144|2mut|as|	atgctgccagtttcaactgc	vvi-miR167b|-5_mismatch probe
vvi|miR-145|nat|as|	aagcttagatcatgctggca	vvi-miR167b|+5_perfect match probe
vvi|miR-145|2mut|as|	aagcttacatcatcctggca	vvi-miR167b|+5_mismatch probe

vvi|miR-146|nat|as|	atgctggcagcttcaactgc	vvi-miR167c|-5_perfect match probe
vvi|miR-146|2mut|as|	atgctgccagtttcaactgc	vvi-miR167c|-5_mismatch probe
vvi|miR-147|nat|as|	agttgagatcatgctggcag	vvi-miR167c|+5_perfect match probe
vvi|miR-147|2mut|as|	agttgagataatcctggcag	vvi-miR167c|+5_mismatch probe
vvi|miR-148|nat|as|	atgctggcagcttcacttat	vvi-miR167d|-5_perfect match probe
vvi|miR-148|2mut|as|	atgctgccagtttcacttat	vvi-miR167d|-5_mismatch probe
vvi|miR-149|nat|as|	aaagctagatcatgctggca	vvi-miR167d|+5_perfect match probe
vvi|miR-149|2mut|as|	aaagttagatcatcctggca	vvi-miR167d|+5_mismatch probe
vvi|miR-150|nat|as|	atgctggcagcttcaactga	vvi-miR167e|-5_perfect match probe
vvi|miR-150|2mut|as|	atgctgccagtttcaactga	vvi-miR167e|-5_mismatch probe
vvi|miR-151|nat|as|	aagtttagatcatgctggca	vvi-miR167e|+5_perfect match probe
vvi|miR-151|2mut|as|	aagtttacatcatcctggca	vvi-miR167e|+5_mismatch probe
vvi|miR-152|nat|as|	acctgcaccaagcgaattag	vvi-miR168|-5_perfect match probe
vvi|miR-152|2mut|as|	acctgaaccaagagaattag	vvi-miR168|-5_mismatch probe
vvi|miR-153|nat|as|	gtcggttcccgacctgcacc	vvi-miR168|+5_perfect match probe
vvi|miR-153|2mut|as|	gtcggttccagacctccacc	vvi-miR168|+5_mismatch probe
vvi|miR-154|nat|as|	agtcatccttggctgcattc	vvi-miR169a|-5_perfect match probe
vvi|miR-154|2mut|as|	agtcattcttgcctgcattc	vvi-miR169a|-5_mismatch probe
vvi|miR-155|nat|as|	aaattccggcaagtcatcct	vvi-miR169a|+5_perfect match probe
vvi|miR-155|2mut|as|	aaattccgccaagccatcct	vvi-miR169a|+5_mismatch probe
vvi|miR-156|nat|as|	agtcatccttcgctaccaaa	vvi-miR169y|-5_perfect match probe
vvi|miR-156|2mut|as|	agtcattcttccctaccaaa	vvi-miR169y|-5_mismatch probe
vvi|miR-157|nat|as|	ggctgtaggcaagtcatcct	vvi-miR169y|+5_perfect match probe
vvi|miR-157|2mut|as|	ggctgtagccaagccatcct	vvi-miR169y|+5_mismatch probe
vvi|miR-158|nat|as|	gccatccttggctcaattcg	vvi-miR169b|-5_perfect match probe
vvi|miR-158|2mut|as|	gccatccttccctcaattcg	vvi-miR169b|-5_mismatch probe
vvi|miR-159|nat|as|	gatgacggcaagccatcctt	vvi-miR169b|+5_perfect match probe
vvi|miR-159|2mut|as|	gatgacgccaagtcatcctt	vvi-miR169b|+5_mismatch probe
vvi|miR-160|nat|as|	agtcatccttggctgcattc	vvi-miR169c|-5_perfect match probe
vvi|miR-160|2mut|as|	agtcattcttgcctgcattc	vvi-miR169c|-5_mismatch probe
vvi|miR-161|nat|as|	catctccggcaagtcatcct	vvi-miR169c|+5_perfect match probe
vvi|miR-161|2mut|as|	catctccgccaagccatcct	vvi-miR169c|+5_mismatch probe
vvi|miR-162|nat|as|	aatcattcttggctgcattc	vvi-miR169d|-5_perfect match probe
vvi|miR-162|2mut|as|	aatcatttttgcctgcattc	vvi-miR169d|-5_mismatch probe

vvi|miR-163|nat|as|	aaattccggcaaatcattct	vvi-miR169d|+5_perfect match probe
vvi|miR-163|2mut|as|	aaattccgccaaataattct	vvi-miR169d|+5_mismatch probe
vvi|miR-164|nat|as|	agtcatccttggctatcaga	vvi-miR169e|-5_perfect match probe
vvi|miR-164|2mut|as|	agtcattcttgcctatcaga	vvi-miR169e|-5_mismatch probe
vvi|miR-165|nat|as|	tagggcaggcaagtcatcct	vvi-miR169e|+5_perfect match probe
vvi|miR-165|2mut|as|	tagggcagccaagccatcct	vvi-miR169e|+5_mismatch probe
vvi|miR-166|nat|as|	agtcatccttggctgcacca	vvi-miR169f|-5_perfect match probe
vvi|miR-166|2mut|as|	agtcattcttgcctgcacca	vvi-miR169f|-5_mismatch probe
vvi|miR-167|nat|as|	tattttcggcaagtcatcct	vvi-miR169f|+5_perfect match probe
vvi|miR-167|2mut|as|	tattttcgccaagccatcct	vvi-miR169f|+5_mismatch probe
vvi|miR-168|nat|as|	agtcatccttggctgcacca	vvi-miR169g|-5_perfect match probe
vvi|miR-168|2mut|as|	agtcattcttgcctgcacca	vvi-miR169g|-5_mismatch probe
vvi|miR-169|nat|as|	gagtcggcaagtcatccttg	vvi-miR169g|+5_perfect match probe
vvi|miR-169|2mut|as|	gagtcgccaagtcattcttg	vvi-miR169g|+5_mismatch probe
vvi|miR-170|nat|as|	gccatccttggctcaattcc	vvi-miR169h|-5_perfect match probe
vvi|miR-170|2mut|as|	gccatccttccctcaattcc	vvi-miR169h|-5_mismatch probe
vvi|miR-171|nat|as|	aaggacggcaagccatcctt	vvi-miR169h|+5_perfect match probe
vvi|miR-171|2mut|as|	aaggacgccaagtcatcctt	vvi-miR169h|+5_mismatch probe
vvi|miR-172|nat|as|	agtcatccttggctcaattc	vvi-miR169i|-5_perfect match probe
vvi|miR-172|2mut|as|	agtcattcttgcctcaattc	vvi-miR169i|-5_mismatch probe
vvi|miR-173|nat|as|	aaatgacggccagtcatcct	vvi-miR169i|+5_perfect match probe
vvi|miR-173|2mut|as|	aaatgacggtaagtcatcct	vvi-miR169i|+5_mismatch probe
vvi|miR-174|nat|as|	agtcatccttggctgcactc	vvi-miR169j|-5_perfect match probe
vvi|miR-174|2mut|as|	agtcattcttgcctgcactc	vvi-miR169j|-5_mismatch probe
vvi|miR-175|nat|as|	gaattccggcaagtcatcct	vvi-miR169j|+5_perfect match probe
vvi|miR-175|2mut|as|	gaattccgccaagccatcct	vvi-miR169j|+5_mismatch probe
vvi|miR-176|nat|as|	agtcatccttggctgcactc	vvi-miR169k|-5_perfect match probe
vvi|miR-176|2mut|as|	agtcattcttgcctgcactc	vvi-miR169k|-5_mismatch probe
vvi|miR-177|nat|as|	gaattccggcaagtcatcct	vvi-miR169k|+5_perfect match probe
vvi|miR-177|2mut|as|	gaattccgccaagccatcct	vvi-miR169k|+5_mismatch probe
vvi|miR-178|nat|as|	agtcatccttggctcaattc	vvi-miR169l|-5_perfect match probe
vvi|miR-178|2mut|as|	agtcattcttgcctcaattc	vvi-miR169l|-5_mismatch probe
vvi|miR-179|nat|as|	aaaggacggcaagtcatcct	vvi-miR169l|+5_perfect match probe
vvi|miR-179|2mut|as|	aaaggacgccaagccatcct	vvi-miR169l|+5_mismatch probe

<table>
<tr><td>vvi|miR-180|nat|as|</td><td>agtcatccttggctcacttc</td><td>vvi-miR169m|-5_perfect match probe</td></tr>
<tr><td>vvi|miR-180|2mut|as|</td><td>agtcattcttgcctcacttc</td><td>vvi-miR169m|-5_mismatch probe</td></tr>
<tr><td></td><td></td><td>vvi-miR169m|+5_perfect match probe</td></tr>
<tr><td>vvi|miR-181|nat|as|</td><td>agctgccggcaagtcatcct</td><td></td></tr>
<tr><td>vvi|miR-181|2mut|as|</td><td>agctgccgccaagccatcct</td><td>vvi-miR169m|+5_mismatch probe</td></tr>
<tr><td>vvi|miR-182|nat|as|</td><td>agtcatccttggctctattc</td><td>vvi-miR169n|-5_perfect match probe</td></tr>
<tr><td>vvi|miR-182|2mut|as|</td><td>agtcattcttgcctctattc</td><td>vvi-miR169n|-5_mismatch probe</td></tr>
<tr><td>vvi|miR-183|nat|as|</td><td>agttgccggcaagtcatcct</td><td>vvi-miR169n|+5_perfect match probe</td></tr>
<tr><td>vvi|miR-183|2mut|as|</td><td>agttgccgccaagccatcct</td><td>vvi-miR169n|+5_mismatch probe</td></tr>
<tr><td>vvi|miR-184|nat|as|</td><td>agtcatccttggctcaaatc</td><td>vvi-miR169o|-5_perfect match probe</td></tr>
<tr><td>vvi|miR-184|2mut|as|</td><td>agtcattcttgcctcaaatc</td><td>vvi-miR169o|-5_mismatch probe</td></tr>
<tr><td>vvi|miR-185|nat|as|</td><td>tgatggcggcaagtcatcct</td><td>vvi-miR169o|+5_perfect match probe</td></tr>
<tr><td>vvi|miR-185|2mut|as|</td><td>tgatggaggcaagccatcct</td><td>vvi-miR169o|+5_mismatch probe</td></tr>
<tr><td>vvi|miR-186|nat|as|</td><td>agtcatccttggctcaattc</td><td>vvi-miR169p|-5_perfect match probe</td></tr>
<tr><td>vvi|miR-186|2mut|as|</td><td>agtcattcttgcctcaattc</td><td>vvi-miR169p|-5_mismatch probe</td></tr>
<tr><td>vvi|miR-187|nat|as|</td><td>agctgccggcaagtcatcct</td><td>vvi-miR169p|+5_perfect match probe</td></tr>
<tr><td>vvi|miR-187|2mut|as|</td><td>agctgccgccaagccatcct</td><td>vvi-miR169p|+5_mismatch probe</td></tr>
<tr><td>vvi|miR-188|nat|as|</td><td>agtcatccttggctctattc</td><td>vvi-miR169q|-5_perfect match probe</td></tr>
<tr><td>vvi|miR-188|2mut|as|</td><td>agtcattcttgcctctattc</td><td>vvi-miR169q|-5_mismatch probe</td></tr>
<tr><td>vvi|miR-189|nat|as|</td><td>aaatgccggcaagtcatcct</td><td>vvi-miR169q|+5_perfect match probe</td></tr>
<tr><td>vvi|miR-189|2mut|as|</td><td>aaatgccgccaagccatcct</td><td>vvi-miR169q|+5_mismatch probe</td></tr>
<tr><td>vvi|miR-190|nat|as|</td><td>gtcatccttgactcaattcc</td><td>vvi-miR169r|-5_perfect match probe</td></tr>
<tr><td>vvi|miR-190|2mut|as|</td><td>gtcattcttgcctcaattcc</td><td>vvi-miR169r|-5_mismatch probe</td></tr>
<tr><td>vvi|miR-191|nat|as|</td><td>tatatcggcaagtcatcctt</td><td>vvi-miR169r|+5_perfect match probe</td></tr>
<tr><td>vvi|miR-191|2mut|as|</td><td>tatatcgccaagccatcctt</td><td>vvi-miR169r|+5_mismatch probe</td></tr>
<tr><td>vvi|miR-192|nat|as|</td><td>agtcatccttggctgcaatc</td><td>vvi-miR169s|-5_perfect match probe</td></tr>
<tr><td>vvi|miR-192|2mut|as|</td><td>agtcattcttgcctgcaatc</td><td>vvi-miR169s|-5_mismatch probe</td></tr>
<tr><td>vvi|miR-193|nat|as|</td><td>aagtgccggcaagtcatcct</td><td>vvi-miR169s|+5_perfect match probe</td></tr>
<tr><td>vvi|miR-193|2mut|as|</td><td>aagtgccgccaagccatcct</td><td>vvi-miR169s|+5_mismatch probe</td></tr>
<tr><td>vvi|miR-194|nat|as|</td><td>gtcatccttgactcgattcc</td><td>vvi-miR169t|-5_perfect match probe</td></tr>
<tr><td>vvi|miR-194|2mut|as|</td><td>gtcattcttgactagattcc</td><td>vvi-miR169t|-5_mismatch probe</td></tr>
<tr><td>vvi|miR-195|nat|as|</td><td>tatatcggcaagtcatcctt</td><td>vvi-miR169t|+5_perfect match probe</td></tr>
<tr><td>vvi|miR-195|2mut|as|</td><td>tatatcgccaagccatcctt</td><td>vvi-miR169t|+5_mismatch probe</td></tr>
<tr><td>vvi|miR-196|nat|as|</td><td>gtcatccttgactcaattcc</td><td>vvi-miR169u|-5_perfect match probe</td></tr>
</table>

vvi|miR-196|2mut|as|	gtcattcttgcctcaattcc	vvi-miR169u|-5_mismatch probe
vvi|miR-197|nat|as|	tataacggcaagtcatcctt	vvi-miR169u|+5_perfect match probe
vvi|miR-197|2mut|as|	tataacgccaagccatcctt	vvi-miR169u|+5_mismatch probe
vvi|miR-198|nat|as|	attcatccttggctttgttc	vvi-miR169v|-5_perfect match probe
vvi|miR-198|2mut|as|	attcattcttgcctttgttc	vvi-miR169v|-5_mismatch probe
vvi|miR-199|nat|as|	catcgccggcaattcatcct	vvi-miR169v|+5_perfect match probe
vvi|miR-199|2mut|as|	catccccggcaattaatcct	vvi-miR169v|+5_mismatch probe
vvi|miR-200|nat|as|	agtcatccttggctgcacca	vvi-miR169w|-5_perfect match probe
vvi|miR-200|2mut|as|	agtcattcttgcctgcacca	vvi-miR169w|-5_mismatch probe
		vvi-miR169w|+5_perfect match
vvi|miR-201|nat|as|	agttgccggcaagtcatcct	probe
vvi|miR-201|2mut|as|	agttgccgccaagccatcct	vvi-miR169w|+5_mismatch probe
vvi|miR-202|nat|as|	agtcatccttggctaccaga	vvi-miR169x|-5_perfect match probe
vvi|miR-202|2mut|as|	agtcattcttgcctaccaga	vvi-miR169x|-5_mismatch probe
vvi|miR-203|nat|as|	ggatttaggcaagtcatcct	vvi-miR169x|+5_perfect match probe
vvi|miR-203|2mut|as|	ggatttagccaagccatcct	vvi-miR169x|+5_mismatch probe
vvi|miR-204|nat|as|	gttgggacggctcaatcaaa	vvi-miR171a|-5_perfect match probe
vvi|miR-204|2mut|as|	gttgggacgcttcaatcaaa	vvi-miR171a|-5_mismatch probe
vvi|miR-205|nat|as|	ggaagcgtgatgttgggacg	vvi-miR171a|+5_perfect match probe
vvi|miR-205|2mut|as|	ggaagagtgatgttgtgacg	vvi-miR171a|+5_mismatch probe
vvi|miR-206|nat|as|	gacgcggctcaatcaaatca	vvi-miR171b|-5_perfect match probe
vvi|miR-206|2mut|as|	gacgaggctcaataaaatca	vvi-miR171b|-5_mismatch probe
vvi|miR-207|nat|as|	aaggagatattgacgcggct	vvi-miR171b|+5_perfect match probe
vvi|miR-207|2mut|as|	aaggcgatattgacgaggct	vvi-miR171b|+5_mismatch probe
vvi|miR-208|nat|as|	attggcacggctcaatcaaa	vvi-miR171c|-5_perfect match probe
vvi|miR-208|2mut|as|	attggcacgcttcaatcaaa	vvi-miR171c|-5_mismatch probe
vvi|miR-209|nat|as|	tgacacgtgatattggcacg	vvi-miR171c|+5_perfect match probe
vvi|miR-209|2mut|as|	tgacacctgatattgccacg	vvi-miR171c|+5_mismatch probe
vvi|miR-210|nat|as|	attggcacggctcaatcaaa	vvi-miR171d|-5_perfect match probe
vvi|miR-210|2mut|as|	attggcacgcttcaatcaaa	vvi-miR171d|-5_mismatch probe
vvi|miR-211|nat|as|	taggacgtgatattggcacg	vvi-miR171d|+5_perfect match probe
vvi|miR-211|2mut|as|	taggacctgatattgccacg	vvi-miR171d|+5_mismatch probe
vvi|miR-212|nat|as|	ggcgcggctcaatcagagat	vvi-miR171e|-5_perfect match probe
vvi|miR-212|2mut|as|	ggcgaggctcaatccgagat	vvi-miR171e|-5_mismatch probe

vvi|miR-213|nat|as|	aaagtgatattggcgcggct	vvi-miR171e|+5_perfect match probe
vvi|miR-213|2mut|as|	aaagcgatattggcgaggct	vvi-miR171e|+5_mismatch probe
vvi|miR-214|nat|as|	attggcgcggctcaatctga	vvi-miR171f|-5_perfect match probe
vvi|miR-214|2mut|as|	attggcgaggttcaatctga	vvi-miR171f|-5_mismatch probe
vvi|miR-215|nat|as|	agaaaagtgatattggcgcg	vvi-miR171f|+5_perfect match probe
vvi|miR-215|2mut|as|	agaaaagcgatattgccgcg	vvi-miR171f|+5_mismatch probe
vvi|miR-216|nat|as|	attggttcggctcaaggagt	vvi-miR171g|-5_perfect match probe
vvi|miR-216|2mut|as|	attggttcgcttcaaggagt	vvi-miR171g|-5_mismatch probe
vvi|miR-217|nat|as|	gctcgggtgatattggttcg	vvi-miR171g|+5_perfect match probe
vvi|miR-217|2mut|as|	gctcaggtgatattcgttcg	vvi-miR171g|+5_mismatch probe
vvi|miR-218|nat|as|	ggcgcggctcaaccaaacat	vvi-miR171h|-5_perfect match probe
vvi|miR-218|2mut|as|	ggcgaggctcaacaaaacat	vvi-miR171h|-5_mismatch probe
vvi|miR-219|nat|as|	gacgggatattggcgcggct	vvi-miR171h|+5_perfect match probe
vvi|miR-219|2mut|as|	gacgtgatattggcgaggct	vvi-miR171h|+5_mismatch probe
vvi|miR-220|nat|as|	attggcacggctcaatcaga	vvi-miR171i|-5_perfect match probe
vvi|miR-220|2mut|as|	attggcacgcttcaatcaga	vvi-miR171i|-5_mismatch probe
vvi|miR-221|nat|as|	aacatgatgatattggcacg	vvi-miR171i|+5_perfect match probe
vvi|miR-221|2mut|as|	aacatgataatattgccacg	vvi-miR171i|+5_mismatch probe
vvi|miR-222|nat|as|	catcatcaagattcacatgc	vvi-miR172a|-5_perfect match probe
vvi|miR-222|2mut|as|	catcataaagattcccatgc	vvi-miR172a|-5_mismatch probe
vvi|miR-223|nat|as|	tgcgcatgtagcatcatcaa	vvi-miR172a|+5_perfect match probe
vvi|miR-223|2mut|as|	tgcgaatgtagaatcatcaa	vvi-miR172a|+5_mismatch probe
vvi|miR-224|nat|as|	catcatcaagattcacatgc	vvi-miR172b|-5_perfect match probe
vvi|miR-224|2mut|as|	catcataaagattcccatgc	vvi-miR172b|-5_mismatch probe
vvi|miR-225|nat|as|	tgcaggtgtagcatcatcaa	vvi-miR172b|+5_perfect match probe
vvi|miR-225|2mut|as|	tgcagctgtagaatcatcaa	vvi-miR172b|+5_mismatch probe
vvi|miR-226|nat|as|	catcatcaagattcccacat	vvi-miR172c|-5_perfect match probe
vvi|miR-226|2mut|as|	catcataaagattctcacat	vvi-miR172c|-5_mismatch probe
vvi|miR-227|nat|as|	tgccgctgcagcatcatcaa	vvi-miR172c|+5_perfect match probe
vvi|miR-227|2mut|as|	tgcccctgcagaatcatcaa	vvi-miR172c|+5_mismatch probe
vvi|miR-228|nat|as|	catcatcaagattctcatct	vvi-miR172d|-5_perfect match probe
vvi|miR-228|2mut|as|	catcataaagattctaatct	vvi-miR172d|-5_mismatch probe
vvi|miR-229|nat|as|	tgcgaatgcagcatcatcaa	vvi-miR172d|+5_perfect match probe
vvi|miR-229|2mut|as|	tgcgaatgaagaatcatcaa	vvi-miR172d|+5_mismatch probe

vvi|miR-230|nat|as|	tcccttcagtccaagacgtg	vvi-miR319b|-5_perfect match probe
vvi|miR-230|2mut|as|	tcccttcagccaaagacgtg	vvi-miR319b|-5_mismatch probe
vvi|miR-231|nat|as|	tacaagggagctcccttcag	vvi-miR319b|+5_perfect match probe
vvi|miR-231|2mut|as|	tacaagtgagctctcttcag	vvi-miR319b|+5_mismatch probe
vvi|miR-232|nat|as|	tcccttcagtccaagcacag	vvi-miR319c|-5_perfect match probe
vvi|miR-232|2mut|as|	tcccttcagcccaagaacag	vvi-miR319c|-5_mismatch probe
vvi|miR-233|nat|as|	gtgaagggagctcccttcag	vvi-miR319c|+5_perfect match probe
vvi|miR-233|2mut|as|	gtgaagtgagctctcttcag	vvi-miR319c|+5_mismatch probe
vvi|miR-234|nat|as|	tcccttcagtccaaaacaag	vvi-miR319e|-5_perfect match probe
vvi|miR-234|2mut|as|	tcccttcagccaaaaacaag	vvi-miR319e|-5_mismatch probe
vvi|miR-235|nat|as|	gaactaggagctcccttcag	vvi-miR319e|+5_perfect match probe
vvi|miR-235|2mut|as|	gaactaggacctctcttcag	vvi-miR319e|+5_mismatch probe
vvi|miR-236|nat|as|	tcccttcagtccaagcacag	vvi-miR319f|-5_perfect match probe
vvi|miR-236|2mut|as|	tcccttcagcccaagaacag	vvi-miR319f|-5_mismatch probe
vvi|miR-237|nat|as|	tagaagggagctcccttcag	vvi-miR319f|+5_perfect match probe
vvi|miR-237|2mut|as|	tagaagtgagctctcttcag	vvi-miR319f|+5_mismatch probe
vvi|miR-238|nat|as|	tcccttcagtccaatgagtg	vvi-miR319g|-5_perfect match probe
vvi|miR-238|2mut|as|	tcccttcagccaaatgagtg	vvi-miR319g|-5_mismatch probe
vvi|miR-239|nat|as|	gccctgggagctcccttcag	vvi-miR319g|+5_perfect match probe
vvi|miR-239|2mut|as|	gccctgtgagctctcttcag	vvi-miR319g|+5_mismatch probe
vvi|miR-240|nat|as|	atccctcctgagcttaacag	vvi-miR390|-5_perfect match probe
vvi|miR-240|2mut|as|	atcccttctgagtttaacag	vvi-miR390|-5_mismatch probe
vvi|miR-241|nat|as|	ctcatggcgctatccctcct	vvi-miR390|+5_perfect match probe
vvi|miR-241|2mut|as|	ctcatggccctatctctcct	vvi-miR390|+5_mismatch probe
vvi|miR-242|nat|as|	tgcgatccctttggaactgt	vvi-miR393a|-5_perfect match probe
vvi|miR-242|2mut|as|	tgcgatctctttcgaactgt	vvi-miR393a|-5_mismatch probe
vvi|miR-243|nat|as|	ccatgggatcaatgcgatcc	vvi-miR393a|+5_perfect match probe
vvi|miR-243|2mut|as|	ccatgtgatcaatgagatcc	vvi-miR393a|+5_mismatch probe
vvi|miR-244|nat|as|	tgcgatccctttggatgcct	vvi-miR393b|-5_perfect match probe
vvi|miR-244|2mut|as|	tgcgatctctttcgatgcct	vvi-miR393b|-5_mismatch probe
vvi|miR-245|nat|as|	gtttgggatcaatgcgatcc	vvi-miR393b|+5_perfect match probe
vvi|miR-245|2mut|as|	gtttgtgatcaatgagatcc	vvi-miR393b|+5_mismatch probe
vvi|miR-246|nat|as|	tggacagaatgccaaaatgg	vvi-miR394a|-5_perfect match probe
vvi|miR-246|2mut|as|	tggaaagaatgtcaaaatgg	vvi-miR394a|-5_mismatch probe

vvi|miR-247|nat|as|	tgcatggaggtggacagaat	vvi-miR394a|+5_perfect match probe
vvi|miR-247|2mut|as|	tgcatcgaggtcgacagaat	vvi-miR394a|+5_mismatch probe
vvi|miR-248|nat|as|	tggacagaatgccaataaac	vvi-miR394b|-5_perfect match probe
vvi|miR-248|2mut|as|	tggaaagaatgtcaataaac	vvi-miR394b|-5_mismatch probe
vvi|miR-249|nat|as|	agatgggaggtggacagaat	vvi-miR394b|+5_perfect match probe
vvi|miR-249|2mut|as|	agatgtgaggtcgacagaat	vvi-miR394b|+5_mismatch probe
vvi|miR-250|nat|as|	tggacagaatgccaaaatgg	vvi-miR394c|-5_perfect match probe
vvi|miR-250|2mut|as|	tggaaagaatgtcaaaatgg	vvi-miR394c|-5_mismatch probe
vvi|miR-251|nat|as|	tatatggaggtggacagaat	vvi-miR394c|+5_perfect match probe
vvi|miR-251|2mut|as|	tatatcgaggtcgacagaat	vvi-miR394c|+5_mismatch probe
vvi|miR-252|nat|as|	ccccaaacacttcagtagga	vvi-miR395a|-5_perfect match probe
vvi|miR-252|2mut|as|	ccccaaacccttccgtagga	vvi-miR395a|-5_mismatch probe
vvi|miR-253|nat|as|	accaggagttcccccaaaca	vvi-miR395a|+5_perfect match probe
vvi|miR-253|2mut|as|	accagaagttccctcaaaca	vvi-miR395a|+5_mismatch probe
vvi|miR-254|nat|as|	ccccaaacacttcagtacgt	vvi-miR395b|-5_perfect match probe
vvi|miR-254|2mut|as|	ccccaaacccttccgtacgt	vvi-miR395b|-5_mismatch probe
vvi|miR-255|nat|as|	accaggagttcccccaaaca	vvi-miR395b|+5_perfect match probe
vvi|miR-255|2mut|as|	accagaagttccctcaaaca	vvi-miR395b|+5_mismatch probe
vvi|miR-256|nat|as|	ccccaaacacttcagtagga	vvi-miR395c|-5_perfect match probe
vvi|miR-256|2mut|as|	ccccaaacccttccgtagga	vvi-miR395c|-5_mismatch probe
vvi|miR-257|nat|as|	accaggagttcccccaaaca	vvi-miR395c|+5_perfect match probe
vvi|miR-257|2mut|as|	accagaagttccctcaaaca	vvi-miR395c|+5_mismatch probe
vvi|miR-258|nat|as|	ccccaaacacttcagtagga	vvi-miR395d|-5_perfect match probe
vvi|miR-258|2mut|as|	ccccaaacccttccgtagga	vvi-miR395d|-5_mismatch probe
vvi|miR-259|nat|as|	accaggagttcccccaaaca	vvi-miR395d|+5_perfect match probe
vvi|miR-259|2mut|as|	accagaagttccctcaaaca	vvi-miR395d|+5_mismatch probe
vvi|miR-260|nat|as|	ccccaaacacttcagtagga	vvi-miR395e|-5_perfect match probe
vvi|miR-260|2mut|as|	ccccaaacccttccgtagga	vvi-miR395e|-5_mismatch probe
vvi|miR-261|nat|as|	accaggagttcccccaaaca	vvi-miR395e|+5_perfect match probe
vvi|miR-261|2mut|as|	accagaagttccctcaaaca	vvi-miR395e|+5_mismatch probe
vvi|miR-262|nat|as|	ccccaaacacttcagtggga	vvi-miR395f|-5_perfect match probe
vvi|miR-262|2mut|as|	ccccaaacccttcagcggga	vvi-miR395f|-5_mismatch probe
vvi|miR-263|nat|as|	accaggagttcccccaaaca	vvi-miR395f|+5_perfect match probe
vvi|miR-263|2mut|as|	accagaagttccctcaaaca	vvi-miR395f|+5_mismatch probe

<table>
<tr><td>vvi|miR-264|nat|as|</td><td>ccccaaacacttcagtagga</td><td>vvi-miR395g|-5_perfect match probe</td></tr>
<tr><td>vvi|miR-264|2mut|as|</td><td>ccccaaacccttccgtagga</td><td>vvi-miR395g|-5_mismatch probe</td></tr>
<tr><td>vvi|miR-265|nat|as|</td><td>accgggagttcccccaaaca</td><td>vvi-miR395g|+5_perfect match probe</td></tr>
<tr><td>vvi|miR-265|2mut|as|</td><td>accgtgagttccctcaaaca</td><td>vvi-miR395g|+5_mismatch probe</td></tr>
<tr><td>vvi|miR-266|nat|as|</td><td>ccccaaacacttcagtagga</td><td>vvi-miR395h|-5_perfect match probe</td></tr>
<tr><td>vvi|miR-266|2mut|as|</td><td>ccccaaacccttccgtagga</td><td>vvi-miR395h|-5_mismatch probe</td></tr>
<tr><td>vvi|miR-267|nat|as|</td><td>accaggagttcccccaaaca</td><td>vvi-miR395h|+5_perfect match probe</td></tr>
<tr><td>vvi|miR-267|2mut|as|</td><td>accagaagttccctcaaaca</td><td>vvi-miR395h|+5_mismatch probe</td></tr>
<tr><td>vvi|miR-268|nat|as|</td><td>ccccaaacacttcagtagga</td><td>vvi-miR395i|-5_perfect match probe</td></tr>
<tr><td>vvi|miR-268|2mut|as|</td><td>ccccaaacccttccgtagga</td><td>vvi-miR395i|-5_mismatch probe</td></tr>
<tr><td>vvi|miR-269|nat|as|</td><td>accaggagttcccccaaaca</td><td>vvi-miR395i|+5_perfect match probe</td></tr>
<tr><td>vvi|miR-269|2mut|as|</td><td>accagaagttccctcaaaca</td><td>vvi-miR395i|+5_mismatch probe</td></tr>
<tr><td>vvi|miR-270|nat|as|</td><td>ccccaaacacttcagtagga</td><td>vvi-miR395j|-5_perfect match probe</td></tr>
<tr><td>vvi|miR-270|2mut|as|</td><td>ccccaaacccttccgtagga</td><td>vvi-miR395j|-5_mismatch probe</td></tr>
<tr><td>vvi|miR-271|nat|as|</td><td>accaggagttcccccaaaca</td><td>vvi-miR395j|+5_perfect match probe</td></tr>
<tr><td>vvi|miR-271|2mut|as|</td><td>accagaagttccctcaaaca</td><td>vvi-miR395j|+5_mismatch probe</td></tr>
<tr><td>vvi|miR-272|nat|as|</td><td>ccccaaacacttcagtagga</td><td>vvi-miR395k|-5_perfect match probe</td></tr>
<tr><td>vvi|miR-272|2mut|as|</td><td>ccccaaacccttccgtagga</td><td>vvi-miR395k|-5_mismatch probe</td></tr>
<tr><td>vvi|miR-273|nat|as|</td><td>accaggagttcccccaaaca</td><td>vvi-miR395k|+5_perfect match probe</td></tr>
<tr><td>vvi|miR-273|2mut|as|</td><td>accagaagttccctcaaaca</td><td>vvi-miR395k|+5_mismatch probe</td></tr>
<tr><td>vvi|miR-274|nat|as|</td><td>ccccaaacacttcagtagga</td><td>vvi-miR395l|-5_perfect match probe</td></tr>
<tr><td>vvi|miR-274|2mut|as|</td><td>ccccaaacccttccgtagga</td><td>vvi-miR395l|-5_mismatch probe</td></tr>
<tr><td>vvi|miR-275|nat|as|</td><td>accaggagttcccccaaaca</td><td>vvi-miR395l|+5_perfect match probe</td></tr>
<tr><td>vvi|miR-275|2mut|as|</td><td>accagaagttccctcaaaca</td><td>vvi-miR395l|+5_mismatch probe</td></tr>
<tr><td>vvi|miR-276|nat|as|</td><td>ccccaaacacttcagtagga</td><td>vvi-miR395m|-5_perfect match probe</td></tr>
<tr><td>vvi|miR-276|2mut|as|</td><td>ccccaaacccttccgtagga</td><td>vvi-miR395m|-5_mismatch probe</td></tr>
<tr><td>vvi|miR-277|nat|as|</td><td>accaggagttcccccaaaca</td><td>vvi-miR395m|+5_perfect match probe</td></tr>
<tr><td>vvi|miR-277|2mut|as|</td><td>accagaagttccctcaaaca</td><td>vvi-miR395m|+5_mismatch probe</td></tr>
<tr><td>vvi|miR-278|nat|as|</td><td>ctccagactcttcagtagag</td><td>vvi-miR395n|-5_perfect match probe</td></tr>
<tr><td>vvi|miR-278|2mut|as|</td><td>ctccagcctcttccgtagag</td><td>vvi-miR395n|-5_mismatch probe</td></tr>
<tr><td>vvi|miR-279|nat|as|</td><td>accaagagttcctccagact</td><td>vvi-miR395n|+5_perfect match probe</td></tr>
<tr><td>vvi|miR-279|2mut|as|</td><td>accaagacttcctcaagact</td><td>vvi-miR395n|+5_mismatch probe</td></tr>
<tr><td>vvi|miR-280|nat|as|</td><td>agaaagctgtgggaggacat</td><td>vvi-miR396a|-5_perfect match probe</td></tr>
</table>

vvi|miR-280|2mut|as|	agaaagttgtgtgaggacat	vvi-miR396a|-5_mismatch probe
vvi|miR-281|nat|as|	aatagaagctcaagaaagct	vvi-miR396a|+5_perfect match probe
vvi|miR-281|2mut|as|	aatagaagttaaagaaagct	vvi-miR396a|+5_mismatch probe
vvi|miR-282|nat|as|	agaaagctgtgggaaaccat	vvi-miR396b|-5_perfect match probe
vvi|miR-282|2mut|as|	agaaagttgtgtgaaaccat	vvi-miR396b|-5_mismatch probe
vvi|miR-283|nat|as|	cactgaagttcaagaaagct	vvi-miR396b|+5_perfect match probe
vvi|miR-283|2mut|as|	cactaaagttaaagaaagct	vvi-miR396b|+5_mismatch probe
vvi|miR-284|nat|as|	aagaaagctgtggaagatca	vvi-miR396c|-5_perfect match probe
vvi|miR-284|2mut|as|	aagaaagttgtcgaagatca	vvi-miR396c|-5_mismatch probe
vvi|miR-285|nat|as|	caaaacagttcaagaaagct	vvi-miR396c|+5_perfect match probe
vvi|miR-285|2mut|as|	caaaaaagttaaagaaagct	vvi-miR396c|+5_mismatch probe
vvi|miR-286|nat|as|	aagaaagctgtggaagaata	vvi-miR396d|-5_perfect match probe
vvi|miR-286|2mut|as|	aagaaagttgtcgaagaata	vvi-miR396d|-5_mismatch probe
vvi|miR-287|nat|as|	aaatgcagttcaagaaagct	vvi-miR396d|+5_perfect match probe
vvi|miR-287|2mut|as|	aaatgaagttaaagaaagct	vvi-miR396d|+5_mismatch probe
vvi|miR-288|nat|as|	cgctgcactcaatgatgttt	vvi-miR397a|-5_perfect match probe
vvi|miR-288|2mut|as|	cgctgaactcaataatgttt	vvi-miR397a|-5_mismatch probe
vvi|miR-289|nat|as|	agtttcatcaacgctgcact	vvi-miR397a|+5_perfect match probe
vvi|miR-289|2mut|as|	agtttcataaaccctgcact	vvi-miR397a|+5_mismatch probe
vvi|miR-290|nat|as|	cgctgcactcaatgatgttt	vvi-miR397b|-5_perfect match probe
vvi|miR-290|2mut|as|	cgctgaactcaataatgttt	vvi-miR397b|-5_mismatch probe
vvi|miR-291|nat|as|	agtttcatcaacgctgcact	vvi-miR397b|+5_perfect match probe
vvi|miR-291|2mut|as|	agtttcataaaccctgcact	vvi-miR397b|+5_mismatch probe
vvi|miR-292|nat|as|	tgacctgagaacacaaaatg	vvi-miR398a|-5_perfect match probe
vvi|miR-292|2mut|as|	tgacttgagaacccaaaatg	vvi-miR398a|-5_mismatch probe
vvi|miR-293|nat|as|	ccccaaaggggtgacctgag	vvi-miR398a|+5_perfect match probe
vvi|miR-293|2mut|as|	ccccaaaggtgtgatctgag	vvi-miR398a|+5_mismatch probe
vvi|miR-294|nat|as|	cgacctgagaacacatgaga	vvi-miR398b|-5_perfect match probe
vvi|miR-294|2mut|as|	cgacttgagaacccatgaga	vvi-miR398b|-5_mismatch probe
vvi|miR-295|nat|as|	tccagcaggggcgacctgag	vvi-miR398b|+5_perfect match probe
vvi|miR-295|2mut|as|	tccagcaggtgagacctgag	vvi-miR398b|+5_mismatch probe
vvi|miR-296|nat|as|	cgacctgagaacacatgaga	vvi-miR398c|-5_perfect match probe
vvi|miR-296|2mut|as|	cgacttgagaacccatgaga	vvi-miR398c|-5_mismatch probe
vvi|miR-297|nat|as|	tccagcaggggcgacctgag	vvi-miR398c|+5_perfect match probe

vvi|miR-297|2mut|as|	tccagcaggtgagacctgag	vvi-miR398c|+5_mismatch probe
vvi|miR-298|nat|as|	aattctcctttggcagagca	vvi-miR399a|-5_perfect match probe
vvi|miR-298|2mut|as|	aattcttctttgccagagca	vvi-miR399a|-5_mismatch probe
vvi|miR-299|nat|as|	aataacagggcaattctcct	vvi-miR399a|+5_perfect match probe
vvi|miR-299|2mut|as|	aataacaggcaaattctcct	vvi-miR399a|+5_mismatch probe
vvi|miR-300|nat|as|	aactctcctttggcaagtca	vvi-miR399b|-5_perfect match probe
vvi|miR-300|2mut|as|	aactcttctttgccaagtca	vvi-miR399b|-5_mismatch probe
vvi|miR-301|nat|as|	agtcacagggcaactctcct	vvi-miR399b|+5_perfect match probe
vvi|miR-301|2mut|as|	agtcacaggcaaactctcct	vvi-miR399b|+5_mismatch probe
vvi|miR-302|nat|as|	aactctcctttggcagatca	vvi-miR399c|-5_perfect match probe
vvi|miR-302|2mut|as|	aactcttctttgccagatca	vvi-miR399c|-5_mismatch probe
vvi|miR-303|nat|as|	agacacagggcaactctcct	vvi-miR399c|+5_perfect match probe
vvi|miR-303|2mut|as|	agacacaggcaaactctcct	vvi-miR399c|+5_mismatch probe
vvi|miR-304|nat|as|	aaatctcctttggcagacac	vvi-miR399d|-5_perfect match probe
vvi|miR-304|2mut|as|	aaatcttctttgccagacac	vvi-miR399d|-5_mismatch probe
vvi|miR-305|nat|as|	aattcacgagcaaatctcct	vvi-miR399d|+5_perfect match probe
vvi|miR-305|2mut|as|	aattcaagagaaaatctcct	vvi-miR399d|+5_mismatch probe
vvi|miR-306|nat|as|	aaatctcctttggcagaggg	vvi-miR399e|-5_perfect match probe
vvi|miR-306|2mut|as|	aaatcttctttgccagaggg	vvi-miR399e|-5_mismatch probe
vvi|miR-307|nat|as|	aattgccgggcaaatctcct	vvi-miR399e|+5_perfect match probe
vvi|miR-307|2mut|as|	aattgccggcaaaatctcct	vvi-miR399e|+5_mismatch probe
vvi|miR-308|nat|as|	aaatctccttcggcagcgag	vvi-miR399f|-5_perfect match probe
vvi|miR-308|2mut|as|	aaatcttcttcgccagcgag	vvi-miR399f|-5_mismatch probe
vvi|miR-309|nat|as|	aattgcaggacaaatctcct	vvi-miR399f|+5_perfect match probe
vvi|miR-309|2mut|as|	aattgaaggaaaaatctcct	vvi-miR399f|+5_mismatch probe
vvi|miR-310|nat|as|	aaatctcctttggcagtgag	vvi-miR399g|-5_perfect match probe
vvi|miR-310|2mut|as|	aaatcttctttgccagtgag	vvi-miR399g|-5_mismatch probe
vvi|miR-311|nat|as|	aattgaggggcaaatctcct	vvi-miR399g|+5_perfect match probe
vvi|miR-311|2mut|as|	aattgagggcaaaatctcct	vvi-miR399g|+5_mismatch probe
vvi|miR-312|nat|as|	aattctcctttggcaaaaca	vvi-miR399h|-5_perfect match probe
vvi|miR-312|2mut|as|	aattcttctttgccaaaaca	vvi-miR399h|-5_mismatch probe
vvi|miR-313|nat|as|	aatggcagggcaattctcct	vvi-miR399h|+5_perfect match probe
vvi|miR-313|2mut|as|	aatggcaggcaaattctcct	vvi-miR399h|+5_mismatch probe
vvi|miR-314|nat|as|	aactctcctttggcgggtca	vvi-miR399i|-5_perfect match probe

vvi|miR-314|2mut|as|	aactcttctttggagggtca	vvi-miR399i|-5_mismatch probe
vvi|miR-315|nat|as|	agtcacagggcaactctcct	vvi-miR399i|+5_perfect match probe
vvi|miR-315|2mut|as|	agtcacaggcaaactctcct	vvi-miR399i|+5_mismatch probe
vvi|miR-316|nat|as|	tgtgcgtgaatctaacgccg	vvi-miR403a|-5_perfect match probe
vvi|miR-316|2mut|as|	tgtgagtgaatctaaagccg	vvi-miR403a|-5_mismatch probe
vvi|miR-317|nat|as|	actcacgagtttgtgcgtga	vvi-miR403a|+5_perfect match probe
vvi|miR-317|2mut|as|	actcaagagtttgtgagtga	vvi-miR403a|+5_mismatch probe
vvi|miR-318|nat|as|	tgtgcgtgaatctaacaccc	vvi-miR403b|-5_perfect match probe
vvi|miR-318|2mut|as|	tgtgagtgaatctaaaaccc	vvi-miR403b|-5_mismatch probe
vvi|miR-319|nat|as|	gatcccgagtttgtgcgtga	vvi-miR403b|+5_perfect match probe
vvi|miR-319|2mut|as|	gatccagagtttgtgagtga	vvi-miR403b|+5_mismatch probe
vvi|miR-320|nat|as|	tgtgcgtgaatctaacggcg	vvi-miR403c|-5_perfect match probe
vvi|miR-320|2mut|as|	tgtgagtgaatctaaaggcg	vvi-miR403c|-5_mismatch probe
vvi|miR-321|nat|as|	gatcacgagtttgtgcgtga	vvi-miR403c|+5_perfect match probe
vvi|miR-321|2mut|as|	gatcaagagtttgtgagtga	vvi-miR403c|+5_mismatch probe
vvi|miR-322|nat|as|	tgtgcgtgaatctaacaccc	vvi-miR403d|-5_perfect match probe
vvi|miR-322|2mut|as|	tgtgagtgaatctaaaaccc	vvi-miR403d|-5_mismatch probe
vvi|miR-323|nat|as|	gatcccgagtttgtgcgtga	vvi-miR403d|+5_perfect match probe
vvi|miR-323|2mut|as|	gatccagagtttgtgagtga	vvi-miR403d|+5_mismatch probe
vvi|miR-324|nat|as|	tgtgcgtgaatctaacggcg	vvi-miR403e|-5_perfect match probe
vvi|miR-324|2mut|as|	tgtgagtgaatctaaaggcg	vvi-miR403e|-5_mismatch probe
vvi|miR-325|nat|as|	gatcgcgagtttgtgcgtga	vvi-miR403e|+5_perfect match probe
vvi|miR-325|2mut|as|	gatcgagagtttgtgagtga	vvi-miR403e|+5_mismatch probe
vvi|miR-326|nat|as|	tgtgcgtgaatctaagacca	vvi-miR403f|-5_perfect match probe
vvi|miR-326|2mut|as|	tgtgagtgaatctaacacca	vvi-miR403f|-5_mismatch probe
vvi|miR-327|nat|as|	gattacgagtttgtgcgtga	vvi-miR403f|+5_perfect match probe
vvi|miR-327|2mut|as|	gattaagagtttgtgagtga	vvi-miR403f|+5_mismatch probe
vvi|miR-328|nat|as|	gaagaggcagtgcatgggta	vvi-miR408|-5_perfect match probe
vvi|miR-328|2mut|as|	gaagagccagtgaatgggta	vvi-miR408|-5_mismatch probe
vvi|miR-329|nat|as|	acagagccagggaagaggca	vvi-miR408|+5_perfect match probe
vvi|miR-329|2mut|as|	acagagtcagtgaagaggca	vvi-miR408|+5_mismatch probe
vvi|miR-330|nat|as|	gcctttgagggagattgaca	vvi-miR477a|-5_perfect match probe
vvi|miR-330|2mut|as|	gcctttgagtaagattgaca	vvi-miR477a|-5_mismatch probe
vvi|miR-331|nat|as|	gaaaattggaagcctttgag	vvi-miR477a|+5_perfect match probe

vvi|miR-331|2mut|as|	gaaaattcgaagtctttgag	vvi-miR477a|+5_mismatch probe
vvi|miR-332|nat|as|	cgaaccaataccacaccatg	vvi-miR479|-5_perfect match probe
vvi|miR-332|2mut|as|	cgaacaaatacaacaccatg	vvi-miR479|-5_mismatch probe
vvi|miR-333|nat|as|	aagaagatgagccgaaccaa	vvi-miR479|+5_perfect match probe
vvi|miR-333|2mut|as|	aagaagataagtcgaaccaa	vvi-miR479|+5_mismatch probe
vvi|miR-334|nat|as|	aatgggaggagtaggaaaga	vvi-miR482a|-5_perfect match probe
vvi|miR-334|2mut|as|	aatgtgaggagtagaaaaga	vvi-miR482a|-5_mismatch probe
vvi|miR-335|nat|as|	caataggaatgggaggagta	vvi-miR482a|+5_perfect match probe
vvi|miR-335|2mut|as|	caatagaaatgtgaggagta	vvi-miR482a|+5_mismatch probe
vvi|miR-336|nat|as|	tctctctcgttgtcaagcaa	vvi-miR535a|-5_perfect match probe
vvi|miR-336|2mut|as|	tctctctccttctcaagcaa	vvi-miR535a|-5_mismatch probe
vvi|miR-337|nat|as|	tgactagcgtgctctctctc	vvi-miR535a|+5_perfect match probe
vvi|miR-337|2mut|as|	tgactagagtcctctctctc	vvi-miR535a|+5_mismatch probe
vvi|miR-338|nat|as|	tctctctcgttgtcaagcaa	vvi-miR535b|-5_perfect match probe
vvi|miR-338|2mut|as|	tctctctccttctcaagcaa	vvi-miR535b|-5_mismatch probe
vvi|miR-339|nat|as|	tgactagcgtgctctctctc	vvi-miR535b|+5_perfect match probe
vvi|miR-339|2mut|as|	tgactagagtcctctctctc	vvi-miR535b|+5_mismatch probe
vvi|miR-340|nat|as|	tctctctcgttgtcaagcaa	vvi-miR535c|-5_perfect match probe
vvi|miR-340|2mut|as|	tctctctccttctcaagcaa	vvi-miR535c|-5_mismatch probe
vvi|miR-341|nat|as|	tgactagcgtgctctctctc	vvi-miR535c|+5_perfect match probe
vvi|miR-341|2mut|as|	tgactagagtcctctctctc	vvi-miR535c|+5_mismatch probe
vvi|miR-342|nat|as|	tctctctcgttgtcaagcaa	vvi-miR535d|-5_perfect match probe
vvi|miR-342|2mut|as|	tctctctccttctcaagcaa	vvi-miR535d|-5_mismatch probe
vvi|miR-343|nat|as|	agactagcgtgctctctctc	vvi-miR535d|+5_perfect match probe
vvi|miR-343|2mut|as|	agactagagtcctctctctc	vvi-miR535d|+5_mismatch probe
vvi|miR-344|nat|as|	tctctctcgttgtcaagcaa	vvi-miR535e|-5_perfect match probe
vvi|miR-344|2mut|as|	tctctctccttctcaagcaa	vvi-miR535e|-5_mismatch probe
vvi|miR-345|nat|as|	agactagcgtgctctctctc	vvi-miR535e|+5_perfect match probe
vvi|miR-345|2mut|as|	agactagagtcctctctctc	vvi-miR535e|+5_mismatch probe
vvi|miR-346|nat|as|	ctcatttgagcaagaaactt	vvi-miR828a|-5_perfect match probe
vvi|miR-346|2mut|as|	ctcatttgacaaagaaactt	vvi-miR828a|-5_mismatch probe
vvi|miR-347|nat|as|	ttgtttggaatactcatttg	vvi-miR828a|+5_perfect match probe
vvi|miR-347|2mut|as|	ttgtttcgaatactaatttg	vvi-miR828a|+5_mismatch probe
vvi|miR-348|nat|as|	ctcatttgagcaagaaacca	vvi-miR828b|-5_perfect match probe

vvi|miR-348|2mut|as|	ctcatttgacaaagaaacca	vvi-miR828b|-5_mismatch probe
vvi|miR-349|nat|as|	tgatgtggaacactcatttg	vvi-miR828b|+5_perfect match probe
vvi|miR-349|2mut|as|	tgatgtcgaaccctcatttg	vvi-miR828b|+5_mismatch probe
vvi|miR-350|nat|as|	ttggtatcagagctagacct	vvi-miR845a|-5_perfect match probe
vvi|miR-350|2mut|as|	ttggtatccgagttagacct	vvi-miR845a|-5_mismatch probe
vvi|miR-351|nat|as|	tgttttatcaattggtatca	vvi-miR845a|+5_perfect match probe
vvi|miR-351|2mut|as|	tgttttataaattcgtatca	vvi-miR845a|+5_mismatch probe
vvi|miR-352|nat|as|	ttggtatcagagctagacct	vvi-miR845b|-5_perfect match probe
vvi|miR-352|2mut|as|	ttggtatccgagttagacct	vvi-miR845b|-5_mismatch probe
vvi|miR-353|nat|as|	tgttttatcaattggtatca	vvi-miR845b|+5_perfect match probe
vvi|miR-353|2mut|as|	tgttttataaattcgtatca	vvi-miR845b|+5_mismatch probe
vvi|miR-354|nat|as|	ttggtatcagagcctcatgt	vvi-miR845c|-5_perfect match probe
vvi|miR-354|2mut|as|	ttggtatccgagtctcatgt	vvi-miR845c|-5_mismatch probe
vvi|miR-355|nat|as|	ttatgcatcaattggtatca	vvi-miR845c|+5_perfect match probe
vvi|miR-355|2mut|as|	ttatgaatcaattcgtatca	vvi-miR845c|+5_mismatch probe
vvi|miR-356|nat|as|	ttggtatcagagccataggt	vvi-miR845d|-5_perfect match probe
vvi|miR-356|2mut|as|	ttggtatccgagtcataggt	vvi-miR845d|-5_mismatch probe
vvi|miR-357|nat|as|	tttcccatcaattggtatca	vvi-miR845d|+5_perfect match probe
vvi|miR-357|2mut|as|	tttctcatcaattcgtatca	vvi-miR845d|+5_mismatch probe
vvi|miR-358|nat|as|	ttggtatcagagccataggt	vvi-miR845e|-5_perfect match probe
vvi|miR-358|2mut|as|	ttggtatccgagtcataggt	vvi-miR845e|-5_mismatch probe
vvi|miR-359|nat|as|	tttcccatcaattggtatca	vvi-miR845e|+5_perfect match probe
vvi|miR-359|2mut|as|	tttctcatcaattcgtatca	vvi-miR845e|+5_mismatch probe
vvi|miR-360|nat|as|	tcttctgtcaacatatttct	vvi-miR156a|-10_perfect match probe
vvi|miR-360|2mut|as|	tcttctctcaaaatatttct	vvi-miR156a|-10_mismatch probe
vvi|miR-361|nat|as|	atgctgggttgtgctccctc	vvi-miR156a|+10_perfect match probe
vvi|miR-361|2mut|as|	atgctgtgttgtcctccctc	vvi-miR156a|+10_mismatch probe
vvi|miR-362|nat|as|	tcttctgtcagttttcaatg	vvi-miR156b|-10_perfect match probe
vvi|miR-362|2mut|as|	tcttctctcagttttaaatg	vvi-miR156b|-10_mismatch probe
vvi|miR-363|nat|as|	ttgcctctgtgtgctcactc	vvi-miR156b|+10_perfect match probe

vvi|miR-363|2mut|as|	ttgcttctgtgtcctcactc	vvi-miR156b|+10_mismatch probe
		vvi-miR156c|-10_perfect match
vvi|miR-364|nat|as|	tcttctgtcacctattcctt	probe
vvi|miR-364|2mut|as|	tcttctgtcctctattcctt	vvi-miR156c|-10_mismatch probe
		vvi-miR156c|+10_perfect match
vvi|miR-365|nat|as|	cagaccatgtgtgctcactc	probe
vvi|miR-365|2mut|as|	cagacaatgtgtcctcactc	vvi-miR156c|+10_mismatch probe
		vvi-miR156d|-10_perfect match
vvi|miR-366|nat|as|	tcttctgtcagtctctctct	probe
vvi|miR-366|2mut|as|	tcttctctcagcctctctct	vvi-miR156d|-10_mismatch probe
		vvi-miR156d|+10_perfect match
vvi|miR-367|nat|as|	ttgcctgcatgtgctcactc	probe
vvi|miR-367|2mut|as|	ttgcctgaatgtcctcactc	vvi-miR156d|+10_mismatch probe
		vvi-miR156e|-10_perfect match
vvi|miR-368|nat|as|	tcctctgtcacctaccatac	probe
vvi|miR-368|2mut|as|	tcctctgtcccctacaatac	vvi-miR156e|-10_mismatch probe
		vvi-miR156e|+10_perfect match
vvi|miR-369|nat|as|	agaaccatgagtgctcactc	probe
vvi|miR-369|2mut|as|	agaacaatgagtcctcactc	vvi-miR156e|+10_mismatch probe
vvi|miR-370|nat|as|	cttctgtcaacagaacataa	vvi-miR156f|-10_perfect match probe
vvi|miR-370|2mut|as|	cttctctcaacagaaaataa	vvi-miR156f|-10_mismatch probe
vvi|miR-371|nat|as|	tgctctctatcttctgtcaa	vvi-miR156f|0_perfect match probe
vvi|miR-371|2mut|as|	tgctccctatcttctctcaa	vvi-miR156f|0_mismatch probe
		vvi-miR156f|+10_perfect match
vvi|miR-372|nat|as|	tgttcagttgtgctctctat	probe
vvi|miR-372|2mut|as|	tgttcagttctcctctctat	vvi-miR156f|+10_mismatch probe
		vvi-miR156g|-10_perfect match
vvi|miR-373|nat|as|	cttctgtcaacaacatcacc	probe
vvi|miR-373|2mut|as|	cttctctcaacaaaatcacc	vvi-miR156g|-10_mismatch probe
vvi|miR-374|nat|as|	tgctctctatcttctgtcaa	vvi-miR156g|0_perfect match probe
vvi|miR-374|2mut|as|	tgctccctatcttctctcaa	vvi-miR156g|0_mismatch probe
		vvi-miR156g|+10_perfect match
vvi|miR-375|nat|as|	tcatcatctgtgctctctat	probe
vvi|miR-375|2mut|as|	tcatcatctctcctctctat	vvi-miR156g|+10_mismatch probe

<table>
<tr><td></td><td></td><td>vvi-miR156h|-10_perfect match</td></tr>
<tr><td>vvi|miR-376|nat|as|</td><td>tcttctgtcattgtgaggca</td><td>probe</td></tr>
<tr><td>vvi|miR-376|2mut|as|</td><td>tcttctctcattgcgaggca</td><td>vvi-miR156h|-10_mismatch probe</td></tr>
<tr><td></td><td></td><td>vvi-miR156h|+10_perfect match</td></tr>
<tr><td>vvi|miR-377|nat|as|</td><td>tcccaccagcatgctctctc</td><td>probe</td></tr>
<tr><td>vvi|miR-377|2mut|as|</td><td>tcccaccagaatcctctctc</td><td>vvi-miR156h|+10_mismatch probe</td></tr>
<tr><td>vvi|miR-378|nat|as|</td><td>tcttctgtcaacaacgtcac</td><td>vvi-miR156i|-10_perfect match probe</td></tr>
<tr><td>vvi|miR-378|2mut|as|</td><td>tcttctctcaacaacctcac</td><td>vvi-miR156i|-10_mismatch probe</td></tr>
<tr><td></td><td></td><td>vvi-miR156i|+10_perfect match</td></tr>
<tr><td>vvi|miR-379|nat|as|</td><td>ttcatcatctgtgctctcta</td><td>probe</td></tr>
<tr><td>vvi|miR-379|2mut|as|</td><td>ttcatcatccgtcctctcta</td><td>vvi-miR156i|+10_mismatch probe</td></tr>
<tr><td></td><td></td><td>vvi-miR159a|-10_perfect match</td></tr>
<tr><td>vvi|miR-380|nat|as|</td><td>tcactccaaggccagaaaga</td><td>probe</td></tr>
<tr><td>vvi|miR-380|2mut|as|</td><td>tcactcaaaggtcagaaaga</td><td>vvi-miR159a|-10_mismatch probe</td></tr>
<tr><td>vvi|miR-381|nat|as|</td><td>agagctcccttcactccaag</td><td>vvi-miR159a|0_perfect match probe</td></tr>
<tr><td>vvi|miR-381|2mut|as|</td><td>agagctctcttcacttcaag</td><td>vvi-miR159a|0_mismatch probe</td></tr>
<tr><td></td><td></td><td>vvi-miR159a|+10_perfect match</td></tr>
<tr><td>vvi|miR-382|nat|as|</td><td>aagggttatgagagctccct</td><td>probe</td></tr>
<tr><td>vvi|miR-382|2mut|as|</td><td>aaggcttatgagagttccct</td><td>vvi-miR159a|+10_mismatch probe</td></tr>
<tr><td></td><td></td><td>vvi-miR159b|-10_perfect match</td></tr>
<tr><td>vvi|miR-383|nat|as|</td><td>tcactccaaggccagaaaag</td><td>probe</td></tr>
<tr><td>vvi|miR-383|2mut|as|</td><td>tcactcaaaggtcagaaaag</td><td>vvi-miR159b|-10_mismatch probe</td></tr>
<tr><td>vvi|miR-384|nat|as|</td><td>agagctcccttcactccaag</td><td>vvi-miR159b|0_perfect match probe</td></tr>
<tr><td>vvi|miR-384|2mut|as|</td><td>agagctctcttcacttcaag</td><td>vvi-miR159b|0_mismatch probe</td></tr>
<tr><td></td><td></td><td>vvi-miR159b|+10_perfect match</td></tr>
<tr><td>vvi|miR-385|nat|as|</td><td>atcggttatgagagctccct</td><td>probe</td></tr>
<tr><td>vvi|miR-385|2mut|as|</td><td>atcgcttatgagagttccct</td><td>vvi-miR159b|+10_mismatch probe</td></tr>
<tr><td></td><td></td><td>vvi-miR159c|-10_perfect match</td></tr>
<tr><td>vvi|miR-386|nat|as|</td><td>tcaatccaaacaaggatcta</td><td>probe</td></tr>
<tr><td>vvi|miR-386|2mut|as|</td><td>tcaatcaaaacaagaatcta</td><td>vvi-miR159c|-10_mismatch probe</td></tr>
<tr><td>vvi|miR-387|nat|as|</td><td>agagctcccttcaatccaaa</td><td>vvi-miR159c|0_perfect match probe</td></tr>
<tr><td>vvi|miR-387|2mut|as|</td><td>agagctctcttcaattcaaa</td><td>vvi-miR159c|0_mismatch probe</td></tr>
<tr><td></td><td></td><td>vvi-miR159c|+10_perfect match</td></tr>
<tr><td>vvi|miR-388|nat|as|</td><td>agagaggtgtagagctccct</td><td>probe</td></tr>
</table>

vvi|miR-388|2mut|as|	agagagctgtagagttccct
vvi|miR-389|nat|as|	gagccaggcagatcaacccc
vvi|miR-389|2mut|as|	gagccagccagataaacccc
vvi|miR-390|nat|as|	ggcattcagggagccaggca
vvi|miR-390|2mut|as|	ggcattcagtgagtcaggca
vvi|miR-391|nat|as|	cttcttagatggcattcagg
vvi|miR-391|2mut|as|	cttcttacatgccattcagg
vvi|miR-392|nat|as|	gagccaggcagaacaatatt
vvi|miR-392|2mut|as|	gagccagccagaaaaatatt
vvi|miR-393|nat|as|	ggcattcagggagccaggca
vvi|miR-393|2mut|as|	ggcattcagtgagtcaggca
vvi|miR-394|nat|as|	cttcttagatggcattcagg
vvi|miR-394|2mut|as|	cttcttacatgccattcagg
vvi|miR-395|nat|as|	gagccaggcacaacccattt
vvi|miR-395|2mut|as|	gagccagccacaactcattt
vvi|miR-396|nat|as|	ggcatacagggagccaggca
vvi|miR-396|2mut|as|	ggcatacagtgagtcaggca
vvi|miR-397|nat|as|	ctctacgtgtggcatacagg
vvi|miR-397|2mut|as|	ctctacctgtgccatacagg
vvi|miR-398|nat|as|	gagccaggcatgtaagtatg
vvi|miR-398|2mut|as|	gagccagccatctaagtatg
vvi|miR-399|nat|as|	ggcatacagggagccaggca
vvi|miR-399|2mut|as|	ggcatacagtgagtcaggca
vvi|miR-400|nat|as|	ctttgcaaatggcatacagg
vvi|miR-400|2mut|as|	ctttgaaaatgccatacagg
vvi|miR-401|nat|as|	gagccaggcagaacaatatt

vvi-miR159c|+10_mismatch probe
vvi-miR160a|-10_perfect match probe
vvi-miR160a|-10_mismatch probe
vvi-miR160a|0_perfect match probe
vvi-miR160a|0_mismatch probe
vvi-miR160a|+10_perfect match probe
vvi-miR160a|+10_mismatch probe
vvi-miR160b|-10_perfect match probe
vvi-miR160b|-10_mismatch probe
vvi-miR160b|0_perfect match probe
vvi-miR160b|0_mismatch probe
vvi-miR160b|+10_perfect match probe
vvi-miR160b|+10_mismatch probe
vvi-miR160c|-10_perfect match probe
vvi-miR160c|-10_mismatch probe
vvi-miR160c|0_perfect match probe
vvi-miR160c|0_mismatch probe
vvi-miR160c|+10_perfect match probe
vvi-miR160c|+10_mismatch probe
vvi-miR160d|-10_perfect match probe
vvi-miR160d|-10_mismatch probe
vvi-miR160d|0_perfect match probe
vvi-miR160d|0_mismatch probe
vvi-miR160d|+10_perfect match probe
vvi-miR160d|+10_mismatch probe
vvi-miR160e|-10_perfect match

<table>
<tr><td></td><td></td><td>probe</td></tr>
<tr><td>vvi|miR-401|2mut|as|</td><td>gagccagccagaaaaatatt</td><td>vvi-miR160e|-10_mismatch probe</td></tr>
<tr><td>vvi|miR-402|nat|as|</td><td>ggcattcagggagccaggca</td><td>vvi-miR160e|0_perfect match probe</td></tr>
<tr><td>vvi|miR-402|2mut|as|</td><td>ggcattcagtgagtcaggca</td><td>vvi-miR160e|0_mismatch probe</td></tr>
<tr><td></td><td></td><td>vvi-miR160e|+10_perfect match</td></tr>
<tr><td>vvi|miR-403|nat|as|</td><td>cttcttagatggcattcagg</td><td>probe</td></tr>
<tr><td>vvi|miR-403|2mut|as|</td><td>cttcttacatgccattcagg</td><td>vvi-miR160e|+10_mismatch probe</td></tr>
<tr><td>vvi|miR-404|nat|as|</td><td>gagccaggcatatatgctta</td><td>vvi-miR160f|-10_perfect match probe</td></tr>
<tr><td>vvi|miR-404|2mut|as|</td><td>gagccagccatatatcctta</td><td>vvi-miR160f|-10_mismatch probe</td></tr>
<tr><td>vvi|miR-405|nat|as|</td><td>ggcatacagggagccaggca</td><td>vvi-miR160f|0_perfect match probe</td></tr>
<tr><td>vvi|miR-405|2mut|as|</td><td>ggcatacagtgagtcaggca</td><td>vvi-miR160f|0_mismatch probe</td></tr>
<tr><td></td><td></td><td>vvi-miR160f|+10_perfect match</td></tr>
<tr><td>vvi|miR-406|nat|as|</td><td>ctctgcaaatggcatacagg</td><td>probe</td></tr>
<tr><td>vvi|miR-406|2mut|as|</td><td>ctctgaaaatgccatacagg</td><td>vvi-miR160f|+10_mismatch probe</td></tr>
<tr><td>vvi|miR-407|nat|as|</td><td>ggtttatcgaccgattcttg</td><td>vvi-miR162|-10_perfect match probe</td></tr>
<tr><td>vvi|miR-407|2mut|as|</td><td>ggtttatagacagattcttg</td><td>vvi-miR162|-10_mismatch probe</td></tr>
<tr><td>vvi|miR-408|nat|as|</td><td>tggatgcagaggtttatcga</td><td>vvi-miR162|0_perfect match probe</td></tr>
<tr><td>vvi|miR-408|2mut|as|</td><td>tggatgaagagctttatcga</td><td>vvi-miR162|0_mismatch probe</td></tr>
<tr><td>vvi|miR-409|nat|as|</td><td>gagtgaacgctggatgcaga</td><td>vvi-miR162|+10_perfect match probe</td></tr>
<tr><td>vvi|miR-409|2mut|as|</td><td>gagtgaaccctggatccaga</td><td>vvi-miR162|+10_mismatch probe</td></tr>
<tr><td></td><td></td><td>vvi-miR164a|-10_perfect match</td></tr>
<tr><td>vvi|miR-410|nat|as|</td><td>tgcttctccaacaaggagct</td><td>probe</td></tr>
<tr><td>vvi|miR-410|2mut|as|</td><td>tgcttctcaaacaagaagct</td><td>vvi-miR164a|-10_mismatch probe</td></tr>
<tr><td>vvi|miR-411|nat|as|</td><td>gcacgtgccctgcttctcca</td><td>vvi-miR164a|0_perfect match probe</td></tr>
<tr><td>vvi|miR-411|2mut|as|</td><td>gcacgtgtcctccttctcca</td><td>vvi-miR164a|0_mismatch probe</td></tr>
<tr><td></td><td></td><td>vvi-miR164a|+10_perfect match</td></tr>
<tr><td>vvi|miR-412|nat|as|</td><td>aggcaaatctgcacgtgccc</td><td>probe</td></tr>
<tr><td>vvi|miR-412|2mut|as|</td><td>aggcaaatccgaacgtgccc</td><td>vvi-miR164a|+10_mismatch probe</td></tr>
<tr><td></td><td></td><td>vvi-miR164b|-10_perfect match</td></tr>
<tr><td>vvi|miR-413|nat|as|</td><td>tgcttctccagcatggttta</td><td>probe</td></tr>
<tr><td>vvi|miR-413|2mut|as|</td><td>tgcttctcaagaatggttta</td><td>vvi-miR164b|-10_mismatch probe</td></tr>
<tr><td>vvi|miR-414|nat|as|</td><td>gcatgtgccctgcttctcca</td><td>vvi-miR164b|0_perfect match probe</td></tr>
<tr><td>vvi|miR-414|2mut|as|</td><td>gcatgtgtcctccttctcca</td><td>vvi-miR164b|0_mismatch probe</td></tr>
<tr><td>vvi|miR-415|nat|as|</td><td>gattgatccagcatgtgccc</td><td>vvi-miR164b|+10_perfect match</td></tr>
</table>

		probe
vvi|miR-415|2mut|as|	gattgatcaagaatgtgccc	vvi-miR164b|+10_mismatch probe
vvi|miR-416|nat|as|	tgcttctccatcaagagctt	vvi-miR164d|-10_perfect match probe
vvi|miR-416|2mut|as|	tgcttctcaataaagagctt	vvi-miR164d|-10_mismatch probe
vvi|miR-417|nat|as|	gcacgtgccctgcttctcca	vvi-miR164d|0_perfect match probe
vvi|miR-417|2mut|as|	gcacgtgtcctccttctcca	vvi-miR164d|0_mismatch probe
vvi|miR-418|nat|as|	tttgtgaactgcacgtgccc	vvi-miR164d|+10_perfect match probe
vvi|miR-418|2mut|as|	tttgcgaactgaacgtgccc	vvi-miR164d|+10_mismatch probe
vvi|miR-419|nat|as|	cctggtccgacgacaccaac	vvi-miR166c|-10_perfect match probe
vvi|miR-419|2mut|as|	cctggtcagaagacaccaac	vvi-miR166c|-10_mismatch probe
vvi|miR-420|nat|as|	gggaatgaagcctggtccga	vvi-miR166c|0_perfect match probe
vvi|miR-420|2mut|as|	gggaatgaactctggtccga	vvi-miR166c|0_mismatch probe
vvi|miR-421|nat|as|	gataattggggggaatgaag	vvi-miR166c|+10_perfect match probe
vvi|miR-421|2mut|as|	gataattggtgtgaatgaag	vvi-miR166c|+10_mismatch probe
vvi|miR-422|nat|as|	cctggtccgagggcactaac	vvi-miR166d|-10_perfect match probe
vvi|miR-422|2mut|as|	cctggtcagaggccactaac	vvi-miR166d|-10_mismatch probe
vvi|miR-423|nat|as|	gggaatgaagcctggtccga	vvi-miR166d|0_perfect match probe
vvi|miR-423|2mut|as|	gggaatgaactctggtccga	vvi-miR166d|0_mismatch probe
vvi|miR-424|nat|as|	agtgattgaggggaatgaag	vvi-miR166d|+10_perfect match probe
vvi|miR-424|2mut|as|	agtgattgacgtgaatgaag	vvi-miR166d|+10_mismatch probe
vvi|miR-425|nat|as|	cctggtccgacgacaccaac	vvi-miR166e|-10_perfect match probe
vvi|miR-425|2mut|as|	cctggtcagaagacaccaac	vvi-miR166e|-10_mismatch probe
vvi|miR-426|nat|as|	gggaatgaagcctggtccga	vvi-miR166e|0_perfect match probe
vvi|miR-426|2mut|as|	gggaatgaactctggtccga	vvi-miR166e|0_mismatch probe
vvi|miR-427|nat|as|	ataaattggggggaatgaag	vvi-miR166e|+10_perfect match probe
vvi|miR-427|2mut|as|	ataaattggtgtgaatgaag	vvi-miR166e|+10_mismatch probe

vvi|miR-428|nat|as|	cctggtccgaagctttttga	vvi-miR166f|-10_perfect match probe
vvi|miR-428|2mut|as|	cctggtcagaagttttttga	vvi-miR166f|-10_mismatch probe
vvi|miR-429|nat|as|	gggaatgaagcctggtccga	vvi-miR166f|0_perfect match probe
vvi|miR-429|2mut|as|	gggaatgaactctggtccga	vvi-miR166f|0_mismatch probe
		vvi-miR166f|+10_perfect match probe
vvi|miR-430|nat|as|	gtgtgttgaggggaatgaag	
vvi|miR-430|2mut|as|	gtgtgttgacgtgaatgaag	vvi-miR166f|+10_mismatch probe
		vvi-miR166g|-10_perfect match probe
vvi|miR-431|nat|as|	cctggtccgaaatcattcaa	
vvi|miR-431|2mut|as|	cctggtcagaaataattcaa	vvi-miR166g|-10_mismatch probe
vvi|miR-432|nat|as|	gggaatgaagcctggtccga	vvi-miR166g|0_perfect match probe
vvi|miR-432|2mut|as|	gggaatgaactctggtccga	vvi-miR166g|0_mismatch probe
		vvi-miR166g|+10_perfect match probe
vvi|miR-433|nat|as|	ttgggttgaggggaatgaag	
vvi|miR-433|2mut|as|	ttggcttgaggtgaatgaag	vvi-miR166g|+10_mismatch probe
		vvi-miR166h|-10_perfect match probe
vvi|miR-434|nat|as|	cctggtccgagatcattcag	
vvi|miR-434|2mut|as|	cctggtcagagataattcag	vvi-miR166h|-10_mismatch probe
vvi|miR-435|nat|as|	gggaatgaagcctggtccga	vvi-miR166h|0_perfect match probe
vvi|miR-435|2mut|as|	gggaatgaactctggtccga	vvi-miR166h|0_mismatch probe
		vvi-miR166h|+10_perfect match probe
vvi|miR-436|nat|as|	ttgagttggggggaatgaag	
vvi|miR-436|2mut|as|	ttgagttggtgtgaatgaag	vvi-miR166h|+10_mismatch probe
		vvi-miR166a|-10_perfect match probe
vvi|miR-437|nat|as|	cctggtccgagatcaaccga	
vvi|miR-437|2mut|as|	cctggtcagagataaaccga	vvi-miR166a|-10_mismatch probe
vvi|miR-438|nat|as|	aggaatgaagcctggtccga	vvi-miR166a|0_perfect match probe
vvi|miR-438|2mut|as|	aggaatgaactctggtccga	vvi-miR166a|0_mismatch probe
		vvi-miR166a|+10_perfect match probe
vvi|miR-439|nat|as|	gcttggtgtcaggaatgaag	
vvi|miR-439|2mut|as|	gcttcgtgtcagaaatgaag	vvi-miR166a|+10_mismatch probe
		vvi-miR166b|-10_perfect match probe
vvi|miR-440|nat|as|	cctggtccgacaccttcact	
vvi|miR-440|2mut|as|	cctggtcagacatcttcact	vvi-miR166b|-10_mismatch probe

vvi|miR-441|nat|as| aggaatgaagcctggtccga vvi-miR166b|0_perfect match probe
vvi|miR-441|2mut|as| aggaatgaactctggtccga vvi-miR166b|0_mismatch probe
vvi-miR166b|+10_perfect match
vvi|miR-442|nat|as| tatatttgagaggaatgaag probe
vvi|miR-442|2mut|as| tatatttaagagaaatgaag vvi-miR166b|+10_mismatch probe
vvi-miR167a|-10_perfect match
vvi|miR-443|nat|as| ggcagcttcaacctctcaaa probe
vvi|miR-443|2mut|as| ggcagtttcaatctctcaaa vvi-miR167a|-10_mismatch probe
vvi|miR-444|nat|as| agatcatgctggcagcttca vvi-miR167a|0_perfect match probe
vvi|miR-444|2mut|as| agatcatcctgccagcttca vvi-miR167a|0_mismatch probe
vvi-miR167a|+10_perfect match
vvi|miR-445|nat|as| ttgtttcaccagatcatgct probe
vvi|miR-445|2mut|as| ttgtttcacacgatcatgct vvi-miR167a|+10_mismatch probe
vvi-miR167b|-10_perfect match
vvi|miR-446|nat|as| ggcagcttcaactgctattg probe
vvi|miR-446|2mut|as| ggcagtttcaactcctattg vvi-miR167b|-10_mismatch probe
vvi|miR-447|nat|as| agatcatgctggcagcttca vvi-miR167b|0_perfect match probe
vvi|miR-447|2mut|as| agatcatcctgccagcttca vvi-miR167b|0_mismatch probe
vvi-miR167b|+10_perfect match
vvi|miR-448|nat|as| agaaaagcttagatcatgct probe
vvi|miR-448|2mut|as| agaaaagtttagataatgct vvi-miR167b|+10_mismatch probe
vvi-miR167c|-10_perfect match
vvi|miR-449|nat|as| ggcagcttcaactgctactg probe
vvi|miR-449|2mut|as| ggcagtttcaactcctactg vvi-miR167c|-10_mismatch probe
vvi-miR167c|+10_perfect match
vvi|miR-450|nat|as| agggaagttgagatcatgct probe
vvi|miR-450|2mut|as| agggaagttaagataatgct vvi-miR167c|+10_mismatch probe
vvi-miR167d|-10_perfect match
vvi|miR-451|nat|as| ggcagcttcacttattccct probe
vvi|miR-451|2mut|as| ggcagtttcatttattccct vvi-miR167d|-10_mismatch probe
vvi|miR-452|nat|as| agatcatgctggcagcttca vvi-miR167d|0_perfect match probe
vvi|miR-452|2mut|as| agatcatcctgccagcttca vvi-miR167d|0_mismatch probe
vvi-miR167d|+10_perfect match
vvi|miR-453|nat|as| agccaaagctagatcatgct probe

vvi|miR-453|2mut|as|	agccaaagttagataatgct
vvi|miR-454|nat|as|	ggcagcttcaactgatagtg
vvi|miR-454|2mut|as|	ggcagtttcaaccgatagtg
vvi|miR-455|nat|as|	agatcatgctggcagcttca
vvi|miR-455|2mut|as|	agatcatcctgccagcttca
vvi|miR-456|nat|as|	aagcaagtttagatcatgct
vvi|miR-456|2mut|as|	aagcaactttagataatgct
vvi|miR-457|nat|as|	caccaagcgaattagagacc
vvi|miR-457|2mut|as|	caccaagagaattagcgacc
vvi|miR-458|nat|as|	tcccgacctgcaccaagcga
vvi|miR-458|2mut|as|	tcccgacctcaaccaagcga
vvi|miR-459|nat|as|	cgaagtcggttcccgacctg
vvi|miR-459|2mut|as|	cgaagtcagttccagacctg
vvi|miR-460|nat|as|	tccttggctgcattccactc
vvi|miR-460|2mut|as|	tccttgcctgaattccactc
vvi|miR-461|nat|as|	cggcaagtcatccttggctg
vvi|miR-461|2mut|as|	cggcaagccatccttcgctg
vvi|miR-462|nat|as|	atataaattccggcaagtca
vvi|miR-462|2mut|as|	atataaatttcgccaagtca
vvi|miR-463|nat|as|	tccttcgctaccaaacaacg
vvi|miR-463|2mut|as|	tccttccctacaaaacaacg
vvi|miR-464|nat|as|	aggcaagtcatccttcgcta
vvi|miR-464|2mut|as|	aggcaagccatccttagcta
vvi|miR-465|nat|as|	aggaggctgtaggcaagtca
vvi|miR-465|2mut|as|	aggagcctgtagccaagtca
vvi|miR-466|nat|as|	ccttggctcaattcgacccc
vvi|miR-466|2mut|as|	ccttgcctcaattagacccc

vvi-miR167d|+10_mismatch probe
vvi-miR167e|-10_perfect match probe
vvi-miR167e|-10_mismatch probe
vvi-miR167e|0_perfect match probe
vvi-miR167e|0_mismatch probe
vvi-miR167e|+10_perfect match probe
vvi-miR167e|+10_mismatch probe
vvi-miR168|-10_perfect match probe
vvi-miR168|-10_mismatch probe
vvi-miR168|0_perfect match probe
vvi-miR168|0_mismatch probe
vvi-miR168|+10_perfect match probe
vvi-miR168|+10_mismatch probe
vvi-miR169a|-10_perfect match probe
vvi-miR169a|-10_mismatch probe
vvi-miR169a|0_perfect match probe
vvi-miR169a|0_mismatch probe
vvi-miR169a|+10_perfect match probe
vvi-miR169a|+10_mismatch probe
vvi-miR169y|-10_perfect match probe
vvi-miR169y|-10_mismatch probe
vvi-miR169y|0_perfect match probe
vvi-miR169y|0_mismatch probe
vvi-miR169y|+10_perfect match probe
vvi-miR169y|+10_mismatch probe
vvi-miR169b|-10_perfect match probe
vvi-miR169b|-10_mismatch probe

vvi|miR-467|nat|as|	ggcaagccatccttggctca
vvi|miR-467|2mut|as|	ggcaagtcatccttgcctca
vvi|miR-468|nat|as|	tgcagatgacggcaagccat
vvi|miR-468|2mut|as|	tgcagatgaagccaagccat
vvi|miR-469|nat|as|	tccttggctgcattctactc
vvi|miR-469|2mut|as|	tccttgcctgaattctactc
vvi|miR-470|nat|as|	cggcaagtcatccttggctg
vvi|miR-470|2mut|as|	cggcaagccatccttcgctg
vvi|miR-471|nat|as|	gccccatctccggcaagtca
vvi|miR-471|2mut|as|	gcccaatctccgccaagtca
vvi|miR-472|nat|as|	ttcttggctgcattccactc
vvi|miR-472|2mut|as|	ttcttgcctgaattccactc
vvi|miR-473|nat|as|	cggcaaatcattcttggctg
vvi|miR-473|2mut|as|	cggcaaataattcttcgctg
vvi|miR-474|nat|as|	atataaattccggcaaatca
vvi|miR-474|2mut|as|	atataaatttcgccaaatca
vvi|miR-475|nat|as|	tccttggctatcagacaaga
vvi|miR-475|2mut|as|	tccttgcctatcagaaaaga
vvi|miR-476|nat|as|	aggcaagtcatccttggcta
vvi|miR-476|2mut|as|	aggcaagccatccttcgcta
vvi|miR-477|nat|as|	gggatagggcaggcaagtca
vvi|miR-477|2mut|as|	gggataggccagccaagtca
vvi|miR-478|nat|as|	tccttggctgcaccataata
vvi|miR-478|2mut|as|	tccttgcctgaaccataata
vvi|miR-479|nat|as|	cggcaagtcatccttggctg
vvi|miR-479|2mut|as|	cggcaagccatccttcgctg
vvi|miR-480|nat|as|	aaattattttcggcaagtca

vvi-miR169b|0_perfect match probe
vvi-miR169b|0_mismatch probe
vvi-miR169b|+10_perfect match probe
vvi-miR169b|+10_mismatch probe
vvi-miR169c|-10_perfect match probe
vvi-miR169c|-10_mismatch probe
vvi-miR169c|0_perfect match probe
vvi-miR169c|0_mismatch probe
vvi-miR169c|+10_perfect match probe
vvi-miR169c|+10_mismatch probe
vvi-miR169d|-10_perfect match probe
vvi-miR169d|-10_mismatch probe
vvi-miR169d|0_perfect match probe
vvi-miR169d|0_mismatch probe
vvi-miR169d|+10_perfect match probe
vvi-miR169d|+10_mismatch probe
vvi-miR169e|-10_perfect match probe
vvi-miR169e|-10_mismatch probe
vvi-miR169e|0_perfect match probe
vvi-miR169e|0_mismatch probe
vvi-miR169e|+10_perfect match probe
vvi-miR169e|+10_mismatch probe
vvi-miR169f|-10_perfect match probe
vvi-miR169f|-10_mismatch probe
vvi-miR169f|0_perfect match probe
vvi-miR169f|0_mismatch probe
vvi-miR169f|+10_perfect match

		probe
vvi|miR-480|2mut|as|	aaattatttccgccaagtca	vvi-miR169f|+10_mismatch probe
		vvi-miR169g|-10_perfect match
vvi|miR-481|nat|as|	tccttggctgcaccatactt	probe
vvi|miR-481|2mut|as|	tccttgcctgaaccatactt	vvi-miR169g|-10_mismatch probe
vvi|miR-482|nat|as|	cggcaagtcatccttggctg	vvi-miR169g|0_perfect match probe
vvi|miR-482|2mut|as|	cggcaagccatccttcgctg	vvi-miR169g|0_mismatch probe
		vvi-miR169g|+10_perfect match
vvi|miR-483|nat|as|	agtagagagtcggcaagtca	probe
vvi|miR-483|2mut|as|	agtagagagccgccaagtca	vvi-miR169g|+10_mismatch probe
		vvi-miR169h|-10_perfect match
vvi|miR-484|nat|as|	ccttggctcaattccaccct	probe
vvi|miR-484|2mut|as|	ccttgcctcaattcaaccct	vvi-miR169h|-10_mismatch probe
vvi|miR-485|nat|as|	ggcaagccatccttggctca	vvi-miR169h|0_perfect match probe
vvi|miR-485|2mut|as|	ggcaagtcatccttgcctca	vvi-miR169h|0_mismatch probe
		vvi-miR169h|+10_perfect match
vvi|miR-486|nat|as|	gacaaaggacggcaagccat	probe
vvi|miR-486|2mut|as|	gacaaaggaagccaagccat	vvi-miR169h|+10_mismatch probe
vvi|miR-487|nat|as|	tccttggctcaattccaccc	vvi-miR169i|-10_perfect match probe
vvi|miR-487|2mut|as|	tccttgcctcaattcaaccc	vvi-miR169i|-10_mismatch probe
vvi|miR-488|nat|as|	cggccagtcatccttggctc	vvi-miR169i|0_perfect match probe
vvi|miR-488|2mut|as|	cggcaagtcatccttcgctc	vvi-miR169i|0_mismatch probe
		vvi-miR169i|+10_perfect match
vvi|miR-489|nat|as|	tgaaaaatgacggccagtca	probe
vvi|miR-489|2mut|as|	tgaaaaatgccggtcagtca	vvi-miR169i|+10_mismatch probe
vvi|miR-490|nat|as|	tccttggctgcactccactc	vvi-miR169j|-10_perfect match probe
vvi|miR-490|2mut|as|	tccttgcctgaactccactc	vvi-miR169j|-10_mismatch probe
vvi|miR-491|nat|as|	cggcaagtcatccttggctg	vvi-miR169j|0_perfect match probe
vvi|miR-491|2mut|as|	cggcaagccatccttcgctg	vvi-miR169j|0_mismatch probe
		vvi-miR169j|+10_perfect match
vvi|miR-492|nat|as|	atgtgaattccggcaagtca	probe
vvi|miR-492|2mut|as|	atgtgaatttcgccaagtca	vvi-miR169j|+10_mismatch probe
		vvi-miR169k|-10_perfect match
vvi|miR-493|nat|as|	tccttggctgcactccactc	probe

vvi|miR-493|2mut|as|	tccttgcctgaactccactc
vvi|miR-494|nat|as|	cggcaagtcatccttggctg
vvi|miR-494|2mut|as|	cggcaagccatccttcgctg
vvi|miR-495|nat|as|	atgtgaattccggcaagtca
vvi|miR-495|2mut|as|	atgtgaatttcgccaagtca
vvi|miR-496|nat|as|	tccttggctcaattccaccc
vvi|miR-496|2mut|as|	tccttgcctcaattcaaccc
vvi|miR-497|nat|as|	cggcaagtcatccttggctc
vvi|miR-497|2mut|as|	cggcaagccatccttcgctc
vvi|miR-498|nat|as|	atgcaaaggacggcaagtca
vvi|miR-498|2mut|as|	atgcaaagaacgccaagtca
vvi|miR-499|nat|as|	tccttggctcacttccactc
vvi|miR-499|2mut|as|	tccttgcctcacttcaactc
vvi|miR-500|nat|as|	cggcaagtcatccttggctc
vvi|miR-500|2mut|as|	cggcaagccatccttcgctc
vvi|miR-501|nat|as|	ctgcagctgccggcaagtca
vvi|miR-501|2mut|as|	ctgcagctgtcgccaagtca
vvi|miR-502|nat|as|	tccttggctctattccactc
vvi|miR-502|2mut|as|	tccttgcctctattcaactc
vvi|miR-503|nat|as|	cggcaagtcatccttggctc
vvi|miR-503|2mut|as|	cggcaagccatccttcgctc
vvi|miR-504|nat|as|	ctgcagttgccggcaagtca
vvi|miR-504|2mut|as|	ctgcagttgtcgccaagtca
vvi|miR-505|nat|as|	tccttggctcaaatccaccc
vvi|miR-505|2mut|as|	tccttgcctcaaatcaaccc
vvi|miR-506|nat|as|	cggcaagtcatccttggctc
vvi|miR-506|2mut|as|	cggcaagccatccttcgctc

vvi-miR169k|-10_mismatch probe
vvi-miR169k|0_perfect match probe
vvi-miR169k|0_mismatch probe
vvi-miR169k|+10_perfect match probe
vvi-miR169k|+10_mismatch probe
vvi-miR169l|-10_perfect match probe
vvi-miR169l|-10_mismatch probe
vvi-miR169l|0_perfect match probe
vvi-miR169l|0_mismatch probe
vvi-miR169l|+10_perfect match probe
vvi-miR169l|+10_mismatch probe
vvi-miR169m|-10_perfect match probe
vvi-miR169m|-10_mismatch probe
vvi-miR169m|0_perfect match probe
vvi-miR169m|0_mismatch probe
vvi-miR169m|+10_perfect match probe
vvi-miR169m|+10_mismatch probe
vvi-miR169n|-10_perfect match probe
vvi-miR169n|-10_mismatch probe
vvi-miR169n|0_perfect match probe
vvi-miR169n|0_mismatch probe
vvi-miR169n|+10_perfect match probe
vvi-miR169n|+10_mismatch probe
vvi-miR169o|-10_perfect match probe
vvi-miR169o|-10_mismatch probe
vvi-miR169o|0_perfect match probe
vvi-miR169o|0_mismatch probe

<table>
<tr><td>vvi|miR-507|nat|as|</td><td>ctgctgatggcggcaagtca</td><td>vvi-miR169o|+10_perfect match probe</td></tr>
<tr><td>vvi|miR-507|2mut|as|</td><td>ctgctgatgcaggcaagtca</td><td>vvi-miR169o|+10_mismatch probe</td></tr>
<tr><td>vvi|miR-508|nat|as|</td><td>tccttggctcaattccacac</td><td>vvi-miR169p|-10_perfect match probe</td></tr>
<tr><td>vvi|miR-508|2mut|as|</td><td>tccttgcctcaattcaacac</td><td>vvi-miR169p|-10_mismatch probe</td></tr>
<tr><td>vvi|miR-509|nat|as|</td><td>cggcaagtcatccttggctc</td><td>vvi-miR169p|0_perfect match probe</td></tr>
<tr><td>vvi|miR-509|2mut|as|</td><td>cggcaagccatccttcgctc</td><td>vvi-miR169p|0_mismatch probe</td></tr>
<tr><td>vvi|miR-510|nat|as|</td><td>ctgcagctgccggcaagtca</td><td>vvi-miR169p|+10_perfect match probe</td></tr>
<tr><td>vvi|miR-510|2mut|as|</td><td>ctgcagctgtcgccaagtca</td><td>vvi-miR169p|+10_mismatch probe</td></tr>
<tr><td>vvi|miR-511|nat|as|</td><td>tccttggctctattccaccc</td><td>vvi-miR169q|-10_perfect match probe</td></tr>
<tr><td>vvi|miR-511|2mut|as|</td><td>tccttgcctctattcaaccc</td><td>vvi-miR169q|-10_mismatch probe</td></tr>
<tr><td>vvi|miR-512|nat|as|</td><td>cggcaagtcatccttggctc</td><td>vvi-miR169q|0_perfect match probe</td></tr>
<tr><td>vvi|miR-512|2mut|as|</td><td>cggcaagccatccttcgctc</td><td>vvi-miR169q|0_mismatch probe</td></tr>
<tr><td>vvi|miR-513|nat|as|</td><td>ctgcaaatgccggcaagtca</td><td>vvi-miR169q|+10_perfect match probe</td></tr>
<tr><td>vvi|miR-513|2mut|as|</td><td>ctgcaaatgtcgccaagtca</td><td>vvi-miR169q|+10_mismatch probe</td></tr>
<tr><td>vvi|miR-514|nat|as|</td><td>ccttgactcaattccaccct</td><td>vvi-miR169r|-10_perfect match probe</td></tr>
<tr><td>vvi|miR-514|2mut|as|</td><td>ccttgcctcaattcaaccct</td><td>vvi-miR169r|-10_mismatch probe</td></tr>
<tr><td>vvi|miR-515|nat|as|</td><td>ggcaagtcatccttgactca</td><td>vvi-miR169r|0_perfect match probe</td></tr>
<tr><td>vvi|miR-515|2mut|as|</td><td>ggcaagccattcttgactca</td><td>vvi-miR169r|0_mismatch probe</td></tr>
<tr><td>vvi|miR-516|nat|as|</td><td>aatatatatcggcaagtcat</td><td>vvi-miR169r|+10_perfect match probe</td></tr>
<tr><td>vvi|miR-516|2mut|as|</td><td>aatatatatagccaagtcat</td><td>vvi-miR169r|+10_mismatch probe</td></tr>
<tr><td>vvi|miR-517|nat|as|</td><td>tccttggctgcaatccactt</td><td>vvi-miR169s|-10_perfect match probe</td></tr>
<tr><td>vvi|miR-517|2mut|as|</td><td>tccttgcctgaaatccactt</td><td>vvi-miR169s|-10_mismatch probe</td></tr>
<tr><td>vvi|miR-518|nat|as|</td><td>cggcaagtcatccttggctg</td><td>vvi-miR169s|0_perfect match probe</td></tr>
<tr><td>vvi|miR-518|2mut|as|</td><td>cggcaagccatccttcgctg</td><td>vvi-miR169s|0_mismatch probe</td></tr>
<tr><td>vvi|miR-519|nat|as|</td><td>tgccaagtgccggcaagtca</td><td>vvi-miR169s|+10_perfect match probe</td></tr>
<tr><td>vvi|miR-519|2mut|as|</td><td>tgccaagtgtcgccaagtca</td><td>vvi-miR169s|+10_mismatch probe</td></tr>
<tr><td>vvi|miR-520|nat|as|</td><td>ccttgactcgattccaccct</td><td>vvi-miR169t|-10_perfect match probe</td></tr>
</table>

vvi|miR-520|2mut|as| ccttgactagattcaaccct vvi-miR169t|-10_mismatch probe
vvi|miR-521|nat|as| ggcaagtcatccttgactcg vvi-miR169t|0_perfect match probe
vvi|miR-521|2mut|as| ggcaagccattcttgactcg vvi-miR169t|0_mismatch probe
vvi-miR169t|+10_perfect match
vvi|miR-522|nat|as| aatatatatcggcaagtcat probe
vvi|miR-522|2mut|as| aatatatatagccaagtcat vvi-miR169t|+10_mismatch probe
vvi-miR169u|-10_perfect match
vvi|miR-523|nat|as| ccttgactcaattccaccct probe
vvi|miR-523|2mut|as| ccttgcctcaattcaaccct vvi-miR169u|-10_mismatch probe
vvi|miR-524|nat|as| ggcaagtcatccttgactca vvi-miR169u|0_perfect match probe
vvi|miR-524|2mut|as| ggcaagccattcttgactca vvi-miR169u|0_mismatch probe
vvi-miR169u|+10_perfect match
vvi|miR-525|nat|as| aatatataacggcaagtcat probe
vvi|miR-525|2mut|as| aatatataaagccaagtcat vvi-miR169u|+10_mismatch probe
vvi-miR169v|-10_perfect match
vvi|miR-526|nat|as| tccttggctttgttcctccc probe
vvi|miR-526|2mut|as| tccttgcctttgtttctccc vvi-miR169v|-10_mismatch probe
vvi|miR-527|nat|as| cggcaattcatccttggctt vvi-miR169v|0_perfect match probe
vvi|miR-527|2mut|as| cggcaattaatccttcgctt vvi-miR169v|0_mismatch probe
vvi-miR169v|+10_perfect match
vvi|miR-528|nat|as| ctttcatcgccggcaattca probe
vvi|miR-528|2mut|as| ctttcatccccgccaattca vvi-miR169v|+10_mismatch probe
vvi-miR169w|-10_perfect match
vvi|miR-529|nat|as| tccttggctgcaccacataa probe
vvi|miR-529|2mut|as| tccttgcctgaaccacataa vvi-miR169w|-10_mismatch probe
vvi|miR-530|nat|as| cggcaagtcatccttggctg vvi-miR169w|0_perfect match probe
vvi|miR-530|2mut|as| cggcaagccatccttcgctg vvi-miR169w|0_mismatch probe
vvi-miR169w|+10_perfect match
vvi|miR-531|nat|as| agggagttgccggcaagtca probe
vvi|miR-531|2mut|as| agggagttgtcgccaagtca vvi-miR169w|+10_mismatch probe
vvi-miR169x|-10_perfect match
vvi|miR-532|nat|as| tccttggctaccagacgaga probe
vvi|miR-532|2mut|as| tccttgcctaccagaagaga vvi-miR169x|-10_mismatch probe
vvi|miR-533|nat|as| aggcaagtcatccttggcta vvi-miR169x|0_perfect match probe

vvi|miR-533|2mut|as|	aggcaagccatccttcgcta
vvi|miR-534|nat|as|	tggtggatttaggcaagtca
vvi|miR-534|2mut|as|	tggtcgatttagccaagtca
vvi|miR-535|nat|as|	gacggctcaatcaaaccaaa
vvi|miR-535|2mut|as|	gacgcctcaatcaaatcaaa
vvi|miR-536|nat|as|	gtgatgttgggacggctcaa
vvi|miR-536|2mut|as|	gtgatgttgtgacgcctcaa
vvi|miR-537|nat|as|	ggtgggaagcgtgatgttgg
vvi|miR-537|2mut|as|	ggtgggaagactgatgttgg
vvi|miR-538|nat|as|	ggctcaatcaaatcacatgg
vvi|miR-538|2mut|as|	ggctcaataaaatcccatgg
vvi|miR-539|nat|as|	atattgacgcggctcaatca
vvi|miR-539|2mut|as|	atattgacgagcctcaatca
vvi|miR-540|nat|as|	gcaaaaggagatattgacgc
vvi|miR-540|2mut|as|	gcaaaagaagatattaacgc
vvi|miR-541|nat|as|	cacggctcaatcaaaaacca
vvi|miR-541|2mut|as|	cacgcctcaataaaaaacca
vvi|miR-542|nat|as|	gtgatattggcacggctcaa
vvi|miR-542|2mut|as|	gtgatattgccacgcctcaa
vvi|miR-543|nat|as|	gcaatgacacgtgatattgg
vvi|miR-543|2mut|as|	gcaatgacaactgatattgg
vvi|miR-544|nat|as|	cacggctcaatcaaacaaca
vvi|miR-544|2mut|as|	cacgcctcaatcaaaaaaca
vvi|miR-545|nat|as|	gtgatattggcacggctcaa
vvi|miR-545|2mut|as|	gtgatattgccacgcctcaa
vvi|miR-546|nat|as|	gaagtaggacgtgatattgg

vvi-miR169x|0_mismatch probe
vvi-miR169x|+10_perfect match probe
vvi-miR169x|+10_mismatch probe
vvi-miR171a|-10_perfect match probe
vvi-miR171a|-10_mismatch probe
vvi-miR171a|0_perfect match probe
vvi-miR171a|0_mismatch probe
vvi-miR171a|+10_perfect match probe
vvi-miR171a|+10_mismatch probe
vvi-miR171b|-10_perfect match probe
vvi-miR171b|-10_mismatch probe
vvi-miR171b|0_perfect match probe
vvi-miR171b|0_mismatch probe
vvi-miR171b|+10_perfect match probe
vvi-miR171b|+10_mismatch probe
vvi-miR171c|-10_perfect match probe
vvi-miR171c|-10_mismatch probe
vvi-miR171c|0_perfect match probe
vvi-miR171c|0_mismatch probe
vvi-miR171c|+10_perfect match probe
vvi-miR171c|+10_mismatch probe
vvi-miR171d|-10_perfect match probe
vvi-miR171d|-10_mismatch probe
vvi-miR171d|0_perfect match probe
vvi-miR171d|0_mismatch probe
vvi-miR171d|+10_perfect match

		probe
vvi|miR-546|2mut|as|	gaagtaggaactgatattgg	vvi-miR171d|+10_mismatch probe
vvi|miR-547|nat|as|	ggctcaatcagagatcgttc	vvi-miR171e|-10_perfect match probe
vvi|miR-547|2mut|as|	ggctcaatccgagatagttc	vvi-miR171e|-10_mismatch probe
vvi|miR-548|nat|as|	atattggcgcggctcaatca	vvi-miR171e|0_perfect match probe
vvi|miR-548|2mut|as|	atattggcgagcctcaatca	vvi-miR171e|0_mismatch probe
vvi|miR-549|nat|as|	gcagaaagtgatattggcgc	vvi-miR171e|+10_perfect match probe
vvi|miR-549|2mut|as|	gcagaaagcgatattcgcgc	vvi-miR171e|+10_mismatch probe
vvi|miR-550|nat|as|	cgcggctcaatctgagatca	vvi-miR171f|-10_perfect match probe
vvi|miR-550|2mut|as|	cgcgcctcaatccgagatca	vvi-miR171f|-10_mismatch probe
vvi|miR-551|nat|as|	gtgatattggcgcggctcaa	vvi-miR171f|0_perfect match probe
vvi|miR-551|2mut|as|	gtgatattgccgaggctcaa	vvi-miR171f|0_mismatch probe
vvi|miR-552|nat|as|	ccacagaaaagtgatattgg	vvi-miR171f|+10_perfect match probe
vvi|miR-552|2mut|as|	ccaccgaaaagcgatattgg	vvi-miR171f|+10_mismatch probe
vvi|miR-553|nat|as|	ttcggctcaaggagttggtg	vvi-miR171g|-10_perfect match probe
vvi|miR-553|2mut|as|	ttcgcctcaagaagttggtg	vvi-miR171g|-10_mismatch probe
vvi|miR-554|nat|as|	gtgatattggttcggctcaa	vvi-miR171g|0_perfect match probe
vvi|miR-554|2mut|as|	gtgatattcgttcgcctcaa	vvi-miR171g|0_mismatch probe
vvi|miR-555|nat|as|	tttggctcgggtgatattgg	vvi-miR171g|+10_perfect match probe
vvi|miR-555|2mut|as|	tttgcctcggctgatattgg	vvi-miR171g|+10_mismatch probe
vvi|miR-556|nat|as|	ggctcaaccaaacatcagag	vvi-miR171h|-10_perfect match probe
vvi|miR-556|2mut|as|	ggctcaacaaaaaatcagag	vvi-miR171h|-10_mismatch probe
vvi|miR-557|nat|as|	atattggcgcggctcaacca	vvi-miR171h|0_perfect match probe
vvi|miR-557|2mut|as|	atattggcgagcctcaacca	vvi-miR171h|0_mismatch probe
vvi|miR-558|nat|as|	gatagacgggatattggcgc	vvi-miR171h|+10_perfect match probe
vvi|miR-558|2mut|as|	gatagacaggatattcgcgc	vvi-miR171h|+10_mismatch probe
vvi|miR-559|nat|as|	cacggctcaatcagattaaa	vvi-miR171i|-10_perfect match probe

vvi|miR-559|2mut|as|	cacgcctcaatccgattaaa	vvi-miR171i|-10_mismatch probe
vvi|miR-560|nat|as|	atgatattggcacggctcaa	vvi-miR171i|0_perfect match probe
vvi|miR-560|2mut|as|	atgatattgccacgcctcaa	vvi-miR171i|0_mismatch probe
vvi|miR-561|nat|as|	agtgaacatgatgatattgg	vvi-miR171i|+10_perfect match probe
vvi|miR-561|2mut|as|	agtgaaaatgataatattgg	vvi-miR171i|+10_mismatch probe
vvi|miR-562|nat|as|	tcaagattcacatgcaaatg	vvi-miR172a|-10_perfect match probe
vvi|miR-562|2mut|as|	tcaagattcccatgaaaatg	vvi-miR172a|-10_mismatch probe
vvi|miR-563|nat|as|	tgtagcatcatcaagattca	vvi-miR172a|0_perfect match probe
vvi|miR-563|2mut|as|	tgtagaatcataaagattca	vvi-miR172a|0_mismatch probe
vvi|miR-564|nat|as|	tgtttgcgcatgtagcatca	vvi-miR172a|+10_perfect match probe
vvi|miR-564|2mut|as|	tgtttgcccatgtagaatca	vvi-miR172a|+10_mismatch probe
vvi|miR-565|nat|as|	tcaagattcacatgcgaaag	vvi-miR172b|-10_perfect match probe
vvi|miR-565|2mut|as|	tcaagattcccatgagaaag	vvi-miR172b|-10_mismatch probe
vvi|miR-566|nat|as|	tgtagcatcatcaagattca	vvi-miR172b|0_perfect match probe
vvi|miR-566|2mut|as|	tgtagaatcataaagattca	vvi-miR172b|0_mismatch probe
vvi|miR-567|nat|as|	tgtttgcaggtgtagcatca	vvi-miR172b|+10_perfect match probe
vvi|miR-567|2mut|as|	tgtttgaaggtgtagaatca	vvi-miR172b|+10_mismatch probe
vvi|miR-568|nat|as|	tcaagattcccacatcaaag	vvi-miR172c|-10_perfect match probe
vvi|miR-568|2mut|as|	tcaagattctaacatcaaag	vvi-miR172c|-10_mismatch probe
vvi|miR-569|nat|as|	tgcagcatcatcaagattcc	vvi-miR172c|0_perfect match probe
vvi|miR-569|2mut|as|	tgcagaatcataaagattcc	vvi-miR172c|0_mismatch probe
vvi|miR-570|nat|as|	ttattgccgctgcagcatca	vvi-miR172c|+10_perfect match probe
vvi|miR-570|2mut|as|	ttattgcccctgcagaatca	vvi-miR172c|+10_mismatch probe
vvi|miR-571|nat|as|	tcaagattctcatctcaaaa	vvi-miR172d|-10_perfect match probe
vvi|miR-571|2mut|as|	tcaagattcccatctaaaaa	vvi-miR172d|-10_mismatch probe
vvi|miR-572|nat|as|	tgcagcatcatcaagattct	vvi-miR172d|0_perfect match probe

vvi|miR-572|2mut|as|	tgcagaatcataaagattct
vvi|miR-573|nat|as|	tcactgcgaatgcagcatca
vvi|miR-573|2mut|as|	tcactgagaatgcagaatca
vvi|miR-574|nat|as|	tcagtccaagacgtgacagc
vvi|miR-574|2mut|as|	tcagtcaaagacctgacagc
vvi|miR-575|nat|as|	ggagctcccttcagtccaag
vvi|miR-575|2mut|as|	ggagctctcttcagttcaag
vvi|miR-576|nat|as|	ggaatacaagggagctccct
vvi|miR-576|2mut|as|	ggaatacaactgagctccct
vvi|miR-577|nat|as|	tcagtccaagcacagggagc
vvi|miR-577|2mut|as|	tcagtccaaccacagtgagc
vvi|miR-578|nat|as|	ggagctcccttcagtccaag
vvi|miR-578|2mut|as|	ggagctctcttcagttcaag
vvi|miR-579|nat|as|	tgcagtgaagggagctccct
vvi|miR-579|2mut|as|	tgcagtgaactgagctccct
vvi|miR-580|nat|as|	tcagtccaaaacaaggagca
vvi|miR-580|2mut|as|	tcagtcaaaaacaagaagca
vvi|miR-581|nat|as|	ggagctcccttcagtccaaa
vvi|miR-581|2mut|as|	ggagctctcttcagttcaaa
vvi|miR-582|nat|as|	agaagaactaggagctccct
vvi|miR-582|2mut|as|	agaagaattaggagttccct
vvi|miR-583|nat|as|	tcagtccaagcacagacaag
vvi|miR-583|2mut|as|	tcagtccaacaacagacaag
vvi|miR-584|nat|as|	ggagctcccttcagtccaag
vvi|miR-584|2mut|as|	ggagctctcttcagttcaag
vvi|miR-585|nat|as|	acagtagaagggagctccct

vvi-miR172d|0_mismatch probe
vvi-miR172d|+10_perfect match probe
vvi-miR172d|+10_mismatch probe
vvi-miR319b|-10_perfect match probe
vvi-miR319b|-10_mismatch probe
vvi-miR319b|0_perfect match probe
vvi-miR319b|0_mismatch probe
vvi-miR319b|+10_perfect match probe
vvi-miR319b|+10_mismatch probe
vvi-miR319c|-10_perfect match probe
vvi-miR319c|-10_mismatch probe
vvi-miR319c|0_perfect match probe
vvi-miR319c|0_mismatch probe
vvi-miR319c|+10_perfect match probe
vvi-miR319c|+10_mismatch probe
vvi-miR319e|-10_perfect match probe
vvi-miR319e|-10_mismatch probe
vvi-miR319e|0_perfect match probe
vvi-miR319e|0_mismatch probe
vvi-miR319e|+10_perfect match probe
vvi-miR319e|+10_mismatch probe
vvi-miR319f|-10_perfect match probe
vvi-miR319f|-10_mismatch probe
vvi-miR319f|0_perfect match probe
vvi-miR319f|0_mismatch probe
vvi-miR319f|+10_perfect match probe

vvi|miR-585|2mut|as|	acagtagaactgagctccct
vvi|miR-586|nat|as|	tcagtccaatgagtgggcaa
vvi|miR-586|2mut|as|	tcagtcaaatgagtgtgcaa
vvi|miR-587|nat|as|	ggagctcccttcagtccaat
vvi|miR-587|2mut|as|	ggagctctcttcagttcaat
vvi|miR-588|nat|as|	atctgccctgggagctccct
vvi|miR-588|2mut|as|	atctgtcctgtgagctccct
vvi|miR-589|nat|as|	tcctgagcttaacagattct
vvi|miR-589|2mut|as|	tcctgagtttaaaagattct
vvi|miR-590|nat|as|	gcgctatccctcctgagctt
vvi|miR-590|2mut|as|	gcgctatctcttctgagctt
vvi|miR-591|nat|as|	atggctcatggcgctatccc
vvi|miR-591|2mut|as|	atggttcatggccctatccc
vvi|miR-592|nat|as|	tccctttggaactgtccacc
vvi|miR-592|2mut|as|	tccctttcgaactgttcacc
vvi|miR-593|nat|as|	atcaatgcgatccctttgga
vvi|miR-593|2mut|as|	atcaatgagatctctttgga
vvi|miR-594|nat|as|	taaccatgggatcaatgcga
vvi|miR-594|2mut|as|	taaccatgtgataaatgcga
vvi|miR-595|nat|as|	tccctttggatgcctcctcc
vvi|miR-595|2mut|as|	tccctttcgatgtctcctcc
vvi|miR-596|nat|as|	atcaatgcgatccctttgga
vvi|miR-596|2mut|as|	atcaatgagatctctttgga
vvi|miR-597|nat|as|	agagtttgggatcaatgcga
vvi|miR-597|2mut|as|	agagtttgtgataaatgcga
vvi|miR-598|nat|as|	agaatgccaaaatggctctg
vvi|miR-598|2mut|as|	agaatgtcaaaatgcctctg

vvi-miR319f|+10_mismatch probe
vvi-miR319g|-10_perfect match probe
vvi-miR319g|-10_mismatch probe
vvi-miR319g|0_perfect match probe
vvi-miR319g|0_mismatch probe
vvi-miR319g|+10_perfect match probe
vvi-miR319g|+10_mismatch probe
vvi-miR390|-10_perfect match probe
vvi-miR390|-10_mismatch probe
vvi-miR390|0_perfect match probe
vvi-miR390|0_mismatch probe
vvi-miR390|+10_perfect match probe
vvi-miR390|+10_mismatch probe
vvi-miR393a|-10_perfect match probe
vvi-miR393a|-10_mismatch probe
vvi-miR393a|0_perfect match probe
vvi-miR393a|0_mismatch probe
vvi-miR393a|+10_perfect match probe
vvi-miR393a|+10_mismatch probe
vvi-miR393b|-10_perfect match probe
vvi-miR393b|-10_mismatch probe
vvi-miR393b|0_perfect match probe
vvi-miR393b|0_mismatch probe
vvi-miR393b|+10_perfect match probe
vvi-miR393b|+10_mismatch probe
vvi-miR394a|-10_perfect match probe
vvi-miR394a|-10_mismatch probe

vvi|miR-599|nat|as|	tgatgtgcatggaggtggac	vvi-miR394a|+10_perfect match probe
vvi|miR-599|2mut|as|	tgatgtgaatcgaggtggac	vvi-miR394a|+10_mismatch probe
vvi|miR-600|nat|as|	agaatgccaataaactctgt	vvi-miR394b|-10_perfect match probe
vvi|miR-600|2mut|as|	agaatgtcaataaattctgt	vvi-miR394b|-10_mismatch probe
vvi|miR-601|nat|as|	tcaagagatgggaggtggac	vvi-miR394b|+10_perfect match probe
vvi|miR-601|2mut|as|	tcaagagatctgaggtggac	vvi-miR394b|+10_mismatch probe
vvi|miR-602|nat|as|	agaatgccaaaatggctctg	vvi-miR394c|-10_perfect match probe
vvi|miR-602|2mut|as|	agaatgtcaaaatgcctctg	vvi-miR394c|-10_mismatch probe
vvi|miR-603|nat|as|	tggtatatatggaggtggac	vvi-miR394c|+10_perfect match probe
vvi|miR-603|2mut|as|	tggtatataccgaggtggac	vvi-miR394c|+10_mismatch probe
vvi|miR-604|nat|as|	aacacttcagtaggaagtca	vvi-miR395a|-10_perfect match probe
vvi|miR-604|2mut|as|	aacacttccgtagaaagtca	vvi-miR395a|-10_mismatch probe
vvi|miR-605|nat|as|	agttcccccaaacacttcag	vvi-miR395a|0_perfect match probe
vvi|miR-605|2mut|as|	agttccctcaaacccttcag	vvi-miR395a|0_mismatch probe
vvi|miR-606|nat|as|	cggtaccaggagttccccca	vvi-miR395a|+10_perfect match probe
vvi|miR-606|2mut|as|	cggtacaaggagttctccca	vvi-miR395a|+10_mismatch probe
vvi|miR-607|nat|as|	aacacttcagtacgtaggca	vvi-miR395b|-10_perfect match probe
vvi|miR-607|2mut|as|	aacacttccgtacctaggca	vvi-miR395b|-10_mismatch probe
vvi|miR-608|nat|as|	agttcccccaaacacttcag	vvi-miR395b|0_perfect match probe
vvi|miR-608|2mut|as|	agttccctcaaacccttcag	vvi-miR395b|0_mismatch probe
vvi|miR-609|nat|as|	tggcaccaggagttccccca	vvi-miR395b|+10_perfect match probe
vvi|miR-609|2mut|as|	tggcacaaggagttctccca	vvi-miR395b|+10_mismatch probe
vvi|miR-610|nat|as|	aacacttcagtaggaagtca	vvi-miR395c|-10_perfect match probe
vvi|miR-610|2mut|as|	aacacttccgtagaaagtca	vvi-miR395c|-10_mismatch probe

<table>
<tr><td>vvi|miR-611|nat|as|</td><td>agttcccccaaacacttcag</td><td>vvi-miR395c|0_perfect match probe</td></tr>
<tr><td>vvi|miR-611|2mut|as|</td><td>agttccctcaaacccttcag</td><td>vvi-miR395c|0_mismatch probe</td></tr>
<tr><td></td><td></td><td>vvi-miR395c|+10_perfect match</td></tr>
<tr><td>vvi|miR-612|nat|as|</td><td>tggcaccaggagttccccca</td><td>probe</td></tr>
<tr><td>vvi|miR-612|2mut|as|</td><td>tggcacaaggagttctccca</td><td>vvi-miR395c|+10_mismatch probe</td></tr>
<tr><td></td><td></td><td>vvi-miR395d|-10_perfect match</td></tr>
<tr><td>vvi|miR-613|nat|as|</td><td>aacacttcagtaggaagtca</td><td>probe</td></tr>
<tr><td>vvi|miR-613|2mut|as|</td><td>aacacttccgtagaaagtca</td><td>vvi-miR395d|-10_mismatch probe</td></tr>
<tr><td>vvi|miR-614|nat|as|</td><td>agttcccccaaacacttcag</td><td>vvi-miR395d|0_perfect match probe</td></tr>
<tr><td>vvi|miR-614|2mut|as|</td><td>agttccctcaaacccttcag</td><td>vvi-miR395d|0_mismatch probe</td></tr>
<tr><td></td><td></td><td>vvi-miR395d|+10_perfect match</td></tr>
<tr><td>vvi|miR-615|nat|as|</td><td>tgacaccaggagttccccca</td><td>probe</td></tr>
<tr><td>vvi|miR-615|2mut|as|</td><td>tgacacaaggagttctccca</td><td>vvi-miR395d|+10_mismatch probe</td></tr>
<tr><td></td><td></td><td>vvi-miR395e|-10_perfect match</td></tr>
<tr><td>vvi|miR-616|nat|as|</td><td>aacacttcagtaggaagtca</td><td>probe</td></tr>
<tr><td>vvi|miR-616|2mut|as|</td><td>aacacttccgtagaaagtca</td><td>vvi-miR395e|-10_mismatch probe</td></tr>
<tr><td>vvi|miR-617|nat|as|</td><td>agttcccccaaacacttcag</td><td>vvi-miR395e|0_perfect match probe</td></tr>
<tr><td>vvi|miR-617|2mut|as|</td><td>agttccctcaaacccttcag</td><td>vvi-miR395e|0_mismatch probe</td></tr>
<tr><td></td><td></td><td>vvi-miR395e|+10_perfect match</td></tr>
<tr><td>vvi|miR-618|nat|as|</td><td>tggcaccaggagttccccca</td><td>probe</td></tr>
<tr><td>vvi|miR-618|2mut|as|</td><td>tggcacaaggagttctccca</td><td>vvi-miR395e|+10_mismatch probe</td></tr>
<tr><td>vvi|miR-619|nat|as|</td><td>aacacttcagtgggaagtca</td><td>vvi-miR395f|-10_perfect match probe</td></tr>
<tr><td>vvi|miR-619|2mut|as|</td><td>aacacttccgtgtgaagtca</td><td>vvi-miR395f|-10_mismatch probe</td></tr>
<tr><td>vvi|miR-620|nat|as|</td><td>agttcccccaaacacttcag</td><td>vvi-miR395f|0_perfect match probe</td></tr>
<tr><td>vvi|miR-620|2mut|as|</td><td>agttccctcaaacccttcag</td><td>vvi-miR395f|0_mismatch probe</td></tr>
<tr><td></td><td></td><td>vvi-miR395f|+10_perfect match</td></tr>
<tr><td>vvi|miR-621|nat|as|</td><td>tgacaccaggagttccccca</td><td>probe</td></tr>
<tr><td>vvi|miR-621|2mut|as|</td><td>tgacacaaggagttctccca</td><td>vvi-miR395f|+10_mismatch probe</td></tr>
<tr><td></td><td></td><td>vvi-miR395g|-10_perfect match</td></tr>
<tr><td>vvi|miR-622|nat|as|</td><td>aacacttcagtaggaattga</td><td>probe</td></tr>
<tr><td>vvi|miR-622|2mut|as|</td><td>aacacttccgtagaaattga</td><td>vvi-miR395g|-10_mismatch probe</td></tr>
<tr><td>vvi|miR-623|nat|as|</td><td>agttcccccaaacacttcag</td><td>vvi-miR395g|0_perfect match probe</td></tr>
<tr><td>vvi|miR-623|2mut|as|</td><td>agttccctcaaacccttcag</td><td>vvi-miR395g|0_mismatch probe</td></tr>
<tr><td>vvi|miR-624|nat|as|</td><td>tgacaccgggagttccccca</td><td>vvi-miR395g|+10_perfect match</td></tr>
</table>

		probe
vvi|miR-624|2mut|as|	tgacaccaggagttctccca	vvi-miR395g|+10_mismatch probe
		vvi-miR395h|-10_perfect match
vvi|miR-625|nat|as|	aacacttcagtaggaagtca	probe
vvi|miR-625|2mut|as|	aacacttccgtagaaagtca	vvi-miR395h|-10_mismatch probe
vvi|miR-626|nat|as|	agttcccccaaacacttcag	vvi-miR395h|0_perfect match probe
vvi|miR-626|2mut|as|	agttccctcaaacccttcag	vvi-miR395h|0_mismatch probe
		vvi-miR395h|+10_perfect match
vvi|miR-627|nat|as|	tagcaccaggagttcccccca	probe
vvi|miR-627|2mut|as|	tagcacaaggagttctccca	vvi-miR395h|+10_mismatch probe
vvi|miR-628|nat|as|	aacacttcagtaggaagtca	vvi-miR395i|-10_perfect match probe
vvi|miR-628|2mut|as|	aacacttccgtagaaagtca	vvi-miR395i|-10_mismatch probe
vvi|miR-629|nat|as|	agttcccccaaacacttcag	vvi-miR395i|0_perfect match probe
vvi|miR-629|2mut|as|	agttccctcaaacccttcag	vvi-miR395i|0_mismatch probe
		vvi-miR395i|+10_perfect match
vvi|miR-630|nat|as|	tgacaccaggagttcccccca	probe
vvi|miR-630|2mut|as|	tgacacaaggagttctccca	vvi-miR395i|+10_mismatch probe
vvi|miR-631|nat|as|	aacacttcagtaggaagtca	vvi-miR395j|-10_perfect match probe
vvi|miR-631|2mut|as|	aacacttccgtagaaagtca	vvi-miR395j|-10_mismatch probe
vvi|miR-632|nat|as|	agttcccccaaacacttcag	vvi-miR395j|0_perfect match probe
vvi|miR-632|2mut|as|	agttccctcaaacccttcag	vvi-miR395j|0_mismatch probe
		vvi-miR395j|+10_perfect match
vvi|miR-633|nat|as|	tgacaccaggagttcccccca	probe
vvi|miR-633|2mut|as|	tgacacaaggagttctccca	vvi-miR395j|+10_mismatch probe
		vvi-miR395k|-10_perfect match
vvi|miR-634|nat|as|	aacacttcagtaggaagtca	probe
vvi|miR-634|2mut|as|	aacacttccgtagaaagtca	vvi-miR395k|-10_mismatch probe
vvi|miR-635|nat|as|	agttcccccaaacacttcag	vvi-miR395k|0_perfect match probe
vvi|miR-635|2mut|as|	agttccctcaaacccttcag	vvi-miR395k|0_mismatch probe
		vvi-miR395k|+10_perfect match
vvi|miR-636|nat|as|	tggcaccaggagttcccccca	probe
vvi|miR-636|2mut|as|	tggcacaaggagttctccca	vvi-miR395k|+10_mismatch probe
vvi|miR-637|nat|as|	aacacttcagtaggaagtca	vvi-miR395l|-10_perfect match probe
vvi|miR-637|2mut|as|	aacacttccgtagaaagtca	vvi-miR395l|-10_mismatch probe

vvi|miR-638|nat|as|	agttcccccaaacacttcag	vvi-miR395l|0_perfect match probe
vvi|miR-638|2mut|as|	agttccctcaaacccttcag	vvi-miR395l|0_mismatch probe
		vvi-miR395l|+10_perfect match
vvi|miR-639|nat|as|	tgacaccaggagttccccca	probe
vvi|miR-639|2mut|as|	tgacacaaggagttctccca	vvi-miR395l|+10_mismatch probe
		vvi-miR395m|-10_perfect match
vvi|miR-640|nat|as|	aacacttcagtaggaagtca	probe
vvi|miR-640|2mut|as|	aacacttccgtagaaagtca	vvi-miR395m|-10_mismatch probe
vvi|miR-641|nat|as|	agttcccccaaacacttcag	vvi-miR395m|0_perfect match probe
vvi|miR-641|2mut|as|	agttccctcaaacccttcag	vvi-miR395m|0_mismatch probe
		vvi-miR395m|+10_perfect match
vvi|miR-642|nat|as|	tggcaccaggagttccccca	probe
vvi|miR-642|2mut|as|	tggcacaaggagttctccca	vvi-miR395m|+10_mismatch probe
		vvi-miR395n|-10_perfect match
vvi|miR-643|nat|as|	gactcttcagtagaggaaat	probe
vvi|miR-643|2mut|as|	gactcttccgtagagaaaat	vvi-miR395n|-10_mismatch probe
vvi|miR-644|nat|as|	agttcctccagactcttcag	vvi-miR395n|0_perfect match probe
vvi|miR-644|2mut|as|	agttcctcaagcctcttcag	vvi-miR395n|0_mismatch probe
		vvi-miR395n|+10_perfect match
vvi|miR-645|nat|as|	tggcaccaagagttcctcca	probe
vvi|miR-645|2mut|as|	tggcacaaagagtttctcca	vvi-miR395n|+10_mismatch probe
		vvi-miR396a|-10_perfect match
vvi|miR-646|nat|as|	gctgtgggaggacatggcaa	probe
vvi|miR-646|2mut|as|	gctgtgtgaggacatcgcaa	vvi-miR396a|-10_mismatch probe
vvi|miR-647|nat|as|	gctcaagaaagctgtgggag	vvi-miR396a|0_perfect match probe
vvi|miR-647|2mut|as|	gctcaacaaagttgtgggag	vvi-miR396a|0_mismatch probe
		vvi-miR396a|+10_perfect match
vvi|miR-648|nat|as|	tgaaatagaagctcaagaaa	probe
vvi|miR-648|2mut|as|	tgaaatacaagttcaagaaa	vvi-miR396a|+10_mismatch probe
		vvi-miR396b|-10_perfect match
vvi|miR-649|nat|as|	gctgtgggaaaccatggctt	probe
vvi|miR-649|2mut|as|	gctgtgtgaaaccatcgctt	vvi-miR396b|-10_mismatch probe
vvi|miR-650|nat|as|	gttcaagaaagctgtgggaa	vvi-miR396b|0_perfect match probe
vvi|miR-650|2mut|as|	gttcaacaaagttgtgggaa	vvi-miR396b|0_mismatch probe

vvi|miR-651|nat|as|	atccactgaagttcaagaaa	vvi-miR396b|+10_perfect match probe
vvi|miR-651|2mut|as|	atcccctgaagttaaagaaa	vvi-miR396b|+10_mismatch probe
vvi|miR-652|nat|as|	agctgtggaagatcacagaa	vvi-miR396c|-10_perfect match probe
vvi|miR-652|2mut|as|	agctgtcgaagatcccagaa	vvi-miR396c|-10_mismatch probe
vvi|miR-653|nat|as|	agttcaagaaagctgtggaa	vvi-miR396c|0_perfect match probe
vvi|miR-653|2mut|as|	agttaaagaaagttgtggaa	vvi-miR396c|0_mismatch probe
vvi|miR-654|nat|as|	cttccaaaacagttcaagaa	vvi-miR396c|+10_perfect match probe
vvi|miR-654|2mut|as|	cttcaaaaaccgttcaagaa	vvi-miR396c|+10_mismatch probe
vvi|miR-655|nat|as|	agctgtggaagaatacaaag	vvi-miR396d|-10_perfect match probe
vvi|miR-655|2mut|as|	agctgtcgaagaataaaaag	vvi-miR396d|-10_mismatch probe
vvi|miR-656|nat|as|	agttcaagaaagctgtggaa	vvi-miR396d|0_perfect match probe
vvi|miR-656|2mut|as|	agttaaagaaagttgtggaa	vvi-miR396d|0_mismatch probe
vvi|miR-657|nat|as|	tgaaaaatgcagttcaagaa	vvi-miR396d|+10_perfect match probe
vvi|miR-657|2mut|as|	tgaaaaatgacgttcaagaa	vvi-miR396d|+10_mismatch probe
vvi|miR-658|nat|as|	cactcaatgatgttttcttc	vvi-miR397a|-10_perfect match probe
vvi|miR-658|2mut|as|	cactcaataatcttttcttc	vvi-miR397a|-10_mismatch probe
vvi|miR-659|nat|as|	atcaacgctgcactcaatga	vvi-miR397a|0_perfect match probe
vvi|miR-659|2mut|as|	atcaaccctgaactcaatga	vvi-miR397a|0_mismatch probe
vvi|miR-660|nat|as|	cttcagtttcatcaacgctg	vvi-miR397a|+10_perfect match probe
vvi|miR-660|2mut|as|	cttccgtttcatcaaagctg	vvi-miR397a|+10_mismatch probe
vvi|miR-661|nat|as|	cactcaatgatgttttcttc	vvi-miR397b|-10_perfect match probe
vvi|miR-661|2mut|as|	cactcaataatcttttcttc	vvi-miR397b|-10_mismatch probe
vvi|miR-662|nat|as|	atcaacgctgcactcaatga	vvi-miR397b|0_perfect match probe
vvi|miR-662|2mut|as|	atcaaccctgaactcaatga	vvi-miR397b|0_mismatch probe
vvi|miR-663|nat|as|	cttcagtttcatcaacgctg	vvi-miR397b|+10_perfect match probe

vvi|miR-663|2mut|as|	cttccgtttcatcaaagctg	vvi-miR397b|+10_mismatch probe
vvi|miR-664|nat|as|	tgagaacacaaaatgcatta	vvi-miR398a|-10_perfect match probe
vvi|miR-664|2mut|as|	tgagaacccaaaatgaatta	vvi-miR398a|-10_mismatch probe
vvi|miR-665|nat|as|	aggggtgacctgagaacaca	vvi-miR398a|0_perfect match probe
vvi|miR-665|2mut|as|	aggggtgatccgagaacaca	vvi-miR398a|0_mismatch probe
vvi|miR-666|nat|as|	ggtgccccaaaggggtgacc	vvi-miR398a|+10_perfect match probe
vvi|miR-666|2mut|as|	ggtgtcccaaaggtgtgacc	vvi-miR398a|+10_mismatch probe
vvi|miR-667|nat|as|	tgagaacacatgagagcaaa	vvi-miR398b|-10_perfect match probe
vvi|miR-667|2mut|as|	tgagaacccatgagaccaaa	vvi-miR398b|-10_mismatch probe
vvi|miR-668|nat|as|	aggggcgacctgagaacaca	vvi-miR398b|0_perfect match probe
vvi|miR-668|2mut|as|	aggggagacccgagaacaca	vvi-miR398b|0_mismatch probe
vvi|miR-669|nat|as|	ggagtccagcaggggcgacc	vvi-miR398b|+10_perfect match probe
vvi|miR-669|2mut|as|	ggagtccagaaggggagacc	vvi-miR398b|+10_mismatch probe
vvi|miR-670|nat|as|	tgagaacacatgagagcaaa	vvi-miR398c|-10_perfect match probe
vvi|miR-670|2mut|as|	tgagaacccatgagaccaaa	vvi-miR398c|-10_mismatch probe
vvi|miR-671|nat|as|	aggggcgacctgagaacaca	vvi-miR398c|0_perfect match probe
vvi|miR-671|2mut|as|	aggggagacccgagaacaca	vvi-miR398c|0_mismatch probe
vvi|miR-672|nat|as|	ggagtccagcaggggcgacc	vvi-miR398c|+10_perfect match probe
vvi|miR-672|2mut|as|	ggagtccagaaggggagacc	vvi-miR398c|+10_mismatch probe
vvi|miR-673|nat|as|	tcctttggcagagcagctac	vvi-miR399a|-10_perfect match probe
vvi|miR-673|2mut|as|	tcctttgccagagaagctac	vvi-miR399a|-10_mismatch probe
vvi|miR-674|nat|as|	agggcaattctcctttggca	vvi-miR399a|0_perfect match probe
vvi|miR-674|2mut|as|	agggaaattcttctttggca	vvi-miR399a|0_mismatch probe
vvi|miR-675|nat|as|	attgaataacagggcaattc	vvi-miR399a|+10_perfect match probe
vvi|miR-675|2mut|as|	attgaataaaaggccaattc	vvi-miR399a|+10_mismatch probe
vvi|miR-676|nat|as|	tcctttggcaagtcacatct	vvi-miR399b|-10_perfect match

		probe
vvi|miR-676|2mut|as|	tcctttgccaagtcccatct	vvi-miR399b|-10_mismatch probe
vvi|miR-677|nat|as|	agggcaactctcctttggca	vvi-miR399b|0_perfect match probe
vvi|miR-677|2mut|as|	agggaaactcttctttggca	vvi-miR399b|0_mismatch probe
		vvi-miR399b|+10_perfect match
vvi|miR-678|nat|as|	aagcagtcacagggcaactc	probe
vvi|miR-678|2mut|as|	aagcagtcccaggccaactc	vvi-miR399b|+10_mismatch probe
		vvi-miR399c|-10_perfect match
vvi|miR-679|nat|as|	tcctttggcagatcattcac	probe
vvi|miR-679|2mut|as|	tcctttgccagataattcac	vvi-miR399c|-10_mismatch probe
vvi|miR-680|nat|as|	agggcaactctcctttggca	vvi-miR399c|0_perfect match probe
vvi|miR-680|2mut|as|	agggaaactcttctttggca	vvi-miR399c|0_mismatch probe
		vvi-miR399c|+10_perfect match
vvi|miR-681|nat|as|	aaccagacacagggcaactc	probe
vvi|miR-681|2mut|as|	aaccagacccaggccaactc	vvi-miR399c|+10_mismatch probe
		vvi-miR399d|-10_perfect match
vvi|miR-682|nat|as|	tcctttggcagacacactca	probe
vvi|miR-682|2mut|as|	tcctttgccagacaccctca	vvi-miR399d|-10_mismatch probe
vvi|miR-683|nat|as|	cgagcaaatctcctttggca	vvi-miR399d|0_perfect match probe
vvi|miR-683|2mut|as|	cgagaaaatcttctttggca	vvi-miR399d|0_mismatch probe
		vvi-miR399d|+10_perfect match
vvi|miR-684|nat|as|	gttaaattcacgagcaaatc	probe
vvi|miR-684|2mut|as|	gttaaattcccgagaaaatc	vvi-miR399d|+10_mismatch probe
		vvi-miR399e|-10_perfect match
vvi|miR-685|nat|as|	tcctttggcagagggcacac	probe
vvi|miR-685|2mut|as|	tcctttgccagaggccacac	vvi-miR399e|-10_mismatch probe
vvi|miR-686|nat|as|	cgggcaaatctcctttggca	vvi-miR399e|0_perfect match probe
vvi|miR-686|2mut|as|	cgggaaaatcttctttggca	vvi-miR399e|0_mismatch probe
		vvi-miR399e|+10_perfect match
vvi|miR-687|nat|as|	gaagaattgccgggcaaatc	probe
vvi|miR-687|2mut|as|	gaagaattgtcggccaaatc	vvi-miR399e|+10_mismatch probe
vvi|miR-688|nat|as|	tccttcggcagcgagaagac	vvi-miR399f|-10_perfect match probe
vvi|miR-688|2mut|as|	tccttcgccagagagaagac	vvi-miR399f|-10_mismatch probe
vvi|miR-689|nat|as|	aggacaaatctccttcggca	vvi-miR399f|0_perfect match probe

vvi|miR-689|2mut|as|	aggaaaaatctccttaggca
vvi|miR-690|nat|as|	gaagaattgcaggacaaatc
vvi|miR-690|2mut|as|	gaagaattgaagaacaaatc
vvi|miR-691|nat|as|	tcctttggcagtgagcttag
vvi|miR-691|2mut|as|	tcctttgccagtgagtttag
vvi|miR-692|nat|as|	ggggcaaatctcctttggca
vvi|miR-692|2mut|as|	ggggaaaatcttctttggca
vvi|miR-693|nat|as|	gctgaattgaggggcaaatc
vvi|miR-693|2mut|as|	gctgaattaagggccaaatc
vvi|miR-694|nat|as|	tcctttggcaaaacagaagt
vvi|miR-694|2mut|as|	tcctttgccaaaaaagaagt
vvi|miR-695|nat|as|	agggcaattctcctttggca
vvi|miR-695|2mut|as|	agggaaattcttctttggca
vvi|miR-696|nat|as|	agcgaatggcagggcaattc
vvi|miR-696|2mut|as|	agcgaatgccaggccaattc
vvi|miR-697|nat|as|	tcctttggcgggtcattgga
vvi|miR-697|2mut|as|	tcctttggagtgtcattgga
vvi|miR-698|nat|as|	agggcaactctcctttggcg
vvi|miR-698|2mut|as|	agggaaactcttctttggcg
vvi|miR-699|nat|as|	aagtagtcacagggcaactc
vvi|miR-699|2mut|as|	aagtagtcccaggccaactc
vvi|miR-700|nat|as|	gtgaatctaacgccgtcgaa
vvi|miR-700|2mut|as|	gtgaatttaaccccgtcgaa
vvi|miR-701|nat|as|	gagtttgtgcgtgaatctaa
vvi|miR-701|2mut|as|	gagtttgtgactgaatctaa
vvi|miR-702|nat|as|	cagaactcacgagtttgtgc

vvi-miR399f|0_mismatch probe
vvi-miR399f|+10_perfect match probe
vvi-miR399f|+10_mismatch probe
vvi-miR399g|-10_perfect match probe
vvi-miR399g|-10_mismatch probe
vvi-miR399g|0_perfect match probe
vvi-miR399g|0_mismatch probe
vvi-miR399g|+10_perfect match probe
vvi-miR399g|+10_mismatch probe
vvi-miR399h|-10_perfect match probe
vvi-miR399h|-10_mismatch probe
vvi-miR399h|0_perfect match probe
vvi-miR399h|0_mismatch probe
vvi-miR399h|+10_perfect match probe
vvi-miR399h|+10_mismatch probe
vvi-miR399i|-10_perfect match probe
vvi-miR399i|-10_mismatch probe
vvi-miR399i|0_perfect match probe
vvi-miR399i|0_mismatch probe
vvi-miR399i|+10_perfect match probe
vvi-miR399i|+10_mismatch probe
vvi-miR403a|-10_perfect match probe
vvi-miR403a|-10_mismatch probe
vvi-miR403a|0_perfect match probe
vvi-miR403a|0_mismatch probe
vvi-miR403a|+10_perfect match probe

vvi|miR-702|2mut|as|	cagaactcaaaagtttgtgc	vvi-miR403a|+10_mismatch probe
vvi|miR-703|nat|as|	gtgaatctaacacccctttg	vvi-miR403b|-10_perfect match probe
vvi|miR-703|2mut|as|	gtgaatttaacacctctttg	vvi-miR403b|-10_mismatch probe
vvi|miR-704|nat|as|	gagtttgtgcgtgaatctaa	vvi-miR403b|0_perfect match probe
vvi|miR-704|2mut|as|	gagtttgtgactgaatctaa	vvi-miR403b|0_mismatch probe
vvi|miR-705|nat|as|	gacagatcccgagtttgtgc	vvi-miR403b|+10_perfect match probe
vvi|miR-705|2mut|as|	gacagatccaaagtttgtgc	vvi-miR403b|+10_mismatch probe
vvi|miR-706|nat|as|	gtgaatctaacggcgtcgaa	vvi-miR403c|-10_perfect match probe
vvi|miR-706|2mut|as|	gtgaatttaacggagtcgaa	vvi-miR403c|-10_mismatch probe
vvi|miR-707|nat|as|	gagtttgtgcgtgaatctaa	vvi-miR403c|0_perfect match probe
vvi|miR-707|2mut|as|	gagtttgtgactgaatctaa	vvi-miR403c|0_mismatch probe
vvi|miR-708|nat|as|	gacagatcacgagtttgtgc	vvi-miR403c|+10_perfect match probe
vvi|miR-708|2mut|as|	gacagatcaaaagtttgtgc	vvi-miR403c|+10_mismatch probe
vvi|miR-709|nat|as|	gtgaatctaacacccctttg	vvi-miR403d|-10_perfect match probe
vvi|miR-709|2mut|as|	gtgaatttaacacctctttg	vvi-miR403d|-10_mismatch probe
vvi|miR-710|nat|as|	gagtttgtgcgtgaatctaa	vvi-miR403d|0_perfect match probe
vvi|miR-710|2mut|as|	gagtttgtgactgaatctaa	vvi-miR403d|0_mismatch probe
vvi|miR-711|nat|as|	gacagatcccgagtttgtgc	vvi-miR403d|+10_perfect match probe
vvi|miR-711|2mut|as|	gacagatccaaagtttgtgc	vvi-miR403d|+10_mismatch probe
vvi|miR-712|nat|as|	gtgaatctaacggcgtcgaa	vvi-miR403e|-10_perfect match probe
vvi|miR-712|2mut|as|	gtgaatttaacggagtcgaa	vvi-miR403e|-10_mismatch probe
vvi|miR-713|nat|as|	gagtttgtgcgtgaatctaa	vvi-miR403e|0_perfect match probe
vvi|miR-713|2mut|as|	gagtttgtgactgaatctaa	vvi-miR403e|0_mismatch probe
vvi|miR-714|nat|as|	gacagatcgcgagtttgtgc	vvi-miR403e|+10_perfect match probe
vvi|miR-714|2mut|as|	gacagatcgaaagtttgtgc	vvi-miR403e|+10_mismatch probe
vvi|miR-715|nat|as|	gtgaatctaagaccatagaa	vvi-miR403f|-10_perfect match probe

<table>
<tr><td>vvi|miR-715|2mut|as|</td><td>gtgaatttaagacaatagaa</td><td>vvi-miR403f|-10_mismatch probe</td></tr>
<tr><td>vvi|miR-716|nat|as|</td><td>gagtttgtgcgtgaatctaa</td><td>vvi-miR403f|0_perfect match probe</td></tr>
<tr><td>vvi|miR-716|2mut|as|</td><td>gagtttgtgactgaatctaa</td><td>vvi-miR403f|0_mismatch probe</td></tr>
<tr><td>vvi|miR-717|nat|as|</td><td>gacagattacgagtttgtgc</td><td>vvi-miR403f|+10_perfect match probe</td></tr>
<tr><td>vvi|miR-717|2mut|as|</td><td>gacagattaaaagtttgtgc</td><td>vvi-miR403f|+10_mismatch probe</td></tr>
<tr><td>vvi|miR-718|nat|as|</td><td>ggcagtgcatgggtagagca</td><td>vvi-miR408|-10_perfect match probe</td></tr>
<tr><td>vvi|miR-718|2mut|as|</td><td>ggcagtgaatgtgtagagca</td><td>vvi-miR408|-10_mismatch probe</td></tr>
<tr><td>vvi|miR-719|nat|as|</td><td>ccagggaagaggcagtgcat</td><td>vvi-miR408|0_perfect match probe</td></tr>
<tr><td>vvi|miR-719|2mut|as|</td><td>ccagtgaagagccagtgcat</td><td>vvi-miR408|0_mismatch probe</td></tr>
<tr><td>vvi|miR-720|nat|as|</td><td>agagacagagccagggaaga</td><td>vvi-miR408|+10_perfect match probe</td></tr>
<tr><td>vvi|miR-720|2mut|as|</td><td>agagacagactcagggaaga</td><td>vvi-miR408|+10_mismatch probe</td></tr>
<tr><td>vvi|miR-721|nat|as|</td><td>tgagggagattgacaacttc</td><td>vvi-miR477a|-10_perfect match probe</td></tr>
<tr><td>vvi|miR-721|2mut|as|</td><td>tgagtgagattgaaaacttc</td><td>vvi-miR477a|-10_mismatch probe</td></tr>
<tr><td>vvi|miR-722|nat|as|</td><td>tggaagcctttgagggagat</td><td>vvi-miR477a|0_perfect match probe</td></tr>
<tr><td>vvi|miR-722|2mut|as|</td><td>tggaagtctttgagtgagat</td><td>vvi-miR477a|0_mismatch probe</td></tr>
<tr><td>vvi|miR-723|nat|as|</td><td>ccaggaaaattggaagcctt</td><td>vvi-miR477a|+10_perfect match probe</td></tr>
<tr><td>vvi|miR-723|2mut|as|</td><td>ccagaaaaattggaaccctt</td><td>vvi-miR477a|+10_mismatch probe</td></tr>
<tr><td>vvi|miR-724|nat|as|</td><td>caataccacaccatgtctac</td><td>vvi-miR479|-10_perfect match probe</td></tr>
<tr><td>vvi|miR-724|2mut|as|</td><td>caatacaacacaatgtctac</td><td>vvi-miR479|-10_mismatch probe</td></tr>
<tr><td>vvi|miR-725|nat|as|</td><td>tgagccgaaccaataccaca</td><td>vvi-miR479|0_perfect match probe</td></tr>
<tr><td>vvi|miR-725|2mut|as|</td><td>tgagtcgaacaaataccaca</td><td>vvi-miR479|0_mismatch probe</td></tr>
<tr><td>vvi|miR-726|nat|as|</td><td>tgaaagaagatgagccgaac</td><td>vvi-miR479|+10_perfect match probe</td></tr>
<tr><td>vvi|miR-726|2mut|as|</td><td>tgaaagaacatgagtcgaac</td><td>vvi-miR479|+10_mismatch probe</td></tr>
<tr><td>vvi|miR-727|nat|as|</td><td>gaggagtaggaaagactagc</td><td>vvi-miR482a|-10_perfect match probe</td></tr>
<tr><td>vvi|miR-727|2mut|as|</td><td>gaggagtagaaaagcctagc</td><td>vvi-miR482a|-10_mismatch probe</td></tr>
<tr><td>vvi|miR-728|nat|as|</td><td>ataggaatgggaggagtagg</td><td>vvi-miR482a|0_perfect match probe</td></tr>
<tr><td>vvi|miR-728|2mut|as|</td><td>ataggaatgtgagaagtagg</td><td>vvi-miR482a|0_mismatch probe</td></tr>
<tr><td>vvi|miR-729|nat|as|</td><td>gaaaacaataggaatgg</td><td>vvi-miR482a|+10_perfect match probe</td></tr>
<tr><td>vvi|miR-729|2mut|as|</td><td>gaaaaaaatagaaatgg</td><td>vvi-miR482a|+10_mismatch probe</td></tr>
</table>

vvi|miR-730|nat|as|	ctcgttgtcaagcaattaca	vvi-miR535a|-10_perfect match probe
vvi|miR-730|2mut|as|	ctcgttctcaagaaattaca	vvi-miR535a|-10_mismatch probe
vvi|miR-731|nat|as|	cgtgctctctctcgttgtca	vvi-miR535a|0_perfect match probe
vvi|miR-731|2mut|as|	cgtgttctctctccttgtca	vvi-miR535a|0_mismatch probe
vvi|miR-732|nat|as|	tgctgactagcgtgctctct	vvi-miR535a|+10_perfect match probe
vvi|miR-732|2mut|as|	tgctgactacagtgctctct	vvi-miR535a|+10_mismatch probe
vvi|miR-733|nat|as|	ctcgttgtcaagcaattaca	vvi-miR535b|-10_perfect match probe
vvi|miR-733|2mut|as|	ctcgttctcaagaaattaca	vvi-miR535b|-10_mismatch probe
vvi|miR-734|nat|as|	cgtgctctctctcgttgtca	vvi-miR535b|0_perfect match probe
vvi|miR-734|2mut|as|	cgtgttctctctccttgtca	vvi-miR535b|0_mismatch probe
vvi|miR-735|nat|as|	tgctgactagcgtgctctct	vvi-miR535b|+10_perfect match probe
vvi|miR-735|2mut|as|	tgctgactacagtgctctct	vvi-miR535b|+10_mismatch probe
vvi|miR-736|nat|as|	ctcgttgtcaagcaattaca	vvi-miR535c|-10_perfect match probe
vvi|miR-736|2mut|as|	ctcgttctcaagaaattaca	vvi-miR535c|-10_mismatch probe
vvi|miR-737|nat|as|	cgtgctctctctcgttgtca	vvi-miR535c|0_perfect match probe
vvi|miR-737|2mut|as|	cgtgttctctctccttgtca	vvi-miR535c|0_mismatch probe
vvi|miR-738|nat|as|	tgctgactagcgtgctctct	vvi-miR535c|+10_perfect match probe
vvi|miR-738|2mut|as|	tgctgactacagtgctctct	vvi-miR535c|+10_mismatch probe
vvi|miR-739|nat|as|	ctcgttgtcaagcaattaca	vvi-miR535d|-10_perfect match probe
vvi|miR-739|2mut|as|	ctcgttctcaagaaattaca	vvi-miR535d|-10_mismatch probe
vvi|miR-740|nat|as|	cgtgctctctctcgttgtca	vvi-miR535d|0_perfect match probe
vvi|miR-740|2mut|as|	cgtgttctctctccttgtca	vvi-miR535d|0_mismatch probe
vvi|miR-741|nat|as|	tgcagactagcgtgctctct	vvi-miR535d|+10_perfect match probe
vvi|miR-741|2mut|as|	tgcagactacagtgctctct	vvi-miR535d|+10_mismatch probe
vvi|miR-742|nat|as|	ctcgttgtcaagcaattaca	vvi-miR535e|-10_perfect match probe

vvi|miR-742|2mut|as|	ctcgttctcaagaaattaca	vvi-miR535e|-10_mismatch probe
vvi|miR-743|nat|as|	cgtgctctctctcgttgtca	vvi-miR535e|0_perfect match probe
vvi|miR-743|2mut|as|	cgtgttctctctccttgtca	vvi-miR535e|0_mismatch probe
		vvi-miR535e|+10_perfect match
vvi|miR-744|nat|as|	tgcagactagcgtgctctct	probe
vvi|miR-744|2mut|as|	tgcagactacagtgctctct	vvi-miR535e|+10_mismatch probe
		vvi-miR828a|-10_perfect match
vvi|miR-745|nat|as|	ttgagcaagaaactttata	probe
vvi|miR-745|2mut|as|	ttgagaaagaaattttata	vvi-miR828a|-10_mismatch probe
vvi|miR-746|nat|as|	gaatactcatttgagcaaga	vvi-miR828a|0_perfect match probe
vvi|miR-746|2mut|as|	gaatactaatttgagaaaga	vvi-miR828a|0_mismatch probe
		vvi-miR828a|+10_perfect match
vvi|miR-747|nat|as|	ctgttgtttggaatactcat	probe
vvi|miR-747|2mut|as|	ctgttgtttcaaatactcat	vvi-miR828a|+10_mismatch probe
		vvi-miR828b|-10_perfect match
vvi|miR-748|nat|as|	ttgagcaagaaaccaagagc	probe
vvi|miR-748|2mut|as|	ttgagaaagaaacaaagagc	vvi-miR828b|-10_mismatch probe
vvi|miR-749|nat|as|	gaacactcatttgagcaaga	vvi-miR828b|0_perfect match probe
vvi|miR-749|2mut|as|	gaaccctcatttgagaaaga	vvi-miR828b|0_mismatch probe
		vvi-miR828b|+10_perfect match
vvi|miR-750|nat|as|	ggttgatgtggaacactcat	probe
vvi|miR-750|2mut|as|	ggttgatgtcaaacactcat	vvi-miR828b|+10_mismatch probe
		vvi-miR845a|-10_perfect match
vvi|miR-751|nat|as|	atcagagctagacctcttgc	probe
vvi|miR-751|2mut|as|	atcagagttagatctcttgc	vvi-miR845a|-10_mismatch probe
vvi|miR-752|nat|as|	atcaattggtatcagagcta	vvi-miR845a|0_perfect match probe
vvi|miR-752|2mut|as|	atcaattcgtatccgagcta	vvi-miR845a|0_mismatch probe
		vvi-miR845a|+10_perfect match
vvi|miR-753|nat|as|	cctttgttttatcaattggt	probe
vvi|miR-753|2mut|as|	cctttcttttataaattggt	vvi-miR845a|+10_mismatch probe
		vvi-miR845b|-10_perfect match
vvi|miR-754|nat|as|	atcagagctagacctcttgc	probe
vvi|miR-754|2mut|as|	atcagagttagatctcttgc	vvi-miR845b|-10_mismatch probe
vvi|miR-755|nat|as|	atcaattggtatcagagcta	vvi-miR845b|0_perfect match probe

<table>
<tr><td>vvi|miR-755|2mut|as|</td><td>atcaattcgtatccgagcta</td><td>vvi-miR845b|0_mismatch probe</td></tr>
<tr><td></td><td></td><td>vvi-miR845b|+10_perfect match</td></tr>
<tr><td>vvi|miR-756|nat|as|</td><td>cctttgttttatcaattggt</td><td>probe</td></tr>
<tr><td>vvi|miR-756|2mut|as|</td><td>cctttcttttataaattggt</td><td>vvi-miR845b|+10_mismatch probe</td></tr>
<tr><td></td><td></td><td>vvi-miR845c|-10_perfect match</td></tr>
<tr><td>vvi|miR-757|nat|as|</td><td>atcagagcctcatgtttaat</td><td>probe</td></tr>
<tr><td>vvi|miR-757|2mut|as|</td><td>atcagagtctcatctttaat</td><td>vvi-miR845c|-10_mismatch probe</td></tr>
<tr><td>vvi|miR-758|nat|as|</td><td>atcaattggtatcagagcct</td><td>vvi-miR845c|0_perfect match probe</td></tr>
<tr><td>vvi|miR-758|2mut|as|</td><td>atcaattcgtatccgagcct</td><td>vvi-miR845c|0_mismatch probe</td></tr>
<tr><td></td><td></td><td>vvi-miR845c|+10_perfect match</td></tr>
<tr><td>vvi|miR-759|nat|as|</td><td>tggtttatgcatcaattggt</td><td>probe</td></tr>
<tr><td>vvi|miR-759|2mut|as|</td><td>tggtttatgaataaattggt</td><td>vvi-miR845c|+10_mismatch probe</td></tr>
<tr><td></td><td></td><td>vvi-miR845d|-10_perfect match</td></tr>
<tr><td>vvi|miR-760|nat|as|</td><td>atcagagccataggttagta</td><td>probe</td></tr>
<tr><td>vvi|miR-760|2mut|as|</td><td>atcagagtcatagcttagta</td><td>vvi-miR845d|-10_mismatch probe</td></tr>
<tr><td>vvi|miR-761|nat|as|</td><td>atcaattggtatcagagcca</td><td>vvi-miR845d|0_perfect match probe</td></tr>
<tr><td>vvi|miR-761|2mut|as|</td><td>atcaattcgtatccgagcca</td><td>vvi-miR845d|0_mismatch probe</td></tr>
<tr><td></td><td></td><td>vvi-miR845d|+10_perfect match</td></tr>
<tr><td>vvi|miR-762|nat|as|</td><td>ggtttttcccatcaattggt</td><td>probe</td></tr>
<tr><td>vvi|miR-762|2mut|as|</td><td>ggtttttctcataaattggt</td><td>vvi-miR845d|+10_mismatch probe</td></tr>
<tr><td></td><td></td><td>vvi-miR845e|-10_perfect match</td></tr>
<tr><td>vvi|miR-763|nat|as|</td><td>atcagagccataggttagta</td><td>probe</td></tr>
<tr><td>vvi|miR-763|2mut|as|</td><td>atcagagtcatagcttagta</td><td>vvi-miR845e|-10_mismatch probe</td></tr>
<tr><td>vvi|miR-764|nat|as|</td><td>atcaattggtatcagagcca</td><td>vvi-miR845e|0_perfect match probe</td></tr>
<tr><td>vvi|miR-764|2mut|as|</td><td>atcaattcgtatccgagcca</td><td>vvi-miR845e|0_mismatch probe</td></tr>
<tr><td></td><td></td><td>vvi-miR845e|+10_perfect match</td></tr>
<tr><td>vvi|miR-765|nat|as|</td><td>agtttttcccatcaattggt</td><td>probe</td></tr>
<tr><td>vvi|miR-765|2mut|as|</td><td>agtttttctcataaattggt</td><td>vvi-miR845e|+10_mismatch probe</td></tr>
<tr><td>vvi|miR-766|nat|as|</td><td>tgacagaagaagagagagcac</td><td>vvi-miR156a|*_perfect match probe</td></tr>
<tr><td>vvi|miR-766|2mut|as|</td><td>tgaccgaagaagagagcgcac</td><td>vvi-miR156a|*_mismatch probe</td></tr>
<tr><td>vvi|miR-767|nat|as|</td><td>tgacagaaagagaaatgagcac</td><td>vvi-miR156b|*_perfect match probe</td></tr>
<tr><td>vvi|miR-767|2mut|as|</td><td>tgaccgaaagagaaataagcac</td><td>vvi-miR156b|*_mismatch probe</td></tr>
<tr><td>vvi|miR-768|nat|as|</td><td>tgacagatagagagtgagcac</td><td>vvi-miR156c|*_perfect match probe</td></tr>
<tr><td>vvi|miR-768|2mut|as|</td><td>tgaccgatagagagcgagcac</td><td>vvi-miR156c|*_mismatch probe</td></tr>
</table>

vvi|miR-769|nat|as|	tgacagaaagagaggtgagcac	vvi-miR156d|*_perfect match probe
vvi|miR-769|2mut|as|	tgaccgaaagagagctgagcac	vvi-miR156d|*_mismatch probe
vvi|miR-770|nat|as|	tgacagatagggagtaagcac	vvi-miR156e|*_perfect match probe
vvi|miR-770|2mut|as|	tgacagatagtaagtaagcac	vvi-miR156e|*_mismatch probe
vvi|miR-771|nat|as|	ttgacaggagaagagagaggac	vvi-miR156f|*_perfect match probe
vvi|miR-771|2mut|as|	ttgacagaagaagagagcggac	vvi-miR156f|*_mismatch probe
vvi|miR-772|nat|as|	tgacagaagtctagagagcac	vvi-miR156g|*_perfect match probe
vvi|miR-772|2mut|as|	tgacagaagcctagagcgcac	vvi-miR156g|*_mismatch probe
vvi|miR-773|nat|as|	tgaaaggcgcagaaagaac	vvi-miR156h|*_perfect match probe
vvi|miR-773|2mut|as|	tgaaaggccccgaaagaac	vvi-miR156h|*_mismatch probe
vvi|miR-774|nat|as|	tgacagaagaatagagagcac	vvi-miR156i|*_perfect match probe
vvi|miR-774|2mut|as|	tgaccgaagaatagagcgcac	vvi-miR156i|*_mismatch probe
vvi|miR-775|nat|as|	ctggagcgtaaaggagctccc	vvi-miR159a|*_perfect match probe
vvi|miR-775|2mut|as|	ctggagagtaaaggagttccc	vvi-miR159a|*_mismatch probe
vvi|miR-776|nat|as|	ctggagtgtaaagaagctccca	vvi-miR159b|*_perfect match probe
vvi|miR-776|2mut|as|	ctggagcgtaaagaagttccca	vvi-miR159b|*_mismatch probe
vvi|miR-777|nat|as|	ttggacttcaaggagctcca	vvi-miR159c|*_perfect match probe
vvi|miR-777|2mut|as|	ttggccttcaaggagttcca	vvi-miR159c|*_mismatch probe
vvi|miR-778|nat|as|	tgcatgactcctctgatgcca	vvi-miR160a|*_perfect match probe
vvi|miR-778|2mut|as|	tgcatgacttctccgatgcca	vvi-miR160a|*_mismatch probe
vvi|miR-779|nat|as|	tgcttgactcccctcatgcca	vvi-miR160b|*_perfect match probe
vvi|miR-779|2mut|as|	tgcttgactcttctcatgcca	vvi-miR160b|*_mismatch probe
vvi|miR-780|nat|as|	tgcttggctcctcgcacgcca	vvi-miR160c|*_perfect match probe
vvi|miR-780|2mut|as|	tgcttgcctcctcccacgcca	vvi-miR160c|*_mismatch probe
vvi|miR-781|nat|as|	tgcatggctcctcgcacgcca	vvi-miR160d|*_perfect match probe
vvi|miR-781|2mut|as|	tgcatgcctcctcccacgcca	vvi-miR160d|*_mismatch probe
vvi|miR-782|nat|as|	tgcttgactcccctcatgcca	vvi-miR160e|*_perfect match probe
vvi|miR-782|2mut|as|	tgcttgactcttctcatgcca	vvi-miR160e|*_mismatch probe
vvi|miR-783|nat|as|	tgcatggctcctcatacgcca	vvi-miR160f|*_perfect match probe
vvi|miR-783|2mut|as|	tgcatgcctcctcataagcca	vvi-miR160f|*_mismatch probe
vvi|miR-784|nat|as|	tcgatgaaccgctgcatccag	vvi-miR162|*_perfect match probe
vvi|miR-784|2mut|as|	tcgatgaacccctgaatccag	vvi-miR162|*_mismatch probe
vvi|miR-785|nat|as|	tggagaaggggagcacgtgc	vvi-miR164a|*_perfect match probe
vvi|miR-785|2mut|as|	tggagaaggtgagaacgtgc	vvi-miR164a|*_mismatch probe

vvi|miR-786|nat|as|	ggagagccagggcacatg	vvi-miR164b|*_perfect match probe
vvi|miR-786|2mut|as|	ggagagtcaggccacatg	vvi-miR164b|*_mismatch probe
vvi|miR-787|nat|as|	tggagaaggggagcacgtgca	vvi-miR164d|*_perfect match probe
vvi|miR-787|2mut|as|	tggagaaggtgagaacgtgca	vvi-miR164d|*_mismatch probe
vvi|miR-788|nat|as|	tcgagccagacaacattcccc	vvi-miR166c|*_perfect match probe
vvi|miR-788|2mut|as|	tcgagtcagacaaaattcccc	vvi-miR166c|*_mismatch probe
vvi|miR-789|nat|as|	tcgagccagacaacaatcccct	vvi-miR166d|*_perfect match probe
vvi|miR-789|2mut|as|	tcgagtcagacaacaattccct	vvi-miR166d|*_mismatch probe
vvi|miR-790|nat|as|	tcgagccagacaacattcccc	vvi-miR166e|*_perfect match probe
vvi|miR-790|2mut|as|	tcgagtcagacaaaattcccc	vvi-miR166e|*_mismatch probe
vvi|miR-791|nat|as|	tcgagccagccaacattcccc	vvi-miR166f|*_perfect match probe
vvi|miR-791|2mut|as|	tcgagccagtcaaaattcccc	vvi-miR166f|*_mismatch probe
vvi|miR-792|nat|as|	tcgaaccagacaacattcccct	vvi-miR166g|*_perfect match probe
vvi|miR-792|2mut|as|	tcgaacaagacaacatttccct	vvi-miR166g|*_mismatch probe
vvi|miR-793|nat|as|	tcgaaccagacagcgttcccc	vvi-miR166h|*_perfect match probe
vvi|miR-793|2mut|as|	tcgaacaagacagagttcccc	vvi-miR166h|*_mismatch probe
vvi|miR-794|nat|as|	ttggatcaaacctcattccaa	vvi-miR166a|*_perfect match probe
vvi|miR-794|2mut|as|	ttggatcaaatttcattccaa	vvi-miR166a|*_mismatch probe
vvi|miR-795|nat|as|	tcgagccagccaacattcccc	vvi-miR166b|*_perfect match probe
vvi|miR-795|2mut|as|	tcgagccagtcaaaattcccc	vvi-miR166b|*_mismatch probe
vvi|miR-796|nat|as|	tgaagctgcaagatgacctg	vvi-miR167a|*_perfect match probe
vvi|miR-796|2mut|as|	tgaagctgaaagatgccctg	vvi-miR167a|*_mismatch probe
vvi|miR-797|nat|as|	tgaaactgccacatgatcta	vvi-miR167b|*_perfect match probe
vvi|miR-797|2mut|as|	tgaaactgtcccatgatcta	vvi-miR167b|*_mismatch probe
vvi|miR-798|nat|as|	tgaggctaccacatgatct	vvi-miR167c|*_perfect match probe
vvi|miR-798|2mut|as|	tgagcctaccccatgatct	vvi-miR167c|*_mismatch probe
vvi|miR-799|nat|as|	tgaggctgtcagggcatgaccta	vvi-miR167d|*_perfect match probe
vvi|miR-799|2mut|as|	tgagcctgtcaggccatgaccta	vvi-miR167d|*_mismatch probe
vvi|miR-800|nat|as|	tgaaactgccacatgatct	vvi-miR167e|*_perfect match probe
vvi|miR-800|2mut|as|	tgaaactgtcccatgatct	vvi-miR167e|*_mismatch probe
vvi|miR-801|nat|as|	tcagttgatgcaaggcggga	vvi-miR168|*_perfect match probe
vvi|miR-801|2mut|as|	tcagttgatccaaggaggga	vvi-miR168|*_mismatch probe
vvi|miR-802|nat|as|	agccaaggacaacttgccag	vvi-miR169a|*_perfect match probe
vvi|miR-802|2mut|as|	agccaagaacaacttcccag	vvi-miR169a|*_mismatch probe

vvi|miR-803|nat|as|	tgccaaggagacttgcc	vvi-miR169y|*_perfect match probe
vvi|miR-803|2mut|as|	tgccaagaagccttgcc	vvi-miR169y|*_mismatch probe
vvi|miR-804|nat|as|	gagccaaggatgactaaccg	vvi-miR169b|*_perfect match probe
vvi|miR-804|2mut|as|	gagcaaaggatgcctaaccg	vvi-miR169b|*_mismatch probe
vvi|miR-805|nat|as|	agccaaggacaacttgccgg	vvi-miR169c|*_perfect match probe
vvi|miR-805|2mut|as|	agccaagaacaacttcccgg	vvi-miR169c|*_mismatch probe
vvi|miR-806|nat|as|	agccaaggacaacttgccag	vvi-miR169d|*_perfect match probe
vvi|miR-806|2mut|as|	agccaagaacaacttcccag	vvi-miR169d|*_mismatch probe
vvi|miR-807|nat|as|	ttagccaaggtgactgcctg	vvi-miR169e|*_perfect match probe
vvi|miR-807|2mut|as|	ttagtcaaggtgactccctg	vvi-miR169e|*_mismatch probe
vvi|miR-808|nat|as|	agccaaacacaacttgcc	vvi-miR169f|*_perfect match probe
vvi|miR-808|2mut|as|	agccaaaccaaacttgcc	vvi-miR169f|*_mismatch probe
vvi|miR-809|nat|as|	agccaaagacaacttgcc	vvi-miR169g|*_perfect match probe
vvi|miR-809|2mut|as|	agccaaagcaaacttgcc	vvi-miR169g|*_mismatch probe
vvi|miR-810|nat|as|	gagtcaaggataaccctccg	vvi-miR169h|*_perfect match probe
vvi|miR-810|2mut|as|	gagtcaagaataactctccg	vvi-miR169h|*_mismatch probe
vvi|miR-811|nat|as|	gagacagggataaccagccgt	vvi-miR169i|*_perfect match probe
vvi|miR-811|2mut|as|	gagacagtgataaccacccgt	vvi-miR169i|*_mismatch probe
vvi|miR-812|nat|as|	agccaaggacaacctgccag	vvi-miR169j|*_perfect match probe
vvi|miR-812|2mut|as|	agccaagaacaacctcccag	vvi-miR169j|*_mismatch probe
vvi|miR-813|nat|as|	agccaaggacaacctgccag	vvi-miR169k|*_perfect match probe
vvi|miR-813|2mut|as|	agccaagaacaacctcccag	vvi-miR169k|*_mismatch probe
vvi|miR-814|nat|as|	gagccaagaataactagccgt	vvi-miR169l|*_perfect match probe
vvi|miR-814|2mut|as|	gagcaaagaataactacccgt	vvi-miR169l|*_mismatch probe
vvi|miR-815|nat|as|	gagcctcggatgcttgccag	vvi-miR169m|*_perfect match probe
vvi|miR-815|2mut|as|	gagcctcagatgcttcccag	vvi-miR169m|*_mismatch probe
vvi|miR-816|nat|as|	gagcctcagatgcttgccagttatg	vvi-miR169n|*_perfect match probe
vvi|miR-816|2mut|as|	gagcctcagatccttgtcagttatg	vvi-miR169n|*_mismatch probe
vvi|miR-817|nat|as|	gagccaagaataactcgctgtc	vvi-miR169o|*_perfect match probe
vvi|miR-817|2mut|as|	gagcaaagaataactccctgtc	vvi-miR169o|*_mismatch probe
vvi|miR-818|nat|as|	gagcctcggatgcttgccag	vvi-miR169p|*_perfect match probe
vvi|miR-818|2mut|as|	gagcctcagatgcttcccag	vvi-miR169p|*_mismatch probe
vvi|miR-819|nat|as|	gagccaggattacatgccgg	vvi-miR169q|*_perfect match probe
vvi|miR-819|2mut|as|	gagcaaggattacatcccgg	vvi-miR169q|*_mismatch probe

vvi|miR-820|nat|as|	tgagtcaagtcaacttgccg	vvi-miR169r|*_perfect match probe
vvi|miR-820|2mut|as|	tgagtcaagcaaacttgccg	vvi-miR169r|*_mismatch probe
vvi|miR-821|nat|as|	agccaggaacaacttgccgg	vvi-miR169s|*_perfect match probe
vvi|miR-821|2mut|as|	agccagaaacaacttcccgg	vvi-miR169s|*_mismatch probe
vvi|miR-822|nat|as|	gagtcaagtcaacttgccg	vvi-miR169t|*_perfect match probe
vvi|miR-822|2mut|as|	gagtcaagccaatttgccg	vvi-miR169t|*_mismatch probe
vvi|miR-823|nat|as|	gagtcaagtcaacttgccg	vvi-miR169u|*_perfect match probe
vvi|miR-823|2mut|as|	gagtcaagccaatttgccg	vvi-miR169u|*_mismatch probe
vvi|miR-824|nat|as|	agccaagaaataaattgccga	vvi-miR169v|*_perfect match probe
vvi|miR-824|2mut|as|	agccaacaaataaattcccga	vvi-miR169v|*_mismatch probe
vvi|miR-825|nat|as|	agctaagaatgactcgccga	vvi-miR169w|*_perfect match probe
vvi|miR-825|2mut|as|	agctaacaatgactccccga	vvi-miR169w|*_mismatch probe
vvi|miR-826|nat|as|	tagccgaaggaatgactgccta	vvi-miR169x|*_perfect match probe
vvi|miR-826|2mut|as|	tagcagaaggaatgactcccta	vvi-miR169x|*_mismatch probe
vvi|miR-827|nat|as|	ttgagccgtgccaatatcatg	vvi-miR171a|*_perfect match probe
vvi|miR-827|2mut|as|	ttgagccgtgtaaatatcatg	vvi-miR171a|*_mismatch probe
vvi|miR-828|nat|as|	aattgaggcacgccaatacc	vvi-miR171b|*_perfect match probe
vvi|miR-828|2mut|as|	aattgagccaccccaatacc	vvi-miR171b|*_mismatch probe
vvi|miR-829|nat|as|	ttgaaccgcaccaatatcccg	vvi-miR171c|*_perfect match probe
vvi|miR-829|2mut|as|	ttgaaccccacaaatatcccg	vvi-miR171c|*_mismatch probe
vvi|miR-830|nat|as|	ttgaaccgtatcaatatctcg	vvi-miR171d|*_perfect match probe
vvi|miR-830|2mut|as|	ttgaacagtataaatatctcg	vvi-miR171d|*_mismatch probe
vvi|miR-831|nat|as|	gattgaacctcaccaacatc	vvi-miR171e|*_perfect match probe
vvi|miR-831|2mut|as|	gattgaatctcacaaacatc	vvi-miR171e|*_mismatch probe
vvi|miR-832|nat|as|	ttgaacctcaccaacatcgct	vvi-miR171f|*_perfect match probe
vvi|miR-832|2mut|as|	ttgaacctcataaacatcgct	vvi-miR171f|*_mismatch probe
vvi|miR-833|nat|as|	ccgatggaaccaacat	vvi-miR171g|*_perfect match probe
vvi|miR-833|2mut|as|	ccgatcgaacaaacat	vvi-miR171g|*_mismatch probe
vvi|miR-834|nat|as|	ggttgaaccgcaccaacatc	vvi-miR171h|*_perfect match probe
vvi|miR-834|2mut|as|	ggttgaacccaaccaacatc	vvi-miR171h|*_mismatch probe
vvi|miR-835|nat|as|	ttgagccattccaacatcccc	vvi-miR171i|*_perfect match probe
vvi|miR-835|2mut|as|	ttgagtcattcaaacatcccc	vvi-miR171i|*_mismatch probe
vvi|miR-836|nat|as|	gaatcttgatgatgctgcat	vvi-miR172a|*_perfect match probe
vvi|miR-836|2mut|as|	gaatcttaatgatcctgcat	vvi-miR172a|*_mismatch probe

vvi|miR-837|nat|as|	gaatcttgatgatgctgca	vvi-miR172b|*_perfect match probe
vvi|miR-837|2mut|as|	gaatcttaatgatcctgca	vvi-miR172b|*_mismatch probe
vvi|miR-838|nat|as|	gaatcttgatgatgctcca	vvi-miR172c|*_perfect match probe
vvi|miR-838|2mut|as|	gaatcttaatgatcctcca	vvi-miR172c|*_mismatch probe
vvi|miR-839|nat|as|	gaatcttgatgatgctgcat	vvi-miR172d|*_perfect match probe
vvi|miR-839|2mut|as|	gaatcttaatgatcctgcat	vvi-miR172d|*_mismatch probe
vvi|miR-840|nat|as|	ctggactaaagagagctcc	vvi-miR319b|*_perfect match probe
vvi|miR-840|2mut|as|	ctggcctaaagagacctcc	vvi-miR319b|*_mismatch probe
vvi|miR-841|nat|as|	tggactgaagaaagctctc	vvi-miR319c|*_perfect match probe
vvi|miR-841|2mut|as|	tggaccgaagaaagttctc	vvi-miR319c|*_mismatch probe
vvi|miR-842|nat|as|	tttgggctgcaaaggaaccccc	vvi-miR319e|*_perfect match probe
vvi|miR-842|2mut|as|	tttggcctgcaaaggaatcccc	vvi-miR319e|*_mismatch probe
vvi|miR-843|nat|as|	tggactgaaggaagctctc	vvi-miR319f|*_perfect match probe
vvi|miR-843|2mut|as|	tggactgaacgaagttctc	vvi-miR319f|*_mismatch probe
vvi|miR-844|nat|as|	attggactaaaaggagctc	vvi-miR319g|*_perfect match probe
vvi|miR-844|2mut|as|	attgaactaaaagaagctc	vvi-miR319g|*_mismatch probe
vvi|miR-845|nat|as|	aaactcaggatagatagcgcc	vvi-miR390|*_perfect match probe
vvi|miR-845|2mut|as|	aaactcagaatagataccgcc	vvi-miR390|*_mismatch probe
vvi|miR-846|nat|as|	tcctaagggatagcatgatcc	vvi-miR393a|*_perfect match probe
vvi|miR-846|2mut|as|	tcctaagtgatagaatgatcc	vvi-miR393a|*_mismatch probe
vvi|miR-847|nat|as|	tccaaagggatagcatgatcc	vvi-miR393b|*_perfect match probe
vvi|miR-847|2mut|as|	tccaaagtgatagaatgatcc	vvi-miR393b|*_mismatch probe
vvi|miR-848|nat|as|	ttggcatgctggccacctcc	vvi-miR394a|*_perfect match probe
vvi|miR-848|2mut|as|	ttggcatcctggtcacctcc	vvi-miR394a|*_mismatch probe
vvi|miR-849|nat|as|	ttggcagtatgcccacctcc	vvi-miR394b|*_perfect match probe
vvi|miR-849|2mut|as|	ttggaagtatgtccacctcc	vvi-miR394b|*_mismatch probe
vvi|miR-850|nat|as|	ttggcatcctggccgcctcc	vvi-miR394c|*_perfect match probe
vvi|miR-850|2mut|as|	ttggaatcctggccccctcc	vvi-miR394c|*_mismatch probe
vvi|miR-851|nat|as|	tgaagtgatcaagggaactc	vvi-miR395a|*_perfect match probe
vvi|miR-851|2mut|as|	tgaagcgatcaagtgaactc	vvi-miR395a|*_mismatch probe
vvi|miR-852|nat|as|	tgaagtggtaaggggaactc	vvi-miR395b|*_perfect match probe
vvi|miR-852|2mut|as|	tgaagtcgtaaggtgaactc	vvi-miR395b|*_mismatch probe
vvi|miR-853|nat|as|	tgaagtggtcaagggaactc	vvi-miR395c|*_perfect match probe
vvi|miR-853|2mut|as|	tgaagtcgtcaagtgaactc	vvi-miR395c|*_mismatch probe

vvi|miR-854|nat|as|	tgaagtggtcaggggaactc	vvi-miR395d|*_perfect match probe
vvi|miR-854|2mut|as|	tgaagtcgtcaggtgaactc	vvi-miR395d|*_mismatch probe
vvi|miR-855|nat|as|	tgaagtggtcaagggaactc	vvi-miR395e|*_perfect match probe
vvi|miR-855|2mut|as|	tgaagtcgtcaagtgaactc	vvi-miR395e|*_mismatch probe
vvi|miR-856|nat|as|	tgaagtggtcaggggaactc	vvi-miR395f|*_perfect match probe
vvi|miR-856|2mut|as|	tgaagtcgtcaggtgaactc	vvi-miR395f|*_mismatch probe
vvi|miR-857|nat|as|	tgaagtgctcaggggaactc	vvi-miR395g|*_perfect match probe
vvi|miR-857|2mut|as|	tgaagtcctcaggtgaactc	vvi-miR395g|*_mismatch probe
vvi|miR-858|nat|as|	tgaagtggtcaagggaactc	vvi-miR395h|*_perfect match probe
vvi|miR-858|2mut|as|	tgaagtcgtcaagtgaactc	vvi-miR395h|*_mismatch probe
vvi|miR-859|nat|as|	tgaagtggtcaggggaactc	vvi-miR395i|*_perfect match probe
vvi|miR-859|2mut|as|	tgaagtcgtcaggtgaactc	vvi-miR395i|*_mismatch probe
vvi|miR-860|nat|as|	tgaagtggtcaggggaactc	vvi-miR395j|*_perfect match probe
vvi|miR-860|2mut|as|	tgaagtcgtcaggtgaactc	vvi-miR395j|*_mismatch probe
vvi|miR-861|nat|as|	tgaagtggtcaagggaactc	vvi-miR395k|*_perfect match probe
vvi|miR-861|2mut|as|	tgaagtcgtcaagtgaactc	vvi-miR395k|*_mismatch probe
vvi|miR-862|nat|as|	tgaagtggtcaggggaactc	vvi-miR395l|*_perfect match probe
vvi|miR-862|2mut|as|	tgaagtcgtcaggtgaactc	vvi-miR395l|*_mismatch probe
vvi|miR-863|nat|as|	tgaagtgttcaagggaactc	vvi-miR395m|*_perfect match probe
vvi|miR-863|2mut|as|	tgaagcgttcaagtgaactc	vvi-miR395m|*_mismatch probe
vvi|miR-864|nat|as|	ctggaggggttgggggaactc	vvi-miR395n|*_perfect match probe
vvi|miR-864|2mut|as|	ctggaggtgttgggtgaactc	vvi-miR395n|*_mismatch probe
vvi|miR-865|nat|as|	ccacagctttcttgaact	vvi-miR396a|*_perfect match probe
vvi|miR-865|2mut|as|	ccacagttttcttaaact	vvi-miR396a|*_mismatch probe
vvi|miR-866|nat|as|	tttccacagctttcttgaactt	vvi-miR396b|*_perfect match probe
vvi|miR-866|2mut|as|	tttccacagttttcttaaactt	vvi-miR396b|*_mismatch probe
vvi|miR-867|nat|as|	ttccacaactttcctgagctg	vvi-miR396c|*_perfect match probe
vvi|miR-867|2mut|as|	ttccccaacttttctgagctg	vvi-miR396c|*_mismatch probe
vvi|miR-868|nat|as|	tcccacagctttattgaaccg	vvi-miR396d|*_perfect match probe
vvi|miR-868|2mut|as|	tcccacagttttattaaaccg	vvi-miR396d|*_mismatch probe
vvi|miR-869|nat|as|	tgattgagtgcagcgccaatg	vvi-miR397a|*_perfect match probe
vvi|miR-869|2mut|as|	tgattgagtgaagccccaatg	vvi-miR397a|*_mismatch probe
vvi|miR-870|nat|as|	tgattgagtgcagcgccaatg	vvi-miR397b|*_perfect match probe
vvi|miR-870|2mut|as|	tgattgagtgaagccccaatg	vvi-miR397b|*_mismatch probe

vvi|miR-871|nat|as|	gtgttctcaggtgccactccct	vvi-miR398a|*_perfect match probe
vvi|miR-871|2mut|as|	gtgttctcagctgtcactccct	vvi-miR398a|*_mismatch probe
vvi|miR-872|nat|as|	tgtgattctcaggtcacacctg	vvi-miR398b|*_perfect match probe
vvi|miR-872|2mut|as|	tgtgattctccgctcacacctg	vvi-miR398b|*_mismatch probe
vvi|miR-873|nat|as|	tgtgattctcaggtcactcctg	vvi-miR398c|*_perfect match probe
vvi|miR-873|2mut|as|	tgtgattctccgctcactcctg	vvi-miR398c|*_mismatch probe
vvi|miR-874|nat|as|	tgccaaaggagaatcacactg	vvi-miR399a|*_perfect match probe
vvi|miR-874|2mut|as|	tgccaaagaagaatcccactg	vvi-miR399a|*_mismatch probe
vvi|miR-875|nat|as|	tgccaagaaagaggtgcccta	vvi-miR399b|*_perfect match probe
vvi|miR-875|2mut|as|	tgccaagaaacaggtgtccta	vvi-miR399b|*_mismatch probe
vvi|miR-876|nat|as|	taccaagtgaaagaggccctg	vvi-miR399c|*_perfect match probe
vvi|miR-876|2mut|as|	taccaagcgaaagaggtcctg	vvi-miR399c|*_mismatch probe
vvi|miR-877|nat|as|	tgccaaaagaaatctgctc	vvi-miR399d|*_perfect match probe
vvi|miR-877|2mut|as|	tgccaaaacaaatccgctc	vvi-miR399d|*_mismatch probe
vvi|miR-878|nat|as|	tgccaaaagataatttgccc	vvi-miR399e|*_perfect match probe
vvi|miR-878|2mut|as|	tgccaaaacataattcgccc	vvi-miR399e|*_mismatch probe
vvi|miR-879|nat|as|	tgccaaagcagatttggccctctaatg	vvi-miR399f|*_perfect match probe
vvi|miR-879|2mut|as|	tgccaaagaagatttggtcctctaatg	vvi-miR399f|*_mismatch probe
vvi|miR-880|nat|as|	tgccaatggagtattgccc	vvi-miR399g|*_perfect match probe
vvi|miR-880|2mut|as|	tgccaatcgactattgccc	vvi-miR399g|*_mismatch probe
vvi|miR-881|nat|as|	tgccaaaggaggattgcactg	vvi-miR399h|*_perfect match probe
vvi|miR-881|2mut|as|	tgccaaagaaggattgaactg	vvi-miR399h|*_mismatch probe
vvi|miR-882|nat|as|	gccagaaggagagaagcccta	vvi-miR399i|*_perfect match probe
vvi|miR-882|2mut|as|	gccagaagaagagaagtccta	vvi-miR399i|*_mismatch probe
vvi|miR-883|nat|as|	tttgattcacgcacaaact	vvi-miR403a|*_perfect match probe
vvi|miR-883|2mut|as|	tttgattcaaccacaaact	vvi-miR403a|*_mismatch probe
vvi|miR-884|nat|as|	ttggattcgcgcacaaactcg	vvi-miR403b|*_perfect match probe
vvi|miR-884|2mut|as|	ttggattcgccaacaaactcg	vvi-miR403b|*_mismatch probe
vvi|miR-885|nat|as|	tttgattcacgcacaaacttg	vvi-miR403c|*_perfect match probe
vvi|miR-885|2mut|as|	tttgattcaccaacaaacttg	vvi-miR403c|*_mismatch probe
vvi|miR-886|nat|as|	gattcgcgcacaaactcg	vvi-miR403d|*_perfect match probe
vvi|miR-886|2mut|as|	gattcgcccccaaactcg	vvi-miR403d|*_mismatch probe
vvi|miR-887|nat|as|	tttgattcacgcacaaacttg	vvi-miR403e|*_perfect match probe
vvi|miR-887|2mut|as|	tttgattcaccaacaaacttg	vvi-miR403e|*_mismatch probe

vvi|miR-888|nat|as|	ttagagtcacgcacaaactc	vvi-miR403f|*_perfect match probe
vvi|miR-888|2mut|as|	ttagagtcaaccacaaactc	vvi-miR403f|*_mismatch probe
vvi|miR-889|nat|as|	atgcactacctcgtccccg	vvi-miR408|*_perfect match probe
vvi|miR-889|2mut|as|	atgcactatctcctccccg	vvi-miR408|*_mismatch probe
vvi|miR-890|nat|as|	aggtcccccactgtcttccaa	vvi-miR477a|*_perfect match probe
vvi|miR-890|2mut|as|	aggtccctcactctcttccaa	vvi-miR477a|*_mismatch probe
vvi|miR-891|nat|as|	gtgatattggttcggctcgtc	vvi-miR479|*_perfect match probe
vvi|miR-891|2mut|as|	gtgatattcgttcgcctcgtc	vvi-miR479|*_mismatch probe
vvi|miR-892|nat|as|	cctactctccaattcc	vvi-miR482a|*_perfect match probe
vvi|miR-892|2mut|as|	cctattctcaaattcc	vvi-miR482a|*_mismatch probe
vvi|miR-893|nat|as|	tgacagcgagagagagcacgcc	vvi-miR535a|*_perfect match probe
vvi|miR-893|2mut|as|	tgacagagagagagagaacgcc	vvi-miR535a|*_mismatch probe
vvi|miR-894|nat|as|	tgacagcgagagagagcacgcc	vvi-miR535b|*_perfect match probe
vvi|miR-894|2mut|as|	tgacagagagagagagaacgcc	vvi-miR535b|*_mismatch probe
vvi|miR-895|nat|as|	tgacagcgagagagagcacgcc	vvi-miR535c|*_perfect match probe
vvi|miR-895|2mut|as|	tgacagagagagagagaacgcc	vvi-miR535c|*_mismatch probe
vvi|miR-896|nat|as|	tgacagcgacagagagcacgcc	vvi-miR535d|*_perfect match probe
vvi|miR-896|2mut|as|	tgacagagacagagagaacgcc	vvi-miR535d|*_mismatch probe
vvi|miR-897|nat|as|	tgacagcgacagagagcacgcc	vvi-miR535e|*_perfect match probe
vvi|miR-897|2mut|as|	tgacagagacagagagaacgcc	vvi-miR535e|*_mismatch probe
vvi|miR-898|nat|as|	cttcctcaaatgagcatctca	vvi-miR828a|*_perfect match probe
vvi|miR-898|2mut|as|	cttcttcaaatgagaatctca	vvi-miR828a|*_mismatch probe
vvi|miR-899|nat|as|	ttgcaggaaatgactatcca	vvi-miR828b|*_perfect match probe
vvi|miR-899|2mut|as|	ttgcagaaaatgcctatcca	vvi-miR828b|*_mismatch probe
vvi|miR-900|nat|as|	taactcatgaaggaactaat	vvi-miR845a|*_perfect match probe
vvi|miR-900|2mut|as|	taactcataaagaaactaat	vvi-miR845a|*_mismatch probe
vvi|miR-901|nat|as|	aactcatgaaggaactaat	vvi-miR845b|*_perfect match probe
vvi|miR-901|2mut|as|	aactcataaagaaactaat	vvi-miR845b|*_mismatch probe
vvi|miR-902|nat|as|	aaactcagacaaccaaaaca	vvi-miR845c|*_perfect match probe
vvi|miR-902|2mut|as|	aaactcagaaaacaaaaaca	vvi-miR845c|*_mismatch probe
vvi|miR-903|nat|as|	tggccctagggataattaatg	vvi-miR845d|*_perfect match probe
vvi|miR-903|2mut|as|	tggccctagtgctaattaatg	vvi-miR845d|*_mismatch probe
vvi|miR-904|nat|as|	tggccctagggataattaatg	vvi-miR845e|*_perfect match probe
vvi|miR-904|2mut|as|	tggccctagtgctaattaatg	vvi-miR845e|*_mismatch probe

vvi|miR-905|nat|as|	aagaagagagagcacactcc	vvi-miR156a|*-5_perfect match probe
vvi|miR-905|2mut|as|	aagaagagcgagaacactcc	vvi-miR156a|*-5_mismatch probe
vvi|miR-906|nat|as|	gatgatgacagaagaagaga	vvi-miR156a|*+5_perfect match probe
vvi|miR-906|2mut|as|	gatgatgaaagaacaagaga	vvi-miR156a|*+5_mismatch probe
vvi|miR-907|nat|as|	aagagaaatgagcacgcaaa	vvi-miR156b|*-5_perfect match probe
vvi|miR-907|2mut|as|	aagagaaataagcacccaaa	vvi-miR156b|*-5_mismatch probe
vvi|miR-908|nat|as|	gaagctgacagaaagagaaa	vvi-miR156b|*+5_perfect match probe
vvi|miR-908|2mut|as|	gaagttgacagaaagcgaaa	vvi-miR156b|*+5_mismatch probe
vvi|miR-909|nat|as|	atagagagtgagcacgcaga	vvi-miR156c|*-5_perfect match probe
vvi|miR-909|2mut|as|	atagagagcgagcacccaga	vvi-miR156c|*-5_mismatch probe
vvi|miR-910|nat|as|	acagatgacagatagagagt	vvi-miR156c|*+5_perfect match probe
vvi|miR-910|2mut|as|	acagatgaaagatagcgagt	vvi-miR156c|*+5_mismatch probe
vvi|miR-911|nat|as|	aagagaggtgagcacgcacc	vvi-miR156d|*-5_perfect match probe
vvi|miR-911|2mut|as|	aagagagctgagcacccacc	vvi-miR156d|*-5_mismatch probe
vvi|miR-912|nat|as|	gaagctgacagaaagagagg	vvi-miR156d|*+5_perfect match probe
vvi|miR-912|2mut|as|	gaagttgacagaaagcgagg	vvi-miR156d|*+5_mismatch probe
vvi|miR-913|nat|as|	atagggagtaagcacacaga	vvi-miR156e|*-5_perfect match probe
vvi|miR-913|2mut|as|	atagtgagtaagaacacaga	vvi-miR156e|*-5_mismatch probe
vvi|miR-914|nat|as|	aggggtgacagatagggagt	vvi-miR156e|*+5_perfect match probe
vvi|miR-914|2mut|as|	agggctgacagatagtgagt	vvi-miR156e|*+5_mismatch probe
vvi|miR-915|nat|as|	gagaagagagaggacaagac	vvi-miR156f|*-5_perfect match probe
vvi|miR-915|2mut|as|	gagaagagcgagaacaagac	vvi-miR156f|*-5_mismatch probe
vvi|miR-916|nat|as|	ttctgttgacaggagaagag	vvi-miR156f|*+5_perfect match probe

vvi|miR-916|2mut|as|	ttctgttgaaagaagaagag
vvi|miR-917|nat|as|	aagtctagagagcacaaagg
vvi|miR-917|2mut|as|	aagtctagcgagaacaaagg
vvi|miR-918|nat|as|	ggtgatgacagaagtctaga
vvi|miR-918|2mut|as|	ggtgatgaaagaagcctaga
vvi|miR-919|nat|as|	aggcgcagaaagaactcagc
vvi|miR-919|2mut|as|	aggcccagaaagaattcagc
vvi|miR-920|nat|as|	cacaatgaaaggcgcagaaa
vvi|miR-920|2mut|as|	cacaataaaaggcccagaaa
vvi|miR-921|nat|as|	aagaatagagagcacaaagg
vvi|miR-921|2mut|as|	aagaatagcgagaacaaagg
vvi|miR-922|nat|as|	ggtgatgacagaagaataga
vvi|miR-922|2mut|as|	ggtgatgaaagaacaataga
vvi|miR-923|nat|as|	cgtaaaggagctcccataaa
vvi|miR-923|2mut|as|	cgtaaaggacctctcataaa
vvi|miR-924|nat|as|	cagaactggagcgtaaagga
vvi|miR-924|2mut|as|	cagaactcgagagtaaagga
vvi|miR-925|nat|as|	gtaaagaagctcccacaagc
vvi|miR-925|2mut|as|	gtaaagaagttctcacaagc
vvi|miR-926|nat|as|	cagttctggagtgtaaagaa
vvi|miR-926|2mut|as|	cagttctcgagcgtaaagaa
vvi|miR-927|nat|as|	cttcaaggagctccactcca
vvi|miR-927|2mut|as|	cttcaaggacttccactcca
vvi|miR-928|nat|as|	ctctattggacttcaaggag

vvi-miR156f|*+5_mismatch probe
vvi-miR156g|*-5_perfect match probe
vvi-miR156g|*-5_mismatch probe
vvi-miR156g|*+5_perfect match probe
vvi-miR156g|*+5_mismatch probe
vvi-miR156h|*-5_perfect match probe
vvi-miR156h|*-5_mismatch probe
vvi-miR156h|*+5_perfect match probe
vvi-miR156h|*+5_mismatch probe
vvi-miR156i|*-5_perfect match probe
vvi-miR156i|*-5_mismatch probe
vvi-miR156i|*+5_perfect match probe
vvi-miR156i|*+5_mismatch probe
vvi-miR159a|*-5_perfect match probe
vvi-miR159a|*-5_mismatch probe
vvi-miR159a|*+5_perfect match probe
vvi-miR159a|*+5_mismatch probe
vvi-miR159b|*-5_perfect match probe
vvi-miR159b|*-5_mismatch probe
vvi-miR159b|*+5_perfect match probe
vvi-miR159b|*+5_mismatch probe
vvi-miR159c|*-5_perfect match probe
vvi-miR159c|*-5_mismatch probe
vvi-miR159c|*+5_perfect match

		probe
vvi|miR-928|2mut|as|	ctctattcgacttaaaggag	vvi-miR159c|*+5_mismatch probe
		vvi-miR160a|*-5_perfect match
vvi|miR-929|nat|as|	actcctctgatgccaactag	probe
vvi|miR-929|2mut|as|	actcttctgatgtcaactag	vvi-miR160a|*-5_mismatch probe
		vvi-miR160a|*+5_perfect match
vvi|miR-930|nat|as|	gggcctgcatgactcctctg	probe
vvi|miR-930|2mut|as|	gggcctgaatgacttctctg	vvi-miR160a|*+5_mismatch probe
		vvi-miR160b|*-5_perfect match
vvi|miR-931|nat|as|	actcccctcatgccaaatag	probe
vvi|miR-931|2mut|as|	actcctctcatgtcaaatag	vvi-miR160b|*-5_mismatch probe
		vvi-miR160b|*+5_perfect match
vvi|miR-932|nat|as|	tcgcctgcttgactcccctc	probe
vvi|miR-932|2mut|as|	tcgcctccttgactctcctc	vvi-miR160b|*+5_mismatch probe
		vvi-miR160c|*-5_perfect match
vvi|miR-933|nat|as|	gctcctcgcacgccacccgc	probe
vvi|miR-933|2mut|as|	gctcctcccaccccacccgc	vvi-miR160c|*-5_mismatch probe
		vvi-miR160c|*+5_perfect match
vvi|miR-934|nat|as|	gggtatgcttggctcctcgc	probe
vvi|miR-934|2mut|as|	gggtatccttgcctcctcgc	vvi-miR160c|*+5_mismatch probe
		vvi-miR160d|*-5_perfect match
vvi|miR-935|nat|as|	gctcctcgcacgccatccgc	probe
vvi|miR-935|2mut|as|	gctcctcccaccccatccgc	vvi-miR160d|*-5_mismatch probe
		vvi-miR160d|*+5_perfect match
vvi|miR-936|nat|as|	atgcatgcatggctcctcgc	probe
vvi|miR-936|2mut|as|	atgcatgaatgcctcctcgc	vvi-miR160d|*+5_mismatch probe
		vvi-miR160e|*-5_perfect match
vvi|miR-937|nat|as|	actcccctcatgccaaatag	probe
vvi|miR-937|2mut|as|	actcctctcatgtcaaatag	vvi-miR160e|*-5_mismatch probe
		vvi-miR160e|*+5_perfect match
vvi|miR-938|nat|as|	tcgcctgcttgactcccctc	probe
vvi|miR-938|2mut|as|	tcgcctccttgactctcctc	vvi-miR160e|*+5_mismatch probe
vvi|miR-939|nat|as|	gctcctcatacgccatccac	vvi-miR160f|*-5_perfect match probe
vvi|miR-939|2mut|as|	gctcttcataccccatccac	vvi-miR160f|*-5_mismatch probe

vvi|miR-940|nat|as|	gcatatgcatggctcctcat	vvi-miR160f|*+5_perfect match probe
vvi|miR-940|2mut|as|	gcatatgaatgcctcctcat	vvi-miR160f|*+5_mismatch probe
vvi|miR-941|nat|as|	aaccgctgcatccagtgact	vvi-miR162|*-5_perfect match probe
vvi|miR-941|2mut|as|	aacccctgcatcaagtgact	vvi-miR162|*-5_mismatch probe
vvi|miR-942|nat|as|	agagatcgatgaaccgctgc	vvi-miR162|*+5_perfect match probe
vvi|miR-942|2mut|as|	agagatagatgaacccctgc	vvi-miR162|*+5_mismatch probe
vvi|miR-943|nat|as|	aaggggagcacgtgcaagcc	vvi-miR164a|*-5_perfect match probe
vvi|miR-943|2mut|as|	aaggtgagcacgtgaaagcc	vvi-miR164a|*-5_mismatch probe
vvi|miR-944|nat|as|	catgttggagaaggggagca	vvi-miR164a|*+5_perfect match probe
vvi|miR-944|2mut|as|	catgttcgagaaggtgagca	vvi-miR164a|*+5_mismatch probe
vvi|miR-945|nat|as|	gagccagggcacatgtaaaa	vvi-miR164b|*-5_perfect match probe
vvi|miR-945|2mut|as|	gagccaggccccatgtaaaa	vvi-miR164b|*-5_mismatch probe
vvi|miR-946|nat|as|	aagtgggagagccagggcac	vvi-miR164b|*+5_perfect match probe
vvi|miR-946|2mut|as|	aagtgtgagagtcagggcac	vvi-miR164b|*+5_mismatch probe
vvi|miR-947|nat|as|	aggggagcacgtgcagagca	vvi-miR164d|*-5_perfect match probe
vvi|miR-947|2mut|as|	aggggagaacgtgaagagca	vvi-miR164d|*-5_mismatch probe
vvi|miR-948|nat|as|	catgttggagaaggggagca	vvi-miR164d|*+5_perfect match probe
vvi|miR-948|2mut|as|	catgttcgagaaggtgagca	vvi-miR164d|*+5_mismatch probe
vvi|miR-949|nat|as|	cagacaacattcccctcaaa	vvi-miR166c|*-5_perfect match probe
vvi|miR-949|2mut|as|	cagacaaaattcctctcaaa	vvi-miR166c|*-5_mismatch probe
vvi|miR-950|nat|as|	tgtcctcgagccagacaaca	vvi-miR166c|*+5_perfect match probe
vvi|miR-950|2mut|as|	tgtcctcgactcagacaaca	vvi-miR166c|*+5_mismatch probe
vvi|miR-951|nat|as|	agacaacaatcccctcaaag	vvi-miR166d|*-5_perfect match probe
vvi|miR-951|2mut|as|	agacaaaaatcctctcaaag	vvi-miR166d|*-5_mismatch probe

<table>
<tr><td>vvi|miR-952|nat|as|</td><td>tggcctcgagccagacaaca</td><td>vvi-miR166d|*+5_perfect match probe</td></tr>
<tr><td>vvi|miR-952|2mut|as|</td><td>tggcctcgactcagacaaca</td><td>vvi-miR166d|*+5_mismatch probe</td></tr>
<tr><td>vvi|miR-953|nat|as|</td><td>cagacaacattcccctcaaa</td><td>vvi-miR166e|*-5_perfect match probe</td></tr>
<tr><td>vvi|miR-953|2mut|as|</td><td>cagacaaaattcctctcaaa</td><td>vvi-miR166e|*-5_mismatch probe</td></tr>
<tr><td>vvi|miR-954|nat|as|</td><td>tgtcctcgagccagacaaca</td><td>vvi-miR166e|*+5_perfect match probe</td></tr>
<tr><td>vvi|miR-954|2mut|as|</td><td>tgtcctcgactcagacaaca</td><td>vvi-miR166e|*+5_mismatch probe</td></tr>
<tr><td>vvi|miR-955|nat|as|</td><td>cagccaacattccccacaac</td><td>vvi-miR166f|*-5_perfect match probe</td></tr>
<tr><td>vvi|miR-955|2mut|as|</td><td>cagcaaacattcctcacaac</td><td>vvi-miR166f|*-5_mismatch probe</td></tr>
<tr><td>vvi|miR-956|nat|as|</td><td>aagcctcgagccagccaaca</td><td>vvi-miR166f|*+5_perfect match probe</td></tr>
<tr><td>vvi|miR-956|2mut|as|</td><td>aagcctcgacccagtcaaca</td><td>vvi-miR166f|*+5_mismatch probe</td></tr>
<tr><td>vvi|miR-957|nat|as|</td><td>agacaacattcccctcaact</td><td>vvi-miR166g|*-5_perfect match probe</td></tr>
<tr><td>vvi|miR-957|2mut|as|</td><td>agacaaaattcctctcaact</td><td>vvi-miR166g|*-5_mismatch probe</td></tr>
<tr><td>vvi|miR-958|nat|as|</td><td>ggatctcgaaccagacaaca</td><td>vvi-miR166g|*+5_perfect match probe</td></tr>
<tr><td>vvi|miR-958|2mut|as|</td><td>ggatctagaacaagacaaca</td><td>vvi-miR166g|*+5_mismatch probe</td></tr>
<tr><td>vvi|miR-959|nat|as|</td><td>cagacagcgttcccctcaac</td><td>vvi-miR166h|*-5_perfect match probe</td></tr>
<tr><td>vvi|miR-959|2mut|as|</td><td>cagacagagttcctctcaac</td><td>vvi-miR166h|*-5_mismatch probe</td></tr>
<tr><td>vvi|miR-960|nat|as|</td><td>tggtctcgaaccagacagcg</td><td>vvi-miR166h|*+5_perfect match probe</td></tr>
<tr><td>vvi|miR-960|2mut|as|</td><td>tggtctagaacaagacagcg</td><td>vvi-miR166h|*+5_mismatch probe</td></tr>
<tr><td>vvi|miR-961|nat|as|</td><td>caaacctcattccaaacacc</td><td>vvi-miR166a|*-5_perfect match probe</td></tr>
<tr><td>vvi|miR-961|2mut|as|</td><td>caaatctcattcaaaacacc</td><td>vvi-miR166a|*-5_mismatch probe</td></tr>
<tr><td>vvi|miR-962|nat|as|</td><td>ggatcttggatcaaacctca</td><td>vvi-miR166a|*+5_perfect match probe</td></tr>
<tr><td>vvi|miR-962|2mut|as|</td><td>ggatcttcgatcaaatctca</td><td>vvi-miR166a|*+5_mismatch probe</td></tr>
<tr><td>vvi|miR-963|nat|as|</td><td>cagccaacattcccctcaaa</td><td>vvi-miR166b|*-5_perfect match probe</td></tr>
</table>

vvi|miR-963|2mut|as|	cagcaaacattcctctcaaa	vvi-miR166b|*-5_mismatch probe
vvi|miR-964|nat|as|	atgcctcgagccagccaaca	vvi-miR166b|*+5_perfect match probe
vvi|miR-964|2mut|as|	atgcctcgacccagtcaaca	vvi-miR166b|*+5_mismatch probe
vvi|miR-965|nat|as|	ctgcaagatgacctgatcat	vvi-miR167a|*-5_perfect match probe
vvi|miR-965|2mut|as|	ctgcaagataatctgatcat	vvi-miR167a|*-5_mismatch probe
vvi|miR-966|nat|as|	gtgattgaagctgcaagatg	vvi-miR167a|*+5_perfect match probe
vvi|miR-966|2mut|as|	gtgattgaacctgaaagatg	vvi-miR167a|*+5_mismatch probe
vvi|miR-967|nat|as|	ctgccacatgatctagtctt	vvi-miR167b|*-5_perfect match probe
vvi|miR-967|2mut|as|	ctgcaacatgatctactctt	vvi-miR167b|*-5_mismatch probe
vvi|miR-968|nat|as|	acaggtgaaactgccacatg	vvi-miR167b|*+5_perfect match probe
vvi|miR-968|2mut|as|	acagctgaaactgtcacatg	vvi-miR167b|*+5_mismatch probe
vvi|miR-969|nat|as|	gctaccacatgatctgatct	vvi-miR167c|*-5_perfect match probe
vvi|miR-969|2mut|as|	gctacaacatgatccgatct	vvi-miR167c|*-5_mismatch probe
vvi|miR-970|nat|as|	acaggtgaggctaccacatg	vvi-miR167c|*+5_perfect match probe
vvi|miR-970|2mut|as|	acaggtgagcttaccacatg	vvi-miR167c|*+5_mismatch probe
vvi|miR-971|nat|as|	tcagggcatgacctagctag	vvi-miR167d|*-5_perfect match probe
vvi|miR-971|2mut|as|	tcaggccatgacctacctag	vvi-miR167d|*-5_mismatch probe
vvi|miR-972|nat|as|	aggagtgaggctgtcagggc	vvi-miR167d|*+5_perfect match probe
vvi|miR-972|2mut|as|	aggagtgagcttgtcagggc	vvi-miR167d|*+5_mismatch probe
vvi|miR-973|nat|as|	actgccacatgatctgatct	vvi-miR167e|*-5_perfect match probe
vvi|miR-973|2mut|as|	actgtcacatgatccgatct	vvi-miR167e|*-5_mismatch probe
vvi|miR-974|nat|as|	acaggtgaaactgccacatg	vvi-miR167e|*+5_perfect match probe
vvi|miR-974|2mut|as|	acagctgaaactgtcacatg	vvi-miR167e|*+5_mismatch probe

vvi|miR-975|nat|as|	tgatgcaaggcgggatcgga
vvi|miR-975|2mut|as|	tgatgcaagcagggatcgga
vvi|miR-976|nat|as|	ccgattcagttgatgcaagg
vvi|miR-976|2mut|as|	ccgattccgttgatgaaagg
vvi|miR-977|nat|as|	aggacaacttgccagggttc
vvi|miR-977|2mut|as|	aggaaaacttgtcagggttc
vvi|miR-978|nat|as|	aatgtagccaaggacaactt
vvi|miR-978|2mut|as|	aatgtagtcaagaacaactt
vvi|miR-979|nat|as|	ccaaggagacttgccataaa
vvi|miR-979|2mut|as|	ccaagaagacttgtcataaa
vvi|miR-980|nat|as|	tcaggtgccaaggagacttg
vvi|miR-980|2mut|as|	tcaggtgtcaagaagacttg
vvi|miR-981|nat|as|	aaggatgactaaccgtgcac
vvi|miR-981|2mut|as|	aaggatgcctaacagtgcac
vvi|miR-982|nat|as|	caaatgagccaaggatgact
vvi|miR-982|2mut|as|	caaatgagtcaagaatgact
vvi|miR-983|nat|as|	aggacaacttgccggattaa
vvi|miR-983|2mut|as|	aggaaaacttgtcggattaa
vvi|miR-984|nat|as|	aatgtagccaaggacaactt
vvi|miR-984|2mut|as|	aatgtagtcaagaacaactt
vvi|miR-985|nat|as|	aggacaacttgccagggttc
vvi|miR-985|2mut|as|	aggaaaacttgtcagggttc
vvi|miR-986|nat|as|	aatgtagccaaggacaactt
vvi|miR-986|2mut|as|	aatgtagtcaagaacaactt

vvi-miR168|*-5_perfect match probe
vvi-miR168|*-5_mismatch probe
vvi-miR168|*+5_perfect match probe
vvi-miR168|*+5_mismatch probe
vvi-miR169a|*-5_perfect match probe
vvi-miR169a|*-5_mismatch probe
vvi-miR169a|*+5_perfect match probe
vvi-miR169a|*+5_mismatch probe
vvi-miR169y|*-5_perfect match probe
vvi-miR169y|*-5_mismatch probe
vvi-miR169y|*+5_perfect match probe
vvi-miR169y|*+5_mismatch probe
vvi-miR169b|*-5_perfect match probe
vvi-miR169b|*-5_mismatch probe
vvi-miR169b|*+5_perfect match probe
vvi-miR169b|*+5_mismatch probe
vvi-miR169c|*-5_perfect match probe
vvi-miR169c|*-5_mismatch probe
vvi-miR169c|*+5_perfect match probe
vvi-miR169c|*+5_mismatch probe
vvi-miR169d|*-5_perfect match probe
vvi-miR169d|*-5_mismatch probe
vvi-miR169d|*+5_perfect match probe
vvi-miR169d|*+5_mismatch probe

<table>
<tr><td>vvi|miR-987|nat|as|</td><td>caaggtgactgcctggaacc</td><td>vvi-miR169e|*-5_perfect match probe</td></tr>
<tr><td>vvi|miR-987|2mut|as|</td><td>caagctgactgtctggaacc</td><td>vvi-miR169e|*-5_mismatch probe</td></tr>
<tr><td>vvi|miR-988|nat|as|</td><td>taaaattagccaaggtgact</td><td>vvi-miR169e|*+5_perfect match probe</td></tr>
<tr><td>vvi|miR-988|2mut|as|</td><td>taaaattagtaaaggtgact</td><td>vvi-miR169e|*+5_mismatch probe</td></tr>
<tr><td>vvi|miR-989|nat|as|</td><td>caaacacaacttgcccatta</td><td>vvi-miR169f|*-5_perfect match probe</td></tr>
<tr><td>vvi|miR-989|2mut|as|</td><td>caaacccaacttgtccatta</td><td>vvi-miR169f|*-5_mismatch probe</td></tr>
<tr><td>vvi|miR-990|nat|as|</td><td>catgtagccaaacacaactt</td><td>vvi-miR169f|*+5_perfect match probe</td></tr>
<tr><td>vvi|miR-990|2mut|as|</td><td>catgtagtcaaacccaactt</td><td>vvi-miR169f|*+5_mismatch probe</td></tr>
<tr><td>vvi|miR-991|nat|as|</td><td>caaagacaacttgccggctt</td><td>vvi-miR169g|*-5_perfect match probe</td></tr>
<tr><td>vvi|miR-991|2mut|as|</td><td>caaagaaaacttgtcggctt</td><td>vvi-miR169g|*-5_mismatch probe</td></tr>
<tr><td>vvi|miR-992|nat|as|</td><td>catgtagccaaagacaactt</td><td>vvi-miR169g|*+5_perfect match probe</td></tr>
<tr><td>vvi|miR-992|2mut|as|</td><td>catgtagtcaaagaaaactt</td><td>vvi-miR169g|*+5_mismatch probe</td></tr>
<tr><td>vvi|miR-993|nat|as|</td><td>aaggataaccctccgtgcgt</td><td>vvi-miR169h|*-5_perfect match probe</td></tr>
<tr><td>vvi|miR-993|2mut|as|</td><td>aaggataactctcagtgcgt</td><td>vvi-miR169h|*-5_mismatch probe</td></tr>
<tr><td>vvi|miR-994|nat|as|</td><td>taaaggagtcaaggataacc</td><td>vvi-miR169h|*+5_perfect match probe</td></tr>
<tr><td>vvi|miR-994|2mut|as|</td><td>taaagaagtcaagaataacc</td><td>vvi-miR169h|*+5_mismatch probe</td></tr>
<tr><td>vvi|miR-995|nat|as|</td><td>gggataaccagccgtgcatg</td><td>vvi-miR169i|*-5_perfect match probe</td></tr>
<tr><td>vvi|miR-995|2mut|as|</td><td>gggataacaagtcgtgcatg</td><td>vvi-miR169i|*-5_mismatch probe</td></tr>
<tr><td>vvi|miR-996|nat|as|</td><td>taaaggagacagggataacc</td><td>vvi-miR169i|*+5_perfect match probe</td></tr>
<tr><td>vvi|miR-996|2mut|as|</td><td>taaagaagacagtgataacc</td><td>vvi-miR169i|*+5_mismatch probe</td></tr>
<tr><td>vvi|miR-997|nat|as|</td><td>aggacaacctgccagggaca</td><td>vvi-miR169j|*-5_perfect match probe</td></tr>
<tr><td>vvi|miR-997|2mut|as|</td><td>aggacaatctgtcagggaca</td><td>vvi-miR169j|*-5_mismatch probe</td></tr>
<tr><td>vvi|miR-998|nat|as|</td><td>aaggtagccaaggacaacct</td><td>vvi-miR169j|*+5_perfect match probe</td></tr>
<tr><td>vvi|miR-998|2mut|as|</td><td>aaggtagtcaagaacaacct</td><td>vvi-miR169j|*+5_mismatch probe</td></tr>
<tr><td>vvi|miR-999|nat|as|</td><td>aggacaacctgccagggaca</td><td>vvi-miR169k|*-5_perfect match</td></tr>
</table>

<table>
<tr><td></td><td></td><td>probe</td></tr>
<tr><td>vvi|miR-999|2mut|as|</td><td>aggacaatctgtcagggaca</td><td>vvi-miR169k|*-5_mismatch probe</td></tr>
<tr><td></td><td></td><td>vvi-miR169k|*+5_perfect match</td></tr>
<tr><td>vvi|miR-1000|nat|as|</td><td>aaggtagccaaggacaacct</td><td>probe</td></tr>
<tr><td>vvi|miR-1000|2mut|as|</td><td>aaggtagtcaagaacaacct</td><td>vvi-miR169k|*+5_mismatch probe</td></tr>
<tr><td>vvi|miR-1001|nat|as|</td><td>agaataactagccgtgcatg</td><td>vvi-miR169l|*-5_perfect match probe</td></tr>
<tr><td>vvi|miR-1001|2mut|as|</td><td>agaataattagtcgtgcatg</td><td>vvi-miR169l|*-5_mismatch probe</td></tr>
<tr><td></td><td></td><td>vvi-miR169l|*+5_perfect match</td></tr>
<tr><td>vvi|miR-1002|nat|as|</td><td>caaatgagccaagaataact</td><td>probe</td></tr>
<tr><td>vvi|miR-1002|2mut|as|</td><td>caaatgagtcaacaataact</td><td>vvi-miR169l|*+5_mismatch probe</td></tr>
<tr><td></td><td></td><td>vvi-miR169m|*-5_perfect match</td></tr>
<tr><td>vvi|miR-1003|nat|as|</td><td>tcggatgcttgccagttatg</td><td>probe</td></tr>
<tr><td>vvi|miR-1003|2mut|as|</td><td>tcggatccttgtcagttatg</td><td>vvi-miR169m|*-5_mismatch probe</td></tr>
<tr><td></td><td></td><td>vvi-miR169m|*+5_perfect match</td></tr>
<tr><td>vvi|miR-1004|nat|as|</td><td>aaacagagcctcggatgctt</td><td>probe</td></tr>
<tr><td>vvi|miR-1004|2mut|as|</td><td>aaacagagtctcagatgctt</td><td>vvi-miR169m|*+5_mismatch probe</td></tr>
<tr><td></td><td></td><td>vvi-miR169n|*-5_perfect match</td></tr>
<tr><td>vvi|miR-1005|nat|as|</td><td>tgcttgccagttatgaccag</td><td>probe</td></tr>
<tr><td>vvi|miR-1005|2mut|as|</td><td>tgcttgtcagttatgcccag</td><td>vvi-miR169n|*-5_mismatch probe</td></tr>
<tr><td></td><td></td><td>vvi-miR169n|*+5_perfect match</td></tr>
<tr><td>vvi|miR-1006|nat|as|</td><td>aaatagagcctcagatgctt</td><td>probe</td></tr>
<tr><td>vvi|miR-1006|2mut|as|</td><td>aaatagagtctccgatgctt</td><td>vvi-miR169n|*+5_mismatch probe</td></tr>
<tr><td></td><td></td><td>vvi-miR169o|*-5_perfect match</td></tr>
<tr><td>vvi|miR-1007|nat|as|</td><td>gaataactcgctgtccatgc</td><td>probe</td></tr>
<tr><td>vvi|miR-1007|2mut|as|</td><td>gaataactccttgtccatgc</td><td>vvi-miR169o|*-5_mismatch probe</td></tr>
<tr><td></td><td></td><td>vvi-miR169o|*+5_perfect match</td></tr>
<tr><td>vvi|miR-1008|nat|as|</td><td>cgcatgagccaagaataact</td><td>probe</td></tr>
<tr><td>vvi|miR-1008|2mut|as|</td><td>cgcatgagtcaacaataact</td><td>vvi-miR169o|*+5_mismatch probe</td></tr>
<tr><td></td><td></td><td>vvi-miR169p|*-5_perfect match</td></tr>
<tr><td>vvi|miR-1009|nat|as|</td><td>tcggatgcttgccagttata</td><td>probe</td></tr>
<tr><td>vvi|miR-1009|2mut|as|</td><td>tcggatccttgtcagttata</td><td>vvi-miR169p|*-5_mismatch probe</td></tr>
<tr><td></td><td></td><td>vvi-miR169p|*+5_perfect match</td></tr>
<tr><td>vvi|miR-1010|nat|as|</td><td>aaacagagcctcggatgctt</td><td>probe</td></tr>
<tr><td>vvi|miR-1010|2mut|as|</td><td>aaacagagtctcagatgctt</td><td>vvi-miR169p|*+5_mismatch probe</td></tr>
</table>

		vvi-miR169q|*-5_perfect match
vvi|miR-1011|nat|as|	aggattacatgccggctgtt	probe
vvi|miR-1011|2mut|as|	aggattaaatgccgcctgtt	vvi-miR169q|*-5_mismatch probe
		vvi-miR169q|*+5_perfect match
vvi|miR-1012|nat|as|	aaatagagccaggattacat	probe
vvi|miR-1012|2mut|as|	aaatagagtcagaattacat	vvi-miR169q|*+5_mismatch probe
vvi|miR-1013|nat|as|	caagtcaacttgccggttac	vvi-miR169r|*-5_perfect match probe
vvi|miR-1013|2mut|as|	caagccaacttgtcggttac	vvi-miR169r|*-5_mismatch probe
		vvi-miR169r|*+5_perfect match
vvi|miR-1014|nat|as|	caaactgagtcaagtcaact	probe
vvi|miR-1014|2mut|as|	caaactgagccaagccaact	vvi-miR169r|*+5_mismatch probe
vvi|miR-1015|nat|as|	ggaacaacttgccgggaaga	vvi-miR169s|*-5_perfect match probe
vvi|miR-1015|2mut|as|	ggaaaaacttgtcgggaaga	vvi-miR169s|*-5_mismatch probe
		vvi-miR169s|*+5_perfect match
vvi|miR-1016|nat|as|	aatgtagccaggaacaactt	probe
vvi|miR-1016|2mut|as|	aatgtagtcagaaacaactt	vvi-miR169s|*+5_mismatch probe
vvi|miR-1017|nat|as|	caagtcaacttgccggttac	vvi-miR169t|*-5_perfect match probe
vvi|miR-1017|2mut|as|	caagccaacttgtcggttac	vvi-miR169t|*-5_mismatch probe
		vvi-miR169t|*+5_perfect match
vvi|miR-1018|nat|as|	aaactgagtcaagtcaactt	probe
vvi|miR-1018|2mut|as|	aaactgagccaagccaactt	vvi-miR169t|*+5_mismatch probe
		vvi-miR169u|*-5_perfect match
vvi|miR-1019|nat|as|	caagtcaacttgccggtaac	probe
vvi|miR-1019|2mut|as|	caagccaacttgtcggtaac	vvi-miR169u|*-5_mismatch probe
		vvi-miR169u|*+5_perfect match
vvi|miR-1020|nat|as|	aaacagagtcaagtcaactt	probe
vvi|miR-1020|2mut|as|	aaacagagccaagccaactt	vvi-miR169u|*+5_mismatch probe
		vvi-miR169v|*-5_perfect match
vvi|miR-1021|nat|as|	gaaataaattgccgatccat	probe
vvi|miR-1021|2mut|as|	gaaataaatcgtcgatccat	vvi-miR169v|*-5_mismatch probe
		vvi-miR169v|*+5_perfect match
vvi|miR-1022|nat|as|	aacatagccaagaaataaat	probe
vvi|miR-1022|2mut|as|	aacatagtcaacaaataaat	vvi-miR169v|*+5_mismatch probe
vvi|miR-1023|nat|as|	agaatgactcgccgaccacc	vvi-miR169w|*-5_perfect match

		probe
vvi|miR-1023|2mut|as|	agaatgactacccgaccacc	vvi-miR169w|*-5_mismatch probe
		vvi-miR169w|*+5_perfect match
vvi|miR-1024|nat|as|	aatgtagctaagaatgactc	probe
vvi|miR-1024|2mut|as|	aatgtagttaagaataactc	vvi-miR169w|*+5_mismatch probe
		vvi-miR169x|*-5_perfect match
vvi|miR-1025|nat|as|	aggaatgactgcctaaagac	probe
vvi|miR-1025|2mut|as|	aggaatgcctgtctaaagac	vvi-miR169x|*-5_mismatch probe
		vvi-miR169x|*+5_perfect match
vvi|miR-1026|nat|as|	cagtttagccgaaggaatga	probe
vvi|miR-1026|2mut|as|	cagtttagtcaaaggaatga	vvi-miR169x|*+5_mismatch probe
		vvi-miR171a|*-5_perfect match
vvi|miR-1027|nat|as|	cgtgccaatatcatgttcaa	probe
vvi|miR-1027|2mut|as|	cgtgtcaatatcatcttcaa	vvi-miR171a|*-5_mismatch probe
		vvi-miR171a|*+5_perfect match
vvi|miR-1028|nat|as|	tctgattgagccgtgccaat	probe
vvi|miR-1028|2mut|as|	tctgattgacccgtgtcaat	vvi-miR171a|*+5_mismatch probe
		vvi-miR171b|*-5_perfect match
vvi|miR-1029|nat|as|	aggcacgccaatacctcccc	probe
vvi|miR-1029|2mut|as|	aggcaccccaatatctcccc	vvi-miR171b|*-5_mismatch probe
		vvi-miR171b|*+5_perfect match
vvi|miR-1030|nat|as|	ctttaaattgaggcacgcca	probe
vvi|miR-1030|2mut|as|	ctttaaattaagccacgcca	vvi-miR171b|*+5_mismatch probe
		vvi-miR171c|*-5_perfect match
vvi|miR-1031|nat|as|	cgcaccaatatcccgtaata	probe
vvi|miR-1031|2mut|as|	cgcacaaatatccagtaata	vvi-miR171c|*-5_mismatch probe
		vvi-miR171c|*+5_perfect match
vvi|miR-1032|nat|as|	tcttattgaaccgcaccaat	probe
vvi|miR-1032|2mut|as|	tcttattaaaccccaccaat	vvi-miR171c|*+5_mismatch probe
		vvi-miR171d|*-5_perfect match
vvi|miR-1033|nat|as|	cgtatcaatatctcgtgtat	probe
vvi|miR-1033|2mut|as|	cgtataaatatctcctgtat	vvi-miR171d|*-5_mismatch probe
		vvi-miR171d|*+5_perfect match
vvi|miR-1034|nat|as|	tctaattgaaccgtatcaat	probe

<table>
<tr><td>vvi|miR-1034|2mut|as|</td><td>tctaattaaacagtatcaat</td><td>vvi-miR171d|*+5_mismatch probe</td></tr>
<tr><td></td><td></td><td>vvi-miR171e|*-5_perfect match</td></tr>
<tr><td>vvi|miR-1035|nat|as|</td><td>aacctcaccaacatcgctta</td><td>probe</td></tr>
<tr><td>vvi|miR-1035|2mut|as|</td><td>aacctcacaaacatccctta</td><td>vvi-miR171e|*-5_mismatch probe</td></tr>
<tr><td></td><td></td><td>vvi-miR171e|*+5_perfect match</td></tr>
<tr><td>vvi|miR-1036|nat|as|</td><td>cttcggattgaacctcacca</td><td>probe</td></tr>
<tr><td>vvi|miR-1036|2mut|as|</td><td>cttcagattgaatctcacca</td><td>vvi-miR171e|*+5_mismatch probe</td></tr>
<tr><td>vvi|miR-1037|nat|as|</td><td>ctcaccaacatcgctttctt</td><td>vvi-miR171f|*-5_perfect match probe</td></tr>
<tr><td>vvi|miR-1037|2mut|as|</td><td>ctcacaaacatccctttctt</td><td>vvi-miR171f|*-5_mismatch probe</td></tr>
<tr><td></td><td></td><td>vvi-miR171f|*+5_perfect match</td></tr>
<tr><td>vvi|miR-1038|nat|as|</td><td>tcagattgaacctcaccaac</td><td>probe</td></tr>
<tr><td>vvi|miR-1038|2mut|as|</td><td>tcagattaaacctcatcaac</td><td>vvi-miR171f|*+5_mismatch probe</td></tr>
<tr><td></td><td></td><td>vvi-miR171g|*-5_perfect match</td></tr>
<tr><td>vvi|miR-1039|nat|as|</td><td>cgatggaaccaacatggaga</td><td>probe</td></tr>
<tr><td>vvi|miR-1039|2mut|as|</td><td>cgatggaacaaacatcgaga</td><td>vvi-miR171g|*-5_mismatch probe</td></tr>
<tr><td></td><td></td><td>vvi-miR171g|*+5_perfect match</td></tr>
<tr><td>vvi|miR-1040|nat|as|</td><td>ccccaccgatggaaccaaca</td><td>probe</td></tr>
<tr><td>vvi|miR-1040|2mut|as|</td><td>ccccacagatggaacaaaca</td><td>vvi-miR171g|*+5_mismatch probe</td></tr>
<tr><td></td><td></td><td>vvi-miR171h|*-5_perfect match</td></tr>
<tr><td>vvi|miR-1041|nat|as|</td><td>aaccgcaccaacatctcgtg</td><td>probe</td></tr>
<tr><td>vvi|miR-1041|2mut|as|</td><td>aaccccaccaaaatctcgtg</td><td>vvi-miR171h|*-5_mismatch probe</td></tr>
<tr><td></td><td></td><td>vvi-miR171h|*+5_perfect match</td></tr>
<tr><td>vvi|miR-1042|nat|as|</td><td>agttaggttgaaccgcacca</td><td>probe</td></tr>
<tr><td>vvi|miR-1042|2mut|as|</td><td>agttagcttgaaccccacca</td><td>vvi-miR171h|*+5_mismatch probe</td></tr>
<tr><td>vvi|miR-1043|nat|as|</td><td>cattccaacatccccacctt</td><td>vvi-miR171i|*-5_perfect match probe</td></tr>
<tr><td>vvi|miR-1043|2mut|as|</td><td>cattcaaacatcctcacctt</td><td>vvi-miR171i|*-5_mismatch probe</td></tr>
<tr><td></td><td></td><td>vvi-miR171i|*+5_perfect match</td></tr>
<tr><td>vvi|miR-1044|nat|as|</td><td>tttgattgagccattccaac</td><td>probe</td></tr>
<tr><td>vvi|miR-1044|2mut|as|</td><td>tttgattgactcattccaac</td><td>vvi-miR171i|*+5_mismatch probe</td></tr>
<tr><td></td><td></td><td>vvi-miR172a|*-5_perfect match</td></tr>
<tr><td>vvi|miR-1045|nat|as|</td><td>ttgatgatgctgcatcggca</td><td>probe</td></tr>
<tr><td>vvi|miR-1045|2mut|as|</td><td>ttgatgatcctgaatcggca</td><td>vvi-miR172a|*-5_mismatch probe</td></tr>
<tr><td></td><td></td><td>vvi-miR172a|*+5_perfect match</td></tr>
<tr><td>vvi|miR-1046|nat|as|</td><td>gatgagaatcttgatgatgc</td><td>probe</td></tr>
</table>

vvi|miR-1046|2mut|as|	gatgcgaatcttgataatgc
vvi|miR-1047|nat|as|	cttgatgatgctgcatcggc
vvi|miR-1047|2mut|as|	cttgatgatcctgaatcggc
vvi|miR-1048|nat|as|	gttgagaatcttgatgatgc
vvi|miR-1048|2mut|as|	gttgcgaatcttgataatgc
vvi|miR-1049|nat|as|	cttgatgatgctccatccgc
vvi|miR-1049|2mut|as|	cttgatgatcttccatccgc
vvi|miR-1050|nat|as|	cttgtgaatcttgatgatgc
vvi|miR-1050|2mut|as|	cttgcgaatcttgataatgc
vvi|miR-1051|nat|as|	ttgatgatgctgcatcagct
vvi|miR-1051|2mut|as|	ttgatgatcctgaatcagct
vvi|miR-1052|nat|as|	ggtgtgaatcttgatgatgc
vvi|miR-1052|2mut|as|	ggtgcgaatcttgataatgc
vvi|miR-1053|nat|as|	actaaagagagctccttctt
vvi|miR-1053|2mut|as|	actaaagagcgttccttctt
vvi|miR-1054|nat|as|	tcggactggactaaagagag
vvi|miR-1054|2mut|as|	tcggactcgattaaagagag
vvi|miR-1055|nat|as|	ctgaagaaagctctcttcaa
vvi|miR-1055|2mut|as|	ctgaagaaacttctcttcaa
vvi|miR-1056|nat|as|	atgagtggactgaagaaagc
vvi|miR-1056|2mut|as|	atgagtcgaccgaagaaagc
vvi|miR-1057|nat|as|	tgcaaaggaacccccatttc
vvi|miR-1057|2mut|as|	tgcaaagaaaccctcatttc

vvi-miR172a|*+5_mismatch probe
vvi-miR172b|*-5_perfect match probe
vvi-miR172b|*-5_mismatch probe
vvi-miR172b|*+5_perfect match probe
vvi-miR172b|*+5_mismatch probe
vvi-miR172c|*-5_perfect match probe
vvi-miR172c|*-5_mismatch probe
vvi-miR172c|*+5_perfect match probe
vvi-miR172c|*+5_mismatch probe
vvi-miR172d|*-5_perfect match probe
vvi-miR172d|*-5_mismatch probe
vvi-miR172d|*+5_perfect match probe
vvi-miR172d|*+5_mismatch probe
vvi-miR319b|*-5_perfect match probe
vvi-miR319b|*-5_mismatch probe
vvi-miR319b|*+5_perfect match probe
vvi-miR319b|*+5_mismatch probe
vvi-miR319c|*-5_perfect match probe
vvi-miR319c|*-5_mismatch probe
vvi-miR319c|*+5_perfect match probe
vvi-miR319c|*+5_mismatch probe
vvi-miR319e|*-5_perfect match probe
vvi-miR319e|*-5_mismatch probe

<table>
<tr><td></td><td></td><td>vvi-miR319e|*+5_perfect match</td></tr>
<tr><td>vvi|miR-1058|nat|as|</td><td>gttgttttgggctgcaaagg</td><td>probe</td></tr>
<tr><td>vvi|miR-1058|2mut|as|</td><td>gttgttttgtcctgcaaagg</td><td>vvi-miR319e|*+5_mismatch probe</td></tr>
<tr><td>vvi|miR-1059|nat|as|</td><td>ctgaaggaagctctcttaaa</td><td>vvi-miR319f|*-5_perfect match probe</td></tr>
<tr><td>vvi|miR-1059|2mut|as|</td><td>ctgaaggaacttctcttaaa</td><td>vvi-miR319f|*-5_mismatch probe</td></tr>
<tr><td></td><td></td><td>vvi-miR319f|*+5_perfect match</td></tr>
<tr><td>vvi|miR-1060|nat|as|</td><td>atgagtggactgaaggaagc</td><td>probe</td></tr>
<tr><td>vvi|miR-1060|2mut|as|</td><td>atgagtcgactgaagaaagc</td><td>vvi-miR319f|*+5_mismatch probe</td></tr>
<tr><td></td><td></td><td>vvi-miR319g|*-5_perfect match</td></tr>
<tr><td>vvi|miR-1061|nat|as|</td><td>gactaaaaggagctcttcag</td><td>probe</td></tr>
<tr><td>vvi|miR-1061|2mut|as|</td><td>gactaaaagaagttcttcag</td><td>vvi-miR319g|*-5_mismatch probe</td></tr>
<tr><td></td><td></td><td>vvi-miR319g|*+5_perfect match</td></tr>
<tr><td>vvi|miR-1062|nat|as|</td><td>tctttattggactaaaagga</td><td>probe</td></tr>
<tr><td>vvi|miR-1062|2mut|as|</td><td>tctttattcgcctaaaagga</td><td>vvi-miR319g|*+5_mismatch probe</td></tr>
<tr><td>vvi|miR-1063|nat|as|</td><td>aggatagatagcgccacaca</td><td>vvi-miR390|*-5_perfect match probe</td></tr>
<tr><td>vvi|miR-1063|2mut|as|</td><td>aggatacatagccccacaca</td><td>vvi-miR390|*-5_mismatch probe</td></tr>
<tr><td>vvi|miR-1064|nat|as|</td><td>ccgtgaaactcaggatagat</td><td>vvi-miR390|*+5_perfect match probe</td></tr>
<tr><td>vvi|miR-1064|2mut|as|</td><td>ccgtaaaactcagaatagat</td><td>vvi-miR390|*+5_mismatch probe</td></tr>
<tr><td></td><td></td><td>vvi-miR393a|*-5_perfect match</td></tr>
<tr><td>vvi|miR-1065|nat|as|</td><td>gggatagcatgatccaatgg</td><td>probe</td></tr>
<tr><td>vvi|miR-1065|2mut|as|</td><td>gggatagaatgatcaaatgg</td><td>vvi-miR393a|*-5_mismatch probe</td></tr>
<tr><td></td><td></td><td>vvi-miR393a|*+5_perfect match</td></tr>
<tr><td>vvi|miR-1066|nat|as|</td><td>agagttcctaagggatagca</td><td>probe</td></tr>
<tr><td>vvi|miR-1066|2mut|as|</td><td>agagtttctaagtgatagca</td><td>vvi-miR393a|*+5_mismatch probe</td></tr>
<tr><td></td><td></td><td>vvi-miR393b|*-5_perfect match</td></tr>
<tr><td>vvi|miR-1067|nat|as|</td><td>gggatagcatgatccaaaga</td><td>probe</td></tr>
<tr><td>vvi|miR-1067|2mut|as|</td><td>gggatagaatgatcaaaaga</td><td>vvi-miR393b|*-5_mismatch probe</td></tr>
<tr><td></td><td></td><td>vvi-miR393b|*+5_perfect match</td></tr>
<tr><td>vvi|miR-1068|nat|as|</td><td>aggaatccaaagggatagca</td><td>probe</td></tr>
<tr><td>vvi|miR-1068|2mut|as|</td><td>aggaatcaaaagtgatagca</td><td>vvi-miR393b|*+5_mismatch probe</td></tr>
<tr><td></td><td></td><td>vvi-miR394a|*-5_perfect match</td></tr>
<tr><td>vvi|miR-1069|nat|as|</td><td>atgctggccacctccaaaag</td><td>probe</td></tr>
<tr><td>vvi|miR-1069|2mut|as|</td><td>atgctggtcacctcaaaaag</td><td>vvi-miR394a|*-5_mismatch probe</td></tr>
<tr><td>vvi|miR-1070|nat|as|</td><td>ccactttggcatgctggcca</td><td>vvi-miR394a|*+5_perfect match</td></tr>
</table>

		probe
vvi|miR-1070|2mut|as|	ccactttgccatcctggcca	vvi-miR394a|*+5_mismatch probe
vvi|miR-1071|nat|as|	agtatgcccacctccacaga	vvi-miR394b|*-5_perfect match probe
vvi|miR-1071|2mut|as|	agtatgtccacctcaacaga	vvi-miR394b|*-5_mismatch probe
vvi|miR-1072|nat|as|	cttggttggcagtatgccca	vvi-miR394b|*+5_perfect match probe
vvi|miR-1072|2mut|as|	cttggttgccagtatcccca	vvi-miR394b|*+5_mismatch probe
vvi|miR-1073|nat|as|	atcctggccgcctccaaacg	vvi-miR394c|*-5_perfect match probe
vvi|miR-1073|2mut|as|	atcctggccctctccaaacg	vvi-miR394c|*-5_mismatch probe
vvi|miR-1074|nat|as|	ccaatttggcatcctggccg	vvi-miR394c|*+5_perfect match probe
vvi|miR-1074|2mut|as|	ccaatttgccatcctcgccg	vvi-miR394c|*+5_mismatch probe
vvi|miR-1075|nat|as|	tgatcaagggaactctaggg	vvi-miR395a|*-5_perfect match probe
vvi|miR-1075|2mut|as|	tgatcaagtgaattctaggg	vvi-miR395a|*-5_mismatch probe
vvi|miR-1076|nat|as|	cctagtgaagtgatcaaggg	vvi-miR395a|*+5_perfect match probe
vvi|miR-1076|2mut|as|	cctagcgaagcgatcaaggg	vvi-miR395a|*+5_mismatch probe
vvi|miR-1077|nat|as|	tggtaaggggaactctaggg	vvi-miR395b|*-5_perfect match probe
vvi|miR-1077|2mut|as|	tggtaaggtgaattctaggg	vvi-miR395b|*-5_mismatch probe
vvi|miR-1078|nat|as|	cccagtgaagtggtaagggg	vvi-miR395b|*+5_perfect match probe
vvi|miR-1078|2mut|as|	cccagcgaagtcgtaagggg	vvi-miR395b|*+5_mismatch probe
vvi|miR-1079|nat|as|	tggtcaagggaactctaggg	vvi-miR395c|*-5_perfect match probe
vvi|miR-1079|2mut|as|	tggtcaagtgaattctaggg	vvi-miR395c|*-5_mismatch probe
vvi|miR-1080|nat|as|	cccagtgaagtggtcaaggg	vvi-miR395c|*+5_perfect match probe
vvi|miR-1080|2mut|as|	cccagcgaagtcgtcaaggg	vvi-miR395c|*+5_mismatch probe
vvi|miR-1081|nat|as|	tggtcaggggaactctaggg	vvi-miR395d|*-5_perfect match probe

vvi|miR-1081|2mut|as|	tggtcaggtgaattctaggg	vvi-miR395d|*-5_mismatch probe
		vvi-miR395d|*+5_perfect match probe
vvi|miR-1082|nat|as|	cccaatgaagtggtcagggg	
vvi|miR-1082|2mut|as|	cccaataaagtcgtcagggg	vvi-miR395d|*+5_mismatch probe
		vvi-miR395e|*-5_perfect match probe
vvi|miR-1083|nat|as|	tggtcaagggaactctaggg	
vvi|miR-1083|2mut|as|	tggtcaagtgaattctaggg	vvi-miR395e|*-5_mismatch probe
		vvi-miR395e|*+5_perfect match probe
vvi|miR-1084|nat|as|	cccagtgaagtggtcaaggg	
vvi|miR-1084|2mut|as|	cccagcgaagtcgtcaaggg	vvi-miR395e|*+5_mismatch probe
vvi|miR-1085|nat|as|	tggtcaggggaactctaggg	vvi-miR395f|*-5_perfect match probe
vvi|miR-1085|2mut|as|	tggtcaggtgaattctaggg	vvi-miR395f|*-5_mismatch probe
		vvi-miR395f|*+5_perfect match probe
vvi|miR-1086|nat|as|	cccagtgaagtggtcagggg	
vvi|miR-1086|2mut|as|	cccagcgaagtcgtcagggg	vvi-miR395f|*+5_mismatch probe
		vvi-miR395g|*-5_perfect match probe
vvi|miR-1087|nat|as|	tgctcaggggaactctaggg	
vvi|miR-1087|2mut|as|	tgctcaggtgaattctaggg	vvi-miR395g|*-5_mismatch probe
		vvi-miR395g|*+5_perfect match probe
vvi|miR-1088|nat|as|	cccaatgaagtgctcagggg	
vvi|miR-1088|2mut|as|	cccaataaagtcctcagggg	vvi-miR395g|*+5_mismatch probe
		vvi-miR395h|*-5_perfect match probe
vvi|miR-1089|nat|as|	tggtcaagggaactctaggg	
vvi|miR-1089|2mut|as|	tggtcaagtgaattctaggg	vvi-miR395h|*-5_mismatch probe
		vvi-miR395h|*+5_perfect match probe
vvi|miR-1090|nat|as|	cccagtgaagtggtcaaggg	
vvi|miR-1090|2mut|as|	cccagcgaagtcgtcaaggg	vvi-miR395h|*+5_mismatch probe
vvi|miR-1091|nat|as|	tggtcaggggaactctaggg	vvi-miR395i|*-5_perfect match probe
vvi|miR-1091|2mut|as|	tggtcaggtgaattctaggg	vvi-miR395i|*-5_mismatch probe
		vvi-miR395i|*+5_perfect match probe
vvi|miR-1092|nat|as|	cccagtgaagtggtcagggg	
vvi|miR-1092|2mut|as|	cccagcgaagtcgtcagggg	vvi-miR395i|*+5_mismatch probe
vvi|miR-1093|nat|as|	tggtcaggggaactctaggg	vvi-miR395j|*-5_perfect match probe
vvi|miR-1093|2mut|as|	tggtcaggtgaattctaggg	vvi-miR395j|*-5_mismatch probe

vvi|miR-1094|nat|as|	cccagtgaagtggtcagggg	vvi-miR395j|*+5_perfect match probe
vvi|miR-1094|2mut|as|	cccagcgaagtcgtcagggg	vvi-miR395j|*+5_mismatch probe
vvi|miR-1095|nat|as|	tggtcaagggaactctaggg	vvi-miR395k|*-5_perfect match probe
vvi|miR-1095|2mut|as|	tggtcaagtgaattctaggg	vvi-miR395k|*-5_mismatch probe
vvi|miR-1096|nat|as|	cccagtgaagtggtcaaggg	vvi-miR395k|*+5_perfect match probe
vvi|miR-1096|2mut|as|	cccagcgaagtcgtcaaggg	vvi-miR395k|*+5_mismatch probe
vvi|miR-1097|nat|as|	tggtcaggggaactctaggg	vvi-miR395l|*-5_perfect match probe
vvi|miR-1097|2mut|as|	tggtcaggtgaattctaggg	vvi-miR395l|*-5_mismatch probe
vvi|miR-1098|nat|as|	cccagtgaagtggtcagggg	vvi-miR395l|*+5_perfect match probe
vvi|miR-1098|2mut|as|	cccagcgaagtcgtcagggg	vvi-miR395l|*+5_mismatch probe
vvi|miR-1099|nat|as|	tgttcaagggaactctaggg	vvi-miR395m|*-5_perfect match probe
vvi|miR-1099|2mut|as|	tgttcaagtgaattctaggg	vvi-miR395m|*-5_mismatch probe
vvi|miR-1100|nat|as|	cccagtgaagtgttcaaggg	vvi-miR395m|*+5_perfect match probe
vvi|miR-1100|2mut|as|	cccagcgaagcgttcaaggg	vvi-miR395m|*+5_mismatch probe
vvi|miR-1101|nat|as|	gggttgggggaactctcaag	vvi-miR395n|*-5_perfect match probe
vvi|miR-1101|2mut|as|	gggttgggtgaattctcaag	vvi-miR395n|*-5_mismatch probe
vvi|miR-1102|nat|as|	tcatactggaggggttgggg	vvi-miR395n|*+5_perfect match probe
vvi|miR-1102|2mut|as|	tcatactcgaggtgttgggg	vvi-miR395n|*+5_mismatch probe
vvi|miR-1103|nat|as|	cagctttcttgaactacttt	vvi-miR396a|*-5_perfect match probe
vvi|miR-1103|2mut|as|	cagcttttttaaactacttt	vvi-miR396a|*-5_mismatch probe
vvi|miR-1104|nat|as|	cttttccacagctttcttga	vvi-miR396a|*+5_perfect match probe
vvi|miR-1104|2mut|as|	cttttcaacagttttcttga	vvi-miR396a|*+5_mismatch probe
vvi|miR-1105|nat|as|	agctttcttgaacttcttct	vvi-miR396b|*-5_perfect match probe

vvi|miR-1105|2mut|as|	agctttcttaaatttcttct	vvi-miR396b|*-5_mismatch probe
vvi|miR-1106|nat|as|	atgcttttccacagctttct	vvi-miR396b|*+5_perfect match probe
vvi|miR-1106|2mut|as|	atgcttttcaacagttttct	vvi-miR396b|*+5_mismatch probe
vvi|miR-1107|nat|as|	aactttcctgagctgttggt	vvi-miR396c|*-5_perfect match probe
vvi|miR-1107|2mut|as|	aacttttctgagttgttggt	vvi-miR396c|*-5_mismatch probe
vvi|miR-1108|nat|as|	taatgttccacaactttcct	vvi-miR396c|*+5_perfect match probe
vvi|miR-1108|2mut|as|	taatgttcaaaaactttcct	vvi-miR396c|*+5_mismatch probe
vvi|miR-1109|nat|as|	agctttattgaaccgcaact	vvi-miR396d|*-5_perfect match probe
vvi|miR-1109|2mut|as|	agctttattaaaccccaact	vvi-miR396d|*-5_mismatch probe
vvi|miR-1110|nat|as|	tatcttcccacagctttatt	vvi-miR396d|*+5_perfect match probe
vvi|miR-1110|2mut|as|	tatcttctcacagttttatt	vvi-miR396d|*+5_mismatch probe
vvi|miR-1111|nat|as|	agtgcagcgccaatggaatc	vvi-miR397a|*-5_perfect match probe
vvi|miR-1111|2mut|as|	agtgcagccccaatcgaatc	vvi-miR397a|*-5_mismatch probe
vvi|miR-1112|nat|as|	gacatgattgagtgcagcg	vvi-miR397a|*+5_perfect match probe
vvi|miR-1112|2mut|as|	gacatgattaagtgaagcg	vvi-miR397a|*+5_mismatch probe
vvi|miR-1113|nat|as|	agtgcagcgccaatggaatc	vvi-miR397b|*-5_perfect match probe
vvi|miR-1113|2mut|as|	agtgcagccccaatcgaatc	vvi-miR397b|*-5_mismatch probe
vvi|miR-1114|nat|as|	gacatgattgagtgcagcg	vvi-miR397b|*+5_perfect match probe
vvi|miR-1114|2mut|as|	gacatgattaagtgaagcg	vvi-miR397b|*+5_mismatch probe
vvi|miR-1115|nat|as|	caggtgccactcccttgggg	vvi-miR398a|*-5_perfect match probe
vvi|miR-1115|2mut|as|	caggtgtcactctcttgggg	vvi-miR398a|*-5_mismatch probe
vvi|miR-1116|nat|as|	aaccggtgttctcaggtgcc	vvi-miR398a|*+5_perfect match probe
vvi|miR-1116|2mut|as|	aaccagtgttctcagctgcc	vvi-miR398a|*+5_mismatch probe

		vvi-miR398b|*-5_perfect match
vvi|miR-1117|nat|as|	ctcaggtcacacctgtagga	probe
vvi|miR-1117|2mut|as|	ctcagctcacatctgtagga	vvi-miR398b|*-5_mismatch probe
		vvi-miR398b|*+5_perfect match
vvi|miR-1118|nat|as|	gggcatgtgattctcaggtc	probe
vvi|miR-1118|2mut|as|	gggcatgcgattctccggtc	vvi-miR398b|*+5_mismatch probe
		vvi-miR398c|*-5_perfect match
vvi|miR-1119|nat|as|	ctcaggtcactcctgtagga	probe
vvi|miR-1119|2mut|as|	ctcagctcacttctgtagga	vvi-miR398c|*-5_mismatch probe
		vvi-miR398c|*+5_perfect match
vvi|miR-1120|nat|as|	gggcatgtgattctcaggtc	probe
vvi|miR-1120|2mut|as|	gggcatgcgattctccggtc	vvi-miR398c|*+5_mismatch probe
		vvi-miR399a|*-5_perfect match
vvi|miR-1121|nat|as|	aggagaatcacactgttatt	probe
vvi|miR-1121|2mut|as|	aggagaatccccctgttatt	vvi-miR399a|*-5_mismatch probe
		vvi-miR399a|*+5_perfect match
vvi|miR-1122|nat|as|	atctctgccaaaggagaatc	probe
vvi|miR-1122|2mut|as|	atctctgtcaaagaagaatc	vvi-miR399a|*+5_mismatch probe
		vvi-miR399b|*-5_perfect match
vvi|miR-1123|nat|as|	gaaagaggtgccctatgatt	probe
vvi|miR-1123|2mut|as|	gaaagaggtctcctatgatt	vvi-miR399b|*-5_mismatch probe
		vvi-miR399b|*+5_perfect match
vvi|miR-1124|nat|as|	gtgcctgccaagaaagaggt	probe
vvi|miR-1124|2mut|as|	gtgcctgtcaagaaacaggt	vvi-miR399b|*+5_mismatch probe
		vvi-miR399c|*-5_perfect match
vvi|miR-1125|nat|as|	gtgaaagaggccctgagacc	probe
vvi|miR-1125|2mut|as|	gtgaaagagctcctgagacc	vvi-miR399c|*-5_mismatch probe
		vvi-miR399c|*+5_perfect match
vvi|miR-1126|nat|as|	tcacctaccaagtgaaagag	probe
vvi|miR-1126|2mut|as|	tcacctacaaagcgaaagag	vvi-miR399c|*+5_mismatch probe
		vvi-miR399d|*-5_perfect match
vvi|miR-1127|nat|as|	aaaagaaatctgctctataa	probe
vvi|miR-1127|2mut|as|	aaaagaaatttcctctataa	vvi-miR399d|*-5_mismatch probe
vvi|miR-1128|nat|as|	ccatctgccaaaagaaatct	vvi-miR399d|*+5_perfect match

probe
vvi|miR-1128|2mut|as| ccatctgtcaaaacaaatct vvi-miR399d|*+5_mismatch probe
vvi-miR399e|*-5_perfect match
vvi|miR-1129|nat|as| aaagataatttgccctgtaa probe
vvi|miR-1129|2mut|as| aaagctaatttgtcctgtaa vvi-miR399e|*-5_mismatch probe
vvi-miR399e|*+5_perfect match
vvi|miR-1130|nat|as| ctgcctgccaaaagataatt probe
vvi|miR-1130|2mut|as| ctgcctgtcaaaacataatt vvi-miR399e|*+5_mismatch probe
vvi|miR-1131|nat|as| tttggccctctaatgcaac vvi-miR399f|*-5_perfect match probe
vvi|miR-1131|2mut|as| tttggtcctctaatccaac vvi-miR399f|*-5_mismatch probe
vvi-miR399f|*+5_perfect match
vvi|miR-1132|nat|as| cttcatgccaaagcagattt probe
vvi|miR-1132|2mut|as| cttcatgtcaaagaagattt vvi-miR399f|*+5_mismatch probe
vvi-miR399g|*-5_perfect match
vvi|miR-1133|nat|as| aatggagtattgcccagcaa probe
vvi|miR-1133|2mut|as| aatgaagtattgtccagcaa vvi-miR399g|*-5_mismatch probe
vvi-miR399g|*+5_perfect match
vvi|miR-1134|nat|as| ccaactgccaatggagtatt probe
vvi|miR-1134|2mut|as| ccaactgtcaatcgagtatt vvi-miR399g|*+5_mismatch probe
vvi-miR399h|*-5_perfect match
vvi|miR-1135|nat|as| aggaggattgcactgttatt probe
vvi|miR-1135|2mut|as| aggaggattcaactgttatt vvi-miR399h|*-5_mismatch probe
vvi-miR399h|*+5_perfect match
vvi|miR-1136|nat|as| ctttctgccaaaggaggatt probe
vvi|miR-1136|2mut|as| ctttctgtcaaagaaggatt vvi-miR399h|*+5_mismatch probe
vvi|miR-1137|nat|as| aggagagaagccctacaact vvi-miR399i|*-5_perfect match probe
vvi|miR-1137|2mut|as| aggagagaactcctacaact vvi-miR399i|*-5_mismatch probe
vvi-miR399i|*+5_perfect match
vvi|miR-1138|nat|as| ctcctgccagaaggagagaa probe
vvi|miR-1138|2mut|as| ctcctgtcagaagaagagaa vvi-miR399i|*+5_mismatch probe
vvi-miR403a|*-5_perfect match
vvi|miR-1139|nat|as| attcacgcacaaacttgaga probe
vvi|miR-1139|2mut|as| attcacccacaaatttgaga vvi-miR403a|*-5_mismatch probe
vvi|miR-1140|nat|as| tggggtttgattcacgcaca vvi-miR403a|*+5_perfect match

		probe
vvi|miR-1140|2mut|as|	tgggctttgattcacccaca	vvi-miR403a|*+5_mismatch probe
		vvi-miR403b|*-5_perfect match
vvi|miR-1141|nat|as|	tcgcgcacaaactcgaggtt	probe
vvi|miR-1141|2mut|as|	tcgcccacaaactagaggtt	vvi-miR403b|*-5_mismatch probe
		vvi-miR403b|*+5_perfect match
vvi|miR-1142|nat|as|	aggcgttggattcgcgcaca	probe
vvi|miR-1142|2mut|as|	aggccttggattcgcccaca	vvi-miR403b|*+5_mismatch probe
		vvi-miR403c|*-5_perfect match
vvi|miR-1143|nat|as|	tcacgcacaaacttgagatg	probe
vvi|miR-1143|2mut|as|	tcacccacaaacttaagatg	vvi-miR403c|*-5_mismatch probe
		vvi-miR403c|*+5_perfect match
vvi|miR-1144|nat|as|	tggggtttgattcacgcaca	probe
vvi|miR-1144|2mut|as|	tgggctttgattcacccaca	vvi-miR403c|*+5_mismatch probe
		vvi-miR403d|*-5_perfect match
vvi|miR-1145|nat|as|	tcgcgcacaaactcgaggtt	probe
vvi|miR-1145|2mut|as|	tcgcccacaaactagaggtt	vvi-miR403d|*-5_mismatch probe
		vvi-miR403d|*+5_perfect match
vvi|miR-1146|nat|as|	ggttggattcgcgcacaaac	probe
vvi|miR-1146|2mut|as|	ggttggattagcccacaaac	vvi-miR403d|*+5_mismatch probe
		vvi-miR403e|*-5_perfect match
vvi|miR-1147|nat|as|	tcacgcacaaacttgagatg	probe
vvi|miR-1147|2mut|as|	tcacccacaaacttaagatg	vvi-miR403e|*-5_mismatch probe
		vvi-miR403e|*+5_perfect match
vvi|miR-1148|nat|as|	tggggtttgattcacgcaca	probe
vvi|miR-1148|2mut|as|	tgggctttgattcacccaca	vvi-miR403e|*+5_mismatch probe
vvi|miR-1149|nat|as|	gtcacgcacaaactcctcat	vvi-miR403f|*-5_perfect match probe
vvi|miR-1149|2mut|as|	gtcacccacaaacttctcat	vvi-miR403f|*-5_mismatch probe
		vvi-miR403f|*+5_perfect match
vvi|miR-1150|nat|as|	cggttttagagtcacgcaca	probe
vvi|miR-1150|2mut|as|	cggttttagcgtcacccaca	vvi-miR403f|*+5_mismatch probe
vvi|miR-1151|nat|as|	actacctcgtccccgtcttc	vvi-miR408|*-5_perfect match probe
vvi|miR-1151|2mut|as|	actacctcctcccagtcttc	vvi-miR408|*-5_mismatch probe
vvi|miR-1152|nat|as|	catccatgcactacctcgtc	vvi-miR408|*+5_perfect match probe

vvi|miR-1152|2mut|as|	catccatgaactatctcgtc	vvi-miR408|*+5_mismatch probe
vvi|miR-1153|nat|as|	cccactgtcttccaactttc	vvi-miR477a|*-5_perfect match probe
vvi|miR-1153|2mut|as|	cccactctcttcaaactttc	vvi-miR477a|*-5_mismatch probe
vvi|miR-1154|nat|as|	ttccaaggtcccccactgtc	vvi-miR477a|*+5_perfect match probe
vvi|miR-1154|2mut|as|	ttccaagctccctcactgtc	vvi-miR477a|*+5_mismatch probe
vvi|miR-1155|nat|as|	ttggttcggctcgtctttga	vvi-miR479|*-5_perfect match probe
vvi|miR-1155|2mut|as|	ttggttcgcctcctctttga	vvi-miR479|*-5_mismatch probe
vvi|miR-1156|nat|as|	caagagtgatattggttcgg	vvi-miR479|*+5_perfect match probe
vvi|miR-1156|2mut|as|	caagagcgatattcgttcgg	vvi-miR479|*+5_mismatch probe
vvi|miR-1157|nat|as|	ctactctccaattcccaaac	vvi-miR482a|*-5_perfect match probe
vvi|miR-1157|2mut|as|	ctactctcaaattctcaaac	vvi-miR482a|*-5_mismatch probe
vvi|miR-1158|nat|as|	gctttcctactctccaattc	vvi-miR482a|*+5_perfect match probe
vvi|miR-1158|2mut|as|	gcttttctactctcaaattc	vvi-miR482a|*+5_mismatch probe
vvi|miR-1159|nat|as|	gagagagagcacgcctgtac	vvi-miR535a|*-5_perfect match probe
vvi|miR-1159|2mut|as|	gagagagagaacccctgtac	vvi-miR535a|*-5_mismatch probe
vvi|miR-1160|nat|as|	ttgtatgacagcgagagaga	vvi-miR535a|*+5_perfect match probe
vvi|miR-1160|2mut|as|	ttgtatgaaagagagagaga	vvi-miR535a|*+5_mismatch probe
vvi|miR-1161|nat|as|	gagagagagcacgcctgtac	vvi-miR535b|*-5_perfect match probe
vvi|miR-1161|2mut|as|	gagagagagaacccctgtac	vvi-miR535b|*-5_mismatch probe
vvi|miR-1162|nat|as|	ttgtatgacagcgagagaga	vvi-miR535b|*+5_perfect match probe
vvi|miR-1162|2mut|as|	ttgtatgaaagagagagaga	vvi-miR535b|*+5_mismatch probe
vvi|miR-1163|nat|as|	gagagagagcacgcctgtac	vvi-miR535c|*-5_perfect match probe
vvi|miR-1163|2mut|as|	gagagagagaacccctgtac	vvi-miR535c|*-5_mismatch probe
vvi|miR-1164|nat|as|	ttgtatgacagcgagagaga	vvi-miR535c|*+5_perfect match probe

vvi|miR-1164|2mut|as|	ttgtatgaaagagagagaga	vvi-miR535c|*+5_mismatch probe
vvi|miR-1165|nat|as|	gacagagagcacgcctgtac	vvi-miR535d|*-5_perfect match probe
vvi|miR-1165|2mut|as|	gacagagagaacccctgtac	vvi-miR535d|*-5_mismatch probe
vvi|miR-1166|nat|as|	ttgtatgacagcgacagaga	vvi-miR535d|*+5_perfect match probe
vvi|miR-1166|2mut|as|	ttgtatgaaagagacagaga	vvi-miR535d|*+5_mismatch probe
vvi|miR-1167|nat|as|	gacagagagcacgcctgtac	vvi-miR535e|*-5_perfect match probe
vvi|miR-1167|2mut|as|	gacagagagaacccctgtac	vvi-miR535e|*-5_mismatch probe
vvi|miR-1168|nat|as|	ttgtatgacagcgacagaga	vvi-miR535e|*+5_perfect match probe
vvi|miR-1168|2mut|as|	ttgtatgaaagagacagaga	vvi-miR535e|*+5_mismatch probe
vvi|miR-1169|nat|as|	caaatgagcatctcaacaac	vvi-miR828a|*-5_perfect match probe
vvi|miR-1169|2mut|as|	caaatgagaatctaaacaac	vvi-miR828a|*-5_mismatch probe
vvi|miR-1170|nat|as|	ggttgcttcctcaaatgagc	vvi-miR828a|*+5_perfect match probe
vvi|miR-1170|2mut|as|	ggttccttcctaaaatgagc	vvi-miR828a|*+5_mismatch probe
vvi|miR-1171|nat|as|	ggaaatgactatccaggttg	vvi-miR828b|*-5_perfect match probe
vvi|miR-1171|2mut|as|	ggaaatgcctatcaaggttg	vvi-miR828b|*-5_mismatch probe
vvi|miR-1172|nat|as|	tggttttgcaggaaatgact	vvi-miR828b|*+5_perfect match probe
vvi|miR-1172|2mut|as|	tggttttgaagaaaatgact	vvi-miR828b|*+5_mismatch probe
vvi|miR-1173|nat|as|	catgaaggaactaatcaagt	vvi-miR845a|*-5_perfect match probe
vvi|miR-1173|2mut|as|	catgaagaaactaataaagt	vvi-miR845a|*-5_mismatch probe
vvi|miR-1174|nat|as|	tgcaataactcatgaaggaa	vvi-miR845a|*+5_perfect match probe
vvi|miR-1174|2mut|as|	tgcaataattcataaaggaa	vvi-miR845a|*+5_mismatch probe
vvi|miR-1175|nat|as|	catgaaggaactaatcaagt	vvi-miR845b|*-5_perfect match probe
vvi|miR-1175|2mut|as|	catgaagaaactaataaagt	vvi-miR845b|*-5_mismatch probe

<table>
<tr><td></td><td></td><td>vvi-miR845b|*+5_perfect match</td></tr>
<tr><td>vvi|miR-1176|nat|as|</td><td>gaaataactcatgaaggaac</td><td>probe</td></tr>
<tr><td>vvi|miR-1176|2mut|as|</td><td>gaaataactaatgaacgaac</td><td>vvi-miR845b|*+5_mismatch probe</td></tr>
<tr><td></td><td></td><td>vvi-miR845c|*-5_perfect match</td></tr>
<tr><td>vvi|miR-1177|nat|as|</td><td>cagacaaccaaaacacataa</td><td>probe</td></tr>
<tr><td>vvi|miR-1177|2mut|as|</td><td>cagacaacaaaaacccataa</td><td>vvi-miR845c|*-5_mismatch probe</td></tr>
<tr><td></td><td></td><td>vvi-miR845c|*+5_perfect match</td></tr>
<tr><td>vvi|miR-1178|nat|as|</td><td>acatgaaactcagacaacca</td><td>probe</td></tr>
<tr><td>vvi|miR-1178|2mut|as|</td><td>acataaaactcagaaaacca</td><td>vvi-miR845c|*+5_mismatch probe</td></tr>
<tr><td></td><td></td><td>vvi-miR845d|*-5_perfect match</td></tr>
<tr><td>vvi|miR-1179|nat|as|</td><td>tagggataattaatgttggt</td><td>probe</td></tr>
<tr><td>vvi|miR-1179|2mut|as|</td><td>taggaataattaatcttggt</td><td>vvi-miR845d|*-5_mismatch probe</td></tr>
<tr><td></td><td></td><td>vvi-miR845d|*+5_perfect match</td></tr>
<tr><td>vvi|miR-1180|nat|as|</td><td>tcccatggccctagggataa</td><td>probe</td></tr>
<tr><td>vvi|miR-1180|2mut|as|</td><td>tcccatggtcctagtgataa</td><td>vvi-miR845d|*+5_mismatch probe</td></tr>
<tr><td></td><td></td><td>vvi-miR845e|*-5_perfect match</td></tr>
<tr><td>vvi|miR-1181|nat|as|</td><td>tagggataattaatgtgcgt</td><td>probe</td></tr>
<tr><td>vvi|miR-1181|2mut|as|</td><td>taggaataattaatgcgcgt</td><td>vvi-miR845e|*-5_mismatch probe</td></tr>
<tr><td></td><td></td><td>vvi-miR845e|*+5_perfect match</td></tr>
<tr><td>vvi|miR-1182|nat|as|</td><td>tcccatggccctagggataa</td><td>probe</td></tr>
<tr><td>vvi|miR-1182|2mut|as|</td><td>tcccatggtcctagtgataa</td><td>vvi-miR845e|*+5_mismatch probe</td></tr>
<tr><td></td><td></td><td>vvi-miR156a|*-10_perfect match</td></tr>
<tr><td>vvi|miR-1183|nat|as|</td><td>gagagagcacactcccaaga</td><td>probe</td></tr>
<tr><td>vvi|miR-1183|2mut|as|</td><td>gagagagaacactctcaaga</td><td>vvi-miR156a|*-10_mismatch probe</td></tr>
<tr><td></td><td></td><td>vvi-miR156a|*+10_perfect match</td></tr>
<tr><td>vvi|miR-1184|nat|as|</td><td>gtgtgatgatgacagaagaa</td><td>probe</td></tr>
<tr><td>vvi|miR-1184|2mut|as|</td><td>gtgtgataatgaaagaagaa</td><td>vvi-miR156a|*+10_mismatch probe</td></tr>
<tr><td></td><td></td><td>vvi-miR156b|*-10_perfect match</td></tr>
<tr><td>vvi|miR-1185|nat|as|</td><td>aaatgagcacgcaaaagcaa</td><td>probe</td></tr>
<tr><td>vvi|miR-1185|2mut|as|</td><td>aaatgagaacccaaaagcaa</td><td>vvi-miR156b|*-10_mismatch probe</td></tr>
<tr><td></td><td></td><td>vvi-miR156b|*+10_perfect match</td></tr>
<tr><td>vvi|miR-1186|nat|as|</td><td>ctggaagctgacagaaagag</td><td>probe</td></tr>
<tr><td>vvi|miR-1186|2mut|as|</td><td>ctggaagttgaaagaaagag</td><td>vvi-miR156b|*+10_mismatch probe</td></tr>
<tr><td>vvi|miR-1187|nat|as|</td><td>gagtgagcacgcagagcttc</td><td>vvi-miR156c|*-10_perfect match</td></tr>
</table>

<table>
<tr><td></td><td></td><td>probe</td></tr>
<tr><td>vvi|miR-1187|2mut|as|</td><td>gagtgagaacccagagcttc</td><td>vvi-miR156c|*-10_mismatch probe</td></tr>
<tr><td></td><td></td><td>vvi-miR156c|*+10_perfect match</td></tr>
<tr><td>vvi|miR-1188|nat|as|</td><td>gaggacagatgacagataga</td><td>probe</td></tr>
<tr><td>vvi|miR-1188|2mut|as|</td><td>gaggaaagatgaaagataga</td><td>vvi-miR156c|*+10_mismatch probe</td></tr>
<tr><td></td><td></td><td>vvi-miR156d|*-10_perfect match</td></tr>
<tr><td>vvi|miR-1189|nat|as|</td><td>aggtgagcacgcaccggcat</td><td>probe</td></tr>
<tr><td>vvi|miR-1189|2mut|as|</td><td>aggtgagaacccaccggcat</td><td>vvi-miR156d|*-10_mismatch probe</td></tr>
<tr><td></td><td></td><td>vvi-miR156d|*+10_perfect match</td></tr>
<tr><td>vvi|miR-1190|nat|as|</td><td>cctgaagctgacagaaagag</td><td>probe</td></tr>
<tr><td>vvi|miR-1190|2mut|as|</td><td>cctgaagttgaaagaaagag</td><td>vvi-miR156d|*+10_mismatch probe</td></tr>
<tr><td></td><td></td><td>vvi-miR156e|*-10_perfect match</td></tr>
<tr><td>vvi|miR-1191|nat|as|</td><td>gagtaagcacacagagcttc</td><td>probe</td></tr>
<tr><td>vvi|miR-1191|2mut|as|</td><td>gagtaagaacacagaccttc</td><td>vvi-miR156e|*-10_mismatch probe</td></tr>
<tr><td></td><td></td><td>vvi-miR156e|*+10_perfect match</td></tr>
<tr><td>vvi|miR-1192|nat|as|</td><td>cttgaggggtgacagatagg</td><td>probe</td></tr>
<tr><td>vvi|miR-1192|2mut|as|</td><td>cttgaggtgtgaaagatagg</td><td>vvi-miR156e|*+10_mismatch probe</td></tr>
<tr><td></td><td></td><td>vvi-miR156f|*-10_perfect match</td></tr>
<tr><td>vvi|miR-1193|nat|as|</td><td>gagagaggacaagactgctc</td><td>probe</td></tr>
<tr><td>vvi|miR-1193|2mut|as|</td><td>gagagagaacaagcctgctc</td><td>vvi-miR156f|*-10_mismatch probe</td></tr>
<tr><td>vvi|miR-1194|nat|as|</td><td>gacaggagaagagagaggac</td><td>vvi-miR156f|*0_perfect match probe</td></tr>
<tr><td>vvi|miR-1194|2mut|as|</td><td>gacagaagaagagagcggac</td><td>vvi-miR156f|*0_mismatch probe</td></tr>
<tr><td></td><td></td><td>vvi-miR156f|*+10_perfect match</td></tr>
<tr><td>vvi|miR-1195|nat|as|</td><td>aagttctgttgacaggagaa</td><td>probe</td></tr>
<tr><td>vvi|miR-1195|2mut|as|</td><td>aagttctcttgacagaagaa</td><td>vvi-miR156f|*+10_mismatch probe</td></tr>
<tr><td></td><td></td><td>vvi-miR156g|*-10_perfect match</td></tr>
<tr><td>vvi|miR-1196|nat|as|</td><td>tagagagcacaaaggagtga</td><td>probe</td></tr>
<tr><td>vvi|miR-1196|2mut|as|</td><td>tagagagaacaaagaagtga</td><td>vvi-miR156g|*-10_mismatch probe</td></tr>
<tr><td>vvi|miR-1197|nat|as|</td><td>gacagaagtctagagagcac</td><td>vvi-miR156g|*0_perfect match probe</td></tr>
<tr><td>vvi|miR-1197|2mut|as|</td><td>gacagaagcctagagcgcac</td><td>vvi-miR156g|*0_mismatch probe</td></tr>
<tr><td></td><td></td><td>vvi-miR156g|*+10_perfect match</td></tr>
<tr><td>vvi|miR-1198|nat|as|</td><td>ctgaggtgatgacagaagtc</td><td>probe</td></tr>
<tr><td>vvi|miR-1198|2mut|as|</td><td>ctgagctgatgaaagaagtc</td><td>vvi-miR156g|*+10_mismatch probe</td></tr>
<tr><td>vvi|miR-1199|nat|as|</td><td>cagaaagaactcagctgatc</td><td>vvi-miR156h|*-10_perfect match</td></tr>
</table>

<table>
<tr><td></td><td></td><td>probe</td></tr>
<tr><td>vvi|miR-1199|2mut|as|</td><td>cagaaagaattcagttgatc</td><td>vvi-miR156h|*-10_mismatch probe</td></tr>
<tr><td></td><td></td><td>vvi-miR156h|*+10_perfect match</td></tr>
<tr><td>vvi|miR-1200|nat|as|</td><td>gcaggacacaatgaaaggcg</td><td>probe</td></tr>
<tr><td>vvi|miR-1200|2mut|as|</td><td>gcagaacacaataaaaggcg</td><td>vvi-miR156h|*+10_mismatch probe</td></tr>
<tr><td></td><td></td><td>vvi-miR156i|*-10_perfect match</td></tr>
<tr><td>vvi|miR-1201|nat|as|</td><td>tagagagcacaaaggagtga</td><td>probe</td></tr>
<tr><td>vvi|miR-1201|2mut|as|</td><td>tagagagaacaaagaagtga</td><td>vvi-miR156i|*-10_mismatch probe</td></tr>
<tr><td></td><td></td><td>vvi-miR156i|*+10_perfect match</td></tr>
<tr><td>vvi|miR-1202|nat|as|</td><td>ttaaggtgatgacagaagaa</td><td>probe</td></tr>
<tr><td>vvi|miR-1202|2mut|as|</td><td>ttaagctgatgaaagaagaa</td><td>vvi-miR156i|*+10_mismatch probe</td></tr>
<tr><td></td><td></td><td>vvi-miR159a|*-10_perfect match</td></tr>
<tr><td>vvi|miR-1203|nat|as|</td><td>aggagctcccataaaccc</td><td>probe</td></tr>
<tr><td>vvi|miR-1203|2mut|as|</td><td>aggagctctaataaaccc</td><td>vvi-miR159a|*-10_mismatch probe</td></tr>
<tr><td>vvi|miR-1204|nat|as|</td><td>tggagcgtaaaggagctccc</td><td>vvi-miR159a|*0_perfect match probe</td></tr>
<tr><td>vvi|miR-1204|2mut|as|</td><td>tggagagtaaaggagttccc</td><td>vvi-miR159a|*0_mismatch probe</td></tr>
<tr><td></td><td></td><td>vvi-miR159a|*+10_perfect match</td></tr>
<tr><td>vvi|miR-1205|nat|as|</td><td>ctttcagaactggagcgtaa</td><td>probe</td></tr>
<tr><td>vvi|miR-1205|2mut|as|</td><td>ctttccgaactggagagtaa</td><td>vvi-miR159a|*+10_mismatch probe</td></tr>
<tr><td></td><td></td><td>vvi-miR159b|*-10_perfect match</td></tr>
<tr><td>vvi|miR-1206|nat|as|</td><td>gaagctcccacaagccc</td><td>probe</td></tr>
<tr><td>vvi|miR-1206|2mut|as|</td><td>gaagctctcccaagccc</td><td>vvi-miR159b|*-10_mismatch probe</td></tr>
<tr><td>vvi|miR-1207|nat|as|</td><td>ggagtgtaaagaagctccca</td><td>vvi-miR159b|*0_perfect match probe</td></tr>
<tr><td>vvi|miR-1207|2mut|as|</td><td>ggagcgtaaagaagttccca</td><td>vvi-miR159b|*0_mismatch probe</td></tr>
<tr><td></td><td></td><td>vvi-miR159b|*+10_perfect match</td></tr>
<tr><td>vvi|miR-1208|nat|as|</td><td>tttcagttctggagtgtaaa</td><td>probe</td></tr>
<tr><td>vvi|miR-1208|2mut|as|</td><td>tttcagttcccgagtgtaaa</td><td>vvi-miR159b|*+10_mismatch probe</td></tr>
<tr><td></td><td></td><td>vvi-miR159c|*-10_perfect match</td></tr>
<tr><td>vvi|miR-1209|nat|as|</td><td>aggagctccactccaaaa</td><td>probe</td></tr>
<tr><td>vvi|miR-1209|2mut|as|</td><td>aggagttccactcaaaaa</td><td>vvi-miR159c|*-10_mismatch probe</td></tr>
<tr><td>vvi|miR-1210|nat|as|</td><td>ttggacttcaaggagctcca</td><td>vvi-miR159c|*0_perfect match probe</td></tr>
<tr><td>vvi|miR-1210|2mut|as|</td><td>ttggccttcaaggagttcca</td><td>vvi-miR159c|*0_mismatch probe</td></tr>
<tr><td></td><td></td><td>vvi-miR159c|*+10_perfect match</td></tr>
<tr><td>vvi|miR-1211|nat|as|</td><td>agaccctctattggacttca</td><td>probe</td></tr>
</table>

vvi|miR-1211|2mut|as|	agactctctattcgacttca	vvi-miR159c|*+10_mismatch probe
		vvi-miR160a|*-10_perfect match
vvi|miR-1212|nat|as|	tctgatgccaactaggaaga	probe
vvi|miR-1212|2mut|as|	tctgatgtcaactagaaaga	vvi-miR160a|*-10_mismatch probe
vvi|miR-1213|nat|as|	gcatgactcctctgatgcca	vvi-miR160a|*0_perfect match probe
vvi|miR-1213|2mut|as|	gcatgacttctccgatgcca	vvi-miR160a|*0_mismatch probe
		vvi-miR160a|*+10_perfect match
vvi|miR-1214|nat|as|	gttggggcctgcatgactcc	probe
vvi|miR-1214|2mut|as|	gttggggtctgaatgactcc	vvi-miR160a|*+10_mismatch probe
		vvi-miR160b|*-10_perfect match
vvi|miR-1215|nat|as|	cctcatgccaaataggaagg	probe
vvi|miR-1215|2mut|as|	cctcatgtcaaatagaaagg	vvi-miR160b|*-10_mismatch probe
vvi|miR-1216|nat|as|	gcttgactcccctcatgcca	vvi-miR160b|*0_perfect match probe
vvi|miR-1216|2mut|as|	gcttgactcttctcatgcca	vvi-miR160b|*0_mismatch probe
		vvi-miR160b|*+10_perfect match
vvi|miR-1217|nat|as|	tgtttcgcctgcttgactcc	probe
vvi|miR-1217|2mut|as|	tgtttcccctccttgactcc	vvi-miR160b|*+10_mismatch probe
		vvi-miR160c|*-10_perfect match
vvi|miR-1218|nat|as|	tcgcacgccacccgcaaata	probe
vvi|miR-1218|2mut|as|	tcgcaccccacccccaaata	vvi-miR160c|*-10_mismatch probe
vvi|miR-1219|nat|as|	gcttggctcctcgcacgcca	vvi-miR160c|*0_perfect match probe
vvi|miR-1219|2mut|as|	gcttgcctcctcccacgcca	vvi-miR160c|*0_mismatch probe
		vvi-miR160c|*+10_perfect match
vvi|miR-1220|nat|as|	tggtgggtatgcttggctcc	probe
vvi|miR-1220|2mut|as|	tggtgtgtatgcttgcctcc	vvi-miR160c|*+10_mismatch probe
		vvi-miR160d|*-10_perfect match
vvi|miR-1221|nat|as|	tcgcacgccatccgcgaagg	probe
vvi|miR-1221|2mut|as|	tcgcaccccatccgagaagg	vvi-miR160d|*-10_mismatch probe
vvi|miR-1222|nat|as|	gcatggctcctcgcacgcca	vvi-miR160d|*0_perfect match probe
vvi|miR-1222|2mut|as|	gcatgcctcctcccacgcca	vvi-miR160d|*0_mismatch probe
		vvi-miR160d|*+10_perfect match
vvi|miR-1223|nat|as|	caagatgcatgcatggctcc	probe
vvi|miR-1223|2mut|as|	caagatgaatgcatgcctcc	vvi-miR160d|*+10_mismatch probe
vvi|miR-1224|nat|as|	cctcatgccaaataggaagg	vvi-miR160e|*-10_perfect match

<table>
<tr><td></td><td></td><td>probe</td></tr>
<tr><td>vvi|miR-1224|2mut|as|</td><td>cctcatgtcaaatagaaagg</td><td>vvi-miR160e|*-10_mismatch probe</td></tr>
<tr><td>vvi|miR-1225|nat|as|</td><td>gcttgactcccctcatgcca</td><td>vvi-miR160e|*0_perfect match probe</td></tr>
<tr><td>vvi|miR-1225|2mut|as|</td><td>gcttgactcttctcatgcca</td><td>vvi-miR160e|*0_mismatch probe</td></tr>
<tr><td>vvi|miR-1226|nat|as|</td><td>tgtttcgcctgcttgactcc</td><td>vvi-miR160e|*+10_perfect match probe</td></tr>
<tr><td>vvi|miR-1226|2mut|as|</td><td>tgtttcccctccttgactcc</td><td>vvi-miR160e|*+10_mismatch probe</td></tr>
<tr><td>vvi|miR-1227|nat|as|</td><td>tcatacgccatccacgaagg</td><td>vvi-miR160f|*-10_perfect match probe</td></tr>
<tr><td>vvi|miR-1227|2mut|as|</td><td>tcataccccatccaagaagg</td><td>vvi-miR160f|*-10_mismatch probe</td></tr>
<tr><td>vvi|miR-1228|nat|as|</td><td>gcatggctcctcatacgcca</td><td>vvi-miR160f|*0_perfect match probe</td></tr>
<tr><td>vvi|miR-1228|2mut|as|</td><td>gcatgcctcctcataagcca</td><td>vvi-miR160f|*0_mismatch probe</td></tr>
<tr><td>vvi|miR-1229|nat|as|</td><td>tggggcatatgcatggctcc</td><td>vvi-miR160f|*+10_perfect match probe</td></tr>
<tr><td>vvi|miR-1229|2mut|as|</td><td>tgggccatatgcatgcctcc</td><td>vvi-miR160f|*+10_mismatch probe</td></tr>
<tr><td>vvi|miR-1230|nat|as|</td><td>ctgcatccagtgacttca</td><td>vvi-miR162|*-10_perfect match probe</td></tr>
<tr><td>vvi|miR-1230|2mut|as|</td><td>ctgcatcaagcgacttca</td><td>vvi-miR162|*-10_mismatch probe</td></tr>
<tr><td>vvi|miR-1231|nat|as|</td><td>cgatgaaccgctgcatccag</td><td>vvi-miR162|*0_perfect match probe</td></tr>
<tr><td>vvi|miR-1231|2mut|as|</td><td>cgatgaacccctgaatccag</td><td>vvi-miR162|*0_mismatch probe</td></tr>
<tr><td>vvi|miR-1232|nat|as|</td><td>aggaagagatcgatgaaccg</td><td>vvi-miR162|*+10_perfect match probe</td></tr>
<tr><td>vvi|miR-1232|2mut|as|</td><td>aggaagcgatagatgaaccg</td><td>vvi-miR162|*+10_mismatch probe</td></tr>
<tr><td>vvi|miR-1233|nat|as|</td><td>gagcacgtgcaagcctgtgg</td><td>vvi-miR164a|*-10_perfect match probe</td></tr>
<tr><td>vvi|miR-1233|2mut|as|</td><td>gagcacgtgaaagtctgtgg</td><td>vvi-miR164a|*-10_mismatch probe</td></tr>
<tr><td>vvi|miR-1234|nat|as|</td><td>tggagaaggggagcacgtgc</td><td>vvi-miR164a|*0_perfect match probe</td></tr>
<tr><td>vvi|miR-1234|2mut|as|</td><td>tggagaaggtgagaacgtgc</td><td>vvi-miR164a|*0_mismatch probe</td></tr>
<tr><td>vvi|miR-1235|nat|as|</td><td>gaacccatgttggagaaggg</td><td>vvi-miR164a|*+10_perfect match probe</td></tr>
<tr><td>vvi|miR-1235|2mut|as|</td><td>gaactcatgttcgagaaggg</td><td>vvi-miR164a|*+10_mismatch probe</td></tr>
<tr><td>vvi|miR-1236|nat|as|</td><td>agggcacatgtaaaatctca</td><td>vvi-miR164b|*-10_perfect match probe</td></tr>
<tr><td>vvi|miR-1236|2mut|as|</td><td>agggaacatgtaaaacctca</td><td>vvi-miR164b|*-10_mismatch probe</td></tr>
</table>

vvi|miR-1237|nat|as|	tgggagagccagggcacatg	vvi-miR164b|*0_perfect match probe
vvi|miR-1237|2mut|as|	tgggagagtcaggccacatg	vvi-miR164b|*0_mismatch probe
		vvi-miR164b|*+10_perfect match
vvi|miR-1238|nat|as|	aggatccaagtgggagagcc	probe
vvi|miR-1238|2mut|as|	aggatcaaagtgtgagagcc	vvi-miR164b|*+10_mismatch probe
		vvi-miR164d|*-10_perfect match
vvi|miR-1239|nat|as|	agcacgtgcagagcagatta	probe
vvi|miR-1239|2mut|as|	agcacgtgaagagaagatta	vvi-miR164d|*-10_mismatch probe
vvi|miR-1240|nat|as|	ggagaaggggagcacgtgca	vvi-miR164d|*0_perfect match probe
vvi|miR-1240|2mut|as|	ggagaaggtgagaacgtgca	vvi-miR164d|*0_mismatch probe
		vvi-miR164d|*+10_perfect match
vvi|miR-1241|nat|as|	aacccatgttggagaagggg	probe
vvi|miR-1241|2mut|as|	aaccaatgttcgagaagggg	vvi-miR164d|*+10_mismatch probe
		vvi-miR166c|*-10_perfect match
vvi|miR-1242|nat|as|	aacattcccctcaaaatag	probe
vvi|miR-1242|2mut|as|	aacattcctctaaaaatag	vvi-miR166c|*-10_mismatch probe
vvi|miR-1243|nat|as|	cgagccagacaacattcccc	vvi-miR166c|*0_perfect match probe
vvi|miR-1243|2mut|as|	cgagtcagacaaaattcccc	vvi-miR166c|*0_mismatch probe
		vvi-miR166c|*+10_perfect match
vvi|miR-1244|nat|as|	taagtgtcctcgagccagac	probe
vvi|miR-1244|2mut|as|	taagtgttctcgagtcagac	vvi-miR166c|*+10_mismatch probe
		vvi-miR166d|*-10_perfect match
vvi|miR-1245|nat|as|	acaatcccctcaaagcag	probe
vvi|miR-1245|2mut|as|	acaatcctctaaaagcag	vvi-miR166d|*-10_mismatch probe
vvi|miR-1246|nat|as|	gagccagacaacaatcccct	vvi-miR166d|*0_perfect match probe
vvi|miR-1246|2mut|as|	gagcaagacaacaattccct	vvi-miR166d|*0_mismatch probe
		vvi-miR166d|*+10_perfect match
vvi|miR-1247|nat|as|	tggtggcctcgagccagaca	probe
vvi|miR-1247|2mut|as|	tggtggtctcgagtcagaca	vvi-miR166d|*+10_mismatch probe
		vvi-miR166e|*-10_perfect match
vvi|miR-1248|nat|as|	aacattcccctcaaaacac	probe
vvi|miR-1248|2mut|as|	aacattcctctaaaaacac	vvi-miR166e|*-10_mismatch probe
vvi|miR-1249|nat|as|	cgagccagacaacattcccc	vvi-miR166e|*0_perfect match probe
vvi|miR-1249|2mut|as|	cgagtcagacaaaattcccc	vvi-miR166e|*0_mismatch probe

<table>
<tr><td>vvi|miR-1250|nat|as|</td><td>ttggtgtcctcgagccagac</td><td>vvi-miR166e|*+10_perfect match probe</td></tr>
<tr><td>vvi|miR-1250|2mut|as|</td><td>ttggtgttctcgagtcagac</td><td>vvi-miR166e|*+10_mismatch probe</td></tr>
<tr><td>vvi|miR-1251|nat|as|</td><td>aacattccccacaactaca</td><td>vvi-miR166f|*-10_perfect match probe</td></tr>
<tr><td>vvi|miR-1251|2mut|as|</td><td>aacattcctcccaactaca</td><td>vvi-miR166f|*-10_mismatch probe</td></tr>
<tr><td>vvi|miR-1252|nat|as|</td><td>cgagccagccaacattcccc</td><td>vvi-miR166f|*0_perfect match probe</td></tr>
<tr><td>vvi|miR-1252|2mut|as|</td><td>cgagccagtcaaaattcccc</td><td>vvi-miR166f|*0_mismatch probe</td></tr>
<tr><td>vvi|miR-1253|nat|as|</td><td>aagaaagcctcgagccagcc</td><td>vvi-miR166f|*+10_perfect match probe</td></tr>
<tr><td>vvi|miR-1253|2mut|as|</td><td>aagaaagtctcgagtcagcc</td><td>vvi-miR166f|*+10_mismatch probe</td></tr>
<tr><td>vvi|miR-1254|nat|as|</td><td>acattcccctcaactgtt</td><td>vvi-miR166g|*-10_perfect match probe</td></tr>
<tr><td>vvi|miR-1254|2mut|as|</td><td>acattcctctaaactgtt</td><td>vvi-miR166g|*-10_mismatch probe</td></tr>
<tr><td>vvi|miR-1255|nat|as|</td><td>gaaccagacaacattcccct</td><td>vvi-miR166g|*0_perfect match probe</td></tr>
<tr><td>vvi|miR-1255|2mut|as|</td><td>gaacaagacaacatttccct</td><td>vvi-miR166g|*0_mismatch probe</td></tr>
<tr><td>vvi|miR-1256|nat|as|</td><td>gaaggatctcgaaccagaca</td><td>vvi-miR166g|*+10_perfect match probe</td></tr>
<tr><td>vvi|miR-1256|2mut|as|</td><td>gaaggatctaaaaccagaca</td><td>vvi-miR166g|*+10_mismatch probe</td></tr>
<tr><td>vvi|miR-1257|nat|as|</td><td>agcgttcccctcaactgtt</td><td>vvi-miR166h|*-10_perfect match probe</td></tr>
<tr><td>vvi|miR-1257|2mut|as|</td><td>agcgttcctctaaactgtt</td><td>vvi-miR166h|*-10_mismatch probe</td></tr>
<tr><td>vvi|miR-1258|nat|as|</td><td>cgaaccagacagcgttcccc</td><td>vvi-miR166h|*0_perfect match probe</td></tr>
<tr><td>vvi|miR-1258|2mut|as|</td><td>cgaacaagacagagttcccc</td><td>vvi-miR166h|*0_mismatch probe</td></tr>
<tr><td>vvi|miR-1259|nat|as|</td><td>cgagtggtctcgaaccagac</td><td>vvi-miR166h|*+10_perfect match probe</td></tr>
<tr><td>vvi|miR-1259|2mut|as|</td><td>cgagtcgtctagaaccagac</td><td>vvi-miR166h|*+10_mismatch probe</td></tr>
<tr><td>vvi|miR-1260|nat|as|</td><td>ctcattccaaacacccgc</td><td>vvi-miR166a|*-10_perfect match probe</td></tr>
<tr><td>vvi|miR-1260|2mut|as|</td><td>ctcattcaaaacatccgc</td><td>vvi-miR166a|*-10_mismatch probe</td></tr>
<tr><td>vvi|miR-1261|nat|as|</td><td>tggatcaaacctcattccaa</td><td>vvi-miR166a|*0_perfect match probe</td></tr>
<tr><td>vvi|miR-1261|2mut|as|</td><td>tggatcaaatttcattccaa</td><td>vvi-miR166a|*0_mismatch probe</td></tr>
<tr><td>vvi|miR-1262|nat|as|</td><td>ggaaggatcttggatcaaac</td><td>vvi-miR166a|*+10_perfect match probe</td></tr>
</table>

vvi|miR-1262|2mut|as|	ggaagaatcttcgatcaaac
vvi|miR-1263|nat|as|	aacattcccctcaaaaaac
vvi|miR-1263|2mut|as|	aacattcctctaaaaaaac
vvi|miR-1264|nat|as|	cgagccagccaacattcccc
vvi|miR-1264|2mut|as|	cgagccagtcaaaattcccc
vvi|miR-1265|nat|as|	gtgaatgcctcgagccagcc
vvi|miR-1265|2mut|as|	gtgaatgtctcgagtcagcc
vvi|miR-1266|nat|as|	agatgacctgatcatggaac
vvi|miR-1266|2mut|as|	agatgatctgatcatcgaac
vvi|miR-1267|nat|as|	tgaagctgcaagatgacctg
vvi|miR-1267|2mut|as|	tgaagctgaaagatgccctg
vvi|miR-1268|nat|as|	agtgagtgattgaagctgca
vvi|miR-1268|2mut|as|	agtgagcgattgaagttgca
vvi|miR-1269|nat|as|	acatgatctagtctttcctg
vvi|miR-1269|2mut|as|	acataatctagcctttcctg
vvi|miR-1270|nat|as|	tgaaactgccacatgatcta
vvi|miR-1270|2mut|as|	tgaaactgtcccatgatcta
vvi|miR-1271|nat|as|	catcaacaggtgaaactgcc
vvi|miR-1271|2mut|as|	catcaacagccgaaactgcc
vvi|miR-1272|nat|as|	cacatgatctgatctttcct
vvi|miR-1272|2mut|as|	cacatgatccaatctttcct
vvi|miR-1273|nat|as|	ccatcaacaggtgaggctac
vvi|miR-1273|2mut|as|	ccatcaacacgtgagcctac
vvi|miR-1274|nat|as|	gcatgacctagctagggtta
vvi|miR-1274|2mut|as|	gcatgatctagctagtgtta

vvi-miR166a|*+10_mismatch probe
vvi-miR166b|*-10_perfect match probe
vvi-miR166b|*-10_mismatch probe
vvi-miR166b|*0_perfect match probe
vvi-miR166b|*0_mismatch probe
vvi-miR166b|*+10_perfect match probe
vvi-miR166b|*+10_mismatch probe
vvi-miR167a|*-10_perfect match probe
vvi-miR167a|*-10_mismatch probe
vvi-miR167a|*0_perfect match probe
vvi-miR167a|*0_mismatch probe
vvi-miR167a|*+10_perfect match probe
vvi-miR167a|*+10_mismatch probe
vvi-miR167b|*-10_perfect match probe
vvi-miR167b|*-10_mismatch probe
vvi-miR167b|*0_perfect match probe
vvi-miR167b|*0_mismatch probe
vvi-miR167b|*+10_perfect match probe
vvi-miR167b|*+10_mismatch probe
vvi-miR167c|*-10_perfect match probe
vvi-miR167c|*-10_mismatch probe
vvi-miR167c|*+10_perfect match probe
vvi-miR167c|*+10_mismatch probe
vvi-miR167d|*-10_perfect match probe
vvi-miR167d|*-10_mismatch probe

vvi|miR-1275|nat|as|	ggctgtcagggcatgaccta
vvi|miR-1275|2mut|as|	ggctgtcagtccatgaccta
vvi|miR-1276|nat|as|	gaaggagtgaggctgtcagg
vvi|miR-1276|2mut|as|	gaagaagtgagcctgtcagg
vvi|miR-1277|nat|as|	cacatgatctgatctctgtt
vvi|miR-1277|2mut|as|	cacatgatccaatctctgtt
vvi|miR-1278|nat|as|	gtgaaactgccacatgatct
vvi|miR-1278|2mut|as|	gtgaaactgtaacatgatct
vvi|miR-1279|nat|as|	ccaacaacaggtgaaactgc
vvi|miR-1279|2mut|as|	ccaacaacacctgaaactgc
vvi|miR-1280|nat|as|	caaggcgggatcggaattcg
vvi|miR-1280|2mut|as|	caaggagggatcagaattcg
vvi|miR-1281|nat|as|	tcagttgatgcaaggcggga
vvi|miR-1281|2mut|as|	tcagttgatccaaggaggga
vvi|miR-1282|nat|as|	cgtctccgattcagttgatg
vvi|miR-1282|2mut|as|	cgtctcagattccgttgatg
vvi|miR-1283|nat|as|	aacttgccagggttcactat
vvi|miR-1283|2mut|as|	aacttgtcagtgttcactat
vvi|miR-1284|nat|as|	agccaaggacaacttgccag
vvi|miR-1284|2mut|as|	agccaagaacaacttcccag
vvi|miR-1285|nat|as|	aatcaaatgtagccaaggac
vvi|miR-1285|2mut|as|	aatcaaatctagtcaaggac
vvi|miR-1286|nat|as|	gagacttgccataaaccaca
vvi|miR-1286|2mut|as|	gagacttgtcataaatcaca
vvi|miR-1287|nat|as|	aggtgccaaggagacttgcc
vvi|miR-1287|2mut|as|	aggtgtcaagaagacttgcc

vvi-miR167d|*0_perfect match probe
vvi-miR167d|*0_mismatch probe
vvi-miR167d|*+10_perfect match probe
vvi-miR167d|*+10_mismatch probe
vvi-miR167e|*-10_perfect match probe
vvi-miR167e|*-10_mismatch probe
vvi-miR167e|*0_perfect match probe
vvi-miR167e|*0_mismatch probe
vvi-miR167e|*+10_perfect match probe
vvi-miR167e|*+10_mismatch probe
vvi-miR168|*-10_perfect match probe
vvi-miR168|*-10_mismatch probe
vvi-miR168|*0_perfect match probe
vvi-miR168|*0_mismatch probe
vvi-miR168|*+10_perfect match probe
vvi-miR168|*+10_mismatch probe
vvi-miR169a|*-10_perfect match probe
vvi-miR169a|*-10_mismatch probe
vvi-miR169a|*0_perfect match probe
vvi-miR169a|*0_mismatch probe
vvi-miR169a|*+10_perfect match probe
vvi-miR169a|*+10_mismatch probe
vvi-miR169y|*-10_perfect match probe
vvi-miR169y|*-10_mismatch probe
vvi-miR169y|*0_perfect match probe
vvi-miR169y|*0_mismatch probe

vvi|miR-1288|nat|as|	gaggccatcaggtgccaag	vvi-miR169y|*+10_perfect match probe
vvi|miR-1288|2mut|as|	gaggtcatcaggtgtcaag	vvi-miR169y|*+10_mismatch probe
vvi|miR-1289|nat|as|	tgactaaccgtgcacgcaca	vvi-miR169b|*-10_perfect match probe
vvi|miR-1289|2mut|as|	tgactaacagtgcacccaca	vvi-miR169b|*-10_mismatch probe
vvi|miR-1290|nat|as|	gagccaaggatgactaaccg	vvi-miR169b|*0_perfect match probe
vvi|miR-1290|2mut|as|	gagcaaaggatgcctaaccg	vvi-miR169b|*0_mismatch probe
vvi|miR-1291|nat|as|	agggccaaatgagccaagga	vvi-miR169b|*+10_perfect match probe
vvi|miR-1291|2mut|as|	agggtcaaatgagtcaagga	vvi-miR169b|*+10_mismatch probe
vvi|miR-1292|nat|as|	aacttgccggattaacgagg	vvi-miR169c|*-10_perfect match probe
vvi|miR-1292|2mut|as|	aacttgtcggattaaagagg	vvi-miR169c|*-10_mismatch probe
vvi|miR-1293|nat|as|	agccaaggacaacttgccgg	vvi-miR169c|*0_perfect match probe
vvi|miR-1293|2mut|as|	agccaagaacaacttcccgg	vvi-miR169c|*0_mismatch probe
vvi|miR-1294|nat|as|	agcccaatgtagccaaggac	vvi-miR169c|*+10_perfect match probe
vvi|miR-1294|2mut|as|	agccaaatgtagtcaaggac	vvi-miR169c|*+10_mismatch probe
vvi|miR-1295|nat|as|	aacttgccagggttcactat	vvi-miR169d|*-10_perfect match probe
vvi|miR-1295|2mut|as|	aacttgtcagtgttcactat	vvi-miR169d|*-10_mismatch probe
vvi|miR-1296|nat|as|	agccaaggacaacttgccag	vvi-miR169d|*0_perfect match probe
vvi|miR-1296|2mut|as|	agccaagaacaacttcccag	vvi-miR169d|*0_mismatch probe
vvi|miR-1297|nat|as|	aatcaaatgtagccaaggac	vvi-miR169d|*+10_perfect match probe
vvi|miR-1297|2mut|as|	aatcaaatctagtcaaggac	vvi-miR169d|*+10_mismatch probe
vvi|miR-1298|nat|as|	tgactgcctggaacccaaac	vvi-miR169e|*-10_perfect match probe
vvi|miR-1298|2mut|as|	tgactgtctggaactcaaac	vvi-miR169e|*-10_mismatch probe
vvi|miR-1299|nat|as|	ttagccaaggtgactgcctg	vvi-miR169e|*0_perfect match probe
vvi|miR-1299|2mut|as|	ttagtcaaggtgactccctg	vvi-miR169e|*0_mismatch probe
vvi|miR-1300|nat|as|	gcctgtaaaattagccaagg	vvi-miR169e|*+10_perfect match probe

vvi|miR-1300|2mut|as|	gcctctaaaattagtcaagg	vvi-miR169e|*+10_mismatch probe
		vvi-miR169f|*-10_perfect match
vvi|miR-1301|nat|as|	acaacttgcccattattgat	probe
vvi|miR-1301|2mut|as|	acaacttgtcaattattgat	vvi-miR169f|*-10_mismatch probe
vvi|miR-1302|nat|as|	gtagccaaacacaacttgcc	vvi-miR169f|*0_perfect match probe
vvi|miR-1302|2mut|as|	gtagtcaaacccaacttgcc	vvi-miR169f|*0_mismatch probe
		vvi-miR169f|*+10_perfect match
vvi|miR-1303|nat|as|	agataaacatgtagccaaac	probe
vvi|miR-1303|2mut|as|	agataaaaatgtagtcaaac	vvi-miR169f|*+10_mismatch probe
		vvi-miR169g|*-10_perfect match
vvi|miR-1304|nat|as|	acaacttgccggcttcatca	probe
vvi|miR-1304|2mut|as|	acaacttgtcgccttcatca	vvi-miR169g|*-10_mismatch probe
vvi|miR-1305|nat|as|	gtagccaaagacaacttgcc	vvi-miR169g|*0_perfect match probe
vvi|miR-1305|2mut|as|	gtagtcaaagaaaacttgcc	vvi-miR169g|*0_mismatch probe
		vvi-miR169g|*+10_perfect match
vvi|miR-1306|nat|as|	aaagaagcatgtagccaaag	probe
vvi|miR-1306|2mut|as|	aaagaagaatgtagtcaaag	vvi-miR169g|*+10_mismatch probe
		vvi-miR169h|*-10_perfect match
vvi|miR-1307|nat|as|	taaccctccgtgcgtgacca	probe
vvi|miR-1307|2mut|as|	taaccctcagtgagtgacca	vvi-miR169h|*-10_mismatch probe
vvi|miR-1308|nat|as|	gagtcaaggataaccctccg	vvi-miR169h|*0_perfect match probe
vvi|miR-1308|2mut|as|	gagtcaagaataactctccg	vvi-miR169h|*0_mismatch probe
		vvi-miR169h|*+10_perfect match
vvi|miR-1309|nat|as|	ggagctaaaggagtcaagga	probe
vvi|miR-1309|2mut|as|	ggagttaaagaagtcaagga	vvi-miR169h|*+10_mismatch probe
		vvi-miR169i|*-10_perfect match
vvi|miR-1310|nat|as|	aaccagccgtgcatgaccag	probe
vvi|miR-1310|2mut|as|	aaccagtcgtgaatgaccag	vvi-miR169i|*-10_mismatch probe
vvi|miR-1311|nat|as|	agacagggataaccagccgt	vvi-miR169i|*0_perfect match probe
vvi|miR-1311|2mut|as|	agacagtgataaccacccgt	vvi-miR169i|*0_mismatch probe
		vvi-miR169i|*+10_perfect match
vvi|miR-1312|nat|as|	gagctaaaggagacagggat	probe
vvi|miR-1312|2mut|as|	gagctaaagaagacacggat	vvi-miR169i|*+10_mismatch probe
vvi|miR-1313|nat|as|	aacctgccagggacaccatg	vvi-miR169j|*-10_perfect match

		probe
vvi|miR-1313|2mut|as|	aacctgtcagtgacaccatg	vvi-miR169j|*-10_mismatch probe
vvi|miR-1314|nat|as|	agccaaggacaacctgccag	vvi-miR169j|*0_perfect match probe
vvi|miR-1314|2mut|as|	agccaagaacaacctcccag	vvi-miR169j|*0_mismatch probe
		vvi-miR169j|*+10_perfect match
vvi|miR-1315|nat|as|	agcgaaaggtagccaaggac	probe
vvi|miR-1315|2mut|as|	agcgaaagctagtcaaggac	vvi-miR169j|*+10_mismatch probe
		vvi-miR169k|*-10_perfect match
vvi|miR-1316|nat|as|	aacctgccagggacaccatg	probe
vvi|miR-1316|2mut|as|	aacctgtcagtgacaccatg	vvi-miR169k|*-10_mismatch probe
vvi|miR-1317|nat|as|	agccaaggacaacctgccag	vvi-miR169k|*0_perfect match probe
vvi|miR-1317|2mut|as|	agccaagaacaacctcccag	vvi-miR169k|*0_mismatch probe
		vvi-miR169k|*+10_perfect match
vvi|miR-1318|nat|as|	aacgaaaggtagccaaggac	probe
vvi|miR-1318|2mut|as|	aacgaaagctagtcaaggac	vvi-miR169k|*+10_mismatch probe
		vvi-miR169l|*-10_perfect match
vvi|miR-1319|nat|as|	aactagccgtgcatgcccag	probe
vvi|miR-1319|2mut|as|	aactagtcgtgcatgtccag	vvi-miR169l|*-10_mismatch probe
vvi|miR-1320|nat|as|	agccaagaataactagccgt	vvi-miR169l|*0_perfect match probe
vvi|miR-1320|2mut|as|	agccaacaataactacccgt	vvi-miR169l|*0_mismatch probe
		vvi-miR169l|*+10_perfect match
vvi|miR-1321|nat|as|	gggccaaatgagccaagaat	probe
vvi|miR-1321|2mut|as|	gggcaaaatgagtcaagaat	vvi-miR169l|*+10_mismatch probe
		vvi-miR169m|*-10_perfect match
vvi|miR-1322|nat|as|	tgcttgccagttatggccag	probe
vvi|miR-1322|2mut|as|	tgcttgtcagttatgcccag	vvi-miR169m|*-10_mismatch probe
		vvi-miR169m|*0_perfect match
vvi|miR-1323|nat|as|	gagcctcggatgcttgccag	probe
vvi|miR-1323|2mut|as|	gagcctcagatgcttcccag	vvi-miR169m|*0_mismatch probe
		vvi-miR169m|*+10_perfect match
vvi|miR-1324|nat|as|	gggtgaaacagagcctcgga	probe
vvi|miR-1324|2mut|as|	gggtgaaaaagagtctcgga	vvi-miR169m|*+10_mismatch probe
		vvi-miR169n|*-10_perfect match
vvi|miR-1325|nat|as|	gccagttatgaccagttaaa	probe

vvi|miR-1325|2mut|as|	gccagttataacaagttaaa	vvi-miR169n|*-10_mismatch probe
vvi|miR-1326|nat|as|	tcagatgcttgccagttatg	vvi-miR169n|*0_perfect match probe
vvi|miR-1326|2mut|as|	tcagatccttgtcagttatg	vvi-miR169n|*0_mismatch probe
vvi|miR-1327|nat|as|	aaatagagcctcagatgctt	vvi-miR169n|*+10_perfect match probe
vvi|miR-1327|2mut|as|	aaatagagtctccgatgctt	vvi-miR169n|*+10_mismatch probe
vvi|miR-1328|nat|as|	actcgctgtccatgcccagt	vvi-miR169o|*-10_perfect match probe
vvi|miR-1328|2mut|as|	actccctgtccatgtccagt	vvi-miR169o|*-10_mismatch probe
vvi|miR-1329|nat|as|	gccaagaataactcgctgtc	vvi-miR169o|*0_perfect match probe
vvi|miR-1329|2mut|as|	gccaacaataactccctgtc	vvi-miR169o|*0_mismatch probe
vvi|miR-1330|nat|as|	ggccgcatgagccaagaata	vvi-miR169o|*+10_perfect match probe
vvi|miR-1330|2mut|as|	ggccccatgagtcaagaata	vvi-miR169o|*+10_mismatch probe
vvi|miR-1331|nat|as|	tgcttgccagttatagccag	vvi-miR169p|*-10_perfect match probe
vvi|miR-1331|2mut|as|	tgcttgtcagttatacccag	vvi-miR169p|*-10_mismatch probe
vvi|miR-1332|nat|as|	gagcctcggatgcttgccag	vvi-miR169p|*0_perfect match probe
vvi|miR-1332|2mut|as|	gagcctcagatgcttcccag	vvi-miR169p|*0_mismatch probe
vvi|miR-1333|nat|as|	gggtgaaacagagcctcgga	vvi-miR169p|*+10_perfect match probe
vvi|miR-1333|2mut|as|	gggtgaaaaagagtctcgga	vvi-miR169p|*+10_mismatch probe
vvi|miR-1334|nat|as|	tacatgccggctgttccagt	vvi-miR169q|*-10_perfect match probe
vvi|miR-1334|2mut|as|	tacatgccgcttgttccagt	vvi-miR169q|*-10_mismatch probe
vvi|miR-1335|nat|as|	gagccaggattacatgccgg	vvi-miR169q|*0_perfect match probe
vvi|miR-1335|2mut|as|	gagcaaggattacatcccgg	vvi-miR169q|*0_mismatch probe
vvi|miR-1336|nat|as|	ggaccaaatagagccaggat	vvi-miR169q|*+10_perfect match probe
vvi|miR-1336|2mut|as|	ggacaaaatagagtcaggat	vvi-miR169q|*+10_mismatch probe
vvi|miR-1337|nat|as|	caacttgccggttacacata	vvi-miR169r|*-10_perfect match probe
vvi|miR-1337|2mut|as|	caacttgtcgcttacacata	vvi-miR169r|*-10_mismatch probe
vvi|miR-1338|nat|as|	tgagtcaagtcaacttgccg	vvi-miR169r|*0_perfect match probe

vvi|miR-1338|2mut|as|	tgagtcaagcaaacttgccg
vvi|miR-1339|nat|as|	agggccaaactgagtcaagt
vvi|miR-1339|2mut|as|	agggtcaaactgagccaagt
vvi|miR-1340|nat|as|	aacttgccgggaagatgatc
vvi|miR-1340|2mut|as|	aacttgtcggaaagatgatc
vvi|miR-1341|nat|as|	agccaggaacaacttgccgg
vvi|miR-1341|2mut|as|	agccagaaacaacttcccgg
vvi|miR-1342|nat|as|	agcagaatgtagccaggaac
vvi|miR-1342|2mut|as|	agcagaatctagtcaggaac
vvi|miR-1343|nat|as|	caacttgccggttacacata
vvi|miR-1343|2mut|as|	caacttgtcgcttacacata
vvi|miR-1344|nat|as|	tgagtcaagtcaacttgccg
vvi|miR-1344|2mut|as|	tgagtcaagcaaacttgccg
vvi|miR-1345|nat|as|	agggccaaactgagtcaagt
vvi|miR-1345|2mut|as|	agggtcaaactgagccaagt
vvi|miR-1346|nat|as|	caacttgccggtaacacata
vvi|miR-1346|2mut|as|	caacttgtcgctaacacata
vvi|miR-1347|nat|as|	agagtcaagtcaacttgccg
vvi|miR-1347|2mut|as|	agagtcaagcaaacttgccg
vvi|miR-1348|nat|as|	agggccaaacagagtcaagt
vvi|miR-1348|2mut|as|	agggtcaaacagagccaagt
vvi|miR-1349|nat|as|	aaattgccgatccatggaca
vvi|miR-1349|2mut|as|	aaattgtcgatccatcgaca
vvi|miR-1350|nat|as|	gccaagaaataaattgccga
vvi|miR-1350|2mut|as|	gccaacaaataaattcccga
vvi|miR-1351|nat|as|	gcccaacatagccaagaaat

vvi-miR169r|*0_mismatch probe
vvi-miR169r|*+10_perfect match probe
vvi-miR169r|*+10_mismatch probe
vvi-miR169s|*-10_perfect match probe
vvi-miR169s|*-10_mismatch probe
vvi-miR169s|*0_perfect match probe
vvi-miR169s|*0_mismatch probe
vvi-miR169s|*+10_perfect match probe
vvi-miR169s|*+10_mismatch probe
vvi-miR169t|*-10_perfect match probe
vvi-miR169t|*-10_mismatch probe
vvi-miR169t|*0_perfect match probe
vvi-miR169t|*0_mismatch probe
vvi-miR169t|*+10_perfect match probe
vvi-miR169t|*+10_mismatch probe
vvi-miR169u|*-10_perfect match probe
vvi-miR169u|*-10_mismatch probe
vvi-miR169u|*0_perfect match probe
vvi-miR169u|*0_mismatch probe
vvi-miR169u|*+10_perfect match probe
vvi-miR169u|*+10_mismatch probe
vvi-miR169v|*-10_perfect match probe
vvi-miR169v|*-10_mismatch probe
vvi-miR169v|*0_perfect match probe
vvi-miR169v|*0_mismatch probe
vvi-miR169v|*+10_perfect match

		probe
vvi|miR-1351|2mut|as|	gcccaaaatagtcaagaaat	vvi-miR169v|*+10_mismatch probe
		vvi-miR169w|*-10_perfect match
vvi|miR-1352|nat|as|	gactcgccgaccaccatttt	probe
vvi|miR-1352|2mut|as|	gactccccgaccacaatttt	vvi-miR169w|*-10_mismatch probe
vvi|miR-1353|nat|as|	agctaagaatgactcgccga	vvi-miR169w|*0_perfect match probe
vvi|miR-1353|2mut|as|	agctaacaatgactccccga	vvi-miR169w|*0_mismatch probe
		vvi-miR169w|*+10_perfect match
vvi|miR-1354|nat|as|	gcagaaatgtagctaagaat	probe
vvi|miR-1354|2mut|as|	gcagaaatctagttaagaat	vvi-miR169w|*+10_mismatch probe
		vvi-miR169x|*-10_perfect match
vvi|miR-1355|nat|as|	tgactgcctaaagaccacgg	probe
vvi|miR-1355|2mut|as|	tgactgtctaaagacaacgg	vvi-miR169x|*-10_mismatch probe
vvi|miR-1356|nat|as|	gccgaaggaatgactgccta	vvi-miR169x|*0_perfect match probe
vvi|miR-1356|2mut|as|	gccgaagaaatgactcccta	vvi-miR169x|*0_mismatch probe
		vvi-miR169x|*+10_perfect match
vvi|miR-1357|nat|as|	ggtcagtttagccgaaggaa	probe
vvi|miR-1357|2mut|as|	ggtccgtttagtcgaaggaa	vvi-miR169x|*+10_mismatch probe
		vvi-miR171a|*-10_perfect match
vvi|miR-1358|nat|as|	caatatcatgttcaatac	probe
vvi|miR-1358|2mut|as|	caatataatcttcaatac	vvi-miR171a|*-10_mismatch probe
vvi|miR-1359|nat|as|	tgagccgtgccaatatcatg	vvi-miR171a|*0_perfect match probe
vvi|miR-1359|2mut|as|	tgagccgtgtaaatatcatg	vvi-miR171a|*0_mismatch probe
		vvi-miR171a|*+10_perfect match
vvi|miR-1360|nat|as|	ttaatctgattgagccgtgc	probe
vvi|miR-1360|2mut|as|	ttaatccgattgagtcgtgc	vvi-miR171a|*+10_mismatch probe
		vvi-miR171b|*-10_perfect match
vvi|miR-1361|nat|as|	cgccaatacctcccccca	probe
vvi|miR-1361|2mut|as|	cgccaatatctcctccca	vvi-miR171b|*-10_mismatch probe
vvi|miR-1362|nat|as|	aattgaggcacgccaatacc	vvi-miR171b|*0_perfect match probe
vvi|miR-1362|2mut|as|	aattgagccaccccaatacc	vvi-miR171b|*0_mismatch probe
		vvi-miR171b|*+10_perfect match
vvi|miR-1363|nat|as|	catgtctttaaattgaggca	probe
vvi|miR-1363|2mut|as|	catgcctttaaattaaggca	vvi-miR171b|*+10_mismatch probe

vvi|miR-1364|nat|as|	caatatcccgtaatacca	vvi-miR171c|*-10_perfect match probe
vvi|miR-1364|2mut|as|	caatatccactaatacca	vvi-miR171c|*-10_mismatch probe
vvi|miR-1365|nat|as|	tgaaccgcaccaatatcccg	vvi-miR171c|*0_perfect match probe
vvi|miR-1365|2mut|as|	tgaaccccacaaatatcccg	vvi-miR171c|*0_mismatch probe
vvi|miR-1366|nat|as|	gctttcttattgaaccgcac	vvi-miR171c|*+10_perfect match probe
vvi|miR-1366|2mut|as|	gcttttttattgaacagcac	vvi-miR171c|*+10_mismatch probe
vvi|miR-1367|nat|as|	caatatctcgtgtatcta	vvi-miR171d|*-10_perfect match probe
vvi|miR-1367|2mut|as|	caatatctactgtatcta	vvi-miR171d|*-10_mismatch probe
vvi|miR-1368|nat|as|	tgaaccgtatcaatatctcg	vvi-miR171d|*0_perfect match probe
vvi|miR-1368|2mut|as|	tgaacagtataaatatctcg	vvi-miR171d|*0_mismatch probe
vvi|miR-1369|nat|as|	gctttctaattgaaccgtat	vvi-miR171d|*+10_perfect match probe
vvi|miR-1369|2mut|as|	gctttttaattgaacagtat	vvi-miR171d|*+10_mismatch probe
vvi|miR-1370|nat|as|	caccaacatcgcttactttc	vvi-miR171e|*-10_perfect match probe
vvi|miR-1370|2mut|as|	caccaacataccttactttc	vvi-miR171e|*-10_mismatch probe
vvi|miR-1371|nat|as|	gattgaacctcaccaacatc	vvi-miR171e|*0_perfect match probe
vvi|miR-1371|2mut|as|	gattgaatctcacaaacatc	vvi-miR171e|*0_mismatch probe
vvi|miR-1372|nat|as|	tccgtcttcggattgaacct	vvi-miR171e|*+10_perfect match probe
vvi|miR-1372|2mut|as|	tccgtcttcaaattgaacct	vvi-miR171e|*+10_mismatch probe
vvi|miR-1373|nat|as|	caacatcgctttcttcaa	vvi-miR171f|*-10_perfect match probe
vvi|miR-1373|2mut|as|	caacatcccttttttcaa	vvi-miR171f|*-10_mismatch probe
vvi|miR-1374|nat|as|	tgaacctcaccaacatcgct	vvi-miR171f|*0_perfect match probe
vvi|miR-1374|2mut|as|	tgaacctcataaacatcgct	vvi-miR171f|*0_mismatch probe
vvi|miR-1375|nat|as|	atcttcagattgaacctcac	vvi-miR171f|*+10_perfect match probe
vvi|miR-1375|2mut|as|	atcttccgattgaatctcac	vvi-miR171f|*+10_mismatch probe
vvi|miR-1376|nat|as|	gaaccaacatggagaggctg	vvi-miR171g|*-10_perfect match probe

vvi|miR-1376|2mut|as|	gaacaaacatcgagaggctg	vvi-miR171g|*-10_mismatch probe
vvi|miR-1377|nat|as|	cccaccgatggaaccaacat	vvi-miR171g|*0_perfect match probe
vvi|miR-1377|2mut|as|	cccacagatggaacaaacat	vvi-miR171g|*0_mismatch probe
vvi|miR-1378|nat|as|	gttggtgtcccccaccgatg	vvi-miR171g|*+10_perfect match probe
vvi|miR-1378|2mut|as|	gttggtgtctcccacagatg	vvi-miR171g|*+10_mismatch probe
vvi|miR-1379|nat|as|	caccaacatctcgtgaaa	vvi-miR171h|*-10_perfect match probe
vvi|miR-1379|2mut|as|	caccaaaatctcctgaaa	vvi-miR171h|*-10_mismatch probe
vvi|miR-1380|nat|as|	ggttgaaccgcaccaacatc	vvi-miR171h|*0_perfect match probe
vvi|miR-1380|2mut|as|	ggttgaacccaaccaacatc	vvi-miR171h|*0_mismatch probe
vvi|miR-1381|nat|as|	actacagttaggttgaaccg	vvi-miR171h|*+10_perfect match probe
vvi|miR-1381|2mut|as|	actaaagttagcttgaaccg	vvi-miR171h|*+10_mismatch probe
vvi|miR-1382|nat|as|	caacatccccaccttcttc	vvi-miR171i|*-10_perfect match probe
vvi|miR-1382|2mut|as|	caacatcctcatcttcttc	vvi-miR171i|*-10_mismatch probe
vvi|miR-1383|nat|as|	tgagccattccaacatcccc	vvi-miR171i|*0_perfect match probe
vvi|miR-1383|2mut|as|	tgagtcattcaaacatcccc	vvi-miR171i|*0_mismatch probe
vvi|miR-1384|nat|as|	ttgatttgattgagccattc	vvi-miR171i|*+10_perfect match probe
vvi|miR-1384|2mut|as|	ttgatttaattgagtcattc	vvi-miR171i|*+10_mismatch probe
vvi|miR-1385|nat|as|	gatgctgcatcggcaata	vvi-miR172a|*-10_perfect match probe
vvi|miR-1385|2mut|as|	gatgctgaatcgccaata	vvi-miR172a|*-10_mismatch probe
vvi|miR-1386|nat|as|	gaatcttgatgatgctgcat	vvi-miR172a|*0_perfect match probe
vvi|miR-1386|2mut|as|	gaatcttaatgatcctgcat	vvi-miR172a|*0_mismatch probe
vvi|miR-1387|nat|as|	tcaaggatgagaatcttgat	vvi-miR172a|*+10_perfect match probe
vvi|miR-1387|2mut|as|	tcaagaatgagaattttgat	vvi-miR172a|*+10_mismatch probe
vvi|miR-1388|nat|as|	tgatgctgcatcggcaata	vvi-miR172b|*-10_perfect match probe
vvi|miR-1388|2mut|as|	tgatgctgaatcgccaata	vvi-miR172b|*-10_mismatch probe
vvi|miR-1389|nat|as|	agaatcttgatgatgctgca	vvi-miR172b|*0_perfect match probe

vvi|miR-1389|2mut|as|	agaatcttaatgatcctgca	vvi-miR172b|*0_mismatch probe
		vvi-miR172b|*+10_perfect match
vvi|miR-1390|nat|as|	tttggggttgagaatcttga	probe
vvi|miR-1390|2mut|as|	tttggtgttgcgaatcttga	vvi-miR172b|*+10_mismatch probe
		vvi-miR172c|*-10_perfect match
vvi|miR-1391|nat|as|	tgatgctccatccgcaaac	probe
vvi|miR-1391|2mut|as|	tgatcctccatccccaaac	vvi-miR172c|*-10_mismatch probe
vvi|miR-1392|nat|as|	tgaatcttgatgatgctcca	vvi-miR172c|*0_perfect match probe
vvi|miR-1392|2mut|as|	tgaatcttaatgatcctcca	vvi-miR172c|*0_mismatch probe
		vvi-miR172c|*+10_perfect match
vvi|miR-1393|nat|as|	tcaatacttgtgaatcttga	probe
vvi|miR-1393|2mut|as|	tcaatacttccgaatcttga	vvi-miR172c|*+10_mismatch probe
		vvi-miR172d|*-10_perfect match
vvi|miR-1394|nat|as|	gatgctgcatcagctaac	probe
vvi|miR-1394|2mut|as|	gatgctgaatcagttaac	vvi-miR172d|*-10_mismatch probe
vvi|miR-1395|nat|as|	gaatcttgatgatgctgcat	vvi-miR172d|*0_perfect match probe
vvi|miR-1395|2mut|as|	gaatcttaatgatcctgcat	vvi-miR172d|*0_mismatch probe
		vvi-miR172d|*+10_perfect match
vvi|miR-1396|nat|as|	cattgggtgtgaatcttgat	probe
vvi|miR-1396|2mut|as|	cattgtgtgtaaatcttgat	vvi-miR172d|*+10_mismatch probe
		vvi-miR319b|*-10_perfect match
vvi|miR-1397|nat|as|	agagagctccttcttccaca	probe
vvi|miR-1397|2mut|as|	agagagttccttctttcaca	vvi-miR319b|*-10_mismatch probe
vvi|miR-1398|nat|as|	actggactaaagagagctcc	vvi-miR319b|*0_perfect match probe
vvi|miR-1398|2mut|as|	actgaactaaagagacctcc	vvi-miR319b|*0_mismatch probe
		vvi-miR319b|*+10_perfect match
vvi|miR-1399|nat|as|	gctgtctcggactggactaa	probe
vvi|miR-1399|2mut|as|	gctgtctcagactcgactaa	vvi-miR319b|*+10_mismatch probe
		vvi-miR319c|*-10_perfect match
vvi|miR-1400|nat|as|	gaaagctctcttcaatgtaa	probe
vvi|miR-1400|2mut|as|	gaaagttctcttaaatgtaa	vvi-miR319c|*-10_mismatch probe
vvi|miR-1401|nat|as|	gtggactgaagaaagctctc	vvi-miR319c|*0_perfect match probe
vvi|miR-1401|2mut|as|	gtggcctgaagaaagttctc	vvi-miR319c|*0_mismatch probe
vvi|miR-1402|nat|as|	ccacccatgagtggactgaa	vvi-miR319c|*+10_perfect match

<table>
<tr><td></td><td></td><td>probe</td></tr>
<tr><td>vvi|miR-1402|2mut|as|</td><td>ccactcatgagtcgactgaa</td><td>vvi-miR319c|*+10_mismatch probe</td></tr>
<tr><td>vvi|miR-1403|nat|as|</td><td>aggaacccccatttctgca</td><td>vvi-miR319e|*-10_perfect match probe</td></tr>
<tr><td>vvi|miR-1403|2mut|as|</td><td>aggaaccctcatttttgca</td><td>vvi-miR319e|*-10_mismatch probe</td></tr>
<tr><td>vvi|miR-1404|nat|as|</td><td>tgggctgcaaaggaaccccc</td><td>vvi-miR319e|*0_perfect match probe</td></tr>
<tr><td>vvi|miR-1404|2mut|as|</td><td>tgggctgaaaaggaatcccc</td><td>vvi-miR319e|*0_mismatch probe</td></tr>
<tr><td>vvi|miR-1405|nat|as|</td><td>ggagttgttttgggctgcaa</td><td>vvi-miR319e|*+10_perfect match probe</td></tr>
<tr><td>vvi|miR-1405|2mut|as|</td><td>ggagttcttttggcctgcaa</td><td>vvi-miR319e|*+10_mismatch probe</td></tr>
<tr><td>vvi|miR-1406|nat|as|</td><td>ggaagctctcttaaaccact</td><td>vvi-miR319f|*-10_perfect match probe</td></tr>
<tr><td>vvi|miR-1406|2mut|as|</td><td>ggaagttctcttaaatcact</td><td>vvi-miR319f|*-10_mismatch probe</td></tr>
<tr><td>vvi|miR-1407|nat|as|</td><td>gtggactgaaggaagctctc</td><td>vvi-miR319f|*0_perfect match probe</td></tr>
<tr><td>vvi|miR-1407|2mut|as|</td><td>gtggcctgaaggaagttctc</td><td>vvi-miR319f|*0_mismatch probe</td></tr>
<tr><td>vvi|miR-1408|nat|as|</td><td>ccatccatgagtggactgaa</td><td>vvi-miR319f|*+10_perfect match probe</td></tr>
<tr><td>vvi|miR-1408|2mut|as|</td><td>ccatcaatgagtcgactgaa</td><td>vvi-miR319f|*+10_mismatch probe</td></tr>
<tr><td>vvi|miR-1409|nat|as|</td><td>aaaggagctcttcagctgtt</td><td>vvi-miR319g|*-10_perfect match probe</td></tr>
<tr><td>vvi|miR-1409|2mut|as|</td><td>aaaggagttcttcagttgtt</td><td>vvi-miR319g|*-10_mismatch probe</td></tr>
<tr><td>vvi|miR-1410|nat|as|</td><td>tattggactaaaaggagctc</td><td>vvi-miR319g|*0_perfect match probe</td></tr>
<tr><td>vvi|miR-1410|2mut|as|</td><td>tattcgactaaaagaagctc</td><td>vvi-miR319g|*0_mismatch probe</td></tr>
<tr><td>vvi|miR-1411|nat|as|</td><td>cagccctctttattggacta</td><td>vvi-miR319g|*+10_perfect match probe</td></tr>
<tr><td>vvi|miR-1411|2mut|as|</td><td>cagctctctttattcgacta</td><td>vvi-miR319g|*+10_mismatch probe</td></tr>
<tr><td>vvi|miR-1412|nat|as|</td><td>agatagcgccacacacacaa</td><td>vvi-miR390|*-10_perfect match probe</td></tr>
<tr><td>vvi|miR-1412|2mut|as|</td><td>agatagccccacacccacaa</td><td>vvi-miR390|*-10_mismatch probe</td></tr>
<tr><td>vvi|miR-1413|nat|as|</td><td>aactcaggatagatagcgcc</td><td>vvi-miR390|*0_perfect match probe</td></tr>
<tr><td>vvi|miR-1413|2mut|as|</td><td>aactcagaatagataccgcc</td><td>vvi-miR390|*0_mismatch probe</td></tr>
<tr><td>vvi|miR-1414|nat|as|</td><td>gaacccgtgaaactcaggat</td><td>vvi-miR390|*+10_perfect match probe</td></tr>
<tr><td>vvi|miR-1414|2mut|as|</td><td>gaaccagtgaaactccggat</td><td>vvi-miR390|*+10_mismatch probe</td></tr>
</table>

vvi|miR-1415|nat|as|	agcatgatccaatgggaaaa	vvi-miR393a|*-10_perfect match probe
vvi|miR-1415|2mut|as|	agcatgatcaaatgtgaaaa	vvi-miR393a|*-10_mismatch probe
vvi|miR-1416|nat|as|	cctaagggatagcatgatcc	vvi-miR393a|*0_perfect match probe
vvi|miR-1416|2mut|as|	cctaagtgatagaatgatcc	vvi-miR393a|*0_mismatch probe
vvi|miR-1417|nat|as|	atggagagttcctaagggat	vvi-miR393a|*+10_perfect match probe
vvi|miR-1417|2mut|as|	atggagactttctaagggat	vvi-miR393a|*+10_mismatch probe
vvi|miR-1418|nat|as|	agcatgatccaaagacatgg	vvi-miR393b|*-10_perfect match probe
vvi|miR-1418|2mut|as|	agcatgatcaaaagaaatgg	vvi-miR393b|*-10_mismatch probe
vvi|miR-1419|nat|as|	ccaaagggatagcatgatcc	vvi-miR393b|*0_perfect match probe
vvi|miR-1419|2mut|as|	ccaaagtgatagaatgatcc	vvi-miR393b|*0_mismatch probe
vvi|miR-1420|nat|as|	aaggaggaatccaaagggat	vvi-miR393b|*+10_perfect match probe
vvi|miR-1420|2mut|as|	aaggagaaatcaaaagggat	vvi-miR393b|*+10_mismatch probe
vvi|miR-1421|nat|as|	ggccacctccaaaagaatat	vvi-miR394a|*-10_perfect match probe
vvi|miR-1421|2mut|as|	ggccacctcaaaaacaatat	vvi-miR394a|*-10_mismatch probe
vvi|miR-1422|nat|as|	cagagccactttggcatgct	vvi-miR394a|*+10_perfect match probe
vvi|miR-1422|2mut|as|	cagagtcactttgccatgct	vvi-miR394a|*+10_mismatch probe
vvi|miR-1423|nat|as|	gcccacctccacagagagaa	vvi-miR394b|*-10_perfect match probe
vvi|miR-1423|2mut|as|	gcccacctcaccagagagaa	vvi-miR394b|*-10_mismatch probe
vvi|miR-1424|nat|as|	cagagcttggttggcagtat	vvi-miR394b|*+10_perfect match probe
vvi|miR-1424|2mut|as|	cagagtttggttgccagtat	vvi-miR394b|*+10_mismatch probe
vvi|miR-1425|nat|as|	ggccgcctccaaacgcttgt	vvi-miR394c|*-10_perfect match probe
vvi|miR-1425|2mut|as|	ggccccctccaaacccttgt	vvi-miR394c|*-10_mismatch probe
vvi|miR-1426|nat|as|	cagagccaatttggcatcct	vvi-miR394c|*+10_perfect match probe
vvi|miR-1426|2mut|as|	cagagtcaatttgccatcct	vvi-miR394c|*+10_mismatch probe

vvi|miR-1427|nat|as|	aagggaactctaggggac	vvi-miR395a|*-10_perfect match probe
vvi|miR-1427|2mut|as|	aaggaaactctagtggac	vvi-miR395a|*-10_mismatch probe
vvi|miR-1428|nat|as|	tgaagtgatcaagggaactc	vvi-miR395a|*0_perfect match probe
vvi|miR-1428|2mut|as|	tgaagcgatcaagtgaactc	vvi-miR395a|*0_mismatch probe
vvi|miR-1429|nat|as|	agctccctagtgaagtgatc	vvi-miR395a|*+10_perfect match probe
vvi|miR-1429|2mut|as|	agctctctagtgaagcgatc	vvi-miR395a|*+10_mismatch probe
vvi|miR-1430|nat|as|	aggggaactctaggggac	vvi-miR395b|*-10_perfect match probe
vvi|miR-1430|2mut|as|	agggaaactctagtggac	vvi-miR395b|*-10_mismatch probe
vvi|miR-1431|nat|as|	tgaagtggtaaggggaactc	vvi-miR395b|*0_perfect match probe
vvi|miR-1431|2mut|as|	tgaagtcgtaaggtgaactc	vvi-miR395b|*0_mismatch probe
vvi|miR-1432|nat|as|	agatccccagtgaagtggta	vvi-miR395b|*+10_perfect match probe
vvi|miR-1432|2mut|as|	agatcctcagtgaagcggta	vvi-miR395b|*+10_mismatch probe
vvi|miR-1433|nat|as|	aagggaactctaggggac	vvi-miR395c|*-10_perfect match probe
vvi|miR-1433|2mut|as|	aaggaaactctagtggac	vvi-miR395c|*-10_mismatch probe
vvi|miR-1434|nat|as|	tgaagtggtcaagggaactc	vvi-miR395c|*0_perfect match probe
vvi|miR-1434|2mut|as|	tgaagtcgtcaagtgaactc	vvi-miR395c|*0_mismatch probe
vvi|miR-1435|nat|as|	aggtccccagtgaagtggtc	vvi-miR395c|*+10_perfect match probe
vvi|miR-1435|2mut|as|	aggtcctcagtgaagcggtc	vvi-miR395c|*+10_mismatch probe
vvi|miR-1436|nat|as|	aggggaactctagggggc	vvi-miR395d|*-10_perfect match probe
vvi|miR-1436|2mut|as|	agggaaactctagtgggc	vvi-miR395d|*-10_mismatch probe
vvi|miR-1437|nat|as|	tgaagtggtcaggggaactc	vvi-miR395d|*0_perfect match probe
vvi|miR-1437|2mut|as|	tgaagtcgtcaggtgaactc	vvi-miR395d|*0_mismatch probe
vvi|miR-1438|nat|as|	agatccccaatgaagtggtc	vvi-miR395d|*+10_perfect match probe
vvi|miR-1438|2mut|as|	agatcctcaatgaagcggtc	vvi-miR395d|*+10_mismatch probe
vvi|miR-1439|nat|as|	aagggaactctaggggag	vvi-miR395e|*-10_perfect match probe

<table>
<tr><td>vvi|miR-1439|2mut|as|</td><td>aaggaaactctagtggag</td><td>vvi-miR395e|*-10_mismatch probe</td></tr>
<tr><td>vvi|miR-1440|nat|as|</td><td>tgaagtggtcaagggaactc</td><td>vvi-miR395e|*0_perfect match probe</td></tr>
<tr><td>vvi|miR-1440|2mut|as|</td><td>tgaagtcgtcaagtgaactc</td><td>vvi-miR395e|*0_mismatch probe</td></tr>
<tr><td>vvi|miR-1441|nat|as|</td><td>aggtccccagtgaagtggtc</td><td>vvi-miR395e|*+10_perfect match probe</td></tr>
<tr><td>vvi|miR-1441|2mut|as|</td><td>aggtcctcagtgaagcggtc</td><td>vvi-miR395e|*+10_mismatch probe</td></tr>
<tr><td>vvi|miR-1442|nat|as|</td><td>aggggaactctagggtac</td><td>vvi-miR395f|*-10_perfect match probe</td></tr>
<tr><td>vvi|miR-1442|2mut|as|</td><td>agggaaactctagtgtac</td><td>vvi-miR395f|*-10_mismatch probe</td></tr>
<tr><td>vvi|miR-1443|nat|as|</td><td>tgaagtggtcaggggaactc</td><td>vvi-miR395f|*0_perfect match probe</td></tr>
<tr><td>vvi|miR-1443|2mut|as|</td><td>tgaagtcgtcaggtgaactc</td><td>vvi-miR395f|*0_mismatch probe</td></tr>
<tr><td>vvi|miR-1444|nat|as|</td><td>agatccccagtgaagtggtc</td><td>vvi-miR395f|*+10_perfect match probe</td></tr>
<tr><td>vvi|miR-1444|2mut|as|</td><td>agatcctcagtgaagcggtc</td><td>vvi-miR395f|*+10_mismatch probe</td></tr>
<tr><td>vvi|miR-1445|nat|as|</td><td>aggggaactctaggggac</td><td>vvi-miR395g|*-10_perfect match probe</td></tr>
<tr><td>vvi|miR-1445|2mut|as|</td><td>agggaaactctagtggac</td><td>vvi-miR395g|*-10_mismatch probe</td></tr>
<tr><td>vvi|miR-1446|nat|as|</td><td>tgaagtgctcaggggaactc</td><td>vvi-miR395g|*0_perfect match probe</td></tr>
<tr><td>vvi|miR-1446|2mut|as|</td><td>tgaagtcctcaggtgaactc</td><td>vvi-miR395g|*0_mismatch probe</td></tr>
<tr><td>vvi|miR-1447|nat|as|</td><td>ggatccccaatgaagtgctc</td><td>vvi-miR395g|*+10_perfect match probe</td></tr>
<tr><td>vvi|miR-1447|2mut|as|</td><td>ggatcctcaatgaagcgctc</td><td>vvi-miR395g|*+10_mismatch probe</td></tr>
<tr><td>vvi|miR-1448|nat|as|</td><td>aagggaactctaggggac</td><td>vvi-miR395h|*-10_perfect match probe</td></tr>
<tr><td>vvi|miR-1448|2mut|as|</td><td>aaggaaactctagtggac</td><td>vvi-miR395h|*-10_mismatch probe</td></tr>
<tr><td>vvi|miR-1449|nat|as|</td><td>tgaagtggtcaagggaactc</td><td>vvi-miR395h|*0_perfect match probe</td></tr>
<tr><td>vvi|miR-1449|2mut|as|</td><td>tgaagtcgtcaagtgaactc</td><td>vvi-miR395h|*0_mismatch probe</td></tr>
<tr><td>vvi|miR-1450|nat|as|</td><td>aggtccccagtgaagtggtc</td><td>vvi-miR395h|*+10_perfect match probe</td></tr>
<tr><td>vvi|miR-1450|2mut|as|</td><td>aggtcctcagtgaagcggtc</td><td>vvi-miR395h|*+10_mismatch probe</td></tr>
<tr><td>vvi|miR-1451|nat|as|</td><td>aggggaactctagggggc</td><td>vvi-miR395i|*-10_perfect match probe</td></tr>
<tr><td>vvi|miR-1451|2mut|as|</td><td>agggaaactctagtgggc</td><td>vvi-miR395i|*-10_mismatch probe</td></tr>
<tr><td>vvi|miR-1452|nat|as|</td><td>tgaagtggtcaggggaactc</td><td>vvi-miR395i|*0_perfect match probe</td></tr>
</table>

vvi|miR-1452|2mut|as|	tgaagtcgtcaggtgaactc	vvi-miR395i|*0_mismatch probe
		vvi-miR395i|*+10_perfect match probe
vvi|miR-1453|nat|as|	agatccccagtgaagtggtc	
vvi|miR-1453|2mut|as|	agatcctcagtgaagcggtc	vvi-miR395i|*+10_mismatch probe
		vvi-miR395j|*-10_perfect match probe
vvi|miR-1454|nat|as|	aggggaactctagggggc	
vvi|miR-1454|2mut|as|	agggaaactctagtgggc	vvi-miR395j|*-10_mismatch probe
vvi|miR-1455|nat|as|	tgaagtggtcaggggaactc	vvi-miR395j|*0_perfect match probe
vvi|miR-1455|2mut|as|	tgaagtcgtcaggtgaactc	vvi-miR395j|*0_mismatch probe
		vvi-miR395j|*+10_perfect match probe
vvi|miR-1456|nat|as|	agatccccagtgaagtggtc	
vvi|miR-1456|2mut|as|	agatcctcagtgaagcggtc	vvi-miR395j|*+10_mismatch probe
		vvi-miR395k|*-10_perfect match probe
vvi|miR-1457|nat|as|	aagggaactctaggggga	
vvi|miR-1457|2mut|as|	aaggaaactctacggga	vvi-miR395k|*-10_mismatch probe
vvi|miR-1458|nat|as|	tgaagtggtcaagggaactc	vvi-miR395k|*0_perfect match probe
vvi|miR-1458|2mut|as|	tgaagtcgtcaagtgaactc	vvi-miR395k|*0_mismatch probe
		vvi-miR395k|*+10_perfect match probe
vvi|miR-1459|nat|as|	aggtccccagtgaagtggtc	
vvi|miR-1459|2mut|as|	aggtcctcagtgaagcggtc	vvi-miR395k|*+10_mismatch probe
		vvi-miR395l|*-10_perfect match probe
vvi|miR-1460|nat|as|	aggggaactctagggggc	
vvi|miR-1460|2mut|as|	agggaaactctagtgggc	vvi-miR395l|*-10_mismatch probe
vvi|miR-1461|nat|as|	tgaagtggtcaggggaactc	vvi-miR395l|*0_perfect match probe
vvi|miR-1461|2mut|as|	tgaagtcgtcaggtgaactc	vvi-miR395l|*0_mismatch probe
		vvi-miR395l|*+10_perfect match probe
vvi|miR-1462|nat|as|	gatcccccagtgaagtggtc	
vvi|miR-1462|2mut|as|	gatccctcagtgaagcggtc	vvi-miR395l|*+10_mismatch probe
		vvi-miR395m|*-10_perfect match probe
vvi|miR-1463|nat|as|	aagggaactctaggggac	
vvi|miR-1463|2mut|as|	aaggaaactctagtggac	vvi-miR395m|*-10_mismatch probe
		vvi-miR395m|*0_perfect match probe
vvi|miR-1464|nat|as|	tgaagtgttcaagggaactc	
vvi|miR-1464|2mut|as|	tgaagcgttcaagtgaactc	vvi-miR395m|*0_mismatch probe

<table>
<tr><td>vvi|miR-1465|nat|as|</td><td>aggtccccagtgaagtgttc</td><td>vvi-miR395m|*+10_perfect match probe</td></tr>
<tr><td>vvi|miR-1465|2mut|as|</td><td>aggtcctcagtgaagcgttc</td><td>vvi-miR395m|*+10_mismatch probe</td></tr>
<tr><td>vvi|miR-1466|nat|as|</td><td>gggggaactctcaaggcc</td><td>vvi-miR395n|*-10_perfect match probe</td></tr>
<tr><td>vvi|miR-1466|2mut|as|</td><td>ggggaaactctaaaggcc</td><td>vvi-miR395n|*-10_mismatch probe</td></tr>
<tr><td>vvi|miR-1467|nat|as|</td><td>tggaggggttgggggaactc</td><td>vvi-miR395n|*0_perfect match probe</td></tr>
<tr><td>vvi|miR-1467|2mut|as|</td><td>tggaggtgttgggtgaactc</td><td>vvi-miR395n|*0_mismatch probe</td></tr>
<tr><td>vvi|miR-1468|nat|as|</td><td>atggtcatactggaggggtt</td><td>vvi-miR395n|*+10_perfect match probe</td></tr>
<tr><td>vvi|miR-1468|2mut|as|</td><td>atggccatactggagtggtt</td><td>vvi-miR395n|*+10_mismatch probe</td></tr>
<tr><td>vvi|miR-1469|nat|as|</td><td>ttcttgaactacttttccat</td><td>vvi-miR396a|*-10_perfect match probe</td></tr>
<tr><td>vvi|miR-1469|2mut|as|</td><td>ttcttaaactatttttccat</td><td>vvi-miR396a|*-10_mismatch probe</td></tr>
<tr><td>vvi|miR-1470|nat|as|</td><td>ttccacagctttcttgaact</td><td>vvi-miR396a|*0_perfect match probe</td></tr>
<tr><td>vvi|miR-1470|2mut|as|</td><td>ttccacagttttcttaaact</td><td>vvi-miR396a|*0_mismatch probe</td></tr>
<tr><td>vvi|miR-1471|nat|as|</td><td>tgtcatgcttttccacagct</td><td>vvi-miR396a|*+10_perfect match probe</td></tr>
<tr><td>vvi|miR-1471|2mut|as|</td><td>tgtcatccttttcaacagct</td><td>vvi-miR396a|*+10_mismatch probe</td></tr>
<tr><td>vvi|miR-1472|nat|as|</td><td>tcttgaacttcttctccctg</td><td>vvi-miR396b|*-10_perfect match probe</td></tr>
<tr><td>vvi|miR-1472|2mut|as|</td><td>tcttaaacttcttcttcctg</td><td>vvi-miR396b|*-10_mismatch probe</td></tr>
<tr><td>vvi|miR-1473|nat|as|</td><td>tccacagctttcttgaactt</td><td>vvi-miR396b|*0_perfect match probe</td></tr>
<tr><td>vvi|miR-1473|2mut|as|</td><td>tccacagttttcttaaactt</td><td>vvi-miR396b|*0_mismatch probe</td></tr>
<tr><td>vvi|miR-1474|nat|as|</td><td>gccatgcttttccacagctt</td><td>vvi-miR396b|*+10_perfect match probe</td></tr>
<tr><td>vvi|miR-1474|2mut|as|</td><td>gccatccttttcaacagctt</td><td>vvi-miR396b|*+10_mismatch probe</td></tr>
<tr><td>vvi|miR-1475|nat|as|</td><td>tcctgagctgttggttgaac</td><td>vvi-miR396c|*-10_perfect match probe</td></tr>
<tr><td>vvi|miR-1475|2mut|as|</td><td>tcctgagttgttcgttgaac</td><td>vvi-miR396c|*-10_mismatch probe</td></tr>
<tr><td>vvi|miR-1476|nat|as|</td><td>tccacaactttcctgagctg</td><td>vvi-miR396c|*0_perfect match probe</td></tr>
<tr><td>vvi|miR-1476|2mut|as|</td><td>tccaaaacttttctgagctg</td><td>vvi-miR396c|*0_mismatch probe</td></tr>
<tr><td>vvi|miR-1477|nat|as|</td><td>tcagtaatgttccacaactt</td><td>vvi-miR396c|*+10_perfect match probe</td></tr>
</table>

vvi|miR-1477|2mut|as|	tcagtaatcttcaacaactt
vvi|miR-1478|nat|as|	tattgaaccgcaactgttac
vvi|miR-1478|2mut|as|	tattgaacccaaactgttac
vvi|miR-1479|nat|as|	cccacagctttattgaaccg
vvi|miR-1479|2mut|as|	cccacagttttattaaaccg
vvi|miR-1480|nat|as|	ttggtatcttcccacagctt
vvi|miR-1480|2mut|as|	ttggtattttctcacagctt
vvi|miR-1481|nat|as|	agcgccaatggaatcaacca
vvi|miR-1481|2mut|as|	agcgtcaatgaaatcaacca
vvi|miR-1482|nat|as|	gattgagtgcagcgccaatg
vvi|miR-1482|2mut|as|	gattgagtgaagccccaatg
vvi|miR-1483|nat|as|	gacatgattgagtgc
vvi|miR-1483|2mut|as|	gacataattaagtgc
vvi|miR-1484|nat|as|	agcgccaatggaatcaacca
vvi|miR-1484|2mut|as|	agcgtcaatgaaatcaacca
vvi|miR-1485|nat|as|	gattgagtgcagcgccaatg
vvi|miR-1485|2mut|as|	gattgagtgaagccccaatg
vvi|miR-1486|nat|as|	gacatgattgagtgc
vvi|miR-1486|2mut|as|	gacataattaagtgc
vvi|miR-1487|nat|as|	gccactcccttggggtgt
vvi|miR-1487|2mut|as|	gccactctcttggtgtgt
vvi|miR-1488|nat|as|	gttctcaggtgccactccct
vvi|miR-1488|2mut|as|	gttctcagctgtcactccct
vvi|miR-1489|nat|as|	cacaaccggtgttctcaggt
vvi|miR-1489|2mut|as|	cacaaccagtcttctcaggt
vvi|miR-1490|nat|as|	gtcacacctgtaggacac

vvi-miR396c|*+10_mismatch probe
vvi-miR396d|*-10_perfect match probe
vvi-miR396d|*-10_mismatch probe
vvi-miR396d|*0_perfect match probe
vvi-miR396d|*0_mismatch probe
vvi-miR396d|*+10_perfect match probe
vvi-miR396d|*+10_mismatch probe
vvi-miR397a|*-10_perfect match probe
vvi-miR397a|*-10_mismatch probe
vvi-miR397a|*0_perfect match probe
vvi-miR397a|*0_mismatch probe
vvi-miR397a|*+10_perfect match probe
vvi-miR397a|*+10_mismatch probe
vvi-miR397b|*-10_perfect match probe
vvi-miR397b|*-10_mismatch probe
vvi-miR397b|*0_perfect match probe
vvi-miR397b|*0_mismatch probe
vvi-miR397b|*+10_perfect match probe
vvi-miR397b|*+10_mismatch probe
vvi-miR398a|*-10_perfect match probe
vvi-miR398a|*-10_mismatch probe
vvi-miR398a|*0_perfect match probe
vvi-miR398a|*0_mismatch probe
vvi-miR398a|*+10_perfect match probe
vvi-miR398a|*+10_mismatch probe
vvi-miR398b|*-10_perfect match

vvi|miR-1490|2mut|as| gtcacatctgtagaacac
vvi|miR-1491|nat|as| tgattctcaggtcacacctg
vvi|miR-1491|2mut|as| tgattctcacctcacacctg
vvi|miR-1492|nat|as| tgcgggcatgtgattctcag
vvi|miR-1492|2mut|as| tgcggccatgcgattctcag
vvi|miR-1493|nat|as| gtcactcctgtaggacac
vvi|miR-1493|2mut|as| gtcacttctgtagaacac
vvi|miR-1494|nat|as| tgattctcaggtcactcctg
vvi|miR-1494|2mut|as| tgattctcacctcactcctg
vvi|miR-1495|nat|as| tgcgggcatgtgattctcag
vvi|miR-1495|2mut|as| tgcggccatgcgattctcag
vvi|miR-1496|nat|as| aatcacactgttattctc
vvi|miR-1496|2mut|as| aatcaccctcttattctc
vvi|miR-1497|nat|as| gccaaaggagaatcacactg
vvi|miR-1497|2mut|as| gccaaagaagaatcccactg
vvi|miR-1498|nat|as| tgatatctctgccaaaggag
vvi|miR-1498|2mut|as| tgatatctccgtcaaaggag
vvi|miR-1499|nat|as| aggtgccctatgattgat
vvi|miR-1499|2mut|as| aggtgtcctataattgat
vvi|miR-1500|nat|as| gccaagaaagaggtgcccta
vvi|miR-1500|2mut|as| gccaagaaacaggtgtccta
vvi|miR-1501|nat|as| gccagtgcctgccaagaaag
vvi|miR-1501|2mut|as| gccagtgtctgtcaagaaag
vvi|miR-1502|nat|as| agaggccctgagaccggt
vvi|miR-1502|2mut|as| agaggtcctgagatcggt

probe
vvi-miR398b|*-10_mismatch probe
vvi-miR398b|*0_perfect match probe
vvi-miR398b|*0_mismatch probe
vvi-miR398b|*+10_perfect match probe
vvi-miR398b|*+10_mismatch probe
vvi-miR398c|*-10_perfect match probe
vvi-miR398c|*-10_mismatch probe
vvi-miR398c|*0_perfect match probe
vvi-miR398c|*0_mismatch probe
vvi-miR398c|*+10_perfect match probe
vvi-miR398c|*+10_mismatch probe
vvi-miR399a|*-10_perfect match probe
vvi-miR399a|*-10_mismatch probe
vvi-miR399a|*0_perfect match probe
vvi-miR399a|*0_mismatch probe
vvi-miR399a|*+10_perfect match probe
vvi-miR399a|*+10_mismatch probe
vvi-miR399b|*-10_perfect match probe
vvi-miR399b|*-10_mismatch probe
vvi-miR399b|*0_perfect match probe
vvi-miR399b|*0_mismatch probe
vvi-miR399b|*+10_perfect match probe
vvi-miR399b|*+10_mismatch probe
vvi-miR399c|*-10_perfect match probe
vvi-miR399c|*-10_mismatch probe

<table>
<tr><td>vvi|miR-1503|nat|as|</td><td>accaagtgaaagaggccctg</td><td>vvi-miR399c|*0_perfect match probe</td></tr>
<tr><td>vvi|miR-1503|2mut|as|</td><td>accaagcgaaagaggtcctg</td><td>vvi-miR399c|*0_mismatch probe</td></tr>
<tr><td></td><td></td><td>vvi-miR399c|*+10_perfect match</td></tr>
<tr><td>vvi|miR-1504|nat|as|</td><td>tatgtcacctaccaagtgaa</td><td>probe</td></tr>
<tr><td>vvi|miR-1504|2mut|as|</td><td>tatgtcatctacaaagtgaa</td><td>vvi-miR399c|*+10_mismatch probe</td></tr>
<tr><td></td><td></td><td>vvi-miR399d|*-10_perfect match</td></tr>
<tr><td>vvi|miR-1505|nat|as|</td><td>aaatctgctctataatttac</td><td>probe</td></tr>
<tr><td>vvi|miR-1505|2mut|as|</td><td>aaatctcctccataatttac</td><td>vvi-miR399d|*-10_mismatch probe</td></tr>
<tr><td>vvi|miR-1506|nat|as|</td><td>ctgccaaaagaaatctgctc</td><td>vvi-miR399d|*0_perfect match probe</td></tr>
<tr><td>vvi|miR-1506|2mut|as|</td><td>ctgcaaaaagaaatccgctc</td><td>vvi-miR399d|*0_mismatch probe</td></tr>
<tr><td></td><td></td><td>vvi-miR399d|*+10_perfect match</td></tr>
<tr><td>vvi|miR-1507|nat|as|</td><td>tgatcgccatctgccaaaag</td><td>probe</td></tr>
<tr><td>vvi|miR-1507|2mut|as|</td><td>tgatccccatctgtcaaaag</td><td>vvi-miR399d|*+10_mismatch probe</td></tr>
<tr><td></td><td></td><td>vvi-miR399e|*-10_perfect match</td></tr>
<tr><td>vvi|miR-1508|nat|as|</td><td>taatttgccctgtaatatgc</td><td>probe</td></tr>
<tr><td>vvi|miR-1508|2mut|as|</td><td>taatttgtcctctaatatgc</td><td>vvi-miR399e|*-10_mismatch probe</td></tr>
<tr><td>vvi|miR-1509|nat|as|</td><td>tgccaaaagataatttgccc</td><td>vvi-miR399e|*0_perfect match probe</td></tr>
<tr><td>vvi|miR-1509|2mut|as|</td><td>tgccaaaacataattcgccc</td><td>vvi-miR399e|*0_mismatch probe</td></tr>
<tr><td></td><td></td><td>vvi-miR399e|*+10_perfect match</td></tr>
<tr><td>vvi|miR-1510|nat|as|</td><td>agtggctgcctgccaaaaga</td><td>probe</td></tr>
<tr><td>vvi|miR-1510|2mut|as|</td><td>agtggctgtctgtcaaaaga</td><td>vvi-miR399e|*+10_mismatch probe</td></tr>
<tr><td></td><td></td><td>vvi-miR399f|*-10_perfect match</td></tr>
<tr><td>vvi|miR-1511|nat|as|</td><td>ccctctaatgcaac</td><td>probe</td></tr>
<tr><td>vvi|miR-1511|2mut|as|</td><td>ccctttaatccaac</td><td>vvi-miR399f|*-10_mismatch probe</td></tr>
<tr><td>vvi|miR-1512|nat|as|</td><td>gcagatttggccctctaatg</td><td>vvi-miR399f|*0_perfect match probe</td></tr>
<tr><td>vvi|miR-1512|2mut|as|</td><td>gcagatttgctcctctaatg</td><td>vvi-miR399f|*0_mismatch probe</td></tr>
<tr><td></td><td></td><td>vvi-miR399f|*+10_perfect match</td></tr>
<tr><td>vvi|miR-1513|nat|as|</td><td>tcatgccaaagcagatttgg</td><td>probe</td></tr>
<tr><td>vvi|miR-1513|2mut|as|</td><td>tcatgtcaaagaagatttgg</td><td>vvi-miR399f|*+10_mismatch probe</td></tr>
<tr><td></td><td></td><td>vvi-miR399g|*-10_perfect match</td></tr>
<tr><td>vvi|miR-1514|nat|as|</td><td>agtattgcccagcaattcat</td><td>probe</td></tr>
<tr><td>vvi|miR-1514|2mut|as|</td><td>agtattgtccagaaattcat</td><td>vvi-miR399g|*-10_mismatch probe</td></tr>
<tr><td>vvi|miR-1515|nat|as|</td><td>ctgccaatggagtattgccc</td><td>vvi-miR399g|*0_perfect match probe</td></tr>
<tr><td>vvi|miR-1515|2mut|as|</td><td>ctgccaatcgactattgccc</td><td>vvi-miR399g|*0_mismatch probe</td></tr>
</table>

<table>
<tr><td></td><td></td><td>vvi-miR399g|*+10_perfect match</td></tr>
<tr><td>vvi|miR-1516|nat|as|</td><td>gagtggccaactgccaatgg</td><td>probe</td></tr>
<tr><td>vvi|miR-1516|2mut|as|</td><td>gagtggtcaactgtcaatgg</td><td>vvi-miR399g|*+10_mismatch probe</td></tr>
<tr><td></td><td></td><td>vvi-miR399h|*-10_perfect match</td></tr>
<tr><td>vvi|miR-1517|nat|as|</td><td>gattgcactgttattcct</td><td>probe</td></tr>
<tr><td>vvi|miR-1517|2mut|as|</td><td>gattgaactcttattcct</td><td>vvi-miR399h|*-10_mismatch probe</td></tr>
<tr><td>vvi|miR-1518|nat|as|</td><td>gccaaaggaggattgcactg</td><td>vvi-miR399h|*0_perfect match probe</td></tr>
<tr><td>vvi|miR-1518|2mut|as|</td><td>gccaaagaaggattgaactg</td><td>vvi-miR399h|*0_mismatch probe</td></tr>
<tr><td></td><td></td><td>vvi-miR399h|*+10_perfect match</td></tr>
<tr><td>vvi|miR-1519|nat|as|</td><td>tgatctttctgccaaaggag</td><td>probe</td></tr>
<tr><td>vvi|miR-1519|2mut|as|</td><td>tgatctttccgtcaaaggag</td><td>vvi-miR399h|*+10_mismatch probe</td></tr>
<tr><td></td><td></td><td>vvi-miR399i|*-10_perfect match</td></tr>
<tr><td>vvi|miR-1520|nat|as|</td><td>agaagccctacaactact</td><td>probe</td></tr>
<tr><td>vvi|miR-1520|2mut|as|</td><td>agaagtcctaaaactact</td><td>vvi-miR399i|*-10_mismatch probe</td></tr>
<tr><td>vvi|miR-1521|nat|as|</td><td>ccagaaggagagaagcccta</td><td>vvi-miR399i|*0_perfect match probe</td></tr>
<tr><td>vvi|miR-1521|2mut|as|</td><td>ccagaagaagagaagtccta</td><td>vvi-miR399i|*0_mismatch probe</td></tr>
<tr><td></td><td></td><td>vvi-miR399i|*+10_perfect match</td></tr>
<tr><td>vvi|miR-1522|nat|as|</td><td>ccatctcctgccagaaggag</td><td>probe</td></tr>
<tr><td>vvi|miR-1522|2mut|as|</td><td>ccatctcctctcagaaggag</td><td>vvi-miR399i|*+10_mismatch probe</td></tr>
<tr><td></td><td></td><td>vvi-miR403a|*-10_perfect match</td></tr>
<tr><td>vvi|miR-1523|nat|as|</td><td>cgcacaaacttgagatgtgc</td><td>probe</td></tr>
<tr><td>vvi|miR-1523|2mut|as|</td><td>cgcaaaaacttaagatgtgc</td><td>vvi-miR403a|*-10_mismatch probe</td></tr>
<tr><td>vvi|miR-1524|nat|as|</td><td>gtttgattcacgcacaaact</td><td>vvi-miR403a|*0_perfect match probe</td></tr>
<tr><td>vvi|miR-1524|2mut|as|</td><td>gtttgattcccccacaaact</td><td>vvi-miR403a|*0_mismatch probe</td></tr>
<tr><td></td><td></td><td>vvi-miR403a|*+10_perfect match</td></tr>
<tr><td>vvi|miR-1525|nat|as|</td><td>ctacgatggggtttgattca</td><td>probe</td></tr>
<tr><td>vvi|miR-1525|2mut|as|</td><td>ctacgatggtctttgattca</td><td>vvi-miR403a|*+10_mismatch probe</td></tr>
<tr><td></td><td></td><td>vvi-miR403b|*-10_perfect match</td></tr>
<tr><td>vvi|miR-1526|nat|as|</td><td>cacaaactcgaggtttgt</td><td>probe</td></tr>
<tr><td>vvi|miR-1526|2mut|as|</td><td>cacaaactaaaggtttgt</td><td>vvi-miR403b|*-10_mismatch probe</td></tr>
<tr><td>vvi|miR-1527|nat|as|</td><td>tggattcgcgcacaaactcg</td><td>vvi-miR403b|*0_perfect match probe</td></tr>
<tr><td>vvi|miR-1527|2mut|as|</td><td>tggattcgccaacaaactcg</td><td>vvi-miR403b|*0_mismatch probe</td></tr>
<tr><td></td><td></td><td>vvi-miR403b|*+10_perfect match</td></tr>
<tr><td>vvi|miR-1528|nat|as|</td><td>atcgaggcgttggattcgcg</td><td>probe</td></tr>
</table>

vvi|miR-1528|2mut|as|	atcgaggagttcgattcgcg	vvi-miR403b|*+10_mismatch probe
vvi|miR-1529|nat|as|	cacaaacttgagatgtgc	vvi-miR403c|*-10_perfect match probe
vvi|miR-1529|2mut|as|	cacaaatttaagatgtgc	vvi-miR403c|*-10_mismatch probe
vvi|miR-1530|nat|as|	ttgattcacgcacaaacttg	vvi-miR403c|*0_perfect match probe
vvi|miR-1530|2mut|as|	ttgattcaccaacaaacttg	vvi-miR403c|*0_mismatch probe
vvi|miR-1531|nat|as|	acgatggggtttgattcacg	vvi-miR403c|*+10_perfect match probe
vvi|miR-1531|2mut|as|	acgatggtgtttaattcacg	vvi-miR403c|*+10_mismatch probe
vvi|miR-1532|nat|as|	cacaaactcgaggtttgt	vvi-miR403d|*-10_perfect match probe
vvi|miR-1532|2mut|as|	cacaaactaaaggtttgt	vvi-miR403d|*-10_mismatch probe
vvi|miR-1533|nat|as|	tggattcgcgcacaaactcg	vvi-miR403d|*0_perfect match probe
vvi|miR-1533|2mut|as|	tggattcgccaacaaactcg	vvi-miR403d|*0_mismatch probe
vvi|miR-1534|nat|as|	attgaggggttggattcgcg	vvi-miR403d|*+10_perfect match probe
vvi|miR-1534|2mut|as|	attgaggtgttcgattcgcg	vvi-miR403d|*+10_mismatch probe
vvi|miR-1535|nat|as|	cacaaacttgagatgtgc	vvi-miR403e|*-10_perfect match probe
vvi|miR-1535|2mut|as|	cacaaatttaagatgtgc	vvi-miR403e|*-10_mismatch probe
vvi|miR-1536|nat|as|	ttgattcacgcacaaacttg	vvi-miR403e|*0_perfect match probe
vvi|miR-1536|2mut|as|	ttgattcaccaacaaacttg	vvi-miR403e|*0_mismatch probe
vvi|miR-1537|nat|as|	acgatggggtttgattcacg	vvi-miR403e|*+10_perfect match probe
vvi|miR-1537|2mut|as|	acgatggtgtttaattcacg	vvi-miR403e|*+10_mismatch probe
vvi|miR-1538|nat|as|	gcacaaactcctcatctca	vvi-miR403f|*-10_perfect match probe
vvi|miR-1538|2mut|as|	gcacaaacttttcatctca	vvi-miR403f|*-10_mismatch probe
vvi|miR-1539|nat|as|	ttagagtcacgcacaaactc	vvi-miR403f|*0_perfect match probe
vvi|miR-1539|2mut|as|	ttagagtcaaccacaaactc	vvi-miR403f|*0_mismatch probe
vvi|miR-1540|nat|as|	tcaggcggttttagagtcac	vvi-miR403f|*+10_perfect match probe
vvi|miR-1540|2mut|as|	tcaggaggttttagactcac	vvi-miR403f|*+10_mismatch probe
vvi|miR-1541|nat|as|	ctcgtccccgtcttcctctt	vvi-miR408|*-10_perfect match

<table>
<tr><td></td><td></td><td>probe</td></tr>
<tr><td>vvi|miR-1541|2mut|as|</td><td>ctcgtcccagtctttctctt</td><td>vvi-miR408|*-10_mismatch probe</td></tr>
<tr><td>vvi|miR-1542|nat|as|</td><td>catgcactacctcgtccccg</td><td>vvi-miR408|*0_perfect match probe</td></tr>
<tr><td>vvi|miR-1542|2mut|as|</td><td>catgaactacctcctccccg</td><td>vvi-miR408|*0_mismatch probe</td></tr>
<tr><td>vvi|miR-1543|nat|as|</td><td>tagttccatccatgcactac</td><td>vvi-miR408|*+10_perfect match probe</td></tr>
<tr><td>vvi|miR-1543|2mut|as|</td><td>tagttcaatccatgaactac</td><td>vvi-miR408|*+10_mismatch probe</td></tr>
<tr><td>vvi|miR-1544|nat|as|</td><td>tgtcttccaactttccaacc</td><td>vvi-miR477a|*-10_perfect match probe</td></tr>
<tr><td>vvi|miR-1544|2mut|as|</td><td>tgtcttcaaactttcaaacc</td><td>vvi-miR477a|*-10_mismatch probe</td></tr>
<tr><td>vvi|miR-1545|nat|as|</td><td>ggtcccccactgtcttccaa</td><td>vvi-miR477a|*0_perfect match probe</td></tr>
<tr><td>vvi|miR-1545|2mut|as|</td><td>ggtccctcactctcttccaa</td><td>vvi-miR477a|*0_mismatch probe</td></tr>
<tr><td>vvi|miR-1546|nat|as|</td><td>aagcttccaaggtcccccac</td><td>vvi-miR477a|*+10_perfect match probe</td></tr>
<tr><td>vvi|miR-1546|2mut|as|</td><td>aagcttcaaaggtcctccac</td><td>vvi-miR477a|*+10_mismatch probe</td></tr>
<tr><td>vvi|miR-1547|nat|as|</td><td>tcggctcgtctttgagccga</td><td>vvi-miR479|*-10_perfect match probe</td></tr>
<tr><td>vvi|miR-1547|2mut|as|</td><td>tcggctcctctttgacccga</td><td>vvi-miR479|*-10_mismatch probe</td></tr>
<tr><td>vvi|miR-1548|nat|as|</td><td>tgatattggttcggctcgtc</td><td>vvi-miR479|*0_perfect match probe</td></tr>
<tr><td>vvi|miR-1548|2mut|as|</td><td>tgatattcgttcgcctcgtc</td><td>vvi-miR479|*0_mismatch probe</td></tr>
<tr><td>vvi|miR-1549|nat|as|</td><td>catacaagagtgatattggt</td><td>vvi-miR479|*+10_perfect match probe</td></tr>
<tr><td>vvi|miR-1549|2mut|as|</td><td>cataaaagagcgatattggt</td><td>vvi-miR479|*+10_mismatch probe</td></tr>
<tr><td>vvi|miR-1550|nat|as|</td><td>ctccaattcccaaacatt</td><td>vvi-miR482a|*-10_perfect match probe</td></tr>
<tr><td>vvi|miR-1550|2mut|as|</td><td>ctccaattttcaaacatt</td><td>vvi-miR482a|*-10_mismatch probe</td></tr>
<tr><td>vvi|miR-1551|nat|as|</td><td>ctttcctactctccaattcc</td><td>vvi-miR482a|*0_perfect match probe</td></tr>
<tr><td>vvi|miR-1551|2mut|as|</td><td>cttttctactctcaaattcc</td><td>vvi-miR482a|*0_mismatch probe</td></tr>
<tr><td>vvi|miR-1552|nat|as|</td><td>gatggctaagctttcctact</td><td>vvi-miR482a|*+10_perfect match probe</td></tr>
<tr><td>vvi|miR-1552|2mut|as|</td><td>gatgcctaagttttcctact</td><td>vvi-miR482a|*+10_mismatch probe</td></tr>
<tr><td>vvi|miR-1553|nat|as|</td><td>agagcacgcctgtacacagt</td><td>vvi-miR535a|*-10_perfect match probe</td></tr>
<tr><td>vvi|miR-1553|2mut|as|</td><td>agagcacccctgtacccagt</td><td>vvi-miR535a|*-10_mismatch probe</td></tr>
</table>

<table>
<tr><td>vvi|miR-1554|nat|as|</td><td>acagcgagagagagcacgcc</td><td>vvi-miR535a|*0_perfect match probe</td></tr>
<tr><td>vvi|miR-1554|2mut|as|</td><td>acagagagagagagaacgcc</td><td>vvi-miR535a|*0_mismatch probe</td></tr>
<tr><td></td><td></td><td>vvi-miR535a|*+10_perfect match probe</td></tr>
<tr><td>vvi|miR-1555|nat|as|</td><td>agattgtatgacagcgagag</td><td></td></tr>
<tr><td>vvi|miR-1555|2mut|as|</td><td>agattctatgacagagagag</td><td>vvi-miR535a|*+10_mismatch probe</td></tr>
<tr><td></td><td></td><td>vvi-miR535b|*-10_perfect match probe</td></tr>
<tr><td>vvi|miR-1556|nat|as|</td><td>agagcacgcctgtacacagt</td><td></td></tr>
<tr><td>vvi|miR-1556|2mut|as|</td><td>agagcacccctgtacccagt</td><td>vvi-miR535b|*-10_mismatch probe</td></tr>
<tr><td>vvi|miR-1557|nat|as|</td><td>acagcgagagagagcacgcc</td><td>vvi-miR535b|*0_perfect match probe</td></tr>
<tr><td>vvi|miR-1557|2mut|as|</td><td>acagagagagagagaacgcc</td><td>vvi-miR535b|*0_mismatch probe</td></tr>
<tr><td></td><td></td><td>vvi-miR535b|*+10_perfect match probe</td></tr>
<tr><td>vvi|miR-1558|nat|as|</td><td>agattgtatgacagcgagag</td><td></td></tr>
<tr><td>vvi|miR-1558|2mut|as|</td><td>agattctatgacagagagag</td><td>vvi-miR535b|*+10_mismatch probe</td></tr>
<tr><td></td><td></td><td>vvi-miR535c|*-10_perfect match probe</td></tr>
<tr><td>vvi|miR-1559|nat|as|</td><td>agagcacgcctgtacacagt</td><td></td></tr>
<tr><td>vvi|miR-1559|2mut|as|</td><td>agagcacccctgtacccagt</td><td>vvi-miR535c|*-10_mismatch probe</td></tr>
<tr><td>vvi|miR-1560|nat|as|</td><td>acagcgagagagagcacgcc</td><td>vvi-miR535c|*0_perfect match probe</td></tr>
<tr><td>vvi|miR-1560|2mut|as|</td><td>acagagagagagagaacgcc</td><td>vvi-miR535c|*0_mismatch probe</td></tr>
<tr><td></td><td></td><td>vvi-miR535c|*+10_perfect match probe</td></tr>
<tr><td>vvi|miR-1561|nat|as|</td><td>agattgtatgacagcgagag</td><td></td></tr>
<tr><td>vvi|miR-1561|2mut|as|</td><td>agattctatgacagagagag</td><td>vvi-miR535c|*+10_mismatch probe</td></tr>
<tr><td></td><td></td><td>vvi-miR535d|*-10_perfect match probe</td></tr>
<tr><td>vvi|miR-1562|nat|as|</td><td>agagcacgcctgtacacagt</td><td></td></tr>
<tr><td>vvi|miR-1562|2mut|as|</td><td>agagcacccctgtacccagt</td><td>vvi-miR535d|*-10_mismatch probe</td></tr>
<tr><td>vvi|miR-1563|nat|as|</td><td>acagcgacagagagcacgcc</td><td>vvi-miR535d|*0_perfect match probe</td></tr>
<tr><td>vvi|miR-1563|2mut|as|</td><td>acagagacagagagaacgcc</td><td>vvi-miR535d|*0_mismatch probe</td></tr>
<tr><td></td><td></td><td>vvi-miR535d|*+10_perfect match probe</td></tr>
<tr><td>vvi|miR-1564|nat|as|</td><td>agattgtatgacagcgacag</td><td></td></tr>
<tr><td>vvi|miR-1564|2mut|as|</td><td>agattctatgacagagacag</td><td>vvi-miR535d|*+10_mismatch probe</td></tr>
<tr><td></td><td></td><td>vvi-miR535e|*-10_perfect match probe</td></tr>
<tr><td>vvi|miR-1565|nat|as|</td><td>agagcacgcctgtacacagt</td><td></td></tr>
<tr><td>vvi|miR-1565|2mut|as|</td><td>agagcacccctgtacccagt</td><td>vvi-miR535e|*-10_mismatch probe</td></tr>
<tr><td>vvi|miR-1566|nat|as|</td><td>acagcgacagagagcacgcc</td><td>vvi-miR535e|*0_perfect match probe</td></tr>
<tr><td>vvi|miR-1566|2mut|as|</td><td>acagagacagagagaacgcc</td><td>vvi-miR535e|*0_mismatch probe</td></tr>
</table>

<table>
<tr><td>vvi|miR-1567|nat|as|</td><td>agattgtatgacagcgacag</td><td>vvi-miR535e|*+10_perfect match probe</td></tr>
<tr><td>vvi|miR-1567|2mut|as|</td><td>agattctatgacagagacag</td><td>vvi-miR535e|*+10_mismatch probe</td></tr>
<tr><td>vvi|miR-1568|nat|as|</td><td>gagcatctcaacaacaatcc</td><td>vvi-miR828a|*-10_perfect match probe</td></tr>
<tr><td>vvi|miR-1568|2mut|as|</td><td>gagcatctaaacaaaaatcc</td><td>vvi-miR828a|*-10_mismatch probe</td></tr>
<tr><td>vvi|miR-1569|nat|as|</td><td>ttcctcaaatgagcatctca</td><td>vvi-miR828a|*0_perfect match probe</td></tr>
<tr><td>vvi|miR-1569|2mut|as|</td><td>ttcctaaaatgagaatctca</td><td>vvi-miR828a|*0_mismatch probe</td></tr>
<tr><td>vvi|miR-1570|nat|as|</td><td>ttaaggttgcttcctcaaat</td><td>vvi-miR828a|*+10_perfect match probe</td></tr>
<tr><td>vvi|miR-1570|2mut|as|</td><td>ttaaggttcctttctcaaat</td><td>vvi-miR828a|*+10_mismatch probe</td></tr>
<tr><td>vvi|miR-1571|nat|as|</td><td>tgactatccaggttgatgtg</td><td>vvi-miR828b|*-10_perfect match probe</td></tr>
<tr><td>vvi|miR-1571|2mut|as|</td><td>tgactatcaagcttgatgtg</td><td>vvi-miR828b|*-10_mismatch probe</td></tr>
<tr><td>vvi|miR-1572|nat|as|</td><td>ttgcaggaaatgactatcca</td><td>vvi-miR828b|*0_perfect match probe</td></tr>
<tr><td>vvi|miR-1572|2mut|as|</td><td>ttgcagaaaatgcctatcca</td><td>vvi-miR828b|*0_mismatch probe</td></tr>
<tr><td>vvi|miR-1573|nat|as|</td><td>aaacctggttttgcaggaaa</td><td>vvi-miR828b|*+10_perfect match probe</td></tr>
<tr><td>vvi|miR-1573|2mut|as|</td><td>aaacctcgttttgaaggaaa</td><td>vvi-miR828b|*+10_mismatch probe</td></tr>
<tr><td>vvi|miR-1574|nat|as|</td><td>aggaactaatcaagtgaaa</td><td>vvi-miR845a|*-10_perfect match probe</td></tr>
<tr><td>vvi|miR-1574|2mut|as|</td><td>aggaattaatcaagcgaaa</td><td>vvi-miR845a|*-10_mismatch probe</td></tr>
<tr><td>vvi|miR-1575|nat|as|</td><td>taactcatgaaggaactaat</td><td>vvi-miR845a|*0_perfect match probe</td></tr>
<tr><td>vvi|miR-1575|2mut|as|</td><td>taactcataaagaaactaat</td><td>vvi-miR845a|*0_mismatch probe</td></tr>
<tr><td>vvi|miR-1576|nat|as|</td><td>ttacttgcaataactcatga</td><td>vvi-miR845a|*+10_perfect match probe</td></tr>
<tr><td>vvi|miR-1576|2mut|as|</td><td>ttacttgaaataactaatga</td><td>vvi-miR845a|*+10_mismatch probe</td></tr>
<tr><td>vvi|miR-1577|nat|as|</td><td>aggaactaatcaagtgaaa</td><td>vvi-miR845b|*-10_perfect match probe</td></tr>
<tr><td>vvi|miR-1577|2mut|as|</td><td>aggaattaatcaagcgaaa</td><td>vvi-miR845b|*-10_mismatch probe</td></tr>
<tr><td>vvi|miR-1578|nat|as|</td><td>taactcatgaaggaactaat</td><td>vvi-miR845b|*0_perfect match probe</td></tr>
<tr><td>vvi|miR-1578|2mut|as|</td><td>taactcataaagaaactaat</td><td>vvi-miR845b|*0_mismatch probe</td></tr>
<tr><td>vvi|miR-1579|nat|as|</td><td>ttgcttgaaataactcatga</td><td>vvi-miR845b|*+10_perfect match probe</td></tr>
</table>

vvi|miR-1579|2mut|as|	ttgcttaaaataactaatga	vvi-miR845b|*+10_mismatch probe
vvi|miR-1580|nat|as|	aaccaaaacacataattg	vvi-miR845c|*-10_perfect match probe
vvi|miR-1580|2mut|as|	aaccaaaaaccataattg	vvi-miR845c|*-10_mismatch probe
vvi|miR-1581|nat|as|	aaactcagacaaccaaaaca	vvi-miR845c|*0_perfect match probe
vvi|miR-1581|2mut|as|	aaactcagaaaacaaaaaca	vvi-miR845c|*0_mismatch probe
vvi|miR-1582|nat|as|	tgccaacatgaaactcagac	vvi-miR845c|*+10_perfect match probe
vvi|miR-1582|2mut|as|	tgccaaaatgaaactaagac	vvi-miR845c|*+10_mismatch probe
vvi|miR-1583|nat|as|	ataattaatgttggttat	vvi-miR845d|*-10_perfect match probe
vvi|miR-1583|2mut|as|	ataattaacgttcgttat	vvi-miR845d|*-10_mismatch probe
vvi|miR-1584|nat|as|	ggccctagggataattaatg	vvi-miR845d|*0_perfect match probe
vvi|miR-1584|2mut|as|	ggccctagtgctaattaatg	vvi-miR845d|*0_mismatch probe
vvi|miR-1585|nat|as|	tttatcccatggccctaggg	vvi-miR845d|*+10_perfect match probe
vvi|miR-1585|2mut|as|	tttatctcatggtcctaggg	vvi-miR845d|*+10_mismatch probe
vvi|miR-1586|nat|as|	ataattaatgtgcgttct	vvi-miR845e|*-10_perfect match probe
vvi|miR-1586|2mut|as|	ataattaacgtgagttct	vvi-miR845e|*-10_mismatch probe
vvi|miR-1587|nat|as|	ggccctagggataattaatg	vvi-miR845e|*0_perfect match probe
vvi|miR-1587|2mut|as|	ggccctagtgctaattaatg	vvi-miR845e|*0_mismatch probe
vvi|miR-1588|nat|as|	tttatcccatggccctaggg	vvi-miR845e|*+10_perfect match probe
vvi|miR-1588|2mut|as|	tttatctcatggtcctaggg	vvi-miR845e|*+10_mismatch probe
vvi|miR-1589|nat|as|	gagtctttagctgatgctgg	vvi-miR156a|ctrl_perfect match probe
vvi|miR-1589|2mut|as|	gagtctttacctgatcctgg	vvi-miR156a|ctrl_mismatch probe
vvi|miR-1590|nat|as|	taaacttatacaattgcctc	vvi-miR156b|ctrl_perfect match probe
vvi|miR-1590|2mut|as|	taaatttatacaattccctc	vvi-miR156b|ctrl_mismatch probe
vvi|miR-1591|nat|as|	acccatgcaagaacagacca	vvi-miR156c|ctrl_perfect match probe
vvi|miR-1591|2mut|as|	acccatgaaagaaaagacca	vvi-miR156c|ctrl_mismatch probe

vvi|miR-1592|nat|as|	acccttatacaaattgcctg	vvi-miR156d|ctrl_perfect match probe
vvi|miR-1592|2mut|as|	acccttataaaaattccctg	vvi-miR156d|ctrl_mismatch probe
vvi|miR-1593|nat|as|	ttcatgcaagaaaagaacca	vvi-miR156e|ctrl_perfect match probe
vvi|miR-1593|2mut|as|	ttcatgaaagaaaacaacca	vvi-miR156e|ctrl_mismatch probe
vvi|miR-1594|nat|as|	agccctcttgggtatgttca	vvi-miR156f|ctrl_perfect match probe
vvi|miR-1594|2mut|as|	agccctcttctgtatgttca	vvi-miR156f|ctrl_mismatch probe
vvi|miR-1595|nat|as|	aaactccatgcacatcatca	vvi-miR156g|ctrl_perfect match probe
vvi|miR-1595|2mut|as|	aaactccatcaacatcatca	vvi-miR156g|ctrl_mismatch probe
vvi|miR-1596|nat|as|	gttgtaattgttttcccacc	vvi-miR156h|ctrl_perfect match probe
vvi|miR-1596|2mut|as|	gttgtaattcttttctcacc	vvi-miR156h|ctrl_mismatch probe
vvi|miR-1597|nat|as|	tggctccttgcatttcatca	vvi-miR156i|ctrl_perfect match probe
vvi|miR-1597|2mut|as|	tggctccttcaatttcatca	vvi-miR156i|ctrl_mismatch probe
vvi|miR-1598|nat|as|	agaaagatcaaggcaaccac	vvi-miR159a|ctrl_perfect match probe
vvi|miR-1598|2mut|as|	agaaagataaagccaaccac	vvi-miR159a|ctrl_mismatch probe
vvi|miR-1599|nat|as|	agaaaagataaaagcaaaca	vvi-miR159b|ctrl_perfect match probe
vvi|miR-1599|2mut|as|	agaaaacataaaagaaaaca	vvi-miR159b|ctrl_mismatch probe
vvi|miR-1600|nat|as|	ggatctaaacttggataatg	vvi-miR159c|ctrl_perfect match probe
vvi|miR-1600|2mut|as|	ggatctaaatttcgataatg	vvi-miR159c|ctrl_mismatch probe
vvi|miR-1601|nat|as|	acaaccttgacaagcttctt	vvi-miR160a|ctrl_perfect match probe
vvi|miR-1601|2mut|as|	acaatcttgacaagtttctt	vvi-miR160a|ctrl_mismatch probe
vvi|miR-1602|nat|as|	cactctttaacaagcttctt	vvi-miR160b|ctrl_perfect match probe
vvi|miR-1602|2mut|as|	cactttttaacaagtttctt	vvi-miR160b|ctrl_mismatch probe
vvi|miR-1603|nat|as|	ggaaagggagtggtctctac	vvi-miR160c|ctrl_perfect match probe
vvi|miR-1603|2mut|as|	ggaaagtgagtcgtctctac	vvi-miR160c|ctrl_mismatch probe

		vvi-miR160d|ctrl_perfect match
vvi|miR-1604|nat|as|	aatcctcgggtggactttgc	probe
vvi|miR-1604|2mut|as|	aatcctcaggtcgactttgc	vvi-miR160d|ctrl_mismatch probe
		vvi-miR160e|ctrl_perfect match
vvi|miR-1605|nat|as|	cactctttaacaagcttctt	probe
vvi|miR-1605|2mut|as|	cactttttaacaagtttctt	vvi-miR160e|ctrl_mismatch probe
vvi|miR-1606|nat|as|	gatgtgccggtgggctctgc	vvi-miR160f|ctrl_perfect match probe
vvi|miR-1606|2mut|as|	gatgtgtcggtggcctctgc	vvi-miR160f|ctrl_mismatch probe
vvi|miR-1607|nat|as|	attcttgatctgcttttttc	vvi-miR162|ctrl_perfect match probe
vvi|miR-1607|2mut|as|	attcttaatctccttttttc	vvi-miR162|ctrl_mismatch probe
		vvi-miR164a|ctrl_perfect match
vvi|miR-1608|nat|as|	aagggggaaaagtgaggcaa	probe
vvi|miR-1608|2mut|as|	aagggtgaaaagtgacgcaa	vvi-miR164a|ctrl_mismatch probe
		vvi-miR164b|ctrl_perfect match
vvi|miR-1609|nat|as|	cagatccaggtgctgattga	probe
vvi|miR-1609|2mut|as|	cagatcaaggtcctgattga	vvi-miR164b|ctrl_mismatch probe
		vvi-miR164d|ctrl_perfect match
vvi|miR-1610|nat|as|	agagcagattagaatttgtg	probe
vvi|miR-1610|2mut|as|	agagaagattacaatttgtg	vvi-miR164d|ctrl_mismatch probe
		vvi-miR166c|ctrl_perfect match
vvi|miR-1611|nat|as|	caccaacacatgctcagtac	probe
vvi|miR-1611|2mut|as|	caccaacccatcctcagtac	vvi-miR166c|ctrl_mismatch probe
		vvi-miR166d|ctrl_perfect match
vvi|miR-1612|nat|as|	cactaacacataatcagagg	probe
vvi|miR-1612|2mut|as|	cactaacccataatccgagg	vvi-miR166d|ctrl_mismatch probe
		vvi-miR166e|ctrl_perfect match
vvi|miR-1613|nat|as|	caccaacccatgatcaaaga	probe
vvi|miR-1613|2mut|as|	caccaactcatgataaaaga	vvi-miR166e|ctrl_mismatch probe
vvi|miR-1614|nat|as|	tttttgaggtatacataaaa	vvi-miR166f|ctrl_perfect match probe
vvi|miR-1614|2mut|as|	tttttgagctataaataaaa	vvi-miR166f|ctrl_mismatch probe
		vvi-miR166g|ctrl_perfect match
vvi|miR-1615|nat|as|	cattcaaacgctgcttgagc	probe
vvi|miR-1615|2mut|as|	cattcaaaccctccttgagc	vvi-miR166g|ctrl_mismatch probe
vvi|miR-1616|nat|as|	cattcagattatgtctgtac	vvi-miR166h|ctrl_perfect match

<table>
<tr><td></td><td></td><td>probe</td></tr>
<tr><td>vvi|miR-1616|2mut|as|</td><td>cattccgattatctctgtac</td><td>vvi-miR166h|ctrl_mismatch probe</td></tr>
<tr><td></td><td></td><td>vvi-miR166a|ctrl_perfect match</td></tr>
<tr><td>vvi|miR-1617|nat|as|</td><td>caaccgattatgaagtaact</td><td>probe</td></tr>
<tr><td>vvi|miR-1617|2mut|as|</td><td>caacagattataaagtaact</td><td>vvi-miR166a|ctrl_mismatch probe</td></tr>
<tr><td></td><td></td><td>vvi-miR166b|ctrl_perfect match</td></tr>
<tr><td>vvi|miR-1618|nat|as|</td><td>cttcacttatatatcttctt</td><td>probe</td></tr>
<tr><td>vvi|miR-1618|2mut|as|</td><td>cttcccttatatattttctt</td><td>vvi-miR166b|ctrl_mismatch probe</td></tr>
<tr><td></td><td></td><td>vvi-miR167a|ctrl_perfect match</td></tr>
<tr><td>vvi|miR-1619|nat|as|</td><td>gaaagagatggtgtttgttt</td><td>probe</td></tr>
<tr><td>vvi|miR-1619|2mut|as|</td><td>gaaagagatcctgtttgttt</td><td>vvi-miR167a|ctrl_mismatch probe</td></tr>
<tr><td></td><td></td><td>vvi-miR167b|ctrl_perfect match</td></tr>
<tr><td>vvi|miR-1620|nat|as|</td><td>aaagggtgggcaacagaaaa</td><td>probe</td></tr>
<tr><td>vvi|miR-1620|2mut|as|</td><td>aaagggtggcaaacagaaaa</td><td>vvi-miR167b|ctrl_mismatch probe</td></tr>
<tr><td></td><td></td><td>vvi-miR167c|ctrl_perfect match</td></tr>
<tr><td>vvi|miR-1621|nat|as|</td><td>ccttgacttgtatagggaag</td><td>probe</td></tr>
<tr><td>vvi|miR-1621|2mut|as|</td><td>ccttgacttctatagtgaag</td><td>vvi-miR167c|ctrl_mismatch probe</td></tr>
<tr><td></td><td></td><td>vvi-miR167d|ctrl_perfect match</td></tr>
<tr><td>vvi|miR-1622|nat|as|</td><td>ttctctgtatccctagccaa</td><td>probe</td></tr>
<tr><td>vvi|miR-1622|2mut|as|</td><td>ttctctctatctctagccaa</td><td>vvi-miR167d|ctrl_mismatch probe</td></tr>
<tr><td></td><td></td><td>vvi-miR167e|ctrl_perfect match</td></tr>
<tr><td>vvi|miR-1623|nat|as|</td><td>ctgttcacaaagggaagcaa</td><td>probe</td></tr>
<tr><td>vvi|miR-1623|2mut|as|</td><td>ctgttcccaaagtgaagcaa</td><td>vvi-miR167e|ctrl_mismatch probe</td></tr>
<tr><td>vvi|miR-1624|nat|as|</td><td>cgctgccggagcggcgaagt</td><td>vvi-miR168|ctrl_perfect match probe</td></tr>
<tr><td>vvi|miR-1624|2mut|as|</td><td>cgctgtcggagcggagaagt</td><td>vvi-miR168|ctrl_mismatch probe</td></tr>
<tr><td></td><td></td><td>vvi-miR169a|ctrl_perfect match</td></tr>
<tr><td>vvi|miR-1625|nat|as|</td><td>tctcttctcactatatataa</td><td>probe</td></tr>
<tr><td>vvi|miR-1625|2mut|as|</td><td>tctcttctccttatatataa</td><td>vvi-miR169a|ctrl_mismatch probe</td></tr>
<tr><td></td><td></td><td>vvi-miR169y|ctrl_perfect match</td></tr>
<tr><td>vvi|miR-1626|nat|as|</td><td>attttgaaaccttaaggagg</td><td>probe</td></tr>
<tr><td>vvi|miR-1626|2mut|as|</td><td>attttgaaatcttaacgagg</td><td>vvi-miR169y|ctrl_mismatch probe</td></tr>
<tr><td></td><td></td><td>vvi-miR169b|ctrl_perfect match</td></tr>
<tr><td>vvi|miR-1627|nat|as|</td><td>tctccaactcttgctgcaga</td><td>probe</td></tr>
<tr><td>vvi|miR-1627|2mut|as|</td><td>tctcaaactcttgctccaga</td><td>vvi-miR169b|ctrl_mismatch probe</td></tr>
</table>

vvi|miR-1628|nat|as|	gattaacgaggaatgcccca	vvi-miR169c|ctrl_perfect match probe
vvi|miR-1628|2mut|as|	gattaaagaggaatgtccca	vvi-miR169c|ctrl_mismatch probe
vvi|miR-1629|nat|as|	tctcttctcactatatataa	vvi-miR169d|ctrl_perfect match probe
vvi|miR-1629|2mut|as|	tctcttctccttatatataa	vvi-miR169d|ctrl_mismatch probe
vvi|miR-1630|nat|as|	aaattgacccacttgggata	vvi-miR169e|ctrl_perfect match probe
vvi|miR-1630|2mut|as|	aaattgactcacttgtgata	vvi-miR169e|ctrl_mismatch probe
vvi|miR-1631|nat|as|	attaacgatcgagcaaatta	vvi-miR169f|ctrl_perfect match probe
vvi|miR-1631|2mut|as|	attaaagatcgagaaaatta	vvi-miR169f|ctrl_mismatch probe
vvi|miR-1632|nat|as|	ggcagatggctaagtagaga	vvi-miR169g|ctrl_perfect match probe
vvi|miR-1632|2mut|as|	ggcagatgcctaactagaga	vvi-miR169g|ctrl_mismatch probe
vvi|miR-1633|nat|as|	atgcctcaaatagtgacaaa	vvi-miR169h|ctrl_perfect match probe
vvi|miR-1633|2mut|as|	atgcttcaaatagcgacaaa	vvi-miR169h|ctrl_mismatch probe
vvi|miR-1634|nat|as|	cctcaaataccaactgaaaa	vvi-miR169i|ctrl_perfect match probe
vvi|miR-1634|2mut|as|	cctcaaatataaactgaaaa	vvi-miR169i|ctrl_mismatch probe
vvi|miR-1635|nat|as|	tcattccactctatatgtga	vvi-miR169j|ctrl_perfect match probe
vvi|miR-1635|2mut|as|	tcattcaactttatatgtga	vvi-miR169j|ctrl_mismatch probe
vvi|miR-1636|nat|as|	tcattccactctatatgtga	vvi-miR169k|ctrl_perfect match probe
vvi|miR-1636|2mut|as|	tcattcaactttatatgtga	vvi-miR169k|ctrl_mismatch probe
vvi|miR-1637|nat|as|	agcttcaatgcttgatgcaa	vvi-miR169l|ctrl_perfect match probe
vvi|miR-1637|2mut|as|	agcttcaatctttgatgcaa	vvi-miR169l|ctrl_mismatch probe
vvi|miR-1638|nat|as|	accttaatgccttgctgcag	vvi-miR169m|ctrl_perfect match probe
vvi|miR-1638|2mut|as|	accttaatgtcttcctgcag	vvi-miR169m|ctrl_mismatch probe
vvi|miR-1639|nat|as|	aaccctatgccttgctgcag	vvi-miR169n|ctrl_perfect match probe
vvi|miR-1639|2mut|as|	aaccctatgtcttcctgcag	vvi-miR169n|ctrl_mismatch probe
vvi|miR-1640|nat|as|	agcttcaatgcttgctgctg	vvi-miR169o|ctrl_perfect match probe

vvi|miR-1640|2mut|as|	agcttcaatccttcctgctg	vvi-miR169o|ctrl_mismatch probe
		vvi-miR169p|ctrl_perfect match
vvi|miR-1641|nat|as|	accttaatgccttgctgcag	probe
vvi|miR-1641|2mut|as|	accttaatgtcttcctgcag	vvi-miR169p|ctrl_mismatch probe
		vvi-miR169q|ctrl_perfect match
vvi|miR-1642|nat|as|	ttaatatagacttactgcaa	probe
vvi|miR-1642|2mut|as|	ttaatatagccttaccgcaa	vvi-miR169q|ctrl_mismatch probe
vvi|miR-1643|nat|as|	gcatgccttctgcaaatata	vvi-miR169r|ctrl_perfect match probe
vvi|miR-1643|2mut|as|	gcatgtcttctgaaaatata	vvi-miR169r|ctrl_mismatch probe
vvi|miR-1644|nat|as|	tcgatggtgcctaatgccaa	vvi-miR169s|ctrl_perfect match probe
vvi|miR-1644|2mut|as|	tcgatggtgtctaatcccaa	vvi-miR169s|ctrl_mismatch probe
vvi|miR-1645|nat|as|	caagtccttccgcaaatata	vvi-miR169t|ctrl_perfect match probe
vvi|miR-1645|2mut|as|	caagtcctttcccaaatata	vvi-miR169t|ctrl_mismatch probe
		vvi-miR169u|ctrl_perfect match
vvi|miR-1646|nat|as|	cgtgcccttctgcaaatata	probe
vvi|miR-1646|2mut|as|	cgtgtccttctgaaaatata	vvi-miR169u|ctrl_mismatch probe
		vvi-miR169v|ctrl_perfect match
vvi|miR-1647|nat|as|	gacaaagctgcctactttca	probe
vvi|miR-1647|2mut|as|	gacaaagctctctactttca	vvi-miR169v|ctrl_mismatch probe
		vvi-miR169w|ctrl_perfect match
vvi|miR-1648|nat|as|	catgagtacaataaagggag	probe
vvi|miR-1648|2mut|as|	catgagtaaaataaacggag	vvi-miR169w|ctrl_mismatch probe
		vvi-miR169x|ctrl_perfect match
vvi|miR-1649|nat|as|	agtgttttgaaacctggtgg	probe
vvi|miR-1649|2mut|as|	agtgttttaaaacctcgtgg	vvi-miR169x|ctrl_mismatch probe
		vvi-miR171a|ctrl_perfect match
vvi|miR-1650|nat|as|	aaccaaagatctcaatggtt	probe
vvi|miR-1650|2mut|as|	aaccaaacatctaaatggtt	vvi-miR171a|ctrl_mismatch probe
		vvi-miR171b|ctrl_perfect match
vvi|miR-1651|nat|as|	cacatggctaatatctcttc	probe
vvi|miR-1651|2mut|as|	cacatgcctaatatcccttc	vvi-miR171b|ctrl_mismatch probe
		vvi-miR171c|ctrl_perfect match
vvi|miR-1652|nat|as|	aaaaccagaactcaaaaaga	probe
vvi|miR-1652|2mut|as|	aaaacaagaactaaaaaaga	vvi-miR171c|ctrl_mismatch probe

vvi|miR-1653|nat|as|	aacaacagagttcttaactt	vvi-miR171d|ctrl_perfect match probe
vvi|miR-1653|2mut|as|	aacaaaagagtttttaactt	vvi-miR171d|ctrl_mismatch probe
vvi|miR-1654|nat|as|	atcgttctttacggctcttc	vvi-miR171e|ctrl_perfect match probe
vvi|miR-1654|2mut|as|	atcgtttttacgcctcttc	vvi-miR171e|ctrl_mismatch probe
vvi|miR-1655|nat|as|	gagatcagtttttacattca	vvi-miR171f|ctrl_perfect match probe
vvi|miR-1655|2mut|as|	gagatccgtttttaaattca	vvi-miR171f|ctrl_mismatch probe
vvi|miR-1656|nat|as|	gttggtgtcccccaccgatg	vvi-miR171g|ctrl_perfect match probe
vvi|miR-1656|2mut|as|	gttggtgtctcccacagatg	vvi-miR171g|ctrl_mismatch probe
vvi|miR-1657|nat|as|	atcagagtcgatacattcat	vvi-miR171h|ctrl_perfect match probe
vvi|miR-1657|2mut|as|	atcagagtagataaattcat	vvi-miR171h|ctrl_mismatch probe
vvi|miR-1658|nat|as|	gattaaaggacccaaaaccc	vvi-miR171i|ctrl_perfect match probe
vvi|miR-1658|2mut|as|	gattaaagaactcaaaaccc	vvi-miR171i|ctrl_mismatch probe
vvi|miR-1659|nat|as|	gcaaatgcacggtggtgatg	vvi-miR172a|ctrl_perfect match probe
vvi|miR-1659|2mut|as|	gcaaatgaacagtggtgatg	vvi-miR172a|ctrl_mismatch probe
vvi|miR-1660|nat|as|	gcgaaagcccggcgcggcag	vvi-miR172b|ctrl_perfect match probe
vvi|miR-1660|2mut|as|	gcgaaagtccggcgaggcag	vvi-miR172b|ctrl_mismatch probe
vvi|miR-1661|nat|as|	atcaaagagccatcggagtg	vvi-miR172c|ctrl_perfect match probe
vvi|miR-1661|2mut|as|	atcaaagagtcatcagagtg	vvi-miR172c|ctrl_mismatch probe
vvi|miR-1662|nat|as|	ctcaaaatctttggcagaga	vvi-miR172d|ctrl_perfect match probe
vvi|miR-1662|2mut|as|	ctcaaaatttttgccagaga	vvi-miR172d|ctrl_mismatch probe
vvi|miR-1663|nat|as|	tgacagccataactgaacgc	vvi-miR319b|ctrl_perfect match probe
vvi|miR-1663|2mut|as|	tgacagtcataaccgaacgc	vvi-miR319b|ctrl_mismatch probe
vvi|miR-1664|nat|as|	agggagcttaagatccaatt	vvi-miR319c|ctrl_perfect match probe
vvi|miR-1664|2mut|as|	agggagtttaagatcaaatt	vvi-miR319c|ctrl_mismatch probe

vvi|miR-1665|nat|as|	aggagcatgaagttcatcat	vvi-miR319e|ctrl_perfect match probe
vvi|miR-1665|2mut|as|	aggagaatgaagttaatcat	vvi-miR319e|ctrl_mismatch probe
vvi|miR-1666|nat|as|	agacaagaaaaggatgaaat	vvi-miR319f|ctrl_perfect match probe
vvi|miR-1666|2mut|as|	agacaacaaaagaatgaaat	vvi-miR319f|ctrl_mismatch probe
vvi|miR-1667|nat|as|	tgggcaaagcgtgcggagga	vvi-miR319g|ctrl_perfect match probe
vvi|miR-1667|2mut|as|	tgggcaaagagtgaggagga	vvi-miR319g|ctrl_mismatch probe
vvi|miR-1668|nat|as|	caatcattttcatcatggct	vvi-miR390|ctrl_perfect match probe
vvi|miR-1668|2mut|as|	caataattttcataatggct	vvi-miR390|ctrl_mismatch probe
vvi|miR-1669|nat|as|	aagagaggtggaagataacc	vvi-miR393a|ctrl_perfect match probe
vvi|miR-1669|2mut|as|	aagagaggtcaaagataacc	vvi-miR393a|ctrl_mismatch probe
vvi|miR-1670|nat|as|	gatccaaagacatggagagt	vvi-miR393b|ctrl_perfect match probe
vvi|miR-1670|2mut|as|	gatcaaaagacatcgagagt	vvi-miR393b|ctrl_mismatch probe
vvi|miR-1671|nat|as|	gaatatagacccatgatgtg	vvi-miR394a|ctrl_perfect match probe
vvi|miR-1671|2mut|as|	gaatatagattcatgatgtg	vvi-miR394a|ctrl_mismatch probe
vvi|miR-1672|nat|as|	aaaagagagattttcaagag	vvi-miR394b|ctrl_perfect match probe
vvi|miR-1672|2mut|as|	aaaagagcgattttaaagag	vvi-miR394b|ctrl_mismatch probe
vvi|miR-1673|nat|as|	aattcagaagaattggtata	vvi-miR394c|ctrl_perfect match probe
vvi|miR-1673|2mut|as|	aattccgaagaattcgtata	vvi-miR394c|ctrl_mismatch probe
vvi|miR-1674|nat|as|	gaagtcattaaaactagcga	vvi-miR395a|ctrl_perfect match probe
vvi|miR-1674|2mut|as|	gaagccattaaaattagcga	vvi-miR395a|ctrl_mismatch probe
vvi|miR-1675|nat|as|	gtaggcattcattagagaag	vvi-miR395b|ctrl_perfect match probe
vvi|miR-1675|2mut|as|	gtagccattcattagcgaag	vvi-miR395b|ctrl_mismatch probe
vvi|miR-1676|nat|as|	gaagtcattataactagaga	vvi-miR395c|ctrl_perfect match probe
vvi|miR-1676|2mut|as|	gaagccattataattagaga	vvi-miR395c|ctrl_mismatch probe

vvi|miR-1677|nat|as|	gaagtcattagagaagatcc	vvi-miR395d|ctrl_perfect match probe
vvi|miR-1677|2mut|as|	gaagccattagcgaagatcc	vvi-miR395d|ctrl_mismatch probe
vvi|miR-1678|nat|as|	gaagtcattataattagaga	vvi-miR395e|ctrl_perfect match probe
vvi|miR-1678|2mut|as|	gaagccattataattcgaga	vvi-miR395e|ctrl_mismatch probe
vvi|miR-1679|nat|as|	gaagtcattagagaagatcc	vvi-miR395f|ctrl_perfect match probe
vvi|miR-1679|2mut|as|	gaagccattagcgaagatcc	vvi-miR395f|ctrl_mismatch probe
vvi|miR-1680|nat|as|	gaattgaaaactaaggatcc	vvi-miR395g|ctrl_perfect match probe
vvi|miR-1680|2mut|as|	gaattaaaaactaagaatcc	vvi-miR395g|ctrl_mismatch probe
vvi|miR-1681|nat|as|	gaagtcattataattagaga	vvi-miR395h|ctrl_perfect match probe
vvi|miR-1681|2mut|as|	gaagccattataattcgaga	vvi-miR395h|ctrl_mismatch probe
vvi|miR-1682|nat|as|	gaagtcattacagaagatcc	vvi-miR395i|ctrl_perfect match probe
vvi|miR-1682|2mut|as|	gaagccattaaagaagatcc	vvi-miR395i|ctrl_mismatch probe
vvi|miR-1683|nat|as|	gaagtcattaaagaagatcc	vvi-miR395j|ctrl_perfect match probe
vvi|miR-1683|2mut|as|	gaagccattaaagaacatcc	vvi-miR395j|ctrl_mismatch probe
vvi|miR-1684|nat|as|	gaagtcattataattagaga	vvi-miR395k|ctrl_perfect match probe
vvi|miR-1684|2mut|as|	gaagccattataattcgaga	vvi-miR395k|ctrl_mismatch probe
vvi|miR-1685|nat|as|	gaagtcattacagaagatcc	vvi-miR395l|ctrl_perfect match probe
vvi|miR-1685|2mut|as|	gaagccattaaagaagatcc	vvi-miR395l|ctrl_mismatch probe
vvi|miR-1686|nat|as|	gaagtcattataactagaga	vvi-miR395m|ctrl_perfect match probe
vvi|miR-1686|2mut|as|	gaagccattataattagaga	vvi-miR395m|ctrl_mismatch probe
vvi|miR-1687|nat|as|	aggaaatcaccctggaaata	vvi-miR395n|ctrl_perfect match probe
vvi|miR-1687|2mut|as|	aggaaatcattctggaaata	vvi-miR395n|ctrl_mismatch probe
vvi|miR-1688|nat|as|	tcttcataaggagtgtgaaa	vvi-miR396a|ctrl_perfect match probe
vvi|miR-1688|2mut|as|	tcttcataacaagtgtgaaa	vvi-miR396a|ctrl_mismatch probe
vvi|miR-1689|nat|as|	ctcctacgtttacccatcca	vvi-miR396b|ctrl_perfect match probe

vvi|miR-1689|2mut|as|	ctcctacctttactcatcca	vvi-miR396b|ctrl_mismatch probe
		vvi-miR396c|ctrl_perfect match
vvi|miR-1690|nat|as|	atggaagtggaaggcttcca	probe
vvi|miR-1690|2mut|as|	atggaagtcgaagccttcca	vvi-miR396c|ctrl_mismatch probe
		vvi-miR396d|ctrl_perfect match
vvi|miR-1691|nat|as|	acacaccagtagtctgaaaa	probe
vvi|miR-1691|2mut|as|	acacacaagtagcctgaaaa	vvi-miR396d|ctrl_mismatch probe
		vvi-miR397a|ctrl_perfect match
vvi|miR-1692|nat|as|	tgaaaaatggaatacttcag	probe
vvi|miR-1692|2mut|as|	tgaaaaatcgaatatttcag	vvi-miR397a|ctrl_mismatch probe
		vvi-miR397b|ctrl_perfect match
vvi|miR-1693|nat|as|	tgaaaaatggaatacttcag	probe
vvi|miR-1693|2mut|as|	tgaaaaatcgaatatttcag	vvi-miR397b|ctrl_mismatch probe
		vvi-miR398a|ctrl_perfect match
vvi|miR-1694|nat|as|	tgcattagctagcagagctt	probe
vvi|miR-1694|2mut|as|	tgcattagttagaagagctt	vvi-miR398a|ctrl_mismatch probe
		vvi-miR398b|ctrl_perfect match
vvi|miR-1695|nat|as|	gagcaaagatgggtggtgcg	probe
vvi|miR-1695|2mut|as|	gagcaaacatgtgtggtgcg	vvi-miR398b|ctrl_mismatch probe
		vvi-miR398c|ctrl_perfect match
vvi|miR-1696|nat|as|	gagcaaagatgggtgatgcg	probe
vvi|miR-1696|2mut|as|	gagcaaacatgtgtgatgcg	vvi-miR398c|ctrl_mismatch probe
		vvi-miR399a|ctrl_perfect match
vvi|miR-1697|nat|as|	cagctaccgccttctactcc	probe
vvi|miR-1697|2mut|as|	cagctacccctttctactcc	vvi-miR399a|ctrl_mismatch probe
		vvi-miR399b|ctrl_perfect match
vvi|miR-1698|nat|as|	cacatctttttactgcagct	probe
vvi|miR-1698|2mut|as|	cacattttttactgaagct	vvi-miR399b|ctrl_mismatch probe
		vvi-miR399c|ctrl_perfect match
vvi|miR-1699|nat|as|	cattcacatctatgtcacct	probe
vvi|miR-1699|2mut|as|	cattcccatctatctcacct	vvi-miR399c|ctrl_mismatch probe
		vvi-miR399d|ctrl_perfect match
vvi|miR-1700|nat|as|	acactcaatgccctttgagc	probe
vvi|miR-1700|2mut|as|	acactcaatctcctttgagc	vvi-miR399d|ctrl_mismatch probe

vvi|miR-1701|nat|as|	ggcacactacaagaatgcat	vvi-miR399e|ctrl_perfect match probe
vvi|miR-1701|2mut|as|	ggcaccctacaacaatgcat	vvi-miR399e|ctrl_mismatch probe
vvi|miR-1702|nat|as|	agaagactgcagacacagac	vvi-miR399f|ctrl_perfect match probe
vvi|miR-1702|2mut|as|	agaagactgaagacccagac	vvi-miR399f|ctrl_mismatch probe
vvi|miR-1703|nat|as|	agcttagtctgctacttgtg	vvi-miR399g|ctrl_perfect match probe
vvi|miR-1703|2mut|as|	agcttagcctcctacttgtg	vvi-miR399g|ctrl_mismatch probe
vvi|miR-1704|nat|as|	cagaagtatgcatgtgcatg	vvi-miR399h|ctrl_perfect match probe
vvi|miR-1704|2mut|as|	cagaagtatccatgtccatg	vvi-miR399h|ctrl_mismatch probe
vvi|miR-1705|nat|as|	cattggaagttaatcaacac	vvi-miR399i|ctrl_perfect match probe
vvi|miR-1705|2mut|as|	cattcgaagttaataaacac	vvi-miR399i|ctrl_mismatch probe
vvi|miR-1706|nat|as|	cgtcgaatcgccccgtgatt	vvi-miR403a|ctrl_perfect match probe
vvi|miR-1706|2mut|as|	cgtcgaatcctcccgtgatt	vvi-miR403a|ctrl_mismatch probe
vvi|miR-1707|nat|as|	ccctttgaaagaagatcgag	vvi-miR403b|ctrl_perfect match probe
vvi|miR-1707|2mut|as|	ccctttaaaagaacatcgag	vvi-miR403b|ctrl_mismatch probe
vvi|miR-1708|nat|as|	cgtcgaatcgccccgtgatt	vvi-miR403c|ctrl_perfect match probe
vvi|miR-1708|2mut|as|	cgtcgaatcctcccgtgatt	vvi-miR403c|ctrl_mismatch probe
vvi|miR-1709|nat|as|	ccctttgaaagaagattgag	vvi-miR403d|ctrl_perfect match probe
vvi|miR-1709|2mut|as|	ccctttaaaagaacattgag	vvi-miR403d|ctrl_mismatch probe
vvi|miR-1710|nat|as|	cgtcgaatcgccccgtgatt	vvi-miR403e|ctrl_perfect match probe
vvi|miR-1710|2mut|as|	cgtcgaatcctcccgtgatt	vvi-miR403e|ctrl_mismatch probe
vvi|miR-1711|nat|as|	catagaaaaggtatcgcatc	vvi-miR403f|ctrl_perfect match probe
vvi|miR-1711|2mut|as|	catagaaaacgtatcccatc	vvi-miR403f|ctrl_mismatch probe
vvi|miR-1712|nat|as|	tagagcaaaaacagcttaac	vvi-miR408|ctrl_perfect match probe
vvi|miR-1712|2mut|as|	tagagaaaaaacagtttaac	vvi-miR408|ctrl_mismatch probe
vvi|miR-1713|nat|as|	accttgataatcagccagga	vvi-miR477a|ctrl_perfect match probe

vvi|miR-1713|2mut|as|	accttaataatcagtcagga	vvi-miR477a|ctrl_mismatch probe
vvi|miR-1714|nat|as|	gccgatgaaaatgaatgaaa	vvi-miR479|ctrl_perfect match probe
vvi|miR-1714|2mut|as|	gccgataaaaataaatgaaa	vvi-miR479|ctrl_mismatch probe
vvi|miR-1715|nat|as|	gactagctctagaccccaa	vvi-miR482a|ctrl_perfect match probe
vvi|miR-1715|2mut|as|	gactagttctagacctccaa	vvi-miR482a|ctrl_mismatch probe
vvi|miR-1716|nat|as|	tgttcctcgcatgaatgctg	vvi-miR535a|ctrl_perfect match probe
vvi|miR-1716|2mut|as|	tgttcctcccataaatgctg	vvi-miR535a|ctrl_mismatch probe
vvi|miR-1717|nat|as|	tgttcctcgcatgaatgctg	vvi-miR535b|ctrl_perfect match probe
vvi|miR-1717|2mut|as|	tgttcctcccataaatgctg	vvi-miR535b|ctrl_mismatch probe
vvi|miR-1718|nat|as|	tgttcctcgcatgaatgctg	vvi-miR535c|ctrl_perfect match probe
vvi|miR-1718|2mut|as|	tgttcctcccataaatgctg	vvi-miR535c|ctrl_mismatch probe
vvi|miR-1719|nat|as|	tgttcctcgcatgaatgcag	vvi-miR535d|ctrl_perfect match probe
vvi|miR-1719|2mut|as|	tgttcctcccataaatgcag	vvi-miR535d|ctrl_mismatch probe
vvi|miR-1720|nat|as|	tgttcctcgcatgaatgcag	vvi-miR535e|ctrl_perfect match probe
vvi|miR-1720|2mut|as|	tgttcctcccataaatgcag	vvi-miR535e|ctrl_mismatch probe
vvi|miR-1721|nat|as|	tcattgcttcacaagctgtt	vvi-miR828a|ctrl_perfect match probe
vvi|miR-1721|2mut|as|	tcattccttcacaagttgtt	vvi-miR828a|ctrl_mismatch probe
vvi|miR-1722|nat|as|	ggaaatgactatccaggttg	vvi-miR828b|ctrl_perfect match probe
vvi|miR-1722|2mut|as|	ggaaatgcctatcaaggttg	vvi-miR828b|ctrl_mismatch probe
vvi|miR-1723|nat|as|	ctcttgcttaagacttaatc	vvi-miR845a|ctrl_perfect match probe
vvi|miR-1723|2mut|as|	ctcttccttaagccttaatc	vvi-miR845a|ctrl_mismatch probe
vvi|miR-1724|nat|as|	ctcttgcttaagacttaatc	vvi-miR845b|ctrl_perfect match probe
vvi|miR-1724|2mut|as|	ctcttccttaagccttaatc	vvi-miR845b|ctrl_mismatch probe
vvi|miR-1725|nat|as|	gtttaatcattaagaaattg	vvi-miR845c|ctrl_perfect match

		probe
vvi|miR-1725|2mut|as|	gtttaataattaacaaattg	vvi-miR845c|ctrl_mismatch probe
		vvi-miR845d|ctrl_perfect match
vvi|miR-1726|nat|as|	gttagtaattgtgctagaac	probe
vvi|miR-1726|2mut|as|	gttactaattgtcctagaac	vvi-miR845d|ctrl_mismatch probe
		vvi-miR845e|ctrl_perfect match
vvi|miR-1727|nat|as|	gttagtaatcgtgctagaac	probe
vvi|miR-1727|2mut|as|	gttagtaatagtcctagaac	vvi-miR845e|ctrl_mismatch probe
		vvi-miR156a|compl_perfect match
vvi|miR-1728|nat|as|	tgacagaagagagggagcac	probe
vvi|miR-1728|2mut|as|	tgaccgaagagagtgagcac	vvi-miR156a|compl_mismatch probe
		vvi-miR156b|compl_perfect match
vvi|miR-1729|nat|as|	tgacagaagagagtgagcac	probe
vvi|miR-1729|2mut|as|	tgaccgaagagagcgagcac	vvi-miR156b|compl_mismatch probe
		vvi-miR156e|compl_perfect match
vvi|miR-1730|nat|as|	tgacagaggagagtgagcac	probe
vvi|miR-1730|2mut|as|	tgacagagaagagcgagcac	vvi-miR156e|compl_mismatch probe
		vvi-miR156f|compl_perfect match
vvi|miR-1731|nat|as|	ttgacagaagatagagagcac	probe
vvi|miR-1731|2mut|as|	ttgaaagaagatagagcgcac	vvi-miR156f|compl_mismatch probe
		vvi-miR156h|compl_perfect match
vvi|miR-1732|nat|as|	tgacagaagagagagagcat	probe
vvi|miR-1732|2mut|as|	tgaccgaagagagagcgcat	vvi-miR156h|compl_mismatch probe
		vvi-miR156i|compl_perfect match
vvi|miR-1733|nat|as|	tgacagaagatagagagcac	probe
vvi|miR-1733|2mut|as|	tgaccgaagatagagcgcac	vvi-miR156i|compl_mismatch probe
		vvi-miR159a|compl_perfect match
vvi|miR-1734|nat|as|	cttggagtgaagggagctctc	probe
vvi|miR-1734|2mut|as|	cttgaagtgaagtgagctctc	vvi-miR159a|compl_mismatch probe
		vvi-miR159b|compl_perfect match
vvi|miR-1735|nat|as|	cttggagtgaagggagctctca	probe
vvi|miR-1735|2mut|as|	cttgaagtgaagtgagctctca	vvi-miR159b|compl_mismatch probe
		vvi-miR159c|compl_perfect match
vvi|miR-1736|nat|as|	tttggattgaagggagctcta	probe

vvi|miR-1736|2mut|as|	tttgaattgaagtgagctcta	vvi-miR159c|compl_mismatch probe
vvi|miR-1737|nat|as|	tgcctggctccctgaatgcca	vvi-miR160a|compl_perfect match probe
vvi|miR-1737|2mut|as|	tgcctgcctccttgaatgcca	vvi-miR160a|compl_mismatch probe
vvi|miR-1738|nat|as|	tgcctggctccctgtatgcca	vvi-miR160c|compl_perfect match probe
vvi|miR-1738|2mut|as|	tgcctgcctccttgtatgcca	vvi-miR160c|compl_mismatch probe
vvi|miR-1739|nat|as|	tcgataaacctctgcatccag	vvi-miR162|compl_perfect match probe
vvi|miR-1739|2mut|as|	tcgataaatctctgaatccag	vvi-miR162|compl_mismatch probe
vvi|miR-1740|nat|as|	tggagaagcagggcacgtgca	vvi-miR164a|compl_perfect match probe
vvi|miR-1740|2mut|as|	tggagaagaaggccacgtgca	vvi-miR164a|compl_mismatch probe
vvi|miR-1741|nat|as|	tggagaagcagggcacatgct	vvi-miR164b|compl_perfect match probe
vvi|miR-1741|2mut|as|	tggagaagaaggccacatgct	vvi-miR164b|compl_mismatch probe
vvi|miR-1742|nat|as|	tcggaccaggcttcattccccc	vvi-miR166c|compl_perfect match probe
vvi|miR-1742|2mut|as|	tcggaccagccttcatttcccc	vvi-miR166c|compl_mismatch probe
vvi|miR-1743|nat|as|	tcggaccaggcttcattcccct	vvi-miR166d|compl_perfect match probe
vvi|miR-1743|2mut|as|	tcggaccagccttcatttccct	vvi-miR166d|compl_mismatch probe
vvi|miR-1744|nat|as|	tcggaccaggcttcattcctg	vvi-miR166a|compl_perfect match probe
vvi|miR-1744|2mut|as|	tcggaccagccttaattcctg	vvi-miR166a|compl_mismatch probe
vvi|miR-1745|nat|as|	tcggaccaggcttcattcctc	vvi-miR166b|compl_perfect match probe
vvi|miR-1745|2mut|as|	tcggaccagccttaattcctc	vvi-miR166b|compl_mismatch probe
vvi|miR-1746|nat|as|	tgaagctgccagcatgatctg	vvi-miR167a|compl_perfect match probe
vvi|miR-1746|2mut|as|	tgaagctgtcagaatgatctg	vvi-miR167a|compl_mismatch probe
vvi|miR-1747|nat|as|	tgaagctgccagcatgatcta	vvi-miR167b|compl_perfect match probe
vvi|miR-1747|2mut|as|	tgaagctgtcagaatgatcta	vvi-miR167b|compl_mismatch probe

vvi|miR-1748|nat|as|	tgaagctgccagcatgatct	vvi-miR167c|compl_perfect match probe
vvi|miR-1748|2mut|as|	tgaagctgtcagaatgatct	vvi-miR167c|compl_mismatch probe
vvi|miR-1749|nat|as|	tcgcttggtgcaggtcgggaa	vvi-miR168|compl_perfect match probe
vvi|miR-1749|2mut|as|	tcgcttggtgaaggtcaggaa	vvi-miR168|compl_mismatch probe
vvi|miR-1750|nat|as|	cagccaaggatgacttgccgg	vvi-miR169a|compl_perfect match probe
vvi|miR-1750|2mut|as|	cagcaaaggatgacttcccgg	vvi-miR169a|compl_mismatch probe
vvi|miR-1751|nat|as|	tagcgaaggatgacttgccta	vvi-miR169y|compl_perfect match probe
vvi|miR-1751|2mut|as|	tagcaaaggatgacttcccta	vvi-miR169y|compl_mismatch probe
vvi|miR-1752|nat|as|	tgagccaaggatggcttgccg	vvi-miR169b|compl_perfect match probe
vvi|miR-1752|2mut|as|	tgagtcaaggatgccttgccg	vvi-miR169b|compl_mismatch probe
vvi|miR-1753|nat|as|	cagccaagaatgatttgccgg	vvi-miR169d|compl_perfect match probe
vvi|miR-1753|2mut|as|	cagcaaagaatgatttcccgg	vvi-miR169d|compl_mismatch probe
vvi|miR-1754|nat|as|	tagccaaggatgacttgcctg	vvi-miR169e|compl_perfect match probe
vvi|miR-1754|2mut|as|	tagcaaaggatgacttccctg	vvi-miR169e|compl_mismatch probe
vvi|miR-1755|nat|as|	cagccaaggatgacttgccga	vvi-miR169f|compl_perfect match probe
vvi|miR-1755|2mut|as|	cagcaaaggatgacttcccga	vvi-miR169f|compl_mismatch probe
vvi|miR-1756|nat|as|	cagccaaggatgacttgcc	vvi-miR169g|compl_perfect match probe
vvi|miR-1756|2mut|as|	cagcaaaggatgccttgcc	vvi-miR169g|compl_mismatch probe
vvi|miR-1757|nat|as|	gagccaaggatgactggccgt	vvi-miR169i|compl_perfect match probe
vvi|miR-1757|2mut|as|	gagcaaaggatgactgcccgt	vvi-miR169i|compl_mismatch probe
vvi|miR-1758|nat|as|	gagccaaggatgacttgccgt	vvi-miR169l|compl_perfect match probe
vvi|miR-1758|2mut|as|	gagcaaaggatgacttcccgt	vvi-miR169l|compl_mismatch probe
vvi|miR-1759|nat|as|	gagccaaggatgacttgccgg	vvi-miR169m|compl_perfect match

		probe
vvi|miR-1759|2mut|as|	gagcaaaggatgacttcccgg	vvi-miR169m|compl_mismatch probe
vvi|miR-1760|nat|as|	gagccaaggatgacttgccgc	vvi-miR169o|compl_perfect match probe
vvi|miR-1760|2mut|as|	gagcaaaggatgacttcccgc	vvi-miR169o|compl_mismatch probe
vvi|miR-1761|nat|as|	tgagtcaaggatgacttgccg	vvi-miR169r|compl_perfect match probe
vvi|miR-1761|2mut|as|	tgagtcaagaatgccttgccg	vvi-miR169r|compl_mismatch probe
vvi|miR-1762|nat|as|	cgagtcaaggatgacttgccg	vvi-miR169t|compl_perfect match probe
vvi|miR-1762|2mut|as|	cgagtcaagaatgccttgccg	vvi-miR169t|compl_mismatch probe
vvi|miR-1763|nat|as|	aagccaaggatgaattgccgg	vvi-miR169v|compl_perfect match probe
vvi|miR-1763|2mut|as|	aagcaaaggatgaattcccgg	vvi-miR169v|compl_mismatch probe
vvi|miR-1764|nat|as|	tagccaaggatgacttgccta	vvi-miR169x|compl_perfect match probe
vvi|miR-1764|2mut|as|	tagcaaaggatgacttcccta	vvi-miR169x|compl_mismatch probe
vvi|miR-1765|nat|as|	ttgagccgtcccaacatcacg	vvi-miR171a|compl_perfect match probe
vvi|miR-1765|2mut|as|	ttgagtcgtccaaacatcacg	vvi-miR171a|compl_mismatch probe
vvi|miR-1766|nat|as|	tgattgagccgcgtcaatatc	vvi-miR171b|compl_perfect match probe
vvi|miR-1766|2mut|as|	tgattgagcccagtcaatatc	vvi-miR171b|compl_mismatch probe
vvi|miR-1767|nat|as|	ttgagccgtgccaatatcacg	vvi-miR171c|compl_perfect match probe
vvi|miR-1767|2mut|as|	ttgagccgtgtaaatatcacg	vvi-miR171c|compl_mismatch probe
vvi|miR-1768|nat|as|	tgattgagccgcgccaatatc	vvi-miR171e|compl_perfect match probe
vvi|miR-1768|2mut|as|	tgattgagcccccccaatatc	vvi-miR171e|compl_mismatch probe
vvi|miR-1769|nat|as|	ttgagccgcgccaatatcact	vvi-miR171f|compl_perfect match probe
vvi|miR-1769|2mut|as|	ttgagccgcccaaatatcact	vvi-miR171f|compl_mismatch probe
vvi|miR-1770|nat|as|	ttgagccgaaccaatatcacc	vvi-miR171g|compl_perfect match probe

vvi|miR-1770|2mut|as|	ttgagtcgaacaaatatcacc	vvi-miR171g|compl_mismatch probe
vvi|miR-1771|nat|as|	tggttgagccgcgccaatatc	vvi-miR171h|compl_perfect match probe
vvi|miR-1771|2mut|as|	tggttgagcccccccaatatc	vvi-miR171h|compl_mismatch probe
vvi|miR-1772|nat|as|	ttgagccgtgccaatatcatc	vvi-miR171i|compl_perfect match probe
vvi|miR-1772|2mut|as|	ttgagccgtgtaaatatcatc	vvi-miR171i|compl_mismatch probe
vvi|miR-1773|nat|as|	tgaatcttgatgatgctacat	vvi-miR172a|compl_perfect match probe
vvi|miR-1773|2mut|as|	tgaatcttaatgatcctacat	vvi-miR172a|compl_mismatch probe
vvi|miR-1774|nat|as|	tgaatcttgatgatgctacac	vvi-miR172b|compl_perfect match probe
vvi|miR-1774|2mut|as|	tgaatcttaatgatcctacac	vvi-miR172b|compl_mismatch probe
vvi|miR-1775|nat|as|	ggaatcttgatgatgctgcag	vvi-miR172c|compl_perfect match probe
vvi|miR-1775|2mut|as|	ggaatcttaatgatcctgcag	vvi-miR172c|compl_mismatch probe
vvi|miR-1776|nat|as|	agaatcttgatgatgctgcat	vvi-miR172d|compl_perfect match probe
vvi|miR-1776|2mut|as|	agaatcttaatgatcctgcat	vvi-miR172d|compl_mismatch probe
vvi|miR-1777|nat|as|	cttggactgaagggagctccc	vvi-miR319b|compl_perfect match probe
vvi|miR-1777|2mut|as|	cttgaactgaagtgagctccc	vvi-miR319b|compl_mismatch probe
vvi|miR-1778|nat|as|	tttggactgaagggagctcct	vvi-miR319e|compl_perfect match probe
vvi|miR-1778|2mut|as|	tttgaactgaagtgagctcct	vvi-miR319e|compl_mismatch probe
vvi|miR-1779|nat|as|	attggactgaagggagctccc	vvi-miR319g|compl_perfect match probe
vvi|miR-1779|2mut|as|	attgaactgaagtgagctccc	vvi-miR319g|compl_mismatch probe
vvi|miR-1780|nat|as|	aagctcaggagggatagcgcc	vvi-miR390|compl_perfect match probe
vvi|miR-1780|2mut|as|	aagctcagaagtgatagcgcc	vvi-miR390|compl_mismatch probe
vvi|miR-1781|nat|as|	tccaaagggatcgcattgatcc	vvi-miR393a|compl_perfect match probe
vvi|miR-1781|2mut|as|	tccaaagtgatcccattgatcc	vvi-miR393a|compl_mismatch probe

vvi|miR-1782|nat|as|	ttggcattctgtccacctcc	vvi-miR394a|compl_perfect match probe
vvi|miR-1782|2mut|as|	ttggaattctgtcaacctcc	vvi-miR394a|compl_mismatch probe
vvi|miR-1783|nat|as|	ctgaagtgtttgggggaactc	vvi-miR395a|compl_perfect match probe
vvi|miR-1783|2mut|as|	ctgaagcgtttgggtgaactc	vvi-miR395a|compl_mismatch probe
vvi|miR-1784|nat|as|	ctgaagagtctggaggaactc	vvi-miR395n|compl_perfect match probe
vvi|miR-1784|2mut|as|	ctgaagagcctcgaggaactc	vvi-miR395n|compl_mismatch probe
vvi|miR-1785|nat|as|	ctcccacagctttcttgagctt	vvi-miR396a|compl_perfect match probe
vvi|miR-1785|2mut|as|	ctcccacagttttcttaagctt	vvi-miR396a|compl_mismatch probe
vvi|miR-1786|nat|as|	ttcccacagctttcttgaactt	vvi-miR396b|compl_perfect match probe
vvi|miR-1786|2mut|as|	ttcccacagttttcttaaactt	vvi-miR396b|compl_mismatch probe
vvi|miR-1787|nat|as|	ttccacagctttcttgaactg	vvi-miR396c|compl_perfect match probe
vvi|miR-1787|2mut|as|	ttccacagttttcttaaactg	vvi-miR396c|compl_mismatch probe
vvi|miR-1788|nat|as|	tcattgagtgcagcgttgatg	vvi-miR397a|compl_perfect match probe
vvi|miR-1788|2mut|as|	tcattgagtgaagagttgatg	vvi-miR397a|compl_mismatch probe
vvi|miR-1789|nat|as|	tgtgttctcaggtcacccctt	vvi-miR398a|compl_perfect match probe
vvi|miR-1789|2mut|as|	tgtgttctcacgtcactcctt	vvi-miR398a|compl_mismatch probe
vvi|miR-1790|nat|as|	tgtgttctcaggtcgcccctg	vvi-miR398b|compl_perfect match probe
vvi|miR-1790|2mut|as|	tgtgttctcacgtccccctg	vvi-miR398b|compl_mismatch probe
vvi|miR-1791|nat|as|	tgccaaaggagaattgccctg	vvi-miR399a|compl_perfect match probe
vvi|miR-1791|2mut|as|	tgccaaagaagaattgtcctg	vvi-miR399a|compl_mismatch probe
vvi|miR-1792|nat|as|	tgccaaaggagagttgccctg	vvi-miR399b|compl_perfect match probe
vvi|miR-1792|2mut|as|	tgccaaagaagagttgtcctg	vvi-miR399b|compl_mismatch probe
vvi|miR-1793|nat|as|	tgccaaaggagatttgctcgt	vvi-miR399d|compl_perfect match

		probe
vvi|miR-1793|2mut|as|	tgccaaagaagatttcctcgt	vvi-miR399d|compl_mismatch probe
vvi|miR-1794|nat|as|	tgccaaaggagatttgcccgg	vvi-miR399e|compl_perfect match probe
vvi|miR-1794|2mut|as|	tgccaaagaagatttgtccgg	vvi-miR399e|compl_mismatch probe
vvi|miR-1795|nat|as|	tgccgaaggagatttgtcctg	vvi-miR399f|compl_perfect match probe
vvi|miR-1795|2mut|as|	tgccaaaggagatttctcctg	vvi-miR399f|compl_mismatch probe
vvi|miR-1796|nat|as|	tgccaaaggagatttgcccct	vvi-miR399g|compl_perfect match probe
vvi|miR-1796|2mut|as|	tgccaaagaagatttgtccct	vvi-miR399g|compl_mismatch probe
vvi|miR-1797|nat|as|	cgccaaaggagagttgccctg	vvi-miR399i|compl_perfect match probe
vvi|miR-1797|2mut|as|	cgccaaagaagagttgtcctg	vvi-miR399i|compl_mismatch probe
vvi|miR-1798|nat|as|	ttagattcacgcacaaactcg	vvi-miR403a|compl_perfect match probe
vvi|miR-1798|2mut|as|	ttagattcaccaacaaactcg	vvi-miR403a|compl_mismatch probe
vvi|miR-1799|nat|as|	atgcactgcctcttccctggc	vvi-miR408|compl_perfect match probe
vvi|miR-1799|2mut|as|	atgcactgtctcttctctggc	vvi-miR408|compl_mismatch probe
vvi|miR-1800|nat|as|	atctccctcaaaggcttccaa	vvi-miR477a|compl_perfect match probe
vvi|miR-1800|2mut|as|	atctctctcaaagccttccaa	vvi-miR477a|compl_mismatch probe
vvi|miR-1801|nat|as|	tgtggtattggttcggctcatc	vvi-miR479|compl_perfect match probe
vvi|miR-1801|2mut|as|	tgtggtattcgttcgcctcatc	vvi-miR479|compl_mismatch probe
vvi|miR-1802|nat|as|	cctactcctcccattcc	vvi-miR482a|compl_perfect match probe
vvi|miR-1802|2mut|as|	cctacttctctcattcc	vvi-miR482a|compl_mismatch probe
vvi|miR-1803|nat|as|	tgacaacgagagagagcacgct	vvi-miR535a|compl_perfect match probe
vvi|miR-1803|2mut|as|	tgacaaagagagagagaacgct	vvi-miR535a|compl_mismatch probe
vvi|miR-1804|nat|as|	tcttgctcaaatgagtattcca	vvi-miR828a|compl_perfect match probe

vvi|miR-1804|2mut|as|	tcttcctcaaataagtattcca	vvi-miR828a|compl_mismatch probe
		vvi-miR828b|compl_perfect match
vvi|miR-1805|nat|as|	tcttgctcaaatgagtgttcca	probe
vvi|miR-1805|2mut|as|	tcttcctcaaatgagcgttcca	vvi-miR828b|compl_mismatch probe
		vvi-miR845a|compl_perfect match
vvi|miR-1806|nat|as|	tagctctgataccaattgata	probe
vvi|miR-1806|2mut|as|	tagctccgatacaaattgata	vvi-miR845a|compl_mismatch probe
		vvi-miR845c|compl_perfect match
vvi|miR-1807|nat|as|	aggctctgataccaattgatg	probe
vvi|miR-1807|2mut|as|	aggctccgatacaaattgatg	vvi-miR845c|compl_mismatch probe
		vvi-miR845d|compl_perfect match
vvi|miR-1808|nat|as|	tggctctgataccaattgatg	probe
vvi|miR-1808|2mut|as|	tggctccgatacaaattgatg	vvi-miR845d|compl_mismatch probe
		vvi-miR156a|*compl_perfect match
vvi|miR-1809|nat|as|	gtgctctctcttcttctgtca	probe
		vvi-miR156a|*compl_mismatch
vvi|miR-1809|2mut|as|	gtgctctcccttcttccgtca	probe
		vvi-miR156b|*compl_perfect match
vvi|miR-1810|nat|as|	gtgctcatttctctttctgtca	probe
		vvi-miR156b|*compl_mismatch
vvi|miR-1810|2mut|as|	gtgctaatttctctttccgtca	probe
		vvi-miR156c|*compl_perfect match
vvi|miR-1811|nat|as|	gtgctcactctctatctgtca	probe
		vvi-miR156c|*compl_mismatch
vvi|miR-1811|2mut|as|	gtgctccctctctatccgtca	probe
		vvi-miR156d|*compl_perfect match
vvi|miR-1812|nat|as|	gtgctcacctctctttctgtca	probe
		vvi-miR156d|*compl_mismatch
vvi|miR-1812|2mut|as|	gtgctcatctctctttccgtca	probe
		vvi-miR156e|*compl_perfect match
vvi|miR-1813|nat|as|	gtgcttactccctatctgtca	probe
		vvi-miR156e|*compl_mismatch
vvi|miR-1813|2mut|as|	gtgcttactctttatctgtca	probe
vvi|miR-1814|nat|as|	gtcctctctcttctcctgtcaa	vvi-miR156f|*compl_perfect match

<table>
<tr><td></td><td></td><td>probe</td></tr>
<tr><td>vvi|miR-1814|2mut|as|</td><td>gtcctctcccttcttctgtcaa</td><td>vvi-miR156f|*compl_mismatch probe</td></tr>
<tr><td>vvi|miR-1815|nat|as|</td><td>gtgctctctagacttctgtca</td><td>vvi-miR156g|*compl_perfect match probe</td></tr>
<tr><td>vvi|miR-1815|2mut|as|</td><td>gtgctccctagccttctgtca</td><td>vvi-miR156g|*compl_mismatch probe</td></tr>
<tr><td>vvi|miR-1816|nat|as|</td><td>gttctttctgcgcctttca</td><td>vvi-miR156h|*compl_perfect match probe</td></tr>
<tr><td>vvi|miR-1816|2mut|as|</td><td>gttctttctcccccctttca</td><td>vvi-miR156h|*compl_mismatch probe</td></tr>
<tr><td>vvi|miR-1817|nat|as|</td><td>gtgctctctattcttctgtca</td><td>vvi-miR156i|*compl_perfect match probe</td></tr>
<tr><td>vvi|miR-1817|2mut|as|</td><td>gtgctccctattcttccgtca</td><td>vvi-miR156i|*compl_mismatch probe</td></tr>
<tr><td>vvi|miR-1818|nat|as|</td><td>gggagctcctttacgctccag</td><td>vvi-miR159a|*compl_perfect match probe</td></tr>
<tr><td>vvi|miR-1818|2mut|as|</td><td>gggagttcctttaccctccag</td><td>vvi-miR159a|*compl_mismatch probe</td></tr>
<tr><td>vvi|miR-1819|nat|as|</td><td>tgggagcttctttacactccag</td><td>vvi-miR159b|*compl_perfect match probe</td></tr>
<tr><td>vvi|miR-1819|2mut|as|</td><td>tgggagtttctttaccctccag</td><td>vvi-miR159b|*compl_mismatch probe</td></tr>
<tr><td>vvi|miR-1820|nat|as|</td><td>tggagctccttgaagtccaa</td><td>vvi-miR159c|*compl_perfect match probe</td></tr>
<tr><td>vvi|miR-1820|2mut|as|</td><td>tggagttccttgaagcccaa</td><td>vvi-miR159c|*compl_mismatch probe</td></tr>
<tr><td>vvi|miR-1821|nat|as|</td><td>tggcatcagaggagtcatgca</td><td>vvi-miR160a|*compl_perfect match probe</td></tr>
<tr><td>vvi|miR-1821|2mut|as|</td><td>tggcatcagacaagtcatgca</td><td>vvi-miR160a|*compl_mismatch probe</td></tr>
<tr><td>vvi|miR-1822|nat|as|</td><td>tggcatgaggggagtcaagca</td><td>vvi-miR160b|*compl_perfect match probe</td></tr>
<tr><td>vvi|miR-1822|2mut|as|</td><td>tggcatgaggtaagtcaagca</td><td>vvi-miR160b|*compl_mismatch probe</td></tr>
<tr><td>vvi|miR-1823|nat|as|</td><td>tggcgtgcgaggagccaagca</td><td>vvi-miR160c|*compl_perfect match</td></tr>
</table>

		probe
		vvi-miR160c|*compl_mismatch
vvi|miR-1823|2mut|as|	tggcgtgagaggagtcaagca	probe
		vvi-miR160d|*compl_perfect match
vvi|miR-1824|nat|as|	tggcgtgcgaggagccatgca	probe
		vvi-miR160d|*compl_mismatch
vvi|miR-1824|2mut|as|	tggcgtgagaggagtcatgca	probe
		vvi-miR160e|*compl_perfect match
vvi|miR-1825|nat|as|	tggcatgaggggagtcaagca	probe
		vvi-miR160e|*compl_mismatch
vvi|miR-1825|2mut|as|	tggcatgaggtaagtcaagca	probe
		vvi-miR160f|*compl_perfect match
vvi|miR-1826|nat|as|	tggcgtatgaggagccatgca	probe
vvi|miR-1826|2mut|as|	tggcctatgaggagtcatgca	vvi-miR160f|*compl_mismatch probe
		vvi-miR162|*compl_perfect match
vvi|miR-1827|nat|as|	ctggatgcagcggttcatcga	probe
vvi|miR-1827|2mut|as|	ctggatgcagaagttcatcga	vvi-miR162|*compl_mismatch probe
		vvi-miR164a|*compl_perfect match
vvi|miR-1828|nat|as|	gcacgtgctccccttctcca	probe
		vvi-miR164a|*compl_mismatch
vvi|miR-1828|2mut|as|	gcacgtcctcctcttctcca	probe
		vvi-miR164b|*compl_perfect match
vvi|miR-1829|nat|as|	catgtgccctggctctcc	probe
		vvi-miR164b|*compl_mismatch
vvi|miR-1829|2mut|as|	catgtgtcctgcctctcc	probe
		vvi-miR164d|*compl_perfect match
vvi|miR-1830|nat|as|	tgcacgtgctccccttctcca	probe
		vvi-miR164d|*compl_mismatch
vvi|miR-1830|2mut|as|	tgcacgtcctcctcttctcca	probe
		vvi-miR166c|*compl_perfect match
vvi|miR-1831|nat|as|	ggggaatgttgtctggctcga	probe
		vvi-miR166c|*compl_mismatch
vvi|miR-1831|2mut|as|	ggggaatgttctctgcctcga	probe
vvi|miR-1832|nat|as|	aggggattgttgtctggctcga	vvi-miR166d|*compl_perfect match

		probe
vvi|miR-1832|2mut|as|	agggaattgttgtctgcctcga	vvi-miR166d|*compl_mismatch probe
vvi|miR-1833|nat|as|	ggggaatgttgtctggctcga	vvi-miR166e|*compl_perfect match probe
vvi|miR-1833|2mut|as|	ggggaatgttctctgcctcga	vvi-miR166e|*compl_mismatch probe
vvi|miR-1834|nat|as|	ggggaatgttggctggctcga	vvi-miR166f|*compl_perfect match probe
vvi|miR-1834|2mut|as|	ggggaatgttcgctgcctcga	vvi-miR166f|*compl_mismatch probe
vvi|miR-1835|nat|as|	aggggaatgttgtctggttcga	vvi-miR166g|*compl_perfect match probe
vvi|miR-1835|2mut|as|	agggaaatgttgtctcgttcga	vvi-miR166g|*compl_mismatch probe
vvi|miR-1836|nat|as|	ggggaacgctgtctggttcga	vvi-miR166h|*compl_perfect match probe
vvi|miR-1836|2mut|as|	ggggaaccctgtctcgttcga	vvi-miR166h|*compl_mismatch probe
vvi|miR-1837|nat|as|	ttggaatgaggtttgatccaa	vvi-miR166a|*compl_perfect match probe
vvi|miR-1837|2mut|as|	ttggaatgagctttaatccaa	vvi-miR166a|*compl_mismatch probe
vvi|miR-1838|nat|as|	ggggaatgttggctggctcga	vvi-miR166b|*compl_perfect match probe
vvi|miR-1838|2mut|as|	ggggaatgttcgctgcctcga	vvi-miR166b|*compl_mismatch probe
vvi|miR-1839|nat|as|	caggtcatcttgcagcttca	vvi-miR167a|*compl_perfect match probe
vvi|miR-1839|2mut|as|	caggccatcttgaagcttca	vvi-miR167a|*compl_mismatch probe
vvi|miR-1840|nat|as|	tagatcatgtggcagtttca	vvi-miR167b|*compl_perfect match probe
vvi|miR-1840|2mut|as|	tagatcatgcgccagtttca	vvi-miR167b|*compl_mismatch probe

vvi|miR-1841|nat|as|	agatcatgtggtagcctca	vvi-miR167c|*compl_perfect match probe
vvi|miR-1841|2mut|as|	agatcatgtcgtagtctca	vvi-miR167c|*compl_mismatch probe
vvi|miR-1842|nat|as|	taggtcatgccctgacagcctca	vvi-miR167d|*compl_perfect match probe
vvi|miR-1842|2mut|as|	taggtcatgtcctgacagtctca	vvi-miR167d|*compl_mismatch probe
vvi|miR-1843|nat|as|	agatcatgtggcagtttca	vvi-miR167e|*compl_perfect match probe
vvi|miR-1843|2mut|as|	agatcatgtcccagtttca	vvi-miR167e|*compl_mismatch probe
vvi|miR-1844|nat|as|	tcccgccttgcatcaactga	vvi-miR168|*compl_perfect match probe
vvi|miR-1844|2mut|as|	tcccccccttgaatcaactga	vvi-miR168|*compl_mismatch probe
vvi|miR-1845|nat|as|	ctggcaagttgtccttggct	vvi-miR169a|*compl_perfect match probe
vvi|miR-1845|2mut|as|	ctggaaagttgttcttggct	vvi-miR169a|*compl_mismatch probe
vvi|miR-1846|nat|as|	ggcaagtctccttggca	vvi-miR169y|*compl_perfect match probe
vvi|miR-1846|2mut|as|	ggcaagccttcttggca	vvi-miR169y|*compl_mismatch probe
vvi|miR-1847|nat|as|	cggttagtcatccttggctc	vvi-miR169b|*compl_perfect match probe
vvi|miR-1847|2mut|as|	cggttagccatccttcgctc	vvi-miR169b|*compl_mismatch probe
vvi|miR-1848|nat|as|	ccggcaagttgtccttggct	vvi-miR169c|*compl_perfect match probe
vvi|miR-1848|2mut|as|	ccggaaagttgttcttggct	vvi-miR169c|*compl_mismatch probe
vvi|miR-1849|nat|as|	ctggcaagttgtccttggct	vvi-miR169d|*compl_perfect match probe
vvi|miR-1849|2mut|as|	ctggaaagttgttcttggct	vvi-miR169d|*compl_mismatch

<table>
<tr><td></td><td></td><td>probe</td></tr>
<tr><td></td><td></td><td>vvi-miR169e|*compl_perfect match</td></tr>
<tr><td>vvi|miR-1850|nat|as|</td><td>caggcagtcaccttggctaa</td><td>probe</td></tr>
<tr><td></td><td></td><td>vvi-miR169e|*compl_mismatch</td></tr>
<tr><td>vvi|miR-1850|2mut|as|</td><td>caggaagtcaccttgcctaa</td><td>probe</td></tr>
<tr><td></td><td></td><td>vvi-miR169f|*compl_perfect match</td></tr>
<tr><td>vvi|miR-1851|nat|as|</td><td>ggcaagttgtgtttggct</td><td>probe</td></tr>
<tr><td>vvi|miR-1851|2mut|as|</td><td>ggcaagttccgtttggct</td><td>vvi-miR169f|*compl_mismatch probe</td></tr>
<tr><td></td><td></td><td>vvi-miR169g|*compl_perfect match</td></tr>
<tr><td>vvi|miR-1852|nat|as|</td><td>ggcaagttgtctttggct</td><td>probe</td></tr>
<tr><td></td><td></td><td>vvi-miR169g|*compl_mismatch</td></tr>
<tr><td>vvi|miR-1852|2mut|as|</td><td>ggcaagttccctttggct</td><td>probe</td></tr>
<tr><td></td><td></td><td>vvi-miR169h|*compl_perfect match</td></tr>
<tr><td>vvi|miR-1853|nat|as|</td><td>cggagggttatccttgactc</td><td>probe</td></tr>
<tr><td></td><td></td><td>vvi-miR169h|*compl_mismatch</td></tr>
<tr><td>vvi|miR-1853|2mut|as|</td><td>cggagtgttattcttgactc</td><td>probe</td></tr>
<tr><td></td><td></td><td>vvi-miR169i|*compl_perfect match</td></tr>
<tr><td>vvi|miR-1854|nat|as|</td><td>acggctggttatccctgtctc</td><td>probe</td></tr>
<tr><td>vvi|miR-1854|2mut|as|</td><td>acggttggttatctctgtctc</td><td>vvi-miR169i|*compl_mismatch probe</td></tr>
<tr><td></td><td></td><td>vvi-miR169j|*compl_perfect match</td></tr>
<tr><td>vvi|miR-1855|nat|as|</td><td>ctggcaggttgtccttggct</td><td>probe</td></tr>
<tr><td>vvi|miR-1855|2mut|as|</td><td>ctggaaggttgttcttggct</td><td>vvi-miR169j|*compl_mismatch probe</td></tr>
<tr><td></td><td></td><td>vvi-miR169k|*compl_perfect match</td></tr>
<tr><td>vvi|miR-1856|nat|as|</td><td>ctggcaggttgtccttggct</td><td>probe</td></tr>
<tr><td></td><td></td><td>vvi-miR169k|*compl_mismatch</td></tr>
<tr><td>vvi|miR-1856|2mut|as|</td><td>ctggaaggttgttcttggct</td><td>probe</td></tr>
<tr><td></td><td></td><td>vvi-miR169l|*compl_perfect match</td></tr>
<tr><td>vvi|miR-1857|nat|as|</td><td>acggctagttattcttggctc</td><td>probe</td></tr>
<tr><td>vvi|miR-1857|2mut|as|</td><td>acggttagttattcttcgctc</td><td>vvi-miR169l|*compl_mismatch probe</td></tr>
<tr><td></td><td></td><td>vvi-miR169m|*compl_perfect match</td></tr>
<tr><td>vvi|miR-1858|nat|as|</td><td>ctggcaagcatccgaggctc</td><td>probe</td></tr>
<tr><td></td><td></td><td>vvi-miR169m|*compl_mismatch</td></tr>
<tr><td>vvi|miR-1858|2mut|as|</td><td>ctggcaagaatcagaggctc</td><td>probe</td></tr>
<tr><td>vvi|miR-1859|nat|as|</td><td>cataactggcaagcatctgaggctc</td><td>vvi-miR169n|*compl_perfect match</td></tr>
</table>

<table>
<tr><td></td><td></td><td>probe</td></tr>
<tr><td>vvi|miR-1859|2mut|as|</td><td>cataactgccaagaatctgaggctc</td><td>vvi-miR169n|*compl_mismatch probe</td></tr>
<tr><td>vvi|miR-1860|nat|as|</td><td>gacagcgagttattcttggctc</td><td>vvi-miR169o|*compl_perfect match probe</td></tr>
<tr><td>vvi|miR-1860|2mut|as|</td><td>gacagagagttattcttcgctc</td><td>vvi-miR169o|*compl_mismatch probe</td></tr>
<tr><td>vvi|miR-1861|nat|as|</td><td>ctggcaagcatccgaggctc</td><td>vvi-miR169p|*compl_perfect match probe</td></tr>
<tr><td>vvi|miR-1861|2mut|as|</td><td>ctggcaagaatcagaggctc</td><td>vvi-miR169p|*compl_mismatch probe</td></tr>
<tr><td>vvi|miR-1862|nat|as|</td><td>ccggcatgtaatcctggctc</td><td>vvi-miR169q|*compl_perfect match probe</td></tr>
<tr><td>vvi|miR-1862|2mut|as|</td><td>ccggaatgtaatcctcgctc</td><td>vvi-miR169q|*compl_mismatch probe</td></tr>
<tr><td>vvi|miR-1863|nat|as|</td><td>cggcaagttgacttgactca</td><td>vvi-miR169r|*compl_perfect match probe</td></tr>
<tr><td>vvi|miR-1863|2mut|as|</td><td>cggcaagttaacttgcctca</td><td>vvi-miR169r|*compl_mismatch probe</td></tr>
<tr><td>vvi|miR-1864|nat|as|</td><td>ccggcaagttgttcctggct</td><td>vvi-miR169s|*compl_perfect match probe</td></tr>
<tr><td>vvi|miR-1864|2mut|as|</td><td>ccggaaagttgtttctggct</td><td>vvi-miR169s|*compl_mismatch probe</td></tr>
<tr><td>vvi|miR-1865|nat|as|</td><td>cggcaagttgacttgactc</td><td>vvi-miR169t|*compl_perfect match probe</td></tr>
<tr><td>vvi|miR-1865|2mut|as|</td><td>cggcaagttaccttgactc</td><td>vvi-miR169t|*compl_mismatch probe</td></tr>
<tr><td>vvi|miR-1866|nat|as|</td><td>cggcaagttgacttgactc</td><td>vvi-miR169u|*compl_perfect match probe</td></tr>
<tr><td>vvi|miR-1866|2mut|as|</td><td>cggcaagttaccttgactc</td><td>vvi-miR169u|*compl_mismatch probe</td></tr>
<tr><td>vvi|miR-1867|nat|as|</td><td>tcggcaatttatttcttggct</td><td>vvi-miR169v|*compl_perfect match probe</td></tr>
<tr><td>vvi|miR-1867|2mut|as|</td><td>tcggaaatttattttttggct</td><td>vvi-miR169v|*compl_mismatch probe</td></tr>
<tr><td>vvi|miR-1868|nat|as|</td><td>tcggcgagtcattcttagct</td><td>vvi-miR169w|*compl_perfect match</td></tr>
</table>

<table>
<tr><td></td><td></td><td>probe</td></tr>
<tr><td>vvi|miR-1868|2mut|as|</td><td>tcggagagtcatttttagct</td><td>vvi-miR169w|*compl_mismatch probe</td></tr>
<tr><td>vvi|miR-1869|nat|as|</td><td>taggcagtcattccttcggcta</td><td>vvi-miR169x|*compl_perfect match probe</td></tr>
<tr><td>vvi|miR-1869|2mut|as|</td><td>taggaagtcattccttcagcta</td><td>vvi-miR169x|*compl_mismatch probe</td></tr>
<tr><td>vvi|miR-1870|nat|as|</td><td>catgatattggcacggctcaa</td><td>vvi-miR171a|*compl_perfect match probe</td></tr>
<tr><td>vvi|miR-1870|2mut|as|</td><td>catgatattgccacgcctcaa</td><td>vvi-miR171a|*compl_mismatch probe</td></tr>
<tr><td>vvi|miR-1871|nat|as|</td><td>ggtattggcgtgcctcaatt</td><td>vvi-miR171b|*compl_perfect match probe</td></tr>
<tr><td>vvi|miR-1871|2mut|as|</td><td>ggtattggagtgtctcaatt</td><td>vvi-miR171b|*compl_mismatch probe</td></tr>
<tr><td>vvi|miR-1872|nat|as|</td><td>cgggatattggtgcggttcaa</td><td>vvi-miR171c|*compl_perfect match probe</td></tr>
<tr><td>vvi|miR-1872|2mut|as|</td><td>cgggatattcgtgaggttcaa</td><td>vvi-miR171c|*compl_mismatch probe</td></tr>
<tr><td>vvi|miR-1873|nat|as|</td><td>cgagatattgatacggttcaa</td><td>vvi-miR171d|*compl_perfect match probe</td></tr>
<tr><td>vvi|miR-1873|2mut|as|</td><td>cgagatattaatacagttcaa</td><td>vvi-miR171d|*compl_mismatch probe</td></tr>
<tr><td>vvi|miR-1874|nat|as|</td><td>gatgttggtgaggttcaatc</td><td>vvi-miR171e|*compl_perfect match probe</td></tr>
<tr><td>vvi|miR-1874|2mut|as|</td><td>gatgttcgtgagcttcaatc</td><td>vvi-miR171e|*compl_mismatch probe</td></tr>
<tr><td>vvi|miR-1875|nat|as|</td><td>agcgatgttggtgaggttcaa</td><td>vvi-miR171f|*compl_perfect match probe</td></tr>
<tr><td>vvi|miR-1875|2mut|as|</td><td>agcgatgttcgtgagcttcaa</td><td>vvi-miR171f|*compl_mismatch probe</td></tr>
<tr><td>vvi|miR-1876|nat|as|</td><td>atgttggttccatcgg</td><td>vvi-miR171g|*compl_perfect match probe</td></tr>
<tr><td>vvi|miR-1876|2mut|as|</td><td>atgttcgttcaatcgg</td><td>vvi-miR171g|*compl_mismatch probe</td></tr>
</table>

vvi|miR-1877|nat|as|	gatgttggtgcggttcaacc	vvi-miR171h|*compl_perfect match probe
vvi|miR-1877|2mut|as|	gatgttggtcaggttcaacc	vvi-miR171h|*compl_mismatch probe
vvi|miR-1878|nat|as|	ggggatgttggaatggctcaa	vvi-miR171i|*compl_perfect match probe
vvi|miR-1878|2mut|as|	ggggatgttcgaatgcctcaa	vvi-miR171i|*compl_mismatch probe
vvi|miR-1879|nat|as|	atgcagcatcatcaagattc	vvi-miR172a|*compl_perfect match probe
vvi|miR-1879|2mut|as|	atgcagaatcataaagattc	vvi-miR172a|*compl_mismatch probe
vvi|miR-1880|nat|as|	tgcagcatcatcaagattc	vvi-miR172b|*compl_perfect match probe
vvi|miR-1880|2mut|as|	tgcagaatcataaagattc	vvi-miR172b|*compl_mismatch probe
vvi|miR-1881|nat|as|	tggagcatcatcaagattc	vvi-miR172c|*compl_perfect match probe
vvi|miR-1881|2mut|as|	tggagaatcataaagattc	vvi-miR172c|*compl_mismatch probe
vvi|miR-1882|nat|as|	atgcagcatcatcaagattc	vvi-miR172d|*compl_perfect match probe
vvi|miR-1882|2mut|as|	atgcagaatcataaagattc	vvi-miR172d|*compl_mismatch probe
vvi|miR-1883|nat|as|	ggagctctctttagtccag	vvi-miR319b|*compl_perfect match probe
vvi|miR-1883|2mut|as|	ggagttctctttagcccag	vvi-miR319b|*compl_mismatch probe
vvi|miR-1884|nat|as|	gagagctttcttcagtcca	vvi-miR319c|*compl_perfect match probe
vvi|miR-1884|2mut|as|	gagagttttcttccgtcca	vvi-miR319c|*compl_mismatch probe
vvi|miR-1885|nat|as|	gggggttcctttgcagcccaaa	vvi-miR319e|*compl_perfect match probe
vvi|miR-1885|2mut|as|	ggggcttcctttgcagtccaaa	vvi-miR319e|*compl_mismatch

		probe
		vvi-miR319f|*compl_perfect match
vvi|miR-1886|nat|as|	gagagcttccttcagtcca	probe
vvi|miR-1886|2mut|as|	gagagtttccttccgtcca	vvi-miR319f|*compl_mismatch probe
		vvi-miR319g|*compl_perfect match
vvi|miR-1887|nat|as|	gagctccttttagtccaat	probe
		vvi-miR319g|*compl_mismatch
vvi|miR-1887|2mut|as|	gagcttcttttagttcaat	probe
		vvi-miR390|*compl_perfect match
vvi|miR-1888|nat|as|	ggcgctatctatcctgagttt	probe
vvi|miR-1888|2mut|as|	ggcgttatctattctgagttt	vvi-miR390|*compl_mismatch probe
		vvi-miR393a|*compl_perfect match
vvi|miR-1889|nat|as|	ggatcatgctatcccttagga	probe
		vvi-miR393a|*compl_mismatch
vvi|miR-1889|2mut|as|	ggatcatcctatctcttagga	probe
		vvi-miR393b|*compl_perfect match
vvi|miR-1890|nat|as|	ggatcatgctatccctttgga	probe
		vvi-miR393b|*compl_mismatch
vvi|miR-1890|2mut|as|	ggatcatcctatctctttgga	probe
		vvi-miR394a|*compl_perfect match
vvi|miR-1891|nat|as|	ggaggtggccagcatgccaa	probe
		vvi-miR394a|*compl_mismatch
vvi|miR-1891|2mut|as|	ggaggtggtcagcatcccaa	probe
		vvi-miR394b|*compl_perfect match
vvi|miR-1892|nat|as|	ggaggtgggcatactgccaa	probe
		vvi-miR394b|*compl_mismatch
vvi|miR-1892|2mut|as|	ggaggtggccatactcccaa	probe
		vvi-miR394c|*compl_perfect match
vvi|miR-1893|nat|as|	ggaggcggccaggatgccaa	probe
		vvi-miR394c|*compl_mismatch
vvi|miR-1893|2mut|as|	ggaggaggccaggatcccaa	probe
		vvi-miR395a|*compl_perfect match
vvi|miR-1894|nat|as|	gagttcccttgatcacttca	probe
vvi|miR-1894|2mut|as|	gagttctcttgatcccttca	vvi-miR395a|*compl_mismatch

		probe
vvi|miR-1895|nat|as|	gagttccccttaccacttca	vvi-miR395b|*compl_perfect match probe
vvi|miR-1895|2mut|as|	gagttcctcttacaacttca	vvi-miR395b|*compl_mismatch probe
vvi|miR-1896|nat|as|	gagttcccttgaccacttca	vvi-miR395c|*compl_perfect match probe
vvi|miR-1896|2mut|as|	gagttctcttgacaacttca	vvi-miR395c|*compl_mismatch probe
vvi|miR-1897|nat|as|	gagttcccctgaccacttca	vvi-miR395d|*compl_perfect match probe
vvi|miR-1897|2mut|as|	gagttcctctgacaacttca	vvi-miR395d|*compl_mismatch probe
vvi|miR-1898|nat|as|	gagttcccttgaccacttca	vvi-miR395e|*compl_perfect match probe
vvi|miR-1898|2mut|as|	gagttctcttgacaacttca	vvi-miR395e|*compl_mismatch probe
vvi|miR-1899|nat|as|	gagttcccctgaccacttca	vvi-miR395f|*compl_perfect match probe
vvi|miR-1899|2mut|as|	gagttcctctgacaacttca	vvi-miR395f|*compl_mismatch probe
vvi|miR-1900|nat|as|	gagttcccctgagcacttca	vvi-miR395g|*compl_perfect match probe
vvi|miR-1900|2mut|as|	gagttcctctgagaacttca	vvi-miR395g|*compl_mismatch probe
vvi|miR-1901|nat|as|	gagttcccttgaccacttca	vvi-miR395h|*compl_perfect match probe
vvi|miR-1901|2mut|as|	gagttctcttgacaacttca	vvi-miR395h|*compl_mismatch probe
vvi|miR-1902|nat|as|	gagttcccctgaccacttca	vvi-miR395i|*compl_perfect match probe
vvi|miR-1902|2mut|as|	gagttcctctgacaacttca	vvi-miR395i|*compl_mismatch probe
vvi|miR-1903|nat|as|	gagttcccctgaccacttca	vvi-miR395j|*compl_perfect match probe
vvi|miR-1903|2mut|as|	gagttcctctgacaacttca	vvi-miR395j|*compl_mismatch probe

vvi|miR-1904|nat|as|	gagttcccttgaccacttca	vvi-miR395k|*compl_perfect match probe
vvi|miR-1904|2mut|as|	gagttctcttgacaacttca	vvi-miR395k|*compl_mismatch probe
vvi|miR-1905|nat|as|	gagttcccctgaccacttca	vvi-miR395l|*compl_perfect match probe
vvi|miR-1905|2mut|as|	gagttcctctgacaacttca	vvi-miR395l|*compl_mismatch probe
vvi|miR-1906|nat|as|	gagttcccttgaacacttca	vvi-miR395m|*compl_perfect match probe
vvi|miR-1906|2mut|as|	gagttctcttgaacccttca	vvi-miR395m|*compl_mismatch probe
vvi|miR-1907|nat|as|	gagttcccccaacccctccag	vvi-miR395n|*compl_perfect match probe
vvi|miR-1907|2mut|as|	gagttccctcaacctctccag	vvi-miR395n|*compl_mismatch probe
vvi|miR-1908|nat|as|	agttcaagaaagctgtgg	vvi-miR396a|*compl_perfect match probe
vvi|miR-1908|2mut|as|	agttaaagaaagttgtgg	vvi-miR396a|*compl_mismatch probe
vvi|miR-1909|nat|as|	aagttcaagaaagctgtggaaa	vvi-miR396b|*compl_perfect match probe
vvi|miR-1909|2mut|as|	aagttaaagaaagttgtggaaa	vvi-miR396b|*compl_mismatch probe
vvi|miR-1910|nat|as|	cagctcaggaaagttgtggaa	vvi-miR396c|*compl_perfect match probe
vvi|miR-1910|2mut|as|	cagctcagaaaagttgcggaa	vvi-miR396c|*compl_mismatch probe
vvi|miR-1911|nat|as|	cggttcaataaagctgtggga	vvi-miR396d|*compl_perfect match probe
vvi|miR-1911|2mut|as|	cggttaaataaagttgtggga	vvi-miR396d|*compl_mismatch probe
vvi|miR-1912|nat|as|	cattggcgctgcactcaatca	vvi-miR397a|*compl_perfect match probe
vvi|miR-1912|2mut|as|	cattggccctgaactcaatca	vvi-miR397a|*compl_mismatch

		probe
vvi|miR-1913|nat|as|	cattggcgctgcactcaatca	vvi-miR397b|*compl_perfect match probe
vvi|miR-1913|2mut|as|	cattggccctgaactcaatca	vvi-miR397b|*compl_mismatch probe
vvi|miR-1914|nat|as|	agggagtggcacctgagaacac	vvi-miR398a|*compl_perfect match probe
vvi|miR-1914|2mut|as|	agggagtgccatctgagaacac	vvi-miR398a|*compl_mismatch probe
vvi|miR-1915|nat|as|	caggtgtgacctgagaatcaca	vvi-miR398b|*compl_perfect match probe
vvi|miR-1915|2mut|as|	caggtgtgatccgagaatcaca	vvi-miR398b|*compl_mismatch probe
vvi|miR-1916|nat|as|	caggagtgacctgagaatcaca	vvi-miR398c|*compl_perfect match probe
vvi|miR-1916|2mut|as|	caggagtgatccgagaatcaca	vvi-miR398c|*compl_mismatch probe
vvi|miR-1917|nat|as|	cagtgtgattctcctttggca	vvi-miR399a|*compl_perfect match probe
vvi|miR-1917|2mut|as|	cagtgcgattcttctttggca	vvi-miR399a|*compl_mismatch probe
vvi|miR-1918|nat|as|	tagggcacctctttcttggca	vvi-miR399b|*compl_perfect match probe
vvi|miR-1918|2mut|as|	taggccacctcttttttggca	vvi-miR399b|*compl_mismatch probe
vvi|miR-1919|nat|as|	cagggcctctttcacttggta	vvi-miR399c|*compl_perfect match probe
vvi|miR-1919|2mut|as|	cagggtctctttcccttggta	vvi-miR399c|*compl_mismatch probe
vvi|miR-1920|nat|as|	gagcagatttcttttggca	vvi-miR399d|*compl_perfect match probe
vvi|miR-1920|2mut|as|	gagccgatttttttggca	vvi-miR399d|*compl_mismatch probe
vvi|miR-1921|nat|as|	gggcaaattatcttttggca	vvi-miR399e|*compl_perfect match

		probe
		vvi-miR399e|*compl_mismatch
vvi|miR-1921|2mut|as|	gggccaattattttttggca	probe
		vvi-miR399f|*compl_perfect match
vvi|miR-1922|nat|as|	cattagagggccaaatctgctttggca	probe
vvi|miR-1922|2mut|as|	cattagagggtcaaatctcctttggca	vvi-miR399f|*compl_mismatch probe
		vvi-miR399g|*compl_perfect match
vvi|miR-1923|nat|as|	gggcaatactccattggca	probe
		vvi-miR399g|*compl_mismatch
vvi|miR-1923|2mut|as|	gggcaatattcaattggca	probe
		vvi-miR399h|*compl_perfect match
vvi|miR-1924|nat|as|	cagtgcaatcctcctttggca	probe
		vvi-miR399h|*compl_mismatch
vvi|miR-1924|2mut|as|	cagtgaaatccttctttggca	probe
		vvi-miR399i|*compl_perfect match
vvi|miR-1925|nat|as|	tagggcttctctccttctggc	probe
vvi|miR-1925|2mut|as|	taggccttctcttcttctggc	vvi-miR399i|*compl_mismatch probe
		vvi-miR403a|*compl_perfect match
vvi|miR-1926|nat|as|	agtttgtgcgtgaatcaaa	probe
		vvi-miR403a|*compl_mismatch
vvi|miR-1926|2mut|as|	agtttgtgagcgaatcaaa	probe
		vvi-miR403b|*compl_perfect match
vvi|miR-1927|nat|as|	cgagtttgtgcgcgaatccaa	probe
		vvi-miR403b|*compl_mismatch
vvi|miR-1927|2mut|as|	cgagtttgtgagagaatccaa	probe
		vvi-miR403c|*compl_perfect match
vvi|miR-1928|nat|as|	caagtttgtgcgtgaatcaaa	probe
		vvi-miR403c|*compl_mismatch
vvi|miR-1928|2mut|as|	caagtttgtgactgaatcaaa	probe
		vvi-miR403d|*compl_perfect match
vvi|miR-1929|nat|as|	cgagtttgtgcgcgaatc	probe
		vvi-miR403d|*compl_mismatch
vvi|miR-1929|2mut|as|	cgagtttgcgcgagaatc	probe
vvi|miR-1930|nat|as|	caagtttgtgcgtgaatcaaa	vvi-miR403e|*compl_perfect match

<table>
<tr><td></td><td></td><td>probe</td></tr>
<tr><td>vvi|miR-1930|2mut|as|</td><td>caagtttgtgactgaatcaaa</td><td>vvi-miR403e|*compl_mismatch probe</td></tr>
<tr><td>vvi|miR-1931|nat|as|</td><td>gagtttgtgcgtgactctaa</td><td>vvi-miR403f|*compl_perfect match probe</td></tr>
<tr><td>vvi|miR-1931|2mut|as|</td><td>gagtttgtgactgactctaa</td><td>vvi-miR403f|*compl_mismatch probe</td></tr>
<tr><td>vvi|miR-1932|nat|as|</td><td>cggggacgaggtagtgcat</td><td>vvi-miR408|*compl_perfect match probe</td></tr>
<tr><td>vvi|miR-1932|2mut|as|</td><td>cggggaagagctagtgcat</td><td>vvi-miR408|*compl_mismatch probe</td></tr>
<tr><td>vvi|miR-1933|nat|as|</td><td>ttggaagacagtgggggacct</td><td>vvi-miR477a|*compl_perfect match probe</td></tr>
<tr><td>vvi|miR-1933|2mut|as|</td><td>ttggaagaaagtgggtgacct</td><td>vvi-miR477a|*compl_mismatch probe</td></tr>
<tr><td>vvi|miR-1934|nat|as|</td><td>gacgagccgaaccaatatcac</td><td>vvi-miR479|*compl_perfect match probe</td></tr>
<tr><td>vvi|miR-1934|2mut|as|</td><td>gacgagtcgaacaaatatcac</td><td>vvi-miR479|*compl_mismatch probe</td></tr>
<tr><td>vvi|miR-1935|nat|as|</td><td>ggaattggagagtagg</td><td>vvi-miR482a|*compl_perfect match probe</td></tr>
<tr><td>vvi|miR-1935|2mut|as|</td><td>ggaattcgagactagg</td><td>vvi-miR482a|*compl_mismatch probe</td></tr>
<tr><td>vvi|miR-1936|nat|as|</td><td>ggcgtgctctctctcgctgtca</td><td>vvi-miR535a|*compl_perfect match probe</td></tr>
<tr><td>vvi|miR-1936|2mut|as|</td><td>ggcgtcctctctctccctgtca</td><td>vvi-miR535a|*compl_mismatch probe</td></tr>
<tr><td>vvi|miR-1937|nat|as|</td><td>ggcgtgctctctctcgctgtca</td><td>vvi-miR535b|*compl_perfect match probe</td></tr>
<tr><td>vvi|miR-1937|2mut|as|</td><td>ggcgtcctctctctccctgtca</td><td>vvi-miR535b|*compl_mismatch probe</td></tr>
<tr><td>vvi|miR-1938|nat|as|</td><td>ggcgtgctctctctcgctgtca</td><td>vvi-miR535c|*compl_perfect match probe</td></tr>
<tr><td>vvi|miR-1938|2mut|as|</td><td>ggcgtcctctctctccctgtca</td><td>vvi-miR535c|*compl_mismatch probe</td></tr>
<tr><td>vvi|miR-1939|nat|as|</td><td>ggcgtgctctctgtcgctgtca</td><td>vvi-miR535d|*compl_perfect match probe</td></tr>
</table>

vvi|miR-1939|2mut|as|	ggcgtcctctctgtccctgtca	vvi-miR535d|*compl_mismatch probe
vvi|miR-1940|nat|as|	ggcgtgctctctgtcgctgtca	vvi-miR535e|*compl_perfect match probe
vvi|miR-1940|2mut|as|	ggcgtcctctctgtccctgtca	vvi-miR535e|*compl_mismatch probe
vvi|miR-1941|nat|as|	tgagatgctcatttgaggaag	vvi-miR828a|*compl_perfect match probe
vvi|miR-1941|2mut|as|	tgagatcctcatttgacgaag	vvi-miR828a|*compl_mismatch probe
vvi|miR-1942|nat|as|	tggatagtcatttcctgcaa	vvi-miR828b|*compl_perfect match probe
vvi|miR-1942|2mut|as|	tggatagccattttctgcaa	vvi-miR828b|*compl_mismatch probe
vvi|miR-1943|nat|as|	attagttccttcatgagtta	vvi-miR845a|*compl_perfect match probe
vvi|miR-1943|2mut|as|	attagtttcttcataagtta	vvi-miR845a|*compl_mismatch probe
vvi|miR-1944|nat|as|	attagttccttcatgagtt	vvi-miR845b|*compl_perfect match probe
vvi|miR-1944|2mut|as|	attagtttcttcataagtt	vvi-miR845b|*compl_mismatch probe
vvi|miR-1945|nat|as|	tgttttggttgtctgagttt	vvi-miR845c|*compl_perfect match probe
vvi|miR-1945|2mut|as|	tgttttcgttctctgagttt	vvi-miR845c|*compl_mismatch probe
vvi|miR-1946|nat|as|	cattaattatccctagggcca	vvi-miR845d|*compl_perfect match probe
vvi|miR-1946|2mut|as|	cattaattattcctagtgcca	vvi-miR845d|*compl_mismatch probe
vvi|miR-1947|nat|as|	cattaattatccctagggcca	vvi-miR845e|*compl_perfect match probe
vvi|miR-1947|2mut|as|	cattaattattcctagtgcca	vvi-miR845e|*compl_mismatch probe

Degradation and Negative control probes have been used to define background level. Mismatch probes have been designed by the Combimatrix Company for each perfect match probe, to asses the quality of each hybridization. For each miRNA precursor molecules many different probes have been designed. In particular there are probes designed on the predicted mature miRNA; probes designed on the predicted miRNA* (marked as |*); probes shifted of 5 or 10 nucleotide towards the 5' (|+5 and |+10) or the 3'(|-5 and |-10)with respect to the start of the expected mature miRNA; probes shifted of 5 or 10 nucleotide towards the 5' (|*+5 and |*+10) or the 3'(|*-5 and |*-10)with respect to the start of the expected mature miRNA*, probes of exactly 20 nucleotide designed on the predicted miRNA, when longer than 20nt (|0); probes of exactly 20 nucleotide designed on the predicted miRNA*, when longer than 20nt (|*0), probes designed on the precursor sequence, outside the miRNA or miRNA* region (|ctrl) and finally complementary probes to the miRNA probe (|compl) and to the miRNA* (|*compl).
