# Supplementary material for: Correction: High throughput approaches reveal splicing of primary microRNA transcripts and tissue specific expression of mature microRNAs in Vitis vinifera
Source: BMC Genomics. 2010 Feb 12;11:109. doi: 10.1186/1471-2164-11-109 (PMC2831844; doi:10.1186/1471-2164-11-109)
Supplement: Additional file 1 — Expression data for Vitis vinifera miRNAs. Supplemental Figure S1: Log expression levels of all predicted miRNAs in all tissues as detected by oligonucleotide array. Supplemental Figure S2: Detailed alignments and frequencies of all small RNA reads mapping to miRNA precursors. Supplemental Table S3: transcript data for 25 Vitis vinifera miRNAs. Supplemental Figure S4: Splice junction read coverage for Vvi-miR394b, Vvi-miR162 and Vvi-miR168. [file 1471-2164-11-109-S1.PDF]

Supplemental Figure S1: Log Expression levels of all predicted miRNAs in all tissues tested by oligonucleotide array. Error bars represent confidence intervals. Y axis represents Log2 of the normalized median of spot intensities

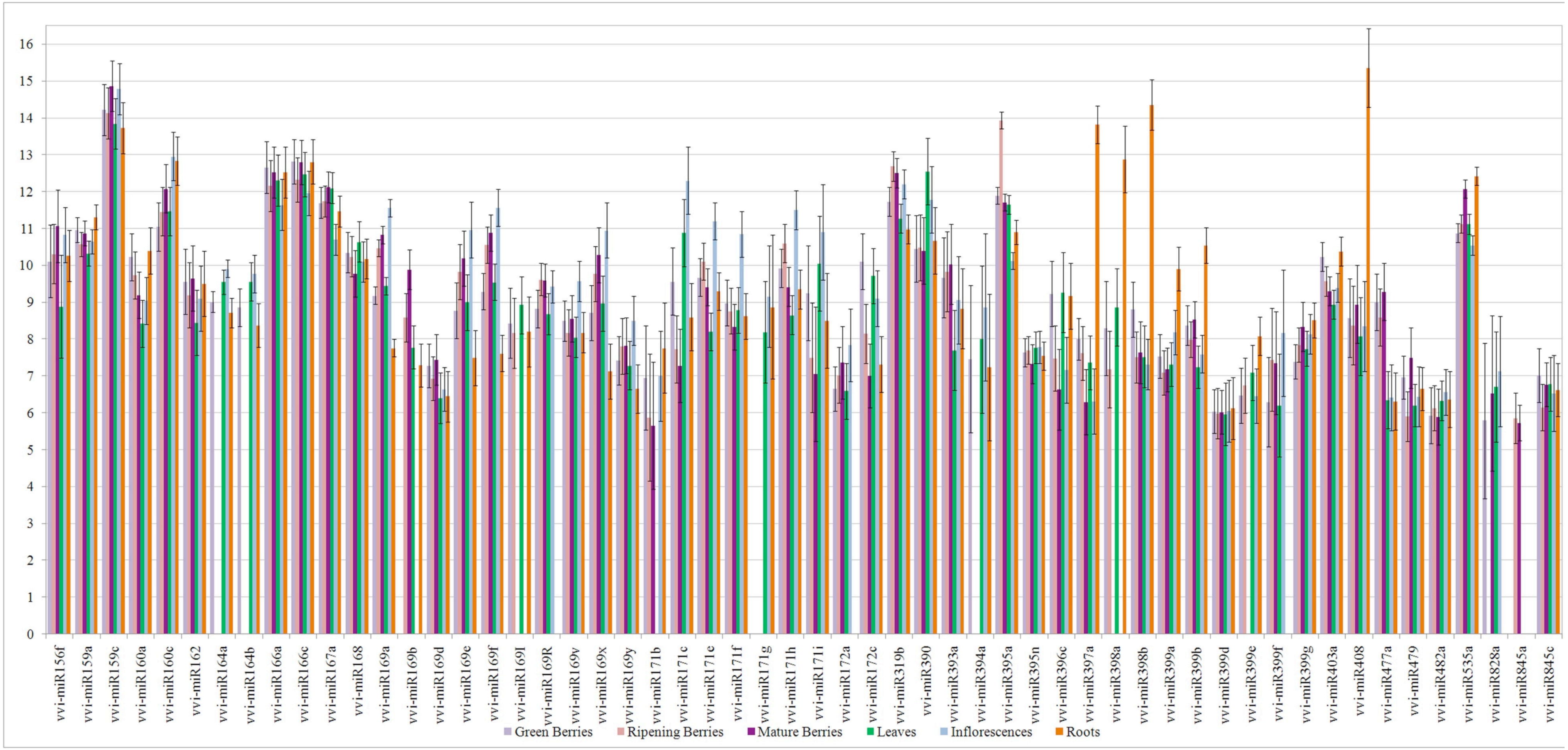

Supplemental Figure S2: Alignments of all small reads mapping to predicted pre-miRNAs and their putative secondary structures: Scaffold, and Chromosome coordinates as well as predicted secondary structures are shown for all putative microRNA precursors considered in this work. Frequencies of matching smallRNA reads aligned to precursors are shown. Intitial mature sequences predicted are shown in red, preferred designations of mature sequences are shown as underlined text.

```
>vvi-MIR156a [sc:66 - 769283 769386] [chr:19 - 8702104 8702207]
......(((.((((((((((((((((((((.(((((.(..(((.((((...)))))))..).)))))..))))))))))).))))))))).)))........                (-46.90)
AGAAAUAUGUUGACAGAAGAGAGGGAGCACAACCCAGCAUCAGCUAAAGACUCUUUGCUUUUCUUGGGAGUGUGCUCUCUCUUCUUCUGUCAUCAUCACACUCC
                                                                             GCUCUCUCUUCUUCUGUCAUCAUC       6
          ugacagaagagagggagcac&             0

>vvi-MIR156b [sc:6 - 5342190 5342291] [chr:4 - 5351258 5351359]
((((.((.((((((((((((((((((((.((((((((..((((....)))))..))))))))).)))))))).))))).))))))))).)).))))).. (-53.30)
CAUUGAAAACUGACAGAAGAGAGUGAGCACACAGAGGCAAUUGUAUAAGUUUAUGCUUUUGCUUUUGCGUGCUCAUUUCUCUUUCUGUCAGCUUCCAGUGCC
          ugacagaagagagugagcac&             16
          UGACAGAAGAGAGUGAGCACa             16
          UGACAGAAGAGAGUGAGCACAC            9
          UGACAGAAGAGAGUGAGCACaa            2
          UGACAGAAGAGAGUGAGCACACA           1
          UGACAGAAGAGAGUGAGC                1

>vvi-MIR156c [sc:6 - 841232 841335] [chr:4 - 850300 850403]
.((((.((((((((((((((((((((((((.((.(((...(((..(((((....)))))..)))))).)).)))))))))))))).))))))))))))))))....                (-60.20)
AAGGAAUAGGUGACAGAAGAGAGUGAGCACACAUGGUCUGUUCUUGCAUGGGUCCAUGCUUGAAGCUCUGCGUGCUCACUCUCUAUCUGUCAUCUGUCCUCUCU
          ugacagaagagagugagcac&             16
          UGACAGAAGAGAGUGAGCACA             16
          UGACAGAAGAGAGUGAGCACAC            9
          UGACAGAAGAGAGUGAGCACAa            2
          UGACAGAAGAGAGUGAGCACACA           1
          UGACAGAAGAGAGUGAGC                1

>vvi-MIR156d [sc:14 + 5411513 5411615] [chr:11 - 5633893 5633995]
.....((..((((((((((((((((((((..((.((((....(((((....)))))...)))).))..))))))).))))).)))))))))..)).......(-47.50)
AGAGAGAGACUGACAGAAGAGAGUGAGCACAUGCAGGCAAUUUGUAUAAGGGUAUACCUAUGCCGGUGCGUGCUCACCUCUCUUUCUGUCAGCUUCAGGUGCU
          UGACAGAAGAGAGUGAGCACAU&           39
          ugacagaagagagugagcac              16
          UGACAGAAGAGAGUGAGCACa             16
          UGACAGAAGAGAGUGAGCACAUa           13
          UGACAGAAGAGAGUGAGCACaa            2
          UGACAGAAGAGAGUGAGCACAUG           2
           GACAGAAGAGAGUGAGCACAUa           1
          UGACAGAAGAGAGUGAGC                1
                                                                        GCUCACCUCUCUUUCUGUCAGCa          1

>vvi-MIR156e [sc:13 - 1481673 1481780] [chr:11 - 1481673 1481780]
((.(((..((((((((((.(((((((((.((.(((....(((..(((((.......)))))..)))))).)).)))))))))))))..)))))))))..))).))..                (-48.20)
GUAUGGUAGGUGACAGAGGAGAGUGAGCACUCAUGGUUCUUUUCUUGCAUGAAGUUUCUAUGCUUGAAGCUCUGUGUGCUUACUCCCUAUCUGUCACCCCUCAAGCAU
          ugacagaggagagugagcac&             0

>vvi-MIR156f [sc:21 + 498862 498966] [chr:14 + 15664264 15664368]
....((((((((((((.(((.((((((.(((((((..((.((.((((....)))).)).))..)))))..))).))))))..))).)))))))))))))......                (-45.40)
UUAUGUUCUGUUGACAGAAGAUAGAGAGCACAACUGAACAUACCCAAGAGGGCUUGUGUUUGAGCAGUCUUGUCCUCUCUCUUCUCCUGUCAACAGAACUUGCUG
          uugacagaagauagagagcac&            497
          UUGACAGAAGAUAGAGAGCACa            425
          UUGACAGAAGAUAGAGAGCACAAC          32
           UGACAGAAGAUAGAGAGCAC             12
          UUGACAGAAGAUAGAGAGC               10
           UGACAGAAGAUAGAGAGCACa            6
          UUGACAGAAGAUAGAGAGCA              5
           UGACAGAAGAUAGAGAGCACAAC          4
          UUGACAGAAGAUAGAGAGCACaa           3
            GACAGAAGAUAGAGAGCACa            2
          UUGACAGAAGAUAGAGAGCAa             1

>vvi-MIR156g [sc:16 + 2308172 2308274] [chr:17 - 3037544 3037646]
((((......((((((((.((((((((((((.(((((.(((((.......))))).))).)).)))))))))))))..)))))))))))))........(-50.00)
GGUGAUGUUGUUGACAGAAGAUAGAGAGCACAGAUGAUGAUGUGCAUGGAGUUUGCAUCUCACUCCUUUGUGCUCUCUAGACUUCUGUCAUCACCUCAGCCCC
          uugacagaagauagagagcac&            497
          UUGACAGAAGAUAGAGAGCACA            425
           UGACAGAAGAUAGAGAGCAC             12
          UUGACAGAAGAUAGAGAGC               10
                                                                        GCUCUCUAGACUUCUGUCA              7
          UUGACAGAAGAUAGAGAGCACAGA          7
           UGACAGAAGAUAGAGAGCACA            6
                                                                        GCUCUCUAGACUUCUGUCAUCAC          6
          UUGACAGAAGAUAGAGAGCACAGAa         5
                                                                     UGUGCUCUCUAGACUUCUGUCA              5
          UUGACAGAAGAUAGAGAGCA              5
                                                                        GCUCUCUAGACUUCUGUCAUCA           5
                                                                        GCUCUCUAGACUUCUGUCAUC            4
            GACAGAAGAUAGAGAGCACA            2
                                                                     UGUGCUCUCUAGACUUCUGUCAUCA           2
          UUGACAGAAGAUAGAGAGCACAG           1
                                                                           CUCUAGACUUCUGUCAUC            1
                                                                    UUGUGCUCUCUAGACUUCUGUCAUC            1
                                                                        GCUCUCUAGACUUCUGUCAUCACC         1
          UUGACAGAAGAUAGAGAGCAa             1

>vvi-MIR156h [sc:18 - 820816 821043] [chr:12 + 4070523 4070750]
.((..((((((((.((..((((((((((......((((.......((((((((((((((((....)))))..)))).).))))))..((((.....(..(((....)))..)....))))...............)))))............(((.((((((((......(((....)))....)))).)))).))))))))))))).)..)).))))))))....)).        (-59.90)
UGCCUCACAAUGACAGAAGAGAGAGAGCAUGCUGGUGGGAAAACAAUUACAACUUUUGAUCAUCUGAUCUGGAAAUGCUUGUAAGCGGCAUUCUCUUGGAUUGUAAUCUGAAUUCUGCCUCUAUCAUCAACCUGCCCACAAACGAUUUCCUUCAACUGAGUGCCUUUCCGGCUUGAGCCUUCUGCAUGAUCAGCUGAGUUCUUUCUGCGCCUUUCAUUGUGUCCUGCC
          ugacagaagagagagagcau&             0

>vvi-MIR156i [sc:167 + 235850 235952] [chr:Un_random + 36776839 36776941]
((((......((((((((((((((((((((((.(((((.((((.........)))).))).)).)))))))))))))).)))))))))))))))........(-49.00)
GGUGACGUUGUUGACAGAAGAUAGAGAGCACAGAUGAUGAAAUGCAAGGAGCCAGCAUCUCACUCCUUUGUGCUCUCUAUUCUUCUGUCAUCACCUUAAGGCC
          UUGACAGAAGAUAGAGAGCAC&            497
```

UUGACAGAAGAUAGAGAGCACA 425
ugacagaagauagagagcac 12
UUGACAGAAGAUAGAGAGC 10
UUGACAGAAGAUAGAGAGCACAGA 7
UGACAGAAGAUAGAGAGCACA 6
UUGACAGAAGAUAGAGAGCACAGAa 5
UUGACAGAAGAUAGAGAGCA 5
GACAGAAGAUAGAGAGCACA 2
UUGACAGAAGAUAGAGAGCACAG 1
GCUCUCUAUUCUUCUGUCAUCA 1
GCUCUCUAUUCUUCUGUCA 1
GCUCUCUAUUCUUCUGUCAUC 1
UUGACAGAAGAUAGAGAGCAa 1
UGCUCUCUAUUCUUCUGUCAUCAa 1

>vvi-MIR159a [sc:40 + 1876099 1876292] [chr:15_random + 3461857 3462050]
((((.((((((((((((((.((((((((..((..(((((......((((.((((..((.(.(((.((((..(((((((((.((((((..((((......))))..))))))..)))))))))..)))))))..).))..)))).))))....)))))..))..)))))))))))))))))))))))))))... (-93.20)
GGGUUUAUGGGAGCUCCUUUACGCUCCAGUUCUGAAAGGAGAUGAUGGUAUCCACAGCUGCUGGUUCAUGAGUACCUAUGGCUGCACAAUAUAUAUAGCAAUGUUGUGUGCAAACUAUGGGUGUGCAUGACCUCGGAGAAGUGGUUGCCUUGAUCUUUCUGGCCUUGGAGUGAAGGGAGCUCUCAUAACCCUUC
cuuggagugaagggagcucuc& 0

>vvi-MIR159b [sc:40 + 1873406 1873590] [chr:15_random + 3459164 3459348]
.((.((((((((((((((((.((((((((...(((((.(((((((.((.(.((((((((.((((((((((.....(((.........)))..((((....))))))))))))))))).)))))))).).)).)))))))).)))))).(......)...))))))))))))))))))))))))))))).... (-75.20)
GGGCUUGUGGGAGCUUCUUUACACUCCAGAACUGAAAGGAGAUACUACUGCUGGUUCAUGAAAACCUAUGGCUGCAUUCUCUAUACUGAAAUUUUGUAUACAAACUAUGGGUUUGCAUGAACUUGGAGAAGUGUUUGCUUUUAUCUUUUCUGGCCUUGGAGUGAAGGGAGCUCUCAUAACCGAUC
cuuggagugaagggagcucuc& 0

>vvi-MIR159c [sc:16 + 2735981 2736170] [chr:17 - 2609648 2609837]
....((.((((((((((((..((((((..((((((((.((((((((..((((((.((((..((((.(..((((((.(((((((.(((.......)))...))))))).))))))..).))))..)))).)))).))))))))).)))..))))))))..)))))))..))))))))))))).))...... (-92.40)
UUUUGGAGUGGAGCUCCUUGAAGUCCAAUAGAGGGUCUUACUGGGUAGAUUGAGCUGCUGACUUAUGGAUCCCACAGCCCUAUCCCGUCAAUGGGGGGCAUUGGAUAGGCUUGUGGCUUGCAUAUCUCAGGAGCUGCAUUAUCCAAGUUUAGAUCCUUGUUUGGAUUGAAGGGAGCUCUACACCUCUCUC
UUUGGAUUGAAGGGAGCUCUAa& 166
uuuggauugaagggagcucua 7
UUUGGAUUGAAGGGAGCUCUACA 6
UUUGGAUUGAAGGGAGC 5
UUUGGAUUGAAGGGAGCUCUAC 5
UUGGAUUGAAGGGAGCUCUAa 2

>vvi-MIR160a [sc:47 + 660403 660505] [chr:12 + 8539030 8539132]
((((....((((.((((((((((.(((((.(((.((((...(((((.....)))))...)))).))).))))))))).))))))).)))))))))......(-52.30)
GGGGUUGAUCUGCCUGGCUCCCUGAAUGCCAUCUAAGAAGCUUGUCAAGGUUGUUGACUCUCUUCCUAGUUGGCAUCAGAGGAGUCAUGCAGGCCCCAACAUU
UGCCUGGCUCCCUGAAUGCCAUC& 1
ugccuggcucccugaaugcca 0

>vvi-MIR160b [sc:383 + 36508 36611] [chr:Un_random + 78660926 78661029]
....((((.((((.(((((((((..((((((..((.((((.(((((((......))))))).)))).))..))))))..))))))))).))))))))....... (-47.00)
AAUAUUGUUCUGCCUGGCUCCCUGAAUGCCAUCUAAGAAGCUUGUUAAAGAGUGUUGACAACCUUCCUAUUUGGCAUGAGGGGAGUCAAGCAGGCGAAACACUU
UGCCUGGCUCCCUGAAUGCCAUC& 1
ugccuggcucccugaaugcca 0

>vvi-MIR160c [sc:76 + 752851 752956] [chr:10 - 8894085 8894190]
...(((((.((((.(((((((.((((((((((.((((((..(((.(((.......))).)))...)))))).)))))))))).))))))).))))))))))...... (-58.30)
AAAUGGGUUGUGCCUGGCUCCCUGUAUGCCACACGUAGAGACCACUCCCUUUCCCGGAUUGGCUAUUUGCGGGUGGCGUGCGAGGAGCCAAGCAUACCCACCAUUC
ugccuggcucccuguaugcca& 2
GCGUGCGAGGAGCCAAGCAUAa 1

>vvi-MIR160d [sc:29 - 1349764 1349865] [chr:8 + 11902361 11902462]
........((((.(((((((.((((((((((((((.(((.(.(((.........))).).))).)))))))))))))).))))))).)))))......... (-49.30)
CAUACUUACAUGCCUGGCUCCCUGUAUGCCAUUUGCAAAGUCCACCCGAGGAUUGGUAGCCUUCGCGGAUGGCGUGCGAGGAGCCAUGCAUGCAUCUUGCUU
ugccuggcucccuguaugcca& 2

>vvi-MIR160e [sc:1809 - 11818 11921 [scaffold not incorporated in genome assembly]
....((((.((((.(((((((((..((((((..((.((((.(((((((......))))))).)))).))..))))))..))))))))).))))))))....... (-47.00)
AAUAUUGUUCUGCCUGGCUCCCUGAAUGCCAUCUAAGAAGCUUGUUAAAGAGUGUUGACAACCUUCCUAUUUGGCAUGAGGGGAGUCAAGCAGGCGAAACACUU
UGCCUGGCUCCCUGAAUGCCA 0
UGCCUGGCUCCCUGAAUGCCAUC& 1

>vvi-MIR160f [sc:17 - 2195098 2195199] [chr:13 + 5553097 5553198]
...(((((..(((.(((((((..((((((((((..(..((.(((.(((....))).))).))..)..))))))))))..))))))).))))))))....... (-51.10)
UAAGCAUAUAUGCCUGGCUCCCUGUAUGCCAUUUGCAGAGCCCACCGGCACAUCGAUGGCCUUCGUGGAUGGCGUAUGAGGAGCCAUGCAUAUGCCCCAUCU
ugccuggcucccuguaugcca& 2

>vvi-MIR162 [sc:16 - 630729 630831] [chr:17 + 4714987 4715089]
((((..(.((((((((((.((((.(((((((..(((.(((..(((((.........))))).))).)))..))))))).)))).)))))))))).))))).....(-41.70)
UGAAGUCACUGGAUGCAGCGGUUCAUCGAUCUCUUCCUGAAAUUGUUGUGAAAAAAGCAGAUCAAGAAUCGGUCGAUAAACCUCUGCAUCCAGCGUUCACUCC
UCGAUAAACCUCUGCAUCCAGa& 5
ucgauaaaccucugcauccag 0

>vvi-MIR164a [sc:42 - 451600 451720] [chr:7 - 3651395 3651515]
(((.((.((((((((((..(((((((((.((.......))..................................(((....)))..)))))))))..))))))))))).))))... (-45.00)
AGCUCCUUGUUGGAGAAGCAGGGCACGUGCAGAUUUGCCUCACUUUUCCCCCUUUUUUCUUCUUUACUCCCACCACCGCCCACAGGCUUGCACGUGCUCCCCUUCUCCAACAUGGGUUCCU
uggagaagcagggcacgugca& 5
UGGAGAAGCAGGGCACGUGCAa 2

>vvi-MIR164b [sc:7 - 511008 511100] [chr:9 - 511008 511100]
....(((.(..(((((..((((((((((..(((((..((((.........))))))))))..)))))))))))..)))))..).)))...... (-38.80)
UAAACCAUGCUGGAGAAGCAGGGCACAUGCUGGAUCAAUCAGCACCUGGAUCUGAGAUUUUACAUGUGCCCUGGCUCUCCCACUUGGAUCCUA
Uggagaagcagggcacaugcu& 0

>vvi-MIR164c [sc:88 + 655651 655747] [chr:8 + 8981162 8981258]
...((((((..((((..(((.(...).))))....(((((((..(((..................)))..))))))).))))..)))))))....... (-25.61)
UUGAGCAAGAUGGAGAAGCAGGGCACGUGCAUUACUAGCUCAUGCACCACAAACCAAUAAUCUUUUGCUCGAGUUAGACUCUUGCUUGCUGCUUUGA
uggagaagcagggcacgugca& 5
UGGAGAAGCAGGGCACGUGCAa 2

>vvi-MIR164d [sc:27 - 1404604 1404721] [chr:14 - 1404604 1404721]
.(((((.((.((((((((..((((((((((((..((............((..((((....))))..))............))..))))))))))))..)))))))).)).)))))... (-48.95)
AAGCUCUUGAUGGAGAAGCAGGGCACGUGCAGUUCACAAAUUCUAAUCUGCUCUGCACGUGCAGUUCACAAAUUCUAAUCUGCUCUGCACGUGCUCCCCUUCUCCAACAUGGGUUCCU
uggagaagcagggcacgugca& 5
UGGAGAAGCAGGGCACGUGCAa 2

>vvi-MIR166a [sc:41 - 494693 494822] [chr:8 - 3340277 3340406]
((.((((((.(((((((((((((.(((.(((((............................((((((.((((..........)))).)))))).))))).))).))))))))))))).))))))..)). (-45.41)
GCGGGUGUUUGGAAUGAGGUUUGAUCCAAGAUCCUUCCUUUUUCUCCAUUAAUUCCUUCAGUACUGGUCAUGAUUUAGUUACUUCAUAAUCGGUUGAUCUCGGACCAGGCUUCAUUCCUGACACCAAGCU
UCGGACCAGGCUUCAUUCC& 43
UCGGACCAGGCUUCAUUC 4
AAUGAGGUUUGAUCCAAGAUCC 1

```
                                                                              UCUCGGACCAGGCUUCAUUCCa                 1
                                                                              UCUCGGACCAGGCUUCAU                     1
                                                                                ucggaccaggcuucauuccug                0

>vvi-MIR166b [sc:38 - 1125312 1125410] [chr:12 + 15726184 15726282]
((..((((((((((((..(.((((..(((.((.(((((..................))))).)).))).)..)))).)..))))))))))))...)).    (-42.41)
GUUUUUUGAGGGGAAUGUUGGCUGGCUCGAGGCAUUCACAUAAGAAGAAGAUAUAUAAGUGAAGGUGUCGGACCAGGCUUCAUUCCUCUCAAAUAUACA
                                                           UCGGACCAGGCUUCAUUCC&                43
                                                           UCGGACCAGGCUUCAUUC                  4
                                                           UCGGACCAGGCUUCAUUCCUCa              4
                                                           UCGGACCAGGCUUCAUUCCUCUC             2
                                                           ucggaccaggcuucauuccuc               1
          GGAAUGUUGGCUGGCUCGAGGa            1

>vvi-MIR166c [sc:37 + 128461 128620] [chr:15 - 7564994 7565153]
.....(((.((((((((..((((((..(((.((((((...........((((..(..((....))..)..)))).....((((((((......(((.........)))...)))))))).)))))).))).)).)))))..))))))))).))).......   (-52.60)
CUAUUUUGAGGGGAAUGUUGUCUGGCUCGAGGACACUUACCUUAACUUUGAUCCGUUUCUCUAGAUCCAUAUCACCUAUAUAUAUGUAUUAUUGUAUUUUUAUUUGUACUGAGCAUGUGUUGGUGUCGUCGGACCAGGCUUCAUUCCCCCAAUUAUCGC
                                                                                                                                   UCGGACCAGGCUUCAUUCCCCa&            52
                                                                                                                                   UCGGACCAGGCUUCAUUCC                43
          GGAAUGUUGUCUGGCUCGAGGA            14
          GGAAUGUUGUCUGGCUCGAGGAC           12

                                                                                                                                   UCGGACCAGGCUUCAUUCCCC              11
                                                                                                                                   UCGGACCAGGCUUCAUUC                 4
                                                                                                                                   UCGGACCAGGCUUCAUUCCCCCa            4
                                                                                                                                   ucggaccaggcuucauuccccc             4
                                                                                                                                   UCGGACCAGGCUUCAUUCCC               2
                                                                                                                                   UCGGACCAGGCUUCAUUCCCCCCa           1
          GGAAUGUUGUCUGGCUCGAGGACAa         1
                                                                                                                                   UCGGACCAGGCUUCAUUCCCCCC            1

>vvi-MIR166d [sc:86 - 899379 899538] [chr:16 - 7520920 7521079]
.....((((((((.((..((((((..(((((.(((.((((...(((((.....))))).....((((((((....................................))))))).).))).))).))))).))))))..)).)))))))))).......   (-50.99)
CUGCUUUGAGGGGAUUGUUGUCUGGCUCGAGGCCACCAACCAUCUUUGAUUGAUUCAAAUACUUUGAUCAGCUUCAACCCAUCUUCUCCUUCAAUUCAAUUCCACCCUCUGAUUAUGUGUUAGUGCCCUCGGACCAGGCUUCAUUCCCCUCAAUCACUGC
                                                                                                                                   UCGGACCAGGCUUCAUUCCCCUC&           64
                                                                                                                                   UCGGACCAGGCUUCAUUCCCCa             52
                                                                                                                                   UCGGACCAGGCUUCAUUCC                43
                                                                                                                                   UCGGACCAGGCUUCAUUCCCC              11
                                                                                                                                   UCGGACCAGGCUUCAUUC                 4
                                                                                                                                   UCGGACCAGGCUUCAUUCCC               2
                                                                                                                                   ucggaccaggcuucauuccccu             2

>vvi-MIR166e [sc:105 - 1099967 1100132] [chr:2 + 2180274 2180439]
(((..(((.((((((((..((((((..(((.(((((((((.....((((((((.(((......)))........(((((.(((..((((((......))))))..))))))))...))))))))))))))))).))).)))))))..)))))))).))).))).)))..            (-71.80)
GUGUUUUGAGGGGAAUGUUGUCUGGCUCGAGGACACCAACUAGAUCUAUGAUCUGCGUGUAAUUGUGAAUGGGUGAUCGUCUCUCAGUUUUGAAGGAGAGAUUUUGAGUGAUCUUUGAUCAUGGGUUGGUGUCGUCGGACCAGGCUUCAUUCCCCCAAUUUAUUG
                                                                                                                                        UCGGACCAGGCUUCAUUCCCCa&      52
                                                                                                                                        UCGGACCAGGCUUCAUUCC          43
          GGAAUGUUGUCUGGCUCGAGGA            14
          GGAAUGUUGUCUGGCUCGAGGAC           12

                                                                                                                                        UCGGACCAGGCUUCAUUCCCC        11
                                                                                                                                        UCGGACCAGGCUUCAUUC           4
                                                                                                                                        ucggaccaggcuucauuccccc       4
                                                                                                                                        UCGGACCAGGCUUCAUUCCCCCa      4
                                                                                                                                        UCGGACCAGGCUUCAUUCCC         2
                                                                                                                                        UCGGACCAGGCUUCAUUCCCCCC      1
          GGAAUGUUGUCUGGCUCGAGGACAa         1


>vvi-MIR166f [sc:20 - 663678 663777] [chr:7 + 14569971 14570070]
(((.((((.((((((((..(.((((..(((((((((.(((((..((.......))..)))))))))))))))..)))).)..)))))))).)))))))...   (-49.50)
UGUAGUUGUGGGGAAUGUUGGCUGGCUCGAGGCUUUCUUGAGUGAUUUUAUGUAUACCUCAAAAAGCUUCGGACCAGGCUUCAUUCCCCUCAACACACUU
                                                             UCGGACCAGGCUUCAUUCCCCUC&              64
                                                             UCGGACCAGGCUUCAUUCCCCa                52
                                                             UCGGACCAGGCUUCAUUCC                   43
                                                             UCGGACCAGGCUUCAUUCCCC                 11
                                                             UCGGACCAGGCUUCAUUC                    4
                                                             UCGGACCAGGCUUCAUUCCC                  2
                                                             ucggaccaggcuucauuccccu                2
                                                            UUCGGACCAGGCUUCAUUCCCCUC               1
          GGAAUGUUGGCUGGCUCGAGGa            1

>vvi-MIR166g [sc:31 - 2931810 2931909] [chr:7 + 257887 257986]
....((((((((((((..(((((((((((((((.((((.(((..((....))..))).)))).))))))))))))))).)..)))))))))))))......   (-57.00)
AACAGUUGAGGGGAAUGUUGUCUGGUUCGAGAUCCUUCGUAUGAAGCUCAAGCAGCGUUUGAAUGAUUUCGGACCAGGCUUCAUUCCCCUCAACCCAAAA
                                                             UCGGACCAGGCUUCAUUCCCCUC&              64
                                                             UCGGACCAGGCUUCAUUCCCCa                52
                                                             UCGGACCAGGCUUCAUUCC                   43
                                                             UCGGACCAGGCUUCAUUCCCC                 11
                                                             UCGGACCAGGCUUCAUUC                    4
                                                             UCGGACCAGGCUUCAUUCCC                  2
                                                             ucggaccaggcuucauuccccu                2
          GGAAUGUUGUCUGGUUCGAGAUC           1
                                                            UUCGGACCAGGCUUCAUUCCCCUC               1

>vvi-MIR166h [sc:2 - 4521489 4521588] [chr:5 - 6593621 6593720]
...((((.((((((.(..((((((((((((.((...((.....)).((((.....))))..)).))))))))))))..).)))))).))))).....   (-42.50)
AACAGUUGAGGGGAACGCUGUCUGGUUCGAGACCACUCGCUUCAAGUACAGACAUAAUCUGAAUGAUCUCGGACCAGGCUUCAUUCCCCCAACUCAACA
                                                             UCGGACCAGGCUUCAUUCCCCa&               52
                                                             UCGGACCAGGCUUCAUUCC                   43
                                                             UCGGACCAGGCUUCAUUCCCC                 11
                                                             UCGGACCAGGCUUCAUUC                    4
                                                             UCGGACCAGGCUUCAUUCCCCCa               4
                                                             ucggaccaggcuucauuccccc                4
                                                             UCGGACCAGGCUUCAUUCCC                  2
                                                             UCGGACCAGGCUUCAUUCCCCCCa              1
                                                           UCUCGGACCAGGCUUCAUUCCa                  1
                                                             UCGGACCAGGCUUCAUUCCCCCC               1
                                                           UCUCGGACCAGGCUUCAU                      1

>vvi-MIR167a [sc:5 + 1563504 1563854] [chr:1 + 1563504 1563854]
..((((.(((((((((((((.((.(((((((((((.(.......(((..(((...((..((.((((((.....((((((.(....).))))))).....(((((((..(((((((((.((((((..(((((((((.........))))).....)))))).....)))))).....))))))....((((.......)))).....))))))))...(((....))).)))))))..................))))))..))...))..)))..)))......................((((.(((
....))).))))......).))))))))))))).))))))))))))).))))....      (-112.70)
UUUGAGAGGUUGAAGCUGCCAGCAUGAUCUGGUGAAACAAACACCAUCUCUUUCUUCUCUAACCCCAUGUCUGGAUUCGUCCACCGAUCCAUUAUUAUAGACCAGGCCGCCCGUUUCCCAUGUAGUGAUCGAUAAUUAGGCUCGGGGUUUUCACUUUUUAGUGGGAUCUAAUCCUUAGGAUGGAUGUUUGUAUGGGUGGUAUAUAUCAUGGUGAGGUCUGUUUUCUAUUUUAAUUCUAACGGGGUUUUGAUUUAGCUGAGGGGGUAUAAUUCAUAGCCUAAUUCCAAAACCUAACUC
CAUAGAGAUAGGGUUCCAUGAUCAGGUCAUCUUGCAGCUUCAAUCACUCACUCA
         UGAAGCUGCCAGCAUGA                 23
         UGAAGCUGCCAGCAUGAUC&              12

                   UCAGGUCAUCUUGCAGCUUCAAUC                                  2

                    AGGUCAUCUUGCAGCUUCAAU                  1
```

```
          ugaagcugccagcaugaucug              0

>vvi-MIR167b [sc:54 + 1827200 1827298] [chr:14 + 6802135 6802233]
((.((((((.((((((((((.((((((((..((((((((................))))))))..))))))))))))))))))).)))))).))..    (-47.69)
CAAUAGCAGUUGAAGCUGCCAGCAUGAUCUAAGCUUUUCUGUUGCCCACCCUUUCUCCAGGAAAGACUAGAUCAUGUGGCAGUUUCACCUGUUGAUGGA
          UGAAGCUGCCAGCAUGA                  23
          UGAAGCUGCCAGCAUGAUCUAa&            19
          UGAAGCUGCCAGCAUGAUC                12
          ugaagcugccagcaugaucua              6
         UUGAAGCUGCCAGCAUGAUCUa              2

>vvi-MIR167c [sc:31 + 2349952 2350042] [chr:7 - 839754 839844]
((.((((((.(((.(((((((.((((((((...(((.(((..........))).)))...)))))))))))))))).))).)))))).))..            (-37.00)
CAGUAGCAGUUGAAGCUGCCAGCAUGAUCUCAACUUCCCUAUACAAGUCAAGGAAAGAUCAGAUCAUGUGGUAGCCUCACCUGUUGAUGGG
          UGAAGCUGCCAGCAUGA                  23
          UGAAGCUGCCAGCAUGAUC_               12
          UGAAGCUGCCAGCAUGAUCUCa&            1
          ugaagcugccagcaugaucuc              0

>vvi-MIR167d [sc:31 + 2344759 2344873] [chr:7 - 844923 845037]
((((((..(((((.((((.(((((((((((((((((((((...(((................)))..))))))....))))))))))))..)).)))).))))).))))))..   (-46.79)
AGGGAAUAAGUGAAGCUGCCAGCAUGAUCUAGCUUUGGCUAGGGAUACAGAGAAAGAGAGAGAUCAGAGCUAACCCUAGCUAGGUCAUGCCCUGACAGCCUCACUCCUUCCUUCU
          UGAAGCUGCCAGCAUGA                  23
          UGAAGCUGCCAGCAUGAUC                12
          Ugaagcugccagcaugaucua&             6

>vvi-MIR167e [sc:2 + 3637629 3637718] [chr:5 + 5709761 5709850]
.((((.(((.((((((((((.((((((((............(((((....)))))...)))))))))))))))))))).)))....))))             (-36.06)
CACUAUCAGUUGAAGCUGCCAGCAUGAUCUAAACUUGCUUCCCUUUGUGAACAGAGAUCAGAUCAUGUGGCAGUUUCACCUGUUGUUGGU
          UGAAGCUGCCAGCAUGA                  23
          UGAAGCUGCCAGCAUGAUCUAa             19
          UGAAGCUGCCAGCAUGAUCUAAAC           13
          UGAAGCUGCCAGCAUGAUC                12
          Ugaagcugccagcaugaucua&             6
          UGAAGCUGCCAGCAUGAUCUAAA            5
         UUGAAGCUGCCAGCAUGAUCUA              2

>vvi-MIR168 [sc:113 + 853207 853338] [chr:2 - 16750063 16750194]
.(((((..(((((.((((((((((.(((((.(.((.(((((..((((((((...)))))))).....((((((((.......)))))))).))))).)).).))))).)))))))))).))))).)))))... (-72.40)
GGUCUCUAAUUCGCUUGGUGCAGGUCGGGAACCGACUUCGCCGCUCCGGCAGCGCCGGAGGCACGCGGCGGCCUACGAUUGGUUGCUGAGCGAAUUCCGAUCCCGCCUUGCAUCAACUGAAUCGGAGACGGC
          UCGCUUGGUGCAGGUCGGGAAa             2
                                                                                                     CCCGCCUUGCAUCAACUGAAUC          2
                                                                                                     CCCGCCUUGCAUCAACUGAAUa          1
          ucgcuuggugcaggucgggaa&             1

>vvi-MIR169a [sc:118 - 461597 461720] [chr:11_random - 1209766 1209889]
(((((.(((((.(((((((((..(((((((((..((((((((((((((((.........(((.....)))))))))).))))).))))).)))))..))))))))))..))))))))))).))))).))))))..          (-61.10)
GAGAGUGGAAUGCAGCCAAGGAUGACUUGCCGGAAUUUAUAUAUAGUGAGAAGAGAAGAGGCUAUAUGCUUUCACAUAUAUAGUGAACCCUGGCAAGUUGUCCUUGGCUACAUUUGAUUCUCUU
            cagccaaggaugacuugccgg&           0

>vvi-MIR169b [sc:118 - 310296 310400] [chr:11_random - 1058465 1058569]
(((((((((.(((((((((((((((.((((((((.(((((.((((((((...))))))))..))))).))).))))).)))))))))))))))))))))))))))...              (-61.20)
GGGGUCGAAUUGAGCCAAGGAUGGCUUGCCGUCAUCUGCAGCAAGAGUUGGAGACUUUUAACUGUGCGUGCACGGUUAGUCAUCCUUGGCUCAUUUGGCCCUUCU
          ugagccaaggauggcuugccg&             0

>vvi-MIR169c [sc:6 - 2254966 2255055] [chr:4 - 2264034 2264123]
((((((..((((.(((((((((..(((((((((((((((((....)))))))..)))))))))))..))))))))).)))))..)))))))..             (-60.00)
GGGAGUAGAAUGCAGCCAAGGAUGACUUGCCGGAGAUGGGGCAUUCCUCGUUAAUCCGGCAAGUUGUCCUUGGCUACAUUGGGCUCUCUU
            cagccaaggaugacuugccgg&           0

>vvi-MIR169d [sc:118 - 468088 468207] [chr:11_random - 1216257 1216376]
((((.(((((.(((((((.(..(((((((((..((((((((((((((((.........(((.....))))))))).))))).))))).)))))..)))))))))..).))))))).))))).)))).).           (-47.30)
GAGUGGAAUGCAGCCAAGAAUGAUUUGCCGGAAUUUAUAUAUAGUGAGAAGAGAAGAGGCUAUAGGCUUUCACAUAUAUAGUGAACCCUGGCAAGUUGUCCUUGGCUACAUUUGAUUCUC
          CAGCCAAGAAUGAUUUG   1
          Cagccaagaaugauuugccgg&             0

>vvi-MIR169e [sc:9 + 870068 870216] [chr:14 - 14285187 14285335]
((((.((..((((((((((((.(((((.(((((.((((...(((.((((((((....((..(((.......))).))....((((((.....)))))))).))))).))).))..)))...)))))))))))))))))).))).))))))...            (-49.90)
GUCUUGUCUGAUAGCCAAGGAUGACUUGCCUGCCCUAUCCCAAGUGGGUCAAUUUCUUUCUUUUUUCCAAGUAGUGUUUGAUAUUUGAUAUAUCAUGAAUCCGGUUGUUUGGGUUCCAGGCAGUCACCUUGGCUAAUUUUACAGGCUCU
           UAGCCAAGGAUGACUUGCCUGCa&          1
           uagccaaggaugacuugccug             0

>vvi-MIR169f [sc:84 - 316911 317094] [chr:1_random - 1379004 1379187]
((((.((((....(((((((.(..(((((((.........((((((.((((((.....(((................))).....))).)).)))))))........(((((.........((((((......)))))).))))).........))))))..).)))))))).)))))..)))))....        (-47.59)
GAUAUUAUGGUGCAGCCAAGGAUGACUUGCCGAAAAUAAUUUGCUCGAUCGUUAAUGUGCUUCUCCAGUUUCUAUUGCAAUUUAUGCAUAGGGCAAUAUAUAUAUCCUUUAUAGCUUGUUAAUUCAGAUAUUGACUGAGGAAUCAAUAAUGGGCAAGUUGUGUUUGGCUACAUGUUUAUCUCAU
            cagccaaggaugacuugccga&           0

>vvi-MIR169g [sc:34 + 1507120 1507240] [chr:8 + 20118758 20118878]
(((((....(((.(((((((((..((((((((.((.((...((((....((((...((((....)))).)...)))).)..)))).)))).))))))))..))))))))).)))....)))))..            (-52.20)
GAAAGUAUGGUGCAGCCAAGGAUGACUUGCCGACUCUCUACUUAGCCAUCUGCCUUUCUCCAAGGAGGCUUGCAGUACUGAUGAAGCCGGCAAGUUGUCUUUGGCUACAUGCUUCUUUCUG
            CAGCCAAGGAUGACUUGCCGACUC         1
             AGCCAAGGAUGACUUGCCGACUCUC       1
            Cagccaaggaugacuugccga&           0

>vvi-MIR169h [sc:118 - 423404 423504] [chr:11_random - 1171573 1171673]
.((((..((..(((.((((((((((((..((((....(((.((((.((((....)))).)))).))).))))..))))))))))))).)))..)).)))).....  (-34.90)
AGGGUGGAAUUGAGCCAAGGAUGGCUUGCCGUCCUUUGUCACUAUUUGAGGCAUUAACUGGUCACGCACGGAGGGUUAUCCUUGACUCCUUUAGCUCCUCU
          ugagccaaggauggcuugccg&             0

>vvi-MIR169i [sc:118 - 417231 417335] [chr:11_random - 1165400 1165504]
.((((..((..(((.((.(((((((..((((((((...((((((((...........))))))))..))).))))))..))))))).)).)))..)).))))....              (-40.60)
AGGGUGGAAUUGAGCCAAGGAUGACUGGCCGUCAUUUUUCAGUUGGUAUUUGAGGCUUUAACUGGUCAUGCACGGCUGGUUAUCCCUGUCUCCUUUAGCUCCUCU
           Gagccaaggaugacuggccgu&            0

>vvi-MIR169j [sc:118 - 472655 472776] [chr:11_random - 1220824 1220945]
(((((((((.(.(((((((((..((((((((((....(((........((((((.(((((.........)))))))).))).)))...))))))))))..))))))))).).))))))))))..           (-63.90)
GAGAGUGGAGUGCAGCCAAGGAUGACUUGCCGGAAUUCACAUAUAGAGUGGAAUGAGGCAAUAGACCGGCCUCUUCUCAUGGUGUCCCUGGCAGGUUGUCCUUGGCUACCUUUCGCUCUCUU
            Cagccaaggaugacuugccgg&           0

>vvi-MIR169k [sc:118 - 466033 466154] [chr:11_random - 1214202 1214323]
(((((((((.(.(((((((((..((((((((((..(((((........)))))....((.((((((....)))))).))..........))))))))))..))))))))).).))))))))))..           (-61.10)
GAGAGUGGAGUGCAGCCAAGGAUGACUUGCCGGAAUUCACAUAUAGAGUGGAAUGAGGCAAGAGGCCGGCCUCUUCCCAUGGUGUCCCUGGCAGGUUGUCCUUGGCUACCUUUCGUUCUCUU
            Cagccaaggaugacuugccgg&           0

>vvi-MIR169l [sc:118 - 389957 390059] [chr:11_random - 1138126 1138228]
.((((.((((.(((((((((((((((.(((((.(..(((......)))..)..((((......))))....))))).)))))))))))))))))))).)))).....(-50.10)
AGGGUGGAAUUGAGCCAAGGAUGACUUGCCGUCCUUUGCAUCAAGCAUUGAAGCUUUAACUGGGCAUGCACGGCUAGUUAUUCUUGGCUCAUUUGGCCCCUCU
          UGAGCCAAGGAUGACUUG__               3
           Gagccaaggaugacuugccgu&            0
```

```
>vvi-MIR169m [sc:118 - 214491 214592] [chr:11_random - 962660 962761]
((.((((((..(((((..(((((.((((((((..((....))..(((....((......)).)))....)))))))))))))..)))))..)))))).)).. (-46.20)
AGAGUGGAAGUGAGCCAAGGAUGACUUGCCGGCAGCUGCAGCAAGGCAUUAAGGUUUAACUGGCCAUAACUGGCAAGCAUCCGAGGCUCUGUUUCACCCUCU
          UGAGCCAAGGAUGACUUG  3
           Gagccaaggaugacuugccgg&           0

>vvi-MIR169n [sc:118 - 195487 195587] [chr:11_random - 943656 943756]
...((((((((((((..(((((.((((((.((....)).....(((.(((.......))))))......))))))))))..))))))))))))).....  (-45.00)
UGAGUGGAAUAGAGCCAAGGAUGACUUGCCGGCAACUGCAGCAAGGCAUAGGGUUUAACUGGUCAUAACUGGCAAGCAUCUGAGGCUCUAUUUCACCCUCU
           gagccaaggaugacuugccgg&           0

>vvi-MIR169o [sc:118 - 384917 385019] [chr:11_random - 1133086 1133188]
.((((.(...((((((((((((((((((.((((((..(((.((((......))))...))).).)))).).))))))))))))))))))..).)))).....(-51.70)
AGGGUGGAUUUGAGCCAAGGAUGACUUGCCGCCAUCAGCAGCAAGCAUUGAAGCUUUAACUGGGCAUGGACAGCGAGUUAUUCUUGGCUCAUGCGGCCCCUCU
          UGAGCCAAGGAUGACUUG                3
          UGAGCCAAGGAUGACUUGCCGCCAUC        1
           Gagccaaggaugacuugccgc&                          0

>vvi-MIR169p [sc:118 - 227917 228018] [chr:11_random - 976086 976187]
((.(((((((.(((((..(((((.((((((((..((....))..(((....((......)).)))....)))))))))))))..))))).))))))).)).. (-45.20)
AGUGUGGAAUUGAGCCAAGGAUGACUUGCCGGCAGCUGCAGCAAGGCAUUAAGGUUUAACUGGCUAUAACUGGCAAGCAUCCGAGGCUCUGUUUCACCCUCU
          UGAGCCAAGGAUGACUUG                3
           Gagccaaggaugacuugccgg&           0

>vvi-MIR169q [sc:118 - 191247 191343] [chr:11_random - 939416 939512]
((((..((((((((((.((((.((.(((((((..((.((((.(((....))).)))).))..))))))).)).)))))))))))))))..)))).. (-48.90)
AGGGUGGAAUAGAGCCAAGGAUGACUUGCCGGCAUUUGCAGUAAGUCUAUAUUAACUGGAACAGCCGGCAUGUAAUCCUGGCUCUAUUUGGUCCUCU
           gagccaaggaugacuugccgg&           0

>vvi-MIR169r [sc:118 - 160588 160696] [chr:11_random - 908757 908865]
(((((.(((((((((((((...(((((((((.(((((((..((.((((((........)))))).)).)))))))..)))))))))..))))))))))))).))))).. (-56.10)
AGGGUGGAAUUGAGUCAAGGAUGACUUGCCGAUAUAUAUUUGCAGAAGGCAUGCAGGGGCUUUUAGCUAUGUGUAACCGGCAAGUUGACUUGACUCAGUUUGGCCCUCU
          ugagucaaggaugacuugccg&            0

>vvi-MIR169s [sc:118 - 137852 137950] [chr:11_random - 886021 886119]
((.((..((.((.(((((.(((..(((((((((......((.....))......((.......))))))))))))..))).))))).)).)).)).)).. (-40.50)
GAAAGUGGAUUGCAGCCAAGGAUGACUUGCCGGCACUUGGCAUUAGGCACCAUCGAUCAUCUUCCCGGCAAGUUGUUCCUGGCUACAUUCUGCUCUCUU
            cagccaaggaugacuugccgg&          0

>vvi-MIR169t [sc:118 - 176151 176260] [chr:11_random - 924320 924429]
(((((.((((.((((((((...(((((((((.(((((((..((.(((((.(((....)))))))).)).)))))))..)))))))))..)))))))).)))).))))).. (-53.10)
AGGGUGGAAUCGAGUCAAGGAUGACUUGCCGAUAUAUAUUUGCGGAAGGACUUGCAUGGGCCUUUAGCUAUGUGUAACCGGCAAGUUGACUUGACUCAGUUUGGCCCUCU
          cgagucaaggaugacuugccg&            0

>vvi-MIR169u [sc:118 - 166317 166426] [chr:11_random - 914486 914595]
(((((.((((.((((((((...(((((((((..((((((..((.(((((.(.......).))))).)).))))))...)))))))))..)))))))).)))).))))).. (-52.80)
AGGGUGGAAUUGAGUCAAGGAUGACUUGCCGUUAUAUAUUUGCAGAAGGGCACGCAGGGGCCUUUAGCUAUGUGUUACCGGCAAGUUGACUUGACUCUGUUUGGCCCUCU
          ugagucaaggaugacuugccg&            0

>vvi-MIR169v [sc:118 - 109284 109379] [chr:11_random - 857453 857548]
((((((.((((.((((((((((((((((((((((.((((((((.....)))))...))).).))))))))))))).))))))))).))))...)))))) (-42.20)
AGGGAGGAACAAAGCCAAGGAUGAAUUGCCGGCGAUGAAAGUAGGCAGCUUUGUCCAUGGAUCGGCAAUUUAUUUCUUGGCUAUGUUGGGCUCUCU
           aagccaaggaugaauugccgg&           0

>vvi-MIR169w [sc:21 + 3743273 3743415] [chr:14 + 18908675 18908817]
(((...(..(((.(((.(((((((((((((((((....((.(((.(((((((.(((.((((((((((........).)))))))))))))...))).))))))).))..)))))))))))))))))).))).))).)..).))).... (-57.00)
GGUUAUGUGGUGCAGCCAAGGAUGACUUGCCGGCAACUCCCUUUAUUGUACUCAUGCAUGCUCAUACUUAUACUGUUGUGGGCAUCAUUUAAGUGGCAAAAAUGGUGGUCGGCGAGUCAUUCUUAGCUACAUUUCUGCCUCAU
            cagccaaggaugacuugccgg&          0

>vvi-MIR169x [sc:16 + 4993946 4994070] [chr:17 - 351748 351872
(((..(((...((((((((((((((.(((((((.(((((((((((....))).))))..(((.........)))..........)))).)))))))))))).))))).))))).....))).))).. (-42.70)
GUCUCGUCUGGUAGCCAAGGAUGACUUGCCUAAAUCCACCAGGUUUCAAAACACUGAAUGUAAUUAUCCACAAAGCGAUUCCGUGGUCUUUAGGCAGUCAUUCCUUCGGCUAAACUGACCGGCUC
          uagccaaggaugacuugccua&            0

>vvi-MIR169y [sc:180 - 169908 170155] [chr:1 + 14661042 14661289]
.((..((.(((.((.(((((.((((((((...((((.(.((((((((........((((((((((...(((.((((....)))).))).................(((.......))).((((........((((((.....))))))..........)))).((((((....))))))....)))).....))))))))))))).))).).))))...))))))))))))).))))).)).).))... (-73.77)
CGUUGUUUGGUAGCGAAGGAUGACUUGCCUACAGCCUCCUUAAGGUUUCAAAAUACCGAGCUUGUCUACCUUUGAUCAAAUGAUUCAAGAAACAAAAGAAAAGAAAGGCCAUGGCAGCCAUCCAUCAGAGUCAACAUGAGAAUCAUGUUCUAUAGAAAUUGGAGUCAUUCUUGAGAAUGAACACGACACUAUUAGCUUGGAAUCUAUAAUGUGGUUUAUGGCAAGUCUCCUUGGCACCUGAUGGCCUC
          uagcgaaggaugacuugccua&            0

>vvi-MIR171a [sc:9 + 461317 461415] [chr:14 - 14693988 14694086]
..(((...(((((((((((((.((((((((((((....((((..((((.....))))..))))....)))))))))))).)))))))))))))...)))    (-47.80)
UGGUGGGAAGCGUGAUGUUGGGACGGCUCAAUCAAACCAAAGAUCUCAAUGGUUGAGUCCUUUAAUCUGAUUGAGCCGUGCCAAUAUCAUGUUCAAUAC
                                                                   UGAUUGAGCCGUGCCAAUA                22
                                                                   UGAUUGAGCCGUGCCAAUAUCAU            7
                                                                   UGAUUGAGCCGUGCCAAUAUC &            3
                                                                   UGAUUGAGCCGUGCCAAUAUCAUa           2
                                                                   UGAUUGAGCCGUGCCAAUAUCA             1
                                                                      uugagccgugccaauaucaug           0

>vvi-MIR171b [sc:36 - 591211 591309] [chr:12 + 7277319 7277417]
(..(((((((((((((((((.(((((((.((..((((((((((((......)).))))))))))..)).))))))).)))))))))))))))))..)..    (-46.90)
UGGGGGGAGGUAUUGGCGUGCCUCAAUUUAAAGACAUGGUUAGAUGAAGAGAUAUUAGCCAUGUGAUUUGAUUGAGCCGCGUCAAUAUCUCCUUUUGCA
                                                                    ugauugagccgcgucaauauc&            0

>vvi-MIR171c [sc:36 - 536559 536661] [chr:12 + 7331967 7332069]
.((((.((((.((((((((..(((((((((.(((((.(((.(((((.((....))))))).))).))))).)))))))))..)))))))).))))....))))(-48.00)
UGGUAUUACGGAUAUUGGUGCGGUUCAAUAAGAAAGCAGUGCUCGCAAUCUUUUUGAGUUCUGGUUUUUGAUUGAGCCGUGCCAAUAUCACGUGUCAUUGCU
                                                                     UGAUUGAGCCGUGCCAAUA              22
                                                                     UGAUUGAGCCGUGCCAAUAUC&           3
                                                                     UGAUUGAGCCGUGCCAAUAUCa           1
                                                                        uugagccgugccaauaucacg         0

vvi-MIR171d [sc:795 - 2155 2254] [scaffold not incorporated in genome assembly]
.......(((.(((((((.(((((((((((((((.(((.(.(((((......))))).).))).))))))))))))))).))))))).)))......... (-38.60)
UAGAUACACGAGAUAUUGAUACGGUUCAAUUAGAAAGCUGUGUUCUAAGUUAAGAACUCUGUUGUUUGAUUGAGCCGUGCCAAUAUCACGUCCUACUUCU
                                                                  UGAUUGAGCCGUGCCAAUA                   22
                                                                  UGAUUGAGCCGUGCCAAUAUC&                3
                                                               UGAUUGAGCCGUGCCAAUAUCA        1
                                                                     UUGAGCCGUGCCAAUAUCACG    0
```

```
>vvi-MIR171e [sc:13 + 5162582 5162684] [chr:11 + 5162582 5162684]
((((((....(((((((((.((((((((.((.((((..((((((......))))))...)).)))).))))))))).)))))))))))))))))......(-44.70)
GGAAAGUAAGCGAUGUUGGUGAGGUUCAAUCCGAAGACGGAUUUACGAAGAGCCGUAAAGAACGAUCUCUGAUUGAGCCGCGCCAAUAUCACUUUCUGCUAAG
                                                                                 ugauugagccgcgccaauauc&             0

>vvi-MIR171f [sc:104 - 699397 699499] [chr:9 + 7000493 7000595]
.....((((.(((((((((.(((((((((((.((((..(((.(((......))).)))..)))))))))))))))))).)))))))))).))))).......(-45.90)
UUGAAGAAAGCGAUGUUGGUGAGGUUCAAUCUGAAGAUUGAUUUAUGCUUGAAUGUAAAAACUGAUCUCAGAUUGAGCCGCGCCAAUAUCACUUUUCUGUGGC
                                                                                    uugagccgcgccaauaucacu&          0

>vvi-MIR171g [sc:1 - 10508015 10508090] [chr:18 + 3212869 3212944]
....(.(((...(((((((((..((((.((((........))))))))...))))))))))).....))).)....             (-21.70)
GCCAGCCUCUCCAUGUUGGUUCCAUCGGUGGGGGACACCAACUCCUUGAGCCGAACCAAUAUCACCCGAGCCAAAU
                                                       uugagccgaaccaauaucacc&       0

>vvi-MIR171h [sc:16 + 3531871 3531966] [chr:17 - 1813852 1813947]
....(((.(((((((((((((((((((.(((..(((.(((.(((....))).))).)))..))).)))))))))))))))))))))).))).......        (-47.30)
UUUCACGAGAUGUUGGUGCGGUUCAACCUAACUGUAGUGUCCAUGAAUGUAUCGACUCUGAUGUUUGGUUGAGCCGCGCCAAUAUCCCGUCUAUCU
                                                                          ugguugagccgcgccaauauc&  0

>vvi-MIR171i [sc:16 - 4463645 4463747] [chr:17 + 882071 882173]
...(((.(((..((((((((.(((((((((((.((.(((...((((((...))))))...))).)).))))))))))))).)))))))).)).))).))).....(-44.90)
GAAGAAGGUGGGGAUGUUGGAAUGGCUCAAUCAAAUCAAAUCUCCCAAGGGUUUUGGGUCCUUUAAUCUGAUUGAGCCGUGCCAAUAUCAUCAUGUUCACUUC
                                                                                UGAUUGAGCCGUGCCAAUAUCAUC            2363
                                                                                UGAUUGAGCCGUGCCAAUA                 22
                                                                                UGAUUGAGCCGUGCCAAUAUCAU             7
                                                                                 GAUUGAGCCGUGCCAAUAUCAUC            3
                                                                                UGAUUGAGCCGUGCCAAUAUC&              3
                                                                                     GAGCCGUGCCAAUAUCAUC            3
                                                                                UGAUUGAGCCGUGCCAAUAUCAUa            2
                                                                                   uugagccgugccaauaucauc            1
                                                                                UGAUUGAGCCGUGCCAAUAUCA              1
                                                                                    UGAGCCGUGCCAAUAUCAUC            1

>vvi-MIR172a [sc:25 + 2163229 2163338] [chr:6: - 21821956 21822065]
....((((.((((((((((((((((((((.(((((....))..((..(.(((...))).)..))............))).))))))))))))))))))))).))))).. (-47.20)
UUGUUUGCGCAUGUAGCAUCAUCAAGAUUCACAUGCAAAUGCACGGUGGUGAUGUUAUCUCUGCCAAACUUUUCAAGGAUGAGAAUCUUGAUGAUGCUGCAUCGGCAAUA
                                                                                         UGAGAAUCUUGAUGAUGCUGCAUC                4

>vvi-MIR172b [sc:17 + 1425044 1425161] [chr:13 - 6323135 6323252]
....((((.((((((((((((((((((((((....((....)).((((.(((((((((.........)))))))).((.....))).)))).)))))))))))))))))))))).))))).. (-65.26)
UUGUUUGCAGGUGUAGCAUCAUCAAGAUUCACAUGCGAAAGCCCGGCGCGGCAGCGAUGCCAUCUUCGCUGCCUCAAGUUUUGGGGUUGAGAAUCUUGAUGAUGCUGCAUCGGCAAUA
                                                                                                UGAGAAUCUUGAUGAUGCUGCAUC        4

>vvi-MIR172c [sc:17 - 4437525 4437662] [chr:13 - 3310634 3310771]
..((((.(.((.((((((((((((((((.((.((...((((..((...((..(((..((.((.((((.....)))))).))...)))..)).)).))..)))).)).)).)))))))))))))))).)).).)))).... (-57.90)
GUUUGCGGAUGGAGCAUCAUCAAGAUUCACAAGUAUUGAGACUCAGUGCGUGGUGGUGAUGGUGACUUUUGUGGUCCCUUCCUACACUCCGAUGGCUCUUUGAUGUGGGAAUCUUGAUGAUGCUGCAGCGGCAAUAAA
                                                                                                                    ggaaucuugaugaugcugcag&    0

>vvi-MIR172d [sc:29 - 1692538 1692646] [chr:8 - 11559580 11559688]
....((.((((((((((((((((((((((.((.((((......(((((.(((...))).))))).......))).).)).).))))))))))))))))))))).)).......  (-52.22)
GUUAGCUGAUGCAGCAUCAUCAAGAUUCACACCCAAUGGAAGGGCAGUGAUGCAAUCUCUGCCAAAGAUUUUGAGAUGAGAAUCUUGAUGAUGCUGCAUUCGCAGUGAA
                                                                                  agaaucuugaugaugcugcau&    0

>vvi-MIR319b [sc:5 + 4170832 4171025] [chr:1 + 4170832 4171025]
.((((((...(((((((((((((((((((..((.((((((((....(((((.(((..(((.(((..((((((..(((((((..((((.(((..............)))))))..))))))))..)))))))..))).))).)..))))))))))..))))))).)).)).))))))))))))))))))))......)))))).          (-102.82)
UGUGGAAGAAGGAGCUCUCUUUAGUCCAGUCCGAGACAGCUUCAAGGCUGAUACGUGGCUGCUGACUCGUUGCUUCAAGAACUCAUCACAUAGGCCUGGAAAAGAUGGAUGGUUUUUUGAUCCAACGAUGCAGGAGCUGCGUUCAGUUAUGGCUGUCACGUCUUGGACUGAAGGGAGCUCCCUUGUAUUCCACA
                                                                                                                                                   UUGGACUGAAGGGAGCUCCCU&        1
                                                                                                                                                  cuuggacugaagggagcuccc          0

>vvi-MIR319c [sc:132 + 439049 439230] [chr:2 - 661750 661931]
...((.(((((.(((((..((((((((((..((((((..((..(((((((.((((.(((.(((..(((((((..((..(((((.((.........)).)))))..))..)))))))..))).)).).)))).))))))).))..)))))))..))))))))))..))))).))))).))..... (-83.40)
UUACAUUGAAGAGAGCUUUCUUCAGUCCACUCAUGGGUGGCAGUAGGAUUGAAUUAGCUGCCGACUCAUUCAUCCAAAUACUGUGUUAAGGACUACCCAGUAAAUGAUUGAAUGAUGCGGGAGACAAAUUGGAUCUUAAGCUCCCUGUGCUUGGACUGAAGGGAGCUCCCUUCACUGCAAUC
                                                                                                                                      UUGGACUGAAGGGAGCUCCCUUC     3
                                                                                                                                      UUGGACUGAAGGGAGCUCCCU&      1
                                                                                                                                     cuuggacugaagggagcuccc        0

>vvi-MIR319e [sc:13 + 4274222 4274328] [chr:11 + 4274222 4274328]
.(.((((.(((((((((((((.(((.(((((((((.......((((((...........))))).))...))))))))).)))))))))))))))).)))).)...            (-40.40)
UGCAGAAAUGGGGGUUCCUUUGCAGCCCAAAACAACUCCAUCGCUGAAGAAGAUGAUGAACUUCAUGCUCCUUGUUUUGGACUGAAGGGAGCUCCUAGUUCUUCUCU
                                                                                      uuuggacugaagggagcuccu&        0

>vvi-MIR319f [sc:3 - 409791 409983] [chr:6 + 9069150 9069342]
((((...(((.(((((((((((((((((..((((((..((..((((((..((.(((.((.(((..((((((((.((..((((((((....(((......))).))))))))..)).))))))))..))).)).))).)).))..)))))))..))..))))))..))))))))))))))))).))))))))).....              (-88.70)
AGUGGUUUAAGAGAGCUUCCUUCAGUCCACUCAUGGAUGGGUUAGGGGUUUGGAUUAGCUGCCGACUCAUUCAUUCAAACACAGUAGAAUGGAAAUGGAGUUUACUACUGUGAAUGUGUGAAUGAUGCGGGAGGUAAAUUUCAUCCUUUUCUUGUCUGUGCUUGGACUGAAGGGAGCUCCCUUCUACUGUUUU
                                                                                                                                                  UUGGACUGAAGGGAGCUCCCUUC        3
                                                                                                                                                  UUGGACUGAAGGGAGCUCCCU&         1
                                                                                                                                                 cuuggacugaagggagcuccc           0

>vvi-MIR319g [sc:16 + 1677842 1678063] [chr:17 - 3667755 3667976]
.....(((...((((((((((((((((((..((.((((.(((.(((((..((.(((.((((.(((((((...(((((.(((((.((.((((.((..(((((((...))))).))..)).)))).)).......))))).))))).)))...)))))))).)))).))).)..))...)))).).))).)))).)).))))))))))))))))))....)))......                (-96.00)
GAACAGCUGAAGAGCUCCUUUUAGUCCAAUAAAGAGGGCUGAGGGGCGGCCAGAGCUGCCAUCUCAUGCACUUGGCUAUGCUUAUUCUUUUCUCAAUCAAUUCGGUUCGAGUAUGGCUGUGGAGUGACAAAUGCUAAGUUUAGCCUGAUGCAUGACAUGGGAGCAACUCCUCCGCACGCUUUGCCCACUCAUUGGACUGAAGGGAGCUCCCAGGGCAGAUCU
                                                                                                                                                                                       UUGGACUGAAGGGAGCUCCCA&        2
                                                                                                                                                                                      auuggacugaagggagcuccc          0

>vvi-MIR390 [sc:3 - 1385305 1385444] [chr:6 + 8093689 8093828]
((((((((.(((((((((.((((((((((((.((.((((((...(((....(((((((((....)))).))))))....)))..)))))))...))...))))))))))))).))))))))))).))))))))).. (-62.30)
AGAAUCUGUUAAGCUCAGGAGGGAUAGCGCCAUGAGCCAUGAUGAAAAUGAUUGUUGAGUUGAAAAUUUUUUCUAGCUUGUUUUUUGGGUCAUCAUUUUGUGUGUGUGGCGCUAUCUAUCCUGAGUUUCACGGGUUCUUC
        aagcucaggagggauagcgcc&                         1

>vvi-MIR393a [sc:222 - 104163 104288] [chr:Un_random - 54807498 54807623]
(((((.(((((.(((((((.((((.(((((.((((..(.................(((((((...........)))))))......)..)))).))))))))).))))))).)))))).))))))..       (-53.90)
GGUGGACAGUUCCAAAGGGAUCGCAUUGAUCCCAUGGUUAUCUUCCACCUCUCUUACCAGACUAUCAUAACAAAGUCUGGCCUUUUUCCCAUUGGAUCAUGCUAUCCCUUAGGAACUCUCCAUCAG
        UCCAAAGGGAUCGCAUUGAUCa&                         1
        uccaaagggaucgcauugaucc                          0

>vvi-MIR393b [sc:17 - 3375224 3375306] [chr:13 + 4372990 4373072]
((((((((.(((((((((((.((((.(((((.(((..(.....)..))))))))))))).))))))))))))).))))))))..      (-45.70)
GGAGGAGGCAUCCAAAGGGAUCGCAUUGAUCCCAAACUCUCCAUGUCUUUGGAUCAUGCUAUCCCUUUGGAUUCCUCCUUUGG
        UCCAAAGGAUCGCAUUGAUCa&                          1
        uccaaagggaucgcauugaucc                          0
```

```
>vvi-MIR394a [sc:38 + 1925563 1925651] [chr:12 - 14925943 14926031]
((((((((((((((((.(((.((((((((((.......................))))))))).))).)))))))))))))))))..               (-51.01)
CAGAGCCAUUUUGGCAUUCUGUCCACCUCCAUGCACAUCAUGGGUCUAUAUUCUUUUGGAGGUGGCCAGCAUGCCAAAGUGGCUCUGUA
          UUGGCAUUCUGUCCACCUCCAU&            1
          uuggcauucuguccaccucc               0

>vvi-MIR394b [sc:1 + 12335062 12335154] [chr:18 - 1385805 1385897]
((((((((.((((((.(.((((((((((((((......(((((......)))))....)).))))))))))).).))))))..))))))))).           (-42.60)
ACAGAGUUUAUUGGCAUUCUGUCCACCUCCCAUCUCUUGAAAAUCUCUCUUUUCUCUCUGUGGAGGUGGGCAUACUGCCAACCAAGCUCUGUU
          uuggcauucuguccaccucc&              0

>vvi-MIR394c [sc:1 + 10217177 10217278] [chr:18 - 3503681 3503782]
(((((((.(((((((((((((.((.((((((......(((((((....)))))))(((......)))..)))))).)).)))))))))))))).))))))))..  (-59.50)
CAGAGCCAUUUUGGCAUUCUGUCCACCUCCAUAUAUACCAAUUCUUCUGAAUUGGCGCCCACAAGCGUUUGGAGGCGGCCAGGAUGCCAAAUUGGCUCUGUA
          UUGGCAUUCUGUCCACCUCCAU&            1
          UUGGCAUUCUGUCCACCUCCAUa            1
          uuggcauucuguccaccucc               0

>vvi-MIR395a [sc:5 + 6669509 6669600] [chr:1 + 6669509 6669600]
....((..(((((((((..(.(((((((.((((((((...............).))))))).))))))).)..)))))))))..))......           (-40.86)
GUCCCCUAGAGUUCCCUUGAUCACUUCACUAGGGAGCUUCGCUAGUUUUAAUGACUUCCUACUGAAGUGUUUGGGGGAACUCCUGGUACCGA
                                                            cugaaguguuugggggaacuc&     0

>vvi-MIR395b [sc:5 + 6643441 6643530] [chr:1 + 6643441 6643530]
....((..((((((((((...(((((((...(((...(((......)))..)))......)))))))...))))))))))..))......              (-34.90)
GUCCCCUAGAGUUCCCCUUACCACUUCACUGGGGAUCUUCUCUAAUGAAUGCCUACGUACUGAAGUGUUUGGGGGAACUCCUGGUGCCAU
                                                          cugaaguguuugggggaacuc&       0

>vvi-MIR395c [sc:5 - 6640691 6640782] [chr:1 - 6640691 6640782]
....((..(((((((((..(.(((((((.((((((...((..((....))..)).)))))).))))))).)..)))))))))..))......           (-37.80)
GUCCCCUAGAGUUCCCUUGACCACUUCACUGGGGACCUUCUCUAGUUAUAAUGACUUCCUACUGAAGUGUUUGGGGGAACUCCUGGUGCCAU
                                                            cugaaguguuugggggaacuc&     0

>vvi-MIR395d [sc:5 + 6653540 6653625] [chr:1 + 6653540 6653625]
....((..(((((((((..(.(((((((.((((((((.........)).)))))).))))))).)..)))))))))..))......  (-40.80)
GCCCCCUAGAGUUCCCCUGACCACUUCAUUGGGGAUCUUCUCUAAUGACUUCCUACUGAAGUGUUUGGGGGAACUCCUGGUGUCAU
                                                      cugaaguguuugggggaacuc&           0

>vvi-MIR395e [sc:5 + 6646025 6646116] [chr:1 + 6646025 6646116]
....((..(((((((((..(.(((((((.((((((...((............)).)))))).))))))).)..)))))))))..))......           (-37.30)
CUCCCCUAGAGUUCCCUUGACCACUUCACUGGGGACCUUCUCUAAUUAUAAUGACUUCCUACUGAAGUGUUUGGGGGAACUCCUGGUGCCAU
                                                            cugaaguguuugggggaacuc&     0

>vvi-MIR395f [sc:5 + 6630319 6630404] [chr:1 + 6630319 6630404]
(.((((..(((((((((..(.(((((((.((((((((.........)).)))))).))))))).)..)))))))))..)).))).. (-44.20)
GUACCCUAGAGUUCCCCUGACCACUUCACUGGGGAUCUUCUCUAAUGACUUCCCACUGAAGUGUUUGGGGGAACUCCUGGUGUCAU
                                                      Cugaaguguuugggggaacuc&           0

>vvi-MIR395g [sc:5 + 6622890 6622975] [chr:1 + 6622890 6622975]
(.(.((..(((((((((..(((((((((.(((((((............))))))).)))))))))..)))))))))..)).).).. (-44.40)
GUCCCCUAGAGUUCCCCUGAGCACUUCAUUGGGGAUCCUUAGUUUUCAAUUCCUACUGAAGUGUUUGGGGGAACUCCCGGUGUCAU
                                                      cugaaguguuugggggaacuc&           0

>vvi-MIR395h [sc:5 + 6706656 6706747] [chr:1 + 6706656 6706747]
(..((...(((((((((..(.(((((((.((((((...((............)).)))))).))))))).)..)))))))))..))..)...           (-37.50)
GUCCCCUAGAGUUCCCUUGACCACUUCACUGGGGACCUUCUCUAAUUAUAAUGACUUCCUACUGAAGUGUUUGGGGGAACUCCUGGUGCUAU
                                                            cugaaguguuugggggaacuc&     0

>vvi-MIR395i [sc:5 + 6702646 6702731] [chr:1 + 6702646 6702731]
....((..(((((((((..(.(((((((.((((((((.........)).)))))).))))))).)..)))))))))..))......  (-40.80)
GCCCCCUAGAGUUCCCCUGACCACUUCACUGGGGAUCUUCUGUAAUGACUUCCUACUGAAGUGUUUGGGGGAACUCCUGGUGUCAU
                                                      cugaaguguuugggggaacuc&           0

>vvi-MIR395j [sc:5 + 6693030 6693115] [chr:1 + 6693030 6693115]
....((..(((((((((..(.(((((((.((((((((.........)).)))))).))))))).)..)))))))))..))......  (-40.80)
GCCCCCUAGAGUUCCCCUGACCACUUCACUGGGGAUCUUCUUUAAUGACUUCCUACUGAAGUGUUUGGGGGAACUCCUGGUGUCAU
                                                      cugaaguguuugggggaacuc&           0

>vvi-MIR395k [sc:5 + 6678361 6678452] [chr:1 + 6678361 6678452]
...((..(((((((((..(.(((((((.((((((...((............)).)))))).))))))).)..)))))))))..)).......           (-37.30)
UCCCCUAGAGUUCCCUUGACCACUUCACUGGGGACCUUCUCUAAUUAUAAUGACUUCCUACUGAAGUGUUUGGGGGAACUCCUGGUGCCAUU
                                                           cugaaguguuugggggaacuc&      0

>vvi-MIR395l [sc:5 + 6699102 6699188] [chr:1 + 6699102 6699188]
....((..(((((((((..(.(((((((.(((((..((.........))..))))).))))))).)..)))))))))..))...... (-41.10)
GCCCCCUAGAGUUCCCCUGACCACUUCACUGGGGGAUCUUCUGUAAUGACUUCCUACUGAAGUGUUUGGGGGAACUCCUGGUGUCAU
                                                       cugaaguguuugggggaacuc&          0

>vvi-MIR395m [sc:5 + 6697815 6697906] [chr:1 + 6697815 6697906]
....((..(((((((((..(((((((((.((((((...((..((....))..)).)))))).)))))))))..)))))))))..))......           (-42.00)
GUCCCCUAGAGUUCCCUUGAACACUUCACUGGGGACCUUCUCUAGUUAUAAUGACUUCCUACUGAAGUGUUUGGGGGAACUCCUGGUGCCAU
                                                            cugaaguguuugggggaacuc&     0

>vvi-MIR395n [sc:101 - 1067989 1068125] [chr:17 - 6465770 6465906]
(((.(..((((((((.(((..(.((.(((((......................(((((.....)))))....................((((.....)))).))))).)).)..))).))))))))..).)))..          (-37.00)
GGCCUUGAGAGUUCCCCCAACCCCUCCAGUAUGACCAUCUCUCUUCUUCUUUUAAUUUGUUCAUCAAAUCUUCUUCUACUAUCUAUUUCCAGGGUGAUUUCCUCUACUGAAGAGUCUGGAGGAACUCUUGGUGCCAC
                                                                                                           cugaagagucuggaggaacuc&     0

>vvi-MIR396a [sc:104 + 339535 339650] [chr:9 - 7360342 7360457]
...(((((((..((((((((((((((((((.((.((((((((.((.(((.....((((......)))).))))))).)))).)).)).)))))))))))))))))))..))))))).  (-51.30)
UCCUGUCAUGCUUUUCCACAGCUUUCUUGAACUACUUUUCCAUCUACCUGUUUGAUGAUCUUCUUCAUAAGGAGUGUGAAAUAGAAGCUCAAGAAAGCUGUGGGAGGACAUGGCAA
             uuccacagcuuucuugaacua&          0

>vvi-MIR396b [sc:13 + 5206054 5206161] [chr:11 + 5206054 5206161]
....((((((.((((((((((((((((((((((((...........(((((.................)))))))))))))))))))))))))))))).)))))).. (-52.67)
UCCUGCCAUGCUUUUCCACAGCUUUCUUGAACUUCUUCUCCCUGUUUGGUGGUCUCCUACGUUUACCCAUCCACUGAAGUUCAAGAAAGCUGUGGGAAACCAUGGCUU
                                                                        GUUCAAGAAAGCUGUGGGAAA     2
             Uuccacagcuuucuugaacu&           0

>vvi-MIR396c [sc:6 - 5105253 5105360] [chr:4 - 5114321 5114428]
(((.(((((.((((((((((((((((((.((((((((((..(((.(((...........))))))..)))))..))))).)))))))))))))))))).))))).))).. (-48.00)
UUCUGUGAUCUUCCACAGCUUUCUUGAACUGUUUUGGAAGCCUUCCACUUCCAUGCAAUGGAGGAGUUCAACCAACAGCUCAGGAAAGUUGUGGAACAUUACUGAGAG
          uuccacagcuuucuugaacug&             0

>vvi-MIR396d [sc:13 - 5212374 5212493] [chr:11 - 5212374 5212493]
....((((..((((((((((((.((((((((((.....((((((...(((((((......)))))))...))))..))........))))))))).)))))))))))))..))))......              (-47.80)
CUUUGUAUUCUUCCACAGCUUUCUUGAACUGCAUUUUUCAGACUACUGGUGUGUUUUUGUAUGCACUCAAAGUCAUUGUAACAGUUGCGGUUCAAUAAAGCUGUGGGAAGAUACCAACAG
          uuccacagcuuucuugaacug&             0
```

```
>vvi-MIR397a [sc:448 + 59654 59771] [chr:Un_random + 85971396 85971513]
.......((((.((((((((((((((..(((.((((..((...((((((((.(.(((((...)))))...).))))))))..)))))).....)))..))))))))))))))).)))).                (-46.40)
GAAGAAAACAUCAUUGAGUGCAGCGUUGAUGAAACUGAAGUAUUCCAUUUUUCAGCUUCUUUGAAGUCCGGCAAGAUGGGUUCUGGUUGAUUCCAUUGGCGCUGCACUCAAUCAUGUC
          UCAUUGAGUGCAGCGUUGAUGA&           2
                                                                                           AUUCCAUUGGCGCUGCACUCAAUC     2
          ucauugagugcagcguugaug             0

>vvi-MIR397b [sc:1477 - 246 363] [chr:Un_random - 146568042 146568159]
.......((((.((((((((((((((..(((.((((..((...((((((((.(.(((((...)))))...).))))))))..)))))).....)))..))))))))))))))).)))).                (-46.40)
GAAGAAAACAUCAUUGAGUGCAGCGUUGAUGAAACUGAAGUAUUCCAUUUUUCAGCUUCUUUGAAGUCCGGCAAGAUGGGUUCUGGUUGAUUCCAUUGGCGCUGCACUCAAUCAUGUC
                                                                                           AUUCCAUUGGCGCUGCACUCAAUC     2
          UCAUUGAGUGCAGCGUUGAUGA&           2
          ucauugagugcagcguugaug             0

>vvi-MIR398a [sc:5 + 717225 717327] [chr:1 + 717225 717327]
...((((((((.(((..(((((((((((..(.((((((((((.((.........)))))))))))).)..))))))))))))))).)))))))).....(-55.60)
ACACCCCAAGGGAGUGGCACCUGAGAACACCGGUUGUGUUGGCUGCGCCAAGCUCUGCUAGCUAAUGCAUUUUGUGUUCUCAGGUCACCCCUUUGGGGCACCA
                                                                               uguguucucaggucaccccuu&          0

>vvi-MIR398b [sc:25 + 3324549 3324636] [chr:6 - 20660658 20660745]
..((((..((((.(((((((((((.((((((...(((...........)))...))))))))))))))))).)))))..)))).... (-44.70)
GUGUCCUACAGGUGUGACCUGAGAAUCACAUGCCCGCACCACCCAUCUUUGCUCUCAUGUGUUCUCAGGUCGCCCCUGCUGGACUCCC
                                                         UGUGUUCUCAGGUCGCCCCUGa&         2
                                                         UGUGUUCUCAGGUCGCCCCUGCU         1
                                                         uguguucucaggucgccccug           0

>vvi-MIR398c [sc:15 - 78481 78568] [chr:6 + 14851882 14851969]
..((((..((((.(((((((((((.((((((...(((...........)))...))))))))))))))))).)))))..)))).... (-45.80)
GUGUCCUACAGGAGUGACCUGAGAAUCACAUGCCCGCAUCACCCAUCUUUGCUCUCAUGUGUUCUCAGGUCGCCCCUGCUGGACUCCC
                                                         UGUGUUCUCAGGUCGCCCCUGa&         2
                                                         UGUGUUCUCAGGUCGCCCCUGCU         1
          GGAGUGACCUGAGAAUC   1
                                                         uguguucucaggucgccccug           0

>vvi-MIR399a [sc:81 - 819715 819811] [chr:10 + 1853461 1853557]
..(((((((.(..((((((((((((((((......(((............)))......))))))))))))))))..).))))))))....      (-51.32)
GAGAAUAACAGUGUGAUUCUCCUUUGGCAGAGAUAUCACGCAUGGAGUAGAAGGCGGUAGCUGCUCUGCCAAAGGAGAAUUGCCCUGUUAUUCAAUU
                                                           ugccaaaggagaauugcccug& 0

>vvi-MIR399b [sc:376 + 84186 84302] [chr:Un_random + 77999653 77999769]
..((.(((((((((.(((((..((((((((((..((..(((..((((......))))...)))..))..........))).))))))))..))))).)))))))))).)).... (-48.22)
AUCAAUCAUAGGGCACCUCUUUCUUGGCAGGCACUGGCUCCUAUAUAUGAAUAUACAUAGCUUAGCUGCAGUAAAAAGAUGUGACUUGCCAAAGGAGAGUUGCCCUGUGACUGCUUC
                                                                                 ugccaaaggagaguugcccug&          0

>vvi-MIR399c [sc:37 - 1888940 1889021] [chr:15 + 5804593 5804674]
(((((.(.((((((..(((((..(((((((((.((((....)))..).))))))))))..)))))..)))))).).))))))..       (-39.40)
ACCGGUCUCAGGGCCUCUUUCACUUGGUAGGUGACAUAGAUGUGAAUGAUCUGCCAAAGGAGAGUUGCCCUGUGUCUGGUUA
                                                   ugccaaaggagaguugcccug& 0

>vvi-MIR399d [sc:81 + 821136 821240] [chr:10 - 1852032 1852136]
.((((((...(((((((((((((((((((((((((((.........)))...(..(((((......)))))..)))))))))))))))))))))))))...))))))...              (-49.20)
GUAAAUUAUAGAGCAGAUUUCUUUUGGCAGAUGGCGAUCACAAGCCAAUGUGCUCAAAGGGCAUUGAGUGUGUCUGCCAAAGGAGAUUUGCUCGUGAAUUUAACU
                                                                             ugccaaaggagauuugcucgu&        0

>vvi-MIR399e [sc:81 + 816930 817041] [chr:10 - 1856231 1856342]
..........((((((((.((((((((((((..((((((..((((....((.........))..))))..)))).))..))))))))))))))))))))............      (-42.10)
GCAUAUUACAGGGCAAAUUAUCUUUUGGCAGGCAGCCACUUAGAGACACAGCCAAGCCAUGCAUUCUUGUAGUGUGCCCUCUGCCAAAGGAGAUUUGCCCGGCAAUUCUUCU
                                                                                    ugccaaaggagauuugcccgg&0

>vvi-MIR399f [sc:81 + 813348 813454] [chr:10 - 1859818 1859924]
((((((.....((((.((((((.((((((((.((((...(((..((((((.........))))))))))))....)))))))).))))))))))))))))).......            (-38.60)
GUUGCAUUAGAGGGCCAAAUCUGCUUUGGCAUGAAGCCCAUUUAGCAGGCUCGUCUGUGUCUGCAGUCUUCUCGCUGCCGAAGGAGAUUUGUCCUGCAAUUCUUCUG
                                                                              ugccgaaggagauuuguccug&       0

>vvi-MIR399g [sc:81 + 827902 828014] [chr:10 - 1845258 1845370]
.(((((((..((((((..((((.((((((((.(((...(((((((....)))))))).......(((.....)))..))).))))))))).)))).)))))))..))))))))...      (-48.80)
AUGAAUUGCUGGGCAAUACUCCAUUGGCAGUUGGCCACUCGGCUGACCGCGGUUGACUUCACAAGUAGCAGACUAAGCUCACUGCCAAAGGAGAUUUGCCCCUCAAUUCAGCU
                                                                                    ugccaaaggagauuugcccu&              0

>vvi-MIR399h [sc:81 - 825627 825716] [chr:10 + 1847556 1847645]
..((((..(((.(((((.((((((((((((((((((..((((....))))...))).))))))))))))))).))))).)))..)))).....             (-41.10)
AGGAAUAACAGUGCAAUCCUCCUUUGGCAGAAAGAUCAUGCACAUGCAUACUUCUGUUUUGCCAAAGGAGAAUUGCCCUGCCAUUCGCUC
                                                          ugccaaaggagaauugcccug&         0

>vvi-MIR399i [sc:112 - 503240 503361] [chr:2 + 4040803 4040924]
((((((..((((((..((((((((.((((.(((((((((((.((((((......))))).)).))))))))((((((((.........))))).))).))))))))))))..)))))))..)))))))..           (-57.90)
AGUAGUUGUAGGGCUUCUCUCCUUCUGGCAGGAGAUGGCAAUAGAUUAUCCUUUGUGGCUUAUCUCCGGUGUUGAUUAACUUCCAAUGACCCGCCAAAGGAGAGUUGCCCUGUGACUACUUC
                                                                                            CGCCAAAGGAGAGUUGCCCUGa&     1
                                                                                            cgccaaaggagaguugcccug       0

>vvi-MIR403a [sc:64 - 1835262 1835376] [chr:5 - 1835262 1835376]
.((...(((((((((((((((((((.(((.((.(((.(..(((((.((((....))))).........)))))..)))).)).))).)))))))))))))))))..)))))...)).   (-48.30)
GCACAUCUCAAGUUUGUGCGUGAAUCAAACCCCAUCGUAGCCGUCCAUAUCUUCGGGUACUAAUCACGGGGCGAUUCGACGGCGUUAGAUUCACGCACAAACUCGUGAGUUCUGA
                                                                                     UUAGAUUCACGCACAAAC                20
          AGUUUGUGCGUGAAUCAAACCCCA           4
                                                                                     UUAGAUUCACGCACAAACUCGa&           4
             UUGUGCGUGAAUCAAACCCCA           1
                                                                                     UUAGAUUCACGCACAAAa                1
                                                                                     UUAGAUUCACGCACAAACUCGU            1
            UUUGUGCGUGAAUCAAACCCCA           1
            UUUGUGCGUGAAUCAAACCCCAUC         1
               GUGCGUGAAUCAAACCCCA           1
        CAAGUUUGUGCGUGAAUCAAACCCCA           1
                                                                                     uuagauucacgcacaaacucg             0

>vvi-MIR403b [sc:64 - 1304545 1304630] [chr:5 - 1304545 1304630]
......(((((((((((((.(((((.((((((((..............)))))))).))))).))))))))))))))........  (-43.44)
ACAAACCUCGAGUUUGUGCGCGAAUCCAACGCCUCGAUCUUCUUUCAAAGGGGUGUUAGAUUCACGCACAAACUCGGGAUCUGUCU
                                                      UUAGAUUCACGCACAAAC             20
                                                      UUAGAUUCACGCACAAACUCGa&        4
                                                      UUAGAUUCACGCACAAAa             1
                                                      uuagauucacgcacaaacucg          0

>vvi-MIR403c [sc:64 - 1302086 1302200] [chr:5 - 1302086 1302200]
(((.(((.(.((((((((((((((((.(((..(.(((.(..(((((.((((....)))).........)))))..)))).)..))).))))))))))))))))).).))).))).)..   (-45.20)
GCACAUCUCAAGUUUGUGCGUGAAUCAAACCCCAUCGUAGCCGUCCAUAUCUUCGGGUACUAAUCACGGGGCGAUUCGACGCCGUUAGAUUCACGCACAAACUCGUGAUCUGUCC
                                                                                     UUAGAUUCACGCACAAAC                20
          AGUUUGUGCGUGAAUCAAACCCCA           4
                                                                                     UUAGAUUCACGCACAAACUCGa&           4
```

```
               UUGUGCGUGAAUCAAACCCCA           1
                                                                                  UUAGAUUCACGCACAAAa                 1
                                                                                  UUAGAUUCACGCACAAACUCGU             1
              UUUGUGCGUGAAUCAAACCCCA           1
              UUUGUGCGUGAAUCAAACCCCAUC         1
                 GUGCGUGAAUCAAACCCCA           1
          CAAGUUUGUGCGUGAAUCAAACCCCA           1
                                                                                  uuagauucacgcacaaacucg              0

>vvi-MIR403d [sc:64 - 1740161 1740246] [chr:5 - 1740161 1740246]
......(((((((((((((.(((((..((((((..............))))))....))))).)))))))))))))........   (-40.24)
ACAAACCUCGAGUUUGUGCGCGAAUCCAACCCCUCAAUCUUCUUUCAAAGGGGUGUUAGAUUCACGCACAAACUCGGGAUCUGUCU
                                                       UUAGAUUCACGCACAAAC                20
                                                       UUAGAUUCACGCACAAACUCGa&           4
                                                       UUAGAUUCACGCACAAAa                1
                                                       uuagauucacgcacaaacucg             0

>vvi-MIR403e [sc:64 - 1738515 1738629] [chr:5 - 1738515 1738629]
(((.(((.(.((((((((((((((((.(((..(.(((.(..(((((.......(((.......)))))))))..)))).)..))).))))))))))))))))).).))).)))..   (-44.31)
GCACAUCUCAAGUUUGUGCGUGAAUCAAACCCCAUCGUAGCCGUCCAUAUCUUCGUGUACUAAUCACGGGGCGAUUCGACGCCGUUAGAUUCACGCACAAACUCGCGAUCUGUCC
                                                                                  UUAGAUUCACGCACAAAC                 20
            AGUUUGUGCGUGAAUCAAACCCCA           4
                                                                                  UUAGAUUCACGCACAAACUCGa&            4
               UUGUGCGUGAAUCAAACCCCA           1
                                                                                  UUAGAUUCACGCACAAAa                 1
              UUUGUGCGUGAAUCAAACCCCA           1
              UUUGUGCGUGAAUCAAACCCCAUC         1
                 GUGCGUGAAUCAAACCCCA           1
          CAAGUUUGUGCGUGAAUCAAACCCCA           1
                                                                                  uuagauucacgcacaaacucg              0

>vvi-MIR403f [sc:42 - 1340023 1340130] [chr:7 - 4539818 4539925]
..((((.(.((((((((((((((.(((((...(((...(((..............))))))..(((........))).))))).))))))))))))))).).)))).....            (-36.92)
UGAGAUGAGGAGUUUGUGCGUGACUCUAAAACCGCCUGAUCUAAACCUUUUCCCGAUGCGAUACCUUUUCUAUGGUCUUAGAUUCACGCACAAACUCGUAAUCUGUCU
                                                                           UUAGAUUCACGCACAAAC         20
                                                                           UUAGAUUCACGCACAAACUCGa&    4
                                                                           UUAGAUUCACGCACAAACUCGU     1
                                                                           UUAGAUUCACGCACAAAa         1
                                                                           uuagauucacgcacaaacucg      0

>vvi-MIR408 [sc:42 + 2173285 2173391] [chr:7 + 5373080 5373186]
.((((..((((((((.((((((((((((((.((((.(....(((((.............)))))...).)))).))))))))))))))).))))))..)))..)))).            (-51.90)
AAGAGGAAGACGGGGACGAGGUAGUGCAUGGAUGGAACUAUUAACAGAAGAAUGUUAAGCUGUUUUUGCUCUACCCAUGCACUGCCUCUUCCCUGGCUCUGUCUCUC
                                                                          AUGCACUGCCUCUUCCCUGGCUC&    38
          CGGGGACGAGGUAGUGCAUGGAU           19
          CGGGGACGAGGUAGUGCAUGGAUa          14
          CGGGGACGAGGUAGUGCAUGGAUG          8
          CGGGGACGAGGUAGUGCAUGGA            5
                                                                          AUGCACUGCCUCUUCCCUGGCa      5
                                                                           UGCACUGCCUCUUCCCUGGCUC     4
                                                                           UGCACUGCCUCUUCCCUGGCUa     4
                                                                           UGCACUGCCUCUUCCCUGGC       3
                                                                           UGCACUGCCUCUUCCCUGGCUCa    2
                                                                           UGCACUGCCUCUUCCCUGGCU      2
                                                                          augcacugccucuucccuggc       1
         ACGGGGACGAGGUAGUGCAUGa             1
                                                                          AUGCACUGCCUCUUCCC           1

>vvi-MIR477 [sc:166 + 518573 518666] [chr:1 + 15382675 15382768]
.(((((..(((..(((((((....((((((((((((.(.((.........)).).)))))))))))))....)))))))..)))..)))))...         (-40.30)
GAAGUUGUCAAUCUCCCUCAAAGGCUUCCAAUUUUCCUGGCUGAUUAUCAAGGUUGGAAAGUUGGAAGACAGUGGGGGACCUUGGAAGCUUACC
          aucucccucaaaggcuuccaa&            0

>vvi-MIR479 [sc:86 + 1068353 1068446] [chr:16 + 7689894 7689987]
(((.(((.((.((((((((((((((((((.((((.........................)))).)))))))))))))))))).))))).)))...         (-38.56)
GUAGACAUGGUGUGGUAUUGGUUCGGCUCAUCUUCUUUCAUUCAUUUUCAUCGGCUCAAAGACGAGCCGAACCAAUAUCACUCUUGUAUGCUUC
                                                            CGAGCCGAACCAAUAUCAC        2
          UGUGGUAUUGGUUCGGCUCAUCa&          1
          ugugguauugguucggcucauc            1

>vvi-MIR482 [sc:101 + 181595 181711] [chr:17 + 5579376 5579492]
......(((((((.((((((((((((((.(((((..((((..((((((((((.........))))))....))))..)))))))))).)))))))))))).)))).)))))))........ (-51.80)
AAUGUUUGGGAAUUGGAGAGUAGGAAAGCUUAGCCAUCUAUUCCCUUCAUGGUUUCCUCUCCAUGGAUUGGGGGUCUAGAGCUAGUCUUUCCUACUCCUCCCAUUCCUAUUGUUUUC
                                                                                   UCUUUCCUACUCCUCCCAUUCCa&         5
                                                                                   UCUUUCCUACUCCUCCCAUU             2
          AAUUGGAGAGUAGGAAAGCUUA            1
             UGGAGAGUAGGAAAGCUUAGCa         1
                AGAGUAGGAAAGCUUAGCCAUC      1
          AAUUGGAGAGUAGGAAAGCU              1
             UGGAGAGUAGGAAAGCUUAGCCAUC      1
                                                                                UAGUCUUUCCUACUCCUCCCAUUC            1
                                                                                        ccuacuccucccauucc           0

>vvi-MIR535a [sc:392 - 4679 4779] [chr:Un_random - 82149759 82149859]
....((((..(((((.(((((((((((((((.((..(((.(((((.......))))).)))..)).))))))))))))))).)))))..))))......  (-51.40)
UGUAAUUGCUUGACAACGAGAGAGAGCACGCUAGUCAGCAUUCAUGCGAGGAACAUGACUGUGUACAGGCGUGCUCUCUCUCGCUGUCAUACAAUCUUCUC
          UGACAACGAGAGAGAGCACGC&            42
          UGACAACGAGAGAGAGCACGCa            38
          UGACAACGAGAGAGAGCAC               2
               ACGAGAGAGAGCACGCUAGUCA       1
                                                                        GCUCUCUCUCGCUGUCAUACAAUC       1
            ACAACGAGAGAGAGCACGCUAGUC        1
          ugacaacgagagagagcacgcu            0

>vvi-MIR535b [sc:392 - 4670 4770] [chr:Un_random - 88067433 88067533]
....((((..(((((.(((((((((((((((.((..(((.(((((.......))))).)))..)).))))))))))))))).)))))..))))......  (-51.40)
UGUAAUUGCUUGACAACGAGAGAGAGCACGCUAGUCAGCAUUCAUGCGAGGAACAUGACUGUGUACAGGCGUGCUCUCUCUCGCUGUCAUACAAUCUUCUC
          UGACAACGAGAGAGAGCACGC&            42
          UGACAACGAGAGAGAGCACGCa            38
          UGACAACGAGAGAGAGCAC               2
               ACGAGAGAGAGCACGCUAGUCA       1
                                                                        GCUCUCUCUCGCUGUCAUACAAUC       1
            ACAACGAGAGAGAGCACGCUAGUC        1
          ugacaacgagagagagcacgcu            0

>vvi-MIR535c [sc:1309 - 299 399] [chr:Un_random - 143826712 143826812]
....((((..(((((.(((((((((((((((.((..(((.(((((.......))))).)))..)).))))))))))))))).)))))..))))......  (-51.40)
UGUAAUUGCUUGACAACGAGAGAGAGCACGCUAGUCAGCAUUCAUGCGAGGAACAUGACUGUGUACAGGCGUGCUCUCUCUCGCUGUCAUACAAUCUUCUC
          UGACAACGAGAGAGAGCACGC&            42
```

```
          UGACAACGAGAGAGAGCACGCa            38
          UGACAACGAGAGAGAGCAC               2
               ACGAGAGAGAGCACGCUAGUCA       1
                                                                             GCUCUCUCUCGCUGUCAUACAAUC       1
            ACAACGAGAGAGAGCACGCUAGUC        1
          ugacaacgagagagagcacgcu            0

>vvi-MIR535d [sc:1152 + 22767 22867] [chr:Un_random - 103652414 103652514]
....(((..(((((.(((.(((((((((((.((..(((.(((((.......))))).)))..)).))))))))))).))).)))))..)))).......  (-47.80)
UGUAAUUGCUUGACAACGAGAGAGAGCACGCUAGUCUGCAUUCAUGCGAGGAACAUGACUGUGUACAGGCGUGCUCUCUGUCGCUGUCAUACAAUCUUCUC
          UGACAACGAGAGAGAGCACGC&            42
          UGACAACGAGAGAGAGCACGCa            38
          UGACAACGAGAGAGAGCAC               2
            ACAACGAGAGAGAGCACGCUAGUC        1
                                                                           GUGCUCUCUGUCGCUGUCAUAa           1
          ugacaacgagagagagcacgcu            0

>vvi-MIR535e [sc:1190 - 13719 13819] [chr:Un_random + 105371228 105371328]
....(((..(((((.(((.(((((((((((.((..(((.(((((.......))))).)))..)).))))))))))).))).)))))..)))).......  (-47.80)
UGUAAUUGCUUGACAACGAGAGAGAGCACGCUAGUCUGCAUUCAUGCGAGGAACAUGACUGUGUACAGGCGUGCUCUCUGUCGCUGUCAUACAAUCUUCUC
          UGACAACGAGAGAGAGCACGC&            42
          UGACAACGAGAGAGAGCACGCa            38
          UGACAACGAGAGAGAGCAC               2
                                                                           GUGCUCUCUGUCGCUGUCAUAa           1
            ACAACGAGAGAGAGCACGCUAGUC        1
          ugacaacgagagagagcacgcu            0

>vvi-MIR828a [sc:86 + 1219681 1219802] [chr:16 + 7841222 7841343]
..(((.(((.(((.((((((((((((..(((.((((((((((((..(.(((((....))))).)..)).)))...)).))))))))).))))))))))))).))).))).)))......            (-42.20)
UAUAAAGUUUCUUGCUCAAAUGAGUAUUCCAAACAACAGCUUGUGAAGCAAUGAUUACUGUCAUAGUCUCUGCAUGUGGAUUGUUGUUGAGAUGCUCAUUUGAGGAAGCAACCUUAAAAAUG
         ucuugcucaaaugaguauucca&            0

>vvi-MIR828b [sc:5 + 2945837 2945908] [chr:1 + 2945837 2945908]
...((((((((..(((..((((((.(((.(((.........)))))).)))))))...)))))))))))).....  (-21.00)
GCUCUUGGUUUCUUGCUCAAAUGAGUGUUCCACAUCAACCUGGAUAGUCAUUUCCUGCAAAACCAGGUUUCU
          ucuugcucaaaugaguguucca&           0

>vvi-MIR845a [sc:86 + 3156 3597] [chr:16 + 6624697 6625138]
.....(((.(((((((...(((.((((((((((..............(((.(((...((((......(((.((((......))))))).....))))...((.....)).))).)))...(((((...(((((((..((((((..(((((((((((((.((((((((((...((((.(((((.((.......(.(((((((.((((((...(((....((((((((.....)))))...)))))).))))))).))))))).)...)).)))))).....(..(((.(((.....))
).)))..)..))))..)))))))))))......((((..(((........)))..)))))))))))))))))..))..))))))...)))))))...)))))....))))......)))))))))....)))))))......)))..   (-108.60)
UUUCACUUGAUUAGUUCCUUCAUGAGUUAUUGCAAGUAAUCUCCAAAUUUCCUUAGCGGAUUUACAUUGACAUAUUCUAUUAUAUUCAUUCCUAUCCAUAGCACAUUGCAAAGUAAAAACGACUUUAGCAUUUAAUUGAAAAUUUCUUCUAUCAAGCUCAUUCCAUUCUUGCUUGGGUUUUGGAACCAAAAUUCCAUCAACUAAUUUAGUGGGUAAAGUUGGGCCAUCUUCAAUGGCAUCCCAUACAUCUAAAUCAGUUGAUUGUAAGUACCAAGUCAUUCUAGCUUUCCAAUAGGG
AUAGUCGGUUCCCGUAAAGAAUGGAGCUCUAUGUUUUGAACAAUUUUCAGUUUGAGAUGAGCUUGAUGGAAUAGCCAUUUUCCUCUUAGAUGAUUAAGUCUUAAGCAAGAGGUCUAGCUCUGAUACCAAUUGAUAAAACAAAGGC

uagcucugauaccaauugaua&        0

>vvi-MIR845b [sc:216 + 313861 314302] [chr:Un_random + 53496712 53497153]
.....(((.(((((((...(((.((((((((((..((......................(((((..(((.((((......)))))))............((.....))...)))))...(((((...(((((((..((((((((.(((((((((((((.((((((((((((((((((((((((....)))))))).(((((((.(((((((((((...((((((.....))))))....))))).....)))))).)))))))...)))).........................(((
(........))))...))))))))))......((((..(((........)))..)))))))))))))))))).))..))))))...)))))))...)))))....))..)))))..)))))))))....)))))))......)))..   (-106.10)
UUUCACUUGAUUAGUUCCUUCAUGAGUUAUUUCAAGCAAUCUCCAAAUUUCCUUAGCAGAUUUACAUUGGCAUAUUCUAUUAUAUUCAUUCCUAUCCAUAGCACAUUGCAAAGUAAAAACGACUUUAGCAUUUAAUUGAAAAUUUCUUCUAUCAAGCUCAUUCCAUUCUUGCUUGAGUUUUGGAACCAAAACUCCAUCAACUAAUUUAGUGGGAAAAGUUGGGCCAUCUUCAAUGACAUCCCAUACAUCUAAAUCAGUUGAUUGUAAGUACCAAGUCAUUCUAACUUUCCAAUAGGG
AUAGUCGGUUCCCGUAAAGAAUGGAGCUCUAUGUUUUGAACAAUUUUCAGUUUGAGAUGAGCUUGAUGGAACAACCAUUUUCCUCUUAGAUGAUUAAGUCUUAAGCAAGAGGUCUAGCUCUGAUACCAAUUGAUAAAACAAAGGC

uagcucugauaccaauugaua&        0

>vvi-MIR845c [sc:31 - 2686178 2686267] [chr:7 + 503529 503618]
...(((((((((((((.(((.((((((((((((...((((.......)))).)))))))))))).))))))))..))))))))).....             (-30.30)
CAAUUAUGUGUUUUGGUUGUCUGAGUUUCAUGUUGGCAAUUUCUUAAUGAUUAAACAUGAGGCUCUGAUACCAAUUGAUGCAUAAACCAU
                                                           aggcucugauaccaauugaug&       0

>vvi-MIR845d [sc:30 - 551951 552081] [chr:Un_random - 108642758 108642888]
....((..((((((((.....(((((((((((..............(((..(((((..((((......))))..)))))...))).........))))))))))).....)))))))))........  (-32.25)
AUAACCAACAUUAAUUAUCCCUAGGGCCAUGGGAUAAACAAAAACUCUGCAAAACCAAGUUAGCACACCCUAGAAUGGUUCUAGCACAAUUACUAACCUAUGGCUCUGAUACCAAUUGAUGGGAAAAACCU
                                                                                                    uggcucugauaccaauugaug&          0

>vvi-MIR845e [sc:168 + 268220 268350] [chr:Un_random + 37572078 37572208]
........((((((((.....(((((((((((............((((......))))..........(((((.....)))))............)))))))))))).....))))))))..........  (-31.30)
AGAACGCACAUUAAUUAUCCCUAGGGCCAUGGGAUAAACAAAAACCUUGAAAAAACAAGUUAGCACACCCUAGAAUGGUUCUAGCACGAUUACUAACCUAUGGCUCUGAUACCAAUUGAUGGGAAAAACUC
                                                                                                    uggcucugauaccaauugaug&          0
```

Supplemental Figure S3: predicted transcript and intron coordinates for 25 *Vitis vinifera* pri-miRNAs. For each miRNA genomic locus showing extensive coverage with Illumina RNAseq reads, Transcription start and (where possible) stop coordinates were estimated from read coverage. Introns were predicted as explained in the main text. The table shows transcript coordinates estimated from different tissues, frequencies of support for introns(by tissue), splice site scores, promoter coordinates estimated by TSS-TCM, and notes showing additional support obtained for introns and transcrtipt coordinates

croRNA	Chr	strand	TSS(TSSP-TCM)	TATA(TSSP-TCM)	Leaf start	Stem start	Root start	Callus start	Leaf Stop	Stem stop	Root stop	Callus stop	donor	donor score	donor support	acceptor	acceptor score	acceptor support	leaf	stem	root	callus	f reads	Additional Info (support for introns, RACE start/stop
iR156d	chr11	-	5634584	TATA-less	5634380	5634415	5634364	5634353	5631341	5631328	5633903	nd												
													5633555	0.571000		5633474	0.330000		0	2	0	0	2	
													5633512	0.389000		5631712	0.164000	leaf callus	2	0	0	0	1	
													5633184	0.370000	stem	5631712	0.164000	leaf callus	1	0	0	0	1	
													5632979	0.593000		5632856	0.392000	stem	0	7	0	0	3	
													5632655	0.606000		5632529	0.426000	leaf stem	2	17	0	1	10	
													5631801	0.328000		5631712	0.164000	leaf callus	1	0	0	0	1	
iR156g	chr17	-	No pred	no	3038168	3037793	nd	3038273	3036995	3037004	nd	3037013												
													3038152	0.355000		3037280	0.490000	stem callus	2	0	0	0	1	
													3037895	0.524000		3037280	0.490000	stem callus	1	0	0	1	2	454 leaf
													3037783	0.430000	stem callus	3037280	0.490000	stem callus	3	1	0	0	2	
													3037711	0.555000		3037280	0.490000	stem callus	12	3	0	0	8	
													3037596	0.401000	stem callus	3037280	0.490000	stem callus	1	0	0	0	1	
													3037392	0.503000	stem callus	3037280	0.490000	stem callus	0	4	0	1	4	
miR156i	chrUn_random	+	36776661	36776634	36776678	36776678	36776742	36776743	nd	nd	nd	nd												
iR159c	chr17	-	No pred	no	2610336	2610326	2610317	2610317	2609123	2609131	nd	2609196												
													2610173	0.508000		2609343	0.374000	leaf stem root	22	8	0	2	8	
iR160c	chr10	-	8894350	8894374	8894318	8894386	8894350	8894371	nd	nd	nd	nd												
													8893990	0.577000		8893881	0.505000	leaf stem root callus	0	3	1	0	4	
miR162	chr17	+	4714318	TATA-less	4714680	4714686	4714687	4714687	4717308	4717340	4717273	4717307												RACE STOP: 4717237/4715594/4715751/4717045/4716530
													4716567	0.501000		4716673	0.490000		4	1	0	0	2	
													4715520	0.504000		4716673	0.490000		0	2	1	1	3	454 berry/RACE
													4715520	0.504000		4716420	0.366000		0	2	0	0	2	
													4715510	0.471000		4716673	0.490000		0	1	0	0	1	RACE
													4715507	0.501000		4716673	0.490000		0	1	0	0	1	
													4715476	0.440000		4716673	0.490000		1	0	0	0	1	454 leaf
													4715350	0.373000		4715429	0.481000		3	0	0	0	1	
													4714863	0.390000		4716240	0.244000		0	1	0	0	1	
													4714768	0.407000		4715429	0.481000		3	1	0	0	2	
													4714759	0.377000		4716673	0.490000		0	0	0	1	1	
													4714759	0.377000		4715429	0.481000		5	3	0	0	4	454 leaf
													4714740	0.470000		4715429	0.481000		3	1	0	0	3	
													4714729	0.426000		4715429	0.481000		3	2	0	1	6	
iR167a	chr1	+	No pred	no	1563294	nd	1563320	1563291	1563916	nd	1563933	1563912												
iR167d	chr7	-	845566	845597	845542	845545	nd	nd	844270	844250	nd	nd												
													844837	0.489000	leaf	844632	0.370000	stem	0	1	0	0	1	
miR168	chr2	-	16750258	16750282	16750255	16750240	16750225	16750239	16747427	16747408	nd	16747419												
													16750027	0.467000		16748197	0.506000	leaf root	2	0	0	0	1	454 berry
													16750019	0.448000		16748197	0.506000	leaf root	4	8	1	5	6	454 berry
													16748088	0.482000		16747680	0.448000	leaf stem callus	0	1	0	0	1	
iR169g	chr8	+	No pred	no	20118544	nd	nd	nd	20119875	nd	nd	nd												
iR171a	chr14	-	No pred	no	14694238	14694267	14694292	14694236	nd	14693362	nd	nd												
iR319c	chr2	-	No pred	no	nd	662167	nd	nd	nd	660898	nd	nd												
iR393b	chr13	+	No pred	no	4372918	4372919	nd	nd	4373963	4373978	nd	nd												
													4373255	0.551000		4373664	0.402000		1	0	0	0	1	
iR394b	chr18	-	1386133	TATA-less	1386084	1386013	nd	nd	1385071	1384873	nd	nd												RACE STOP 1385081/1385085/1385121/1385102/1385108
													1385724	0.510000		1385362	0.467000	leaf stem callus	3	11	0	1	7	RACE
iR396b	chr11	+	No pred	no	5205873	5205921	5205886	5205883	5208124	5208135	5208011	5208094												
													5206245	0.545000		5206387	0.369000	stem callus	3	1	1	0	5	
													5206451	0.542000		5207749	0.404000		0	1	0	1	2	
													5206513	0.439000		5207086	0.171000		0	1	0	0	1	
													5207688	0.409000	leaf	5207749	0.404000		0	0	0	1	1	
													5207750	0.538000		5207816	0.363000	leaf	0	1	0	0	1	
iR397a	chrUn_random	+	No pred	no	85971353	85971349	85971353	85971342	nd	nd	nd	nd												
													85971560	0.435000		85971702	0.385000		0	1	0	0	1	
													85972847	0.571000		85972917	0.439000	stem root callus	0	5	0	40	11	
													85973070	0.598000		85973212	0.454000	stem callus	0	2	0	0	2	
iR397b	chrUn_random	-	No pred	no	146568205	146568210	146568207	146568217	nd	nd	nd	nd												
													146567996	0.435000		146567862	0.385000		0	1	0	2	2	
iR398a	chr1	+	No pred	no	717331	717187	717264	717248	719184	717198	719188	719200												
													717390	0.499000		717577	0.314000	leaf stem root callus	0	2	1	1	4	
													717390	0.499000		717687	0.351000		0	1	0	0	1	
													717390	0.499000		718148	0.353000	leaf stem root callus	0	1	0	0	1	
													717855	0.510000		718148	0.353000	leaf stem root callus	2	15	2	4	11	
													718282	0.533000		718370	0.390000	leaf stem root callus	2	8	0	4	8	
													718500	0.479000		719049	0.363000	leaf stem root callus	0	0	0	1	1	
iR398b	chr6	-	No pred	no	20660773	20660783	20660768	20660767	20660459	20660448	nd	20660443												
iR398c	chr6	+	No pred	no	14851851	14851857	14851856	14851858	14852180	14852215	nd	14852186												
iR403a	chr5	-	1835411	1835441	nd	1835395	1835420	1835389	nd	nd	nd	nd												
													1834959	0.568000		1834086	0.411000		0	4	0	0	2	454 leaf
													1835273	0.431000		1835195	0.349000		0	2	0	0	2	
iR403c	chr5	-	1302236	1302265	1302234	1302219	1302219	1302232	1301756	1301756	1301760	1301760												
													1302097	0.365000		1302012	0.349000		0	2	0	0	2	
iR403e	chr5	-	No pred	no	1738240	1738247	1738248	1738231	nd	nd	nd	nd												
													1737325	0.632000		1737240	0.404000		1	2	0	8	8	
													1738206	0.568000		1737402	0.303000		1	1	1	0	3	
miR408	chr7	+	No pred	no	5372904	5372907	5372907	5372896	5374243	5374226	5374255	5374207												
													5373063	0.555000		5373688	0.179000	leaf root	2	1	0	0	2	
													5373063	0.555000		5373709	0.261000	root	0	0	0	1	1	
													5373063	0.555000		5373745	0.303000		0	2	0	0	2	
													5373063	0.555000		5373858	0.327000	root callus	208	353	143	195	31	
iR482a	chr17	+	No pred	no	5579315	5579315	5579315	5579315	5580798	5580493	5580438	5581255												RACE START: 5579321 RACE STOP: 5580180
													5579322	0.514000	leaf stem root callus	5579599	0.482000	leaf stem root callus	1	0	0	0	1	
													5579346	0.367000	leaf stem root callus	5579599	0.482000	leaf stem root callus	5	0	0	0	2	
													5579360	0.390000	stem callus	5579599	0.482000	leaf stem root callus	0	1	0	0	1	

Supplemental Figure S4: RNAseq reads Supporting predicted splice junctions for *Vitis vinifera* pri-miRNAs shown in Figure 3

```
Vvi-MIR162      chr17   +      4716567                          4716673
CCCCAGGCAGCAAAATTTAGTGTTTCCACAGGTTGCATTTTTG ........ TTTCTTGAGCAGGTATCTGGAATCGGAAAGTTGTTTCTTGTTT
          CAAAATTTAGTGTTTCCACAG                                  GTATCTGGA                    3
         GCAAAATTTAGTGTTTCCACAG                                  GTATCTGG                     2

Vvi-MIR162      chr17   +      4715520                          4716673
GCCAAATTCTTGCTGAATGTAGTAATTTCCAGTAAATTTTAAT ........ TTTCTTGAGCAGGTATCTGGAATCGGAAAGTTGTTTCTTGTTT
        CTTGCTGAATGTAGTAATTTCCA                                  GTATCTGGA                    1
              GAATGTAGTAATTTCCA                                  GTATCTGGAATCG                1
           GCTGAATGTAGTAATTTCCA                                  GTATCTGGAA                   2

Vvi-MIR162      chr17   +      4715520                         4716420
GCCAAATTCTTGCTGAATGTAGTAATTTCCAGTAAATTTTAAT ........ GTTCTTGAGTAGGGGGGATAAGGCTGCTGGTTTTGCGAAGTGC
       TCTTGCTGAATGTAGTAATTTCCA                                  GGGGGA                       1
               AATGTAGTAATTTCCA                                  GGGGGATAAGGCTG               1

Vvi-MIR162      chr17   +      4715510                          4716673
GCATCACAACGCCAAATTCTTGCTGAATGTAGTAATTTCCAGT ........ TTTCTTGAGCAGGTATCTGGAATCGGAAAGTTGTTTCTTGTTT
                 TCTTGCTGAATGTA                                  GTATCTGGAATCGGAA             1

Vvi-MIR162      chr17   +      4715507                          4716673
GTAGCATCACAACGCCAAATTCTTGCTGAATGTAGTAATTTCC ........ TTTCTTGAGCAGGTATCTGGAATCGGAAAGTTGTTTCTTGTTT
       CACAACGCCAAATTCTTGCTGAAT                                  GTATCT                       1

Vvi-MIR162      chr17   +      4715476                          4716673
CTGTGTTCTTCTGTGTTTCGAACAGACTCTGGTAGCATCACAA ........ TTTCTTGAGCAGGTATCTGGAATCGGAAAGTTGTTTCTTGTTT
                      CAGACTCTG                                  GTATCTGGAATCGGAAAGTTG        1

Vvi-MIR162      chr17   +      4715350                          4715429
AGACCATGTTCACAAATAGTCTTGTAAAGCTGTAACAGCCTGA ........ AGTTTATTGCAGGGAAGGAGATCCGCCCTGTGTTCTTCTGTGT
              AATAGTCTTGTAAAGCT                                  GGAAGGAGATCCG                3
```

```
Vvi-MIR162      chr17   +      4714863                          4716240
TCATTTGGTCAGATCTGTGGTTTTTGATTTTGTGTTTTTGAAA ........ ACACTCCATAAGGTTTTTAATTGGGTTAACTTCTATTCTCAT
       GTCAGATCTGTGGTTTTTGATTTT                                  GTTTTT                       1

Vvi-MIR162      chr17   +      4714768                          4715429
ATGGTGACCCTTCAGATTCCTGGTTCACGCTGTTACTCTTTCT ........ AGTTTATTGCAGGGAAGGAGATCCGCCCTGTGTTCTTCTGTGT
                 TCCTGGTTCACGCT                                  GGAAGGAGATCCGCCC             1
              GATTCCTGGTTCACGCT                                  GGAAGGAGATCCG                3

Vvi-MIR162      chr17   +      4714759                          4716673
CGTACGGCAATGGTGACCCTTCAGATTCCTGGTTCACGCTGTT ........ TTTCTTGAGCAGGTATCTGGAATCGGAAAGTTGTTTCTTGTTT
                    TCAGATTCCTG                                  GTATCTGGAATCGGAAAGTTG        1

Vvi-MIR162      chr17   +      4714759                          4715429
CGTACGGCAATGGTGACCCTTCAGATTCCTGGTTCACGCTGTT ........ AGTTTATTGCAGGGAAGGAGATCCGCCCTGTGTTCTTCTGTGT
                     CAGATTCCTG                                  GGAAGGAGATCCGCCCTGTG         2
              GACCCTTCAGATTCCTG                                  GGAAGGAGATCCG                3
            GTGACCCTTCAGATTCCTG                                  GGAAGGAGATC                  1
             TGACCCTTCAGATTCCTG                                  GGAAGGAGATCC                 2

Vvi-MIR162      chr17   +      4714740                          4715429
AGAGAGAGAGGGAGAAAAACGTACGGCAATGGTGACCCTTCAG ........ AGTTTATTGCAGGGAAGGAGATCCGCCCTGTGTTCTTCTGTGT
             GAAAAACGTACGGCAATG                                  GGAAGGAGATCC                 1
                   CGTACGGCAATG                                  GGAAGGAGATCCGCCCTG           1
            AGAAAAACGTACGGCAATG                                  GGAAGGAGATC                  2

Vvi-MIR162      chr17   +      4714729                          4715429
ATAGAGAAGGGAGAGAGAGAGGGAGAAAAACGTACGGCAATGG ........ AGTTTATTGCAGGGAAGGAGATCCGCCCTGTGTTCTTCTGTGT
        GGGAGAGAGAGAGGGAGAAAAAC                                  GGAAGGAGA                    1
                        GAAAAAC                                  GGAAGGAGATCCGCCCTGTGTTC      1
                GAGAGGGAGAAAAAC                                  GGAAGGAGATCCGCC              1
             AGAGAGAGGGAGAAAAAC                                  GGAAGGAGATCC                 1
              GAGAGAGGGAGAAAAAC                                  GGAAGGAGATCCG                1
            GAGAGAGAGGGAGAAAAAC                                  GGAAGGAGATC                  1
```

```
Vvi-MIR168      chr2    -      16750027                         16748197
ATGTGATGATGAAAGACTACTTCGATCTCAGGTTTCTAGGTTG ........ GCTTGTTTTCAGGTGCGGGGGCTCAACAAATTTGTTGCAGGGC
          GAAAGACTACTTCGATCTCAG                                  GTGCGGGGG                   2


Vvi-MIR168      chr2    -      16750019                         16748197
ATGAAAGACTACTTCGATCTCAGGTTTCTAGGTTGGAAAAATT ........ GCTTGTTTTCAGGTGCGGGGGCTCAACAAATTTGTTGCAGGGC
           CTTCGATCTCAGGTTTCTAG                                  GTGCGGGGGC                  5
                       GTTTCTAG                                  GTGCGGGGGCTCAACAAATTTG      3
        CTACTTCGATCTCAGGTTTCTAG                                  GTGCGGG                     4
                ATCTCAGGTTTCTAG                                  GTGCGGGGGCTCAAC             1
               GATCTCAGGTTTCTAG                                  GTGCGGGGGCTCAACA            2
                     AGGTTTCTAG                                  GTGCGGGGGCTCAACAAATT        3

Vvi-MIR168      chr2    -      16748088                         16747680
TCAACCCTAACAATTATTGTCACATGCCCAGGTTTCTTGGTAA ........ TTTATTCTGTAGATCATTGCATGATTGGCCCATTCTCCTCTCT
                      CATGCCCAG                                  ATCATTGCATGATTGGCCCAT   1

Vvi-MIR394B     chr18   -      1385724                          1385362
CTCTCTCGCTCTTCCACTCTAGAGCATCAAGGTGAAAACCCCA ........ CTTGTGTTGCAGGGGTTTCATCAACTCCTCCTCTTTGCCTCTT
                  CTAGAGCATCAAG                                  GGGTTTCATCAACTCCT           1
         TCTTCCACTCTAGAGCATCAAG                                  GGGTTTCATC                  1
                     GAGCATCAAG                                  GGGTTTCATCAACTCCTCCT        2
            TCCACTCTAGAGCATCAAG                                  GGGTTTCATCA                 1
                        CATCAAG                                  GGGTTTCATCAACTCCTCCTCTT     2
           TTCCACTCTAGAGCATCAAG                                  GGGTTTCATC                  5
                    AGAGCATCAAG                                  GGGTTTCATCAACTCCTCC         3
```
