# Supplementary material for: Correction: High throughput approaches reveal splicing of primary microRNA transcripts and tissue specific expression of mature microRNAs in Vitis vinifera
Source: BMC Genomics. 2010 Feb 12;11:109. doi: 10.1186/1471-2164-11-109 (PMC2831844; doi:10.1186/1471-2164-11-109)
Supplement: Additional file 2 — Oligonucleotide Array probes for analysis of Vitis vinifera miRNA expression. Supplemental Table S5: All oligonucleotide array probe sequences. [file 1471-2164-11-109-S2.PDF]

Supplemental Figure S5. Combimatrix microarray spotted probes list.

Probe Name	Probe Sequence 5’-3'	Probe Type
vvi|tRNA-Ala(Chlor)_12_32|PosCtrl|as|	tcagttggtagagctccgctc	Positive control
vvi|tRNA-Phe(Chlor)_48_68|PosCtrl|as|	ccagttcaaatctggttcctg	Positive control
vvi|tRNA-Phe(Chlor)_37_57|PosCtrl|as|	tcctcgtgtcaccagttcaaa	Positive control
vvi|tRNA-Lys(Chlor)_2_23|PosCtrl|as|	ggttgctaactcaatggtagag	Positive control
vvi|tRNA-His(Chlor)_52_72|PosCtrl|as|	cgggttcaattcccgtcgttc	Positive control
vvi|tRNA-His(Chlor)_19_39|PosCtrl|as|	gatcaaggcagtggattgtga	Positive control
vvi|tRNA-Ser(Chlor)_51_71|PosCtrl|as|	agttattcgtaccgagggttc	Positive control
vvi|tRNA-Ser(Chlor)_38_59|PosCtrl|as|	aatccgttgtacgagttattcg	Positive control
vvi|tRNA-Tyr(Chlor)_43_63|PosCtrl|as|	gttggcaatatgtctacgctg	Positive control
vvi|tRNA-Tyr(Chlor)_4_24|PosCtrl|as|	tcgatgcccgagcggctaatg	Positive control
vvi|tRNA-Met(Chlor)_48_68|PosCtrl|as|	cacgggttcaaatcctgtctc	Positive control
vvi|tRNA-Met(Chlor)_33_53|PosCtrl|as|	tcataaccttgaggtcacggg	Positive control
vvi|tRNA-Ile(Chlor)_10_30|PosCtrl|as|	ctctggttcaagtccaggatg	Positive control
vvi|UBQ(AY684128)_74_94|DegradCtrl|as|	acttggtcctccgcctgagag	Degradation control
vvi|Act(AY847627)_170_190|DegradCtrl|as|	ttgtgcttagtggtgggtcga	Degradation control
vvi|Act(AY847627)_144_165|DegradCtrl|as|	tatcaggaaggacctctatggc	Degradation control
vvi|Hsp101(AY764155)_2888_2908|DegradCtrl|as|	gcagtgacctgtaattggtct	Degradation control
vvi|Hsp101(AY764155)_2660_2680|DegradCtrl|as|	tcagacgtgttgattcggatc	Degradation control
vvi|GDBrPK(AF178575)_1085_1106|DegradCtrl|as|	gtcagtggcattgattatcaga	Degradation control
vvi|GDBrPK(AF178575)_1072_1092|DegradCtrl|as|	cagtgcgttcatggtcagtgg	Degradation control
neg|Bio B Spike_394_418|NegCtrl|as|	gcgcctgagattcactcaacgtgcc	Negative control
neg|Bio C Spike_113_137|NegCtrl|as|	acgtgggtgtatttacgctgtggaa	Negative control
neg|Lambda Spike 12_609_633|NegCtrl|as|	cttccggcaatactcgtaaaccata	Negative control
neg|Lambda Spike 5_753_777|NegCtrl|as|	gaacaggttatcgaaatcagccaca	Negative control
neg|Lambda Spike 6_497_521|NegCtrl|as|	tacaaccggaatgttgaccttgcct	Negative control
neg|Lambda Spike 8_329_353|NegCtrl|as|	gtcacctttatctgccgccactcat	Negative control
neg|Lambda Spike 9_1167_1191|NegCtrl|as|	gatgtttgcagacgtaatggtgcgg	Negative control
QC-oligoB-AS-3	gagttgctgttacatcagcgaatgtgcatggaacacactt	Quality control
QC-3prime	tgactgactggactgtgggtgtgcgatacgtgtcc	Quality control

QC-5prime	tgggtgtgcgatacgtgtccgactgactgactgac	Quality control
QC-LLD-1	agatttcttcggtttcccgtggttggcgtctga	Quality control
QC-LLD-2	ggagtatgttcggctgcgcgagagctatgatgccg	Quality control
QC-LLD-3	gtggcgatgttaacctcggcttatggcgtcaccgg	Quality control
QC-LLD-4	cgaacatcccagggtaacacgca	Quality control
NC-SP-phage-03-3	caccgtaattactgtgagccatcatgacgccgatg	Generic negative control
NC-SP-phage-03-7	gacggtgatcgcatcggtgctgttaacaagttcat	Generic negative control
NC-SP-phage-03-8	agtggctgaaacagttgttgattgagtctctttgg	Generic negative control
NC-SP-phage-05-2	ccatgattacgccagttgtacggacacgaactcat	Generic negative control
NC-SP-phage-05-1	ggagacgggcaatcagttcatctttcgtcatggac	Generic negative control
NC-SP-phage-05-16	cctgtccggcatagctgatattttcagggtcatca	Generic negative control
NC-SP-phage-06-1	gactctccggattaactggcggtgacggtaatttc	Generic negative control
NC-SP-phage-06-8	tcatacataccagacagccggtacaccacgttttc	Generic negative control
NC-SP-phage-06-14	gtcacaccgttatgttcaaatgattcggttttcag	Generic negative control
NC-SP-phage-08-9	gtgaccataccgtacaggttagaaaccgtcagcgt	Generic negative control
NC-SP-phage-08-10	ggatcggcgtaactgtttccgttgacgaagttcac	Generic negative control
NC-SP-phage-08-13	ggacagaaagcgtcacgctgtacgttttcaggttg	Generic negative control
NC-SP-phage-09-4	cgaccaaatcgttgtaaatccccgtaaaggcagat	Generic negative control
NC-SP-phage-09-7	catcaggcagagtctcatgtaactgcgccgttaac	Generic negative control
NC-SP-phage-09-11	ctctgggaacaatatgaattacagcgccatcaggc	Generic negative control
NC-SP-phage-12-6	gttacggttgatttcgagttgggtccacttatcgc	Generic negative control
NC-SP-phage-12-14	ccagtaactggctgaacacaccgttgatgatctgc	Generic negative control
NC-SP-phage-12-15	catgttctgatacaggttggtaaccagccagtagt	Generic negative control
NC-bacteria-10-7	gaagaagaatcagggcgacacggtctccatttttg	Generic negative control
NC-bacteria-10-2	cgagcttcaacagcgtccagaataacggtgaacaa	Generic negative control
NC-bacteria-10-5	catgtatcttgcaaacggcaatcgacaccagtctg	Generic negative control
NC-bacteria-9-4	aaagggagctatgtcgagatttcttcggtttcccg	Generic negative control
NC-bacteria-9-9	gaagctgtcgctaactattcagtcaaagggagcta	Generic negative control
NC-bacteria-9-11	gaagtcttcgatgatggatttacgacggtatttca	Generic negative control
NC-bacteria-5-4	ataacgatggtggcgatgttaacctcggcttatgg	Generic negative control
NC-bacteria-5-11	gtaaggagcatcaaaagactgcggtcatctttggg	Generic negative control
NC-bacteria-5-12	aaatgtacaggcgaccagcttgttaacgatgacca	Generic negative control
NC-plant-1	cctaccgtaggtcgacaatatagatattttgttgg	Generic negative control
NC-plant-2	gcggaagaagttgtcggagcaatcgtcatgggaa	Generic negative control

NC-plant-3	tggtaaacagagttcctctaattgtcacagggaac	Generic negative control
NC-plant-4	agcagatctcaaagattgtaggagacatggacgtc	Generic negative control
NC-plant-5	cagcaagctctagaagttcagaggaggcaggagag	Generic negative control
NC-plant-6	ccaactagcgagtagaatcaactatcagcaatggc	Generic negative control
NC-plant-7	ggaagaagaaatacgcacaagtttgtgtcacaatg	Generic negative control
NC-plant-8	acattcgcaaggaaccgtgagctagaagttatcca	Generic negative control
vvi|snRNAU6|nat|as|	tgcgtgtcatccttgcgcagg	Custom Positive Control
vvi|snRNAU6|2mut|as|	tgcgtgtcattcttgcccagg	Custom Positive Control
scaffold_2358|+|1257|1330|10-30|tRNA|nat|as|	cactgccttgatccacttgg	Custom Positive Control
scaffold_2358|+|1257|1330|10-30|tRNA|2mut|as|	cactgtcttgatcaacttgg	Custom Positive Control
scaffold_105|-|365541|365614|10-30|tRNA|nat|as|	tagcgtgataaccaactaca	Custom Positive Control
scaffold_105|-|365541|365614|10-30|tRNA|2mut|as|	tagcctgataacaaactaca	Custom Positive Control
scaffold_111|+|86247|86318|10-30|tRNA|nat|as|	cccaccacgcttccgctgcg	Custom Positive Control
scaffold_111|+|86247|86318|10-30|tRNA|2mut|as|	cccaccacccttcccctgcg	Custom Positive Control
scaffold_834|-|23630|23701|10-30|tRNA|nat|as|	tcagacgcgctaccattgcg	Custom Positive Control
scaffold_834|-|23630|23701|10-30|tRNA|2mut|as|	tcagacgccctacaattgcg	Custom Positive Control
vvi|miR-1|nat|as|	gtgctccctctcttctgtca	vvi-miR156a_perfect match probe
vvi|miR-1|2mut|as|	gtgctctctctcttccgtca	vvi-miR156a_mismatch probe
vvi|miR-2|nat|as|	gtgctcactctcttctgtca	vvi-miR156b_perfect match probe
vvi|miR-2|2mut|as|	gtgctccctctcttccgtca	vvi-miR156b_mismatch probe
vvi|miR-3|nat|as|	gtgctcactctcctctgtca	vvi-miR156e_perfect match probe
vvi|miR-3|2mut|as|	gtgctccctcttctctgtca	vvi-miR156e_mismatch probe
vvi|miR-4|nat|as|	gtgctctctatcttctgtcaa	vvi-miR156f_perfect match probe
vvi|miR-4|2mut|as|	gtgctccctatcttctctcaa	vvi-miR156f_mismatch probe
vvi|miR-5|nat|as|	atgctctctctcttctgtca	vvi-miR156h_perfect match probe
vvi|miR-5|2mut|as|	atgctctccctcttccgtca	vvi-miR156h_mismatch probe
vvi|miR-6|nat|as|	gtgctctctatcttctgtca	vvi-miR156i_perfect match probe
vvi|miR-6|2mut|as|	gtgctccctatcttccgtca	vvi-miR156i_mismatch probe
vvi|miR-7|nat|as|	gagagctcccttcactccaag	vvi-miR159a_perfect match probe
vvi|miR-7|2mut|as|	gagagctctcttcacttcaag	vvi-miR159a_mismatch probe
vvi|miR-8|nat|as|	tgagagctcccttcactccaag	vvi-miR159b_perfect match probe
vvi|miR-8|2mut|as|	tgagagctctcttcacttcaag	vvi-miR159b_mismatch probe
vvi|miR-9|nat|as|	tagagctcccttcaatccaaa	vvi-miR159c_perfect match probe
vvi|miR-9|2mut|as|	tagagctctcttcaattcaaa	vvi-miR159c_mismatch probe

vvi|miR-10|nat|as|	tggcattcagggagccaggca	vvi-miR160a_perfect match probe
vvi|miR-10|2mut|as|	tggcattcagtgagtcaggca	vvi-miR160a_mismatch probe
vvi|miR-11|nat|as|	tggcatacagggagccaggca	vvi-miR160c_perfect match probe
vvi|miR-11|2mut|as|	tggcatacagtgagtcaggca	vvi-miR160c_mismatch probe
vvi|miR-12|nat|as|	ctggatgcagaggtttatcga	vvi-miR162_perfect match probe
vvi|miR-12|2mut|as|	ctggatgaagagctttatcga	vvi-miR162_mismatch probe
vvi|miR-13|nat|as|	tgcacgtgccctgcttctcca	vvi-miR164a_perfect match probe
vvi|miR-13|2mut|as|	tgcacgtgtcctccttctcca	vvi-miR164a_mismatch probe
vvi|miR-14|nat|as|	agcatgtgccctgcttctcca	vvi-miR164b_perfect match probe
vvi|miR-14|2mut|as|	agcatgtgtcctccttctcca	vvi-miR164b_mismatch probe
vvi|miR-15|nat|as|	gggggaatgaagcctggtccga	vvi-miR166c_perfect match probe
vvi|miR-15|2mut|as|	ggggaaatgaagtctggtccga	vvi-miR166c_mismatch probe
vvi|miR-16|nat|as|	aggggaatgaagcctggtccga	vvi-miR166d_perfect match probe
vvi|miR-16|2mut|as|	agggaaatgaagtctggtccga	vvi-miR166d_mismatch probe
vvi|miR-17|nat|as|	caggaatgaagcctggtccga	vvi-miR166a_perfect match probe
vvi|miR-17|2mut|as|	caggaatgaactctggtccga	vvi-miR166a_mismatch probe
vvi|miR-18|nat|as|	gaggaatgaagcctggtccga	vvi-miR166b_perfect match probe
vvi|miR-18|2mut|as|	gaggaatgaactctggtccga	vvi-miR166b_mismatch probe
vvi|miR-19|nat|as|	cagatcatgctggcagcttca	vvi-miR167a_perfect match probe
vvi|miR-19|2mut|as|	cagatcatcctgccagcttca	vvi-miR167a_mismatch probe
vvi|miR-20|nat|as|	tagatcatgctggcagcttca	vvi-miR167b_perfect match probe
vvi|miR-20|2mut|as|	tagatcatcctgccagcttca	vvi-miR167b_mismatch probe
vvi|miR-21|nat|as|	agatcatgctggcagcttca	vvi-miR167c_perfect match probe
vvi|miR-21|2mut|as|	agatcatcctgccagcttca	vvi-miR167c_mismatch probe
vvi|miR-22|nat|as|	ttcccgacctgcaccaagcga	vvi-miR168_perfect match probe
vvi|miR-22|2mut|as|	ttccagacctgaaccaagcga	vvi-miR168_mismatch probe
vvi|miR-23|nat|as|	ccggcaagtcatccttggctg	vvi-miR169a_perfect match probe
vvi|miR-23|2mut|as|	ccggaaagtcatccttcgctg	vvi-miR169a_mismatch probe
vvi|miR-24|nat|as|	taggcaagtcatccttcgcta	vvi-miR169y_perfect match probe
vvi|miR-24|2mut|as|	taggaaagtcatccttagcta	vvi-miR169y_mismatch probe
vvi|miR-25|nat|as|	cggcaagccatccttggctca	vvi-miR169b_perfect match probe
vvi|miR-25|2mut|as|	cggcaagtcatccttgcctca	vvi-miR169b_mismatch probe
vvi|miR-26|nat|as|	ccggcaaatcattcttggctg	vvi-miR169d_perfect match probe
vvi|miR-26|2mut|as|	ccggaaaatcattcttcgctg	vvi-miR169d_mismatch probe

vvi|miR-27|nat|as|	caggcaagtcatccttggcta	vvi-miR169e_perfect match probe
vvi|miR-27|2mut|as|	caggaaagtcatccttcgcta	vvi-miR169e_mismatch probe
vvi|miR-28|nat|as|	tcggcaagtcatccttggctg	vvi-miR169f_perfect match probe
vvi|miR-28|2mut|as|	tcggaaagtcatccttcgctg	vvi-miR169f_mismatch probe
vvi|miR-29|nat|as|	ggcaagtcatccttggctg	vvi-miR169g_perfect match probe
vvi|miR-29|2mut|as|	ggcaagccatccttcgctg	vvi-miR169g_mismatch probe
vvi|miR-30|nat|as|	acggccagtcatccttggctc	vvi-miR169i_perfect match probe
vvi|miR-30|2mut|as|	acggtcagtcatccttcgctc	vvi-miR169i_mismatch probe
vvi|miR-31|nat|as|	acggcaagtcatccttggctc	vvi-miR169l_perfect match probe
vvi|miR-31|2mut|as|	acggaaagtcatccttcgctc	vvi-miR169l_mismatch probe
vvi|miR-32|nat|as|	ccggcaagtcatccttggctc	vvi-miR169m_perfect match probe
vvi|miR-32|2mut|as|	ccggaaagtcatccttcgctc	vvi-miR169m_mismatch probe
vvi|miR-33|nat|as|	gcggcaagtcatccttggctc	vvi-miR169o_perfect match probe
vvi|miR-33|2mut|as|	gcggaaagtcatccttcgctc	vvi-miR169o_mismatch probe
vvi|miR-34|nat|as|	cggcaagtcatccttgactca	vvi-miR169r_perfect match probe
vvi|miR-34|2mut|as|	cggcaagccattcttgactca	vvi-miR169r_mismatch probe
vvi|miR-35|nat|as|	cggcaagtcatccttgactcg	vvi-miR169s_perfect match probe
vvi|miR-35|2mut|as|	cggcaagccattcttgactcg	vvi-miR169s_mismatch probe
vvi|miR-36|nat|as|	ccggcaattcatccttggctt	vvi-miR169v_perfect match probe
vvi|miR-36|2mut|as|	ccggaaattcatccttcgctt	vvi-miR169v_mismatch probe
vvi|miR-37|nat|as|	taggcaagtcatccttggcta	vvi-miR169x_perfect match probe
vvi|miR-37|2mut|as|	taggaaagtcatccttcgcta	vvi-miR169x_mismatch probe
vvi|miR-38|nat|as|	cgtgatgttgggacggctcaa	vvi-miR171a_perfect match probe
vvi|miR-38|2mut|as|	cgtgatgttgtgacgcctcaa	vvi-miR171a_mismatch probe
vvi|miR-39|nat|as|	gatattgacgcggctcaatca	vvi-miR171b_perfect match probe
vvi|miR-39|2mut|as|	gatattgacgagcctcaatca	vvi-miR171b_mismatch probe
vvi|miR-40|nat|as|	cgtgatattggcacggctcaa	vvi-miR171c_perfect match probe
vvi|miR-40|2mut|as|	cgtgatattgccacgcctcaa	vvi-miR171c_mismatch probe
vvi|miR-41|nat|as|	gatattggcgcggctcaatca	vvi-miR171e_perfect match probe
vvi|miR-41|2mut|as|	gatattggcgagcctcaatca	vvi-miR171e_mismatch probe
vvi|miR-42|nat|as|	agtgatattggcgcggctcaa	vvi-miR171f_perfect match probe
vvi|miR-42|2mut|as|	agtgatattgccgaggctcaa	vvi-miR171f_mismatch probe
vvi|miR-43|nat|as|	ggtgatattggttcggctcaa	vvi-miR171g_perfect match probe
vvi|miR-43|2mut|as|	ggtgatattcgttcgcctcaa	vvi-miR171g_mismatch probe

vvi|miR-44|nat|as|	gatattggcgcggctcaacca	vvi-miR171h_perfect match probe
vvi|miR-44|2mut|as|	gatattggcgagcctcaacca	vvi-miR171h_mismatch probe
vvi|miR-45|nat|as|	gatgatattggcacggctcaa	vvi-miR171i_perfect match probe
vvi|miR-45|2mut|as|	gatgatattgccacgcctcaa	vvi-miR171i_mismatch probe
vvi|miR-46|nat|as|	atgtagcatcatcaagattca	vvi-miR172a_perfect match probe
vvi|miR-46|2mut|as|	atgtagaatcataaagattca	vvi-miR172a_mismatch probe
vvi|miR-47|nat|as|	gtgtagcatcatcaagattca	vvi-miR172b_perfect match probe
vvi|miR-47|2mut|as|	gtgtagaatcataaagattca	vvi-miR172b_mismatch probe
vvi|miR-48|nat|as|	ctgcagcatcatcaagattcc	vvi-miR172c_perfect match probe
vvi|miR-48|2mut|as|	ctgcagaatcataaagattcc	vvi-miR172c_mismatch probe
vvi|miR-49|nat|as|	atgcagcatcatcaagattct	vvi-miR172d_perfect match probe
vvi|miR-49|2mut|as|	atgcagaatcataaagattct	vvi-miR172d_mismatch probe
vvi|miR-50|nat|as|	gggagctcccttcagtccaag	vvi-miR319b_perfect match probe
vvi|miR-50|2mut|as|	gggagctctcttcagttcaag	vvi-miR319b_mismatch probe
vvi|miR-51|nat|as|	aggagctcccttcagtccaaa	vvi-miR319e_perfect match probe
vvi|miR-51|2mut|as|	aggagctctcttcagttcaaa	vvi-miR319e_mismatch probe
vvi|miR-52|nat|as|	gggagctcccttcagtccaat	vvi-miR319g_perfect match probe
vvi|miR-52|2mut|as|	gggagctctcttcagttcaat	vvi-miR319g_mismatch probe
vvi|miR-53|nat|as|	ggcgctatccctcctgagctt	vvi-miR390_perfect match probe
vvi|miR-53|2mut|as|	ggcgctatctcttctgagctt	vvi-miR390_mismatch probe
vvi|miR-54|nat|as|	ggatcaatgcgatccctttgga	vvi-miR393a_perfect match probe
vvi|miR-54|2mut|as|	ggatcaatgagatctctttgga	vvi-miR393a_mismatch probe
vvi|miR-55|nat|as|	ggaggtggacagaatgccaa	vvi-miR394a_perfect match probe
vvi|miR-55|2mut|as|	ggaggtcgacagaatcccaa	vvi-miR394a_mismatch probe
vvi|miR-56|nat|as|	gagttcccccaaacacttcag	vvi-miR395a_perfect match probe
vvi|miR-56|2mut|as|	gagttccctcaaacccttcag	vvi-miR395a_mismatch probe
vvi|miR-57|nat|as|	gagttcctccagactcttcag	vvi-miR395n_perfect match probe
vvi|miR-57|2mut|as|	gagttcctcaagcctcttcag	vvi-miR395n_mismatch probe
vvi|miR-58|nat|as|	aagctcaagaaagctgtgggag	vvi-miR396a_perfect match probe
vvi|miR-58|2mut|as|	aagctaaagaaagttgtgggag	vvi-miR396a_mismatch probe
vvi|miR-59|nat|as|	aagttcaagaaagctgtgggaa	vvi-miR396b_perfect match probe
vvi|miR-59|2mut|as|	aagttaaagaaagttgtgggaa	vvi-miR396b_mismatch probe
vvi|miR-60|nat|as|	cagttcaagaaagctgtggaa	vvi-miR396c_perfect match probe
vvi|miR-60|2mut|as|	cagttaaagaaagttgtggaa	vvi-miR396c_mismatch probe

vvi|miR-61|nat|as|	catcaacgctgcactcaatga	vvi-miR397a_perfect match probe
vvi|miR-61|2mut|as|	catcaaccctgaactcaatga	vvi-miR397a_mismatch probe
vvi|miR-62|nat|as|	aaggggtgacctgagaacaca	vvi-miR398a_perfect match probe
vvi|miR-62|2mut|as|	aaggtgtgacccgagaacaca	vvi-miR398a_mismatch probe
vvi|miR-63|nat|as|	caggggcgacctgagaacaca	vvi-miR398b_perfect match probe
vvi|miR-63|2mut|as|	caggggagacccgagaacaca	vvi-miR398b_mismatch probe
vvi|miR-64|nat|as|	cagggcaattctcctttggca	vvi-miR399a_perfect match probe
vvi|miR-64|2mut|as|	caggccaattcttctttggca	vvi-miR399a_mismatch probe
vvi|miR-65|nat|as|	cagggcaactctcctttggca	vvi-miR399b_perfect match probe
vvi|miR-65|2mut|as|	caggccaactcttctttggca	vvi-miR399b_mismatch probe
vvi|miR-66|nat|as|	acgagcaaatctcctttggca	vvi-miR399d_perfect match probe
vvi|miR-66|2mut|as|	acgagaaaatcttctttggca	vvi-miR399d_mismatch probe
vvi|miR-67|nat|as|	ccgggcaaatctcctttggca	vvi-miR399e_perfect match probe
vvi|miR-67|2mut|as|	ccggccaaatcttctttggca	vvi-miR399e_mismatch probe
vvi|miR-68|nat|as|	caggacaaatctccttcggca	vvi-miR399f_perfect match probe
vvi|miR-68|2mut|as|	caggaaaaatctccttaggca	vvi-miR399f_mismatch probe
vvi|miR-69|nat|as|	aggggcaaatctcctttggca	vvi-miR399g_perfect match probe
vvi|miR-69|2mut|as|	agggccaaatcttctttggca	vvi-miR399g_mismatch probe
vvi|miR-70|nat|as|	cagggcaactctcctttggcg	vvi-miR399i_perfect match probe
vvi|miR-70|2mut|as|	caggccaactcttctttggcg	vvi-miR399i_mismatch probe
vvi|miR-71|nat|as|	cgagtttgtgcgtgaatctaa	vvi-miR403a_perfect match probe
vvi|miR-71|2mut|as|	cgagtttgtgactgaatctaa	vvi-miR403a_mismatch probe
vvi|miR-72|nat|as|	gccagggaagaggcagtgcat	vvi-miR408_perfect match probe
vvi|miR-72|2mut|as|	gccagtgaagagccagtgcat	vvi-miR408_mismatch probe
vvi|miR-73|nat|as|	ttggaagcctttgagggagat	vvi-miR477a_perfect match probe
vvi|miR-73|2mut|as|	ttggaagtctttgagtgagat	vvi-miR477a_mismatch probe
vvi|miR-74|nat|as|	gatgagccgaaccaataccaca	vvi-miR479_perfect match probe
vvi|miR-74|2mut|as|	gatgagtcgaacaaataccaca	vvi-miR479_mismatch probe
vvi|miR-75|nat|as|	ggaatgggaggagtagg	vvi-miR482a_perfect match probe
vvi|miR-75|2mut|as|	ggaatgtgagaagtagg	vvi-miR482a_mismatch probe
vvi|miR-76|nat|as|	agcgtgctctctctcgttgtca	vvi-miR535a_perfect match probe
vvi|miR-76|2mut|as|	agcgtcctctctctccttgtca	vvi-miR535a_mismatch probe
vvi|miR-77|nat|as|	tggaatactcatttgagcaaga	vvi-miR828a_perfect match probe
vvi|miR-77|2mut|as|	tggaatactaatttgagaaaga	vvi-miR828a_mismatch probe

vvi|miR-78|nat|as|	tggaacactcatttgagcaaga	vvi-miR828b_perfect match probe
vvi|miR-78|2mut|as|	tggaaccctcatttgagaaaga	vvi-miR828b_mismatch probe
vvi|miR-79|nat|as|	tatcaattggtatcagagcta	vvi-miR845a_perfect match probe
vvi|miR-79|2mut|as|	tatcaattcgtatccgagcta	vvi-miR845a_mismatch probe
vvi|miR-80|nat|as|	catcaattggtatcagagcct	vvi-miR845c_perfect match probe
vvi|miR-80|2mut|as|	catcaattcgtatccgagcct	vvi-miR845c_mismatch probe
vvi|miR-81|nat|as|	catcaattggtatcagagcca	vvi-miR845d_perfect match probe
vvi|miR-81|2mut|as|	catcaattcgtatccgagcca	vvi-miR845d_mismatch probe
vvi|miR-82|nat|as|	ccctctcttctgtcaacata	vvi-miR156a|-5_perfect match probe
vvi|miR-82|2mut|as|	ccctcccttctctcaacata	vvi-miR156a|-5_mismatch probe
vvi|miR-83|nat|as|	gggttgtgctccctctcttc	vvi-miR156a|+5_perfect match probe
vvi|miR-83|2mut|as|	gggttgtcctctctctcttc	vvi-miR156a|+5_mismatch probe
vvi|miR-84|nat|as|	cactctcttctgtcagtttt	vvi-miR156b|-5_perfect match probe
vvi|miR-84|2mut|as|	cactcccttctctcagtttt	vvi-miR156b|-5_mismatch probe
vvi|miR-85|nat|as|	tctgtgtgctcactctcttc	vvi-miR156b|+5_perfect match probe
vvi|miR-85|2mut|as|	tctgtgtcctccctctcttc	vvi-miR156b|+5_mismatch probe
vvi|miR-86|nat|as|	cactctcttctgtcacctat	vvi-miR156c|-5_perfect match probe
vvi|miR-86|2mut|as|	cactcccttctgtcatctat	vvi-miR156c|-5_mismatch probe
vvi|miR-87|nat|as|	catgtgtgctcactctcttc	vvi-miR156c|+5_perfect match probe
vvi|miR-87|2mut|as|	catgtgtcctccctctcttc	vvi-miR156c|+5_mismatch probe
vvi|miR-88|nat|as|	cactctcttctgtcagtctc	vvi-miR156d|-5_perfect match probe
vvi|miR-88|2mut|as|	cactcccttctctcagtctc	vvi-miR156d|-5_mismatch probe
vvi|miR-89|nat|as|	tgcatgtgctcactctcttc	vvi-miR156d|+5_perfect match probe
vvi|miR-89|2mut|as|	tgcatgtcctccctctcttc	vvi-miR156d|+5_mismatch probe
vvi|miR-90|nat|as|	cactctcctctgtcacctac	vvi-miR156e|-5_perfect match probe
vvi|miR-90|2mut|as|	cactcttctctgtcatctac	vvi-miR156e|-5_mismatch probe
vvi|miR-91|nat|as|	catgagtgctcactctcctc	vvi-miR156e|+5_perfect match probe
vvi|miR-91|2mut|as|	catgagtcctccctctcctc	vvi-miR156e|+5_mismatch probe
vvi|miR-92|nat|as|	tctatcttctgtcaacagaa	vvi-miR156f|-5_perfect match probe
vvi|miR-92|2mut|as|	tctatcttccgtcaaaagaa	vvi-miR156f|-5_mismatch probe
vvi|miR-93|nat|as|	cagttgtgctctctatcttc	vvi-miR156f|+5_perfect match probe
vvi|miR-93|2mut|as|	cagttgtcctccctatcttc	vvi-miR156f|+5_mismatch probe
vvi|miR-94|nat|as|	tctatcttctgtcaacaaca	vvi-miR156g|-5_perfect match probe
vvi|miR-94|2mut|as|	tctatcttccgtcaaaaaca	vvi-miR156g|-5_mismatch probe

vvi|miR-95|nat|as|	catctgtgctctctatcttc	vvi-miR156g|+5_perfect match probe
vvi|miR-95|2mut|as|	catctgtcctccctatcttc	vvi-miR156g|+5_mismatch probe
vvi|miR-96|nat|as|	ctctctcttctgtcattgtg	vvi-miR156h|-5_perfect match probe
vvi|miR-96|2mut|as|	ctctcccttctctcattgtg	vvi-miR156h|-5_mismatch probe
vvi|miR-97|nat|as|	ccagcatgctctctctcttc	vvi-miR156h|+5_perfect match probe
vvi|miR-97|2mut|as|	ccagcatcctctctcccttc	vvi-miR156h|+5_mismatch probe
vvi|miR-98|nat|as|	ctctatcttctgtcaacaac	vvi-miR156i|-5_perfect match probe
vvi|miR-98|2mut|as|	ctctatcttttctcaacaac	vvi-miR156i|-5_mismatch probe
vvi|miR-99|nat|as|	catctgtgctctctatcttc	vvi-miR156i|+5_perfect match probe
vvi|miR-99|2mut|as|	catctgtcctccctatcttc	vvi-miR156i|+5_mismatch probe
vvi|miR-100|nat|as|	tcccttcactccaaggccag	vvi-miR159a|-5_perfect match probe
vvi|miR-100|2mut|as|	tcccttccctccaagcccag	vvi-miR159a|-5_mismatch probe
vvi|miR-101|nat|as|	gttatgagagctcccttcac	vvi-miR159a|+5_perfect match probe
vvi|miR-101|2mut|as|	gttatgagacctctcttcac	vvi-miR159a|+5_mismatch probe
vvi|miR-102|nat|as|	tcccttcactccaaggccag	vvi-miR159b|-5_perfect match probe
vvi|miR-102|2mut|as|	tcccttccctccaagcccag	vvi-miR159b|-5_mismatch probe
vvi|miR-103|nat|as|	ggttatgagagctcccttca	vvi-miR159b|+5_perfect match probe
vvi|miR-103|2mut|as|	ggttataagagctctcttca	vvi-miR159b|+5_mismatch probe
vvi|miR-104|nat|as|	tcccttcaatccaaacaagg	vvi-miR159c|-5_perfect match probe
vvi|miR-104|2mut|as|	tcccttaaatcaaaacaagg	vvi-miR159c|-5_mismatch probe
vvi|miR-105|nat|as|	aggtgtagagctcccttcaa	vvi-miR159c|+5_perfect match probe
vvi|miR-105|2mut|as|	aggtgtagacctctcttcaa	vvi-miR159c|+5_mismatch probe
vvi|miR-106|nat|as|	tcagggagccaggcagatca	vvi-miR160a|-5_perfect match probe
vvi|miR-106|2mut|as|	tcagggagtcagccagatca	vvi-miR160a|-5_mismatch probe
vvi|miR-107|nat|as|	ttagatggcattcagggagc	vvi-miR160a|+5_perfect match probe
vvi|miR-107|2mut|as|	ttagatgccattcagtgagc	vvi-miR160a|+5_mismatch probe
vvi|miR-108|nat|as|	tcagggagccaggcagaaca	vvi-miR160b|-5_perfect match probe
vvi|miR-108|2mut|as|	tcagggagtcagccagaaca	vvi-miR160b|-5_mismatch probe
vvi|miR-109|nat|as|	ttagatggcattcagggagc	vvi-miR160b|+5_perfect match probe
vvi|miR-109|2mut|as|	ttagatgccattcagtgagc	vvi-miR160b|+5_mismatch probe
vvi|miR-110|nat|as|	acagggagccaggcacaacc	vvi-miR160c|-5_perfect match probe
vvi|miR-110|2mut|as|	acagggagtcagccacaacc	vvi-miR160c|-5_mismatch probe
vvi|miR-111|nat|as|	acgtgtggcatacagggagc	vvi-miR160c|+5_perfect match probe
vvi|miR-111|2mut|as|	acgtgtgccatacagtgagc	vvi-miR160c|+5_mismatch probe

vvi|miR-112|nat|as|	acagggagccaggcatgtaa	vvi-miR160d|-5_perfect match probe
vvi|miR-112|2mut|as|	acagggagtcagccatgtaa	vvi-miR160d|-5_mismatch probe
vvi|miR-113|nat|as|	gcaaatggcatacagggagc	vvi-miR160d|+5_perfect match probe
vvi|miR-113|2mut|as|	gcaaatgccatacagtgagc	vvi-miR160d|+5_mismatch probe
vvi|miR-114|nat|as|	tcagggagccaggcagaaca	vvi-miR160e|-5_perfect match probe
vvi|miR-114|2mut|as|	tcagggagtcagccagaaca	vvi-miR160e|-5_mismatch probe
vvi|miR-115|nat|as|	ttagatggcattcagggagc	vvi-miR160e|+5_perfect match probe
vvi|miR-115|2mut|as|	ttagatgccattcagtgagc	vvi-miR160e|+5_mismatch probe
vvi|miR-116|nat|as|	acagggagccaggcatatat	vvi-miR160f|-5_perfect match probe
vvi|miR-116|2mut|as|	acagggagtcagccatatat	vvi-miR160f|-5_mismatch probe
vvi|miR-117|nat|as|	gcaaatggcatacagggagc	vvi-miR160f|+5_perfect match probe
vvi|miR-117|2mut|as|	gcaaatgccatacagtgagc	vvi-miR160f|+5_mismatch probe
vvi|miR-118|nat|as|	gcagaggtttatcgaccgat	vvi-miR162|-5_perfect match probe
vvi|miR-118|2mut|as|	gcagagctttatagaccgat	vvi-miR162|-5_mismatch probe
vvi|miR-119|nat|as|	gaacgctggatgcagaggtt	vvi-miR162|+5_perfect match probe
vvi|miR-119|2mut|as|	gaaccctggatgaagaggtt	vvi-miR162|+5_mismatch probe
vvi|miR-120|nat|as|	tgccctgcttctccaacaag	vvi-miR164a|-5_perfect match probe
vvi|miR-120|2mut|as|	tgccctccttctcaaacaag	vvi-miR164a|-5_mismatch probe
vvi|miR-121|nat|as|	aaatctgcacgtgccctgct	vvi-miR164a|+5_perfect match probe
vvi|miR-121|2mut|as|	aaatctgaacgtgtcctgct	vvi-miR164a|+5_mismatch probe
vvi|miR-122|nat|as|	tgccctgcttctccagcatg	vvi-miR164b|-5_perfect match probe
vvi|miR-122|2mut|as|	tgccctccttctccaccatg	vvi-miR164b|-5_mismatch probe
vvi|miR-123|nat|as|	gatccagcatgtgccctgct	vvi-miR164b|+5_perfect match probe
vvi|miR-123|2mut|as|	gatccagaatgtgtcctgct	vvi-miR164b|+5_mismatch probe
vvi|miR-124|nat|as|	tgccctgcttctccatcaag	vvi-miR164d|-5_perfect match probe
vvi|miR-124|2mut|as|	tgccctccttctcaatcaag	vvi-miR164d|-5_mismatch probe
vvi|miR-125|nat|as|	tgaactgcacgtgccctgct	vvi-miR164d|+5_perfect match probe
vvi|miR-125|2mut|as|	tgaactgaacgtgtcctgct	vvi-miR164d|+5_mismatch probe
vvi|miR-126|nat|as|	tgaagcctggtccgacgaca	vvi-miR166c|-5_perfect match probe
vvi|miR-126|2mut|as|	tgaagtctggtcagacgaca	vvi-miR166c|-5_mismatch probe
vvi|miR-127|nat|as|	aattggggggaatgaagcct	vvi-miR166c|+5_perfect match probe
vvi|miR-127|2mut|as|	aattggggtgaataaagcct	vvi-miR166c|+5_mismatch probe
vvi|miR-128|nat|as|	tgaagcctggtccgagggca	vvi-miR166d|-5_perfect match probe
vvi|miR-128|2mut|as|	tgaagtctggtcagagggca	vvi-miR166d|-5_mismatch probe

vvi|miR-129|nat|as|	gattgaggggaatgaagcct	vvi-miR166d|+5_perfect match probe
vvi|miR-129|2mut|as|	gattgaggtgaataaagcct	vvi-miR166d|+5_mismatch probe
vvi|miR-130|nat|as|	tgaagcctggtccgacgaca	vvi-miR166e|-5_perfect match probe
vvi|miR-130|2mut|as|	tgaagtctggtcagacgaca	vvi-miR166e|-5_mismatch probe
vvi|miR-131|nat|as|	aattgggggaatgaagcct	vvi-miR166e|+5_perfect match probe
vvi|miR-131|2mut|as|	aattggggtgaataaagcct	vvi-miR166e|+5_mismatch probe
vvi|miR-132|nat|as|	tgaagcctggtccgaagctt	vvi-miR166f|-5_perfect match probe
vvi|miR-132|2mut|as|	tgaagtctggtcagaagctt	vvi-miR166f|-5_mismatch probe
vvi|miR-133|nat|as|	tgttgaggggaatgaagcct	vvi-miR166f|+5_perfect match probe
vvi|miR-133|2mut|as|	tgttgaggtgaataaagcct	vvi-miR166f|+5_mismatch probe
vvi|miR-134|nat|as|	tgaagcctggtccgaaatca	vvi-miR166g|-5_perfect match probe
vvi|miR-134|2mut|as|	tgaagtctggtcagaaatca	vvi-miR166g|-5_mismatch probe
vvi|miR-135|nat|as|	ggttgaggggaatgaagcct	vvi-miR166g|+5_perfect match probe
vvi|miR-135|2mut|as|	ggttgaggtgaataaagcct	vvi-miR166g|+5_mismatch probe
vvi|miR-136|nat|as|	tgaagcctggtccgagatca	vvi-miR166h|-5_perfect match probe
vvi|miR-136|2mut|as|	tgaagtctggtcagagatca	vvi-miR166h|-5_mismatch probe
vvi|miR-137|nat|as|	agttgggggaatgaagcct	vvi-miR166h|+5_perfect match probe
vvi|miR-137|2mut|as|	agttggggtgaataaagcct	vvi-miR166h|+5_mismatch probe
vvi|miR-138|nat|as|	tgaagcctggtccgagatca	vvi-miR166a|-5_perfect match probe
vvi|miR-138|2mut|as|	tgaagtctggtcagagatca	vvi-miR166a|-5_mismatch probe
vvi|miR-139|nat|as|	ggtgtcaggaatgaagcctg	vvi-miR166a|+5_perfect match probe
vvi|miR-139|2mut|as|	ggtgtcagaaatgaaccctg	vvi-miR166a|+5_mismatch probe
vvi|miR-140|nat|as|	tgaagcctggtccgacacct	vvi-miR166b|-5_perfect match probe
vvi|miR-140|2mut|as|	tgaagtctggtcagacacct	vvi-miR166b|-5_mismatch probe
vvi|miR-141|nat|as|	tttgagaggaatgaagcctg	vvi-miR166b|+5_perfect match probe
vvi|miR-141|2mut|as|	tttgagagaaatgaaccctg	vvi-miR166b|+5_mismatch probe
vvi|miR-142|nat|as|	atgctggcagcttcaacctc	vvi-miR167a|-5_perfect match probe
vvi|miR-142|2mut|as|	atgctgccagtttcaacctc	vvi-miR167a|-5_mismatch probe
vvi|miR-143|nat|as|	ttcaccagatcatgctggca	vvi-miR167a|+5_perfect match probe
vvi|miR-143|2mut|as|	ttcacaagatcatcctggca	vvi-miR167a|+5_mismatch probe
vvi|miR-144|nat|as|	atgctggcagcttcaactgc	vvi-miR167b|-5_perfect match probe
vvi|miR-144|2mut|as|	atgctgccagtttcaactgc	vvi-miR167b|-5_mismatch probe
vvi|miR-145|nat|as|	aagcttagatcatgctggca	vvi-miR167b|+5_perfect match probe
vvi|miR-145|2mut|as|	aagcttacatcatcctggca	vvi-miR167b|+5_mismatch probe

vvi|miR-146|nat|as|	atgctggcagcttcaactgc	vvi-miR167c|-5_perfect match probe
vvi|miR-146|2mut|as|	atgctgccagtttcaactgc	vvi-miR167c|-5_mismatch probe
vvi|miR-147|nat|as|	agttgagatcatgctggcag	vvi-miR167c|+5_perfect match probe
vvi|miR-147|2mut|as|	agttgagataatcctggcag	vvi-miR167c|+5_mismatch probe
vvi|miR-148|nat|as|	atgctggcagcttcacttat	vvi-miR167d|-5_perfect match probe
vvi|miR-148|2mut|as|	atgctgccagtttcacttat	vvi-miR167d|-5_mismatch probe
vvi|miR-149|nat|as|	aaagctagatcatgctggca	vvi-miR167d|+5_perfect match probe
vvi|miR-149|2mut|as|	aaagttagatcatcctggca	vvi-miR167d|+5_mismatch probe
vvi|miR-150|nat|as|	atgctggcagcttcaactga	vvi-miR167e|-5_perfect match probe
vvi|miR-150|2mut|as|	atgctgccagtttcaactga	vvi-miR167e|-5_mismatch probe
vvi|miR-151|nat|as|	aagtttagatcatgctggca	vvi-miR167e|+5_perfect match probe
vvi|miR-151|2mut|as|	aagtttacatcatcctggca	vvi-miR167e|+5_mismatch probe
vvi|miR-152|nat|as|	acctgcaccaagcgaattag	vvi-miR168|-5_perfect match probe
vvi|miR-152|2mut|as|	acctgaaccaagagaattag	vvi-miR168|-5_mismatch probe
vvi|miR-153|nat|as|	gtcggttcccgacctgcacc	vvi-miR168|+5_perfect match probe
vvi|miR-153|2mut|as|	gtcggttccagacctccacc	vvi-miR168|+5_mismatch probe
vvi|miR-154|nat|as|	agtcatccttggctgcattc	vvi-miR169a|-5_perfect match probe
vvi|miR-154|2mut|as|	agtcattcttgcctgcattc	vvi-miR169a|-5_mismatch probe
vvi|miR-155|nat|as|	aaattccggcaagtcatcct	vvi-miR169a|+5_perfect match probe
vvi|miR-155|2mut|as|	aaattccgccaagccatcct	vvi-miR169a|+5_mismatch probe
vvi|miR-156|nat|as|	agtcatccttcgctaccaaa	vvi-miR169y|-5_perfect match probe
vvi|miR-156|2mut|as|	agtcattcttccctaccaaa	vvi-miR169y|-5_mismatch probe
vvi|miR-157|nat|as|	ggctgtaggcaagtcatcct	vvi-miR169y|+5_perfect match probe
vvi|miR-157|2mut|as|	ggctgtagccaagccatcct	vvi-miR169y|+5_mismatch probe
vvi|miR-158|nat|as|	gccatccttggctcaattcg	vvi-miR169b|-5_perfect match probe
vvi|miR-158|2mut|as|	gccatccttccctcaattcg	vvi-miR169b|-5_mismatch probe
vvi|miR-159|nat|as|	gatgacggcaagccatcctt	vvi-miR169b|+5_perfect match probe
vvi|miR-159|2mut|as|	gatgacgccaagtcatcctt	vvi-miR169b|+5_mismatch probe
vvi|miR-160|nat|as|	agtcatccttggctgcattc	vvi-miR169c|-5_perfect match probe
vvi|miR-160|2mut|as|	agtcattcttgcctgcattc	vvi-miR169c|-5_mismatch probe
vvi|miR-161|nat|as|	catctccggcaagtcatcct	vvi-miR169c|+5_perfect match probe
vvi|miR-161|2mut|as|	catctccgccaagccatcct	vvi-miR169c|+5_mismatch probe
vvi|miR-162|nat|as|	aatcattcttggctgcattc	vvi-miR169d|-5_perfect match probe
vvi|miR-162|2mut|as|	aatcatttttgcctgcattc	vvi-miR169d|-5_mismatch probe

vvi|miR-163|nat|as|	aaattccggcaaatcattct	vvi-miR169d|+5_perfect match probe
vvi|miR-163|2mut|as|	aaattccgccaaataattct	vvi-miR169d|+5_mismatch probe
vvi|miR-164|nat|as|	agtcatccttggctatcaga	vvi-miR169e|-5_perfect match probe
vvi|miR-164|2mut|as|	agtcattcttgcctatcaga	vvi-miR169e|-5_mismatch probe
vvi|miR-165|nat|as|	tagggcaggcaagtcatcct	vvi-miR169e|+5_perfect match probe
vvi|miR-165|2mut|as|	tagggcagccaagccatcct	vvi-miR169e|+5_mismatch probe
vvi|miR-166|nat|as|	agtcatccttggctgcacca	vvi-miR169f|-5_perfect match probe
vvi|miR-166|2mut|as|	agtcattcttgcctgcacca	vvi-miR169f|-5_mismatch probe
vvi|miR-167|nat|as|	tattttcggcaagtcatcct	vvi-miR169f|+5_perfect match probe
vvi|miR-167|2mut|as|	tattttcgccaagccatcct	vvi-miR169f|+5_mismatch probe
vvi|miR-168|nat|as|	agtcatccttggctgcacca	vvi-miR169g|-5_perfect match probe
vvi|miR-168|2mut|as|	agtcattcttgcctgcacca	vvi-miR169g|-5_mismatch probe
vvi|miR-169|nat|as|	gagtcggcaagtcatccttg	vvi-miR169g|+5_perfect match probe
vvi|miR-169|2mut|as|	gagtcgccaagtcattcttg	vvi-miR169g|+5_mismatch probe
vvi|miR-170|nat|as|	gccatccttggctcaattcc	vvi-miR169h|-5_perfect match probe
vvi|miR-170|2mut|as|	gccatccttccctcaattcc	vvi-miR169h|-5_mismatch probe
vvi|miR-171|nat|as|	aaggacggcaagccatcctt	vvi-miR169h|+5_perfect match probe
vvi|miR-171|2mut|as|	aaggacgccaagtcatcctt	vvi-miR169h|+5_mismatch probe
vvi|miR-172|nat|as|	agtcatccttggctcaattc	vvi-miR169i|-5_perfect match probe
vvi|miR-172|2mut|as|	agtcattcttgcctcaattc	vvi-miR169i|-5_mismatch probe
vvi|miR-173|nat|as|	aaatgacggccagtcatcct	vvi-miR169i|+5_perfect match probe
vvi|miR-173|2mut|as|	aaatgacggtaagtcatcct	vvi-miR169i|+5_mismatch probe
vvi|miR-174|nat|as|	agtcatccttggctgcactc	vvi-miR169j|-5_perfect match probe
vvi|miR-174|2mut|as|	agtcattcttgcctgcactc	vvi-miR169j|-5_mismatch probe
vvi|miR-175|nat|as|	gaattccggcaagtcatcct	vvi-miR169j|+5_perfect match probe
vvi|miR-175|2mut|as|	gaattccgccaagccatcct	vvi-miR169j|+5_mismatch probe
vvi|miR-176|nat|as|	agtcatccttggctgcactc	vvi-miR169k|-5_perfect match probe
vvi|miR-176|2mut|as|	agtcattcttgcctgcactc	vvi-miR169k|-5_mismatch probe
vvi|miR-177|nat|as|	gaattccggcaagtcatcct	vvi-miR169k|+5_perfect match probe
vvi|miR-177|2mut|as|	gaattccgccaagccatcct	vvi-miR169k|+5_mismatch probe
vvi|miR-178|nat|as|	agtcatccttggctcaattc	vvi-miR169l|-5_perfect match probe
vvi|miR-178|2mut|as|	agtcattcttgcctcaattc	vvi-miR169l|-5_mismatch probe
vvi|miR-179|nat|as|	aaaggacggcaagtcatcct	vvi-miR169l|+5_perfect match probe
vvi|miR-179|2mut|as|	aaaggacgccaagccatcct	vvi-miR169l|+5_mismatch probe

<table>
<tr><td>vvi|miR-180|nat|as|</td><td>agtcatccttggctcacttc</td><td>vvi-miR169m|-5_perfect match probe</td></tr>
<tr><td>vvi|miR-180|2mut|as|</td><td>agtcattcttgcctcacttc</td><td>vvi-miR169m|-5_mismatch probe</td></tr>
<tr><td></td><td></td><td>vvi-miR169m|+5_perfect match probe</td></tr>
<tr><td>vvi|miR-181|nat|as|</td><td>agctgccggcaagtcatcct</td><td></td></tr>
<tr><td>vvi|miR-181|2mut|as|</td><td>agctgccgccaagccatcct</td><td>vvi-miR169m|+5_mismatch probe</td></tr>
<tr><td>vvi|miR-182|nat|as|</td><td>agtcatccttggctctattc</td><td>vvi-miR169n|-5_perfect match probe</td></tr>
<tr><td>vvi|miR-182|2mut|as|</td><td>agtcattcttgcctctattc</td><td>vvi-miR169n|-5_mismatch probe</td></tr>
<tr><td>vvi|miR-183|nat|as|</td><td>agttgccggcaagtcatcct</td><td>vvi-miR169n|+5_perfect match probe</td></tr>
<tr><td>vvi|miR-183|2mut|as|</td><td>agttgccgccaagccatcct</td><td>vvi-miR169n|+5_mismatch probe</td></tr>
<tr><td>vvi|miR-184|nat|as|</td><td>agtcatccttggctcaaatc</td><td>vvi-miR169o|-5_perfect match probe</td></tr>
<tr><td>vvi|miR-184|2mut|as|</td><td>agtcattcttgcctcaaatc</td><td>vvi-miR169o|-5_mismatch probe</td></tr>
<tr><td>vvi|miR-185|nat|as|</td><td>tgatggcggcaagtcatcct</td><td>vvi-miR169o|+5_perfect match probe</td></tr>
<tr><td>vvi|miR-185|2mut|as|</td><td>tgatggaggcaagccatcct</td><td>vvi-miR169o|+5_mismatch probe</td></tr>
<tr><td>vvi|miR-186|nat|as|</td><td>agtcatccttggctcaattc</td><td>vvi-miR169p|-5_perfect match probe</td></tr>
<tr><td>vvi|miR-186|2mut|as|</td><td>agtcattcttgcctcaattc</td><td>vvi-miR169p|-5_mismatch probe</td></tr>
<tr><td>vvi|miR-187|nat|as|</td><td>agctgccggcaagtcatcct</td><td>vvi-miR169p|+5_perfect match probe</td></tr>
<tr><td>vvi|miR-187|2mut|as|</td><td>agctgccgccaagccatcct</td><td>vvi-miR169p|+5_mismatch probe</td></tr>
<tr><td>vvi|miR-188|nat|as|</td><td>agtcatccttggctctattc</td><td>vvi-miR169q|-5_perfect match probe</td></tr>
<tr><td>vvi|miR-188|2mut|as|</td><td>agtcattcttgcctctattc</td><td>vvi-miR169q|-5_mismatch probe</td></tr>
<tr><td>vvi|miR-189|nat|as|</td><td>aaatgccggcaagtcatcct</td><td>vvi-miR169q|+5_perfect match probe</td></tr>
<tr><td>vvi|miR-189|2mut|as|</td><td>aaatgccgccaagccatcct</td><td>vvi-miR169q|+5_mismatch probe</td></tr>
<tr><td>vvi|miR-190|nat|as|</td><td>gtcatccttgactcaattcc</td><td>vvi-miR169r|-5_perfect match probe</td></tr>
<tr><td>vvi|miR-190|2mut|as|</td><td>gtcattcttgcctcaattcc</td><td>vvi-miR169r|-5_mismatch probe</td></tr>
<tr><td>vvi|miR-191|nat|as|</td><td>tatatcggcaagtcatcctt</td><td>vvi-miR169r|+5_perfect match probe</td></tr>
<tr><td>vvi|miR-191|2mut|as|</td><td>tatatcgccaagccatcctt</td><td>vvi-miR169r|+5_mismatch probe</td></tr>
<tr><td>vvi|miR-192|nat|as|</td><td>agtcatccttggctgcaatc</td><td>vvi-miR169s|-5_perfect match probe</td></tr>
<tr><td>vvi|miR-192|2mut|as|</td><td>agtcattcttgcctgcaatc</td><td>vvi-miR169s|-5_mismatch probe</td></tr>
<tr><td>vvi|miR-193|nat|as|</td><td>aagtgccggcaagtcatcct</td><td>vvi-miR169s|+5_perfect match probe</td></tr>
<tr><td>vvi|miR-193|2mut|as|</td><td>aagtgccgccaagccatcct</td><td>vvi-miR169s|+5_mismatch probe</td></tr>
<tr><td>vvi|miR-194|nat|as|</td><td>gtcatccttgactcgattcc</td><td>vvi-miR169t|-5_perfect match probe</td></tr>
<tr><td>vvi|miR-194|2mut|as|</td><td>gtcattcttgactagattcc</td><td>vvi-miR169t|-5_mismatch probe</td></tr>
<tr><td>vvi|miR-195|nat|as|</td><td>tatatcggcaagtcatcctt</td><td>vvi-miR169t|+5_perfect match probe</td></tr>
<tr><td>vvi|miR-195|2mut|as|</td><td>tatatcgccaagccatcctt</td><td>vvi-miR169t|+5_mismatch probe</td></tr>
<tr><td>vvi|miR-196|nat|as|</td><td>gtcatccttgactcaattcc</td><td>vvi-miR169u|-5_perfect match probe</td></tr>
</table>

vvi|miR-196|2mut|as|	gtcattcttgcctcaattcc	vvi-miR169u|-5_mismatch probe
vvi|miR-197|nat|as|	tataacggcaagtcatcctt	vvi-miR169u|+5_perfect match probe
vvi|miR-197|2mut|as|	tataacgccaagccatcctt	vvi-miR169u|+5_mismatch probe
vvi|miR-198|nat|as|	attcatccttggctttgttc	vvi-miR169v|-5_perfect match probe
vvi|miR-198|2mut|as|	attcattcttgcctttgttc	vvi-miR169v|-5_mismatch probe
vvi|miR-199|nat|as|	catcgccggcaattcatcct	vvi-miR169v|+5_perfect match probe
vvi|miR-199|2mut|as|	catccccggcaattaatcct	vvi-miR169v|+5_mismatch probe
vvi|miR-200|nat|as|	agtcatccttggctgcacca	vvi-miR169w|-5_perfect match probe
vvi|miR-200|2mut|as|	agtcattcttgcctgcacca	vvi-miR169w|-5_mismatch probe
vvi|miR-201|nat|as|	agttgccggcaagtcatcct	vvi-miR169w|+5_perfect match probe
vvi|miR-201|2mut|as|	agttgccgccaagccatcct	vvi-miR169w|+5_mismatch probe
vvi|miR-202|nat|as|	agtcatccttggctaccaga	vvi-miR169x|-5_perfect match probe
vvi|miR-202|2mut|as|	agtcattcttgcctaccaga	vvi-miR169x|-5_mismatch probe
vvi|miR-203|nat|as|	ggatttaggcaagtcatcct	vvi-miR169x|+5_perfect match probe
vvi|miR-203|2mut|as|	ggatttagccaagccatcct	vvi-miR169x|+5_mismatch probe
vvi|miR-204|nat|as|	gttgggacggctcaatcaaa	vvi-miR171a|-5_perfect match probe
vvi|miR-204|2mut|as|	gttgggacgcttcaatcaaa	vvi-miR171a|-5_mismatch probe
vvi|miR-205|nat|as|	ggaagcgtgatgttgggacg	vvi-miR171a|+5_perfect match probe
vvi|miR-205|2mut|as|	ggaagagtgatgttgtgacg	vvi-miR171a|+5_mismatch probe
vvi|miR-206|nat|as|	gacgcggctcaatcaaatca	vvi-miR171b|-5_perfect match probe
vvi|miR-206|2mut|as|	gacgaggctcaataaaatca	vvi-miR171b|-5_mismatch probe
vvi|miR-207|nat|as|	aaggagatattgacgcggct	vvi-miR171b|+5_perfect match probe
vvi|miR-207|2mut|as|	aaggcgatattgacgaggct	vvi-miR171b|+5_mismatch probe
vvi|miR-208|nat|as|	attggcacggctcaatcaaa	vvi-miR171c|-5_perfect match probe
vvi|miR-208|2mut|as|	attggcacgcttcaatcaaa	vvi-miR171c|-5_mismatch probe
vvi|miR-209|nat|as|	tgacacgtgatattggcacg	vvi-miR171c|+5_perfect match probe
vvi|miR-209|2mut|as|	tgacacctgatattgccacg	vvi-miR171c|+5_mismatch probe
vvi|miR-210|nat|as|	attggcacggctcaatcaaa	vvi-miR171d|-5_perfect match probe
vvi|miR-210|2mut|as|	attggcacgcttcaatcaaa	vvi-miR171d|-5_mismatch probe
vvi|miR-211|nat|as|	taggacgtgatattggcacg	vvi-miR171d|+5_perfect match probe
vvi|miR-211|2mut|as|	taggacctgatattgccacg	vvi-miR171d|+5_mismatch probe
vvi|miR-212|nat|as|	ggcgcggctcaatcagagat	vvi-miR171e|-5_perfect match probe
vvi|miR-212|2mut|as|	ggcgaggctcaatccgagat	vvi-miR171e|-5_mismatch probe

vvi|miR-213|nat|as|	aaagtgatattggcgcggct	vvi-miR171e|+5_perfect match probe
vvi|miR-213|2mut|as|	aaagcgatattggcgaggct	vvi-miR171e|+5_mismatch probe
vvi|miR-214|nat|as|	attggcgcggctcaatctga	vvi-miR171f|-5_perfect match probe
vvi|miR-214|2mut|as|	attggcgaggttcaatctga	vvi-miR171f|-5_mismatch probe
vvi|miR-215|nat|as|	agaaaagtgatattggcgcg	vvi-miR171f|+5_perfect match probe
vvi|miR-215|2mut|as|	agaaaagcgatattgccgcg	vvi-miR171f|+5_mismatch probe
vvi|miR-216|nat|as|	attggttcggctcaaggagt	vvi-miR171g|-5_perfect match probe
vvi|miR-216|2mut|as|	attggttcgcttcaaggagt	vvi-miR171g|-5_mismatch probe
vvi|miR-217|nat|as|	gctcgggtgatattggttcg	vvi-miR171g|+5_perfect match probe
vvi|miR-217|2mut|as|	gctcaggtgatattcgttcg	vvi-miR171g|+5_mismatch probe
vvi|miR-218|nat|as|	ggcgcggctcaaccaaacat	vvi-miR171h|-5_perfect match probe
vvi|miR-218|2mut|as|	ggcgaggctcaacaaaacat	vvi-miR171h|-5_mismatch probe
vvi|miR-219|nat|as|	gacgggatattggcgcggct	vvi-miR171h|+5_perfect match probe
vvi|miR-219|2mut|as|	gacgtgatattggcgaggct	vvi-miR171h|+5_mismatch probe
vvi|miR-220|nat|as|	attggcacggctcaatcaga	vvi-miR171i|-5_perfect match probe
vvi|miR-220|2mut|as|	attggcacgcttcaatcaga	vvi-miR171i|-5_mismatch probe
vvi|miR-221|nat|as|	aacatgatgatattggcacg	vvi-miR171i|+5_perfect match probe
vvi|miR-221|2mut|as|	aacatgataatattgccacg	vvi-miR171i|+5_mismatch probe
vvi|miR-222|nat|as|	catcatcaagattcacatgc	vvi-miR172a|-5_perfect match probe
vvi|miR-222|2mut|as|	catcataaagattcccatgc	vvi-miR172a|-5_mismatch probe
vvi|miR-223|nat|as|	tgcgcatgtagcatcatcaa	vvi-miR172a|+5_perfect match probe
vvi|miR-223|2mut|as|	tgcgaatgtagaatcatcaa	vvi-miR172a|+5_mismatch probe
vvi|miR-224|nat|as|	catcatcaagattcacatgc	vvi-miR172b|-5_perfect match probe
vvi|miR-224|2mut|as|	catcataaagattcccatgc	vvi-miR172b|-5_mismatch probe
vvi|miR-225|nat|as|	tgcaggtgtagcatcatcaa	vvi-miR172b|+5_perfect match probe
vvi|miR-225|2mut|as|	tgcagctgtagaatcatcaa	vvi-miR172b|+5_mismatch probe
vvi|miR-226|nat|as|	catcatcaagattcccacat	vvi-miR172c|-5_perfect match probe
vvi|miR-226|2mut|as|	catcataaagattctcacat	vvi-miR172c|-5_mismatch probe
vvi|miR-227|nat|as|	tgccgctgcagcatcatcaa	vvi-miR172c|+5_perfect match probe
vvi|miR-227|2mut|as|	tgcccctgcagaatcatcaa	vvi-miR172c|+5_mismatch probe
vvi|miR-228|nat|as|	catcatcaagattctcatct	vvi-miR172d|-5_perfect match probe
vvi|miR-228|2mut|as|	catcataaagattctaatct	vvi-miR172d|-5_mismatch probe
vvi|miR-229|nat|as|	tgcgaatgcagcatcatcaa	vvi-miR172d|+5_perfect match probe
vvi|miR-229|2mut|as|	tgcgaatgaagaatcatcaa	vvi-miR172d|+5_mismatch probe

vvi|miR-230|nat|as|	tcccttcagtccaagacgtg	vvi-miR319b|-5_perfect match probe
vvi|miR-230|2mut|as|	tcccttcagccaaagacgtg	vvi-miR319b|-5_mismatch probe
vvi|miR-231|nat|as|	tacaagggagctcccttcag	vvi-miR319b|+5_perfect match probe
vvi|miR-231|2mut|as|	tacaagtgagctctcttcag	vvi-miR319b|+5_mismatch probe
vvi|miR-232|nat|as|	tcccttcagtccaagcacag	vvi-miR319c|-5_perfect match probe
vvi|miR-232|2mut|as|	tcccttcagcccaagaacag	vvi-miR319c|-5_mismatch probe
vvi|miR-233|nat|as|	gtgaagggagctcccttcag	vvi-miR319c|+5_perfect match probe
vvi|miR-233|2mut|as|	gtgaagtgagctctcttcag	vvi-miR319c|+5_mismatch probe
vvi|miR-234|nat|as|	tcccttcagtccaaaacaag	vvi-miR319e|-5_perfect match probe
vvi|miR-234|2mut|as|	tcccttcagccaaaaacaag	vvi-miR319e|-5_mismatch probe
vvi|miR-235|nat|as|	gaactaggagctcccttcag	vvi-miR319e|+5_perfect match probe
vvi|miR-235|2mut|as|	gaactaggacctctcttcag	vvi-miR319e|+5_mismatch probe
vvi|miR-236|nat|as|	tcccttcagtccaagcacag	vvi-miR319f|-5_perfect match probe
vvi|miR-236|2mut|as|	tcccttcagcccaagaacag	vvi-miR319f|-5_mismatch probe
vvi|miR-237|nat|as|	tagaagggagctcccttcag	vvi-miR319f|+5_perfect match probe
vvi|miR-237|2mut|as|	tagaagtgagctctcttcag	vvi-miR319f|+5_mismatch probe
vvi|miR-238|nat|as|	tcccttcagtccaatgagtg	vvi-miR319g|-5_perfect match probe
vvi|miR-238|2mut|as|	tcccttcagccaaatgagtg	vvi-miR319g|-5_mismatch probe
vvi|miR-239|nat|as|	gccctgggagctcccttcag	vvi-miR319g|+5_perfect match probe
vvi|miR-239|2mut|as|	gccctgtgagctctcttcag	vvi-miR319g|+5_mismatch probe
vvi|miR-240|nat|as|	atccctcctgagcttaacag	vvi-miR390|-5_perfect match probe
vvi|miR-240|2mut|as|	atcccttctgagtttaacag	vvi-miR390|-5_mismatch probe
vvi|miR-241|nat|as|	ctcatggcgctatccctcct	vvi-miR390|+5_perfect match probe
vvi|miR-241|2mut|as|	ctcatggccctatctctcct	vvi-miR390|+5_mismatch probe
vvi|miR-242|nat|as|	tgcgatccctttggaactgt	vvi-miR393a|-5_perfect match probe
vvi|miR-242|2mut|as|	tgcgatctctttcgaactgt	vvi-miR393a|-5_mismatch probe
vvi|miR-243|nat|as|	ccatgggatcaatgcgatcc	vvi-miR393a|+5_perfect match probe
vvi|miR-243|2mut|as|	ccatgtgatcaatgagatcc	vvi-miR393a|+5_mismatch probe
vvi|miR-244|nat|as|	tgcgatccctttggatgcct	vvi-miR393b|-5_perfect match probe
vvi|miR-244|2mut|as|	tgcgatctctttcgatgcct	vvi-miR393b|-5_mismatch probe
vvi|miR-245|nat|as|	gtttgggatcaatgcgatcc	vvi-miR393b|+5_perfect match probe
vvi|miR-245|2mut|as|	gtttgtgatcaatgagatcc	vvi-miR393b|+5_mismatch probe
vvi|miR-246|nat|as|	tggacagaatgccaaaatgg	vvi-miR394a|-5_perfect match probe
vvi|miR-246|2mut|as|	tggaaagaatgtcaaaatgg	vvi-miR394a|-5_mismatch probe

vvi|miR-247|nat|as|	tgcatggaggtggacagaat	vvi-miR394a|+5_perfect match probe
vvi|miR-247|2mut|as|	tgcatcgaggtcgacagaat	vvi-miR394a|+5_mismatch probe
vvi|miR-248|nat|as|	tggacagaatgccaataaac	vvi-miR394b|-5_perfect match probe
vvi|miR-248|2mut|as|	tggaaagaatgtcaataaac	vvi-miR394b|-5_mismatch probe
vvi|miR-249|nat|as|	agatgggaggtggacagaat	vvi-miR394b|+5_perfect match probe
vvi|miR-249|2mut|as|	agatgtgaggtcgacagaat	vvi-miR394b|+5_mismatch probe
vvi|miR-250|nat|as|	tggacagaatgccaaaatgg	vvi-miR394c|-5_perfect match probe
vvi|miR-250|2mut|as|	tggaaagaatgtcaaaatgg	vvi-miR394c|-5_mismatch probe
vvi|miR-251|nat|as|	tatatggaggtggacagaat	vvi-miR394c|+5_perfect match probe
vvi|miR-251|2mut|as|	tatatcgaggtcgacagaat	vvi-miR394c|+5_mismatch probe
vvi|miR-252|nat|as|	ccccaaacacttcagtagga	vvi-miR395a|-5_perfect match probe
vvi|miR-252|2mut|as|	ccccaaacccttccgtagga	vvi-miR395a|-5_mismatch probe
vvi|miR-253|nat|as|	accaggagttcccccaaaca	vvi-miR395a|+5_perfect match probe
vvi|miR-253|2mut|as|	accagaagttccctcaaaca	vvi-miR395a|+5_mismatch probe
vvi|miR-254|nat|as|	ccccaaacacttcagtacgt	vvi-miR395b|-5_perfect match probe
vvi|miR-254|2mut|as|	ccccaaacccttccgtacgt	vvi-miR395b|-5_mismatch probe
vvi|miR-255|nat|as|	accaggagttcccccaaaca	vvi-miR395b|+5_perfect match probe
vvi|miR-255|2mut|as|	accagaagttccctcaaaca	vvi-miR395b|+5_mismatch probe
vvi|miR-256|nat|as|	ccccaaacacttcagtagga	vvi-miR395c|-5_perfect match probe
vvi|miR-256|2mut|as|	ccccaaacccttccgtagga	vvi-miR395c|-5_mismatch probe
vvi|miR-257|nat|as|	accaggagttcccccaaaca	vvi-miR395c|+5_perfect match probe
vvi|miR-257|2mut|as|	accagaagttccctcaaaca	vvi-miR395c|+5_mismatch probe
vvi|miR-258|nat|as|	ccccaaacacttcagtagga	vvi-miR395d|-5_perfect match probe
vvi|miR-258|2mut|as|	ccccaaacccttccgtagga	vvi-miR395d|-5_mismatch probe
vvi|miR-259|nat|as|	accaggagttcccccaaaca	vvi-miR395d|+5_perfect match probe
vvi|miR-259|2mut|as|	accagaagttccctcaaaca	vvi-miR395d|+5_mismatch probe
vvi|miR-260|nat|as|	ccccaaacacttcagtagga	vvi-miR395e|-5_perfect match probe
vvi|miR-260|2mut|as|	ccccaaacccttccgtagga	vvi-miR395e|-5_mismatch probe
vvi|miR-261|nat|as|	accaggagttcccccaaaca	vvi-miR395e|+5_perfect match probe
vvi|miR-261|2mut|as|	accagaagttccctcaaaca	vvi-miR395e|+5_mismatch probe
vvi|miR-262|nat|as|	ccccaaacacttcagtggga	vvi-miR395f|-5_perfect match probe
vvi|miR-262|2mut|as|	ccccaaacccttcagcggga	vvi-miR395f|-5_mismatch probe
vvi|miR-263|nat|as|	accaggagttcccccaaaca	vvi-miR395f|+5_perfect match probe
vvi|miR-263|2mut|as|	accagaagttccctcaaaca	vvi-miR395f|+5_mismatch probe

vvi|miR-264|nat|as|	ccccaaacacttcagtagga	vvi-miR395g|-5_perfect match probe
vvi|miR-264|2mut|as|	ccccaaacccttccgtagga	vvi-miR395g|-5_mismatch probe
vvi|miR-265|nat|as|	accgggagttcccccaaaca	vvi-miR395g|+5_perfect match probe
vvi|miR-265|2mut|as|	accgtgagttccctcaaaca	vvi-miR395g|+5_mismatch probe
vvi|miR-266|nat|as|	ccccaaacacttcagtagga	vvi-miR395h|-5_perfect match probe
vvi|miR-266|2mut|as|	ccccaaacccttccgtagga	vvi-miR395h|-5_mismatch probe
vvi|miR-267|nat|as|	accaggagttcccccaaaca	vvi-miR395h|+5_perfect match probe
vvi|miR-267|2mut|as|	accagaagttccctcaaaca	vvi-miR395h|+5_mismatch probe
vvi|miR-268|nat|as|	ccccaaacacttcagtagga	vvi-miR395i|-5_perfect match probe
vvi|miR-268|2mut|as|	ccccaaacccttccgtagga	vvi-miR395i|-5_mismatch probe
vvi|miR-269|nat|as|	accaggagttcccccaaaca	vvi-miR395i|+5_perfect match probe
vvi|miR-269|2mut|as|	accagaagttccctcaaaca	vvi-miR395i|+5_mismatch probe
vvi|miR-270|nat|as|	ccccaaacacttcagtagga	vvi-miR395j|-5_perfect match probe
vvi|miR-270|2mut|as|	ccccaaacccttccgtagga	vvi-miR395j|-5_mismatch probe
vvi|miR-271|nat|as|	accaggagttcccccaaaca	vvi-miR395j|+5_perfect match probe
vvi|miR-271|2mut|as|	accagaagttccctcaaaca	vvi-miR395j|+5_mismatch probe
vvi|miR-272|nat|as|	ccccaaacacttcagtagga	vvi-miR395k|-5_perfect match probe
vvi|miR-272|2mut|as|	ccccaaacccttccgtagga	vvi-miR395k|-5_mismatch probe
vvi|miR-273|nat|as|	accaggagttcccccaaaca	vvi-miR395k|+5_perfect match probe
vvi|miR-273|2mut|as|	accagaagttccctcaaaca	vvi-miR395k|+5_mismatch probe
vvi|miR-274|nat|as|	ccccaaacacttcagtagga	vvi-miR395l|-5_perfect match probe
vvi|miR-274|2mut|as|	ccccaaacccttccgtagga	vvi-miR395l|-5_mismatch probe
vvi|miR-275|nat|as|	accaggagttcccccaaaca	vvi-miR395l|+5_perfect match probe
vvi|miR-275|2mut|as|	accagaagttccctcaaaca	vvi-miR395l|+5_mismatch probe
vvi|miR-276|nat|as|	ccccaaacacttcagtagga	vvi-miR395m|-5_perfect match probe
vvi|miR-276|2mut|as|	ccccaaacccttccgtagga	vvi-miR395m|-5_mismatch probe
vvi|miR-277|nat|as|	accaggagttcccccaaaca	vvi-miR395m|+5_perfect match probe
vvi|miR-277|2mut|as|	accagaagttccctcaaaca	vvi-miR395m|+5_mismatch probe
vvi|miR-278|nat|as|	ctccagactcttcagtagag	vvi-miR395n|-5_perfect match probe
vvi|miR-278|2mut|as|	ctccagcctcttccgtagag	vvi-miR395n|-5_mismatch probe
vvi|miR-279|nat|as|	accaagagttcctccagact	vvi-miR395n|+5_perfect match probe
vvi|miR-279|2mut|as|	accaagacttcctcaagact	vvi-miR395n|+5_mismatch probe
vvi|miR-280|nat|as|	agaaagctgtgggaggacat	vvi-miR396a|-5_perfect match probe

vvi|miR-280|2mut|as|	agaaagttgtgtgaggacat	vvi-miR396a|-5_mismatch probe
vvi|miR-281|nat|as|	aatagaagctcaagaaagct	vvi-miR396a|+5_perfect match probe
vvi|miR-281|2mut|as|	aatagaagttaaagaaagct	vvi-miR396a|+5_mismatch probe
vvi|miR-282|nat|as|	agaaagctgtgggaaaccat	vvi-miR396b|-5_perfect match probe
vvi|miR-282|2mut|as|	agaaagttgtgtgaaaccat	vvi-miR396b|-5_mismatch probe
vvi|miR-283|nat|as|	cactgaagttcaagaaagct	vvi-miR396b|+5_perfect match probe
vvi|miR-283|2mut|as|	cactaaagttaaagaaagct	vvi-miR396b|+5_mismatch probe
vvi|miR-284|nat|as|	aagaaagctgtggaagatca	vvi-miR396c|-5_perfect match probe
vvi|miR-284|2mut|as|	aagaaagttgtcgaagatca	vvi-miR396c|-5_mismatch probe
vvi|miR-285|nat|as|	caaaacagttcaagaaagct	vvi-miR396c|+5_perfect match probe
vvi|miR-285|2mut|as|	caaaaaagttaaagaaagct	vvi-miR396c|+5_mismatch probe
vvi|miR-286|nat|as|	aagaaagctgtggaagaata	vvi-miR396d|-5_perfect match probe
vvi|miR-286|2mut|as|	aagaaagttgtcgaagaata	vvi-miR396d|-5_mismatch probe
vvi|miR-287|nat|as|	aaatgcagttcaagaaagct	vvi-miR396d|+5_perfect match probe
vvi|miR-287|2mut|as|	aaatgaagttaaagaaagct	vvi-miR396d|+5_mismatch probe
vvi|miR-288|nat|as|	cgctgcactcaatgatgttt	vvi-miR397a|-5_perfect match probe
vvi|miR-288|2mut|as|	cgctgaactcaataatgttt	vvi-miR397a|-5_mismatch probe
vvi|miR-289|nat|as|	agtttcatcaacgctgcact	vvi-miR397a|+5_perfect match probe
vvi|miR-289|2mut|as|	agtttcataaaccctgcact	vvi-miR397a|+5_mismatch probe
vvi|miR-290|nat|as|	cgctgcactcaatgatgttt	vvi-miR397b|-5_perfect match probe
vvi|miR-290|2mut|as|	cgctgaactcaataatgttt	vvi-miR397b|-5_mismatch probe
vvi|miR-291|nat|as|	agtttcatcaacgctgcact	vvi-miR397b|+5_perfect match probe
vvi|miR-291|2mut|as|	agtttcataaaccctgcact	vvi-miR397b|+5_mismatch probe
vvi|miR-292|nat|as|	tgacctgagaacacaaaatg	vvi-miR398a|-5_perfect match probe
vvi|miR-292|2mut|as|	tgacttgagaacccaaaatg	vvi-miR398a|-5_mismatch probe
vvi|miR-293|nat|as|	ccccaaaggggtgacctgag	vvi-miR398a|+5_perfect match probe
vvi|miR-293|2mut|as|	ccccaaaggtgtgatctgag	vvi-miR398a|+5_mismatch probe
vvi|miR-294|nat|as|	cgacctgagaacacatgaga	vvi-miR398b|-5_perfect match probe
vvi|miR-294|2mut|as|	cgacttgagaacccatgaga	vvi-miR398b|-5_mismatch probe
vvi|miR-295|nat|as|	tccagcaggggcgacctgag	vvi-miR398b|+5_perfect match probe
vvi|miR-295|2mut|as|	tccagcaggtgagacctgag	vvi-miR398b|+5_mismatch probe
vvi|miR-296|nat|as|	cgacctgagaacacatgaga	vvi-miR398c|-5_perfect match probe
vvi|miR-296|2mut|as|	cgacttgagaacccatgaga	vvi-miR398c|-5_mismatch probe
vvi|miR-297|nat|as|	tccagcaggggcgacctgag	vvi-miR398c|+5_perfect match probe

vvi|miR-297|2mut|as|	tccagcaggtgagacctgag	vvi-miR398c|+5_mismatch probe
vvi|miR-298|nat|as|	aattctcctttggcagagca	vvi-miR399a|-5_perfect match probe
vvi|miR-298|2mut|as|	aattcttctttgccagagca	vvi-miR399a|-5_mismatch probe
vvi|miR-299|nat|as|	aataacagggcaattctcct	vvi-miR399a|+5_perfect match probe
vvi|miR-299|2mut|as|	aataacaggcaaattctcct	vvi-miR399a|+5_mismatch probe
vvi|miR-300|nat|as|	aactctcctttggcaagtca	vvi-miR399b|-5_perfect match probe
vvi|miR-300|2mut|as|	aactcttctttgccaagtca	vvi-miR399b|-5_mismatch probe
vvi|miR-301|nat|as|	agtcacagggcaactctcct	vvi-miR399b|+5_perfect match probe
vvi|miR-301|2mut|as|	agtcacaggcaaactctcct	vvi-miR399b|+5_mismatch probe
vvi|miR-302|nat|as|	aactctcctttggcagatca	vvi-miR399c|-5_perfect match probe
vvi|miR-302|2mut|as|	aactcttctttgccagatca	vvi-miR399c|-5_mismatch probe
vvi|miR-303|nat|as|	agacacagggcaactctcct	vvi-miR399c|+5_perfect match probe
vvi|miR-303|2mut|as|	agacacaggcaaactctcct	vvi-miR399c|+5_mismatch probe
vvi|miR-304|nat|as|	aaatctcctttggcagacac	vvi-miR399d|-5_perfect match probe
vvi|miR-304|2mut|as|	aaatcttctttgccagacac	vvi-miR399d|-5_mismatch probe
vvi|miR-305|nat|as|	aattcacgagcaaatctcct	vvi-miR399d|+5_perfect match probe
vvi|miR-305|2mut|as|	aattcaagagaaaatctcct	vvi-miR399d|+5_mismatch probe
vvi|miR-306|nat|as|	aaatctcctttggcagaggg	vvi-miR399e|-5_perfect match probe
vvi|miR-306|2mut|as|	aaatcttctttgccagaggg	vvi-miR399e|-5_mismatch probe
vvi|miR-307|nat|as|	aattgccgggcaaatctcct	vvi-miR399e|+5_perfect match probe
vvi|miR-307|2mut|as|	aattgccggcaaaatctcct	vvi-miR399e|+5_mismatch probe
vvi|miR-308|nat|as|	aaatctccttcggcagcgag	vvi-miR399f|-5_perfect match probe
vvi|miR-308|2mut|as|	aaatcttcttcgccagcgag	vvi-miR399f|-5_mismatch probe
vvi|miR-309|nat|as|	aattgcaggacaaatctcct	vvi-miR399f|+5_perfect match probe
vvi|miR-309|2mut|as|	aattgaaggaaaaatctcct	vvi-miR399f|+5_mismatch probe
vvi|miR-310|nat|as|	aaatctcctttggcagtgag	vvi-miR399g|-5_perfect match probe
vvi|miR-310|2mut|as|	aaatcttctttgccagtgag	vvi-miR399g|-5_mismatch probe
vvi|miR-311|nat|as|	aattgaggggcaaatctcct	vvi-miR399g|+5_perfect match probe
vvi|miR-311|2mut|as|	aattgagggcaaaatctcct	vvi-miR399g|+5_mismatch probe
vvi|miR-312|nat|as|	aattctcctttggcaaaaca	vvi-miR399h|-5_perfect match probe
vvi|miR-312|2mut|as|	aattcttctttgccaaaaca	vvi-miR399h|-5_mismatch probe
vvi|miR-313|nat|as|	aatggcagggcaattctcct	vvi-miR399h|+5_perfect match probe
vvi|miR-313|2mut|as|	aatggcaggcaaattctcct	vvi-miR399h|+5_mismatch probe
vvi|miR-314|nat|as|	aactctcctttggcgggtca	vvi-miR399i|-5_perfect match probe

vvi|miR-314|2mut|as|	aactcttctttggagggtca	vvi-miR399i|-5_mismatch probe
vvi|miR-315|nat|as|	agtcacagggcaactctcct	vvi-miR399i|+5_perfect match probe
vvi|miR-315|2mut|as|	agtcacaggcaaactctcct	vvi-miR399i|+5_mismatch probe
vvi|miR-316|nat|as|	tgtgcgtgaatctaacgccg	vvi-miR403a|-5_perfect match probe
vvi|miR-316|2mut|as|	tgtgagtgaatctaaagccg	vvi-miR403a|-5_mismatch probe
vvi|miR-317|nat|as|	actcacgagtttgtgcgtga	vvi-miR403a|+5_perfect match probe
vvi|miR-317|2mut|as|	actcaagagtttgtgagtga	vvi-miR403a|+5_mismatch probe
vvi|miR-318|nat|as|	tgtgcgtgaatctaacaccc	vvi-miR403b|-5_perfect match probe
vvi|miR-318|2mut|as|	tgtgagtgaatctaaaaccc	vvi-miR403b|-5_mismatch probe
vvi|miR-319|nat|as|	gatcccgagtttgtgcgtga	vvi-miR403b|+5_perfect match probe
vvi|miR-319|2mut|as|	gatccagagtttgtgagtga	vvi-miR403b|+5_mismatch probe
vvi|miR-320|nat|as|	tgtgcgtgaatctaacggcg	vvi-miR403c|-5_perfect match probe
vvi|miR-320|2mut|as|	tgtgagtgaatctaaaggcg	vvi-miR403c|-5_mismatch probe
vvi|miR-321|nat|as|	gatcacgagtttgtgcgtga	vvi-miR403c|+5_perfect match probe
vvi|miR-321|2mut|as|	gatcaagagtttgtgagtga	vvi-miR403c|+5_mismatch probe
vvi|miR-322|nat|as|	tgtgcgtgaatctaacaccc	vvi-miR403d|-5_perfect match probe
vvi|miR-322|2mut|as|	tgtgagtgaatctaaaaccc	vvi-miR403d|-5_mismatch probe
vvi|miR-323|nat|as|	gatcccgagtttgtgcgtga	vvi-miR403d|+5_perfect match probe
vvi|miR-323|2mut|as|	gatccagagtttgtgagtga	vvi-miR403d|+5_mismatch probe
vvi|miR-324|nat|as|	tgtgcgtgaatctaacggcg	vvi-miR403e|-5_perfect match probe
vvi|miR-324|2mut|as|	tgtgagtgaatctaaaggcg	vvi-miR403e|-5_mismatch probe
vvi|miR-325|nat|as|	gatcgcgagtttgtgcgtga	vvi-miR403e|+5_perfect match probe
vvi|miR-325|2mut|as|	gatcgagagtttgtgagtga	vvi-miR403e|+5_mismatch probe
vvi|miR-326|nat|as|	tgtgcgtgaatctaagacca	vvi-miR403f|-5_perfect match probe
vvi|miR-326|2mut|as|	tgtgagtgaatctaacacca	vvi-miR403f|-5_mismatch probe
vvi|miR-327|nat|as|	gattacgagtttgtgcgtga	vvi-miR403f|+5_perfect match probe
vvi|miR-327|2mut|as|	gattaagagtttgtgagtga	vvi-miR403f|+5_mismatch probe
vvi|miR-328|nat|as|	gaagaggcagtgcatgggta	vvi-miR408|-5_perfect match probe
vvi|miR-328|2mut|as|	gaagagccagtgaatgggta	vvi-miR408|-5_mismatch probe
vvi|miR-329|nat|as|	acagagccagggaagaggca	vvi-miR408|+5_perfect match probe
vvi|miR-329|2mut|as|	acagagtcagtgaagaggca	vvi-miR408|+5_mismatch probe
vvi|miR-330|nat|as|	gcctttgagggagattgaca	vvi-miR477a|-5_perfect match probe
vvi|miR-330|2mut|as|	gcctttgagtaagattgaca	vvi-miR477a|-5_mismatch probe
vvi|miR-331|nat|as|	gaaaattggaagcctttgag	vvi-miR477a|+5_perfect match probe

vvi|miR-331|2mut|as|	gaaaattcgaagtctttgag	vvi-miR477a|+5_mismatch probe
vvi|miR-332|nat|as|	cgaaccaataccacaccatg	vvi-miR479|-5_perfect match probe
vvi|miR-332|2mut|as|	cgaacaaatacaacaccatg	vvi-miR479|-5_mismatch probe
vvi|miR-333|nat|as|	aagaagatgagccgaaccaa	vvi-miR479|+5_perfect match probe
vvi|miR-333|2mut|as|	aagaagataagtcgaaccaa	vvi-miR479|+5_mismatch probe
vvi|miR-334|nat|as|	aatgggaggagtaggaaaga	vvi-miR482a|-5_perfect match probe
vvi|miR-334|2mut|as|	aatgtgaggagtagaaaaga	vvi-miR482a|-5_mismatch probe
vvi|miR-335|nat|as|	caataggaatgggaggagta	vvi-miR482a|+5_perfect match probe
vvi|miR-335|2mut|as|	caatagaaatgtgaggagta	vvi-miR482a|+5_mismatch probe
vvi|miR-336|nat|as|	tctctctcgttgtcaagcaa	vvi-miR535a|-5_perfect match probe
vvi|miR-336|2mut|as|	tctctctccttctcaagcaa	vvi-miR535a|-5_mismatch probe
vvi|miR-337|nat|as|	tgactagcgtgctctctctc	vvi-miR535a|+5_perfect match probe
vvi|miR-337|2mut|as|	tgactagagtcctctctctc	vvi-miR535a|+5_mismatch probe
vvi|miR-338|nat|as|	tctctctcgttgtcaagcaa	vvi-miR535b|-5_perfect match probe
vvi|miR-338|2mut|as|	tctctctccttctcaagcaa	vvi-miR535b|-5_mismatch probe
vvi|miR-339|nat|as|	tgactagcgtgctctctctc	vvi-miR535b|+5_perfect match probe
vvi|miR-339|2mut|as|	tgactagagtcctctctctc	vvi-miR535b|+5_mismatch probe
vvi|miR-340|nat|as|	tctctctcgttgtcaagcaa	vvi-miR535c|-5_perfect match probe
vvi|miR-340|2mut|as|	tctctctccttctcaagcaa	vvi-miR535c|-5_mismatch probe
vvi|miR-341|nat|as|	tgactagcgtgctctctctc	vvi-miR535c|+5_perfect match probe
vvi|miR-341|2mut|as|	tgactagagtcctctctctc	vvi-miR535c|+5_mismatch probe
vvi|miR-342|nat|as|	tctctctcgttgtcaagcaa	vvi-miR535d|-5_perfect match probe
vvi|miR-342|2mut|as|	tctctctccttctcaagcaa	vvi-miR535d|-5_mismatch probe
vvi|miR-343|nat|as|	agactagcgtgctctctctc	vvi-miR535d|+5_perfect match probe
vvi|miR-343|2mut|as|	agactagagtcctctctctc	vvi-miR535d|+5_mismatch probe
vvi|miR-344|nat|as|	tctctctcgttgtcaagcaa	vvi-miR535e|-5_perfect match probe
vvi|miR-344|2mut|as|	tctctctccttctcaagcaa	vvi-miR535e|-5_mismatch probe
vvi|miR-345|nat|as|	agactagcgtgctctctctc	vvi-miR535e|+5_perfect match probe
vvi|miR-345|2mut|as|	agactagagtcctctctctc	vvi-miR535e|+5_mismatch probe
vvi|miR-346|nat|as|	ctcatttgagcaagaaactt	vvi-miR828a|-5_perfect match probe
vvi|miR-346|2mut|as|	ctcatttgacaaagaaactt	vvi-miR828a|-5_mismatch probe
vvi|miR-347|nat|as|	ttgtttggaatactcatttg	vvi-miR828a|+5_perfect match probe
vvi|miR-347|2mut|as|	ttgtttcgaatactaatttg	vvi-miR828a|+5_mismatch probe
vvi|miR-348|nat|as|	ctcatttgagcaagaaacca	vvi-miR828b|-5_perfect match probe

<table>
<tr><td>vvi|miR-348|2mut|as|</td><td>ctcatttgacaaagaaacca</td><td>vvi-miR828b|-5_mismatch probe</td></tr>
<tr><td>vvi|miR-349|nat|as|</td><td>tgatgtggaacactcatttg</td><td>vvi-miR828b|+5_perfect match probe</td></tr>
<tr><td>vvi|miR-349|2mut|as|</td><td>tgatgtcgaaccctcatttg</td><td>vvi-miR828b|+5_mismatch probe</td></tr>
<tr><td>vvi|miR-350|nat|as|</td><td>ttggtatcagagctagacct</td><td>vvi-miR845a|-5_perfect match probe</td></tr>
<tr><td>vvi|miR-350|2mut|as|</td><td>ttggtatccgagttagacct</td><td>vvi-miR845a|-5_mismatch probe</td></tr>
<tr><td>vvi|miR-351|nat|as|</td><td>tgttttatcaattggtatca</td><td>vvi-miR845a|+5_perfect match probe</td></tr>
<tr><td>vvi|miR-351|2mut|as|</td><td>tgttttataaattcgtatca</td><td>vvi-miR845a|+5_mismatch probe</td></tr>
<tr><td>vvi|miR-352|nat|as|</td><td>ttggtatcagagctagacct</td><td>vvi-miR845b|-5_perfect match probe</td></tr>
<tr><td>vvi|miR-352|2mut|as|</td><td>ttggtatccgagttagacct</td><td>vvi-miR845b|-5_mismatch probe</td></tr>
<tr><td>vvi|miR-353|nat|as|</td><td>tgttttatcaattggtatca</td><td>vvi-miR845b|+5_perfect match probe</td></tr>
<tr><td>vvi|miR-353|2mut|as|</td><td>tgttttataaattcgtatca</td><td>vvi-miR845b|+5_mismatch probe</td></tr>
<tr><td>vvi|miR-354|nat|as|</td><td>ttggtatcagagcctcatgt</td><td>vvi-miR845c|-5_perfect match probe</td></tr>
<tr><td>vvi|miR-354|2mut|as|</td><td>ttggtatccgagtctcatgt</td><td>vvi-miR845c|-5_mismatch probe</td></tr>
<tr><td>vvi|miR-355|nat|as|</td><td>ttatgcatcaattggtatca</td><td>vvi-miR845c|+5_perfect match probe</td></tr>
<tr><td>vvi|miR-355|2mut|as|</td><td>ttatgaatcaattcgtatca</td><td>vvi-miR845c|+5_mismatch probe</td></tr>
<tr><td>vvi|miR-356|nat|as|</td><td>ttggtatcagagccataggt</td><td>vvi-miR845d|-5_perfect match probe</td></tr>
<tr><td>vvi|miR-356|2mut|as|</td><td>ttggtatccgagtcataggt</td><td>vvi-miR845d|-5_mismatch probe</td></tr>
<tr><td>vvi|miR-357|nat|as|</td><td>tttcccatcaattggtatca</td><td>vvi-miR845d|+5_perfect match probe</td></tr>
<tr><td>vvi|miR-357|2mut|as|</td><td>tttctcatcaattcgtatca</td><td>vvi-miR845d|+5_mismatch probe</td></tr>
<tr><td>vvi|miR-358|nat|as|</td><td>ttggtatcagagccataggt</td><td>vvi-miR845e|-5_perfect match probe</td></tr>
<tr><td>vvi|miR-358|2mut|as|</td><td>ttggtatccgagtcataggt</td><td>vvi-miR845e|-5_mismatch probe</td></tr>
<tr><td>vvi|miR-359|nat|as|</td><td>tttcccatcaattggtatca</td><td>vvi-miR845e|+5_perfect match probe</td></tr>
<tr><td>vvi|miR-359|2mut|as|</td><td>tttctcatcaattcgtatca</td><td>vvi-miR845e|+5_mismatch probe</td></tr>
<tr><td>vvi|miR-360|nat|as|</td><td>tcttctgtcaacatatttct</td><td>vvi-miR156a|-10_perfect match probe</td></tr>
<tr><td>vvi|miR-360|2mut|as|</td><td>tcttctctcaaaatatttct</td><td>vvi-miR156a|-10_mismatch probe</td></tr>
<tr><td>vvi|miR-361|nat|as|</td><td>atgctgggttgtgctccctc</td><td>vvi-miR156a|+10_perfect match probe</td></tr>
<tr><td>vvi|miR-361|2mut|as|</td><td>atgctgtgttgtcctccctc</td><td>vvi-miR156a|+10_mismatch probe</td></tr>
<tr><td>vvi|miR-362|nat|as|</td><td>tcttctgtcagttttcaatg</td><td>vvi-miR156b|-10_perfect match probe</td></tr>
<tr><td>vvi|miR-362|2mut|as|</td><td>tcttctctcagttttaaatg</td><td>vvi-miR156b|-10_mismatch probe</td></tr>
<tr><td>vvi|miR-363|nat|as|</td><td>ttgcctctgtgtgctcactc</td><td>vvi-miR156b|+10_perfect match probe</td></tr>
</table>

vvi|miR-363|2mut|as|	ttgcttctgtgtcctcactc
vvi|miR-364|nat|as|	tcttctgtcacctattcctt
vvi|miR-364|2mut|as|	tcttctgtcctctattcctt
vvi|miR-365|nat|as|	cagaccatgtgtgctcactc
vvi|miR-365|2mut|as|	cagacaatgtgtcctcactc
vvi|miR-366|nat|as|	tcttctgtcagtctctctct
vvi|miR-366|2mut|as|	tcttctctcagcctctctct
vvi|miR-367|nat|as|	ttgcctgcatgtgctcactc
vvi|miR-367|2mut|as|	ttgcctgaatgtcctcactc
vvi|miR-368|nat|as|	tcctctgtcacctaccatac
vvi|miR-368|2mut|as|	tcctctgtcccctacaatac
vvi|miR-369|nat|as|	agaaccatgagtgctcactc
vvi|miR-369|2mut|as|	agaacaatgagtcctcactc
vvi|miR-370|nat|as|	cttctgtcaacagaacataa
vvi|miR-370|2mut|as|	cttctctcaacagaaaataa
vvi|miR-371|nat|as|	tgctctctatcttctgtcaa
vvi|miR-371|2mut|as|	tgctccctatcttctctcaa
vvi|miR-372|nat|as|	tgttcagttgtgctctctat
vvi|miR-372|2mut|as|	tgttcagttctcctctctat
vvi|miR-373|nat|as|	cttctgtcaacaacatcacc
vvi|miR-373|2mut|as|	cttctctcaacaaaatcacc
vvi|miR-374|nat|as|	tgctctctatcttctgtcaa
vvi|miR-374|2mut|as|	tgctccctatcttctctcaa
vvi|miR-375|nat|as|	tcatcatctgtgctctctat
vvi|miR-375|2mut|as|	tcatcatctctcctctctat

vvi-miR156b|+10_mismatch probe
vvi-miR156c|-10_perfect match probe
vvi-miR156c|-10_mismatch probe
vvi-miR156c|+10_perfect match probe
vvi-miR156c|+10_mismatch probe
vvi-miR156d|-10_perfect match probe
vvi-miR156d|-10_mismatch probe
vvi-miR156d|+10_perfect match probe
vvi-miR156d|+10_mismatch probe
vvi-miR156e|-10_perfect match probe
vvi-miR156e|-10_mismatch probe
vvi-miR156e|+10_perfect match probe
vvi-miR156e|+10_mismatch probe
vvi-miR156f|-10_perfect match probe
vvi-miR156f|-10_mismatch probe
vvi-miR156f|0_perfect match probe
vvi-miR156f|0_mismatch probe
vvi-miR156f|+10_perfect match probe
vvi-miR156f|+10_mismatch probe
vvi-miR156g|-10_perfect match probe
vvi-miR156g|-10_mismatch probe
vvi-miR156g|0_perfect match probe
vvi-miR156g|0_mismatch probe
vvi-miR156g|+10_perfect match probe
vvi-miR156g|+10_mismatch probe

<table>
<tr><td>vvi|miR-376|nat|as|</td><td>tcttctgtcattgtgaggca</td><td>vvi-miR156h|-10_perfect match probe</td></tr>
<tr><td>vvi|miR-376|2mut|as|</td><td>tcttctctcattgcgaggca</td><td>vvi-miR156h|-10_mismatch probe</td></tr>
<tr><td>vvi|miR-377|nat|as|</td><td>tcccaccagcatgctctctc</td><td>vvi-miR156h|+10_perfect match probe</td></tr>
<tr><td>vvi|miR-377|2mut|as|</td><td>tcccaccagaatcctctctc</td><td>vvi-miR156h|+10_mismatch probe</td></tr>
<tr><td>vvi|miR-378|nat|as|</td><td>tcttctgtcaacaacgtcac</td><td>vvi-miR156i|-10_perfect match probe</td></tr>
<tr><td>vvi|miR-378|2mut|as|</td><td>tcttctctcaacaacctcac</td><td>vvi-miR156i|-10_mismatch probe</td></tr>
<tr><td>vvi|miR-379|nat|as|</td><td>ttcatcatctgtgctctcta</td><td>vvi-miR156i|+10_perfect match probe</td></tr>
<tr><td>vvi|miR-379|2mut|as|</td><td>ttcatcatccgtcctctcta</td><td>vvi-miR156i|+10_mismatch probe</td></tr>
<tr><td>vvi|miR-380|nat|as|</td><td>tcactccaaggccagaaaga</td><td>vvi-miR159a|-10_perfect match probe</td></tr>
<tr><td>vvi|miR-380|2mut|as|</td><td>tcactcaaaggtcagaaaga</td><td>vvi-miR159a|-10_mismatch probe</td></tr>
<tr><td>vvi|miR-381|nat|as|</td><td>agagctcccttcactccaag</td><td>vvi-miR159a|0_perfect match probe</td></tr>
<tr><td>vvi|miR-381|2mut|as|</td><td>agagctctcttcacttcaag</td><td>vvi-miR159a|0_mismatch probe</td></tr>
<tr><td>vvi|miR-382|nat|as|</td><td>aagggttatgagagctccct</td><td>vvi-miR159a|+10_perfect match probe</td></tr>
<tr><td>vvi|miR-382|2mut|as|</td><td>aaggcttatgagagttccct</td><td>vvi-miR159a|+10_mismatch probe</td></tr>
<tr><td>vvi|miR-383|nat|as|</td><td>tcactccaaggccagaaaag</td><td>vvi-miR159b|-10_perfect match probe</td></tr>
<tr><td>vvi|miR-383|2mut|as|</td><td>tcactcaaaggtcagaaaag</td><td>vvi-miR159b|-10_mismatch probe</td></tr>
<tr><td>vvi|miR-384|nat|as|</td><td>agagctcccttcactccaag</td><td>vvi-miR159b|0_perfect match probe</td></tr>
<tr><td>vvi|miR-384|2mut|as|</td><td>agagctctcttcacttcaag</td><td>vvi-miR159b|0_mismatch probe</td></tr>
<tr><td>vvi|miR-385|nat|as|</td><td>atcggttatgagagctccct</td><td>vvi-miR159b|+10_perfect match probe</td></tr>
<tr><td>vvi|miR-385|2mut|as|</td><td>atcgcttatgagagttccct</td><td>vvi-miR159b|+10_mismatch probe</td></tr>
<tr><td>vvi|miR-386|nat|as|</td><td>tcaatccaaacaaggatcta</td><td>vvi-miR159c|-10_perfect match probe</td></tr>
<tr><td>vvi|miR-386|2mut|as|</td><td>tcaatcaaaacaagaatcta</td><td>vvi-miR159c|-10_mismatch probe</td></tr>
<tr><td>vvi|miR-387|nat|as|</td><td>agagctcccttcaatccaaa</td><td>vvi-miR159c|0_perfect match probe</td></tr>
<tr><td>vvi|miR-387|2mut|as|</td><td>agagctctcttcaattcaaa</td><td>vvi-miR159c|0_mismatch probe</td></tr>
<tr><td>vvi|miR-388|nat|as|</td><td>agagaggtgtagagctccct</td><td>vvi-miR159c|+10_perfect match probe</td></tr>
</table>

vvi|miR-388|2mut|as|	agagagctgtagagttccct
vvi|miR-389|nat|as|	gagccaggcagatcaacccc
vvi|miR-389|2mut|as|	gagccagccagataaacccc
vvi|miR-390|nat|as|	ggcattcagggagccaggca
vvi|miR-390|2mut|as|	ggcattcagtgagtcaggca
vvi|miR-391|nat|as|	cttcttagatggcattcagg
vvi|miR-391|2mut|as|	cttcttacatgccattcagg
vvi|miR-392|nat|as|	gagccaggcagaacaatatt
vvi|miR-392|2mut|as|	gagccagccagaaaaatatt
vvi|miR-393|nat|as|	ggcattcagggagccaggca
vvi|miR-393|2mut|as|	ggcattcagtgagtcaggca
vvi|miR-394|nat|as|	cttcttagatggcattcagg
vvi|miR-394|2mut|as|	cttcttacatgccattcagg
vvi|miR-395|nat|as|	gagccaggcacaacccattt
vvi|miR-395|2mut|as|	gagccagccacaactcattt
vvi|miR-396|nat|as|	ggcatacagggagccaggca
vvi|miR-396|2mut|as|	ggcatacagtgagtcaggca
vvi|miR-397|nat|as|	ctctacgtgtggcatacagg
vvi|miR-397|2mut|as|	ctctacctgtgccatacagg
vvi|miR-398|nat|as|	gagccaggcatgtaagtatg
vvi|miR-398|2mut|as|	gagccagccatctaagtatg
vvi|miR-399|nat|as|	ggcatacagggagccaggca
vvi|miR-399|2mut|as|	ggcatacagtgagtcaggca
vvi|miR-400|nat|as|	ctttgcaaatggcatacagg
vvi|miR-400|2mut|as|	ctttgaaaatgccatacagg
vvi|miR-401|nat|as|	gagccaggcagaacaatatt

vvi-miR159c|+10_mismatch probe
vvi-miR160a|-10_perfect match probe
vvi-miR160a|-10_mismatch probe
vvi-miR160a|0_perfect match probe
vvi-miR160a|0_mismatch probe
vvi-miR160a|+10_perfect match probe
vvi-miR160a|+10_mismatch probe
vvi-miR160b|-10_perfect match probe
vvi-miR160b|-10_mismatch probe
vvi-miR160b|0_perfect match probe
vvi-miR160b|0_mismatch probe
vvi-miR160b|+10_perfect match probe
vvi-miR160b|+10_mismatch probe
vvi-miR160c|-10_perfect match probe
vvi-miR160c|-10_mismatch probe
vvi-miR160c|0_perfect match probe
vvi-miR160c|0_mismatch probe
vvi-miR160c|+10_perfect match probe
vvi-miR160c|+10_mismatch probe
vvi-miR160d|-10_perfect match probe
vvi-miR160d|-10_mismatch probe
vvi-miR160d|0_perfect match probe
vvi-miR160d|0_mismatch probe
vvi-miR160d|+10_perfect match probe
vvi-miR160d|+10_mismatch probe
vvi-miR160e|-10_perfect match

		probe
vvi|miR-401|2mut|as|	gagccagccagaaaaatatt	vvi-miR160e|-10_mismatch probe
vvi|miR-402|nat|as|	ggcattcagggagccaggca	vvi-miR160e|0_perfect match probe
vvi|miR-402|2mut|as|	ggcattcagtgagtcaggca	vvi-miR160e|0_mismatch probe
vvi|miR-403|nat|as|	cttcttagatggcattcagg	vvi-miR160e|+10_perfect match probe
vvi|miR-403|2mut|as|	cttcttacatgccattcagg	vvi-miR160e|+10_mismatch probe
vvi|miR-404|nat|as|	gagccaggcatatatgctta	vvi-miR160f|-10_perfect match probe
vvi|miR-404|2mut|as|	gagccagccatatatcctta	vvi-miR160f|-10_mismatch probe
vvi|miR-405|nat|as|	ggcatacagggagccaggca	vvi-miR160f|0_perfect match probe
vvi|miR-405|2mut|as|	ggcatacagtgagtcaggca	vvi-miR160f|0_mismatch probe
vvi|miR-406|nat|as|	ctctgcaaatggcatacagg	vvi-miR160f|+10_perfect match probe
vvi|miR-406|2mut|as|	ctctgaaaatgccatacagg	vvi-miR160f|+10_mismatch probe
vvi|miR-407|nat|as|	ggtttatcgaccgattcttg	vvi-miR162|-10_perfect match probe
vvi|miR-407|2mut|as|	ggtttatagacagattcttg	vvi-miR162|-10_mismatch probe
vvi|miR-408|nat|as|	tggatgcagaggtttatcga	vvi-miR162|0_perfect match probe
vvi|miR-408|2mut|as|	tggatgaagagctttatcga	vvi-miR162|0_mismatch probe
vvi|miR-409|nat|as|	gagtgaacgctggatgcaga	vvi-miR162|+10_perfect match probe
vvi|miR-409|2mut|as|	gagtgaaccctggatccaga	vvi-miR162|+10_mismatch probe
vvi|miR-410|nat|as|	tgcttctccaacaaggagct	vvi-miR164a|-10_perfect match probe
vvi|miR-410|2mut|as|	tgcttctcaaacaagaagct	vvi-miR164a|-10_mismatch probe
vvi|miR-411|nat|as|	gcacgtgccctgcttctcca	vvi-miR164a|0_perfect match probe
vvi|miR-411|2mut|as|	gcacgtgtcctccttctcca	vvi-miR164a|0_mismatch probe
vvi|miR-412|nat|as|	aggcaaatctgcacgtgccc	vvi-miR164a|+10_perfect match probe
vvi|miR-412|2mut|as|	aggcaaatccgaacgtgccc	vvi-miR164a|+10_mismatch probe
vvi|miR-413|nat|as|	tgcttctccagcatggttta	vvi-miR164b|-10_perfect match probe
vvi|miR-413|2mut|as|	tgcttctcaagaatggttta	vvi-miR164b|-10_mismatch probe
vvi|miR-414|nat|as|	gcatgtgccctgcttctcca	vvi-miR164b|0_perfect match probe
vvi|miR-414|2mut|as|	gcatgtgtcctccttctcca	vvi-miR164b|0_mismatch probe
vvi|miR-415|nat|as|	gattgatccagcatgtgccc	vvi-miR164b|+10_perfect match

		probe
vvi|miR-415|2mut|as|	gattgatcaagaatgtgccc	vvi-miR164b|+10_mismatch probe
vvi|miR-416|nat|as|	tgcttctccatcaagagctt	vvi-miR164d|-10_perfect match probe
vvi|miR-416|2mut|as|	tgcttctcaataaagagctt	vvi-miR164d|-10_mismatch probe
vvi|miR-417|nat|as|	gcacgtgccctgcttctcca	vvi-miR164d|0_perfect match probe
vvi|miR-417|2mut|as|	gcacgtgtcctccttctcca	vvi-miR164d|0_mismatch probe
vvi|miR-418|nat|as|	tttgtgaactgcacgtgccc	vvi-miR164d|+10_perfect match probe
vvi|miR-418|2mut|as|	tttgcgaactgaacgtgccc	vvi-miR164d|+10_mismatch probe
vvi|miR-419|nat|as|	cctggtccgacgacaccaac	vvi-miR166c|-10_perfect match probe
vvi|miR-419|2mut|as|	cctggtcagaagacaccaac	vvi-miR166c|-10_mismatch probe
vvi|miR-420|nat|as|	gggaatgaagcctggtccga	vvi-miR166c|0_perfect match probe
vvi|miR-420|2mut|as|	gggaatgaactctggtccga	vvi-miR166c|0_mismatch probe
vvi|miR-421|nat|as|	gataattggggggaatgaag	vvi-miR166c|+10_perfect match probe
vvi|miR-421|2mut|as|	gataattggtgtgaatgaag	vvi-miR166c|+10_mismatch probe
vvi|miR-422|nat|as|	cctggtccgagggcactaac	vvi-miR166d|-10_perfect match probe
vvi|miR-422|2mut|as|	cctggtcagaggccactaac	vvi-miR166d|-10_mismatch probe
vvi|miR-423|nat|as|	gggaatgaagcctggtccga	vvi-miR166d|0_perfect match probe
vvi|miR-423|2mut|as|	gggaatgaactctggtccga	vvi-miR166d|0_mismatch probe
vvi|miR-424|nat|as|	agtgattgaggggaatgaag	vvi-miR166d|+10_perfect match probe
vvi|miR-424|2mut|as|	agtgattgacgtgaatgaag	vvi-miR166d|+10_mismatch probe
vvi|miR-425|nat|as|	cctggtccgacgacaccaac	vvi-miR166e|-10_perfect match probe
vvi|miR-425|2mut|as|	cctggtcagaagacaccaac	vvi-miR166e|-10_mismatch probe
vvi|miR-426|nat|as|	gggaatgaagcctggtccga	vvi-miR166e|0_perfect match probe
vvi|miR-426|2mut|as|	gggaatgaactctggtccga	vvi-miR166e|0_mismatch probe
vvi|miR-427|nat|as|	ataaattggggggaatgaag	vvi-miR166e|+10_perfect match probe
vvi|miR-427|2mut|as|	ataaattggtgtgaatgaag	vvi-miR166e|+10_mismatch probe

vvi|miR-428|nat|as|	cctggtccgaagctttttga
vvi|miR-428|2mut|as|	cctggtcagaagtttttga
vvi|miR-429|nat|as|	gggaatgaagcctggtccga
vvi|miR-429|2mut|as|	gggaatgaactctggtccga
vvi|miR-430|nat|as|	gtgtgttgaggggaatgaag
vvi|miR-430|2mut|as|	gtgtgttgacgtgaatgaag
vvi|miR-431|nat|as|	cctggtccgaaatcattcaa
vvi|miR-431|2mut|as|	cctggtcagaaataattcaa
vvi|miR-432|nat|as|	gggaatgaagcctggtccga
vvi|miR-432|2mut|as|	gggaatgaactctggtccga
vvi|miR-433|nat|as|	ttgggttgaggggaatgaag
vvi|miR-433|2mut|as|	ttggcttgaggtgaatgaag
vvi|miR-434|nat|as|	cctggtccgagatcattcag
vvi|miR-434|2mut|as|	cctggtcagagataattcag
vvi|miR-435|nat|as|	gggaatgaagcctggtccga
vvi|miR-435|2mut|as|	gggaatgaactctggtccga
vvi|miR-436|nat|as|	ttgagttgggggaatgaag
vvi|miR-436|2mut|as|	ttgagttggtgtgaatgaag
vvi|miR-437|nat|as|	cctggtccgagatcaaccga
vvi|miR-437|2mut|as|	cctggtcagagataaaccga
vvi|miR-438|nat|as|	aggaatgaagcctggtccga
vvi|miR-438|2mut|as|	aggaatgaactctggtccga
vvi|miR-439|nat|as|	gcttggtgtcaggaatgaag
vvi|miR-439|2mut|as|	gcttcgtgtcagaaatgaag
vvi|miR-440|nat|as|	cctggtccgacaccttcact
vvi|miR-440|2mut|as|	cctggtcagacatcttcact

vvi-miR166f|-10_perfect match probe
vvi-miR166f|-10_mismatch probe
vvi-miR166f|0_perfect match probe
vvi-miR166f|0_mismatch probe
vvi-miR166f|+10_perfect match probe
vvi-miR166f|+10_mismatch probe
vvi-miR166g|-10_perfect match probe
vvi-miR166g|-10_mismatch probe
vvi-miR166g|0_perfect match probe
vvi-miR166g|0_mismatch probe
vvi-miR166g|+10_perfect match probe
vvi-miR166g|+10_mismatch probe
vvi-miR166h|-10_perfect match probe
vvi-miR166h|-10_mismatch probe
vvi-miR166h|0_perfect match probe
vvi-miR166h|0_mismatch probe
vvi-miR166h|+10_perfect match probe
vvi-miR166h|+10_mismatch probe
vvi-miR166a|-10_perfect match probe
vvi-miR166a|-10_mismatch probe
vvi-miR166a|0_perfect match probe
vvi-miR166a|0_mismatch probe
vvi-miR166a|+10_perfect match probe
vvi-miR166a|+10_mismatch probe
vvi-miR166b|-10_perfect match probe
vvi-miR166b|-10_mismatch probe

vvi|miR-441|nat|as|	aggaatgaagcctggtccga
vvi|miR-441|2mut|as|	aggaatgaactctggtccga
vvi|miR-442|nat|as|	tatatttgagaggaatgaag
vvi|miR-442|2mut|as|	tatatttaagagaaatgaag
vvi|miR-443|nat|as|	ggcagcttcaacctctcaaa
vvi|miR-443|2mut|as|	ggcagtttcaatctctcaaa
vvi|miR-444|nat|as|	agatcatgctggcagcttca
vvi|miR-444|2mut|as|	agatcatcctgccagcttca
vvi|miR-445|nat|as|	ttgtttcaccagatcatgct
vvi|miR-445|2mut|as|	ttgtttcacacgatcatgct
vvi|miR-446|nat|as|	ggcagcttcaactgctattg
vvi|miR-446|2mut|as|	ggcagtttcaactcctattg
vvi|miR-447|nat|as|	agatcatgctggcagcttca
vvi|miR-447|2mut|as|	agatcatcctgccagcttca
vvi|miR-448|nat|as|	agaaaagcttagatcatgct
vvi|miR-448|2mut|as|	agaaaagtttagataatgct
vvi|miR-449|nat|as|	ggcagcttcaactgctactg
vvi|miR-449|2mut|as|	ggcagtttcaactcctactg
vvi|miR-450|nat|as|	agggaagttgagatcatgct
vvi|miR-450|2mut|as|	agggaagttaagataatgct
vvi|miR-451|nat|as|	ggcagcttcacttattccct
vvi|miR-451|2mut|as|	ggcagtttcatttattccct
vvi|miR-452|nat|as|	agatcatgctggcagcttca
vvi|miR-452|2mut|as|	agatcatcctgccagcttca
vvi|miR-453|nat|as|	agccaaagctagatcatgct

vvi-miR166b|0_perfect match probe
vvi-miR166b|0_mismatch probe
vvi-miR166b|+10_perfect match probe
vvi-miR166b|+10_mismatch probe
vvi-miR167a|-10_perfect match probe
vvi-miR167a|-10_mismatch probe
vvi-miR167a|0_perfect match probe
vvi-miR167a|0_mismatch probe
vvi-miR167a|+10_perfect match probe
vvi-miR167a|+10_mismatch probe
vvi-miR167b|-10_perfect match probe
vvi-miR167b|-10_mismatch probe
vvi-miR167b|0_perfect match probe
vvi-miR167b|0_mismatch probe
vvi-miR167b|+10_perfect match probe
vvi-miR167b|+10_mismatch probe
vvi-miR167c|-10_perfect match probe
vvi-miR167c|-10_mismatch probe
vvi-miR167c|+10_perfect match probe
vvi-miR167c|+10_mismatch probe
vvi-miR167d|-10_perfect match probe
vvi-miR167d|-10_mismatch probe
vvi-miR167d|0_perfect match probe
vvi-miR167d|0_mismatch probe
vvi-miR167d|+10_perfect match probe

vvi|miR-453|2mut|as|	agccaaagttagataatgct
vvi|miR-454|nat|as|	ggcagcttcaactgatagtg
vvi|miR-454|2mut|as|	ggcagtttcaaccgatagtg
vvi|miR-455|nat|as|	agatcatgctggcagcttca
vvi|miR-455|2mut|as|	agatcatcctgccagcttca
vvi|miR-456|nat|as|	aagcaagtttagatcatgct
vvi|miR-456|2mut|as|	aagcaactttagataatgct
vvi|miR-457|nat|as|	caccaagcgaattagagacc
vvi|miR-457|2mut|as|	caccaagagaattagcgacc
vvi|miR-458|nat|as|	tcccgacctgcaccaagcga
vvi|miR-458|2mut|as|	tcccgacctcaaccaagcga
vvi|miR-459|nat|as|	cgaagtcggttcccgacctg
vvi|miR-459|2mut|as|	cgaagtcagttccagacctg
vvi|miR-460|nat|as|	tccttggctgcattccactc
vvi|miR-460|2mut|as|	tccttgcctgaattccactc
vvi|miR-461|nat|as|	cggcaagtcatccttggctg
vvi|miR-461|2mut|as|	cggcaagccatccttcgctg
vvi|miR-462|nat|as|	atataaattccggcaagtca
vvi|miR-462|2mut|as|	atataaatttcgccaagtca
vvi|miR-463|nat|as|	tccttcgctaccaaacaacg
vvi|miR-463|2mut|as|	tccttccctacaaaacaacg
vvi|miR-464|nat|as|	aggcaagtcatccttcgcta
vvi|miR-464|2mut|as|	aggcaagccatccttagcta
vvi|miR-465|nat|as|	aggaggctgtaggcaagtca
vvi|miR-465|2mut|as|	aggagcctgtagccaagtca
vvi|miR-466|nat|as|	ccttggctcaattcgacccc
vvi|miR-466|2mut|as|	ccttgcctcaattagacccc

vvi-miR167d|+10_mismatch probe
vvi-miR167e|-10_perfect match probe
vvi-miR167e|-10_mismatch probe
vvi-miR167e|0_perfect match probe
vvi-miR167e|0_mismatch probe
vvi-miR167e|+10_perfect match probe
vvi-miR167e|+10_mismatch probe
vvi-miR168|-10_perfect match probe
vvi-miR168|-10_mismatch probe
vvi-miR168|0_perfect match probe
vvi-miR168|0_mismatch probe
vvi-miR168|+10_perfect match probe
vvi-miR168|+10_mismatch probe
vvi-miR169a|-10_perfect match probe
vvi-miR169a|-10_mismatch probe
vvi-miR169a|0_perfect match probe
vvi-miR169a|0_mismatch probe
vvi-miR169a|+10_perfect match probe
vvi-miR169a|+10_mismatch probe
vvi-miR169y|-10_perfect match probe
vvi-miR169y|-10_mismatch probe
vvi-miR169y|0_perfect match probe
vvi-miR169y|0_mismatch probe
vvi-miR169y|+10_perfect match probe
vvi-miR169y|+10_mismatch probe
vvi-miR169b|-10_perfect match probe
vvi-miR169b|-10_mismatch probe

vvi|miR-467|nat|as|	ggcaagccatccttggctca
vvi|miR-467|2mut|as|	ggcaagtcatccttgcctca
vvi|miR-468|nat|as|	tgcagatgacggcaagccat
vvi|miR-468|2mut|as|	tgcagatgaagccaagccat
vvi|miR-469|nat|as|	tccttggctgcattctactc
vvi|miR-469|2mut|as|	tccttgcctgaattctactc
vvi|miR-470|nat|as|	cggcaagtcatccttggctg
vvi|miR-470|2mut|as|	cggcaagccatccttcgctg
vvi|miR-471|nat|as|	gccccatctccggcaagtca
vvi|miR-471|2mut|as|	gcccaatctccgccaagtca
vvi|miR-472|nat|as|	ttcttggctgcattccactc
vvi|miR-472|2mut|as|	ttcttgcctgaattccactc
vvi|miR-473|nat|as|	cggcaaatcattcttggctg
vvi|miR-473|2mut|as|	cggcaaataattcttcgctg
vvi|miR-474|nat|as|	atataaattccggcaaatca
vvi|miR-474|2mut|as|	atataaatttcgccaaatca
vvi|miR-475|nat|as|	tccttggctatcagacaaga
vvi|miR-475|2mut|as|	tccttgcctatcagaaaaga
vvi|miR-476|nat|as|	aggcaagtcatccttggcta
vvi|miR-476|2mut|as|	aggcaagccatccttcgcta
vvi|miR-477|nat|as|	gggatagggcaggcaagtca
vvi|miR-477|2mut|as|	gggataggccagccaagtca
vvi|miR-478|nat|as|	tccttggctgcaccataata
vvi|miR-478|2mut|as|	tccttgcctgaaccataata
vvi|miR-479|nat|as|	cggcaagtcatccttggctg
vvi|miR-479|2mut|as|	cggcaagccatccttcgctg
vvi|miR-480|nat|as|	aaattattttcggcaagtca

vvi-miR169b|0_perfect match probe
vvi-miR169b|0_mismatch probe
vvi-miR169b|+10_perfect match probe
vvi-miR169b|+10_mismatch probe
vvi-miR169c|-10_perfect match probe
vvi-miR169c|-10_mismatch probe
vvi-miR169c|0_perfect match probe
vvi-miR169c|0_mismatch probe
vvi-miR169c|+10_perfect match probe
vvi-miR169c|+10_mismatch probe
vvi-miR169d|-10_perfect match probe
vvi-miR169d|-10_mismatch probe
vvi-miR169d|0_perfect match probe
vvi-miR169d|0_mismatch probe
vvi-miR169d|+10_perfect match probe
vvi-miR169d|+10_mismatch probe
vvi-miR169e|-10_perfect match probe
vvi-miR169e|-10_mismatch probe
vvi-miR169e|0_perfect match probe
vvi-miR169e|0_mismatch probe
vvi-miR169e|+10_perfect match probe
vvi-miR169e|+10_mismatch probe
vvi-miR169f|-10_perfect match probe
vvi-miR169f|-10_mismatch probe
vvi-miR169f|0_perfect match probe
vvi-miR169f|0_mismatch probe
vvi-miR169f|+10_perfect match

		probe
vvi|miR-480|2mut|as|	aaattatttccgccaagtca	vvi-miR169f|+10_mismatch probe
		vvi-miR169g|-10_perfect match
vvi|miR-481|nat|as|	tccttggctgcaccatactt	probe
vvi|miR-481|2mut|as|	tccttgcctgaaccatactt	vvi-miR169g|-10_mismatch probe
vvi|miR-482|nat|as|	cggcaagtcatccttggctg	vvi-miR169g|0_perfect match probe
vvi|miR-482|2mut|as|	cggcaagccatccttcgctg	vvi-miR169g|0_mismatch probe
		vvi-miR169g|+10_perfect match
vvi|miR-483|nat|as|	agtagagagtcggcaagtca	probe
vvi|miR-483|2mut|as|	agtagagagccgccaagtca	vvi-miR169g|+10_mismatch probe
		vvi-miR169h|-10_perfect match
vvi|miR-484|nat|as|	ccttggctcaattccaccct	probe
vvi|miR-484|2mut|as|	ccttgcctcaattcaaccct	vvi-miR169h|-10_mismatch probe
vvi|miR-485|nat|as|	ggcaagccatccttggctca	vvi-miR169h|0_perfect match probe
vvi|miR-485|2mut|as|	ggcaagtcatccttgcctca	vvi-miR169h|0_mismatch probe
		vvi-miR169h|+10_perfect match
vvi|miR-486|nat|as|	gacaaaggacggcaagccat	probe
vvi|miR-486|2mut|as|	gacaaaggaagccaagccat	vvi-miR169h|+10_mismatch probe
vvi|miR-487|nat|as|	tccttggctcaattccaccc	vvi-miR169i|-10_perfect match probe
vvi|miR-487|2mut|as|	tccttgcctcaattcaaccc	vvi-miR169i|-10_mismatch probe
vvi|miR-488|nat|as|	cggccagtcatccttggctc	vvi-miR169i|0_perfect match probe
vvi|miR-488|2mut|as|	cggcaagtcatccttcgctc	vvi-miR169i|0_mismatch probe
		vvi-miR169i|+10_perfect match
vvi|miR-489|nat|as|	tgaaaaatgacggccagtca	probe
vvi|miR-489|2mut|as|	tgaaaaatgccggtcagtca	vvi-miR169i|+10_mismatch probe
vvi|miR-490|nat|as|	tccttggctgcactccactc	vvi-miR169j|-10_perfect match probe
vvi|miR-490|2mut|as|	tccttgcctgaactccactc	vvi-miR169j|-10_mismatch probe
vvi|miR-491|nat|as|	cggcaagtcatccttggctg	vvi-miR169j|0_perfect match probe
vvi|miR-491|2mut|as|	cggcaagccatccttcgctg	vvi-miR169j|0_mismatch probe
		vvi-miR169j|+10_perfect match
vvi|miR-492|nat|as|	atgtgaattccggcaagtca	probe
vvi|miR-492|2mut|as|	atgtgaatttcgccaagtca	vvi-miR169j|+10_mismatch probe
		vvi-miR169k|-10_perfect match
vvi|miR-493|nat|as|	tccttggctgcactccactc	probe

vvi|miR-493|2mut|as|	tccttgcctgaactccactc
vvi|miR-494|nat|as|	cggcaagtcatccttggctg
vvi|miR-494|2mut|as|	cggcaagccatccttcgctg
vvi|miR-495|nat|as|	atgtgaattccggcaagtca
vvi|miR-495|2mut|as|	atgtgaatttcgccaagtca
vvi|miR-496|nat|as|	tccttggctcaattccaccc
vvi|miR-496|2mut|as|	tccttgcctcaattcaaccc
vvi|miR-497|nat|as|	cggcaagtcatccttggctc
vvi|miR-497|2mut|as|	cggcaagccatccttcgctc
vvi|miR-498|nat|as|	atgcaaaggacggcaagtca
vvi|miR-498|2mut|as|	atgcaaagaacgccaagtca
vvi|miR-499|nat|as|	tccttggctcacttccactc
vvi|miR-499|2mut|as|	tccttgcctcacttcaactc
vvi|miR-500|nat|as|	cggcaagtcatccttggctc
vvi|miR-500|2mut|as|	cggcaagccatccttcgctc
vvi|miR-501|nat|as|	ctgcagctgccggcaagtca
vvi|miR-501|2mut|as|	ctgcagctgtcgccaagtca
vvi|miR-502|nat|as|	tccttggctctattccactc
vvi|miR-502|2mut|as|	tccttgcctctattcaactc
vvi|miR-503|nat|as|	cggcaagtcatccttggctc
vvi|miR-503|2mut|as|	cggcaagccatccttcgctc
vvi|miR-504|nat|as|	ctgcagttgccggcaagtca
vvi|miR-504|2mut|as|	ctgcagttgtcgccaagtca
vvi|miR-505|nat|as|	tccttggctcaaatccaccc
vvi|miR-505|2mut|as|	tccttgcctcaaatcaaccc
vvi|miR-506|nat|as|	cggcaagtcatccttggctc
vvi|miR-506|2mut|as|	cggcaagccatccttcgctc

vvi-miR169k|-10_mismatch probe
vvi-miR169k|0_perfect match probe
vvi-miR169k|0_mismatch probe
vvi-miR169k|+10_perfect match probe
vvi-miR169k|+10_mismatch probe
vvi-miR169l|-10_perfect match probe
vvi-miR169l|-10_mismatch probe
vvi-miR169l|0_perfect match probe
vvi-miR169l|0_mismatch probe
vvi-miR169l|+10_perfect match probe
vvi-miR169l|+10_mismatch probe
vvi-miR169m|-10_perfect match probe
vvi-miR169m|-10_mismatch probe
vvi-miR169m|0_perfect match probe
vvi-miR169m|0_mismatch probe
vvi-miR169m|+10_perfect match probe
vvi-miR169m|+10_mismatch probe
vvi-miR169n|-10_perfect match probe
vvi-miR169n|-10_mismatch probe
vvi-miR169n|0_perfect match probe
vvi-miR169n|0_mismatch probe
vvi-miR169n|+10_perfect match probe
vvi-miR169n|+10_mismatch probe
vvi-miR169o|-10_perfect match probe
vvi-miR169o|-10_mismatch probe
vvi-miR169o|0_perfect match probe
vvi-miR169o|0_mismatch probe

vvi|miR-507|nat|as| ctgctgatggcggcaagtca vvi-miR169o|+10_perfect match probe
vvi|miR-507|2mut|as| ctgctgatgcaggcaagtca vvi-miR169o|+10_mismatch probe
vvi|miR-508|nat|as| tccttggctcaattccacac vvi-miR169p|-10_perfect match probe
vvi|miR-508|2mut|as| tccttgcctcaattcaacac vvi-miR169p|-10_mismatch probe
vvi|miR-509|nat|as| cggcaagtcatccttggctc vvi-miR169p|0_perfect match probe
vvi|miR-509|2mut|as| cggcaagccatccttcgctc vvi-miR169p|0_mismatch probe
vvi|miR-510|nat|as| ctgcagctgccggcaagtca vvi-miR169p|+10_perfect match probe
vvi|miR-510|2mut|as| ctgcagctgtcgccaagtca vvi-miR169p|+10_mismatch probe
vvi|miR-511|nat|as| tccttggctctattccaccc vvi-miR169q|-10_perfect match probe
vvi|miR-511|2mut|as| tccttgcctctattcaaccc vvi-miR169q|-10_mismatch probe
vvi|miR-512|nat|as| cggcaagtcatccttggctc vvi-miR169q|0_perfect match probe
vvi|miR-512|2mut|as| cggcaagccatccttcgctc vvi-miR169q|0_mismatch probe
vvi|miR-513|nat|as| ctgcaaatgccggcaagtca vvi-miR169q|+10_perfect match probe
vvi|miR-513|2mut|as| ctgcaaatgtcgccaagtca vvi-miR169q|+10_mismatch probe
vvi|miR-514|nat|as| ccttgactcaattccaccct vvi-miR169r|-10_perfect match probe
vvi|miR-514|2mut|as| ccttgcctcaattcaaccct vvi-miR169r|-10_mismatch probe
vvi|miR-515|nat|as| ggcaagtcatccttgactca vvi-miR169r|0_perfect match probe
vvi|miR-515|2mut|as| ggcaagccattcttgactca vvi-miR169r|0_mismatch probe
vvi|miR-516|nat|as| aatatatatcggcaagtcat vvi-miR169r|+10_perfect match probe
vvi|miR-516|2mut|as| aatatatatagccaagtcat vvi-miR169r|+10_mismatch probe
vvi|miR-517|nat|as| tccttggctgcaatccactt vvi-miR169s|-10_perfect match probe
vvi|miR-517|2mut|as| tccttgcctgaaatccactt vvi-miR169s|-10_mismatch probe
vvi|miR-518|nat|as| cggcaagtcatccttggctg vvi-miR169s|0_perfect match probe
vvi|miR-518|2mut|as| cggcaagccatccttcgctg vvi-miR169s|0_mismatch probe
vvi|miR-519|nat|as| tgccaagtgccggcaagtca vvi-miR169s|+10_perfect match probe
vvi|miR-519|2mut|as| tgccaagtgtcgccaagtca vvi-miR169s|+10_mismatch probe
vvi|miR-520|nat|as| ccttgactcgattccaccct vvi-miR169t|-10_perfect match probe

vvi|miR-520|2mut|as|	ccttgactagattcaaccct	vvi-miR169t|-10_mismatch probe
vvi|miR-521|nat|as|	ggcaagtcatccttgactcg	vvi-miR169t|0_perfect match probe
vvi|miR-521|2mut|as|	ggcaagccattcttgactcg	vvi-miR169t|0_mismatch probe
		vvi-miR169t|+10_perfect match probe
vvi|miR-522|nat|as|	aatatatatcggcaagtcat	
vvi|miR-522|2mut|as|	aatatatatagccaagtcat	vvi-miR169t|+10_mismatch probe
		vvi-miR169u|-10_perfect match probe
vvi|miR-523|nat|as|	ccttgactcaattccaccct	
vvi|miR-523|2mut|as|	ccttgcctcaattcaaccct	vvi-miR169u|-10_mismatch probe
vvi|miR-524|nat|as|	ggcaagtcatccttgactca	vvi-miR169u|0_perfect match probe
vvi|miR-524|2mut|as|	ggcaagccattcttgactca	vvi-miR169u|0_mismatch probe
		vvi-miR169u|+10_perfect match probe
vvi|miR-525|nat|as|	aatatataacggcaagtcat	
vvi|miR-525|2mut|as|	aatatataaagccaagtcat	vvi-miR169u|+10_mismatch probe
		vvi-miR169v|-10_perfect match probe
vvi|miR-526|nat|as|	tccttggctttgttcctccc	
vvi|miR-526|2mut|as|	tccttgcctttgtttctccc	vvi-miR169v|-10_mismatch probe
vvi|miR-527|nat|as|	cggcaattcatccttggctt	vvi-miR169v|0_perfect match probe
vvi|miR-527|2mut|as|	cggcaattaatccttcgctt	vvi-miR169v|0_mismatch probe
		vvi-miR169v|+10_perfect match probe
vvi|miR-528|nat|as|	ctttcatcgccggcaattca	
vvi|miR-528|2mut|as|	ctttcatccccgccaattca	vvi-miR169v|+10_mismatch probe
		vvi-miR169w|-10_perfect match probe
vvi|miR-529|nat|as|	tccttggctgcaccacataa	
vvi|miR-529|2mut|as|	tccttgcctgaaccacataa	vvi-miR169w|-10_mismatch probe
vvi|miR-530|nat|as|	cggcaagtcatccttggctg	vvi-miR169w|0_perfect match probe
vvi|miR-530|2mut|as|	cggcaagccatccttcgctg	vvi-miR169w|0_mismatch probe
		vvi-miR169w|+10_perfect match probe
vvi|miR-531|nat|as|	agggagttgccggcaagtca	
vvi|miR-531|2mut|as|	agggagttgtcgccaagtca	vvi-miR169w|+10_mismatch probe
		vvi-miR169x|-10_perfect match probe
vvi|miR-532|nat|as|	tccttggctaccagacgaga	
vvi|miR-532|2mut|as|	tccttgcctaccagaagaga	vvi-miR169x|-10_mismatch probe
vvi|miR-533|nat|as|	aggcaagtcatccttggcta	vvi-miR169x|0_perfect match probe

vvi|miR-533|2mut|as|	aggcaagccatccttcgcta
vvi|miR-534|nat|as|	tggtggatttaggcaagtca
vvi|miR-534|2mut|as|	tggtcgatttagccaagtca
vvi|miR-535|nat|as|	gacggctcaatcaaaccaaa
vvi|miR-535|2mut|as|	gacgcctcaatcaaatcaaa
vvi|miR-536|nat|as|	gtgatgttgggacggctcaa
vvi|miR-536|2mut|as|	gtgatgttgtgacgcctcaa
vvi|miR-537|nat|as|	ggtgggaagcgtgatgttgg
vvi|miR-537|2mut|as|	ggtgggaagactgatgttgg
vvi|miR-538|nat|as|	ggctcaatcaaatcacatgg
vvi|miR-538|2mut|as|	ggctcaataaaatcccatgg
vvi|miR-539|nat|as|	atattgacgcggctcaatca
vvi|miR-539|2mut|as|	atattgacgagcctcaatca
vvi|miR-540|nat|as|	gcaaaaggagatattgacgc
vvi|miR-540|2mut|as|	gcaaaagaagatattaacgc
vvi|miR-541|nat|as|	cacggctcaatcaaaaacca
vvi|miR-541|2mut|as|	cacgcctcaataaaaaacca
vvi|miR-542|nat|as|	gtgatattggcacggctcaa
vvi|miR-542|2mut|as|	gtgatattgccacgcctcaa
vvi|miR-543|nat|as|	gcaatgacacgtgatattgg
vvi|miR-543|2mut|as|	gcaatgacaactgatattgg
vvi|miR-544|nat|as|	cacggctcaatcaaacaaca
vvi|miR-544|2mut|as|	cacgcctcaatcaaaaaaca
vvi|miR-545|nat|as|	gtgatattggcacggctcaa
vvi|miR-545|2mut|as|	gtgatattgccacgcctcaa
vvi|miR-546|nat|as|	gaagtaggacgtgatattgg

vvi-miR169x|0_mismatch probe
vvi-miR169x|+10_perfect match probe
vvi-miR169x|+10_mismatch probe
vvi-miR171a|-10_perfect match probe
vvi-miR171a|-10_mismatch probe
vvi-miR171a|0_perfect match probe
vvi-miR171a|0_mismatch probe
vvi-miR171a|+10_perfect match probe
vvi-miR171a|+10_mismatch probe
vvi-miR171b|-10_perfect match probe
vvi-miR171b|-10_mismatch probe
vvi-miR171b|0_perfect match probe
vvi-miR171b|0_mismatch probe
vvi-miR171b|+10_perfect match probe
vvi-miR171b|+10_mismatch probe
vvi-miR171c|-10_perfect match probe
vvi-miR171c|-10_mismatch probe
vvi-miR171c|0_perfect match probe
vvi-miR171c|0_mismatch probe
vvi-miR171c|+10_perfect match probe
vvi-miR171c|+10_mismatch probe
vvi-miR171d|-10_perfect match probe
vvi-miR171d|-10_mismatch probe
vvi-miR171d|0_perfect match probe
vvi-miR171d|0_mismatch probe
vvi-miR171d|+10_perfect match

		probe
vvi|miR-546|2mut|as|	gaagtaggaactgatattgg	vvi-miR171d|+10_mismatch probe
vvi|miR-547|nat|as|	ggctcaatcagagatcgttc	vvi-miR171e|-10_perfect match probe
vvi|miR-547|2mut|as|	ggctcaatccgagatagttc	vvi-miR171e|-10_mismatch probe
vvi|miR-548|nat|as|	atattggcgcggctcaatca	vvi-miR171e|0_perfect match probe
vvi|miR-548|2mut|as|	atattggcgagcctcaatca	vvi-miR171e|0_mismatch probe
vvi|miR-549|nat|as|	gcagaaagtgatattggcgc	vvi-miR171e|+10_perfect match probe
vvi|miR-549|2mut|as|	gcagaaagcgatattcgcgc	vvi-miR171e|+10_mismatch probe
vvi|miR-550|nat|as|	cgcggctcaatctgagatca	vvi-miR171f|-10_perfect match probe
vvi|miR-550|2mut|as|	cgcgcctcaatccgagatca	vvi-miR171f|-10_mismatch probe
vvi|miR-551|nat|as|	gtgatattggcgcggctcaa	vvi-miR171f|0_perfect match probe
vvi|miR-551|2mut|as|	gtgatattgccgaggctcaa	vvi-miR171f|0_mismatch probe
vvi|miR-552|nat|as|	ccacagaaaagtgatattgg	vvi-miR171f|+10_perfect match probe
vvi|miR-552|2mut|as|	ccaccgaaaagcgatattgg	vvi-miR171f|+10_mismatch probe
vvi|miR-553|nat|as|	ttcggctcaaggagttggtg	vvi-miR171g|-10_perfect match probe
vvi|miR-553|2mut|as|	ttcgcctcaagaagttggtg	vvi-miR171g|-10_mismatch probe
vvi|miR-554|nat|as|	gtgatattggttcggctcaa	vvi-miR171g|0_perfect match probe
vvi|miR-554|2mut|as|	gtgatattcgttcgcctcaa	vvi-miR171g|0_mismatch probe
vvi|miR-555|nat|as|	tttggctcgggtgatattgg	vvi-miR171g|+10_perfect match probe
vvi|miR-555|2mut|as|	tttgcctcggctgatattgg	vvi-miR171g|+10_mismatch probe
vvi|miR-556|nat|as|	ggctcaaccaaacatcagag	vvi-miR171h|-10_perfect match probe
vvi|miR-556|2mut|as|	ggctcaacaaaaaatcagag	vvi-miR171h|-10_mismatch probe
vvi|miR-557|nat|as|	atattggcgcggctcaacca	vvi-miR171h|0_perfect match probe
vvi|miR-557|2mut|as|	atattggcgagcctcaacca	vvi-miR171h|0_mismatch probe
vvi|miR-558|nat|as|	gatagacgggatattggcgc	vvi-miR171h|+10_perfect match probe
vvi|miR-558|2mut|as|	gatagacaggatattcgcgc	vvi-miR171h|+10_mismatch probe
vvi|miR-559|nat|as|	cacggctcaatcagattaaa	vvi-miR171i|-10_perfect match probe

<table>
<tr><td>vvi|miR-559|2mut|as|</td><td>cacgcctcaatccgattaaa</td><td>vvi-miR171i|-10_mismatch probe</td></tr>
<tr><td>vvi|miR-560|nat|as|</td><td>atgatattggcacggctcaa</td><td>vvi-miR171i|0_perfect match probe</td></tr>
<tr><td>vvi|miR-560|2mut|as|</td><td>atgatattgccacgcctcaa</td><td>vvi-miR171i|0_mismatch probe</td></tr>
<tr><td>vvi|miR-561|nat|as|</td><td>agtgaacatgatgatattgg</td><td>vvi-miR171i|+10_perfect match probe</td></tr>
<tr><td>vvi|miR-561|2mut|as|</td><td>agtgaaaatgataatattgg</td><td>vvi-miR171i|+10_mismatch probe</td></tr>
<tr><td>vvi|miR-562|nat|as|</td><td>tcaagattcacatgcaaatg</td><td>vvi-miR172a|-10_perfect match probe</td></tr>
<tr><td>vvi|miR-562|2mut|as|</td><td>tcaagattcccatgaaaatg</td><td>vvi-miR172a|-10_mismatch probe</td></tr>
<tr><td>vvi|miR-563|nat|as|</td><td>tgtagcatcatcaagattca</td><td>vvi-miR172a|0_perfect match probe</td></tr>
<tr><td>vvi|miR-563|2mut|as|</td><td>tgtagaatcataaagattca</td><td>vvi-miR172a|0_mismatch probe</td></tr>
<tr><td>vvi|miR-564|nat|as|</td><td>tgtttgcgcatgtagcatca</td><td>vvi-miR172a|+10_perfect match probe</td></tr>
<tr><td>vvi|miR-564|2mut|as|</td><td>tgtttgcccatgtagaatca</td><td>vvi-miR172a|+10_mismatch probe</td></tr>
<tr><td>vvi|miR-565|nat|as|</td><td>tcaagattcacatgcgaaag</td><td>vvi-miR172b|-10_perfect match probe</td></tr>
<tr><td>vvi|miR-565|2mut|as|</td><td>tcaagattcccatgagaaag</td><td>vvi-miR172b|-10_mismatch probe</td></tr>
<tr><td>vvi|miR-566|nat|as|</td><td>tgtagcatcatcaagattca</td><td>vvi-miR172b|0_perfect match probe</td></tr>
<tr><td>vvi|miR-566|2mut|as|</td><td>tgtagaatcataaagattca</td><td>vvi-miR172b|0_mismatch probe</td></tr>
<tr><td>vvi|miR-567|nat|as|</td><td>tgtttgcaggtgtagcatca</td><td>vvi-miR172b|+10_perfect match probe</td></tr>
<tr><td>vvi|miR-567|2mut|as|</td><td>tgtttgaaggtgtagaatca</td><td>vvi-miR172b|+10_mismatch probe</td></tr>
<tr><td>vvi|miR-568|nat|as|</td><td>tcaagattcccacatcaaag</td><td>vvi-miR172c|-10_perfect match probe</td></tr>
<tr><td>vvi|miR-568|2mut|as|</td><td>tcaagattctaacatcaaag</td><td>vvi-miR172c|-10_mismatch probe</td></tr>
<tr><td>vvi|miR-569|nat|as|</td><td>tgcagcatcatcaagattcc</td><td>vvi-miR172c|0_perfect match probe</td></tr>
<tr><td>vvi|miR-569|2mut|as|</td><td>tgcagaatcataaagattcc</td><td>vvi-miR172c|0_mismatch probe</td></tr>
<tr><td>vvi|miR-570|nat|as|</td><td>ttattgccgctgcagcatca</td><td>vvi-miR172c|+10_perfect match probe</td></tr>
<tr><td>vvi|miR-570|2mut|as|</td><td>ttattgcccctgcagaatca</td><td>vvi-miR172c|+10_mismatch probe</td></tr>
<tr><td>vvi|miR-571|nat|as|</td><td>tcaagattctcatctcaaaa</td><td>vvi-miR172d|-10_perfect match probe</td></tr>
<tr><td>vvi|miR-571|2mut|as|</td><td>tcaagattcccatctaaaaa</td><td>vvi-miR172d|-10_mismatch probe</td></tr>
<tr><td>vvi|miR-572|nat|as|</td><td>tgcagcatcatcaagattct</td><td>vvi-miR172d|0_perfect match probe</td></tr>
</table>

vvi|miR-572|2mut|as| tgcagaatcataaagattct

vvi|miR-573|nat|as| tcactgcgaatgcagcatca
vvi|miR-573|2mut|as| tcactgagaatgcagaatca

vvi|miR-574|nat|as| tcagtccaagacgtgacagc
vvi|miR-574|2mut|as| tcagtcaaagacctgacagc
vvi|miR-575|nat|as| ggagctcccttcagtccaag
vvi|miR-575|2mut|as| ggagctctcttcagttcaag

vvi|miR-576|nat|as| ggaatacaagggagctccct
vvi|miR-576|2mut|as| ggaatacaactgagctccct

vvi|miR-577|nat|as| tcagtccaagcacagggagc
vvi|miR-577|2mut|as| tcagtccaaccacagtgagc
vvi|miR-578|nat|as| ggagctcccttcagtccaag
vvi|miR-578|2mut|as| ggagctctcttcagttcaag

vvi|miR-579|nat|as| tgcagtgaagggagctccct
vvi|miR-579|2mut|as| tgcagtgaactgagctccct

vvi|miR-580|nat|as| tcagtccaaaacaaggagca
vvi|miR-580|2mut|as| tcagtcaaaaacaagaagca
vvi|miR-581|nat|as| ggagctcccttcagtccaaa
vvi|miR-581|2mut|as| ggagctctcttcagttcaaa

vvi|miR-582|nat|as| agaagaactaggagctccct
vvi|miR-582|2mut|as| agaagaattaggagttccct
vvi|miR-583|nat|as| tcagtccaagcacagacaag
vvi|miR-583|2mut|as| tcagtccaacaacagacaag
vvi|miR-584|nat|as| ggagctcccttcagtccaag
vvi|miR-584|2mut|as| ggagctctcttcagttcaag

vvi|miR-585|nat|as| acagtagaagggagctccct

vvi-miR172d|0_mismatch probe
vvi-miR172d|+10_perfect match probe
vvi-miR172d|+10_mismatch probe
vvi-miR319b|-10_perfect match probe
vvi-miR319b|-10_mismatch probe
vvi-miR319b|0_perfect match probe
vvi-miR319b|0_mismatch probe
vvi-miR319b|+10_perfect match probe
vvi-miR319b|+10_mismatch probe
vvi-miR319c|-10_perfect match probe
vvi-miR319c|-10_mismatch probe
vvi-miR319c|0_perfect match probe
vvi-miR319c|0_mismatch probe
vvi-miR319c|+10_perfect match probe
vvi-miR319c|+10_mismatch probe
vvi-miR319e|-10_perfect match probe
vvi-miR319e|-10_mismatch probe
vvi-miR319e|0_perfect match probe
vvi-miR319e|0_mismatch probe
vvi-miR319e|+10_perfect match probe
vvi-miR319e|+10_mismatch probe
vvi-miR319f|-10_perfect match probe
vvi-miR319f|-10_mismatch probe
vvi-miR319f|0_perfect match probe
vvi-miR319f|0_mismatch probe
vvi-miR319f|+10_perfect match probe

vvi|miR-585|2mut|as|	acagtagaactgagctccct
vvi|miR-586|nat|as|	tcagtccaatgagtgggcaa
vvi|miR-586|2mut|as|	tcagtcaaatgagtgtgcaa
vvi|miR-587|nat|as|	ggagctcccttcagtccaat
vvi|miR-587|2mut|as|	ggagctctcttcagttcaat
vvi|miR-588|nat|as|	atctgccctgggagctccct
vvi|miR-588|2mut|as|	atctgtcctgtgagctccct
vvi|miR-589|nat|as|	tcctgagcttaacagattct
vvi|miR-589|2mut|as|	tcctgagtttaaaagattct
vvi|miR-590|nat|as|	gcgctatccctcctgagctt
vvi|miR-590|2mut|as|	gcgctatctcttctgagctt
vvi|miR-591|nat|as|	atggctcatggcgctatccc
vvi|miR-591|2mut|as|	atggttcatggccctatccc
vvi|miR-592|nat|as|	tccctttggaactgtccacc
vvi|miR-592|2mut|as|	tccctttcgaactgttcacc
vvi|miR-593|nat|as|	atcaatgcgatccctttgga
vvi|miR-593|2mut|as|	atcaatgagatctctttgga
vvi|miR-594|nat|as|	taaccatgggatcaatgcga
vvi|miR-594|2mut|as|	taaccatgtgataaatgcga
vvi|miR-595|nat|as|	tccctttggatgcctcctcc
vvi|miR-595|2mut|as|	tccctttcgatgtctcctcc
vvi|miR-596|nat|as|	atcaatgcgatccctttgga
vvi|miR-596|2mut|as|	atcaatgagatctctttgga
vvi|miR-597|nat|as|	agagtttgggatcaatgcga
vvi|miR-597|2mut|as|	agagtttgtgataaatgcga
vvi|miR-598|nat|as|	agaatgccaaaatggctctg
vvi|miR-598|2mut|as|	agaatgtcaaaatgcctctg

vvi-miR319f|+10_mismatch probe
vvi-miR319g|-10_perfect match probe
vvi-miR319g|-10_mismatch probe
vvi-miR319g|0_perfect match probe
vvi-miR319g|0_mismatch probe
vvi-miR319g|+10_perfect match probe
vvi-miR319g|+10_mismatch probe
vvi-miR390|-10_perfect match probe
vvi-miR390|-10_mismatch probe
vvi-miR390|0_perfect match probe
vvi-miR390|0_mismatch probe
vvi-miR390|+10_perfect match probe
vvi-miR390|+10_mismatch probe
vvi-miR393a|-10_perfect match probe
vvi-miR393a|-10_mismatch probe
vvi-miR393a|0_perfect match probe
vvi-miR393a|0_mismatch probe
vvi-miR393a|+10_perfect match probe
vvi-miR393a|+10_mismatch probe
vvi-miR393b|-10_perfect match probe
vvi-miR393b|-10_mismatch probe
vvi-miR393b|0_perfect match probe
vvi-miR393b|0_mismatch probe
vvi-miR393b|+10_perfect match probe
vvi-miR393b|+10_mismatch probe
vvi-miR394a|-10_perfect match probe
vvi-miR394a|-10_mismatch probe

vvi|miR-599|nat|as|	tgatgtgcatggaggtggac	vvi-miR394a|+10_perfect match probe
vvi|miR-599|2mut|as|	tgatgtgaatcgaggtggac	vvi-miR394a|+10_mismatch probe
vvi|miR-600|nat|as|	agaatgccaataaactctgt	vvi-miR394b|-10_perfect match probe
vvi|miR-600|2mut|as|	agaatgtcaataaattctgt	vvi-miR394b|-10_mismatch probe
vvi|miR-601|nat|as|	tcaagagatgggaggtggac	vvi-miR394b|+10_perfect match probe
vvi|miR-601|2mut|as|	tcaagagatctgaggtggac	vvi-miR394b|+10_mismatch probe
vvi|miR-602|nat|as|	agaatgccaaaatggctctg	vvi-miR394c|-10_perfect match probe
vvi|miR-602|2mut|as|	agaatgtcaaaatgcctctg	vvi-miR394c|-10_mismatch probe
vvi|miR-603|nat|as|	tggtatatatggaggtggac	vvi-miR394c|+10_perfect match probe
vvi|miR-603|2mut|as|	tggtatataccgaggtggac	vvi-miR394c|+10_mismatch probe
vvi|miR-604|nat|as|	aacacttcagtaggaagtca	vvi-miR395a|-10_perfect match probe
vvi|miR-604|2mut|as|	aacacttccgtagaaagtca	vvi-miR395a|-10_mismatch probe
vvi|miR-605|nat|as|	agttcccccaaacacttcag	vvi-miR395a|0_perfect match probe
vvi|miR-605|2mut|as|	agttccctcaaacccttcag	vvi-miR395a|0_mismatch probe
vvi|miR-606|nat|as|	cggtaccaggagttcccccca	vvi-miR395a|+10_perfect match probe
vvi|miR-606|2mut|as|	cggtacaaggagttctccca	vvi-miR395a|+10_mismatch probe
vvi|miR-607|nat|as|	aacacttcagtacgtaggca	vvi-miR395b|-10_perfect match probe
vvi|miR-607|2mut|as|	aacacttccgtacctaggca	vvi-miR395b|-10_mismatch probe
vvi|miR-608|nat|as|	agttcccccaaacacttcag	vvi-miR395b|0_perfect match probe
vvi|miR-608|2mut|as|	agttccctcaaacccttcag	vvi-miR395b|0_mismatch probe
vvi|miR-609|nat|as|	tggcaccaggagttcccccca	vvi-miR395b|+10_perfect match probe
vvi|miR-609|2mut|as|	tggcacaaggagttctccca	vvi-miR395b|+10_mismatch probe
vvi|miR-610|nat|as|	aacacttcagtaggaagtca	vvi-miR395c|-10_perfect match probe
vvi|miR-610|2mut|as|	aacacttccgtagaaagtca	vvi-miR395c|-10_mismatch probe

vvi|miR-611|nat|as|	agttcccccaaacacttcag	vvi-miR395c|0_perfect match probe
vvi|miR-611|2mut|as|	agttccctcaaacccttcag	vvi-miR395c|0_mismatch probe
		vvi-miR395c|+10_perfect match
vvi|miR-612|nat|as|	tggcaccaggagttcccccca	probe
vvi|miR-612|2mut|as|	tggcacaaggagttctccca	vvi-miR395c|+10_mismatch probe
		vvi-miR395d|-10_perfect match
vvi|miR-613|nat|as|	aacacttcagtaggaagtca	probe
vvi|miR-613|2mut|as|	aacacttccgtagaaagtca	vvi-miR395d|-10_mismatch probe
vvi|miR-614|nat|as|	agttcccccaaacacttcag	vvi-miR395d|0_perfect match probe
vvi|miR-614|2mut|as|	agttccctcaaacccttcag	vvi-miR395d|0_mismatch probe
		vvi-miR395d|+10_perfect match
vvi|miR-615|nat|as|	tgacaccaggagttcccccca	probe
vvi|miR-615|2mut|as|	tgacacaaggagttctccca	vvi-miR395d|+10_mismatch probe
		vvi-miR395e|-10_perfect match
vvi|miR-616|nat|as|	aacacttcagtaggaagtca	probe
vvi|miR-616|2mut|as|	aacacttccgtagaaagtca	vvi-miR395e|-10_mismatch probe
vvi|miR-617|nat|as|	agttcccccaaacacttcag	vvi-miR395e|0_perfect match probe
vvi|miR-617|2mut|as|	agttccctcaaacccttcag	vvi-miR395e|0_mismatch probe
		vvi-miR395e|+10_perfect match
vvi|miR-618|nat|as|	tggcaccaggagttcccccca	probe
vvi|miR-618|2mut|as|	tggcacaaggagttctccca	vvi-miR395e|+10_mismatch probe
vvi|miR-619|nat|as|	aacacttcagtgggaagtca	vvi-miR395f|-10_perfect match probe
vvi|miR-619|2mut|as|	aacacttccgtgtgaagtca	vvi-miR395f|-10_mismatch probe
vvi|miR-620|nat|as|	agttcccccaaacacttcag	vvi-miR395f|0_perfect match probe
vvi|miR-620|2mut|as|	agttccctcaaacccttcag	vvi-miR395f|0_mismatch probe
		vvi-miR395f|+10_perfect match
vvi|miR-621|nat|as|	tgacaccaggagttcccccca	probe
vvi|miR-621|2mut|as|	tgacacaaggagttctccca	vvi-miR395f|+10_mismatch probe
		vvi-miR395g|-10_perfect match
vvi|miR-622|nat|as|	aacacttcagtaggaattga	probe
vvi|miR-622|2mut|as|	aacacttccgtagaaattga	vvi-miR395g|-10_mismatch probe
vvi|miR-623|nat|as|	agttcccccaaacacttcag	vvi-miR395g|0_perfect match probe
vvi|miR-623|2mut|as|	agttccctcaaacccttcag	vvi-miR395g|0_mismatch probe
vvi|miR-624|nat|as|	tgacaccgggagttcccccca	vvi-miR395g|+10_perfect match

<table>
<tr><td></td><td></td><td>probe</td></tr>
<tr><td>vvi|miR-624|2mut|as|</td><td>tgacaccaggagttctccca</td><td>vvi-miR395g|+10_mismatch probe</td></tr>
<tr><td>vvi|miR-625|nat|as|</td><td>aacacttcagtaggaagtca</td><td>vvi-miR395h|-10_perfect match probe</td></tr>
<tr><td>vvi|miR-625|2mut|as|</td><td>aacacttccgtagaaagtca</td><td>vvi-miR395h|-10_mismatch probe</td></tr>
<tr><td>vvi|miR-626|nat|as|</td><td>agttcccccaaacacttcag</td><td>vvi-miR395h|0_perfect match probe</td></tr>
<tr><td>vvi|miR-626|2mut|as|</td><td>agttccctcaaacccttcag</td><td>vvi-miR395h|0_mismatch probe</td></tr>
<tr><td>vvi|miR-627|nat|as|</td><td>tagcaccaggagttccccca</td><td>vvi-miR395h|+10_perfect match probe</td></tr>
<tr><td>vvi|miR-627|2mut|as|</td><td>tagcacaaggagttctccca</td><td>vvi-miR395h|+10_mismatch probe</td></tr>
<tr><td>vvi|miR-628|nat|as|</td><td>aacacttcagtaggaagtca</td><td>vvi-miR395i|-10_perfect match probe</td></tr>
<tr><td>vvi|miR-628|2mut|as|</td><td>aacacttccgtagaaagtca</td><td>vvi-miR395i|-10_mismatch probe</td></tr>
<tr><td>vvi|miR-629|nat|as|</td><td>agttcccccaaacacttcag</td><td>vvi-miR395i|0_perfect match probe</td></tr>
<tr><td>vvi|miR-629|2mut|as|</td><td>agttccctcaaacccttcag</td><td>vvi-miR395i|0_mismatch probe</td></tr>
<tr><td>vvi|miR-630|nat|as|</td><td>tgacaccaggagttccccca</td><td>vvi-miR395i|+10_perfect match probe</td></tr>
<tr><td>vvi|miR-630|2mut|as|</td><td>tgacacaaggagttctccca</td><td>vvi-miR395i|+10_mismatch probe</td></tr>
<tr><td>vvi|miR-631|nat|as|</td><td>aacacttcagtaggaagtca</td><td>vvi-miR395j|-10_perfect match probe</td></tr>
<tr><td>vvi|miR-631|2mut|as|</td><td>aacacttccgtagaaagtca</td><td>vvi-miR395j|-10_mismatch probe</td></tr>
<tr><td>vvi|miR-632|nat|as|</td><td>agttcccccaaacacttcag</td><td>vvi-miR395j|0_perfect match probe</td></tr>
<tr><td>vvi|miR-632|2mut|as|</td><td>agttccctcaaacccttcag</td><td>vvi-miR395j|0_mismatch probe</td></tr>
<tr><td>vvi|miR-633|nat|as|</td><td>tgacaccaggagttccccca</td><td>vvi-miR395j|+10_perfect match probe</td></tr>
<tr><td>vvi|miR-633|2mut|as|</td><td>tgacacaaggagttctccca</td><td>vvi-miR395j|+10_mismatch probe</td></tr>
<tr><td>vvi|miR-634|nat|as|</td><td>aacacttcagtaggaagtca</td><td>vvi-miR395k|-10_perfect match probe</td></tr>
<tr><td>vvi|miR-634|2mut|as|</td><td>aacacttccgtagaaagtca</td><td>vvi-miR395k|-10_mismatch probe</td></tr>
<tr><td>vvi|miR-635|nat|as|</td><td>agttcccccaaacacttcag</td><td>vvi-miR395k|0_perfect match probe</td></tr>
<tr><td>vvi|miR-635|2mut|as|</td><td>agttccctcaaacccttcag</td><td>vvi-miR395k|0_mismatch probe</td></tr>
<tr><td>vvi|miR-636|nat|as|</td><td>tggcaccaggagttccccca</td><td>vvi-miR395k|+10_perfect match probe</td></tr>
<tr><td>vvi|miR-636|2mut|as|</td><td>tggcacaaggagttctccca</td><td>vvi-miR395k|+10_mismatch probe</td></tr>
<tr><td>vvi|miR-637|nat|as|</td><td>aacacttcagtaggaagtca</td><td>vvi-miR395l|-10_perfect match probe</td></tr>
<tr><td>vvi|miR-637|2mut|as|</td><td>aacacttccgtagaaagtca</td><td>vvi-miR395l|-10_mismatch probe</td></tr>
</table>

vvi|miR-638|nat|as|	agttcccccaaacacttcag
vvi|miR-638|2mut|as|	agttccctcaaacccttcag
vvi|miR-639|nat|as|	tgacaccaggagttccccca
vvi|miR-639|2mut|as|	tgacacaaggagttctccca
vvi|miR-640|nat|as|	aacacttcagtaggaagtca
vvi|miR-640|2mut|as|	aacacttccgtagaaagtca
vvi|miR-641|nat|as|	agttcccccaaacacttcag
vvi|miR-641|2mut|as|	agttccctcaaacccttcag
vvi|miR-642|nat|as|	tggcaccaggagttccccca
vvi|miR-642|2mut|as|	tggcacaaggagttctccca
vvi|miR-643|nat|as|	gactcttcagtagaggaaat
vvi|miR-643|2mut|as|	gactcttccgtagagaaaat
vvi|miR-644|nat|as|	agttcctccagactcttcag
vvi|miR-644|2mut|as|	agttcctcaagcctcttcag
vvi|miR-645|nat|as|	tggcaccaagagttcctcca
vvi|miR-645|2mut|as|	tggcacaaagagtttctcca
vvi|miR-646|nat|as|	gctgtgggaggacatggcaa
vvi|miR-646|2mut|as|	gctgtgtgaggacatcgcaa
vvi|miR-647|nat|as|	gctcaagaaagctgtgggag
vvi|miR-647|2mut|as|	gctcaacaaagttgtgggag
vvi|miR-648|nat|as|	tgaaatagaagctcaagaaa
vvi|miR-648|2mut|as|	tgaaatacaagttcaagaaa
vvi|miR-649|nat|as|	gctgtgggaaaccatggctt
vvi|miR-649|2mut|as|	gctgtgtgaaaccatcgctt
vvi|miR-650|nat|as|	gttcaagaaagctgtgggaa
vvi|miR-650|2mut|as|	gttcaacaaagttgtgggaa

vvi-miR395l|0_perfect match probe
vvi-miR395l|0_mismatch probe
vvi-miR395l|+10_perfect match probe
vvi-miR395l|+10_mismatch probe
vvi-miR395m|-10_perfect match probe
vvi-miR395m|-10_mismatch probe
vvi-miR395m|0_perfect match probe
vvi-miR395m|0_mismatch probe
vvi-miR395m|+10_perfect match probe
vvi-miR395m|+10_mismatch probe
vvi-miR395n|-10_perfect match probe
vvi-miR395n|-10_mismatch probe
vvi-miR395n|0_perfect match probe
vvi-miR395n|0_mismatch probe
vvi-miR395n|+10_perfect match probe
vvi-miR395n|+10_mismatch probe
vvi-miR396a|-10_perfect match probe
vvi-miR396a|-10_mismatch probe
vvi-miR396a|0_perfect match probe
vvi-miR396a|0_mismatch probe
vvi-miR396a|+10_perfect match probe
vvi-miR396a|+10_mismatch probe
vvi-miR396b|-10_perfect match probe
vvi-miR396b|-10_mismatch probe
vvi-miR396b|0_perfect match probe
vvi-miR396b|0_mismatch probe

vvi|miR-651|nat|as|	atccactgaagttcaagaaa	vvi-miR396b|+10_perfect match probe
vvi|miR-651|2mut|as|	atcccctgaagttaaagaaa	vvi-miR396b|+10_mismatch probe
vvi|miR-652|nat|as|	agctgtggaagatcacagaa	vvi-miR396c|-10_perfect match probe
vvi|miR-652|2mut|as|	agctgtcgaagatcccagaa	vvi-miR396c|-10_mismatch probe
vvi|miR-653|nat|as|	agttcaagaaagctgtggaa	vvi-miR396c|0_perfect match probe
vvi|miR-653|2mut|as|	agttaaagaaagttgtggaa	vvi-miR396c|0_mismatch probe
vvi|miR-654|nat|as|	cttccaaaacagttcaagaa	vvi-miR396c|+10_perfect match probe
vvi|miR-654|2mut|as|	cttcaaaaaccgttcaagaa	vvi-miR396c|+10_mismatch probe
vvi|miR-655|nat|as|	agctgtggaagaatacaaag	vvi-miR396d|-10_perfect match probe
vvi|miR-655|2mut|as|	agctgtcgaagaataaaaag	vvi-miR396d|-10_mismatch probe
vvi|miR-656|nat|as|	agttcaagaaagctgtggaa	vvi-miR396d|0_perfect match probe
vvi|miR-656|2mut|as|	agttaaagaaagttgtggaa	vvi-miR396d|0_mismatch probe
vvi|miR-657|nat|as|	tgaaaaatgcagttcaagaa	vvi-miR396d|+10_perfect match probe
vvi|miR-657|2mut|as|	tgaaaaatgacgttcaagaa	vvi-miR396d|+10_mismatch probe
vvi|miR-658|nat|as|	cactcaatgatgttttcttc	vvi-miR397a|-10_perfect match probe
vvi|miR-658|2mut|as|	cactcaataatcttttcttc	vvi-miR397a|-10_mismatch probe
vvi|miR-659|nat|as|	atcaacgctgcactcaatga	vvi-miR397a|0_perfect match probe
vvi|miR-659|2mut|as|	atcaaccctgaactcaatga	vvi-miR397a|0_mismatch probe
vvi|miR-660|nat|as|	cttcagtttcatcaacgctg	vvi-miR397a|+10_perfect match probe
vvi|miR-660|2mut|as|	cttccgtttcatcaaagctg	vvi-miR397a|+10_mismatch probe
vvi|miR-661|nat|as|	cactcaatgatgttttcttc	vvi-miR397b|-10_perfect match probe
vvi|miR-661|2mut|as|	cactcaataatcttttcttc	vvi-miR397b|-10_mismatch probe
vvi|miR-662|nat|as|	atcaacgctgcactcaatga	vvi-miR397b|0_perfect match probe
vvi|miR-662|2mut|as|	atcaaccctgaactcaatga	vvi-miR397b|0_mismatch probe
vvi|miR-663|nat|as|	cttcagtttcatcaacgctg	vvi-miR397b|+10_perfect match probe

vvi|miR-663|2mut|as|	cttccgtttcatcaaagctg
vvi|miR-664|nat|as|	tgagaacacaaaatgcatta
vvi|miR-664|2mut|as|	tgagaacccaaaatgaatta
vvi|miR-665|nat|as|	aggggtgacctgagaacaca
vvi|miR-665|2mut|as|	aggggtgatccgagaacaca
vvi|miR-666|nat|as|	ggtgccccaaaggggtgacc
vvi|miR-666|2mut|as|	ggtgtcccaaaggtgtgacc
vvi|miR-667|nat|as|	tgagaacacatgagagcaaa
vvi|miR-667|2mut|as|	tgagaacccatgagaccaaa
vvi|miR-668|nat|as|	aggggcgacctgagaacaca
vvi|miR-668|2mut|as|	aggggagacccgagaacaca
vvi|miR-669|nat|as|	ggagtccagcaggggcgacc
vvi|miR-669|2mut|as|	ggagtccagaaggggagacc
vvi|miR-670|nat|as|	tgagaacacatgagagcaaa
vvi|miR-670|2mut|as|	tgagaacccatgagaccaaa
vvi|miR-671|nat|as|	aggggcgacctgagaacaca
vvi|miR-671|2mut|as|	aggggagacccgagaacaca
vvi|miR-672|nat|as|	ggagtccagcaggggcgacc
vvi|miR-672|2mut|as|	ggagtccagaaggggagacc
vvi|miR-673|nat|as|	tcctttggcagagcagctac
vvi|miR-673|2mut|as|	tcctttgccagagaagctac
vvi|miR-674|nat|as|	agggcaattctcctttggca
vvi|miR-674|2mut|as|	agggaaattcttctttggca
vvi|miR-675|nat|as|	attgaataacagggcaattc
vvi|miR-675|2mut|as|	attgaataaaaggccaattc
vvi|miR-676|nat|as|	tcctttggcaagtcacatct

vvi-miR397b|+10_mismatch probe
vvi-miR398a|-10_perfect match probe
vvi-miR398a|-10_mismatch probe
vvi-miR398a|0_perfect match probe
vvi-miR398a|0_mismatch probe
vvi-miR398a|+10_perfect match probe
vvi-miR398a|+10_mismatch probe
vvi-miR398b|-10_perfect match probe
vvi-miR398b|-10_mismatch probe
vvi-miR398b|0_perfect match probe
vvi-miR398b|0_mismatch probe
vvi-miR398b|+10_perfect match probe
vvi-miR398b|+10_mismatch probe
vvi-miR398c|-10_perfect match probe
vvi-miR398c|-10_mismatch probe
vvi-miR398c|0_perfect match probe
vvi-miR398c|0_mismatch probe
vvi-miR398c|+10_perfect match probe
vvi-miR398c|+10_mismatch probe
vvi-miR399a|-10_perfect match probe
vvi-miR399a|-10_mismatch probe
vvi-miR399a|0_perfect match probe
vvi-miR399a|0_mismatch probe
vvi-miR399a|+10_perfect match probe
vvi-miR399a|+10_mismatch probe
vvi-miR399b|-10_perfect match

		probe
vvi|miR-676|2mut|as|	tcctttgccaagtcccatct	vvi-miR399b|-10_mismatch probe
vvi|miR-677|nat|as|	agggcaactctcctttggca	vvi-miR399b|0_perfect match probe
vvi|miR-677|2mut|as|	agggaaactcttctttggca	vvi-miR399b|0_mismatch probe
		vvi-miR399b|+10_perfect match
vvi|miR-678|nat|as|	aagcagtcacagggcaactc	probe
vvi|miR-678|2mut|as|	aagcagtcccaggccaactc	vvi-miR399b|+10_mismatch probe
		vvi-miR399c|-10_perfect match
vvi|miR-679|nat|as|	tcctttggcagatcattcac	probe
vvi|miR-679|2mut|as|	tcctttgccagataattcac	vvi-miR399c|-10_mismatch probe
vvi|miR-680|nat|as|	agggcaactctcctttggca	vvi-miR399c|0_perfect match probe
vvi|miR-680|2mut|as|	agggaaactcttctttggca	vvi-miR399c|0_mismatch probe
		vvi-miR399c|+10_perfect match
vvi|miR-681|nat|as|	aaccagacacagggcaactc	probe
vvi|miR-681|2mut|as|	aaccagacccaggccaactc	vvi-miR399c|+10_mismatch probe
		vvi-miR399d|-10_perfect match
vvi|miR-682|nat|as|	tcctttggcagacacactca	probe
vvi|miR-682|2mut|as|	tcctttgccagacaccctca	vvi-miR399d|-10_mismatch probe
vvi|miR-683|nat|as|	cgagcaaatctcctttggca	vvi-miR399d|0_perfect match probe
vvi|miR-683|2mut|as|	cgagaaaatcttctttggca	vvi-miR399d|0_mismatch probe
		vvi-miR399d|+10_perfect match
vvi|miR-684|nat|as|	gttaaattcacgagcaaatc	probe
vvi|miR-684|2mut|as|	gttaaattcccgagaaaatc	vvi-miR399d|+10_mismatch probe
		vvi-miR399e|-10_perfect match
vvi|miR-685|nat|as|	tcctttggcagagggcacac	probe
vvi|miR-685|2mut|as|	tcctttgccagaggccacac	vvi-miR399e|-10_mismatch probe
vvi|miR-686|nat|as|	cgggcaaatctcctttggca	vvi-miR399e|0_perfect match probe
vvi|miR-686|2mut|as|	cgggaaaatcttctttggca	vvi-miR399e|0_mismatch probe
		vvi-miR399e|+10_perfect match
vvi|miR-687|nat|as|	gaagaattgccgggcaaatc	probe
vvi|miR-687|2mut|as|	gaagaattgtcggccaaatc	vvi-miR399e|+10_mismatch probe
vvi|miR-688|nat|as|	tccttcggcagcgagaagac	vvi-miR399f|-10_perfect match probe
vvi|miR-688|2mut|as|	tccttcgccagagagaagac	vvi-miR399f|-10_mismatch probe
vvi|miR-689|nat|as|	aggacaaatctccttcggca	vvi-miR399f|0_perfect match probe

vvi|miR-689|2mut|as|	aggaaaaatctccttaggca
vvi|miR-690|nat|as|	gaagaattgcaggacaaatc
vvi|miR-690|2mut|as|	gaagaattgaagaacaaatc
vvi|miR-691|nat|as|	tcctttggcagtgagcttag
vvi|miR-691|2mut|as|	tcctttgccagtgagtttag
vvi|miR-692|nat|as|	ggggcaaatctcctttggca
vvi|miR-692|2mut|as|	ggggaaaatcttctttggca
vvi|miR-693|nat|as|	gctgaattgaggggcaaatc
vvi|miR-693|2mut|as|	gctgaattaagggccaaatc
vvi|miR-694|nat|as|	tcctttggcaaaacagaagt
vvi|miR-694|2mut|as|	tcctttgccaaaaaagaagt
vvi|miR-695|nat|as|	agggcaattctcctttggca
vvi|miR-695|2mut|as|	agggaaattcttctttggca
vvi|miR-696|nat|as|	agcgaatggcagggcaattc
vvi|miR-696|2mut|as|	agcgaatgccaggccaattc
vvi|miR-697|nat|as|	tcctttggcgggtcattgga
vvi|miR-697|2mut|as|	tcctttggagtgtcattgga
vvi|miR-698|nat|as|	agggcaactctcctttggcg
vvi|miR-698|2mut|as|	agggaaactcttctttggcg
vvi|miR-699|nat|as|	aagtagtcacagggcaactc
vvi|miR-699|2mut|as|	aagtagtcccaggccaactc
vvi|miR-700|nat|as|	gtgaatctaacgccgtcgaa
vvi|miR-700|2mut|as|	gtgaatttaaccccgtcgaa
vvi|miR-701|nat|as|	gagtttgtgcgtgaatctaa
vvi|miR-701|2mut|as|	gagtttgtgactgaatctaa
vvi|miR-702|nat|as|	cagaactcacgagtttgtgc

vvi-miR399f|0_mismatch probe
vvi-miR399f|+10_perfect match probe
vvi-miR399f|+10_mismatch probe
vvi-miR399g|-10_perfect match probe
vvi-miR399g|-10_mismatch probe
vvi-miR399g|0_perfect match probe
vvi-miR399g|0_mismatch probe
vvi-miR399g|+10_perfect match probe
vvi-miR399g|+10_mismatch probe
vvi-miR399h|-10_perfect match probe
vvi-miR399h|-10_mismatch probe
vvi-miR399h|0_perfect match probe
vvi-miR399h|0_mismatch probe
vvi-miR399h|+10_perfect match probe
vvi-miR399h|+10_mismatch probe
vvi-miR399i|-10_perfect match probe
vvi-miR399i|-10_mismatch probe
vvi-miR399i|0_perfect match probe
vvi-miR399i|0_mismatch probe
vvi-miR399i|+10_perfect match probe
vvi-miR399i|+10_mismatch probe
vvi-miR403a|-10_perfect match probe
vvi-miR403a|-10_mismatch probe
vvi-miR403a|0_perfect match probe
vvi-miR403a|0_mismatch probe
vvi-miR403a|+10_perfect match probe

vvi|miR-702|2mut|as|	cagaactcaaaagtttgtgc	vvi-miR403a|+10_mismatch probe
vvi|miR-703|nat|as|	gtgaatctaacacccctttg	vvi-miR403b|-10_perfect match probe
vvi|miR-703|2mut|as|	gtgaatttaacacctctttg	vvi-miR403b|-10_mismatch probe
vvi|miR-704|nat|as|	gagtttgtgcgtgaatctaa	vvi-miR403b|0_perfect match probe
vvi|miR-704|2mut|as|	gagtttgtgactgaatctaa	vvi-miR403b|0_mismatch probe
vvi|miR-705|nat|as|	gacagatcccgagtttgtgc	vvi-miR403b|+10_perfect match probe
vvi|miR-705|2mut|as|	gacagatccaaagtttgtgc	vvi-miR403b|+10_mismatch probe
vvi|miR-706|nat|as|	gtgaatctaacggcgtcgaa	vvi-miR403c|-10_perfect match probe
vvi|miR-706|2mut|as|	gtgaatttaacggagtcgaa	vvi-miR403c|-10_mismatch probe
vvi|miR-707|nat|as|	gagtttgtgcgtgaatctaa	vvi-miR403c|0_perfect match probe
vvi|miR-707|2mut|as|	gagtttgtgactgaatctaa	vvi-miR403c|0_mismatch probe
vvi|miR-708|nat|as|	gacagatcacgagtttgtgc	vvi-miR403c|+10_perfect match probe
vvi|miR-708|2mut|as|	gacagatcaaaagtttgtgc	vvi-miR403c|+10_mismatch probe
vvi|miR-709|nat|as|	gtgaatctaacacccctttg	vvi-miR403d|-10_perfect match probe
vvi|miR-709|2mut|as|	gtgaatttaacacctctttg	vvi-miR403d|-10_mismatch probe
vvi|miR-710|nat|as|	gagtttgtgcgtgaatctaa	vvi-miR403d|0_perfect match probe
vvi|miR-710|2mut|as|	gagtttgtgactgaatctaa	vvi-miR403d|0_mismatch probe
vvi|miR-711|nat|as|	gacagatcccgagtttgtgc	vvi-miR403d|+10_perfect match probe
vvi|miR-711|2mut|as|	gacagatccaaagtttgtgc	vvi-miR403d|+10_mismatch probe
vvi|miR-712|nat|as|	gtgaatctaacggcgtcgaa	vvi-miR403e|-10_perfect match probe
vvi|miR-712|2mut|as|	gtgaatttaacggagtcgaa	vvi-miR403e|-10_mismatch probe
vvi|miR-713|nat|as|	gagtttgtgcgtgaatctaa	vvi-miR403e|0_perfect match probe
vvi|miR-713|2mut|as|	gagtttgtgactgaatctaa	vvi-miR403e|0_mismatch probe
vvi|miR-714|nat|as|	gacagatcgcgagtttgtgc	vvi-miR403e|+10_perfect match probe
vvi|miR-714|2mut|as|	gacagatcgaaagtttgtgc	vvi-miR403e|+10_mismatch probe
vvi|miR-715|nat|as|	gtgaatctaagaccatagaa	vvi-miR403f|-10_perfect match probe

<table>
<tr><td>vvi|miR-715|2mut|as|</td><td>gtgaatttaagacaatagaa</td><td>vvi-miR403f|-10_mismatch probe</td></tr>
<tr><td>vvi|miR-716|nat|as|</td><td>gagtttgtgcgtgaatctaa</td><td>vvi-miR403f|0_perfect match probe</td></tr>
<tr><td>vvi|miR-716|2mut|as|</td><td>gagtttgtgactgaatctaa</td><td>vvi-miR403f|0_mismatch probe</td></tr>
<tr><td>vvi|miR-717|nat|as|</td><td>gacagattacgagtttgtgc</td><td>vvi-miR403f|+10_perfect match probe</td></tr>
<tr><td>vvi|miR-717|2mut|as|</td><td>gacagattaaaagtttgtgc</td><td>vvi-miR403f|+10_mismatch probe</td></tr>
<tr><td>vvi|miR-718|nat|as|</td><td>ggcagtgcatgggtagagca</td><td>vvi-miR408|-10_perfect match probe</td></tr>
<tr><td>vvi|miR-718|2mut|as|</td><td>ggcagtgaatgtgtagagca</td><td>vvi-miR408|-10_mismatch probe</td></tr>
<tr><td>vvi|miR-719|nat|as|</td><td>ccagggaagaggcagtgcat</td><td>vvi-miR408|0_perfect match probe</td></tr>
<tr><td>vvi|miR-719|2mut|as|</td><td>ccagtgaagagccagtgcat</td><td>vvi-miR408|0_mismatch probe</td></tr>
<tr><td>vvi|miR-720|nat|as|</td><td>agagacagagccagggaaga</td><td>vvi-miR408|+10_perfect match probe</td></tr>
<tr><td>vvi|miR-720|2mut|as|</td><td>agagacagactcagggaaga</td><td>vvi-miR408|+10_mismatch probe</td></tr>
<tr><td>vvi|miR-721|nat|as|</td><td>tgagggagattgacaacttc</td><td>vvi-miR477a|-10_perfect match probe</td></tr>
<tr><td>vvi|miR-721|2mut|as|</td><td>tgagtgagattgaaaacttc</td><td>vvi-miR477a|-10_mismatch probe</td></tr>
<tr><td>vvi|miR-722|nat|as|</td><td>tggaagcctttgagggagat</td><td>vvi-miR477a|0_perfect match probe</td></tr>
<tr><td>vvi|miR-722|2mut|as|</td><td>tggaagtctttgagtgagat</td><td>vvi-miR477a|0_mismatch probe</td></tr>
<tr><td>vvi|miR-723|nat|as|</td><td>ccaggaaaattggaagcctt</td><td>vvi-miR477a|+10_perfect match probe</td></tr>
<tr><td>vvi|miR-723|2mut|as|</td><td>ccagaaaaattggaaccctt</td><td>vvi-miR477a|+10_mismatch probe</td></tr>
<tr><td>vvi|miR-724|nat|as|</td><td>caataccacaccatgtctac</td><td>vvi-miR479|-10_perfect match probe</td></tr>
<tr><td>vvi|miR-724|2mut|as|</td><td>caatacaacacaatgtctac</td><td>vvi-miR479|-10_mismatch probe</td></tr>
<tr><td>vvi|miR-725|nat|as|</td><td>tgagccgaaccaataccaca</td><td>vvi-miR479|0_perfect match probe</td></tr>
<tr><td>vvi|miR-725|2mut|as|</td><td>tgagtcgaacaaataccaca</td><td>vvi-miR479|0_mismatch probe</td></tr>
<tr><td>vvi|miR-726|nat|as|</td><td>tgaaagaagatgagccgaac</td><td>vvi-miR479|+10_perfect match probe</td></tr>
<tr><td>vvi|miR-726|2mut|as|</td><td>tgaaagaacatgagtcgaac</td><td>vvi-miR479|+10_mismatch probe</td></tr>
<tr><td>vvi|miR-727|nat|as|</td><td>gaggagtaggaaagactagc</td><td>vvi-miR482a|-10_perfect match probe</td></tr>
<tr><td>vvi|miR-727|2mut|as|</td><td>gaggagtagaaaagcctagc</td><td>vvi-miR482a|-10_mismatch probe</td></tr>
<tr><td>vvi|miR-728|nat|as|</td><td>ataggaatgggaggagtagg</td><td>vvi-miR482a|0_perfect match probe</td></tr>
<tr><td>vvi|miR-728|2mut|as|</td><td>ataggaatgtgagaagtagg</td><td>vvi-miR482a|0_mismatch probe</td></tr>
<tr><td>vvi|miR-729|nat|as|</td><td>gaaaacaataggaatgg</td><td>vvi-miR482a|+10_perfect match probe</td></tr>
<tr><td>vvi|miR-729|2mut|as|</td><td>gaaaaaaatagaaatgg</td><td>vvi-miR482a|+10_mismatch probe</td></tr>
</table>

vvi|miR-730|nat|as|	ctcgttgtcaagcaattaca	vvi-miR535a|-10_perfect match probe
vvi|miR-730|2mut|as|	ctcgttctcaagaaattaca	vvi-miR535a|-10_mismatch probe
vvi|miR-731|nat|as|	cgtgctctctctcgttgtca	vvi-miR535a|0_perfect match probe
vvi|miR-731|2mut|as|	cgtgttctctctccttgtca	vvi-miR535a|0_mismatch probe
vvi|miR-732|nat|as|	tgctgactagcgtgctctct	vvi-miR535a|+10_perfect match probe
vvi|miR-732|2mut|as|	tgctgactacagtgctctct	vvi-miR535a|+10_mismatch probe
vvi|miR-733|nat|as|	ctcgttgtcaagcaattaca	vvi-miR535b|-10_perfect match probe
vvi|miR-733|2mut|as|	ctcgttctcaagaaattaca	vvi-miR535b|-10_mismatch probe
vvi|miR-734|nat|as|	cgtgctctctctcgttgtca	vvi-miR535b|0_perfect match probe
vvi|miR-734|2mut|as|	cgtgttctctctccttgtca	vvi-miR535b|0_mismatch probe
vvi|miR-735|nat|as|	tgctgactagcgtgctctct	vvi-miR535b|+10_perfect match probe
vvi|miR-735|2mut|as|	tgctgactacagtgctctct	vvi-miR535b|+10_mismatch probe
vvi|miR-736|nat|as|	ctcgttgtcaagcaattaca	vvi-miR535c|-10_perfect match probe
vvi|miR-736|2mut|as|	ctcgttctcaagaaattaca	vvi-miR535c|-10_mismatch probe
vvi|miR-737|nat|as|	cgtgctctctctcgttgtca	vvi-miR535c|0_perfect match probe
vvi|miR-737|2mut|as|	cgtgttctctctccttgtca	vvi-miR535c|0_mismatch probe
vvi|miR-738|nat|as|	tgctgactagcgtgctctct	vvi-miR535c|+10_perfect match probe
vvi|miR-738|2mut|as|	tgctgactacagtgctctct	vvi-miR535c|+10_mismatch probe
vvi|miR-739|nat|as|	ctcgttgtcaagcaattaca	vvi-miR535d|-10_perfect match probe
vvi|miR-739|2mut|as|	ctcgttctcaagaaattaca	vvi-miR535d|-10_mismatch probe
vvi|miR-740|nat|as|	cgtgctctctctcgttgtca	vvi-miR535d|0_perfect match probe
vvi|miR-740|2mut|as|	cgtgttctctctccttgtca	vvi-miR535d|0_mismatch probe
vvi|miR-741|nat|as|	tgcagactagcgtgctctct	vvi-miR535d|+10_perfect match probe
vvi|miR-741|2mut|as|	tgcagactacagtgctctct	vvi-miR535d|+10_mismatch probe
vvi|miR-742|nat|as|	ctcgttgtcaagcaattaca	vvi-miR535e|-10_perfect match probe

<table>
<tr><td>vvi|miR-742|2mut|as|</td><td>ctcgttctcaagaaattaca</td><td>vvi-miR535e|-10_mismatch probe</td></tr>
<tr><td>vvi|miR-743|nat|as|</td><td>cgtgctctctctcgttgtca</td><td>vvi-miR535e|0_perfect match probe</td></tr>
<tr><td>vvi|miR-743|2mut|as|</td><td>cgtgttctctctccttgtca</td><td>vvi-miR535e|0_mismatch probe</td></tr>
<tr><td>vvi|miR-744|nat|as|</td><td>tgcagactagcgtgctctct</td><td>vvi-miR535e|+10_perfect match probe</td></tr>
<tr><td>vvi|miR-744|2mut|as|</td><td>tgcagactacagtgctctct</td><td>vvi-miR535e|+10_mismatch probe</td></tr>
<tr><td>vvi|miR-745|nat|as|</td><td>ttgagcaagaaactttata</td><td>vvi-miR828a|-10_perfect match probe</td></tr>
<tr><td>vvi|miR-745|2mut|as|</td><td>ttgagaaagaaattttata</td><td>vvi-miR828a|-10_mismatch probe</td></tr>
<tr><td>vvi|miR-746|nat|as|</td><td>gaatactcatttgagcaaga</td><td>vvi-miR828a|0_perfect match probe</td></tr>
<tr><td>vvi|miR-746|2mut|as|</td><td>gaatactaatttgagaaaga</td><td>vvi-miR828a|0_mismatch probe</td></tr>
<tr><td>vvi|miR-747|nat|as|</td><td>ctgttgtttggaatactcat</td><td>vvi-miR828a|+10_perfect match probe</td></tr>
<tr><td>vvi|miR-747|2mut|as|</td><td>ctgttgtttcaaatactcat</td><td>vvi-miR828a|+10_mismatch probe</td></tr>
<tr><td>vvi|miR-748|nat|as|</td><td>ttgagcaagaaaccaagagc</td><td>vvi-miR828b|-10_perfect match probe</td></tr>
<tr><td>vvi|miR-748|2mut|as|</td><td>ttgagaaagaaacaaagagc</td><td>vvi-miR828b|-10_mismatch probe</td></tr>
<tr><td>vvi|miR-749|nat|as|</td><td>gaacactcatttgagcaaga</td><td>vvi-miR828b|0_perfect match probe</td></tr>
<tr><td>vvi|miR-749|2mut|as|</td><td>gaaccctcatttgagaaaga</td><td>vvi-miR828b|0_mismatch probe</td></tr>
<tr><td>vvi|miR-750|nat|as|</td><td>ggttgatgtggaacactcat</td><td>vvi-miR828b|+10_perfect match probe</td></tr>
<tr><td>vvi|miR-750|2mut|as|</td><td>ggttgatgtcaaacactcat</td><td>vvi-miR828b|+10_mismatch probe</td></tr>
<tr><td>vvi|miR-751|nat|as|</td><td>atcagagctagacctcttgc</td><td>vvi-miR845a|-10_perfect match probe</td></tr>
<tr><td>vvi|miR-751|2mut|as|</td><td>atcagagttagatctcttgc</td><td>vvi-miR845a|-10_mismatch probe</td></tr>
<tr><td>vvi|miR-752|nat|as|</td><td>atcaattggtatcagagcta</td><td>vvi-miR845a|0_perfect match probe</td></tr>
<tr><td>vvi|miR-752|2mut|as|</td><td>atcaattcgtatccgagcta</td><td>vvi-miR845a|0_mismatch probe</td></tr>
<tr><td>vvi|miR-753|nat|as|</td><td>cctttgttttatcaattggt</td><td>vvi-miR845a|+10_perfect match probe</td></tr>
<tr><td>vvi|miR-753|2mut|as|</td><td>cctttcttttataaattggt</td><td>vvi-miR845a|+10_mismatch probe</td></tr>
<tr><td>vvi|miR-754|nat|as|</td><td>atcagagctagacctcttgc</td><td>vvi-miR845b|-10_perfect match probe</td></tr>
<tr><td>vvi|miR-754|2mut|as|</td><td>atcagagttagatctcttgc</td><td>vvi-miR845b|-10_mismatch probe</td></tr>
<tr><td>vvi|miR-755|nat|as|</td><td>atcaattggtatcagagcta</td><td>vvi-miR845b|0_perfect match probe</td></tr>
</table>

vvi|miR-755|2mut|as|	atcaattcgtatccgagcta
vvi|miR-756|nat|as|	cctttgttttatcaattggt
vvi|miR-756|2mut|as|	cctttcttttataaattggt
vvi|miR-757|nat|as|	atcagagcctcatgtttaat
vvi|miR-757|2mut|as|	atcagagtctcatctttaat
vvi|miR-758|nat|as|	atcaattggtatcagagcct
vvi|miR-758|2mut|as|	atcaattcgtatccgagcct
vvi|miR-759|nat|as|	tggtttatgcatcaattggt
vvi|miR-759|2mut|as|	tggtttatgaataaattggt
vvi|miR-760|nat|as|	atcagagccataggttagta
vvi|miR-760|2mut|as|	atcagagtcatagcttagta
vvi|miR-761|nat|as|	atcaattggtatcagagcca
vvi|miR-761|2mut|as|	atcaattcgtatccgagcca
vvi|miR-762|nat|as|	ggtttttcccatcaattggt
vvi|miR-762|2mut|as|	ggtttttctcataaattggt
vvi|miR-763|nat|as|	atcagagccataggttagta
vvi|miR-763|2mut|as|	atcagagtcatagcttagta
vvi|miR-764|nat|as|	atcaattggtatcagagcca
vvi|miR-764|2mut|as|	atcaattcgtatccgagcca
vvi|miR-765|nat|as|	agtttttcccatcaattggt
vvi|miR-765|2mut|as|	agtttttctcataaattggt
vvi|miR-766|nat|as|	tgacagaagaagagagagcac
vvi|miR-766|2mut|as|	tgaccgaagaagagagcgcac
vvi|miR-767|nat|as|	tgacagaaagagaaatgagcac
vvi|miR-767|2mut|as|	tgaccgaaagagaaataagcac
vvi|miR-768|nat|as|	tgacagatagagagtgagcac
vvi|miR-768|2mut|as|	tgaccgatagagagcgagcac

vvi-miR845b|0_mismatch probe
vvi-miR845b|+10_perfect match probe
vvi-miR845b|+10_mismatch probe
vvi-miR845c|-10_perfect match probe
vvi-miR845c|-10_mismatch probe
vvi-miR845c|0_perfect match probe
vvi-miR845c|0_mismatch probe
vvi-miR845c|+10_perfect match probe
vvi-miR845c|+10_mismatch probe
vvi-miR845d|-10_perfect match probe
vvi-miR845d|-10_mismatch probe
vvi-miR845d|0_perfect match probe
vvi-miR845d|0_mismatch probe
vvi-miR845d|+10_perfect match probe
vvi-miR845d|+10_mismatch probe
vvi-miR845e|-10_perfect match probe
vvi-miR845e|-10_mismatch probe
vvi-miR845e|0_perfect match probe
vvi-miR845e|0_mismatch probe
vvi-miR845e|+10_perfect match probe
vvi-miR845e|+10_mismatch probe
vvi-miR156a|*_perfect match probe
vvi-miR156a|*_mismatch probe
vvi-miR156b|*_perfect match probe
vvi-miR156b|*_mismatch probe
vvi-miR156c|*_perfect match probe
vvi-miR156c|*_mismatch probe

vvi|miR-769|nat|as|	tgacagaaagagaggtgagcac	vvi-miR156d|*_perfect match probe
vvi|miR-769|2mut|as|	tgaccgaaagagagctgagcac	vvi-miR156d|*_mismatch probe
vvi|miR-770|nat|as|	tgacagatagggagtaagcac	vvi-miR156e|*_perfect match probe
vvi|miR-770|2mut|as|	tgacagatagtaagtaagcac	vvi-miR156e|*_mismatch probe
vvi|miR-771|nat|as|	ttgacaggagaagagagaggac	vvi-miR156f|*_perfect match probe
vvi|miR-771|2mut|as|	ttgacagaagaagagagcggac	vvi-miR156f|*_mismatch probe
vvi|miR-772|nat|as|	tgacagaagtctagagagcac	vvi-miR156g|*_perfect match probe
vvi|miR-772|2mut|as|	tgacagaagcctagagcgcac	vvi-miR156g|*_mismatch probe
vvi|miR-773|nat|as|	tgaaaggcgcagaaagaac	vvi-miR156h|*_perfect match probe
vvi|miR-773|2mut|as|	tgaaaggccccgaaagaac	vvi-miR156h|*_mismatch probe
vvi|miR-774|nat|as|	tgacagaagaatagagagcac	vvi-miR156i|*_perfect match probe
vvi|miR-774|2mut|as|	tgaccgaagaatagagcgcac	vvi-miR156i|*_mismatch probe
vvi|miR-775|nat|as|	ctggagcgtaaaggagctccc	vvi-miR159a|*_perfect match probe
vvi|miR-775|2mut|as|	ctggagagtaaaggagttccc	vvi-miR159a|*_mismatch probe
vvi|miR-776|nat|as|	ctggagtgtaaagaagctccca	vvi-miR159b|*_perfect match probe
vvi|miR-776|2mut|as|	ctggagcgtaaagaagttccca	vvi-miR159b|*_mismatch probe
vvi|miR-777|nat|as|	ttggacttcaaggagctcca	vvi-miR159c|*_perfect match probe
vvi|miR-777|2mut|as|	ttggccttcaaggagttcca	vvi-miR159c|*_mismatch probe
vvi|miR-778|nat|as|	tgcatgactcctctgatgcca	vvi-miR160a|*_perfect match probe
vvi|miR-778|2mut|as|	tgcatgacttctccgatgcca	vvi-miR160a|*_mismatch probe
vvi|miR-779|nat|as|	tgcttgactcccctcatgcca	vvi-miR160b|*_perfect match probe
vvi|miR-779|2mut|as|	tgcttgactcttctcatgcca	vvi-miR160b|*_mismatch probe
vvi|miR-780|nat|as|	tgcttggctcctcgcacgcca	vvi-miR160c|*_perfect match probe
vvi|miR-780|2mut|as|	tgcttgcctcctcccacgcca	vvi-miR160c|*_mismatch probe
vvi|miR-781|nat|as|	tgcatggctcctcgcacgcca	vvi-miR160d|*_perfect match probe
vvi|miR-781|2mut|as|	tgcatgcctcctcccacgcca	vvi-miR160d|*_mismatch probe
vvi|miR-782|nat|as|	tgcttgactcccctcatgcca	vvi-miR160e|*_perfect match probe
vvi|miR-782|2mut|as|	tgcttgactcttctcatgcca	vvi-miR160e|*_mismatch probe
vvi|miR-783|nat|as|	tgcatggctcctcatacgcca	vvi-miR160f|*_perfect match probe
vvi|miR-783|2mut|as|	tgcatgcctcctcataagcca	vvi-miR160f|*_mismatch probe
vvi|miR-784|nat|as|	tcgatgaaccgctgcatccag	vvi-miR162|*_perfect match probe
vvi|miR-784|2mut|as|	tcgatgaacccctgaatccag	vvi-miR162|*_mismatch probe
vvi|miR-785|nat|as|	tggagaaggggagcacgtgc	vvi-miR164a|*_perfect match probe
vvi|miR-785|2mut|as|	tggagaaggtgagaacgtgc	vvi-miR164a|*_mismatch probe

vvi|miR-786|nat|as|	ggagagccagggcacatg	vvi-miR164b|*_perfect match probe
vvi|miR-786|2mut|as|	ggagagtcaggccacatg	vvi-miR164b|*_mismatch probe
vvi|miR-787|nat|as|	tggagaaggggagcacgtgca	vvi-miR164d|*_perfect match probe
vvi|miR-787|2mut|as|	tggagaaggtgagaacgtgca	vvi-miR164d|*_mismatch probe
vvi|miR-788|nat|as|	tcgagccagacaacattcccc	vvi-miR166c|*_perfect match probe
vvi|miR-788|2mut|as|	tcgagtcagacaaaattcccc	vvi-miR166c|*_mismatch probe
vvi|miR-789|nat|as|	tcgagccagacaacaatcccct	vvi-miR166d|*_perfect match probe
vvi|miR-789|2mut|as|	tcgagtcagacaacaattccct	vvi-miR166d|*_mismatch probe
vvi|miR-790|nat|as|	tcgagccagacaacattcccc	vvi-miR166e|*_perfect match probe
vvi|miR-790|2mut|as|	tcgagtcagacaaaattcccc	vvi-miR166e|*_mismatch probe
vvi|miR-791|nat|as|	tcgagccagccaacattcccc	vvi-miR166f|*_perfect match probe
vvi|miR-791|2mut|as|	tcgagccagtcaaaattcccc	vvi-miR166f|*_mismatch probe
vvi|miR-792|nat|as|	tcgaaccagacaacattcccct	vvi-miR166g|*_perfect match probe
vvi|miR-792|2mut|as|	tcgaacaagacaacatttccct	vvi-miR166g|*_mismatch probe
vvi|miR-793|nat|as|	tcgaaccagacagcgttcccc	vvi-miR166h|*_perfect match probe
vvi|miR-793|2mut|as|	tcgaacaagacagagttcccc	vvi-miR166h|*_mismatch probe
vvi|miR-794|nat|as|	ttggatcaaacctcattccaa	vvi-miR166a|*_perfect match probe
vvi|miR-794|2mut|as|	ttggatcaaatttcattccaa	vvi-miR166a|*_mismatch probe
vvi|miR-795|nat|as|	tcgagccagccaacattcccc	vvi-miR166b|*_perfect match probe
vvi|miR-795|2mut|as|	tcgagccagtcaaaattcccc	vvi-miR166b|*_mismatch probe
vvi|miR-796|nat|as|	tgaagctgcaagatgacctg	vvi-miR167a|*_perfect match probe
vvi|miR-796|2mut|as|	tgaagctgaaagatgccctg	vvi-miR167a|*_mismatch probe
vvi|miR-797|nat|as|	tgaaactgccacatgatcta	vvi-miR167b|*_perfect match probe
vvi|miR-797|2mut|as|	tgaaactgtcccatgatcta	vvi-miR167b|*_mismatch probe
vvi|miR-798|nat|as|	tgaggctaccacatgatct	vvi-miR167c|*_perfect match probe
vvi|miR-798|2mut|as|	tgagcctaccccatgatct	vvi-miR167c|*_mismatch probe
vvi|miR-799|nat|as|	tgaggctgtcagggcatgaccta	vvi-miR167d|*_perfect match probe
vvi|miR-799|2mut|as|	tgagcctgtcaggccatgaccta	vvi-miR167d|*_mismatch probe
vvi|miR-800|nat|as|	tgaaactgccacatgatct	vvi-miR167e|*_perfect match probe
vvi|miR-800|2mut|as|	tgaaactgtcccatgatct	vvi-miR167e|*_mismatch probe
vvi|miR-801|nat|as|	tcagttgatgcaaggcggga	vvi-miR168|*_perfect match probe
vvi|miR-801|2mut|as|	tcagttgatccaaggaggga	vvi-miR168|*_mismatch probe
vvi|miR-802|nat|as|	agccaaggacaacttgccag	vvi-miR169a|*_perfect match probe
vvi|miR-802|2mut|as|	agccaagaacaacttcccag	vvi-miR169a|*_mismatch probe

vvi|miR-803|nat|as|	tgccaaggagacttgcc	vvi-miR169y|*_perfect match probe
vvi|miR-803|2mut|as|	tgccaagaagccttgcc	vvi-miR169y|*_mismatch probe
vvi|miR-804|nat|as|	gagccaaggatgactaaccg	vvi-miR169b|*_perfect match probe
vvi|miR-804|2mut|as|	gagcaaaggatgcctaaccg	vvi-miR169b|*_mismatch probe
vvi|miR-805|nat|as|	agccaaggacaacttgccgg	vvi-miR169c|*_perfect match probe
vvi|miR-805|2mut|as|	agccaagaacaacttcccgg	vvi-miR169c|*_mismatch probe
vvi|miR-806|nat|as|	agccaaggacaacttgccag	vvi-miR169d|*_perfect match probe
vvi|miR-806|2mut|as|	agccaagaacaacttcccag	vvi-miR169d|*_mismatch probe
vvi|miR-807|nat|as|	ttagccaaggtgactgcctg	vvi-miR169e|*_perfect match probe
vvi|miR-807|2mut|as|	ttagtcaaggtgactccctg	vvi-miR169e|*_mismatch probe
vvi|miR-808|nat|as|	agccaaacacaacttgcc	vvi-miR169f|*_perfect match probe
vvi|miR-808|2mut|as|	agccaaaccaaacttgcc	vvi-miR169f|*_mismatch probe
vvi|miR-809|nat|as|	agccaaagacaacttgcc	vvi-miR169g|*_perfect match probe
vvi|miR-809|2mut|as|	agccaaagcaaacttgcc	vvi-miR169g|*_mismatch probe
vvi|miR-810|nat|as|	gagtcaaggataaccctccg	vvi-miR169h|*_perfect match probe
vvi|miR-810|2mut|as|	gagtcaagaataactctccg	vvi-miR169h|*_mismatch probe
vvi|miR-811|nat|as|	gagacagggataaccagccgt	vvi-miR169i|*_perfect match probe
vvi|miR-811|2mut|as|	gagacagtgataaccacccgt	vvi-miR169i|*_mismatch probe
vvi|miR-812|nat|as|	agccaaggacaacctgccag	vvi-miR169j|*_perfect match probe
vvi|miR-812|2mut|as|	agccaagaacaacctcccag	vvi-miR169j|*_mismatch probe
vvi|miR-813|nat|as|	agccaaggacaacctgccag	vvi-miR169k|*_perfect match probe
vvi|miR-813|2mut|as|	agccaagaacaacctcccag	vvi-miR169k|*_mismatch probe
vvi|miR-814|nat|as|	gagccaagaataactagccgt	vvi-miR169l|*_perfect match probe
vvi|miR-814|2mut|as|	gagcaaagaataactacccgt	vvi-miR169l|*_mismatch probe
vvi|miR-815|nat|as|	gagcctcggatgcttgccag	vvi-miR169m|*_perfect match probe
vvi|miR-815|2mut|as|	gagcctcagatgcttcccag	vvi-miR169m|*_mismatch probe
vvi|miR-816|nat|as|	gagcctcagatgcttgccagttatg	vvi-miR169n|*_perfect match probe
vvi|miR-816|2mut|as|	gagcctcagatccttgtcagttatg	vvi-miR169n|*_mismatch probe
vvi|miR-817|nat|as|	gagccaagaataactcgctgtc	vvi-miR169o|*_perfect match probe
vvi|miR-817|2mut|as|	gagcaaagaataactccctgtc	vvi-miR169o|*_mismatch probe
vvi|miR-818|nat|as|	gagcctcggatgcttgccag	vvi-miR169p|*_perfect match probe
vvi|miR-818|2mut|as|	gagcctcagatgcttcccag	vvi-miR169p|*_mismatch probe
vvi|miR-819|nat|as|	gagccaggattacatgccgg	vvi-miR169q|*_perfect match probe
vvi|miR-819|2mut|as|	gagcaaggattacatcccgg	vvi-miR169q|*_mismatch probe

vvi|miR-820|nat|as|	tgagtcaagtcaacttgccg	vvi-miR169r|*_perfect match probe
vvi|miR-820|2mut|as|	tgagtcaagcaaacttgccg	vvi-miR169r|*_mismatch probe
vvi|miR-821|nat|as|	agccaggaacaacttgccgg	vvi-miR169s|*_perfect match probe
vvi|miR-821|2mut|as|	agccagaaacaacttcccgg	vvi-miR169s|*_mismatch probe
vvi|miR-822|nat|as|	gagtcaagtcaacttgccg	vvi-miR169t|*_perfect match probe
vvi|miR-822|2mut|as|	gagtcaagccaatttgccg	vvi-miR169t|*_mismatch probe
vvi|miR-823|nat|as|	gagtcaagtcaacttgccg	vvi-miR169u|*_perfect match probe
vvi|miR-823|2mut|as|	gagtcaagccaatttgccg	vvi-miR169u|*_mismatch probe
vvi|miR-824|nat|as|	agccaagaaataaattgccga	vvi-miR169v|*_perfect match probe
vvi|miR-824|2mut|as|	agccaacaaataaattcccga	vvi-miR169v|*_mismatch probe
vvi|miR-825|nat|as|	agctaagaatgactcgccga	vvi-miR169w|*_perfect match probe
vvi|miR-825|2mut|as|	agctaacaatgactccccga	vvi-miR169w|*_mismatch probe
vvi|miR-826|nat|as|	tagccgaaggaatgactgccta	vvi-miR169x|*_perfect match probe
vvi|miR-826|2mut|as|	tagcagaaggaatgactcccta	vvi-miR169x|*_mismatch probe
vvi|miR-827|nat|as|	ttgagccgtgccaatatcatg	vvi-miR171a|*_perfect match probe
vvi|miR-827|2mut|as|	ttgagccgtgtaaatatcatg	vvi-miR171a|*_mismatch probe
vvi|miR-828|nat|as|	aattgaggcacgccaatacc	vvi-miR171b|*_perfect match probe
vvi|miR-828|2mut|as|	aattgagccaccccaatacc	vvi-miR171b|*_mismatch probe
vvi|miR-829|nat|as|	ttgaaccgcaccaatatcccg	vvi-miR171c|*_perfect match probe
vvi|miR-829|2mut|as|	ttgaaccccacaaatatcccg	vvi-miR171c|*_mismatch probe
vvi|miR-830|nat|as|	ttgaaccgtatcaatatctcg	vvi-miR171d|*_perfect match probe
vvi|miR-830|2mut|as|	ttgaacagtataaatatctcg	vvi-miR171d|*_mismatch probe
vvi|miR-831|nat|as|	gattgaacctcaccaacatc	vvi-miR171e|*_perfect match probe
vvi|miR-831|2mut|as|	gattgaatctcacaaacatc	vvi-miR171e|*_mismatch probe
vvi|miR-832|nat|as|	ttgaacctcaccaacatcgct	vvi-miR171f|*_perfect match probe
vvi|miR-832|2mut|as|	ttgaacctcataaacatcgct	vvi-miR171f|*_mismatch probe
vvi|miR-833|nat|as|	ccgatggaaccaacat	vvi-miR171g|*_perfect match probe
vvi|miR-833|2mut|as|	ccgatcgaacaaacat	vvi-miR171g|*_mismatch probe
vvi|miR-834|nat|as|	ggttgaaccgcaccaacatc	vvi-miR171h|*_perfect match probe
vvi|miR-834|2mut|as|	ggttgaacccaaccaacatc	vvi-miR171h|*_mismatch probe
vvi|miR-835|nat|as|	ttgagccattccaacatcccc	vvi-miR171i|*_perfect match probe
vvi|miR-835|2mut|as|	ttgagtcattcaaacatcccc	vvi-miR171i|*_mismatch probe
vvi|miR-836|nat|as|	gaatcttgatgatgctgcat	vvi-miR172a|*_perfect match probe
vvi|miR-836|2mut|as|	gaatcttaatgatcctgcat	vvi-miR172a|*_mismatch probe

vvi|miR-837|nat|as|	gaatcttgatgatgctgca	vvi-miR172b|*_perfect match probe
vvi|miR-837|2mut|as|	gaatcttaatgatcctgca	vvi-miR172b|*_mismatch probe
vvi|miR-838|nat|as|	gaatcttgatgatgctcca	vvi-miR172c|*_perfect match probe
vvi|miR-838|2mut|as|	gaatcttaatgatcctcca	vvi-miR172c|*_mismatch probe
vvi|miR-839|nat|as|	gaatcttgatgatgctgcat	vvi-miR172d|*_perfect match probe
vvi|miR-839|2mut|as|	gaatcttaatgatcctgcat	vvi-miR172d|*_mismatch probe
vvi|miR-840|nat|as|	ctggactaaagagagctcc	vvi-miR319b|*_perfect match probe
vvi|miR-840|2mut|as|	ctggcctaaagagacctcc	vvi-miR319b|*_mismatch probe
vvi|miR-841|nat|as|	tggactgaagaaagctctc	vvi-miR319c|*_perfect match probe
vvi|miR-841|2mut|as|	tggaccgaagaaagttctc	vvi-miR319c|*_mismatch probe
vvi|miR-842|nat|as|	tttgggctgcaaaggaaccccc	vvi-miR319e|*_perfect match probe
vvi|miR-842|2mut|as|	tttggcctgcaaaggaatcccc	vvi-miR319e|*_mismatch probe
vvi|miR-843|nat|as|	tggactgaaggaagctctc	vvi-miR319f|*_perfect match probe
vvi|miR-843|2mut|as|	tggactgaacgaagttctc	vvi-miR319f|*_mismatch probe
vvi|miR-844|nat|as|	attggactaaaaggagctc	vvi-miR319g|*_perfect match probe
vvi|miR-844|2mut|as|	attgaactaaaagaagctc	vvi-miR319g|*_mismatch probe
vvi|miR-845|nat|as|	aaactcaggatagatagcgcc	vvi-miR390|*_perfect match probe
vvi|miR-845|2mut|as|	aaactcagaatagataccgcc	vvi-miR390|*_mismatch probe
vvi|miR-846|nat|as|	tcctaagggatagcatgatcc	vvi-miR393a|*_perfect match probe
vvi|miR-846|2mut|as|	tcctaagtgatagaatgatcc	vvi-miR393a|*_mismatch probe
vvi|miR-847|nat|as|	tccaaagggatagcatgatcc	vvi-miR393b|*_perfect match probe
vvi|miR-847|2mut|as|	tccaaagtgatagaatgatcc	vvi-miR393b|*_mismatch probe
vvi|miR-848|nat|as|	ttggcatgctggccacctcc	vvi-miR394a|*_perfect match probe
vvi|miR-848|2mut|as|	ttggcatcctggtcacctcc	vvi-miR394a|*_mismatch probe
vvi|miR-849|nat|as|	ttggcagtatgcccacctcc	vvi-miR394b|*_perfect match probe
vvi|miR-849|2mut|as|	ttggaagtatgtccacctcc	vvi-miR394b|*_mismatch probe
vvi|miR-850|nat|as|	ttggcatcctggccgcctcc	vvi-miR394c|*_perfect match probe
vvi|miR-850|2mut|as|	ttggaatcctggccccctcc	vvi-miR394c|*_mismatch probe
vvi|miR-851|nat|as|	tgaagtgatcaagggaactc	vvi-miR395a|*_perfect match probe
vvi|miR-851|2mut|as|	tgaagcgatcaagtgaactc	vvi-miR395a|*_mismatch probe
vvi|miR-852|nat|as|	tgaagtggtaaggggaactc	vvi-miR395b|*_perfect match probe
vvi|miR-852|2mut|as|	tgaagtcgtaaggtgaactc	vvi-miR395b|*_mismatch probe
vvi|miR-853|nat|as|	tgaagtggtcaagggaactc	vvi-miR395c|*_perfect match probe
vvi|miR-853|2mut|as|	tgaagtcgtcaagtgaactc	vvi-miR395c|*_mismatch probe

vvi|miR-854|nat|as|	tgaagtggtcaggggaactc	vvi-miR395d|*_perfect match probe
vvi|miR-854|2mut|as|	tgaagtcgtcaggtgaactc	vvi-miR395d|*_mismatch probe
vvi|miR-855|nat|as|	tgaagtggtcaagggaactc	vvi-miR395e|*_perfect match probe
vvi|miR-855|2mut|as|	tgaagtcgtcaagtgaactc	vvi-miR395e|*_mismatch probe
vvi|miR-856|nat|as|	tgaagtggtcaggggaactc	vvi-miR395f|*_perfect match probe
vvi|miR-856|2mut|as|	tgaagtcgtcaggtgaactc	vvi-miR395f|*_mismatch probe
vvi|miR-857|nat|as|	tgaagtgctcaggggaactc	vvi-miR395g|*_perfect match probe
vvi|miR-857|2mut|as|	tgaagtcctcaggtgaactc	vvi-miR395g|*_mismatch probe
vvi|miR-858|nat|as|	tgaagtggtcaagggaactc	vvi-miR395h|*_perfect match probe
vvi|miR-858|2mut|as|	tgaagtcgtcaagtgaactc	vvi-miR395h|*_mismatch probe
vvi|miR-859|nat|as|	tgaagtggtcaggggaactc	vvi-miR395i|*_perfect match probe
vvi|miR-859|2mut|as|	tgaagtcgtcaggtgaactc	vvi-miR395i|*_mismatch probe
vvi|miR-860|nat|as|	tgaagtggtcaggggaactc	vvi-miR395j|*_perfect match probe
vvi|miR-860|2mut|as|	tgaagtcgtcaggtgaactc	vvi-miR395j|*_mismatch probe
vvi|miR-861|nat|as|	tgaagtggtcaagggaactc	vvi-miR395k|*_perfect match probe
vvi|miR-861|2mut|as|	tgaagtcgtcaagtgaactc	vvi-miR395k|*_mismatch probe
vvi|miR-862|nat|as|	tgaagtggtcaggggaactc	vvi-miR395l|*_perfect match probe
vvi|miR-862|2mut|as|	tgaagtcgtcaggtgaactc	vvi-miR395l|*_mismatch probe
vvi|miR-863|nat|as|	tgaagtgttcaagggaactc	vvi-miR395m|*_perfect match probe
vvi|miR-863|2mut|as|	tgaagcgttcaagtgaactc	vvi-miR395m|*_mismatch probe
vvi|miR-864|nat|as|	ctggaggggttgggggaactc	vvi-miR395n|*_perfect match probe
vvi|miR-864|2mut|as|	ctggaggtgttgggtgaactc	vvi-miR395n|*_mismatch probe
vvi|miR-865|nat|as|	ccacagctttcttgaact	vvi-miR396a|*_perfect match probe
vvi|miR-865|2mut|as|	ccacagttttcttaaact	vvi-miR396a|*_mismatch probe
vvi|miR-866|nat|as|	tttccacagctttcttgaactt	vvi-miR396b|*_perfect match probe
vvi|miR-866|2mut|as|	tttccacagttttcttaaactt	vvi-miR396b|*_mismatch probe
vvi|miR-867|nat|as|	ttccacaactttcctgagctg	vvi-miR396c|*_perfect match probe
vvi|miR-867|2mut|as|	ttccccaacttttctgagctg	vvi-miR396c|*_mismatch probe
vvi|miR-868|nat|as|	tcccacagctttattgaaccg	vvi-miR396d|*_perfect match probe
vvi|miR-868|2mut|as|	tcccacagttttattaaaccg	vvi-miR396d|*_mismatch probe
vvi|miR-869|nat|as|	tgattgagtgcagcgccaatg	vvi-miR397a|*_perfect match probe
vvi|miR-869|2mut|as|	tgattgagtgaagccccaatg	vvi-miR397a|*_mismatch probe
vvi|miR-870|nat|as|	tgattgagtgcagcgccaatg	vvi-miR397b|*_perfect match probe
vvi|miR-870|2mut|as|	tgattgagtgaagccccaatg	vvi-miR397b|*_mismatch probe

vvi|miR-871|nat|as|	gtgttctcaggtgccactccct	vvi-miR398a|*_perfect match probe
vvi|miR-871|2mut|as|	gtgttctcagctgtcactccct	vvi-miR398a|*_mismatch probe
vvi|miR-872|nat|as|	tgtgattctcaggtcacacctg	vvi-miR398b|*_perfect match probe
vvi|miR-872|2mut|as|	tgtgattctccgctcacacctg	vvi-miR398b|*_mismatch probe
vvi|miR-873|nat|as|	tgtgattctcaggtcactcctg	vvi-miR398c|*_perfect match probe
vvi|miR-873|2mut|as|	tgtgattctccgctcactcctg	vvi-miR398c|*_mismatch probe
vvi|miR-874|nat|as|	tgccaaaggagaatcacactg	vvi-miR399a|*_perfect match probe
vvi|miR-874|2mut|as|	tgccaaagaagaatcccactg	vvi-miR399a|*_mismatch probe
vvi|miR-875|nat|as|	tgccaagaaagaggtgccccta	vvi-miR399b|*_perfect match probe
vvi|miR-875|2mut|as|	tgccaagaaacaggtgtccta	vvi-miR399b|*_mismatch probe
vvi|miR-876|nat|as|	taccaagtgaaagaggccctg	vvi-miR399c|*_perfect match probe
vvi|miR-876|2mut|as|	taccaagcgaaagaggtcctg	vvi-miR399c|*_mismatch probe
vvi|miR-877|nat|as|	tgccaaaagaaatctgctc	vvi-miR399d|*_perfect match probe
vvi|miR-877|2mut|as|	tgccaaaacaaatccgctc	vvi-miR399d|*_mismatch probe
vvi|miR-878|nat|as|	tgccaaaagataatttgccc	vvi-miR399e|*_perfect match probe
vvi|miR-878|2mut|as|	tgccaaaacataattcgccc	vvi-miR399e|*_mismatch probe
vvi|miR-879|nat|as|	tgccaaagcagatttggccctctaatg	vvi-miR399f|*_perfect match probe
vvi|miR-879|2mut|as|	tgccaaagaagatttggtcctctaatg	vvi-miR399f|*_mismatch probe
vvi|miR-880|nat|as|	tgccaatggagtattgccc	vvi-miR399g|*_perfect match probe
vvi|miR-880|2mut|as|	tgccaatcgactattgccc	vvi-miR399g|*_mismatch probe
vvi|miR-881|nat|as|	tgccaaaggaggattgcactg	vvi-miR399h|*_perfect match probe
vvi|miR-881|2mut|as|	tgccaaagaaggattgaactg	vvi-miR399h|*_mismatch probe
vvi|miR-882|nat|as|	gccagaaggagagaagccccta	vvi-miR399i|*_perfect match probe
vvi|miR-882|2mut|as|	gccagaagaagagaagtccta	vvi-miR399i|*_mismatch probe
vvi|miR-883|nat|as|	tttgattcacgcacaaact	vvi-miR403a|*_perfect match probe
vvi|miR-883|2mut|as|	tttgattcaaccacaaact	vvi-miR403a|*_mismatch probe
vvi|miR-884|nat|as|	ttggattcgcgcacaaactcg	vvi-miR403b|*_perfect match probe
vvi|miR-884|2mut|as|	ttggattcgccaacaaactcg	vvi-miR403b|*_mismatch probe
vvi|miR-885|nat|as|	tttgattcacgcacaaacttg	vvi-miR403c|*_perfect match probe
vvi|miR-885|2mut|as|	tttgattcaccaacaaacttg	vvi-miR403c|*_mismatch probe
vvi|miR-886|nat|as|	gattcgcgcacaaactcg	vvi-miR403d|*_perfect match probe
vvi|miR-886|2mut|as|	gattcgcccccaaactcg	vvi-miR403d|*_mismatch probe
vvi|miR-887|nat|as|	tttgattcacgcacaaacttg	vvi-miR403e|*_perfect match probe
vvi|miR-887|2mut|as|	tttgattcaccaacaaacttg	vvi-miR403e|*_mismatch probe

vvi|miR-888|nat|as|	ttagagtcacgcacaaactc	vvi-miR403f|*_perfect match probe
vvi|miR-888|2mut|as|	ttagagtcaaccacaaactc	vvi-miR403f|*_mismatch probe
vvi|miR-889|nat|as|	atgcactacctcgtccccg	vvi-miR408|*_perfect match probe
vvi|miR-889|2mut|as|	atgcactatctcctccccg	vvi-miR408|*_mismatch probe
vvi|miR-890|nat|as|	aggtcccccactgtcttccaa	vvi-miR477a|*_perfect match probe
vvi|miR-890|2mut|as|	aggtccctcactctcttccaa	vvi-miR477a|*_mismatch probe
vvi|miR-891|nat|as|	gtgatattggttcggctcgtc	vvi-miR479|*_perfect match probe
vvi|miR-891|2mut|as|	gtgatattcgttcgcctcgtc	vvi-miR479|*_mismatch probe
vvi|miR-892|nat|as|	cctactctccaattcc	vvi-miR482a|*_perfect match probe
vvi|miR-892|2mut|as|	cctattctcaaattcc	vvi-miR482a|*_mismatch probe
vvi|miR-893|nat|as|	tgacagcgagagagagcacgcc	vvi-miR535a|*_perfect match probe
vvi|miR-893|2mut|as|	tgacagagagagagagaacgcc	vvi-miR535a|*_mismatch probe
vvi|miR-894|nat|as|	tgacagcgagagagagcacgcc	vvi-miR535b|*_perfect match probe
vvi|miR-894|2mut|as|	tgacagagagagagagaacgcc	vvi-miR535b|*_mismatch probe
vvi|miR-895|nat|as|	tgacagcgagagagagcacgcc	vvi-miR535c|*_perfect match probe
vvi|miR-895|2mut|as|	tgacagagagagagagaacgcc	vvi-miR535c|*_mismatch probe
vvi|miR-896|nat|as|	tgacagcgacagagagcacgcc	vvi-miR535d|*_perfect match probe
vvi|miR-896|2mut|as|	tgacagagacagagagaacgcc	vvi-miR535d|*_mismatch probe
vvi|miR-897|nat|as|	tgacagcgacagagagcacgcc	vvi-miR535e|*_perfect match probe
vvi|miR-897|2mut|as|	tgacagagacagagagaacgcc	vvi-miR535e|*_mismatch probe
vvi|miR-898|nat|as|	cttcctcaaatgagcatctca	vvi-miR828a|*_perfect match probe
vvi|miR-898|2mut|as|	cttcttcaaatgagaatctca	vvi-miR828a|*_mismatch probe
vvi|miR-899|nat|as|	ttgcaggaaatgactatcca	vvi-miR828b|*_perfect match probe
vvi|miR-899|2mut|as|	ttgcagaaaatgcctatcca	vvi-miR828b|*_mismatch probe
vvi|miR-900|nat|as|	taactcatgaaggaactaat	vvi-miR845a|*_perfect match probe
vvi|miR-900|2mut|as|	taactcataaagaaactaat	vvi-miR845a|*_mismatch probe
vvi|miR-901|nat|as|	aactcatgaaggaactaat	vvi-miR845b|*_perfect match probe
vvi|miR-901|2mut|as|	aactcataaagaaactaat	vvi-miR845b|*_mismatch probe
vvi|miR-902|nat|as|	aaactcagacaaccaaaaca	vvi-miR845c|*_perfect match probe
vvi|miR-902|2mut|as|	aaactcagaaaacaaaaaca	vvi-miR845c|*_mismatch probe
vvi|miR-903|nat|as|	tggccctagggataattaatg	vvi-miR845d|*_perfect match probe
vvi|miR-903|2mut|as|	tggccctagtgctaattaatg	vvi-miR845d|*_mismatch probe
vvi|miR-904|nat|as|	tggccctagggataattaatg	vvi-miR845e|*_perfect match probe
vvi|miR-904|2mut|as|	tggccctagtgctaattaatg	vvi-miR845e|*_mismatch probe

vvi|miR-905|nat|as|	aagaagagagagcacactcc	vvi-miR156a|*-5_perfect match probe
vvi|miR-905|2mut|as|	aagaagagcgagaacactcc	vvi-miR156a|*-5_mismatch probe
vvi|miR-906|nat|as|	gatgatgacagaagaagaga	vvi-miR156a|*+5_perfect match probe
vvi|miR-906|2mut|as|	gatgatgaaagaacaagaga	vvi-miR156a|*+5_mismatch probe
vvi|miR-907|nat|as|	aagagaaatgagcacgcaaa	vvi-miR156b|*-5_perfect match probe
vvi|miR-907|2mut|as|	aagagaaataagcacccaaa	vvi-miR156b|*-5_mismatch probe
vvi|miR-908|nat|as|	gaagctgacagaaagagaaa	vvi-miR156b|*+5_perfect match probe
vvi|miR-908|2mut|as|	gaagttgacagaaagcgaaa	vvi-miR156b|*+5_mismatch probe
vvi|miR-909|nat|as|	atagagagtgagcacgcaga	vvi-miR156c|*-5_perfect match probe
vvi|miR-909|2mut|as|	atagagagcgagcacccaga	vvi-miR156c|*-5_mismatch probe
vvi|miR-910|nat|as|	acagatgacagatagagagt	vvi-miR156c|*+5_perfect match probe
vvi|miR-910|2mut|as|	acagatgaaagatagcgagt	vvi-miR156c|*+5_mismatch probe
vvi|miR-911|nat|as|	aagagaggtgagcacgcacc	vvi-miR156d|*-5_perfect match probe
vvi|miR-911|2mut|as|	aagagagctgagcacccacc	vvi-miR156d|*-5_mismatch probe
vvi|miR-912|nat|as|	gaagctgacagaaagagagg	vvi-miR156d|*+5_perfect match probe
vvi|miR-912|2mut|as|	gaagttgacagaaagcgagg	vvi-miR156d|*+5_mismatch probe
vvi|miR-913|nat|as|	atagggagtaagcacacaga	vvi-miR156e|*-5_perfect match probe
vvi|miR-913|2mut|as|	atagtgagtaagaacacaga	vvi-miR156e|*-5_mismatch probe
vvi|miR-914|nat|as|	aggggtgacagatagggagt	vvi-miR156e|*+5_perfect match probe
vvi|miR-914|2mut|as|	agggctgacagatagtgagt	vvi-miR156e|*+5_mismatch probe
vvi|miR-915|nat|as|	gagaagagagaggacaagac	vvi-miR156f|*-5_perfect match probe
vvi|miR-915|2mut|as|	gagaagagcgagaacaagac	vvi-miR156f|*-5_mismatch probe
vvi|miR-916|nat|as|	ttctgttgacaggagaagag	vvi-miR156f|*+5_perfect match probe

vvi|miR-916|2mut|as|	ttctgttgaaagaagaagag
vvi|miR-917|nat|as|	aagtctagagagcacaaagg
vvi|miR-917|2mut|as|	aagtctagcgagaacaaagg
vvi|miR-918|nat|as|	ggtgatgacagaagtctaga
vvi|miR-918|2mut|as|	ggtgatgaaagaagcctaga
vvi|miR-919|nat|as|	aggcgcagaaagaactcagc
vvi|miR-919|2mut|as|	aggcccagaaagaattcagc
vvi|miR-920|nat|as|	cacaatgaaaggcgcagaaa
vvi|miR-920|2mut|as|	cacaataaaaggcccagaaa
vvi|miR-921|nat|as|	aagaatagagagcacaaagg
vvi|miR-921|2mut|as|	aagaatagcgagaacaaagg
vvi|miR-922|nat|as|	ggtgatgacagaagaataga
vvi|miR-922|2mut|as|	ggtgatgaaagaacaataga
vvi|miR-923|nat|as|	cgtaaaggagctcccataaa
vvi|miR-923|2mut|as|	cgtaaaggacctctcataaa
vvi|miR-924|nat|as|	cagaactggagcgtaaagga
vvi|miR-924|2mut|as|	cagaactcgagagtaaagga
vvi|miR-925|nat|as|	gtaaagaagctcccacaagc
vvi|miR-925|2mut|as|	gtaaagaagttctcacaagc
vvi|miR-926|nat|as|	cagttctggagtgtaaagaa
vvi|miR-926|2mut|as|	cagttctcgagcgtaaagaa
vvi|miR-927|nat|as|	cttcaaggagctccactcca
vvi|miR-927|2mut|as|	cttcaaggacttccactcca
vvi|miR-928|nat|as|	ctctattggacttcaaggag

vvi-miR156f|*+5_mismatch probe
vvi-miR156g|*-5_perfect match probe
vvi-miR156g|*-5_mismatch probe
vvi-miR156g|*+5_perfect match probe
vvi-miR156g|*+5_mismatch probe
vvi-miR156h|*-5_perfect match probe
vvi-miR156h|*-5_mismatch probe
vvi-miR156h|*+5_perfect match probe
vvi-miR156h|*+5_mismatch probe
vvi-miR156i|*-5_perfect match probe
vvi-miR156i|*-5_mismatch probe
vvi-miR156i|*+5_perfect match probe
vvi-miR156i|*+5_mismatch probe
vvi-miR159a|*-5_perfect match probe
vvi-miR159a|*-5_mismatch probe
vvi-miR159a|*+5_perfect match probe
vvi-miR159a|*+5_mismatch probe
vvi-miR159b|*-5_perfect match probe
vvi-miR159b|*-5_mismatch probe
vvi-miR159b|*+5_perfect match probe
vvi-miR159b|*+5_mismatch probe
vvi-miR159c|*-5_perfect match probe
vvi-miR159c|*-5_mismatch probe
vvi-miR159c|*+5_perfect match

		probe
vvi|miR-928|2mut|as|	ctctattcgacttaaaggag	vvi-miR159c|*+5_mismatch probe
		vvi-miR160a|*-5_perfect match
vvi|miR-929|nat|as|	actcctctgatgccaactag	probe
vvi|miR-929|2mut|as|	actcttctgatgtcaactag	vvi-miR160a|*-5_mismatch probe
		vvi-miR160a|*+5_perfect match
vvi|miR-930|nat|as|	gggcctgcatgactcctctg	probe
vvi|miR-930|2mut|as|	gggcctgaatgacttctctg	vvi-miR160a|*+5_mismatch probe
		vvi-miR160b|*-5_perfect match
vvi|miR-931|nat|as|	actcccctcatgccaaatag	probe
vvi|miR-931|2mut|as|	actcctctcatgtcaaatag	vvi-miR160b|*-5_mismatch probe
		vvi-miR160b|*+5_perfect match
vvi|miR-932|nat|as|	tcgcctgcttgactcccctc	probe
vvi|miR-932|2mut|as|	tcgcctccttgactctcctc	vvi-miR160b|*+5_mismatch probe
		vvi-miR160c|*-5_perfect match
vvi|miR-933|nat|as|	gctcctcgcacgccacccgc	probe
vvi|miR-933|2mut|as|	gctcctcccaccccacccgc	vvi-miR160c|*-5_mismatch probe
		vvi-miR160c|*+5_perfect match
vvi|miR-934|nat|as|	gggtatgcttggctcctcgc	probe
vvi|miR-934|2mut|as|	gggtatccttgcctcctcgc	vvi-miR160c|*+5_mismatch probe
		vvi-miR160d|*-5_perfect match
vvi|miR-935|nat|as|	gctcctcgcacgccatccgc	probe
vvi|miR-935|2mut|as|	gctcctcccaccccatccgc	vvi-miR160d|*-5_mismatch probe
		vvi-miR160d|*+5_perfect match
vvi|miR-936|nat|as|	atgcatgcatggctcctcgc	probe
vvi|miR-936|2mut|as|	atgcatgaatgcctcctcgc	vvi-miR160d|*+5_mismatch probe
		vvi-miR160e|*-5_perfect match
vvi|miR-937|nat|as|	actcccctcatgccaaatag	probe
vvi|miR-937|2mut|as|	actcctctcatgtcaaatag	vvi-miR160e|*-5_mismatch probe
		vvi-miR160e|*+5_perfect match
vvi|miR-938|nat|as|	tcgcctgcttgactcccctc	probe
vvi|miR-938|2mut|as|	tcgcctccttgactctcctc	vvi-miR160e|*+5_mismatch probe
vvi|miR-939|nat|as|	gctcctcatacgccatccac	vvi-miR160f|*-5_perfect match probe
vvi|miR-939|2mut|as|	gctcttcataccccatccac	vvi-miR160f|*-5_mismatch probe

<table>
<tr><td>vvi|miR-940|nat|as|</td><td>gcatatgcatggctcctcat</td><td>vvi-miR160f|*+5_perfect match probe</td></tr>
<tr><td>vvi|miR-940|2mut|as|</td><td>gcatatgaatgcctcctcat</td><td>vvi-miR160f|*+5_mismatch probe</td></tr>
<tr><td>vvi|miR-941|nat|as|</td><td>aaccgctgcatccagtgact</td><td>vvi-miR162|*-5_perfect match probe</td></tr>
<tr><td>vvi|miR-941|2mut|as|</td><td>aacccctgcatcaagtgact</td><td>vvi-miR162|*-5_mismatch probe</td></tr>
<tr><td>vvi|miR-942|nat|as|</td><td>agagatcgatgaaccgctgc</td><td>vvi-miR162|*+5_perfect match probe</td></tr>
<tr><td>vvi|miR-942|2mut|as|</td><td>agagatagatgaacccctgc</td><td>vvi-miR162|*+5_mismatch probe</td></tr>
<tr><td>vvi|miR-943|nat|as|</td><td>aaggggagcacgtgcaagcc</td><td>vvi-miR164a|*-5_perfect match probe</td></tr>
<tr><td>vvi|miR-943|2mut|as|</td><td>aaggtgagcacgtgaaagcc</td><td>vvi-miR164a|*-5_mismatch probe</td></tr>
<tr><td>vvi|miR-944|nat|as|</td><td>catgttggagaaggggagca</td><td>vvi-miR164a|*+5_perfect match probe</td></tr>
<tr><td>vvi|miR-944|2mut|as|</td><td>catgttcgagaaggtgagca</td><td>vvi-miR164a|*+5_mismatch probe</td></tr>
<tr><td>vvi|miR-945|nat|as|</td><td>gagccagggcacatgtaaaa</td><td>vvi-miR164b|*-5_perfect match probe</td></tr>
<tr><td>vvi|miR-945|2mut|as|</td><td>gagccaggccccatgtaaaa</td><td>vvi-miR164b|*-5_mismatch probe</td></tr>
<tr><td>vvi|miR-946|nat|as|</td><td>aagtgggagagccagggcac</td><td>vvi-miR164b|*+5_perfect match probe</td></tr>
<tr><td>vvi|miR-946|2mut|as|</td><td>aagtgtgagagtcagggcac</td><td>vvi-miR164b|*+5_mismatch probe</td></tr>
<tr><td>vvi|miR-947|nat|as|</td><td>aggggagcacgtgcagagca</td><td>vvi-miR164d|*-5_perfect match probe</td></tr>
<tr><td>vvi|miR-947|2mut|as|</td><td>aggggagaacgtgaagagca</td><td>vvi-miR164d|*-5_mismatch probe</td></tr>
<tr><td>vvi|miR-948|nat|as|</td><td>catgttggagaaggggagca</td><td>vvi-miR164d|*+5_perfect match probe</td></tr>
<tr><td>vvi|miR-948|2mut|as|</td><td>catgttcgagaaggtgagca</td><td>vvi-miR164d|*+5_mismatch probe</td></tr>
<tr><td>vvi|miR-949|nat|as|</td><td>cagacaacattcccctcaaa</td><td>vvi-miR166c|*-5_perfect match probe</td></tr>
<tr><td>vvi|miR-949|2mut|as|</td><td>cagacaaaattcctctcaaa</td><td>vvi-miR166c|*-5_mismatch probe</td></tr>
<tr><td>vvi|miR-950|nat|as|</td><td>tgtcctcgagccagacaaca</td><td>vvi-miR166c|*+5_perfect match probe</td></tr>
<tr><td>vvi|miR-950|2mut|as|</td><td>tgtcctcgactcagacaaca</td><td>vvi-miR166c|*+5_mismatch probe</td></tr>
<tr><td>vvi|miR-951|nat|as|</td><td>agacaacaatcccctcaaag</td><td>vvi-miR166d|*-5_perfect match probe</td></tr>
<tr><td>vvi|miR-951|2mut|as|</td><td>agacaaaaatcctctcaaag</td><td>vvi-miR166d|*-5_mismatch probe</td></tr>
</table>

vvi|miR-952|nat|as|	tggcctcgagccagacaaca	vvi-miR166d|*+5_perfect match probe
vvi|miR-952|2mut|as|	tggcctcgactcagacaaca	vvi-miR166d|*+5_mismatch probe
vvi|miR-953|nat|as|	cagacaacattcccctcaaa	vvi-miR166e|*-5_perfect match probe
vvi|miR-953|2mut|as|	cagacaaaattcctctcaaa	vvi-miR166e|*-5_mismatch probe
vvi|miR-954|nat|as|	tgtcctcgagccagacaaca	vvi-miR166e|*+5_perfect match probe
vvi|miR-954|2mut|as|	tgtcctcgactcagacaaca	vvi-miR166e|*+5_mismatch probe
vvi|miR-955|nat|as|	cagccaacattccccacaac	vvi-miR166f|*-5_perfect match probe
vvi|miR-955|2mut|as|	cagcaaacattcctcacaac	vvi-miR166f|*-5_mismatch probe
vvi|miR-956|nat|as|	aagcctcgagccagccaaca	vvi-miR166f|*+5_perfect match probe
vvi|miR-956|2mut|as|	aagcctcgacccagtcaaca	vvi-miR166f|*+5_mismatch probe
vvi|miR-957|nat|as|	agacaacattcccctcaact	vvi-miR166g|*-5_perfect match probe
vvi|miR-957|2mut|as|	agacaaaattcctctcaact	vvi-miR166g|*-5_mismatch probe
vvi|miR-958|nat|as|	ggatctcgaaccagacaaca	vvi-miR166g|*+5_perfect match probe
vvi|miR-958|2mut|as|	ggatctagaacaagacaaca	vvi-miR166g|*+5_mismatch probe
vvi|miR-959|nat|as|	cagacagcgttcccctcaac	vvi-miR166h|*-5_perfect match probe
vvi|miR-959|2mut|as|	cagacagagttcctctcaac	vvi-miR166h|*-5_mismatch probe
vvi|miR-960|nat|as|	tggtctcgaaccagacagcg	vvi-miR166h|*+5_perfect match probe
vvi|miR-960|2mut|as|	tggtctagaacaagacagcg	vvi-miR166h|*+5_mismatch probe
vvi|miR-961|nat|as|	caaacctcattccaaacacc	vvi-miR166a|*-5_perfect match probe
vvi|miR-961|2mut|as|	caaatctcattcaaaacacc	vvi-miR166a|*-5_mismatch probe
vvi|miR-962|nat|as|	ggatcttggatcaaacctca	vvi-miR166a|*+5_perfect match probe
vvi|miR-962|2mut|as|	ggatcttcgatcaaatctca	vvi-miR166a|*+5_mismatch probe
vvi|miR-963|nat|as|	cagccaacattcccctcaaa	vvi-miR166b|*-5_perfect match probe

vvi|miR-963|2mut|as|	cagcaaacattcctctcaaa	vvi-miR166b|*-5_mismatch probe
vvi|miR-964|nat|as|	atgcctcgagccagccaaca	vvi-miR166b|*+5_perfect match probe
vvi|miR-964|2mut|as|	atgcctcgacccagtcaaca	vvi-miR166b|*+5_mismatch probe
vvi|miR-965|nat|as|	ctgcaagatgacctgatcat	vvi-miR167a|*-5_perfect match probe
vvi|miR-965|2mut|as|	ctgcaagataatctgatcat	vvi-miR167a|*-5_mismatch probe
vvi|miR-966|nat|as|	gtgattgaagctgcaagatg	vvi-miR167a|*+5_perfect match probe
vvi|miR-966|2mut|as|	gtgattgaacctgaaagatg	vvi-miR167a|*+5_mismatch probe
vvi|miR-967|nat|as|	ctgccacatgatctagtctt	vvi-miR167b|*-5_perfect match probe
vvi|miR-967|2mut|as|	ctgcaacatgatctactctt	vvi-miR167b|*-5_mismatch probe
vvi|miR-968|nat|as|	acaggtgaaactgccacatg	vvi-miR167b|*+5_perfect match probe
vvi|miR-968|2mut|as|	acagctgaaactgtcacatg	vvi-miR167b|*+5_mismatch probe
vvi|miR-969|nat|as|	gctaccacatgatctgatct	vvi-miR167c|*-5_perfect match probe
vvi|miR-969|2mut|as|	gctacaacatgatccgatct	vvi-miR167c|*-5_mismatch probe
vvi|miR-970|nat|as|	acaggtgaggctaccacatg	vvi-miR167c|*+5_perfect match probe
vvi|miR-970|2mut|as|	acaggtgagcttaccacatg	vvi-miR167c|*+5_mismatch probe
vvi|miR-971|nat|as|	tcagggcatgacctagctag	vvi-miR167d|*-5_perfect match probe
vvi|miR-971|2mut|as|	tcaggccatgacctacctag	vvi-miR167d|*-5_mismatch probe
vvi|miR-972|nat|as|	aggagtgaggctgtcagggc	vvi-miR167d|*+5_perfect match probe
vvi|miR-972|2mut|as|	aggagtgagcttgtcagggc	vvi-miR167d|*+5_mismatch probe
vvi|miR-973|nat|as|	actgccacatgatctgatct	vvi-miR167e|*-5_perfect match probe
vvi|miR-973|2mut|as|	actgtcacatgatccgatct	vvi-miR167e|*-5_mismatch probe
vvi|miR-974|nat|as|	acaggtgaaactgccacatg	vvi-miR167e|*+5_perfect match probe
vvi|miR-974|2mut|as|	acagctgaaactgtcacatg	vvi-miR167e|*+5_mismatch probe

vvi|miR-975|nat|as|	tgatgcaaggcgggatcgga	vvi-miR168|*-5_perfect match probe
vvi|miR-975|2mut|as|	tgatgcaagcagggatcgga	vvi-miR168|*-5_mismatch probe
vvi|miR-976|nat|as|	ccgattcagttgatgcaagg	vvi-miR168|*+5_perfect match probe
vvi|miR-976|2mut|as|	ccgattccgttgatgaaagg	vvi-miR168|*+5_mismatch probe
		vvi-miR169a|*-5_perfect match
vvi|miR-977|nat|as|	aggacaacttgccagggttc	probe
vvi|miR-977|2mut|as|	aggaaaacttgtcagggttc	vvi-miR169a|*-5_mismatch probe
		vvi-miR169a|*+5_perfect match
vvi|miR-978|nat|as|	aatgtagccaaggacaactt	probe
vvi|miR-978|2mut|as|	aatgtagtcaagaacaactt	vvi-miR169a|*+5_mismatch probe
		vvi-miR169y|*-5_perfect match
vvi|miR-979|nat|as|	ccaaggagacttgccataaa	probe
vvi|miR-979|2mut|as|	ccaagaagacttgtcataaa	vvi-miR169y|*-5_mismatch probe
		vvi-miR169y|*+5_perfect match
vvi|miR-980|nat|as|	tcaggtgccaaggagacttg	probe
vvi|miR-980|2mut|as|	tcaggtgtcaagaagacttg	vvi-miR169y|*+5_mismatch probe
		vvi-miR169b|*-5_perfect match
vvi|miR-981|nat|as|	aaggatgactaaccgtgcac	probe
vvi|miR-981|2mut|as|	aaggatgcctaacagtgcac	vvi-miR169b|*-5_mismatch probe
		vvi-miR169b|*+5_perfect match
vvi|miR-982|nat|as|	caaatgagccaaggatgact	probe
vvi|miR-982|2mut|as|	caaatgagtcaagaatgact	vvi-miR169b|*+5_mismatch probe
		vvi-miR169c|*-5_perfect match
vvi|miR-983|nat|as|	aggacaacttgccggattaa	probe
vvi|miR-983|2mut|as|	aggaaaacttgtcggattaa	vvi-miR169c|*-5_mismatch probe
		vvi-miR169c|*+5_perfect match
vvi|miR-984|nat|as|	aatgtagccaaggacaactt	probe
vvi|miR-984|2mut|as|	aatgtagtcaagaacaactt	vvi-miR169c|*+5_mismatch probe
		vvi-miR169d|*-5_perfect match
vvi|miR-985|nat|as|	aggacaacttgccagggttc	probe
vvi|miR-985|2mut|as|	aggaaaacttgtcagggttc	vvi-miR169d|*-5_mismatch probe
		vvi-miR169d|*+5_perfect match
vvi|miR-986|nat|as|	aatgtagccaaggacaactt	probe
vvi|miR-986|2mut|as|	aatgtagtcaagaacaactt	vvi-miR169d|*+5_mismatch probe

vvi|miR-987|nat|as|	caaggtgactgcctggaacc	vvi-miR169e|*-5_perfect match probe
vvi|miR-987|2mut|as|	caagctgactgtctggaacc	vvi-miR169e|*-5_mismatch probe
vvi|miR-988|nat|as|	taaaattagccaaggtgact	vvi-miR169e|*+5_perfect match probe
vvi|miR-988|2mut|as|	taaaattagtaaaggtgact	vvi-miR169e|*+5_mismatch probe
vvi|miR-989|nat|as|	caaacacaacttgcccatta	vvi-miR169f|*-5_perfect match probe
vvi|miR-989|2mut|as|	caaacccaacttgtccatta	vvi-miR169f|*-5_mismatch probe
vvi|miR-990|nat|as|	catgtagccaaacacaactt	vvi-miR169f|*+5_perfect match probe
vvi|miR-990|2mut|as|	catgtagtcaaacccaactt	vvi-miR169f|*+5_mismatch probe
vvi|miR-991|nat|as|	caaagacaacttgccggctt	vvi-miR169g|*-5_perfect match probe
vvi|miR-991|2mut|as|	caaagaaaacttgtcggctt	vvi-miR169g|*-5_mismatch probe
vvi|miR-992|nat|as|	catgtagccaaagacaactt	vvi-miR169g|*+5_perfect match probe
vvi|miR-992|2mut|as|	catgtagtcaaagaaaactt	vvi-miR169g|*+5_mismatch probe
vvi|miR-993|nat|as|	aaggataaccctccgtgcgt	vvi-miR169h|*-5_perfect match probe
vvi|miR-993|2mut|as|	aaggataactctcagtgcgt	vvi-miR169h|*-5_mismatch probe
vvi|miR-994|nat|as|	taaaggagtcaaggataacc	vvi-miR169h|*+5_perfect match probe
vvi|miR-994|2mut|as|	taaagaagtcaagaataacc	vvi-miR169h|*+5_mismatch probe
vvi|miR-995|nat|as|	gggataaccagccgtgcatg	vvi-miR169i|*-5_perfect match probe
vvi|miR-995|2mut|as|	gggataacaagtcgtgcatg	vvi-miR169i|*-5_mismatch probe
vvi|miR-996|nat|as|	taaaggagacagggataacc	vvi-miR169i|*+5_perfect match probe
vvi|miR-996|2mut|as|	taaagaagacagtgataacc	vvi-miR169i|*+5_mismatch probe
vvi|miR-997|nat|as|	aggacaacctgccagggaca	vvi-miR169j|*-5_perfect match probe
vvi|miR-997|2mut|as|	aggacaatctgtcagggaca	vvi-miR169j|*-5_mismatch probe
vvi|miR-998|nat|as|	aaggtagccaaggacaacct	vvi-miR169j|*+5_perfect match probe
vvi|miR-998|2mut|as|	aaggtagtcaagaacaacct	vvi-miR169j|*+5_mismatch probe
vvi|miR-999|nat|as|	aggacaacctgccagggaca	vvi-miR169k|*-5_perfect match

<table>
<tr><td></td><td></td><td>probe</td></tr>
<tr><td>vvi|miR-999|2mut|as|</td><td>aggacaatctgtcagggaca</td><td>vvi-miR169k|*-5_mismatch probe</td></tr>
<tr><td></td><td></td><td>vvi-miR169k|*+5_perfect match</td></tr>
<tr><td>vvi|miR-1000|nat|as|</td><td>aaggtagccaaggacaacct</td><td>probe</td></tr>
<tr><td>vvi|miR-1000|2mut|as|</td><td>aaggtagtcaagaacaacct</td><td>vvi-miR169k|*+5_mismatch probe</td></tr>
<tr><td>vvi|miR-1001|nat|as|</td><td>agaataactagccgtgcatg</td><td>vvi-miR169l|*-5_perfect match probe</td></tr>
<tr><td>vvi|miR-1001|2mut|as|</td><td>agaataattagtcgtgcatg</td><td>vvi-miR169l|*-5_mismatch probe</td></tr>
<tr><td></td><td></td><td>vvi-miR169l|*+5_perfect match</td></tr>
<tr><td>vvi|miR-1002|nat|as|</td><td>caaatgagccaagaataact</td><td>probe</td></tr>
<tr><td>vvi|miR-1002|2mut|as|</td><td>caaatgagtcaacaataact</td><td>vvi-miR169l|*+5_mismatch probe</td></tr>
<tr><td></td><td></td><td>vvi-miR169m|*-5_perfect match</td></tr>
<tr><td>vvi|miR-1003|nat|as|</td><td>tcggatgcttgccagttatg</td><td>probe</td></tr>
<tr><td>vvi|miR-1003|2mut|as|</td><td>tcggatccttgtcagttatg</td><td>vvi-miR169m|*-5_mismatch probe</td></tr>
<tr><td></td><td></td><td>vvi-miR169m|*+5_perfect match</td></tr>
<tr><td>vvi|miR-1004|nat|as|</td><td>aaacagagcctcggatgctt</td><td>probe</td></tr>
<tr><td>vvi|miR-1004|2mut|as|</td><td>aaacagagtctcagatgctt</td><td>vvi-miR169m|*+5_mismatch probe</td></tr>
<tr><td></td><td></td><td>vvi-miR169n|*-5_perfect match</td></tr>
<tr><td>vvi|miR-1005|nat|as|</td><td>tgcttgccagttatgaccag</td><td>probe</td></tr>
<tr><td>vvi|miR-1005|2mut|as|</td><td>tgcttgtcagttatgcccag</td><td>vvi-miR169n|*-5_mismatch probe</td></tr>
<tr><td></td><td></td><td>vvi-miR169n|*+5_perfect match</td></tr>
<tr><td>vvi|miR-1006|nat|as|</td><td>aaatagagcctcagatgctt</td><td>probe</td></tr>
<tr><td>vvi|miR-1006|2mut|as|</td><td>aaatagagtctccgatgctt</td><td>vvi-miR169n|*+5_mismatch probe</td></tr>
<tr><td></td><td></td><td>vvi-miR169o|*-5_perfect match</td></tr>
<tr><td>vvi|miR-1007|nat|as|</td><td>gaataactcgctgtccatgc</td><td>probe</td></tr>
<tr><td>vvi|miR-1007|2mut|as|</td><td>gaataactccttgtccatgc</td><td>vvi-miR169o|*-5_mismatch probe</td></tr>
<tr><td></td><td></td><td>vvi-miR169o|*+5_perfect match</td></tr>
<tr><td>vvi|miR-1008|nat|as|</td><td>cgcatgagccaagaataact</td><td>probe</td></tr>
<tr><td>vvi|miR-1008|2mut|as|</td><td>cgcatgagtcaacaataact</td><td>vvi-miR169o|*+5_mismatch probe</td></tr>
<tr><td></td><td></td><td>vvi-miR169p|*-5_perfect match</td></tr>
<tr><td>vvi|miR-1009|nat|as|</td><td>tcggatgcttgccagttata</td><td>probe</td></tr>
<tr><td>vvi|miR-1009|2mut|as|</td><td>tcggatccttgtcagttata</td><td>vvi-miR169p|*-5_mismatch probe</td></tr>
<tr><td></td><td></td><td>vvi-miR169p|*+5_perfect match</td></tr>
<tr><td>vvi|miR-1010|nat|as|</td><td>aaacagagcctcggatgctt</td><td>probe</td></tr>
<tr><td>vvi|miR-1010|2mut|as|</td><td>aaacagagtctcagatgctt</td><td>vvi-miR169p|*+5_mismatch probe</td></tr>
</table>

vvi|miR-1011|nat|as|	aggattacatgccggctgtt	vvi-miR169q|*-5_perfect match probe
vvi|miR-1011|2mut|as|	aggattaaatgccgcctgtt	vvi-miR169q|*-5_mismatch probe
vvi|miR-1012|nat|as|	aaatagagccaggattacat	vvi-miR169q|*+5_perfect match probe
vvi|miR-1012|2mut|as|	aaatagagtcagaattacat	vvi-miR169q|*+5_mismatch probe
vvi|miR-1013|nat|as|	caagtcaacttgccggttac	vvi-miR169r|*-5_perfect match probe
vvi|miR-1013|2mut|as|	caagccaacttgtcggttac	vvi-miR169r|*-5_mismatch probe
vvi|miR-1014|nat|as|	caaactgagtcaagtcaact	vvi-miR169r|*+5_perfect match probe
vvi|miR-1014|2mut|as|	caaactgagccaagccaact	vvi-miR169r|*+5_mismatch probe
vvi|miR-1015|nat|as|	ggaacaacttgccgggaaga	vvi-miR169s|*-5_perfect match probe
vvi|miR-1015|2mut|as|	ggaaaaacttgtcgggaaga	vvi-miR169s|*-5_mismatch probe
vvi|miR-1016|nat|as|	aatgtagccaggaacaactt	vvi-miR169s|*+5_perfect match probe
vvi|miR-1016|2mut|as|	aatgtagtcagaaacaactt	vvi-miR169s|*+5_mismatch probe
vvi|miR-1017|nat|as|	caagtcaacttgccggttac	vvi-miR169t|*-5_perfect match probe
vvi|miR-1017|2mut|as|	caagccaacttgtcggttac	vvi-miR169t|*-5_mismatch probe
vvi|miR-1018|nat|as|	aaactgagtcaagtcaactt	vvi-miR169t|*+5_perfect match probe
vvi|miR-1018|2mut|as|	aaactgagccaagccaactt	vvi-miR169t|*+5_mismatch probe
vvi|miR-1019|nat|as|	caagtcaacttgccggtaac	vvi-miR169u|*-5_perfect match probe
vvi|miR-1019|2mut|as|	caagccaacttgtcggtaac	vvi-miR169u|*-5_mismatch probe
vvi|miR-1020|nat|as|	aaacagagtcaagtcaactt	vvi-miR169u|*+5_perfect match probe
vvi|miR-1020|2mut|as|	aaacagagccaagccaactt	vvi-miR169u|*+5_mismatch probe
vvi|miR-1021|nat|as|	gaaataaattgccgatccat	vvi-miR169v|*-5_perfect match probe
vvi|miR-1021|2mut|as|	gaaataaatcgtcgatccat	vvi-miR169v|*-5_mismatch probe
vvi|miR-1022|nat|as|	aacatagccaagaaataaat	vvi-miR169v|*+5_perfect match probe
vvi|miR-1022|2mut|as|	aacatagtcaacaaataaat	vvi-miR169v|*+5_mismatch probe
vvi|miR-1023|nat|as|	agaatgactcgccgaccacc	vvi-miR169w|*-5_perfect match

		probe
vvi|miR-1023|2mut|as|	agaatgactacccgaccacc	vvi-miR169w|*-5_mismatch probe
		vvi-miR169w|*+5_perfect match
vvi|miR-1024|nat|as|	aatgtagctaagaatgactc	probe
vvi|miR-1024|2mut|as|	aatgtagttaagaataactc	vvi-miR169w|*+5_mismatch probe
		vvi-miR169x|*-5_perfect match
vvi|miR-1025|nat|as|	aggaatgactgcctaaagac	probe
vvi|miR-1025|2mut|as|	aggaatgcctgtctaaagac	vvi-miR169x|*-5_mismatch probe
		vvi-miR169x|*+5_perfect match
vvi|miR-1026|nat|as|	cagtttagccgaaggaatga	probe
vvi|miR-1026|2mut|as|	cagtttagtcaaaggaatga	vvi-miR169x|*+5_mismatch probe
		vvi-miR171a|*-5_perfect match
vvi|miR-1027|nat|as|	cgtgccaatatcatgttcaa	probe
vvi|miR-1027|2mut|as|	cgtgtcaatatcatcttcaa	vvi-miR171a|*-5_mismatch probe
		vvi-miR171a|*+5_perfect match
vvi|miR-1028|nat|as|	tctgattgagccgtgccaat	probe
vvi|miR-1028|2mut|as|	tctgattgacccgtgtcaat	vvi-miR171a|*+5_mismatch probe
		vvi-miR171b|*-5_perfect match
vvi|miR-1029|nat|as|	aggcacgccaatacctcccc	probe
vvi|miR-1029|2mut|as|	aggcaccccaatatctcccc	vvi-miR171b|*-5_mismatch probe
		vvi-miR171b|*+5_perfect match
vvi|miR-1030|nat|as|	ctttaaattgaggcacgcca	probe
vvi|miR-1030|2mut|as|	ctttaaattaagccacgcca	vvi-miR171b|*+5_mismatch probe
		vvi-miR171c|*-5_perfect match
vvi|miR-1031|nat|as|	cgcaccaatatcccgtaata	probe
vvi|miR-1031|2mut|as|	cgcacaaatatccagtaata	vvi-miR171c|*-5_mismatch probe
		vvi-miR171c|*+5_perfect match
vvi|miR-1032|nat|as|	tcttattgaaccgcaccaat	probe
vvi|miR-1032|2mut|as|	tcttattaaaccccaccaat	vvi-miR171c|*+5_mismatch probe
		vvi-miR171d|*-5_perfect match
vvi|miR-1033|nat|as|	cgtatcaatatctcgtgtat	probe
vvi|miR-1033|2mut|as|	cgtataaatatctcctgtat	vvi-miR171d|*-5_mismatch probe
		vvi-miR171d|*+5_perfect match
vvi|miR-1034|nat|as|	tctaattgaaccgtatcaat	probe

vvi|miR-1034|2mut|as|	tctaattaaacagtatcaat	vvi-miR171d|*+5_mismatch probe
		vvi-miR171e|*-5_perfect match
vvi|miR-1035|nat|as|	aacctcaccaacatcgctta	probe
vvi|miR-1035|2mut|as|	aacctcacaaacatccctta	vvi-miR171e|*-5_mismatch probe
		vvi-miR171e|*+5_perfect match
vvi|miR-1036|nat|as|	cttcggattgaacctcacca	probe
vvi|miR-1036|2mut|as|	cttcagattgaatctcacca	vvi-miR171e|*+5_mismatch probe
vvi|miR-1037|nat|as|	ctcaccaacatcgctttctt	vvi-miR171f|*-5_perfect match probe
vvi|miR-1037|2mut|as|	ctcacaaacatccctttctt	vvi-miR171f|*-5_mismatch probe
		vvi-miR171f|*+5_perfect match
vvi|miR-1038|nat|as|	tcagattgaacctcaccaac	probe
vvi|miR-1038|2mut|as|	tcagattaaacctcatcaac	vvi-miR171f|*+5_mismatch probe
		vvi-miR171g|*-5_perfect match
vvi|miR-1039|nat|as|	cgatggaaccaacatggaga	probe
vvi|miR-1039|2mut|as|	cgatggaacaaacatcgaga	vvi-miR171g|*-5_mismatch probe
		vvi-miR171g|*+5_perfect match
vvi|miR-1040|nat|as|	ccccaccgatggaaccaaca	probe
vvi|miR-1040|2mut|as|	ccccacagatggaacaaaca	vvi-miR171g|*+5_mismatch probe
		vvi-miR171h|*-5_perfect match
vvi|miR-1041|nat|as|	aaccgcaccaacatctcgtg	probe
vvi|miR-1041|2mut|as|	aaccccaccaaaatctcgtg	vvi-miR171h|*-5_mismatch probe
		vvi-miR171h|*+5_perfect match
vvi|miR-1042|nat|as|	agttaggttgaaccgcacca	probe
vvi|miR-1042|2mut|as|	agttagcttgaaccccacca	vvi-miR171h|*+5_mismatch probe
vvi|miR-1043|nat|as|	cattccaacatccccacctt	vvi-miR171i|*-5_perfect match probe
vvi|miR-1043|2mut|as|	cattcaaacatcctcacctt	vvi-miR171i|*-5_mismatch probe
		vvi-miR171i|*+5_perfect match
vvi|miR-1044|nat|as|	tttgattgagccattccaac	probe
vvi|miR-1044|2mut|as|	tttgattgactcattccaac	vvi-miR171i|*+5_mismatch probe
		vvi-miR172a|*-5_perfect match
vvi|miR-1045|nat|as|	ttgatgatgctgcatcggca	probe
vvi|miR-1045|2mut|as|	ttgatgatcctgaatcggca	vvi-miR172a|*-5_mismatch probe
		vvi-miR172a|*+5_perfect match
vvi|miR-1046|nat|as|	gatgagaatcttgatgatgc	probe

vvi|miR-1046|2mut|as|	gatgcgaatcttgataatgc
vvi|miR-1047|nat|as|	cttgatgatgctgcatcggc
vvi|miR-1047|2mut|as|	cttgatgatcctgaatcggc
vvi|miR-1048|nat|as|	gttgagaatcttgatgatgc
vvi|miR-1048|2mut|as|	gttgcgaatcttgataatgc
vvi|miR-1049|nat|as|	cttgatgatgctccatccgc
vvi|miR-1049|2mut|as|	cttgatgatcttccatccgc
vvi|miR-1050|nat|as|	cttgtgaatcttgatgatgc
vvi|miR-1050|2mut|as|	cttgcgaatcttgataatgc
vvi|miR-1051|nat|as|	ttgatgatgctgcatcagct
vvi|miR-1051|2mut|as|	ttgatgatcctgaatcagct
vvi|miR-1052|nat|as|	ggtgtgaatcttgatgatgc
vvi|miR-1052|2mut|as|	ggtgcgaatcttgataatgc
vvi|miR-1053|nat|as|	actaaagagagctccttctt
vvi|miR-1053|2mut|as|	actaaagagcgttccttctt
vvi|miR-1054|nat|as|	tcggactggactaaagagag
vvi|miR-1054|2mut|as|	tcggactcgattaaagagag
vvi|miR-1055|nat|as|	ctgaagaaagctctcttcaa
vvi|miR-1055|2mut|as|	ctgaagaaacttctcttcaa
vvi|miR-1056|nat|as|	atgagtggactgaagaaagc
vvi|miR-1056|2mut|as|	atgagtcgaccgaagaaagc
vvi|miR-1057|nat|as|	tgcaaaggaacccccatttc
vvi|miR-1057|2mut|as|	tgcaaagaaaccctcatttc

vvi-miR172a|*+5_mismatch probe
vvi-miR172b|*-5_perfect match probe
vvi-miR172b|*-5_mismatch probe
vvi-miR172b|*+5_perfect match probe
vvi-miR172b|*+5_mismatch probe
vvi-miR172c|*-5_perfect match probe
vvi-miR172c|*-5_mismatch probe
vvi-miR172c|*+5_perfect match probe
vvi-miR172c|*+5_mismatch probe
vvi-miR172d|*-5_perfect match probe
vvi-miR172d|*-5_mismatch probe
vvi-miR172d|*+5_perfect match probe
vvi-miR172d|*+5_mismatch probe
vvi-miR319b|*-5_perfect match probe
vvi-miR319b|*-5_mismatch probe
vvi-miR319b|*+5_perfect match probe
vvi-miR319b|*+5_mismatch probe
vvi-miR319c|*-5_perfect match probe
vvi-miR319c|*-5_mismatch probe
vvi-miR319c|*+5_perfect match probe
vvi-miR319c|*+5_mismatch probe
vvi-miR319e|*-5_perfect match probe
vvi-miR319e|*-5_mismatch probe

vvi|miR-1058|nat|as|	gttgttttgggctgcaaagg	vvi-miR319e|*+5_perfect match probe
vvi|miR-1058|2mut|as|	gttgttttgtcctgcaaagg	vvi-miR319e|*+5_mismatch probe
vvi|miR-1059|nat|as|	ctgaaggaagctctcttaaa	vvi-miR319f|*-5_perfect match probe
vvi|miR-1059|2mut|as|	ctgaaggaacttctcttaaa	vvi-miR319f|*-5_mismatch probe
vvi|miR-1060|nat|as|	atgagtggactgaaggaagc	vvi-miR319f|*+5_perfect match probe
vvi|miR-1060|2mut|as|	atgagtcgactgaagaaagc	vvi-miR319f|*+5_mismatch probe
vvi|miR-1061|nat|as|	gactaaaaggagctcttcag	vvi-miR319g|*-5_perfect match probe
vvi|miR-1061|2mut|as|	gactaaaagaagttcttcag	vvi-miR319g|*-5_mismatch probe
vvi|miR-1062|nat|as|	tctttattggactaaaagga	vvi-miR319g|*+5_perfect match probe
vvi|miR-1062|2mut|as|	tctttattcgcctaaaagga	vvi-miR319g|*+5_mismatch probe
vvi|miR-1063|nat|as|	aggatagatagcgccacaca	vvi-miR390|*-5_perfect match probe
vvi|miR-1063|2mut|as|	aggatacatagccccacaca	vvi-miR390|*-5_mismatch probe
vvi|miR-1064|nat|as|	ccgtgaaactcaggatagat	vvi-miR390|*+5_perfect match probe
vvi|miR-1064|2mut|as|	ccgtaaaactcagaatagat	vvi-miR390|*+5_mismatch probe
vvi|miR-1065|nat|as|	gggatagcatgatccaatgg	vvi-miR393a|*-5_perfect match probe
vvi|miR-1065|2mut|as|	gggatagaatgatcaaatgg	vvi-miR393a|*-5_mismatch probe
vvi|miR-1066|nat|as|	agagttcctaagggatagca	vvi-miR393a|*+5_perfect match probe
vvi|miR-1066|2mut|as|	agagtttctaagtgatagca	vvi-miR393a|*+5_mismatch probe
vvi|miR-1067|nat|as|	gggatagcatgatccaaaga	vvi-miR393b|*-5_perfect match probe
vvi|miR-1067|2mut|as|	gggatagaatgatcaaaaga	vvi-miR393b|*-5_mismatch probe
vvi|miR-1068|nat|as|	aggaatccaaagggatagca	vvi-miR393b|*+5_perfect match probe
vvi|miR-1068|2mut|as|	aggaatcaaaagtgatagca	vvi-miR393b|*+5_mismatch probe
vvi|miR-1069|nat|as|	atgctggccacctccaaaag	vvi-miR394a|*-5_perfect match probe
vvi|miR-1069|2mut|as|	atgctggtcacctcaaaaag	vvi-miR394a|*-5_mismatch probe
vvi|miR-1070|nat|as|	ccactttggcatgctggcca	vvi-miR394a|*+5_perfect match

		probe
vvi|miR-1070|2mut|as|	ccactttgccatcctggcca	vvi-miR394a|*+5_mismatch probe
		vvi-miR394b|*-5_perfect match
vvi|miR-1071|nat|as|	agtatgcccacctccacaga	probe
vvi|miR-1071|2mut|as|	agtatgtccacctcaacaga	vvi-miR394b|*-5_mismatch probe
		vvi-miR394b|*+5_perfect match
vvi|miR-1072|nat|as|	cttggttggcagtatgccca	probe
vvi|miR-1072|2mut|as|	cttggttgccagtatcccca	vvi-miR394b|*+5_mismatch probe
		vvi-miR394c|*-5_perfect match
vvi|miR-1073|nat|as|	atcctggccgcctccaaacg	probe
vvi|miR-1073|2mut|as|	atcctggccctctccaaacg	vvi-miR394c|*-5_mismatch probe
		vvi-miR394c|*+5_perfect match
vvi|miR-1074|nat|as|	ccaatttggcatcctggccg	probe
vvi|miR-1074|2mut|as|	ccaatttgccatcctcgccg	vvi-miR394c|*+5_mismatch probe
		vvi-miR395a|*-5_perfect match
vvi|miR-1075|nat|as|	tgatcaagggaactctaggg	probe
vvi|miR-1075|2mut|as|	tgatcaagtgaattctaggg	vvi-miR395a|*-5_mismatch probe
		vvi-miR395a|*+5_perfect match
vvi|miR-1076|nat|as|	cctagtgaagtgatcaaggg	probe
vvi|miR-1076|2mut|as|	cctagcgaagcgatcaaggg	vvi-miR395a|*+5_mismatch probe
		vvi-miR395b|*-5_perfect match
vvi|miR-1077|nat|as|	tggtaaggggaactctaggg	probe
vvi|miR-1077|2mut|as|	tggtaaggtgaattctaggg	vvi-miR395b|*-5_mismatch probe
		vvi-miR395b|*+5_perfect match
vvi|miR-1078|nat|as|	cccagtgaagtggtaagggg	probe
vvi|miR-1078|2mut|as|	cccagcgaagtcgtaagggg	vvi-miR395b|*+5_mismatch probe
		vvi-miR395c|*-5_perfect match
vvi|miR-1079|nat|as|	tggtcaagggaactctaggg	probe
vvi|miR-1079|2mut|as|	tggtcaagtgaattctaggg	vvi-miR395c|*-5_mismatch probe
		vvi-miR395c|*+5_perfect match
vvi|miR-1080|nat|as|	cccagtgaagtggtcaaggg	probe
vvi|miR-1080|2mut|as|	cccagcgaagtcgtcaaggg	vvi-miR395c|*+5_mismatch probe
		vvi-miR395d|*-5_perfect match
vvi|miR-1081|nat|as|	tggtcaggggaactctaggg	probe

vvi|miR-1081|2mut|as|	tggtcaggtgaattctaggg	vvi-miR395d|*-5_mismatch probe
		vvi-miR395d|*+5_perfect match
vvi|miR-1082|nat|as|	cccaatgaagtggtcagggg	probe
vvi|miR-1082|2mut|as|	cccaataaagtcgtcagggg	vvi-miR395d|*+5_mismatch probe
		vvi-miR395e|*-5_perfect match
vvi|miR-1083|nat|as|	tggtcaagggaactctaggg	probe
vvi|miR-1083|2mut|as|	tggtcaagtgaattctaggg	vvi-miR395e|*-5_mismatch probe
		vvi-miR395e|*+5_perfect match
vvi|miR-1084|nat|as|	cccagtgaagtggtcaaggg	probe
vvi|miR-1084|2mut|as|	cccagcgaagtcgtcaaggg	vvi-miR395e|*+5_mismatch probe
vvi|miR-1085|nat|as|	tggtcaggggaactctaggg	vvi-miR395f|*-5_perfect match probe
vvi|miR-1085|2mut|as|	tggtcaggtgaattctaggg	vvi-miR395f|*-5_mismatch probe
		vvi-miR395f|*+5_perfect match
vvi|miR-1086|nat|as|	cccagtgaagtggtcagggg	probe
vvi|miR-1086|2mut|as|	cccagcgaagtcgtcagggg	vvi-miR395f|*+5_mismatch probe
		vvi-miR395g|*-5_perfect match
vvi|miR-1087|nat|as|	tgctcaggggaactctaggg	probe
vvi|miR-1087|2mut|as|	tgctcaggtgaattctaggg	vvi-miR395g|*-5_mismatch probe
		vvi-miR395g|*+5_perfect match
vvi|miR-1088|nat|as|	cccaatgaagtgctcagggg	probe
vvi|miR-1088|2mut|as|	cccaataaagtcctcagggg	vvi-miR395g|*+5_mismatch probe
		vvi-miR395h|*-5_perfect match
vvi|miR-1089|nat|as|	tggtcaagggaactctaggg	probe
vvi|miR-1089|2mut|as|	tggtcaagtgaattctaggg	vvi-miR395h|*-5_mismatch probe
		vvi-miR395h|*+5_perfect match
vvi|miR-1090|nat|as|	cccagtgaagtggtcaaggg	probe
vvi|miR-1090|2mut|as|	cccagcgaagtcgtcaaggg	vvi-miR395h|*+5_mismatch probe
vvi|miR-1091|nat|as|	tggtcaggggaactctaggg	vvi-miR395i|*-5_perfect match probe
vvi|miR-1091|2mut|as|	tggtcaggtgaattctaggg	vvi-miR395i|*-5_mismatch probe
		vvi-miR395i|*+5_perfect match
vvi|miR-1092|nat|as|	cccagtgaagtggtcagggg	probe
vvi|miR-1092|2mut|as|	cccagcgaagtcgtcagggg	vvi-miR395i|*+5_mismatch probe
vvi|miR-1093|nat|as|	tggtcaggggaactctaggg	vvi-miR395j|*-5_perfect match probe
vvi|miR-1093|2mut|as|	tggtcaggtgaattctaggg	vvi-miR395j|*-5_mismatch probe

vvi|miR-1094|nat|as|	cccagtgaagtggtcagggg	vvi-miR395j|*+5_perfect match probe
vvi|miR-1094|2mut|as|	cccagcgaagtcgtcagggg	vvi-miR395j|*+5_mismatch probe
vvi|miR-1095|nat|as|	tggtcaagggaactctaggg	vvi-miR395k|*-5_perfect match probe
vvi|miR-1095|2mut|as|	tggtcaagtgaattctaggg	vvi-miR395k|*-5_mismatch probe
vvi|miR-1096|nat|as|	cccagtgaagtggtcaaggg	vvi-miR395k|*+5_perfect match probe
vvi|miR-1096|2mut|as|	cccagcgaagtcgtcaaggg	vvi-miR395k|*+5_mismatch probe
vvi|miR-1097|nat|as|	tggtcaggggaactctaggg	vvi-miR395l|*-5_perfect match probe
vvi|miR-1097|2mut|as|	tggtcaggtgaattctaggg	vvi-miR395l|*-5_mismatch probe
vvi|miR-1098|nat|as|	cccagtgaagtggtcagggg	vvi-miR395l|*+5_perfect match probe
vvi|miR-1098|2mut|as|	cccagcgaagtcgtcagggg	vvi-miR395l|*+5_mismatch probe
vvi|miR-1099|nat|as|	tgttcaagggaactctaggg	vvi-miR395m|*-5_perfect match probe
vvi|miR-1099|2mut|as|	tgttcaagtgaattctaggg	vvi-miR395m|*-5_mismatch probe
vvi|miR-1100|nat|as|	cccagtgaagtgttcaaggg	vvi-miR395m|*+5_perfect match probe
vvi|miR-1100|2mut|as|	cccagcgaagcgttcaaggg	vvi-miR395m|*+5_mismatch probe
vvi|miR-1101|nat|as|	gggttgggggaactctcaag	vvi-miR395n|*-5_perfect match probe
vvi|miR-1101|2mut|as|	gggttgggtgaattctcaag	vvi-miR395n|*-5_mismatch probe
vvi|miR-1102|nat|as|	tcatactggaggggttgggg	vvi-miR395n|*+5_perfect match probe
vvi|miR-1102|2mut|as|	tcatactcgaggtgttgggg	vvi-miR395n|*+5_mismatch probe
vvi|miR-1103|nat|as|	cagctttcttgaactacttt	vvi-miR396a|*-5_perfect match probe
vvi|miR-1103|2mut|as|	cagctttttaaactacttt	vvi-miR396a|*-5_mismatch probe
vvi|miR-1104|nat|as|	cttttccacagctttcttga	vvi-miR396a|*+5_perfect match probe
vvi|miR-1104|2mut|as|	cttttcaacagttttcttga	vvi-miR396a|*+5_mismatch probe
vvi|miR-1105|nat|as|	agctttcttgaacttcttct	vvi-miR396b|*-5_perfect match probe

vvi|miR-1105|2mut|as| agctttcttaaatttcttct vvi-miR396b|*-5_mismatch probe

vvi|miR-1106|nat|as| atgcttttccacagctttct vvi-miR396b|*+5_perfect match probe

vvi|miR-1106|2mut|as| atgcttttcaacagttttct vvi-miR396b|*+5_mismatch probe

vvi|miR-1107|nat|as| aactttcctgagctgttggt vvi-miR396c|*-5_perfect match probe

vvi|miR-1107|2mut|as| aacttttctgagttgttggt vvi-miR396c|*-5_mismatch probe

vvi|miR-1108|nat|as| taatgttccacaactttcct vvi-miR396c|*+5_perfect match probe

vvi|miR-1108|2mut|as| taatgttcaaaaactttcct vvi-miR396c|*+5_mismatch probe

vvi|miR-1109|nat|as| agctttattgaaccgcaact vvi-miR396d|*-5_perfect match probe

vvi|miR-1109|2mut|as| agctttattaaaccccaact vvi-miR396d|*-5_mismatch probe

vvi|miR-1110|nat|as| tatcttcccacagctttatt vvi-miR396d|*+5_perfect match probe

vvi|miR-1110|2mut|as| tatcttctcacagttttatt vvi-miR396d|*+5_mismatch probe

vvi|miR-1111|nat|as| agtgcagcgccaatggaatc vvi-miR397a|*-5_perfect match probe

vvi|miR-1111|2mut|as| agtgcagccccaatcgaatc vvi-miR397a|*-5_mismatch probe

vvi|miR-1112|nat|as| gacatgattgagtgcagcg vvi-miR397a|*+5_perfect match probe

vvi|miR-1112|2mut|as| gacatgattaagtgaagcg vvi-miR397a|*+5_mismatch probe

vvi|miR-1113|nat|as| agtgcagcgccaatggaatc vvi-miR397b|*-5_perfect match probe

vvi|miR-1113|2mut|as| agtgcagccccaatcgaatc vvi-miR397b|*-5_mismatch probe

vvi|miR-1114|nat|as| gacatgattgagtgcagcg vvi-miR397b|*+5_perfect match probe

vvi|miR-1114|2mut|as| gacatgattaagtgaagcg vvi-miR397b|*+5_mismatch probe

vvi|miR-1115|nat|as| caggtgccactcccttgggg vvi-miR398a|*-5_perfect match probe

vvi|miR-1115|2mut|as| caggtgtcactctcttgggg vvi-miR398a|*-5_mismatch probe

vvi|miR-1116|nat|as| aaccggtgttctcaggtgcc vvi-miR398a|*+5_perfect match probe

vvi|miR-1116|2mut|as| aaccagtgttctcagctgcc vvi-miR398a|*+5_mismatch probe

vvi|miR-1117|nat|as| ctcaggtcacacctgtagga vvi-miR398b|*-5_perfect match probe
vvi|miR-1117|2mut|as| ctcagctcacatctgtagga vvi-miR398b|*-5_mismatch probe

vvi|miR-1118|nat|as| gggcatgtgattctcaggtc vvi-miR398b|*+5_perfect match probe
vvi|miR-1118|2mut|as| gggcatgcgattctccggtc vvi-miR398b|*+5_mismatch probe

vvi|miR-1119|nat|as| ctcaggtcactcctgtagga vvi-miR398c|*-5_perfect match probe
vvi|miR-1119|2mut|as| ctcagctcacttctgtagga vvi-miR398c|*-5_mismatch probe

vvi|miR-1120|nat|as| gggcatgtgattctcaggtc vvi-miR398c|*+5_perfect match probe
vvi|miR-1120|2mut|as| gggcatgcgattctccggtc vvi-miR398c|*+5_mismatch probe

vvi|miR-1121|nat|as| aggagaatcacactgttatt vvi-miR399a|*-5_perfect match probe
vvi|miR-1121|2mut|as| aggagaatccccctgttatt vvi-miR399a|*-5_mismatch probe

vvi|miR-1122|nat|as| atctctgccaaaggagaatc vvi-miR399a|*+5_perfect match probe
vvi|miR-1122|2mut|as| atctctgtcaaagaagaatc vvi-miR399a|*+5_mismatch probe

vvi|miR-1123|nat|as| gaaagaggtgccctatgatt vvi-miR399b|*-5_perfect match probe
vvi|miR-1123|2mut|as| gaaagaggtctcctatgatt vvi-miR399b|*-5_mismatch probe

vvi|miR-1124|nat|as| gtgcctgccaagaaagaggt vvi-miR399b|*+5_perfect match probe
vvi|miR-1124|2mut|as| gtgcctgtcaagaaacaggt vvi-miR399b|*+5_mismatch probe

vvi|miR-1125|nat|as| gtgaaagaggccctgagacc vvi-miR399c|*-5_perfect match probe
vvi|miR-1125|2mut|as| gtgaaagagctcctgagacc vvi-miR399c|*-5_mismatch probe

vvi|miR-1126|nat|as| tcacctaccaagtgaaagag vvi-miR399c|*+5_perfect match probe
vvi|miR-1126|2mut|as| tcacctacaaagcgaaagag vvi-miR399c|*+5_mismatch probe

vvi|miR-1127|nat|as| aaaagaaatctgctctataa vvi-miR399d|*-5_perfect match probe
vvi|miR-1127|2mut|as| aaaagaaatttcctctataa vvi-miR399d|*-5_mismatch probe
vvi|miR-1128|nat|as| ccatctgccaaaagaaatct vvi-miR399d|*+5_perfect match

		probe
vvi|miR-1128|2mut|as|	ccatctgtcaaaacaaatct	vvi-miR399d|*+5_mismatch probe
		vvi-miR399e|*-5_perfect match
vvi|miR-1129|nat|as|	aaagataatttgccctgtaa	probe
vvi|miR-1129|2mut|as|	aaagctaatttgtcctgtaa	vvi-miR399e|*-5_mismatch probe
		vvi-miR399e|*+5_perfect match
vvi|miR-1130|nat|as|	ctgcctgccaaaagataatt	probe
vvi|miR-1130|2mut|as|	ctgcctgtcaaaacataatt	vvi-miR399e|*+5_mismatch probe
vvi|miR-1131|nat|as|	tttggccctctaatgcaac	vvi-miR399f|*-5_perfect match probe
vvi|miR-1131|2mut|as|	tttggtcctctaatccaac	vvi-miR399f|*-5_mismatch probe
		vvi-miR399f|*+5_perfect match
vvi|miR-1132|nat|as|	cttcatgccaaagcagattt	probe
vvi|miR-1132|2mut|as|	cttcatgtcaaagaagattt	vvi-miR399f|*+5_mismatch probe
		vvi-miR399g|*-5_perfect match
vvi|miR-1133|nat|as|	aatggagtattgcccagcaa	probe
vvi|miR-1133|2mut|as|	aatgaagtattgtccagcaa	vvi-miR399g|*-5_mismatch probe
		vvi-miR399g|*+5_perfect match
vvi|miR-1134|nat|as|	ccaactgccaatggagtatt	probe
vvi|miR-1134|2mut|as|	ccaactgtcaatcgagtatt	vvi-miR399g|*+5_mismatch probe
		vvi-miR399h|*-5_perfect match
vvi|miR-1135|nat|as|	aggaggattgcactgttatt	probe
vvi|miR-1135|2mut|as|	aggaggattcaactgttatt	vvi-miR399h|*-5_mismatch probe
		vvi-miR399h|*+5_perfect match
vvi|miR-1136|nat|as|	ctttctgccaaaggaggatt	probe
vvi|miR-1136|2mut|as|	ctttctgtcaaagaaggatt	vvi-miR399h|*+5_mismatch probe
vvi|miR-1137|nat|as|	aggagagaagccctacaact	vvi-miR399i|*-5_perfect match probe
vvi|miR-1137|2mut|as|	aggagagaactcctacaact	vvi-miR399i|*-5_mismatch probe
		vvi-miR399i|*+5_perfect match
vvi|miR-1138|nat|as|	ctcctgccagaaggagagaa	probe
vvi|miR-1138|2mut|as|	ctcctgtcagaagaagagaa	vvi-miR399i|*+5_mismatch probe
		vvi-miR403a|*-5_perfect match
vvi|miR-1139|nat|as|	attcacgcacaaacttgaga	probe
vvi|miR-1139|2mut|as|	attcacccacaaatttgaga	vvi-miR403a|*-5_mismatch probe
vvi|miR-1140|nat|as|	tggggtttgattcacgcaca	vvi-miR403a|*+5_perfect match

<table>
<tr><td></td><td></td><td>probe</td></tr>
<tr><td>vvi|miR-1140|2mut|as|</td><td>tgggctttgattcacccaca</td><td>vvi-miR403a|*+5_mismatch probe</td></tr>
<tr><td></td><td></td><td>vvi-miR403b|*-5_perfect match</td></tr>
<tr><td>vvi|miR-1141|nat|as|</td><td>tcgcgcacaaactcgaggtt</td><td>probe</td></tr>
<tr><td>vvi|miR-1141|2mut|as|</td><td>tcgcccacaaactagaggtt</td><td>vvi-miR403b|*-5_mismatch probe</td></tr>
<tr><td></td><td></td><td>vvi-miR403b|*+5_perfect match</td></tr>
<tr><td>vvi|miR-1142|nat|as|</td><td>aggcgttggattcgcgcaca</td><td>probe</td></tr>
<tr><td>vvi|miR-1142|2mut|as|</td><td>aggccttggattcgcccaca</td><td>vvi-miR403b|*+5_mismatch probe</td></tr>
<tr><td></td><td></td><td>vvi-miR403c|*-5_perfect match</td></tr>
<tr><td>vvi|miR-1143|nat|as|</td><td>tcacgcacaaacttgagatg</td><td>probe</td></tr>
<tr><td>vvi|miR-1143|2mut|as|</td><td>tcacccacaaacttaagatg</td><td>vvi-miR403c|*-5_mismatch probe</td></tr>
<tr><td></td><td></td><td>vvi-miR403c|*+5_perfect match</td></tr>
<tr><td>vvi|miR-1144|nat|as|</td><td>tggggtttgattcacgcaca</td><td>probe</td></tr>
<tr><td>vvi|miR-1144|2mut|as|</td><td>tgggctttgattcacccaca</td><td>vvi-miR403c|*+5_mismatch probe</td></tr>
<tr><td></td><td></td><td>vvi-miR403d|*-5_perfect match</td></tr>
<tr><td>vvi|miR-1145|nat|as|</td><td>tcgcgcacaaactcgaggtt</td><td>probe</td></tr>
<tr><td>vvi|miR-1145|2mut|as|</td><td>tcgcccacaaactagaggtt</td><td>vvi-miR403d|*-5_mismatch probe</td></tr>
<tr><td></td><td></td><td>vvi-miR403d|*+5_perfect match</td></tr>
<tr><td>vvi|miR-1146|nat|as|</td><td>ggttggattcgcgcacaaac</td><td>probe</td></tr>
<tr><td>vvi|miR-1146|2mut|as|</td><td>ggttggattagcccacaaac</td><td>vvi-miR403d|*+5_mismatch probe</td></tr>
<tr><td></td><td></td><td>vvi-miR403e|*-5_perfect match</td></tr>
<tr><td>vvi|miR-1147|nat|as|</td><td>tcacgcacaaacttgagatg</td><td>probe</td></tr>
<tr><td>vvi|miR-1147|2mut|as|</td><td>tcacccacaaacttaagatg</td><td>vvi-miR403e|*-5_mismatch probe</td></tr>
<tr><td></td><td></td><td>vvi-miR403e|*+5_perfect match</td></tr>
<tr><td>vvi|miR-1148|nat|as|</td><td>tggggtttgattcacgcaca</td><td>probe</td></tr>
<tr><td>vvi|miR-1148|2mut|as|</td><td>tgggctttgattcacccaca</td><td>vvi-miR403e|*+5_mismatch probe</td></tr>
<tr><td>vvi|miR-1149|nat|as|</td><td>gtcacgcacaaactcctcat</td><td>vvi-miR403f|*-5_perfect match probe</td></tr>
<tr><td>vvi|miR-1149|2mut|as|</td><td>gtcacccacaaacttctcat</td><td>vvi-miR403f|*-5_mismatch probe</td></tr>
<tr><td></td><td></td><td>vvi-miR403f|*+5_perfect match</td></tr>
<tr><td>vvi|miR-1150|nat|as|</td><td>cggttttagagtcacgcaca</td><td>probe</td></tr>
<tr><td>vvi|miR-1150|2mut|as|</td><td>cggttttagcgtcacccaca</td><td>vvi-miR403f|*+5_mismatch probe</td></tr>
<tr><td>vvi|miR-1151|nat|as|</td><td>actacctcgtccccgtcttc</td><td>vvi-miR408|*-5_perfect match probe</td></tr>
<tr><td>vvi|miR-1151|2mut|as|</td><td>actacctcctcccagtcttc</td><td>vvi-miR408|*-5_mismatch probe</td></tr>
<tr><td>vvi|miR-1152|nat|as|</td><td>catccatgcactacctcgtc</td><td>vvi-miR408|*+5_perfect match probe</td></tr>
</table>

vvi|miR-1152|2mut|as|	catccatgaactatctcgtc
vvi|miR-1153|nat|as|	cccactgtcttccaactttc
vvi|miR-1153|2mut|as|	cccactctcttcaaactttc
vvi|miR-1154|nat|as|	ttccaaggtcccccactgtc
vvi|miR-1154|2mut|as|	ttccaagctccctcactgtc
vvi|miR-1155|nat|as|	ttggttcggctcgtctttga
vvi|miR-1155|2mut|as|	ttggttcgcctcctctttga
vvi|miR-1156|nat|as|	caagagtgatattggttcgg
vvi|miR-1156|2mut|as|	caagagcgatattcgttcgg
vvi|miR-1157|nat|as|	ctactctccaattcccaaac
vvi|miR-1157|2mut|as|	ctactctcaaattctcaaac
vvi|miR-1158|nat|as|	gctttcctactctccaattc
vvi|miR-1158|2mut|as|	gcttttctactctcaaattc
vvi|miR-1159|nat|as|	gagagagagcacgcctgtac
vvi|miR-1159|2mut|as|	gagagagagaacccctgtac
vvi|miR-1160|nat|as|	ttgtatgacagcgagagaga
vvi|miR-1160|2mut|as|	ttgtatgaaagagagagaga
vvi|miR-1161|nat|as|	gagagagagcacgcctgtac
vvi|miR-1161|2mut|as|	gagagagagaacccctgtac
vvi|miR-1162|nat|as|	ttgtatgacagcgagagaga
vvi|miR-1162|2mut|as|	ttgtatgaaagagagagaga
vvi|miR-1163|nat|as|	gagagagagcacgcctgtac
vvi|miR-1163|2mut|as|	gagagagagaacccctgtac
vvi|miR-1164|nat|as|	ttgtatgacagcgagagaga

vvi-miR408|*+5_mismatch probe
vvi-miR477a|*-5_perfect match probe
vvi-miR477a|*-5_mismatch probe
vvi-miR477a|*+5_perfect match probe
vvi-miR477a|*+5_mismatch probe
vvi-miR479|*-5_perfect match probe
vvi-miR479|*-5_mismatch probe
vvi-miR479|*+5_perfect match probe
vvi-miR479|*+5_mismatch probe
vvi-miR482a|*-5_perfect match probe
vvi-miR482a|*-5_mismatch probe
vvi-miR482a|*+5_perfect match probe
vvi-miR482a|*+5_mismatch probe
vvi-miR535a|*-5_perfect match probe
vvi-miR535a|*-5_mismatch probe
vvi-miR535a|*+5_perfect match probe
vvi-miR535a|*+5_mismatch probe
vvi-miR535b|*-5_perfect match probe
vvi-miR535b|*-5_mismatch probe
vvi-miR535b|*+5_perfect match probe
vvi-miR535b|*+5_mismatch probe
vvi-miR535c|*-5_perfect match probe
vvi-miR535c|*-5_mismatch probe
vvi-miR535c|*+5_perfect match probe

vvi|miR-1164|2mut|as|	ttgtatgaaagagagagaga
vvi|miR-1165|nat|as|	gacagagagcacgcctgtac
vvi|miR-1165|2mut|as|	gacagagagaacccctgtac
vvi|miR-1166|nat|as|	ttgtatgacagcgacagaga
vvi|miR-1166|2mut|as|	ttgtatgaaagagacagaga
vvi|miR-1167|nat|as|	gacagagagcacgcctgtac
vvi|miR-1167|2mut|as|	gacagagagaacccctgtac
vvi|miR-1168|nat|as|	ttgtatgacagcgacagaga
vvi|miR-1168|2mut|as|	ttgtatgaaagagacagaga
vvi|miR-1169|nat|as|	caaatgagcatctcaacaac
vvi|miR-1169|2mut|as|	caaatgagaatctaaacaac
vvi|miR-1170|nat|as|	ggttgcttcctcaaatgagc
vvi|miR-1170|2mut|as|	ggttccttcctaaaatgagc
vvi|miR-1171|nat|as|	ggaaatgactatccaggttg
vvi|miR-1171|2mut|as|	ggaaatgcctatcaaggttg
vvi|miR-1172|nat|as|	tggttttgcaggaaatgact
vvi|miR-1172|2mut|as|	tggttttgaagaaaatgact
vvi|miR-1173|nat|as|	catgaaggaactaatcaagt
vvi|miR-1173|2mut|as|	catgaagaaactaataaagt
vvi|miR-1174|nat|as|	tgcaataactcatgaaggaa
vvi|miR-1174|2mut|as|	tgcaataattcataaaggaa
vvi|miR-1175|nat|as|	catgaaggaactaatcaagt
vvi|miR-1175|2mut|as|	catgaagaaactaataaagt

vvi-miR535c|*+5_mismatch probe
vvi-miR535d|*-5_perfect match probe
vvi-miR535d|*-5_mismatch probe
vvi-miR535d|*+5_perfect match probe
vvi-miR535d|*+5_mismatch probe
vvi-miR535e|*-5_perfect match probe
vvi-miR535e|*-5_mismatch probe
vvi-miR535e|*+5_perfect match probe
vvi-miR535e|*+5_mismatch probe
vvi-miR828a|*-5_perfect match probe
vvi-miR828a|*-5_mismatch probe
vvi-miR828a|*+5_perfect match probe
vvi-miR828a|*+5_mismatch probe
vvi-miR828b|*-5_perfect match probe
vvi-miR828b|*-5_mismatch probe
vvi-miR828b|*+5_perfect match probe
vvi-miR828b|*+5_mismatch probe
vvi-miR845a|*-5_perfect match probe
vvi-miR845a|*-5_mismatch probe
vvi-miR845a|*+5_perfect match probe
vvi-miR845a|*+5_mismatch probe
vvi-miR845b|*-5_perfect match probe
vvi-miR845b|*-5_mismatch probe

<table>
<tr><td>vvi|miR-1176|nat|as|</td><td>gaaataactcatgaaggaac</td><td>vvi-miR845b|*+5_perfect match probe</td></tr>
<tr><td>vvi|miR-1176|2mut|as|</td><td>gaaataactaatgaacgaac</td><td>vvi-miR845b|*+5_mismatch probe</td></tr>
<tr><td>vvi|miR-1177|nat|as|</td><td>cagacaaccaaaacacataa</td><td>vvi-miR845c|*-5_perfect match probe</td></tr>
<tr><td>vvi|miR-1177|2mut|as|</td><td>cagacaacaaaaacccataa</td><td>vvi-miR845c|*-5_mismatch probe</td></tr>
<tr><td>vvi|miR-1178|nat|as|</td><td>acatgaaactcagacaacca</td><td>vvi-miR845c|*+5_perfect match probe</td></tr>
<tr><td>vvi|miR-1178|2mut|as|</td><td>acataaaactcagaaaacca</td><td>vvi-miR845c|*+5_mismatch probe</td></tr>
<tr><td>vvi|miR-1179|nat|as|</td><td>tagggataattaatgttggt</td><td>vvi-miR845d|*-5_perfect match probe</td></tr>
<tr><td>vvi|miR-1179|2mut|as|</td><td>taggaataattaatcttggt</td><td>vvi-miR845d|*-5_mismatch probe</td></tr>
<tr><td>vvi|miR-1180|nat|as|</td><td>tcccatggccctagggataa</td><td>vvi-miR845d|*+5_perfect match probe</td></tr>
<tr><td>vvi|miR-1180|2mut|as|</td><td>tcccatggtcctagtgataa</td><td>vvi-miR845d|*+5_mismatch probe</td></tr>
<tr><td>vvi|miR-1181|nat|as|</td><td>tagggataattaatgtgcgt</td><td>vvi-miR845e|*-5_perfect match probe</td></tr>
<tr><td>vvi|miR-1181|2mut|as|</td><td>taggaataattaatgcgcgt</td><td>vvi-miR845e|*-5_mismatch probe</td></tr>
<tr><td>vvi|miR-1182|nat|as|</td><td>tcccatggccctagggataa</td><td>vvi-miR845e|*+5_perfect match probe</td></tr>
<tr><td>vvi|miR-1182|2mut|as|</td><td>tcccatggtcctagtgataa</td><td>vvi-miR845e|*+5_mismatch probe</td></tr>
<tr><td>vvi|miR-1183|nat|as|</td><td>gagagagcacactcccaaga</td><td>vvi-miR156a|*-10_perfect match probe</td></tr>
<tr><td>vvi|miR-1183|2mut|as|</td><td>gagagagaacactctcaaga</td><td>vvi-miR156a|*-10_mismatch probe</td></tr>
<tr><td>vvi|miR-1184|nat|as|</td><td>gtgtgatgatgacagaagaa</td><td>vvi-miR156a|*+10_perfect match probe</td></tr>
<tr><td>vvi|miR-1184|2mut|as|</td><td>gtgtgataatgaaagaagaa</td><td>vvi-miR156a|*+10_mismatch probe</td></tr>
<tr><td>vvi|miR-1185|nat|as|</td><td>aaatgagcacgcaaaagcaa</td><td>vvi-miR156b|*-10_perfect match probe</td></tr>
<tr><td>vvi|miR-1185|2mut|as|</td><td>aaatgagaacccaaaagcaa</td><td>vvi-miR156b|*-10_mismatch probe</td></tr>
<tr><td>vvi|miR-1186|nat|as|</td><td>ctggaagctgacagaaagag</td><td>vvi-miR156b|*+10_perfect match probe</td></tr>
<tr><td>vvi|miR-1186|2mut|as|</td><td>ctggaagttgaaagaaagag</td><td>vvi-miR156b|*+10_mismatch probe</td></tr>
<tr><td>vvi|miR-1187|nat|as|</td><td>gagtgagcacgcagagcttc</td><td>vvi-miR156c|*-10_perfect match</td></tr>
</table>

		probe
vvi|miR-1187|2mut|as|	gagtgagaacccagagcttc	vvi-miR156c|*-10_mismatch probe
		vvi-miR156c|*+10_perfect match
vvi|miR-1188|nat|as|	gaggacagatgacagataga	probe
vvi|miR-1188|2mut|as|	gaggaaagatgaaagataga	vvi-miR156c|*+10_mismatch probe
		vvi-miR156d|*-10_perfect match
vvi|miR-1189|nat|as|	aggtgagcacgcaccggcat	probe
vvi|miR-1189|2mut|as|	aggtgagaacccaccggcat	vvi-miR156d|*-10_mismatch probe
		vvi-miR156d|*+10_perfect match
vvi|miR-1190|nat|as|	cctgaagctgacagaaagag	probe
vvi|miR-1190|2mut|as|	cctgaagttgaaagaaagag	vvi-miR156d|*+10_mismatch probe
		vvi-miR156e|*-10_perfect match
vvi|miR-1191|nat|as|	gagtaagcacacagagcttc	probe
vvi|miR-1191|2mut|as|	gagtaagaacacagaccttc	vvi-miR156e|*-10_mismatch probe
		vvi-miR156e|*+10_perfect match
vvi|miR-1192|nat|as|	cttgaggggtgacagatagg	probe
vvi|miR-1192|2mut|as|	cttgaggtgtgaaagatagg	vvi-miR156e|*+10_mismatch probe
		vvi-miR156f|*-10_perfect match
vvi|miR-1193|nat|as|	gagagaggacaagactgctc	probe
vvi|miR-1193|2mut|as|	gagagagaacaagcctgctc	vvi-miR156f|*-10_mismatch probe
vvi|miR-1194|nat|as|	gacaggagaagagagaggac	vvi-miR156f|*0_perfect match probe
vvi|miR-1194|2mut|as|	gacagaagaagagagcggac	vvi-miR156f|*0_mismatch probe
		vvi-miR156f|*+10_perfect match
vvi|miR-1195|nat|as|	aagttctgttgacaggagaa	probe
vvi|miR-1195|2mut|as|	aagttctcttgacagaagaa	vvi-miR156f|*+10_mismatch probe
		vvi-miR156g|*-10_perfect match
vvi|miR-1196|nat|as|	tagagagcacaaaggagtga	probe
vvi|miR-1196|2mut|as|	tagagagaacaaagaagtga	vvi-miR156g|*-10_mismatch probe
vvi|miR-1197|nat|as|	gacagaagtctagagagcac	vvi-miR156g|*0_perfect match probe
vvi|miR-1197|2mut|as|	gacagaagcctagagcgcac	vvi-miR156g|*0_mismatch probe
		vvi-miR156g|*+10_perfect match
vvi|miR-1198|nat|as|	ctgaggtgatgacagaagtc	probe
vvi|miR-1198|2mut|as|	ctgagctgatgaaagaagtc	vvi-miR156g|*+10_mismatch probe
vvi|miR-1199|nat|as|	cagaaagaactcagctgatc	vvi-miR156h|*-10_perfect match

		probe
vvi|miR-1199|2mut|as|	cagaaagaattcagttgatc	vvi-miR156h|*-10_mismatch probe
vvi|miR-1200|nat|as|	gcaggacacaatgaaaggcg	vvi-miR156h|*+10_perfect match probe
vvi|miR-1200|2mut|as|	gcagaacacaataaaaggcg	vvi-miR156h|*+10_mismatch probe
vvi|miR-1201|nat|as|	tagagagcacaaaggagtga	vvi-miR156i|*-10_perfect match probe
vvi|miR-1201|2mut|as|	tagagagaacaaagaagtga	vvi-miR156i|*-10_mismatch probe
vvi|miR-1202|nat|as|	ttaaggtgatgacagaagaa	vvi-miR156i|*+10_perfect match probe
vvi|miR-1202|2mut|as|	ttaagctgatgaaagaagaa	vvi-miR156i|*+10_mismatch probe
vvi|miR-1203|nat|as|	aggagctcccataaaccc	vvi-miR159a|*-10_perfect match probe
vvi|miR-1203|2mut|as|	aggagctctaataaaccc	vvi-miR159a|*-10_mismatch probe
vvi|miR-1204|nat|as|	tggagcgtaaaggagctccc	vvi-miR159a|*0_perfect match probe
vvi|miR-1204|2mut|as|	tggagagtaaaggagttccc	vvi-miR159a|*0_mismatch probe
vvi|miR-1205|nat|as|	ctttcagaactggagcgtaa	vvi-miR159a|*+10_perfect match probe
vvi|miR-1205|2mut|as|	ctttccgaactggagagtaa	vvi-miR159a|*+10_mismatch probe
vvi|miR-1206|nat|as|	gaagctcccacaagccc	vvi-miR159b|*-10_perfect match probe
vvi|miR-1206|2mut|as|	gaagctctcccaagccc	vvi-miR159b|*-10_mismatch probe
vvi|miR-1207|nat|as|	ggagtgtaaagaagctccca	vvi-miR159b|*0_perfect match probe
vvi|miR-1207|2mut|as|	ggagcgtaaagaagttccca	vvi-miR159b|*0_mismatch probe
vvi|miR-1208|nat|as|	tttcagttctggagtgtaaa	vvi-miR159b|*+10_perfect match probe
vvi|miR-1208|2mut|as|	tttcagttcccgagtgtaaa	vvi-miR159b|*+10_mismatch probe
vvi|miR-1209|nat|as|	aggagctccactccaaaa	vvi-miR159c|*-10_perfect match probe
vvi|miR-1209|2mut|as|	aggagttccactcaaaaa	vvi-miR159c|*-10_mismatch probe
vvi|miR-1210|nat|as|	ttggacttcaaggagctcca	vvi-miR159c|*0_perfect match probe
vvi|miR-1210|2mut|as|	ttggccttcaaggagttcca	vvi-miR159c|*0_mismatch probe
vvi|miR-1211|nat|as|	agaccctctattggacttca	vvi-miR159c|*+10_perfect match probe

vvi|miR-1211|2mut|as|	agactctctattcgacttca
vvi|miR-1212|nat|as|	tctgatgccaactaggaaga
vvi|miR-1212|2mut|as|	tctgatgtcaactagaaaga
vvi|miR-1213|nat|as|	gcatgactcctctgatgcca
vvi|miR-1213|2mut|as|	gcatgacttctccgatgcca
vvi|miR-1214|nat|as|	gttggggcctgcatgactcc
vvi|miR-1214|2mut|as|	gttggggtctgaatgactcc
vvi|miR-1215|nat|as|	cctcatgccaaataggaagg
vvi|miR-1215|2mut|as|	cctcatgtcaaatagaaagg
vvi|miR-1216|nat|as|	gcttgactcccctcatgcca
vvi|miR-1216|2mut|as|	gcttgactcttctcatgcca
vvi|miR-1217|nat|as|	tgtttcgcctgcttgactcc
vvi|miR-1217|2mut|as|	tgtttcccctccttgactcc
vvi|miR-1218|nat|as|	tcgcacgccacccgcaaata
vvi|miR-1218|2mut|as|	tcgcaccccacccccaaata
vvi|miR-1219|nat|as|	gcttggctcctcgcacgcca
vvi|miR-1219|2mut|as|	gcttgcctcctcccacgcca
vvi|miR-1220|nat|as|	tggtgggtatgcttggctcc
vvi|miR-1220|2mut|as|	tggtgtgtatgcttgcctcc
vvi|miR-1221|nat|as|	tcgcacgccatccgcgaagg
vvi|miR-1221|2mut|as|	tcgcaccccatccgagaagg
vvi|miR-1222|nat|as|	gcatggctcctcgcacgcca
vvi|miR-1222|2mut|as|	gcatgcctcctcccacgcca
vvi|miR-1223|nat|as|	caagatgcatgcatggctcc
vvi|miR-1223|2mut|as|	caagatgaatgcatgcctcc
vvi|miR-1224|nat|as|	cctcatgccaaataggaagg

vvi-miR159c|*+10_mismatch probe
vvi-miR160a|*-10_perfect match probe
vvi-miR160a|*-10_mismatch probe
vvi-miR160a|*0_perfect match probe
vvi-miR160a|*0_mismatch probe
vvi-miR160a|*+10_perfect match probe
vvi-miR160a|*+10_mismatch probe
vvi-miR160b|*-10_perfect match probe
vvi-miR160b|*-10_mismatch probe
vvi-miR160b|*0_perfect match probe
vvi-miR160b|*0_mismatch probe
vvi-miR160b|*+10_perfect match probe
vvi-miR160b|*+10_mismatch probe
vvi-miR160c|*-10_perfect match probe
vvi-miR160c|*-10_mismatch probe
vvi-miR160c|*0_perfect match probe
vvi-miR160c|*0_mismatch probe
vvi-miR160c|*+10_perfect match probe
vvi-miR160c|*+10_mismatch probe
vvi-miR160d|*-10_perfect match probe
vvi-miR160d|*-10_mismatch probe
vvi-miR160d|*0_perfect match probe
vvi-miR160d|*0_mismatch probe
vvi-miR160d|*+10_perfect match probe
vvi-miR160d|*+10_mismatch probe
vvi-miR160e|*-10_perfect match

<table>
<tr><td></td><td></td><td>probe</td></tr>
<tr><td>vvi|miR-1224|2mut|as|</td><td>cctcatgtcaaatagaaagg</td><td>vvi-miR160e|*-10_mismatch probe</td></tr>
<tr><td>vvi|miR-1225|nat|as|</td><td>gcttgactcccctcatgcca</td><td>vvi-miR160e|*0_perfect match probe</td></tr>
<tr><td>vvi|miR-1225|2mut|as|</td><td>gcttgactcttctcatgcca</td><td>vvi-miR160e|*0_mismatch probe</td></tr>
<tr><td>vvi|miR-1226|nat|as|</td><td>tgtttcgcctgcttgactcc</td><td>vvi-miR160e|*+10_perfect match probe</td></tr>
<tr><td>vvi|miR-1226|2mut|as|</td><td>tgtttcccctccttgactcc</td><td>vvi-miR160e|*+10_mismatch probe</td></tr>
<tr><td>vvi|miR-1227|nat|as|</td><td>tcatacgccatccacgaagg</td><td>vvi-miR160f|*-10_perfect match probe</td></tr>
<tr><td>vvi|miR-1227|2mut|as|</td><td>tcataccccatccaagaagg</td><td>vvi-miR160f|*-10_mismatch probe</td></tr>
<tr><td>vvi|miR-1228|nat|as|</td><td>gcatggctcctcatacgcca</td><td>vvi-miR160f|*0_perfect match probe</td></tr>
<tr><td>vvi|miR-1228|2mut|as|</td><td>gcatgcctcctcataagcca</td><td>vvi-miR160f|*0_mismatch probe</td></tr>
<tr><td>vvi|miR-1229|nat|as|</td><td>tggggcatatgcatggctcc</td><td>vvi-miR160f|*+10_perfect match probe</td></tr>
<tr><td>vvi|miR-1229|2mut|as|</td><td>tgggccatatgcatgcctcc</td><td>vvi-miR160f|*+10_mismatch probe</td></tr>
<tr><td>vvi|miR-1230|nat|as|</td><td>ctgcatccagtgacttca</td><td>vvi-miR162|*-10_perfect match probe</td></tr>
<tr><td>vvi|miR-1230|2mut|as|</td><td>ctgcatcaagcgacttca</td><td>vvi-miR162|*-10_mismatch probe</td></tr>
<tr><td>vvi|miR-1231|nat|as|</td><td>cgatgaaccgctgcatccag</td><td>vvi-miR162|*0_perfect match probe</td></tr>
<tr><td>vvi|miR-1231|2mut|as|</td><td>cgatgaacccctgaatccag</td><td>vvi-miR162|*0_mismatch probe</td></tr>
<tr><td>vvi|miR-1232|nat|as|</td><td>aggaagagatcgatgaaccg</td><td>vvi-miR162|*+10_perfect match probe</td></tr>
<tr><td>vvi|miR-1232|2mut|as|</td><td>aggaagcgatagatgaaccg</td><td>vvi-miR162|*+10_mismatch probe</td></tr>
<tr><td>vvi|miR-1233|nat|as|</td><td>gagcacgtgcaagcctgtgg</td><td>vvi-miR164a|*-10_perfect match probe</td></tr>
<tr><td>vvi|miR-1233|2mut|as|</td><td>gagcacgtgaaagtctgtgg</td><td>vvi-miR164a|*-10_mismatch probe</td></tr>
<tr><td>vvi|miR-1234|nat|as|</td><td>tggagaaggggagcacgtgc</td><td>vvi-miR164a|*0_perfect match probe</td></tr>
<tr><td>vvi|miR-1234|2mut|as|</td><td>tggagaaggtgagaacgtgc</td><td>vvi-miR164a|*0_mismatch probe</td></tr>
<tr><td>vvi|miR-1235|nat|as|</td><td>gaacccatgttggagaaggg</td><td>vvi-miR164a|*+10_perfect match probe</td></tr>
<tr><td>vvi|miR-1235|2mut|as|</td><td>gaactcatgttcgagaaggg</td><td>vvi-miR164a|*+10_mismatch probe</td></tr>
<tr><td>vvi|miR-1236|nat|as|</td><td>agggcacatgtaaaatctca</td><td>vvi-miR164b|*-10_perfect match probe</td></tr>
<tr><td>vvi|miR-1236|2mut|as|</td><td>agggaacatgtaaaacctca</td><td>vvi-miR164b|*-10_mismatch probe</td></tr>
</table>

vvi|miR-1237|nat|as|	tgggagagccagggcacatg	vvi-miR164b|*0_perfect match probe
vvi|miR-1237|2mut|as|	tgggagagtcaggccacatg	vvi-miR164b|*0_mismatch probe
		vvi-miR164b|*+10_perfect match
vvi|miR-1238|nat|as|	aggatccaagtgggagagcc	probe
vvi|miR-1238|2mut|as|	aggatcaaagtgtgagagcc	vvi-miR164b|*+10_mismatch probe
		vvi-miR164d|*-10_perfect match
vvi|miR-1239|nat|as|	agcacgtgcagagcagatta	probe
vvi|miR-1239|2mut|as|	agcacgtgaagagaagatta	vvi-miR164d|*-10_mismatch probe
vvi|miR-1240|nat|as|	ggagaaggggagcacgtgca	vvi-miR164d|*0_perfect match probe
vvi|miR-1240|2mut|as|	ggagaaggtgagaacgtgca	vvi-miR164d|*0_mismatch probe
		vvi-miR164d|*+10_perfect match
vvi|miR-1241|nat|as|	aacccatgttggagaagggg	probe
vvi|miR-1241|2mut|as|	aaccaatgttcgagaagggg	vvi-miR164d|*+10_mismatch probe
		vvi-miR166c|*-10_perfect match
vvi|miR-1242|nat|as|	aacattcccctcaaaatag	probe
vvi|miR-1242|2mut|as|	aacattcctctaaaaatag	vvi-miR166c|*-10_mismatch probe
vvi|miR-1243|nat|as|	cgagccagacaacattcccc	vvi-miR166c|*0_perfect match probe
vvi|miR-1243|2mut|as|	cgagtcagacaaaattcccc	vvi-miR166c|*0_mismatch probe
		vvi-miR166c|*+10_perfect match
vvi|miR-1244|nat|as|	taagtgtcctcgagccagac	probe
vvi|miR-1244|2mut|as|	taagtgttctcgagtcagac	vvi-miR166c|*+10_mismatch probe
		vvi-miR166d|*-10_perfect match
vvi|miR-1245|nat|as|	acaatcccctcaaagcag	probe
vvi|miR-1245|2mut|as|	acaatcctctaaaagcag	vvi-miR166d|*-10_mismatch probe
vvi|miR-1246|nat|as|	gagccagacaacaatcccct	vvi-miR166d|*0_perfect match probe
vvi|miR-1246|2mut|as|	gagcaagacaacaattccct	vvi-miR166d|*0_mismatch probe
		vvi-miR166d|*+10_perfect match
vvi|miR-1247|nat|as|	tggtggcctcgagccagaca	probe
vvi|miR-1247|2mut|as|	tggtggtctcgagtcagaca	vvi-miR166d|*+10_mismatch probe
		vvi-miR166e|*-10_perfect match
vvi|miR-1248|nat|as|	aacattcccctcaaaacac	probe
vvi|miR-1248|2mut|as|	aacattcctctaaaaacac	vvi-miR166e|*-10_mismatch probe
vvi|miR-1249|nat|as|	cgagccagacaacattcccc	vvi-miR166e|*0_perfect match probe
vvi|miR-1249|2mut|as|	cgagtcagacaaaattcccc	vvi-miR166e|*0_mismatch probe

vvi|miR-1250|nat|as|	ttggtgtcctcgagccagac	vvi-miR166e|*+10_perfect match probe
vvi|miR-1250|2mut|as|	ttggtgttctcgagtcagac	vvi-miR166e|*+10_mismatch probe
vvi|miR-1251|nat|as|	aacattccccacaactaca	vvi-miR166f|*-10_perfect match probe
vvi|miR-1251|2mut|as|	aacattcctcccaactaca	vvi-miR166f|*-10_mismatch probe
vvi|miR-1252|nat|as|	cgagccagccaacattcccc	vvi-miR166f|*0_perfect match probe
vvi|miR-1252|2mut|as|	cgagccagtcaaaattcccc	vvi-miR166f|*0_mismatch probe
vvi|miR-1253|nat|as|	aagaaagcctcgagccagcc	vvi-miR166f|*+10_perfect match probe
vvi|miR-1253|2mut|as|	aagaaagtctcgagtcagcc	vvi-miR166f|*+10_mismatch probe
vvi|miR-1254|nat|as|	acattcccctcaactgtt	vvi-miR166g|*-10_perfect match probe
vvi|miR-1254|2mut|as|	acattcctctaaactgtt	vvi-miR166g|*-10_mismatch probe
vvi|miR-1255|nat|as|	gaaccagacaacattcccct	vvi-miR166g|*0_perfect match probe
vvi|miR-1255|2mut|as|	gaacaagacaacatttccct	vvi-miR166g|*0_mismatch probe
vvi|miR-1256|nat|as|	gaaggatctcgaaccagaca	vvi-miR166g|*+10_perfect match probe
vvi|miR-1256|2mut|as|	gaaggatctaaaaccagaca	vvi-miR166g|*+10_mismatch probe
vvi|miR-1257|nat|as|	agcgttcccctcaactgtt	vvi-miR166h|*-10_perfect match probe
vvi|miR-1257|2mut|as|	agcgttcctctaaactgtt	vvi-miR166h|*-10_mismatch probe
vvi|miR-1258|nat|as|	cgaaccagacagcgttcccc	vvi-miR166h|*0_perfect match probe
vvi|miR-1258|2mut|as|	cgaacaagacagagttcccc	vvi-miR166h|*0_mismatch probe
vvi|miR-1259|nat|as|	cgagtggtctcgaaccagac	vvi-miR166h|*+10_perfect match probe
vvi|miR-1259|2mut|as|	cgagtcgtctagaaccagac	vvi-miR166h|*+10_mismatch probe
vvi|miR-1260|nat|as|	ctcattccaaacacccgc	vvi-miR166a|*-10_perfect match probe
vvi|miR-1260|2mut|as|	ctcattcaaaacatccgc	vvi-miR166a|*-10_mismatch probe
vvi|miR-1261|nat|as|	tggatcaaacctcattccaa	vvi-miR166a|*0_perfect match probe
vvi|miR-1261|2mut|as|	tggatcaaatttcattccaa	vvi-miR166a|*0_mismatch probe
vvi|miR-1262|nat|as|	ggaaggatcttggatcaaac	vvi-miR166a|*+10_perfect match probe

vvi|miR-1262|2mut|as|	ggaagaatcttcgatcaaac
vvi|miR-1263|nat|as|	aacattcccctcaaaaaac
vvi|miR-1263|2mut|as|	aacattcctctaaaaaaac
vvi|miR-1264|nat|as|	cgagccagccaacattcccc
vvi|miR-1264|2mut|as|	cgagccagtcaaaattcccc
vvi|miR-1265|nat|as|	gtgaatgcctcgagccagcc
vvi|miR-1265|2mut|as|	gtgaatgtctcgagtcagcc
vvi|miR-1266|nat|as|	agatgacctgatcatggaac
vvi|miR-1266|2mut|as|	agatgatctgatcatcgaac
vvi|miR-1267|nat|as|	tgaagctgcaagatgacctg
vvi|miR-1267|2mut|as|	tgaagctgaaagatgccctg
vvi|miR-1268|nat|as|	agtgagtgattgaagctgca
vvi|miR-1268|2mut|as|	agtgagcgattgaagttgca
vvi|miR-1269|nat|as|	acatgatctagtctttcctg
vvi|miR-1269|2mut|as|	acataatctagcctttcctg
vvi|miR-1270|nat|as|	tgaaactgccacatgatcta
vvi|miR-1270|2mut|as|	tgaaactgtcccatgatcta
vvi|miR-1271|nat|as|	catcaacaggtgaaactgcc
vvi|miR-1271|2mut|as|	catcaacagccgaaactgcc
vvi|miR-1272|nat|as|	cacatgatctgatctttcct
vvi|miR-1272|2mut|as|	cacatgatccaatctttcct
vvi|miR-1273|nat|as|	ccatcaacaggtgaggctac
vvi|miR-1273|2mut|as|	ccatcaacacgtgagcctac
vvi|miR-1274|nat|as|	gcatgacctagctagggtta
vvi|miR-1274|2mut|as|	gcatgatctagctagtgtta

vvi-miR166a|*+10_mismatch probe
vvi-miR166b|*-10_perfect match probe
vvi-miR166b|*-10_mismatch probe
vvi-miR166b|*0_perfect match probe
vvi-miR166b|*0_mismatch probe
vvi-miR166b|*+10_perfect match probe
vvi-miR166b|*+10_mismatch probe
vvi-miR167a|*-10_perfect match probe
vvi-miR167a|*-10_mismatch probe
vvi-miR167a|*0_perfect match probe
vvi-miR167a|*0_mismatch probe
vvi-miR167a|*+10_perfect match probe
vvi-miR167a|*+10_mismatch probe
vvi-miR167b|*-10_perfect match probe
vvi-miR167b|*-10_mismatch probe
vvi-miR167b|*0_perfect match probe
vvi-miR167b|*0_mismatch probe
vvi-miR167b|*+10_perfect match probe
vvi-miR167b|*+10_mismatch probe
vvi-miR167c|*-10_perfect match probe
vvi-miR167c|*-10_mismatch probe
vvi-miR167c|*+10_perfect match probe
vvi-miR167c|*+10_mismatch probe
vvi-miR167d|*-10_perfect match probe
vvi-miR167d|*-10_mismatch probe

vvi|miR-1275|nat|as|	ggctgtcagggcatgaccta	vvi-miR167d|*0_perfect match probe
vvi|miR-1275|2mut|as|	ggctgtcagtccatgaccta	vvi-miR167d|*0_mismatch probe
		vvi-miR167d|*+10_perfect match
vvi|miR-1276|nat|as|	gaaggagtgaggctgtcagg	probe
vvi|miR-1276|2mut|as|	gaagaagtgagcctgtcagg	vvi-miR167d|*+10_mismatch probe
		vvi-miR167e|*-10_perfect match
vvi|miR-1277|nat|as|	cacatgatctgatctctgtt	probe
vvi|miR-1277|2mut|as|	cacatgatccaatctctgtt	vvi-miR167e|*-10_mismatch probe
vvi|miR-1278|nat|as|	gtgaaactgccacatgatct	vvi-miR167e|*0_perfect match probe
vvi|miR-1278|2mut|as|	gtgaaactgtaacatgatct	vvi-miR167e|*0_mismatch probe
		vvi-miR167e|*+10_perfect match
vvi|miR-1279|nat|as|	ccaacaacaggtgaaactgc	probe
vvi|miR-1279|2mut|as|	ccaacaacacctgaaactgc	vvi-miR167e|*+10_mismatch probe
		vvi-miR168|*-10_perfect match
vvi|miR-1280|nat|as|	caaggcgggatcggaattcg	probe
vvi|miR-1280|2mut|as|	caaggagggatcagaattcg	vvi-miR168|*-10_mismatch probe
vvi|miR-1281|nat|as|	tcagttgatgcaaggcggga	vvi-miR168|*0_perfect match probe
vvi|miR-1281|2mut|as|	tcagttgatccaaggaggga	vvi-miR168|*0_mismatch probe
		vvi-miR168|*+10_perfect match
vvi|miR-1282|nat|as|	cgtctccgattcagttgatg	probe
vvi|miR-1282|2mut|as|	cgtctcagattccgttgatg	vvi-miR168|*+10_mismatch probe
		vvi-miR169a|*-10_perfect match
vvi|miR-1283|nat|as|	aacttgccagggttcactat	probe
vvi|miR-1283|2mut|as|	aacttgtcagtgttcactat	vvi-miR169a|*-10_mismatch probe
vvi|miR-1284|nat|as|	agccaaggacaacttgccag	vvi-miR169a|*0_perfect match probe
vvi|miR-1284|2mut|as|	agccaagaacaacttcccag	vvi-miR169a|*0_mismatch probe
		vvi-miR169a|*+10_perfect match
vvi|miR-1285|nat|as|	aatcaaatgtagccaaggac	probe
vvi|miR-1285|2mut|as|	aatcaaatctagtcaaggac	vvi-miR169a|*+10_mismatch probe
		vvi-miR169y|*-10_perfect match
vvi|miR-1286|nat|as|	gagacttgccataaaccaca	probe
vvi|miR-1286|2mut|as|	gagacttgtcataaatcaca	vvi-miR169y|*-10_mismatch probe
vvi|miR-1287|nat|as|	aggtgccaaggagacttgcc	vvi-miR169y|*0_perfect match probe
vvi|miR-1287|2mut|as|	aggtgtcaagaagacttgcc	vvi-miR169y|*0_mismatch probe

vvi|miR-1288|nat|as|	gaggccatcaggtgccaag	vvi-miR169y|*+10_perfect match probe
vvi|miR-1288|2mut|as|	gaggtcatcaggtgtcaag	vvi-miR169y|*+10_mismatch probe
vvi|miR-1289|nat|as|	tgactaaccgtgcacgcaca	vvi-miR169b|*-10_perfect match probe
vvi|miR-1289|2mut|as|	tgactaacagtgcacccaca	vvi-miR169b|*-10_mismatch probe
vvi|miR-1290|nat|as|	gagccaaggatgactaaccg	vvi-miR169b|*0_perfect match probe
vvi|miR-1290|2mut|as|	gagcaaaggatgcctaaccg	vvi-miR169b|*0_mismatch probe
vvi|miR-1291|nat|as|	agggccaaatgagccaagga	vvi-miR169b|*+10_perfect match probe
vvi|miR-1291|2mut|as|	agggtcaaatgagtcaagga	vvi-miR169b|*+10_mismatch probe
vvi|miR-1292|nat|as|	aacttgccggattaacgagg	vvi-miR169c|*-10_perfect match probe
vvi|miR-1292|2mut|as|	aacttgtcggattaaagagg	vvi-miR169c|*-10_mismatch probe
vvi|miR-1293|nat|as|	agccaaggacaacttgccgg	vvi-miR169c|*0_perfect match probe
vvi|miR-1293|2mut|as|	agccaagaacaacttcccgg	vvi-miR169c|*0_mismatch probe
vvi|miR-1294|nat|as|	agcccaatgtagccaaggac	vvi-miR169c|*+10_perfect match probe
vvi|miR-1294|2mut|as|	agccaaatgtagtcaaggac	vvi-miR169c|*+10_mismatch probe
vvi|miR-1295|nat|as|	aacttgccagggttcactat	vvi-miR169d|*-10_perfect match probe
vvi|miR-1295|2mut|as|	aacttgtcagtgttcactat	vvi-miR169d|*-10_mismatch probe
vvi|miR-1296|nat|as|	agccaaggacaacttgccag	vvi-miR169d|*0_perfect match probe
vvi|miR-1296|2mut|as|	agccaagaacaacttcccag	vvi-miR169d|*0_mismatch probe
vvi|miR-1297|nat|as|	aatcaaatgtagccaaggac	vvi-miR169d|*+10_perfect match probe
vvi|miR-1297|2mut|as|	aatcaaatctagtcaaggac	vvi-miR169d|*+10_mismatch probe
vvi|miR-1298|nat|as|	tgactgcctggaacccaaac	vvi-miR169e|*-10_perfect match probe
vvi|miR-1298|2mut|as|	tgactgtctggaactcaaac	vvi-miR169e|*-10_mismatch probe
vvi|miR-1299|nat|as|	ttagccaaggtgactgcctg	vvi-miR169e|*0_perfect match probe
vvi|miR-1299|2mut|as|	ttagtcaaggtgactccctg	vvi-miR169e|*0_mismatch probe
vvi|miR-1300|nat|as|	gcctgtaaaattagccaagg	vvi-miR169e|*+10_perfect match probe

vvi|miR-1300|2mut|as|	gcctctaaaattagtcaagg
vvi|miR-1301|nat|as|	acaacttgcccattattgat
vvi|miR-1301|2mut|as|	acaacttgtcaattattgat
vvi|miR-1302|nat|as|	gtagccaaacacaacttgcc
vvi|miR-1302|2mut|as|	gtagtcaaacccaacttgcc
vvi|miR-1303|nat|as|	agataaacatgtagccaaac
vvi|miR-1303|2mut|as|	agataaaaatgtagtcaaac
vvi|miR-1304|nat|as|	acaacttgccggcttcatca
vvi|miR-1304|2mut|as|	acaacttgtcgccttcatca
vvi|miR-1305|nat|as|	gtagccaaagacaacttgcc
vvi|miR-1305|2mut|as|	gtagtcaaagaaaacttgcc
vvi|miR-1306|nat|as|	aaagaagcatgtagccaaag
vvi|miR-1306|2mut|as|	aaagaagaatgtagtcaaag
vvi|miR-1307|nat|as|	taaccctccgtgcgtgacca
vvi|miR-1307|2mut|as|	taaccctcagtgagtgacca
vvi|miR-1308|nat|as|	gagtcaaggataaccctccg
vvi|miR-1308|2mut|as|	gagtcaagaataactctccg
vvi|miR-1309|nat|as|	ggagctaaaggagtcaagga
vvi|miR-1309|2mut|as|	ggagttaaagaagtcaagga
vvi|miR-1310|nat|as|	aaccagccgtgcatgaccag
vvi|miR-1310|2mut|as|	aaccagtcgtgaatgaccag
vvi|miR-1311|nat|as|	agacagggataaccagccgt
vvi|miR-1311|2mut|as|	agacagtgataaccacccgt
vvi|miR-1312|nat|as|	gagctaaaggagacagggat
vvi|miR-1312|2mut|as|	gagctaaagaagacacggat
vvi|miR-1313|nat|as|	aacctgccagggacaccatg

vvi-miR169e|*+10_mismatch probe
vvi-miR169f|*-10_perfect match probe
vvi-miR169f|*-10_mismatch probe
vvi-miR169f|*0_perfect match probe
vvi-miR169f|*0_mismatch probe
vvi-miR169f|*+10_perfect match probe
vvi-miR169f|*+10_mismatch probe
vvi-miR169g|*-10_perfect match probe
vvi-miR169g|*-10_mismatch probe
vvi-miR169g|*0_perfect match probe
vvi-miR169g|*0_mismatch probe
vvi-miR169g|*+10_perfect match probe
vvi-miR169g|*+10_mismatch probe
vvi-miR169h|*-10_perfect match probe
vvi-miR169h|*-10_mismatch probe
vvi-miR169h|*0_perfect match probe
vvi-miR169h|*0_mismatch probe
vvi-miR169h|*+10_perfect match probe
vvi-miR169h|*+10_mismatch probe
vvi-miR169i|*-10_perfect match probe
vvi-miR169i|*-10_mismatch probe
vvi-miR169i|*0_perfect match probe
vvi-miR169i|*0_mismatch probe
vvi-miR169i|*+10_perfect match probe
vvi-miR169i|*+10_mismatch probe
vvi-miR169j|*-10_perfect match

		probe
vvi|miR-1313|2mut|as|	aacctgtcagtgacaccatg	vvi-miR169j|*-10_mismatch probe
vvi|miR-1314|nat|as|	agccaaggacaacctgccag	vvi-miR169j|*0_perfect match probe
vvi|miR-1314|2mut|as|	agccaagaacaacctcccag	vvi-miR169j|*0_mismatch probe
		vvi-miR169j|*+10_perfect match
vvi|miR-1315|nat|as|	agcgaaaggtagccaaggac	probe
vvi|miR-1315|2mut|as|	agcgaaagctagtcaaggac	vvi-miR169j|*+10_mismatch probe
		vvi-miR169k|*-10_perfect match
vvi|miR-1316|nat|as|	aacctgccagggacaccatg	probe
vvi|miR-1316|2mut|as|	aacctgtcagtgacaccatg	vvi-miR169k|*-10_mismatch probe
vvi|miR-1317|nat|as|	agccaaggacaacctgccag	vvi-miR169k|*0_perfect match probe
vvi|miR-1317|2mut|as|	agccaagaacaacctcccag	vvi-miR169k|*0_mismatch probe
		vvi-miR169k|*+10_perfect match
vvi|miR-1318|nat|as|	aacgaaaggtagccaaggac	probe
vvi|miR-1318|2mut|as|	aacgaaagctagtcaaggac	vvi-miR169k|*+10_mismatch probe
		vvi-miR169l|*-10_perfect match
vvi|miR-1319|nat|as|	aactagccgtgcatgcccag	probe
vvi|miR-1319|2mut|as|	aactagtcgtgcatgtccag	vvi-miR169l|*-10_mismatch probe
vvi|miR-1320|nat|as|	agccaagaataactagccgt	vvi-miR169l|*0_perfect match probe
vvi|miR-1320|2mut|as|	agccaacaataactacccgt	vvi-miR169l|*0_mismatch probe
		vvi-miR169l|*+10_perfect match
vvi|miR-1321|nat|as|	gggccaaatgagccaagaat	probe
vvi|miR-1321|2mut|as|	gggcaaaatgagtcaagaat	vvi-miR169l|*+10_mismatch probe
		vvi-miR169m|*-10_perfect match
vvi|miR-1322|nat|as|	tgcttgccagttatggccag	probe
vvi|miR-1322|2mut|as|	tgcttgtcagttatgcccag	vvi-miR169m|*-10_mismatch probe
		vvi-miR169m|*0_perfect match
vvi|miR-1323|nat|as|	gagcctcggatgcttgccag	probe
vvi|miR-1323|2mut|as|	gagcctcagatgcttcccag	vvi-miR169m|*0_mismatch probe
		vvi-miR169m|*+10_perfect match
vvi|miR-1324|nat|as|	gggtgaaacagagcctcgga	probe
vvi|miR-1324|2mut|as|	gggtgaaaaagagtctcgga	vvi-miR169m|*+10_mismatch probe
		vvi-miR169n|*-10_perfect match
vvi|miR-1325|nat|as|	gccagttatgaccagttaaa	probe

vvi|miR-1325|2mut|as|	gccagttataacaagttaaa	vvi-miR169n|*-10_mismatch probe
vvi|miR-1326|nat|as|	tcagatgcttgccagttatg	vvi-miR169n|*0_perfect match probe
vvi|miR-1326|2mut|as|	tcagatccttgtcagttatg	vvi-miR169n|*0_mismatch probe
vvi|miR-1327|nat|as|	aaatagagcctcagatgctt	vvi-miR169n|*+10_perfect match probe
vvi|miR-1327|2mut|as|	aaatagagtctccgatgctt	vvi-miR169n|*+10_mismatch probe
vvi|miR-1328|nat|as|	actcgctgtccatgcccagt	vvi-miR169o|*-10_perfect match probe
vvi|miR-1328|2mut|as|	actccctgtccatgtccagt	vvi-miR169o|*-10_mismatch probe
vvi|miR-1329|nat|as|	gccaagaataactcgctgtc	vvi-miR169o|*0_perfect match probe
vvi|miR-1329|2mut|as|	gccaacaataactccctgtc	vvi-miR169o|*0_mismatch probe
vvi|miR-1330|nat|as|	ggccgcatgagccaagaata	vvi-miR169o|*+10_perfect match probe
vvi|miR-1330|2mut|as|	ggccccatgagtcaagaata	vvi-miR169o|*+10_mismatch probe
vvi|miR-1331|nat|as|	tgcttgccagttatagccag	vvi-miR169p|*-10_perfect match probe
vvi|miR-1331|2mut|as|	tgcttgtcagttatacccag	vvi-miR169p|*-10_mismatch probe
vvi|miR-1332|nat|as|	gagcctcggatgcttgccag	vvi-miR169p|*0_perfect match probe
vvi|miR-1332|2mut|as|	gagcctcagatgcttcccag	vvi-miR169p|*0_mismatch probe
vvi|miR-1333|nat|as|	gggtgaaacagagcctcgga	vvi-miR169p|*+10_perfect match probe
vvi|miR-1333|2mut|as|	gggtgaaaaagagtctcgga	vvi-miR169p|*+10_mismatch probe
vvi|miR-1334|nat|as|	tacatgccggctgttccagt	vvi-miR169q|*-10_perfect match probe
vvi|miR-1334|2mut|as|	tacatgccgcttgttccagt	vvi-miR169q|*-10_mismatch probe
vvi|miR-1335|nat|as|	gagccaggattacatgccgg	vvi-miR169q|*0_perfect match probe
vvi|miR-1335|2mut|as|	gagcaaggattacatcccgg	vvi-miR169q|*0_mismatch probe
vvi|miR-1336|nat|as|	ggaccaaatagagccaggat	vvi-miR169q|*+10_perfect match probe
vvi|miR-1336|2mut|as|	ggacaaaatagagtcaggat	vvi-miR169q|*+10_mismatch probe
vvi|miR-1337|nat|as|	caacttgccggttacacata	vvi-miR169r|*-10_perfect match probe
vvi|miR-1337|2mut|as|	caacttgtcgcttacacata	vvi-miR169r|*-10_mismatch probe
vvi|miR-1338|nat|as|	tgagtcaagtcaacttgccg	vvi-miR169r|*0_perfect match probe

vvi|miR-1338|2mut|as|	tgagtcaagcaaacttgccg
vvi|miR-1339|nat|as|	agggccaaactgagtcaagt
vvi|miR-1339|2mut|as|	agggtcaaactgagccaagt
vvi|miR-1340|nat|as|	aacttgccgggaagatgatc
vvi|miR-1340|2mut|as|	aacttgtcggaaagatgatc
vvi|miR-1341|nat|as|	agccaggaacaacttgccgg
vvi|miR-1341|2mut|as|	agccagaaacaacttcccgg
vvi|miR-1342|nat|as|	agcagaatgtagccaggaac
vvi|miR-1342|2mut|as|	agcagaatctagtcaggaac
vvi|miR-1343|nat|as|	caacttgccggttacacata
vvi|miR-1343|2mut|as|	caacttgtcgcttacacata
vvi|miR-1344|nat|as|	tgagtcaagtcaacttgccg
vvi|miR-1344|2mut|as|	tgagtcaagcaaacttgccg
vvi|miR-1345|nat|as|	agggccaaactgagtcaagt
vvi|miR-1345|2mut|as|	agggtcaaactgagccaagt
vvi|miR-1346|nat|as|	caacttgccggtaacacata
vvi|miR-1346|2mut|as|	caacttgtcgctaacacata
vvi|miR-1347|nat|as|	agagtcaagtcaacttgccg
vvi|miR-1347|2mut|as|	agagtcaagcaaacttgccg
vvi|miR-1348|nat|as|	agggccaaacagagtcaagt
vvi|miR-1348|2mut|as|	agggtcaaacagagccaagt
vvi|miR-1349|nat|as|	aaattgccgatccatggaca
vvi|miR-1349|2mut|as|	aaattgtcgatccatcgaca
vvi|miR-1350|nat|as|	gccaagaaataaattgccga
vvi|miR-1350|2mut|as|	gccaacaaataaattcccga
vvi|miR-1351|nat|as|	gcccaacatagccaagaaat

vvi-miR169r|*0_mismatch probe
vvi-miR169r|*+10_perfect match probe
vvi-miR169r|*+10_mismatch probe
vvi-miR169s|*-10_perfect match probe
vvi-miR169s|*-10_mismatch probe
vvi-miR169s|*0_perfect match probe
vvi-miR169s|*0_mismatch probe
vvi-miR169s|*+10_perfect match probe
vvi-miR169s|*+10_mismatch probe
vvi-miR169t|*-10_perfect match probe
vvi-miR169t|*-10_mismatch probe
vvi-miR169t|*0_perfect match probe
vvi-miR169t|*0_mismatch probe
vvi-miR169t|*+10_perfect match probe
vvi-miR169t|*+10_mismatch probe
vvi-miR169u|*-10_perfect match probe
vvi-miR169u|*-10_mismatch probe
vvi-miR169u|*0_perfect match probe
vvi-miR169u|*0_mismatch probe
vvi-miR169u|*+10_perfect match probe
vvi-miR169u|*+10_mismatch probe
vvi-miR169v|*-10_perfect match probe
vvi-miR169v|*-10_mismatch probe
vvi-miR169v|*0_perfect match probe
vvi-miR169v|*0_mismatch probe
vvi-miR169v|*+10_perfect match

		probe
vvi|miR-1351|2mut|as|	gcccaaaatagtcaagaaat	vvi-miR169v|*+10_mismatch probe
		vvi-miR169w|*-10_perfect match
vvi|miR-1352|nat|as|	gactcgccgaccaccatttt	probe
vvi|miR-1352|2mut|as|	gactccccgaccacaatttt	vvi-miR169w|*-10_mismatch probe
vvi|miR-1353|nat|as|	agctaagaatgactcgccga	vvi-miR169w|*0_perfect match probe
vvi|miR-1353|2mut|as|	agctaacaatgactccccga	vvi-miR169w|*0_mismatch probe
		vvi-miR169w|*+10_perfect match
vvi|miR-1354|nat|as|	gcagaaatgtagctaagaat	probe
vvi|miR-1354|2mut|as|	gcagaaatctagttaagaat	vvi-miR169w|*+10_mismatch probe
		vvi-miR169x|*-10_perfect match
vvi|miR-1355|nat|as|	tgactgcctaaagaccacgg	probe
vvi|miR-1355|2mut|as|	tgactgtctaaagacaacgg	vvi-miR169x|*-10_mismatch probe
vvi|miR-1356|nat|as|	gccgaaggaatgactgccta	vvi-miR169x|*0_perfect match probe
vvi|miR-1356|2mut|as|	gccgaagaaatgactcccta	vvi-miR169x|*0_mismatch probe
		vvi-miR169x|*+10_perfect match
vvi|miR-1357|nat|as|	ggtcagtttagccgaaggaa	probe
vvi|miR-1357|2mut|as|	ggtccgtttagtcgaaggaa	vvi-miR169x|*+10_mismatch probe
		vvi-miR171a|*-10_perfect match
vvi|miR-1358|nat|as|	caatatcatgttcaatac	probe
vvi|miR-1358|2mut|as|	caatataatcttcaatac	vvi-miR171a|*-10_mismatch probe
vvi|miR-1359|nat|as|	tgagccgtgccaatatcatg	vvi-miR171a|*0_perfect match probe
vvi|miR-1359|2mut|as|	tgagccgtgtaaatatcatg	vvi-miR171a|*0_mismatch probe
		vvi-miR171a|*+10_perfect match
vvi|miR-1360|nat|as|	ttaatctgattgagccgtgc	probe
vvi|miR-1360|2mut|as|	ttaatccgattgagtcgtgc	vvi-miR171a|*+10_mismatch probe
		vvi-miR171b|*-10_perfect match
vvi|miR-1361|nat|as|	cgccaatacctccccccca	probe
vvi|miR-1361|2mut|as|	cgccaatatctcctccca	vvi-miR171b|*-10_mismatch probe
vvi|miR-1362|nat|as|	aattgaggcacgccaatacc	vvi-miR171b|*0_perfect match probe
vvi|miR-1362|2mut|as|	aattgagccaccccaatacc	vvi-miR171b|*0_mismatch probe
		vvi-miR171b|*+10_perfect match
vvi|miR-1363|nat|as|	catgtctttaaattgaggca	probe
vvi|miR-1363|2mut|as|	catgcctttaaattaaggca	vvi-miR171b|*+10_mismatch probe

vvi|miR-1364|nat|as|	caatatcccgtaatacca	vvi-miR171c|*-10_perfect match probe
vvi|miR-1364|2mut|as|	caatatccactaatacca	vvi-miR171c|*-10_mismatch probe
vvi|miR-1365|nat|as|	tgaaccgcaccaatatcccg	vvi-miR171c|*0_perfect match probe
vvi|miR-1365|2mut|as|	tgaaccccacaaatatcccg	vvi-miR171c|*0_mismatch probe
vvi|miR-1366|nat|as|	gctttcttattgaaccgcac	vvi-miR171c|*+10_perfect match probe
vvi|miR-1366|2mut|as|	gcttttttattgaacagcac	vvi-miR171c|*+10_mismatch probe
vvi|miR-1367|nat|as|	caatatctcgtgtatcta	vvi-miR171d|*-10_perfect match probe
vvi|miR-1367|2mut|as|	caatatctactgtatcta	vvi-miR171d|*-10_mismatch probe
vvi|miR-1368|nat|as|	tgaaccgtatcaatatctcg	vvi-miR171d|*0_perfect match probe
vvi|miR-1368|2mut|as|	tgaacagtataaatatctcg	vvi-miR171d|*0_mismatch probe
vvi|miR-1369|nat|as|	gctttctaattgaaccgtat	vvi-miR171d|*+10_perfect match probe
vvi|miR-1369|2mut|as|	gctttttaattgaacagtat	vvi-miR171d|*+10_mismatch probe
vvi|miR-1370|nat|as|	caccaacatcgcttactttc	vvi-miR171e|*-10_perfect match probe
vvi|miR-1370|2mut|as|	caccaacataccttactttc	vvi-miR171e|*-10_mismatch probe
vvi|miR-1371|nat|as|	gattgaacctcaccaacatc	vvi-miR171e|*0_perfect match probe
vvi|miR-1371|2mut|as|	gattgaatctcacaaacatc	vvi-miR171e|*0_mismatch probe
vvi|miR-1372|nat|as|	tccgtcttcggattgaacct	vvi-miR171e|*+10_perfect match probe
vvi|miR-1372|2mut|as|	tccgtcttcaaattgaacct	vvi-miR171e|*+10_mismatch probe
vvi|miR-1373|nat|as|	caacatcgctttcttcaa	vvi-miR171f|*-10_perfect match probe
vvi|miR-1373|2mut|as|	caacatcccttttttcaa	vvi-miR171f|*-10_mismatch probe
vvi|miR-1374|nat|as|	tgaacctcaccaacatcgct	vvi-miR171f|*0_perfect match probe
vvi|miR-1374|2mut|as|	tgaacctcataaacatcgct	vvi-miR171f|*0_mismatch probe
vvi|miR-1375|nat|as|	atcttcagattgaacctcac	vvi-miR171f|*+10_perfect match probe
vvi|miR-1375|2mut|as|	atcttccgattgaatctcac	vvi-miR171f|*+10_mismatch probe
vvi|miR-1376|nat|as|	gaaccaacatggagaggctg	vvi-miR171g|*-10_perfect match probe

<table>
<tr><td>vvi|miR-1376|2mut|as|</td><td>gaacaaacatcgagaggctg</td><td>vvi-miR171g|*-10_mismatch probe</td></tr>
<tr><td>vvi|miR-1377|nat|as|</td><td>cccaccgatggaaccaacat</td><td>vvi-miR171g|*0_perfect match probe</td></tr>
<tr><td>vvi|miR-1377|2mut|as|</td><td>cccacagatggaacaaacat</td><td>vvi-miR171g|*0_mismatch probe</td></tr>
<tr><td>vvi|miR-1378|nat|as|</td><td>gttggtgtcccccaccgatg</td><td>vvi-miR171g|*+10_perfect match probe</td></tr>
<tr><td>vvi|miR-1378|2mut|as|</td><td>gttggtgtctcccacagatg</td><td>vvi-miR171g|*+10_mismatch probe</td></tr>
<tr><td>vvi|miR-1379|nat|as|</td><td>caccaacatctcgtgaaa</td><td>vvi-miR171h|*-10_perfect match probe</td></tr>
<tr><td>vvi|miR-1379|2mut|as|</td><td>caccaaaatctcctgaaa</td><td>vvi-miR171h|*-10_mismatch probe</td></tr>
<tr><td>vvi|miR-1380|nat|as|</td><td>ggttgaaccgcaccaacatc</td><td>vvi-miR171h|*0_perfect match probe</td></tr>
<tr><td>vvi|miR-1380|2mut|as|</td><td>ggttgaacccaaccaacatc</td><td>vvi-miR171h|*0_mismatch probe</td></tr>
<tr><td>vvi|miR-1381|nat|as|</td><td>actacagttaggttgaaccg</td><td>vvi-miR171h|*+10_perfect match probe</td></tr>
<tr><td>vvi|miR-1381|2mut|as|</td><td>actaaagttagcttgaaccg</td><td>vvi-miR171h|*+10_mismatch probe</td></tr>
<tr><td>vvi|miR-1382|nat|as|</td><td>caacatccccaccttcttc</td><td>vvi-miR171i|*-10_perfect match probe</td></tr>
<tr><td>vvi|miR-1382|2mut|as|</td><td>caacatcctcatcttcttc</td><td>vvi-miR171i|*-10_mismatch probe</td></tr>
<tr><td>vvi|miR-1383|nat|as|</td><td>tgagccattccaacatcccc</td><td>vvi-miR171i|*0_perfect match probe</td></tr>
<tr><td>vvi|miR-1383|2mut|as|</td><td>tgagtcattcaaacatcccc</td><td>vvi-miR171i|*0_mismatch probe</td></tr>
<tr><td>vvi|miR-1384|nat|as|</td><td>ttgatttgattgagccattc</td><td>vvi-miR171i|*+10_perfect match probe</td></tr>
<tr><td>vvi|miR-1384|2mut|as|</td><td>ttgatttaattgagtcattc</td><td>vvi-miR171i|*+10_mismatch probe</td></tr>
<tr><td>vvi|miR-1385|nat|as|</td><td>gatgctgcatcggcaata</td><td>vvi-miR172a|*-10_perfect match probe</td></tr>
<tr><td>vvi|miR-1385|2mut|as|</td><td>gatgctgaatcgccaata</td><td>vvi-miR172a|*-10_mismatch probe</td></tr>
<tr><td>vvi|miR-1386|nat|as|</td><td>gaatcttgatgatgctgcat</td><td>vvi-miR172a|*0_perfect match probe</td></tr>
<tr><td>vvi|miR-1386|2mut|as|</td><td>gaatcttaatgatcctgcat</td><td>vvi-miR172a|*0_mismatch probe</td></tr>
<tr><td>vvi|miR-1387|nat|as|</td><td>tcaaggatgagaatcttgat</td><td>vvi-miR172a|*+10_perfect match probe</td></tr>
<tr><td>vvi|miR-1387|2mut|as|</td><td>tcaagaatgagaattttgat</td><td>vvi-miR172a|*+10_mismatch probe</td></tr>
<tr><td>vvi|miR-1388|nat|as|</td><td>tgatgctgcatcggcaata</td><td>vvi-miR172b|*-10_perfect match probe</td></tr>
<tr><td>vvi|miR-1388|2mut|as|</td><td>tgatgctgaatcgccaata</td><td>vvi-miR172b|*-10_mismatch probe</td></tr>
<tr><td>vvi|miR-1389|nat|as|</td><td>agaatcttgatgatgctgca</td><td>vvi-miR172b|*0_perfect match probe</td></tr>
</table>

vvi|miR-1389|2mut|as|	agaatcttaatgatcctgca
vvi|miR-1390|nat|as|	tttggggttgagaatcttga
vvi|miR-1390|2mut|as|	tttggtgttgcgaatcttga
vvi|miR-1391|nat|as|	tgatgctccatccgcaaac
vvi|miR-1391|2mut|as|	tgatcctccatccccaaac
vvi|miR-1392|nat|as|	tgaatcttgatgatgctcca
vvi|miR-1392|2mut|as|	tgaatcttaatgatcctcca
vvi|miR-1393|nat|as|	tcaatacttgtgaatcttga
vvi|miR-1393|2mut|as|	tcaatacttccgaatcttga
vvi|miR-1394|nat|as|	gatgctgcatcagctaac
vvi|miR-1394|2mut|as|	gatgctgaatcagttaac
vvi|miR-1395|nat|as|	gaatcttgatgatgctgcat
vvi|miR-1395|2mut|as|	gaatcttaatgatcctgcat
vvi|miR-1396|nat|as|	cattgggtgtgaatcttgat
vvi|miR-1396|2mut|as|	cattgtgtgtaaatcttgat
vvi|miR-1397|nat|as|	agagagctccttcttccaca
vvi|miR-1397|2mut|as|	agagagttccttctttcaca
vvi|miR-1398|nat|as|	actggactaaagagagctcc
vvi|miR-1398|2mut|as|	actgaactaaagagacctcc
vvi|miR-1399|nat|as|	gctgtctcggactggactaa
vvi|miR-1399|2mut|as|	gctgtctcagactcgactaa
vvi|miR-1400|nat|as|	gaaagctctcttcaatgtaa
vvi|miR-1400|2mut|as|	gaaagttctcttaaatgtaa
vvi|miR-1401|nat|as|	gtggactgaagaaagctctc
vvi|miR-1401|2mut|as|	gtggcctgaagaaagttctc
vvi|miR-1402|nat|as|	ccacccatgagtggactgaa

vvi-miR172b|*0_mismatch probe
vvi-miR172b|*+10_perfect match probe
vvi-miR172b|*+10_mismatch probe
vvi-miR172c|*-10_perfect match probe
vvi-miR172c|*-10_mismatch probe
vvi-miR172c|*0_perfect match probe
vvi-miR172c|*0_mismatch probe
vvi-miR172c|*+10_perfect match probe
vvi-miR172c|*+10_mismatch probe
vvi-miR172d|*-10_perfect match probe
vvi-miR172d|*-10_mismatch probe
vvi-miR172d|*0_perfect match probe
vvi-miR172d|*0_mismatch probe
vvi-miR172d|*+10_perfect match probe
vvi-miR172d|*+10_mismatch probe
vvi-miR319b|*-10_perfect match probe
vvi-miR319b|*-10_mismatch probe
vvi-miR319b|*0_perfect match probe
vvi-miR319b|*0_mismatch probe
vvi-miR319b|*+10_perfect match probe
vvi-miR319b|*+10_mismatch probe
vvi-miR319c|*-10_perfect match probe
vvi-miR319c|*-10_mismatch probe
vvi-miR319c|*0_perfect match probe
vvi-miR319c|*0_mismatch probe
vvi-miR319c|*+10_perfect match

		probe
vvi|miR-1402|2mut|as|	ccactcatgagtcgactgaa	vvi-miR319c|*+10_mismatch probe
vvi|miR-1403|nat|as|	aggaacccccatttctgca	vvi-miR319e|*-10_perfect match probe
vvi|miR-1403|2mut|as|	aggaaccctcatttttgca	vvi-miR319e|*-10_mismatch probe
vvi|miR-1404|nat|as|	tgggctgcaaaggaaccccc	vvi-miR319e|*0_perfect match probe
vvi|miR-1404|2mut|as|	tgggctgaaaaggaatcccc	vvi-miR319e|*0_mismatch probe
vvi|miR-1405|nat|as|	ggagttgttttgggctgcaa	vvi-miR319e|*+10_perfect match probe
vvi|miR-1405|2mut|as|	ggagttcttttggcctgcaa	vvi-miR319e|*+10_mismatch probe
vvi|miR-1406|nat|as|	ggaagctctcttaaaccact	vvi-miR319f|*-10_perfect match probe
vvi|miR-1406|2mut|as|	ggaagttctcttaaatcact	vvi-miR319f|*-10_mismatch probe
vvi|miR-1407|nat|as|	gtggactgaaggaagctctc	vvi-miR319f|*0_perfect match probe
vvi|miR-1407|2mut|as|	gtggcctgaaggaagttctc	vvi-miR319f|*0_mismatch probe
vvi|miR-1408|nat|as|	ccatccatgagtggactgaa	vvi-miR319f|*+10_perfect match probe
vvi|miR-1408|2mut|as|	ccatcaatgagtcgactgaa	vvi-miR319f|*+10_mismatch probe
vvi|miR-1409|nat|as|	aaaggagctcttcagctgtt	vvi-miR319g|*-10_perfect match probe
vvi|miR-1409|2mut|as|	aaaggagttcttcagttgtt	vvi-miR319g|*-10_mismatch probe
vvi|miR-1410|nat|as|	tattggactaaaaggagctc	vvi-miR319g|*0_perfect match probe
vvi|miR-1410|2mut|as|	tattcgactaaaagaagctc	vvi-miR319g|*0_mismatch probe
vvi|miR-1411|nat|as|	cagccctctttattggacta	vvi-miR319g|*+10_perfect match probe
vvi|miR-1411|2mut|as|	cagctctctttattcgacta	vvi-miR319g|*+10_mismatch probe
vvi|miR-1412|nat|as|	agatagcgccacacacacaa	vvi-miR390|*-10_perfect match probe
vvi|miR-1412|2mut|as|	agatagccccacacccacaa	vvi-miR390|*-10_mismatch probe
vvi|miR-1413|nat|as|	aactcaggatagatagcgcc	vvi-miR390|*0_perfect match probe
vvi|miR-1413|2mut|as|	aactcagaatagataccgcc	vvi-miR390|*0_mismatch probe
vvi|miR-1414|nat|as|	gaacccgtgaaactcaggat	vvi-miR390|*+10_perfect match probe
vvi|miR-1414|2mut|as|	gaaccagtgaaactccggat	vvi-miR390|*+10_mismatch probe

<table>
<tr><td>vvi|miR-1415|nat|as|</td><td>agcatgatccaatgggaaaa</td><td>vvi-miR393a|*-10_perfect match probe</td></tr>
<tr><td>vvi|miR-1415|2mut|as|</td><td>agcatgatcaaatgtgaaaa</td><td>vvi-miR393a|*-10_mismatch probe</td></tr>
<tr><td>vvi|miR-1416|nat|as|</td><td>cctaagggatagcatgatcc</td><td>vvi-miR393a|*0_perfect match probe</td></tr>
<tr><td>vvi|miR-1416|2mut|as|</td><td>cctaagtgatagaatgatcc</td><td>vvi-miR393a|*0_mismatch probe</td></tr>
<tr><td>vvi|miR-1417|nat|as|</td><td>atggagagttcctaagggat</td><td>vvi-miR393a|*+10_perfect match probe</td></tr>
<tr><td>vvi|miR-1417|2mut|as|</td><td>atggagactttctaagggat</td><td>vvi-miR393a|*+10_mismatch probe</td></tr>
<tr><td>vvi|miR-1418|nat|as|</td><td>agcatgatccaaagacatgg</td><td>vvi-miR393b|*-10_perfect match probe</td></tr>
<tr><td>vvi|miR-1418|2mut|as|</td><td>agcatgatcaaaagaaatgg</td><td>vvi-miR393b|*-10_mismatch probe</td></tr>
<tr><td>vvi|miR-1419|nat|as|</td><td>ccaaagggatagcatgatcc</td><td>vvi-miR393b|*0_perfect match probe</td></tr>
<tr><td>vvi|miR-1419|2mut|as|</td><td>ccaaagtgatagaatgatcc</td><td>vvi-miR393b|*0_mismatch probe</td></tr>
<tr><td>vvi|miR-1420|nat|as|</td><td>aaggaggaatccaaagggat</td><td>vvi-miR393b|*+10_perfect match probe</td></tr>
<tr><td>vvi|miR-1420|2mut|as|</td><td>aaggagaaatcaaaagggat</td><td>vvi-miR393b|*+10_mismatch probe</td></tr>
<tr><td>vvi|miR-1421|nat|as|</td><td>ggccacctccaaaagaatat</td><td>vvi-miR394a|*-10_perfect match probe</td></tr>
<tr><td>vvi|miR-1421|2mut|as|</td><td>ggccacctcaaaaacaatat</td><td>vvi-miR394a|*-10_mismatch probe</td></tr>
<tr><td>vvi|miR-1422|nat|as|</td><td>cagagccactttggcatgct</td><td>vvi-miR394a|*+10_perfect match probe</td></tr>
<tr><td>vvi|miR-1422|2mut|as|</td><td>cagagtcactttgccatgct</td><td>vvi-miR394a|*+10_mismatch probe</td></tr>
<tr><td>vvi|miR-1423|nat|as|</td><td>gcccacctccacagagagaa</td><td>vvi-miR394b|*-10_perfect match probe</td></tr>
<tr><td>vvi|miR-1423|2mut|as|</td><td>gcccacctcaccagagagaa</td><td>vvi-miR394b|*-10_mismatch probe</td></tr>
<tr><td>vvi|miR-1424|nat|as|</td><td>cagagcttggttggcagtat</td><td>vvi-miR394b|*+10_perfect match probe</td></tr>
<tr><td>vvi|miR-1424|2mut|as|</td><td>cagagtttggttgccagtat</td><td>vvi-miR394b|*+10_mismatch probe</td></tr>
<tr><td>vvi|miR-1425|nat|as|</td><td>ggccgcctccaaacgcttgt</td><td>vvi-miR394c|*-10_perfect match probe</td></tr>
<tr><td>vvi|miR-1425|2mut|as|</td><td>ggccccctccaaacccttgt</td><td>vvi-miR394c|*-10_mismatch probe</td></tr>
<tr><td>vvi|miR-1426|nat|as|</td><td>cagagccaatttggcatcct</td><td>vvi-miR394c|*+10_perfect match probe</td></tr>
<tr><td>vvi|miR-1426|2mut|as|</td><td>cagagtcaatttgccatcct</td><td>vvi-miR394c|*+10_mismatch probe</td></tr>
</table>

vvi|miR-1427|nat|as|	aagggaactctaggggac	vvi-miR395a|*-10_perfect match probe
vvi|miR-1427|2mut|as|	aaggaaactctagtggac	vvi-miR395a|*-10_mismatch probe
vvi|miR-1428|nat|as|	tgaagtgatcaagggaactc	vvi-miR395a|*0_perfect match probe
vvi|miR-1428|2mut|as|	tgaagcgatcaagtgaactc	vvi-miR395a|*0_mismatch probe
vvi|miR-1429|nat|as|	agctccctagtgaagtgatc	vvi-miR395a|*+10_perfect match probe
vvi|miR-1429|2mut|as|	agctctctagtgaagcgatc	vvi-miR395a|*+10_mismatch probe
vvi|miR-1430|nat|as|	aggggaactctaggggac	vvi-miR395b|*-10_perfect match probe
vvi|miR-1430|2mut|as|	agggaaactctagtggac	vvi-miR395b|*-10_mismatch probe
vvi|miR-1431|nat|as|	tgaagtggtaaggggaactc	vvi-miR395b|*0_perfect match probe
vvi|miR-1431|2mut|as|	tgaagtcgtaaggtgaactc	vvi-miR395b|*0_mismatch probe
vvi|miR-1432|nat|as|	agatccccagtgaagtggta	vvi-miR395b|*+10_perfect match probe
vvi|miR-1432|2mut|as|	agatcctcagtgaagcggta	vvi-miR395b|*+10_mismatch probe
vvi|miR-1433|nat|as|	aagggaactctaggggac	vvi-miR395c|*-10_perfect match probe
vvi|miR-1433|2mut|as|	aaggaaactctagtggac	vvi-miR395c|*-10_mismatch probe
vvi|miR-1434|nat|as|	tgaagtggtcaagggaactc	vvi-miR395c|*0_perfect match probe
vvi|miR-1434|2mut|as|	tgaagtcgtcaagtgaactc	vvi-miR395c|*0_mismatch probe
vvi|miR-1435|nat|as|	aggtccccagtgaagtggtc	vvi-miR395c|*+10_perfect match probe
vvi|miR-1435|2mut|as|	aggtcctcagtgaagcggtc	vvi-miR395c|*+10_mismatch probe
vvi|miR-1436|nat|as|	aggggaactctagggggc	vvi-miR395d|*-10_perfect match probe
vvi|miR-1436|2mut|as|	agggaaactctagtgggc	vvi-miR395d|*-10_mismatch probe
vvi|miR-1437|nat|as|	tgaagtggtcaggggaactc	vvi-miR395d|*0_perfect match probe
vvi|miR-1437|2mut|as|	tgaagtcgtcaggtgaactc	vvi-miR395d|*0_mismatch probe
vvi|miR-1438|nat|as|	agatccccaatgaagtggtc	vvi-miR395d|*+10_perfect match probe
vvi|miR-1438|2mut|as|	agatcctcaatgaagcggtc	vvi-miR395d|*+10_mismatch probe
vvi|miR-1439|nat|as|	aagggaactctaggggag	vvi-miR395e|*-10_perfect match probe

vvi|miR-1439|2mut|as|	aaggaaactctagtggag	vvi-miR395e|*-10_mismatch probe
vvi|miR-1440|nat|as|	tgaagtggtcaagggaactc	vvi-miR395e|*0_perfect match probe
vvi|miR-1440|2mut|as|	tgaagtcgtcaagtgaactc	vvi-miR395e|*0_mismatch probe
vvi|miR-1441|nat|as|	aggtccccagtgaagtggtc	vvi-miR395e|*+10_perfect match probe
vvi|miR-1441|2mut|as|	aggtcctcagtgaagcggtc	vvi-miR395e|*+10_mismatch probe
vvi|miR-1442|nat|as|	aggggaactctagggtac	vvi-miR395f|*-10_perfect match probe
vvi|miR-1442|2mut|as|	agggaaactctagtgtac	vvi-miR395f|*-10_mismatch probe
vvi|miR-1443|nat|as|	tgaagtggtcaggggaactc	vvi-miR395f|*0_perfect match probe
vvi|miR-1443|2mut|as|	tgaagtcgtcaggtgaactc	vvi-miR395f|*0_mismatch probe
vvi|miR-1444|nat|as|	agatccccagtgaagtggtc	vvi-miR395f|*+10_perfect match probe
vvi|miR-1444|2mut|as|	agatcctcagtgaagcggtc	vvi-miR395f|*+10_mismatch probe
vvi|miR-1445|nat|as|	aggggaactctaggggac	vvi-miR395g|*-10_perfect match probe
vvi|miR-1445|2mut|as|	agggaaactctagtggac	vvi-miR395g|*-10_mismatch probe
vvi|miR-1446|nat|as|	tgaagtgctcaggggaactc	vvi-miR395g|*0_perfect match probe
vvi|miR-1446|2mut|as|	tgaagtcctcaggtgaactc	vvi-miR395g|*0_mismatch probe
vvi|miR-1447|nat|as|	ggatccccaatgaagtgctc	vvi-miR395g|*+10_perfect match probe
vvi|miR-1447|2mut|as|	ggatcctcaatgaagcgctc	vvi-miR395g|*+10_mismatch probe
vvi|miR-1448|nat|as|	aagggaactctaggggac	vvi-miR395h|*-10_perfect match probe
vvi|miR-1448|2mut|as|	aaggaaactctagtggac	vvi-miR395h|*-10_mismatch probe
vvi|miR-1449|nat|as|	tgaagtggtcaagggaactc	vvi-miR395h|*0_perfect match probe
vvi|miR-1449|2mut|as|	tgaagtcgtcaagtgaactc	vvi-miR395h|*0_mismatch probe
vvi|miR-1450|nat|as|	aggtccccagtgaagtggtc	vvi-miR395h|*+10_perfect match probe
vvi|miR-1450|2mut|as|	aggtcctcagtgaagcggtc	vvi-miR395h|*+10_mismatch probe
vvi|miR-1451|nat|as|	aggggaactctagggggc	vvi-miR395i|*-10_perfect match probe
vvi|miR-1451|2mut|as|	agggaaactctagtgggc	vvi-miR395i|*-10_mismatch probe
vvi|miR-1452|nat|as|	tgaagtggtcaggggaactc	vvi-miR395i|*0_perfect match probe

vvi|miR-1452|2mut|as|	tgaagtcgtcaggtgaactc	vvi-miR395i|*0_mismatch probe
		vvi-miR395i|*+10_perfect match probe
vvi|miR-1453|nat|as|	agatccccagtgaagtggtc	
vvi|miR-1453|2mut|as|	agatcctcagtgaagcggtc	vvi-miR395i|*+10_mismatch probe
		vvi-miR395j|*-10_perfect match probe
vvi|miR-1454|nat|as|	aggggaactctagggggc	
vvi|miR-1454|2mut|as|	agggaaactctagtgggc	vvi-miR395j|*-10_mismatch probe
vvi|miR-1455|nat|as|	tgaagtggtcaggggaactc	vvi-miR395j|*0_perfect match probe
vvi|miR-1455|2mut|as|	tgaagtcgtcaggtgaactc	vvi-miR395j|*0_mismatch probe
		vvi-miR395j|*+10_perfect match probe
vvi|miR-1456|nat|as|	agatccccagtgaagtggtc	
vvi|miR-1456|2mut|as|	agatcctcagtgaagcggtc	vvi-miR395j|*+10_mismatch probe
		vvi-miR395k|*-10_perfect match probe
vvi|miR-1457|nat|as|	aagggaactctaggggga	
vvi|miR-1457|2mut|as|	aaggaaactctacggga	vvi-miR395k|*-10_mismatch probe
vvi|miR-1458|nat|as|	tgaagtggtcaagggaactc	vvi-miR395k|*0_perfect match probe
vvi|miR-1458|2mut|as|	tgaagtcgtcaagtgaactc	vvi-miR395k|*0_mismatch probe
		vvi-miR395k|*+10_perfect match probe
vvi|miR-1459|nat|as|	aggtccccagtgaagtggtc	
vvi|miR-1459|2mut|as|	aggtcctcagtgaagcggtc	vvi-miR395k|*+10_mismatch probe
		vvi-miR395l|*-10_perfect match probe
vvi|miR-1460|nat|as|	aggggaactctagggggc	
vvi|miR-1460|2mut|as|	agggaaactctagtgggc	vvi-miR395l|*-10_mismatch probe
vvi|miR-1461|nat|as|	tgaagtggtcaggggaactc	vvi-miR395l|*0_perfect match probe
vvi|miR-1461|2mut|as|	tgaagtcgtcaggtgaactc	vvi-miR395l|*0_mismatch probe
		vvi-miR395l|*+10_perfect match probe
vvi|miR-1462|nat|as|	gatcccccagtgaagtggtc	
vvi|miR-1462|2mut|as|	gatccctcagtgaagcggtc	vvi-miR395l|*+10_mismatch probe
		vvi-miR395m|*-10_perfect match probe
vvi|miR-1463|nat|as|	aagggaactctaggggac	
vvi|miR-1463|2mut|as|	aaggaaactctagtggac	vvi-miR395m|*-10_mismatch probe
		vvi-miR395m|*0_perfect match probe
vvi|miR-1464|nat|as|	tgaagtgttcaagggaactc	
vvi|miR-1464|2mut|as|	tgaagcgttcaagtgaactc	vvi-miR395m|*0_mismatch probe

vvi|miR-1465|nat|as|	aggtccccagtgaagtgttc	vvi-miR395m|*+10_perfect match probe
vvi|miR-1465|2mut|as|	aggtcctcagtgaagcgttc	vvi-miR395m|*+10_mismatch probe
vvi|miR-1466|nat|as|	gggggaactctcaaggcc	vvi-miR395n|*-10_perfect match probe
vvi|miR-1466|2mut|as|	ggggaaactctaaaggcc	vvi-miR395n|*-10_mismatch probe
vvi|miR-1467|nat|as|	tggaggggttgggggaactc	vvi-miR395n|*0_perfect match probe
vvi|miR-1467|2mut|as|	tggaggtgttgggtgaactc	vvi-miR395n|*0_mismatch probe
vvi|miR-1468|nat|as|	atggtcatactggaggggtt	vvi-miR395n|*+10_perfect match probe
vvi|miR-1468|2mut|as|	atggccatactggagtggtt	vvi-miR395n|*+10_mismatch probe
vvi|miR-1469|nat|as|	ttcttgaactacttttccat	vvi-miR396a|*-10_perfect match probe
vvi|miR-1469|2mut|as|	ttcttaaactatttttccat	vvi-miR396a|*-10_mismatch probe
vvi|miR-1470|nat|as|	ttccacagctttcttgaact	vvi-miR396a|*0_perfect match probe
vvi|miR-1470|2mut|as|	ttccacagttttcttaaact	vvi-miR396a|*0_mismatch probe
vvi|miR-1471|nat|as|	tgtcatgcttttccacagct	vvi-miR396a|*+10_perfect match probe
vvi|miR-1471|2mut|as|	tgtcatccttttcaacagct	vvi-miR396a|*+10_mismatch probe
vvi|miR-1472|nat|as|	tcttgaacttcttctccctg	vvi-miR396b|*-10_perfect match probe
vvi|miR-1472|2mut|as|	tcttaaacttcttcttcctg	vvi-miR396b|*-10_mismatch probe
vvi|miR-1473|nat|as|	tccacagctttcttgaactt	vvi-miR396b|*0_perfect match probe
vvi|miR-1473|2mut|as|	tccacagttttcttaaactt	vvi-miR396b|*0_mismatch probe
vvi|miR-1474|nat|as|	gccatgcttttccacagctt	vvi-miR396b|*+10_perfect match probe
vvi|miR-1474|2mut|as|	gccatccttttcaacagctt	vvi-miR396b|*+10_mismatch probe
vvi|miR-1475|nat|as|	tcctgagctgttggttgaac	vvi-miR396c|*-10_perfect match probe
vvi|miR-1475|2mut|as|	tcctgagttgttcgttgaac	vvi-miR396c|*-10_mismatch probe
vvi|miR-1476|nat|as|	tccacaactttcctgagctg	vvi-miR396c|*0_perfect match probe
vvi|miR-1476|2mut|as|	tccaaaacttttctgagctg	vvi-miR396c|*0_mismatch probe
vvi|miR-1477|nat|as|	tcagtaatgttccacaactt	vvi-miR396c|*+10_perfect match probe

vvi|miR-1477|2mut|as|	tcagtaatcttcaacaactt
vvi|miR-1478|nat|as|	tattgaaccgcaactgttac
vvi|miR-1478|2mut|as|	tattgaacccaaactgttac
vvi|miR-1479|nat|as|	cccacagctttattgaaccg
vvi|miR-1479|2mut|as|	cccacagttttattaaaccg
vvi|miR-1480|nat|as|	ttggtatcttcccacagctt
vvi|miR-1480|2mut|as|	ttggtattttctcacagctt
vvi|miR-1481|nat|as|	agcgccaatggaatcaacca
vvi|miR-1481|2mut|as|	agcgtcaatgaaatcaacca
vvi|miR-1482|nat|as|	gattgagtgcagcgccaatg
vvi|miR-1482|2mut|as|	gattgagtgaagccccaatg
vvi|miR-1483|nat|as|	gacatgattgagtgc
vvi|miR-1483|2mut|as|	gacataattaagtgc
vvi|miR-1484|nat|as|	agcgccaatggaatcaacca
vvi|miR-1484|2mut|as|	agcgtcaatgaaatcaacca
vvi|miR-1485|nat|as|	gattgagtgcagcgccaatg
vvi|miR-1485|2mut|as|	gattgagtgaagccccaatg
vvi|miR-1486|nat|as|	gacatgattgagtgc
vvi|miR-1486|2mut|as|	gacataattaagtgc
vvi|miR-1487|nat|as|	gccactcccttggggtgt
vvi|miR-1487|2mut|as|	gccactctcttggtgtgt
vvi|miR-1488|nat|as|	gttctcaggtgccactccct
vvi|miR-1488|2mut|as|	gttctcagctgtcactccct
vvi|miR-1489|nat|as|	cacaaccggtgttctcaggt
vvi|miR-1489|2mut|as|	cacaaccagtcttctcaggt
vvi|miR-1490|nat|as|	gtcacacctgtaggacac

vvi-miR396c|*+10_mismatch probe
vvi-miR396d|*-10_perfect match probe
vvi-miR396d|*-10_mismatch probe
vvi-miR396d|*0_perfect match probe
vvi-miR396d|*0_mismatch probe
vvi-miR396d|*+10_perfect match probe
vvi-miR396d|*+10_mismatch probe
vvi-miR397a|*-10_perfect match probe
vvi-miR397a|*-10_mismatch probe
vvi-miR397a|*0_perfect match probe
vvi-miR397a|*0_mismatch probe
vvi-miR397a|*+10_perfect match probe
vvi-miR397a|*+10_mismatch probe
vvi-miR397b|*-10_perfect match probe
vvi-miR397b|*-10_mismatch probe
vvi-miR397b|*0_perfect match probe
vvi-miR397b|*0_mismatch probe
vvi-miR397b|*+10_perfect match probe
vvi-miR397b|*+10_mismatch probe
vvi-miR398a|*-10_perfect match probe
vvi-miR398a|*-10_mismatch probe
vvi-miR398a|*0_perfect match probe
vvi-miR398a|*0_mismatch probe
vvi-miR398a|*+10_perfect match probe
vvi-miR398a|*+10_mismatch probe
vvi-miR398b|*-10_perfect match

		probe
vvi|miR-1490|2mut|as|	gtcacatctgtagaacac	vvi-miR398b|*-10_mismatch probe
vvi|miR-1491|nat|as|	tgattctcaggtcacacctg	vvi-miR398b|*0_perfect match probe
vvi|miR-1491|2mut|as|	tgattctcacctcacacctg	vvi-miR398b|*0_mismatch probe
		vvi-miR398b|*+10_perfect match
vvi|miR-1492|nat|as|	tgcgggcatgtgattctcag	probe
vvi|miR-1492|2mut|as|	tgcggccatgcgattctcag	vvi-miR398b|*+10_mismatch probe
		vvi-miR398c|*-10_perfect match
vvi|miR-1493|nat|as|	gtcactcctgtaggacac	probe
vvi|miR-1493|2mut|as|	gtcacttctgtagaacac	vvi-miR398c|*-10_mismatch probe
vvi|miR-1494|nat|as|	tgattctcaggtcactcctg	vvi-miR398c|*0_perfect match probe
vvi|miR-1494|2mut|as|	tgattctcacctcactcctg	vvi-miR398c|*0_mismatch probe
		vvi-miR398c|*+10_perfect match
vvi|miR-1495|nat|as|	tgcgggcatgtgattctcag	probe
vvi|miR-1495|2mut|as|	tgcggccatgcgattctcag	vvi-miR398c|*+10_mismatch probe
		vvi-miR399a|*-10_perfect match
vvi|miR-1496|nat|as|	aatcacactgttattctc	probe
vvi|miR-1496|2mut|as|	aatcaccctcttattctc	vvi-miR399a|*-10_mismatch probe
vvi|miR-1497|nat|as|	gccaaaggagaatcacactg	vvi-miR399a|*0_perfect match probe
vvi|miR-1497|2mut|as|	gccaaagaagaatcccactg	vvi-miR399a|*0_mismatch probe
		vvi-miR399a|*+10_perfect match
vvi|miR-1498|nat|as|	tgatatctctgccaaaggag	probe
vvi|miR-1498|2mut|as|	tgatatctccgtcaaaggag	vvi-miR399a|*+10_mismatch probe
		vvi-miR399b|*-10_perfect match
vvi|miR-1499|nat|as|	aggtgccctatgattgat	probe
vvi|miR-1499|2mut|as|	aggtgtcctataattgat	vvi-miR399b|*-10_mismatch probe
vvi|miR-1500|nat|as|	gccaagaaagaggtgcccta	vvi-miR399b|*0_perfect match probe
vvi|miR-1500|2mut|as|	gccaagaaacaggtgtccta	vvi-miR399b|*0_mismatch probe
		vvi-miR399b|*+10_perfect match
vvi|miR-1501|nat|as|	gccagtgcctgccaagaaag	probe
vvi|miR-1501|2mut|as|	gccagtgtctgtcaagaaag	vvi-miR399b|*+10_mismatch probe
		vvi-miR399c|*-10_perfect match
vvi|miR-1502|nat|as|	agaggccctgagaccggt	probe
vvi|miR-1502|2mut|as|	agaggtcctgagatcggt	vvi-miR399c|*-10_mismatch probe

vvi|miR-1503|nat|as|	accaagtgaaagaggccctg
vvi|miR-1503|2mut|as|	accaagcgaaagaggtcctg
vvi|miR-1504|nat|as|	tatgtcacctaccaagtgaa
vvi|miR-1504|2mut|as|	tatgtcatctacaaagtgaa
vvi|miR-1505|nat|as|	aaatctgctctataatttac
vvi|miR-1505|2mut|as|	aaatctcctccataatttac
vvi|miR-1506|nat|as|	ctgccaaaagaaatctgctc
vvi|miR-1506|2mut|as|	ctgcaaaaagaaatccgctc
vvi|miR-1507|nat|as|	tgatcgccatctgccaaaag
vvi|miR-1507|2mut|as|	tgatccccatctgtcaaaag
vvi|miR-1508|nat|as|	taatttgccctgtaatatgc
vvi|miR-1508|2mut|as|	taatttgtcctctaatatgc
vvi|miR-1509|nat|as|	tgccaaaagataatttgccc
vvi|miR-1509|2mut|as|	tgccaaaacataattcgccc
vvi|miR-1510|nat|as|	agtggctgcctgccaaaaga
vvi|miR-1510|2mut|as|	agtggctgtctgtcaaaaga
vvi|miR-1511|nat|as|	ccctctaatgcaac
vvi|miR-1511|2mut|as|	ccctttaatccaac
vvi|miR-1512|nat|as|	gcagatttggccctctaatg
vvi|miR-1512|2mut|as|	gcagatttgctcctctaatg
vvi|miR-1513|nat|as|	tcatgccaaagcagatttgg
vvi|miR-1513|2mut|as|	tcatgtcaaagaagatttgg
vvi|miR-1514|nat|as|	agtattgcccagcaattcat
vvi|miR-1514|2mut|as|	agtattgtccagaaattcat
vvi|miR-1515|nat|as|	ctgccaatggagtattgccc
vvi|miR-1515|2mut|as|	ctgccaatcgactattgccc

vvi-miR399c|*0_perfect match probe
vvi-miR399c|*0_mismatch probe
vvi-miR399c|*+10_perfect match probe
vvi-miR399c|*+10_mismatch probe
vvi-miR399d|*-10_perfect match probe
vvi-miR399d|*-10_mismatch probe
vvi-miR399d|*0_perfect match probe
vvi-miR399d|*0_mismatch probe
vvi-miR399d|*+10_perfect match probe
vvi-miR399d|*+10_mismatch probe
vvi-miR399e|*-10_perfect match probe
vvi-miR399e|*-10_mismatch probe
vvi-miR399e|*0_perfect match probe
vvi-miR399e|*0_mismatch probe
vvi-miR399e|*+10_perfect match probe
vvi-miR399e|*+10_mismatch probe
vvi-miR399f|*-10_perfect match probe
vvi-miR399f|*-10_mismatch probe
vvi-miR399f|*0_perfect match probe
vvi-miR399f|*0_mismatch probe
vvi-miR399f|*+10_perfect match probe
vvi-miR399f|*+10_mismatch probe
vvi-miR399g|*-10_perfect match probe
vvi-miR399g|*-10_mismatch probe
vvi-miR399g|*0_perfect match probe
vvi-miR399g|*0_mismatch probe

<table>
<tr><td>vvi|miR-1516|nat|as|</td><td>gagtggccaactgccaatgg</td><td>vvi-miR399g|*+10_perfect match probe</td></tr>
<tr><td>vvi|miR-1516|2mut|as|</td><td>gagtggtcaactgtcaatgg</td><td>vvi-miR399g|*+10_mismatch probe</td></tr>
<tr><td>vvi|miR-1517|nat|as|</td><td>gattgcactgttattcct</td><td>vvi-miR399h|*-10_perfect match probe</td></tr>
<tr><td>vvi|miR-1517|2mut|as|</td><td>gattgaactcttattcct</td><td>vvi-miR399h|*-10_mismatch probe</td></tr>
<tr><td>vvi|miR-1518|nat|as|</td><td>gccaaaggaggattgcactg</td><td>vvi-miR399h|*0_perfect match probe</td></tr>
<tr><td>vvi|miR-1518|2mut|as|</td><td>gccaaagaaggattgaactg</td><td>vvi-miR399h|*0_mismatch probe</td></tr>
<tr><td>vvi|miR-1519|nat|as|</td><td>tgatctttctgccaaaggag</td><td>vvi-miR399h|*+10_perfect match probe</td></tr>
<tr><td>vvi|miR-1519|2mut|as|</td><td>tgatctttccgtcaaaggag</td><td>vvi-miR399h|*+10_mismatch probe</td></tr>
<tr><td>vvi|miR-1520|nat|as|</td><td>agaagccctacaactact</td><td>vvi-miR399i|*-10_perfect match probe</td></tr>
<tr><td>vvi|miR-1520|2mut|as|</td><td>agaagtcctaaaactact</td><td>vvi-miR399i|*-10_mismatch probe</td></tr>
<tr><td>vvi|miR-1521|nat|as|</td><td>ccagaaggagagaagcccta</td><td>vvi-miR399i|*0_perfect match probe</td></tr>
<tr><td>vvi|miR-1521|2mut|as|</td><td>ccagaagaagagaagtccta</td><td>vvi-miR399i|*0_mismatch probe</td></tr>
<tr><td>vvi|miR-1522|nat|as|</td><td>ccatctcctgccagaaggag</td><td>vvi-miR399i|*+10_perfect match probe</td></tr>
<tr><td>vvi|miR-1522|2mut|as|</td><td>ccatctcctctcagaaggag</td><td>vvi-miR399i|*+10_mismatch probe</td></tr>
<tr><td>vvi|miR-1523|nat|as|</td><td>cgcacaaacttgagatgtgc</td><td>vvi-miR403a|*-10_perfect match probe</td></tr>
<tr><td>vvi|miR-1523|2mut|as|</td><td>cgcaaaaacttaagatgtgc</td><td>vvi-miR403a|*-10_mismatch probe</td></tr>
<tr><td>vvi|miR-1524|nat|as|</td><td>gtttgattcacgcacaaact</td><td>vvi-miR403a|*0_perfect match probe</td></tr>
<tr><td>vvi|miR-1524|2mut|as|</td><td>gtttgattcccccacaaact</td><td>vvi-miR403a|*0_mismatch probe</td></tr>
<tr><td>vvi|miR-1525|nat|as|</td><td>ctacgatggggtttgattca</td><td>vvi-miR403a|*+10_perfect match probe</td></tr>
<tr><td>vvi|miR-1525|2mut|as|</td><td>ctacgatggtctttgattca</td><td>vvi-miR403a|*+10_mismatch probe</td></tr>
<tr><td>vvi|miR-1526|nat|as|</td><td>cacaaactcgaggtttgt</td><td>vvi-miR403b|*-10_perfect match probe</td></tr>
<tr><td>vvi|miR-1526|2mut|as|</td><td>cacaaactaaaggtttgt</td><td>vvi-miR403b|*-10_mismatch probe</td></tr>
<tr><td>vvi|miR-1527|nat|as|</td><td>tggattcgcgcacaaactcg</td><td>vvi-miR403b|*0_perfect match probe</td></tr>
<tr><td>vvi|miR-1527|2mut|as|</td><td>tggattcgccaacaaactcg</td><td>vvi-miR403b|*0_mismatch probe</td></tr>
<tr><td>vvi|miR-1528|nat|as|</td><td>atcgaggcgttggattcgcg</td><td>vvi-miR403b|*+10_perfect match probe</td></tr>
</table>

vvi|miR-1528|2mut|as|	atcgaggagttcgattcgcg
vvi|miR-1529|nat|as|	cacaaacttgagatgtgc
vvi|miR-1529|2mut|as|	cacaaatttaagatgtgc
vvi|miR-1530|nat|as|	ttgattcacgcacaaacttg
vvi|miR-1530|2mut|as|	ttgattcaccaacaaacttg
vvi|miR-1531|nat|as|	acgatggggtttgattcacg
vvi|miR-1531|2mut|as|	acgatggtgtttaattcacg
vvi|miR-1532|nat|as|	cacaaactcgaggtttgt
vvi|miR-1532|2mut|as|	cacaaactaaaggtttgt
vvi|miR-1533|nat|as|	tggattcgcgcacaaactcg
vvi|miR-1533|2mut|as|	tggattcgccaacaaactcg
vvi|miR-1534|nat|as|	attgaggggttggattcgcg
vvi|miR-1534|2mut|as|	attgaggtgttcgattcgcg
vvi|miR-1535|nat|as|	cacaaacttgagatgtgc
vvi|miR-1535|2mut|as|	cacaaatttaagatgtgc
vvi|miR-1536|nat|as|	ttgattcacgcacaaacttg
vvi|miR-1536|2mut|as|	ttgattcaccaacaaacttg
vvi|miR-1537|nat|as|	acgatggggtttgattcacg
vvi|miR-1537|2mut|as|	acgatggtgtttaattcacg
vvi|miR-1538|nat|as|	gcacaaactcctcatctca
vvi|miR-1538|2mut|as|	gcacaaacttttcatctca
vvi|miR-1539|nat|as|	ttagagtcacgcacaaactc
vvi|miR-1539|2mut|as|	ttagagtcaaccacaaactc
vvi|miR-1540|nat|as|	tcaggcggttttagagtcac
vvi|miR-1540|2mut|as|	tcaggaggttttagactcac
vvi|miR-1541|nat|as|	ctcgtccccgtcttcctctt

vvi-miR403b|*+10_mismatch probe
vvi-miR403c|*-10_perfect match probe
vvi-miR403c|*-10_mismatch probe
vvi-miR403c|*0_perfect match probe
vvi-miR403c|*0_mismatch probe
vvi-miR403c|*+10_perfect match probe
vvi-miR403c|*+10_mismatch probe
vvi-miR403d|*-10_perfect match probe
vvi-miR403d|*-10_mismatch probe
vvi-miR403d|*0_perfect match probe
vvi-miR403d|*0_mismatch probe
vvi-miR403d|*+10_perfect match probe
vvi-miR403d|*+10_mismatch probe
vvi-miR403e|*-10_perfect match probe
vvi-miR403e|*-10_mismatch probe
vvi-miR403e|*0_perfect match probe
vvi-miR403e|*0_mismatch probe
vvi-miR403e|*+10_perfect match probe
vvi-miR403e|*+10_mismatch probe
vvi-miR403f|*-10_perfect match probe
vvi-miR403f|*-10_mismatch probe
vvi-miR403f|*0_perfect match probe
vvi-miR403f|*0_mismatch probe
vvi-miR403f|*+10_perfect match probe
vvi-miR403f|*+10_mismatch probe
vvi-miR408|*-10_perfect match

		probe
vvi|miR-1541|2mut|as|	ctcgtcccagtctttctctt	vvi-miR408|*-10_mismatch probe
vvi|miR-1542|nat|as|	catgcactacctcgtccccg	vvi-miR408|*0_perfect match probe
vvi|miR-1542|2mut|as|	catgaactacctcctccccg	vvi-miR408|*0_mismatch probe
		vvi-miR408|*+10_perfect match
vvi|miR-1543|nat|as|	tagttccatccatgcactac	probe
vvi|miR-1543|2mut|as|	tagttcaatccatgaactac	vvi-miR408|*+10_mismatch probe
		vvi-miR477a|*-10_perfect match
vvi|miR-1544|nat|as|	tgtcttccaactttccaacc	probe
vvi|miR-1544|2mut|as|	tgtcttcaaactttcaaacc	vvi-miR477a|*-10_mismatch probe
vvi|miR-1545|nat|as|	ggtcccccactgtcttccaa	vvi-miR477a|*0_perfect match probe
vvi|miR-1545|2mut|as|	ggtccctcactctcttccaa	vvi-miR477a|*0_mismatch probe
		vvi-miR477a|*+10_perfect match
vvi|miR-1546|nat|as|	aagcttccaaggtcccccac	probe
vvi|miR-1546|2mut|as|	aagcttcaaaggtcctccac	vvi-miR477a|*+10_mismatch probe
		vvi-miR479|*-10_perfect match
vvi|miR-1547|nat|as|	tcggctcgtctttgagccga	probe
vvi|miR-1547|2mut|as|	tcggctcctctttgacccga	vvi-miR479|*-10_mismatch probe
vvi|miR-1548|nat|as|	tgatattggttcggctcgtc	vvi-miR479|*0_perfect match probe
vvi|miR-1548|2mut|as|	tgatattcgttcgcctcgtc	vvi-miR479|*0_mismatch probe
		vvi-miR479|*+10_perfect match
vvi|miR-1549|nat|as|	catacaagagtgatattggt	probe
vvi|miR-1549|2mut|as|	cataaaagagcgatattggt	vvi-miR479|*+10_mismatch probe
		vvi-miR482a|*-10_perfect match
vvi|miR-1550|nat|as|	ctccaattcccaaacatt	probe
vvi|miR-1550|2mut|as|	ctccaattttcaaacatt	vvi-miR482a|*-10_mismatch probe
vvi|miR-1551|nat|as|	ctttcctactctccaattcc	vvi-miR482a|*0_perfect match probe
vvi|miR-1551|2mut|as|	cttttctactctcaaattcc	vvi-miR482a|*0_mismatch probe
		vvi-miR482a|*+10_perfect match
vvi|miR-1552|nat|as|	gatggctaagctttcctact	probe
vvi|miR-1552|2mut|as|	gatgcctaagttttcctact	vvi-miR482a|*+10_mismatch probe
		vvi-miR535a|*-10_perfect match
vvi|miR-1553|nat|as|	agagcacgcctgtacacagt	probe
vvi|miR-1553|2mut|as|	agagcacccctgtacccagt	vvi-miR535a|*-10_mismatch probe

vvi|miR-1554|nat|as| acagcgagagagagcacgcc vvi-miR535a|*0_perfect match probe
vvi|miR-1554|2mut|as| acagagagagagagaacgcc vvi-miR535a|*0_mismatch probe
vvi|miR-1555|nat|as| agattgtatgacagcgagag vvi-miR535a|*+10_perfect match probe
vvi|miR-1555|2mut|as| agattctatgacagagagag vvi-miR535a|*+10_mismatch probe
vvi|miR-1556|nat|as| agagcacgcctgtacacagt vvi-miR535b|*-10_perfect match probe
vvi|miR-1556|2mut|as| agagcacccctgtacccagt vvi-miR535b|*-10_mismatch probe
vvi|miR-1557|nat|as| acagcgagagagagcacgcc vvi-miR535b|*0_perfect match probe
vvi|miR-1557|2mut|as| acagagagagagagaacgcc vvi-miR535b|*0_mismatch probe
vvi|miR-1558|nat|as| agattgtatgacagcgagag vvi-miR535b|*+10_perfect match probe
vvi|miR-1558|2mut|as| agattctatgacagagagag vvi-miR535b|*+10_mismatch probe
vvi|miR-1559|nat|as| agagcacgcctgtacacagt vvi-miR535c|*-10_perfect match probe
vvi|miR-1559|2mut|as| agagcacccctgtacccagt vvi-miR535c|*-10_mismatch probe
vvi|miR-1560|nat|as| acagcgagagagagcacgcc vvi-miR535c|*0_perfect match probe
vvi|miR-1560|2mut|as| acagagagagagagaacgcc vvi-miR535c|*0_mismatch probe
vvi|miR-1561|nat|as| agattgtatgacagcgagag vvi-miR535c|*+10_perfect match probe
vvi|miR-1561|2mut|as| agattctatgacagagagag vvi-miR535c|*+10_mismatch probe
vvi|miR-1562|nat|as| agagcacgcctgtacacagt vvi-miR535d|*-10_perfect match probe
vvi|miR-1562|2mut|as| agagcacccctgtacccagt vvi-miR535d|*-10_mismatch probe
vvi|miR-1563|nat|as| acagcgacagagagcacgcc vvi-miR535d|*0_perfect match probe
vvi|miR-1563|2mut|as| acagagacagagagaacgcc vvi-miR535d|*0_mismatch probe
vvi|miR-1564|nat|as| agattgtatgacagcgacag vvi-miR535d|*+10_perfect match probe
vvi|miR-1564|2mut|as| agattctatgacagagacag vvi-miR535d|*+10_mismatch probe
vvi|miR-1565|nat|as| agagcacgcctgtacacagt vvi-miR535e|*-10_perfect match probe
vvi|miR-1565|2mut|as| agagcacccctgtacccagt vvi-miR535e|*-10_mismatch probe
vvi|miR-1566|nat|as| acagcgacagagagcacgcc vvi-miR535e|*0_perfect match probe
vvi|miR-1566|2mut|as| acagagacagagagaacgcc vvi-miR535e|*0_mismatch probe

vvi|miR-1567|nat|as|	agattgtatgacagcgacag	vvi-miR535e|*+10_perfect match probe
vvi|miR-1567|2mut|as|	agattctatgacagagacag	vvi-miR535e|*+10_mismatch probe
vvi|miR-1568|nat|as|	gagcatctcaacaacaatcc	vvi-miR828a|*-10_perfect match probe
vvi|miR-1568|2mut|as|	gagcatctaaacaaaaatcc	vvi-miR828a|*-10_mismatch probe
vvi|miR-1569|nat|as|	ttcctcaaatgagcatctca	vvi-miR828a|*0_perfect match probe
vvi|miR-1569|2mut|as|	ttcctaaaatgagaatctca	vvi-miR828a|*0_mismatch probe
vvi|miR-1570|nat|as|	ttaaggttgcttcctcaaat	vvi-miR828a|*+10_perfect match probe
vvi|miR-1570|2mut|as|	ttaaggttcctttctcaaat	vvi-miR828a|*+10_mismatch probe
vvi|miR-1571|nat|as|	tgactatccaggttgatgtg	vvi-miR828b|*-10_perfect match probe
vvi|miR-1571|2mut|as|	tgactatcaagcttgatgtg	vvi-miR828b|*-10_mismatch probe
vvi|miR-1572|nat|as|	ttgcaggaaatgactatcca	vvi-miR828b|*0_perfect match probe
vvi|miR-1572|2mut|as|	ttgcagaaaatgcctatcca	vvi-miR828b|*0_mismatch probe
vvi|miR-1573|nat|as|	aaacctggttttgcaggaaa	vvi-miR828b|*+10_perfect match probe
vvi|miR-1573|2mut|as|	aaacctcgttttgaaggaaa	vvi-miR828b|*+10_mismatch probe
vvi|miR-1574|nat|as|	aggaactaatcaagtgaaa	vvi-miR845a|*-10_perfect match probe
vvi|miR-1574|2mut|as|	aggaattaatcaagcgaaa	vvi-miR845a|*-10_mismatch probe
vvi|miR-1575|nat|as|	taactcatgaaggaactaat	vvi-miR845a|*0_perfect match probe
vvi|miR-1575|2mut|as|	taactcataaagaaactaat	vvi-miR845a|*0_mismatch probe
vvi|miR-1576|nat|as|	ttacttgcaataactcatga	vvi-miR845a|*+10_perfect match probe
vvi|miR-1576|2mut|as|	ttacttgaaataactaatga	vvi-miR845a|*+10_mismatch probe
vvi|miR-1577|nat|as|	aggaactaatcaagtgaaa	vvi-miR845b|*-10_perfect match probe
vvi|miR-1577|2mut|as|	aggaattaatcaagcgaaa	vvi-miR845b|*-10_mismatch probe
vvi|miR-1578|nat|as|	taactcatgaaggaactaat	vvi-miR845b|*0_perfect match probe
vvi|miR-1578|2mut|as|	taactcataaagaaactaat	vvi-miR845b|*0_mismatch probe
vvi|miR-1579|nat|as|	ttgcttgaaataactcatga	vvi-miR845b|*+10_perfect match probe

vvi|miR-1579|2mut|as|	ttgcttaaaataactaatga	vvi-miR845b|*+10_mismatch probe
vvi|miR-1580|nat|as|	aaccaaaacacataattg	vvi-miR845c|*-10_perfect match probe
vvi|miR-1580|2mut|as|	aaccaaaaaccataattg	vvi-miR845c|*-10_mismatch probe
vvi|miR-1581|nat|as|	aaactcagacaaccaaaaca	vvi-miR845c|*0_perfect match probe
vvi|miR-1581|2mut|as|	aaactcagaaaacaaaaaca	vvi-miR845c|*0_mismatch probe
vvi|miR-1582|nat|as|	tgccaacatgaaactcagac	vvi-miR845c|*+10_perfect match probe
vvi|miR-1582|2mut|as|	tgccaaaatgaaactaagac	vvi-miR845c|*+10_mismatch probe
vvi|miR-1583|nat|as|	ataattaatgttggttat	vvi-miR845d|*-10_perfect match probe
vvi|miR-1583|2mut|as|	ataattaacgttcgttat	vvi-miR845d|*-10_mismatch probe
vvi|miR-1584|nat|as|	ggccctagggataattaatg	vvi-miR845d|*0_perfect match probe
vvi|miR-1584|2mut|as|	ggccctagtgctaattaatg	vvi-miR845d|*0_mismatch probe
vvi|miR-1585|nat|as|	tttatcccatggccctaggg	vvi-miR845d|*+10_perfect match probe
vvi|miR-1585|2mut|as|	tttatctcatggtcctaggg	vvi-miR845d|*+10_mismatch probe
vvi|miR-1586|nat|as|	ataattaatgtgcgttct	vvi-miR845e|*-10_perfect match probe
vvi|miR-1586|2mut|as|	ataattaacgtgagttct	vvi-miR845e|*-10_mismatch probe
vvi|miR-1587|nat|as|	ggccctagggataattaatg	vvi-miR845e|*0_perfect match probe
vvi|miR-1587|2mut|as|	ggccctagtgctaattaatg	vvi-miR845e|*0_mismatch probe
vvi|miR-1588|nat|as|	tttatcccatggccctaggg	vvi-miR845e|*+10_perfect match probe
vvi|miR-1588|2mut|as|	tttatctcatggtcctaggg	vvi-miR845e|*+10_mismatch probe
vvi|miR-1589|nat|as|	gagtctttagctgatgctgg	vvi-miR156a|ctrl_perfect match probe
vvi|miR-1589|2mut|as|	gagtctttacctgatcctgg	vvi-miR156a|ctrl_mismatch probe
vvi|miR-1590|nat|as|	taaacttatacaattgcctc	vvi-miR156b|ctrl_perfect match probe
vvi|miR-1590|2mut|as|	taaatttatacaattccctc	vvi-miR156b|ctrl_mismatch probe
vvi|miR-1591|nat|as|	acccatgcaagaacagacca	vvi-miR156c|ctrl_perfect match probe
vvi|miR-1591|2mut|as|	acccatgaaagaaaagacca	vvi-miR156c|ctrl_mismatch probe

vvi|miR-1592|nat|as|	acccttatacaaattgcctg	vvi-miR156d|ctrl_perfect match probe
vvi|miR-1592|2mut|as|	acccttataaaaattccctg	vvi-miR156d|ctrl_mismatch probe
vvi|miR-1593|nat|as|	ttcatgcaagaaaagaacca	vvi-miR156e|ctrl_perfect match probe
vvi|miR-1593|2mut|as|	ttcatgaaagaaaacaacca	vvi-miR156e|ctrl_mismatch probe
vvi|miR-1594|nat|as|	agccctcttgggtatgttca	vvi-miR156f|ctrl_perfect match probe
vvi|miR-1594|2mut|as|	agccctcttctgtatgttca	vvi-miR156f|ctrl_mismatch probe
vvi|miR-1595|nat|as|	aaactccatgcacatcatca	vvi-miR156g|ctrl_perfect match probe
vvi|miR-1595|2mut|as|	aaactccatcaacatcatca	vvi-miR156g|ctrl_mismatch probe
vvi|miR-1596|nat|as|	gttgtaattgttttcccacc	vvi-miR156h|ctrl_perfect match probe
vvi|miR-1596|2mut|as|	gttgtaattcttttctcacc	vvi-miR156h|ctrl_mismatch probe
vvi|miR-1597|nat|as|	tggctccttgcatttcatca	vvi-miR156i|ctrl_perfect match probe
vvi|miR-1597|2mut|as|	tggctccttcaatttcatca	vvi-miR156i|ctrl_mismatch probe
vvi|miR-1598|nat|as|	agaaagatcaaggcaaccac	vvi-miR159a|ctrl_perfect match probe
vvi|miR-1598|2mut|as|	agaaagataaagccaaccac	vvi-miR159a|ctrl_mismatch probe
vvi|miR-1599|nat|as|	agaaaagataaaagcaaaca	vvi-miR159b|ctrl_perfect match probe
vvi|miR-1599|2mut|as|	agaaaacataaaagaaaaca	vvi-miR159b|ctrl_mismatch probe
vvi|miR-1600|nat|as|	ggatctaaacttggataatg	vvi-miR159c|ctrl_perfect match probe
vvi|miR-1600|2mut|as|	ggatctaaatttcgataatg	vvi-miR159c|ctrl_mismatch probe
vvi|miR-1601|nat|as|	acaaccttgacaagcttctt	vvi-miR160a|ctrl_perfect match probe
vvi|miR-1601|2mut|as|	acaatcttgacaagtttctt	vvi-miR160a|ctrl_mismatch probe
vvi|miR-1602|nat|as|	cactctttaacaagcttctt	vvi-miR160b|ctrl_perfect match probe
vvi|miR-1602|2mut|as|	cactttttaacaagtttctt	vvi-miR160b|ctrl_mismatch probe
vvi|miR-1603|nat|as|	ggaaagggagtggtctctac	vvi-miR160c|ctrl_perfect match probe
vvi|miR-1603|2mut|as|	ggaaagtgagtcgtctctac	vvi-miR160c|ctrl_mismatch probe

vvi|miR-1604|nat|as|	aatcctcgggtggactttgc	vvi-miR160d|ctrl_perfect match probe
vvi|miR-1604|2mut|as|	aatcctcaggtcgactttgc	vvi-miR160d|ctrl_mismatch probe
vvi|miR-1605|nat|as|	cactctttaacaagcttctt	vvi-miR160e|ctrl_perfect match probe
vvi|miR-1605|2mut|as|	cactttttaacaagtttctt	vvi-miR160e|ctrl_mismatch probe
vvi|miR-1606|nat|as|	gatgtgccggtgggctctgc	vvi-miR160f|ctrl_perfect match probe
vvi|miR-1606|2mut|as|	gatgtgtcggtggcctctgc	vvi-miR160f|ctrl_mismatch probe
vvi|miR-1607|nat|as|	attcttgatctgcttttttc	vvi-miR162|ctrl_perfect match probe
vvi|miR-1607|2mut|as|	attcttaatctcctttttc	vvi-miR162|ctrl_mismatch probe
vvi|miR-1608|nat|as|	aagggggaaaagtgaggcaa	vvi-miR164a|ctrl_perfect match probe
vvi|miR-1608|2mut|as|	aagggtgaaaagtgacgcaa	vvi-miR164a|ctrl_mismatch probe
vvi|miR-1609|nat|as|	cagatccaggtgctgattga	vvi-miR164b|ctrl_perfect match probe
vvi|miR-1609|2mut|as|	cagatcaaggtcctgattga	vvi-miR164b|ctrl_mismatch probe
vvi|miR-1610|nat|as|	agagcagattagaatttgtg	vvi-miR164d|ctrl_perfect match probe
vvi|miR-1610|2mut|as|	agagaagattacaatttgtg	vvi-miR164d|ctrl_mismatch probe
vvi|miR-1611|nat|as|	caccaacacatgctcagtac	vvi-miR166c|ctrl_perfect match probe
vvi|miR-1611|2mut|as|	caccaacccatcctcagtac	vvi-miR166c|ctrl_mismatch probe
vvi|miR-1612|nat|as|	cactaacacataatcagagg	vvi-miR166d|ctrl_perfect match probe
vvi|miR-1612|2mut|as|	cactaacccataatccgagg	vvi-miR166d|ctrl_mismatch probe
vvi|miR-1613|nat|as|	caccaacccatgatcaaaga	vvi-miR166e|ctrl_perfect match probe
vvi|miR-1613|2mut|as|	caccaactcatgataaaaga	vvi-miR166e|ctrl_mismatch probe
vvi|miR-1614|nat|as|	tttttgaggtatacataaaa	vvi-miR166f|ctrl_perfect match probe
vvi|miR-1614|2mut|as|	tttttgagctataaataaaa	vvi-miR166f|ctrl_mismatch probe
vvi|miR-1615|nat|as|	cattcaaacgctgcttgagc	vvi-miR166g|ctrl_perfect match probe
vvi|miR-1615|2mut|as|	cattcaaaccctccttgagc	vvi-miR166g|ctrl_mismatch probe
vvi|miR-1616|nat|as|	cattcagattatgtctgtac	vvi-miR166h|ctrl_perfect match

<table>
<tr><td></td><td></td><td>probe</td></tr>
<tr><td>vvi|miR-1616|2mut|as|</td><td>cattccgattatctctgtac</td><td>vvi-miR166h|ctrl_mismatch probe</td></tr>
<tr><td></td><td></td><td>vvi-miR166a|ctrl_perfect match</td></tr>
<tr><td>vvi|miR-1617|nat|as|</td><td>caaccgattatgaagtaact</td><td>probe</td></tr>
<tr><td>vvi|miR-1617|2mut|as|</td><td>caacagattataaagtaact</td><td>vvi-miR166a|ctrl_mismatch probe</td></tr>
<tr><td></td><td></td><td>vvi-miR166b|ctrl_perfect match</td></tr>
<tr><td>vvi|miR-1618|nat|as|</td><td>cttcacttatatatcttctt</td><td>probe</td></tr>
<tr><td>vvi|miR-1618|2mut|as|</td><td>cttcccttatatattttctt</td><td>vvi-miR166b|ctrl_mismatch probe</td></tr>
<tr><td></td><td></td><td>vvi-miR167a|ctrl_perfect match</td></tr>
<tr><td>vvi|miR-1619|nat|as|</td><td>gaaagagatggtgtttgttt</td><td>probe</td></tr>
<tr><td>vvi|miR-1619|2mut|as|</td><td>gaaagagatcctgtttgttt</td><td>vvi-miR167a|ctrl_mismatch probe</td></tr>
<tr><td></td><td></td><td>vvi-miR167b|ctrl_perfect match</td></tr>
<tr><td>vvi|miR-1620|nat|as|</td><td>aaagggtgggcaacagaaaa</td><td>probe</td></tr>
<tr><td>vvi|miR-1620|2mut|as|</td><td>aaagggtggcaaacagaaaa</td><td>vvi-miR167b|ctrl_mismatch probe</td></tr>
<tr><td></td><td></td><td>vvi-miR167c|ctrl_perfect match</td></tr>
<tr><td>vvi|miR-1621|nat|as|</td><td>ccttgacttgtatagggaag</td><td>probe</td></tr>
<tr><td>vvi|miR-1621|2mut|as|</td><td>ccttgacttctatagtgaag</td><td>vvi-miR167c|ctrl_mismatch probe</td></tr>
<tr><td></td><td></td><td>vvi-miR167d|ctrl_perfect match</td></tr>
<tr><td>vvi|miR-1622|nat|as|</td><td>ttctctgtatccctagccaa</td><td>probe</td></tr>
<tr><td>vvi|miR-1622|2mut|as|</td><td>ttctctctatctctagccaa</td><td>vvi-miR167d|ctrl_mismatch probe</td></tr>
<tr><td></td><td></td><td>vvi-miR167e|ctrl_perfect match</td></tr>
<tr><td>vvi|miR-1623|nat|as|</td><td>ctgttcacaaagggaagcaa</td><td>probe</td></tr>
<tr><td>vvi|miR-1623|2mut|as|</td><td>ctgttcccaaagtgaagcaa</td><td>vvi-miR167e|ctrl_mismatch probe</td></tr>
<tr><td>vvi|miR-1624|nat|as|</td><td>cgctgccggagcggcgaagt</td><td>vvi-miR168|ctrl_perfect match probe</td></tr>
<tr><td>vvi|miR-1624|2mut|as|</td><td>cgctgtcggagcggagaagt</td><td>vvi-miR168|ctrl_mismatch probe</td></tr>
<tr><td></td><td></td><td>vvi-miR169a|ctrl_perfect match</td></tr>
<tr><td>vvi|miR-1625|nat|as|</td><td>tctcttctcactatatataa</td><td>probe</td></tr>
<tr><td>vvi|miR-1625|2mut|as|</td><td>tctcttctccttatatataa</td><td>vvi-miR169a|ctrl_mismatch probe</td></tr>
<tr><td></td><td></td><td>vvi-miR169y|ctrl_perfect match</td></tr>
<tr><td>vvi|miR-1626|nat|as|</td><td>attttgaaaccttaaggagg</td><td>probe</td></tr>
<tr><td>vvi|miR-1626|2mut|as|</td><td>attttgaaatcttaacgagg</td><td>vvi-miR169y|ctrl_mismatch probe</td></tr>
<tr><td></td><td></td><td>vvi-miR169b|ctrl_perfect match</td></tr>
<tr><td>vvi|miR-1627|nat|as|</td><td>tctccaactcttgctgcaga</td><td>probe</td></tr>
<tr><td>vvi|miR-1627|2mut|as|</td><td>tctcaaactcttgctccaga</td><td>vvi-miR169b|ctrl_mismatch probe</td></tr>
</table>

vvi|miR-1628|nat|as|	gattaacgaggaatgcccca	vvi-miR169c|ctrl_perfect match probe
vvi|miR-1628|2mut|as|	gattaaagaggaatgtccca	vvi-miR169c|ctrl_mismatch probe
vvi|miR-1629|nat|as|	tctcttctcactatatataa	vvi-miR169d|ctrl_perfect match probe
vvi|miR-1629|2mut|as|	tctcttctccttatatataa	vvi-miR169d|ctrl_mismatch probe
vvi|miR-1630|nat|as|	aaattgacccacttgggata	vvi-miR169e|ctrl_perfect match probe
vvi|miR-1630|2mut|as|	aaattgactcacttgtgata	vvi-miR169e|ctrl_mismatch probe
vvi|miR-1631|nat|as|	attaacgatcgagcaaatta	vvi-miR169f|ctrl_perfect match probe
vvi|miR-1631|2mut|as|	attaaagatcgagaaaatta	vvi-miR169f|ctrl_mismatch probe
vvi|miR-1632|nat|as|	ggcagatggctaagtagaga	vvi-miR169g|ctrl_perfect match probe
vvi|miR-1632|2mut|as|	ggcagatgcctaactagaga	vvi-miR169g|ctrl_mismatch probe
vvi|miR-1633|nat|as|	atgcctcaaatagtgacaaa	vvi-miR169h|ctrl_perfect match probe
vvi|miR-1633|2mut|as|	atgcttcaaatagcgacaaa	vvi-miR169h|ctrl_mismatch probe
vvi|miR-1634|nat|as|	cctcaaataccaactgaaaa	vvi-miR169i|ctrl_perfect match probe
vvi|miR-1634|2mut|as|	cctcaaatataaactgaaaa	vvi-miR169i|ctrl_mismatch probe
vvi|miR-1635|nat|as|	tcattccactctatatgtga	vvi-miR169j|ctrl_perfect match probe
vvi|miR-1635|2mut|as|	tcattcaactttatatgtga	vvi-miR169j|ctrl_mismatch probe
vvi|miR-1636|nat|as|	tcattccactctatatgtga	vvi-miR169k|ctrl_perfect match probe
vvi|miR-1636|2mut|as|	tcattcaactttatatgtga	vvi-miR169k|ctrl_mismatch probe
vvi|miR-1637|nat|as|	agcttcaatgcttgatgcaa	vvi-miR169l|ctrl_perfect match probe
vvi|miR-1637|2mut|as|	agcttcaatctttgatgcaa	vvi-miR169l|ctrl_mismatch probe
vvi|miR-1638|nat|as|	accttaatgccttgctgcag	vvi-miR169m|ctrl_perfect match probe
vvi|miR-1638|2mut|as|	accttaatgtcttcctgcag	vvi-miR169m|ctrl_mismatch probe
vvi|miR-1639|nat|as|	aaccctatgccttgctgcag	vvi-miR169n|ctrl_perfect match probe
vvi|miR-1639|2mut|as|	aaccctatgtcttcctgcag	vvi-miR169n|ctrl_mismatch probe
vvi|miR-1640|nat|as|	agcttcaatgcttgctgctg	vvi-miR169o|ctrl_perfect match probe

vvi|miR-1640|2mut|as|	agcttcaatccttcctgctg	vvi-miR169o|ctrl_mismatch probe
vvi|miR-1641|nat|as|	accttaatgccttgctgcag	vvi-miR169p|ctrl_perfect match probe
vvi|miR-1641|2mut|as|	accttaatgtcttcctgcag	vvi-miR169p|ctrl_mismatch probe
vvi|miR-1642|nat|as|	ttaatatagacttactgcaa	vvi-miR169q|ctrl_perfect match probe
vvi|miR-1642|2mut|as|	ttaatatagccttaccgcaa	vvi-miR169q|ctrl_mismatch probe
vvi|miR-1643|nat|as|	gcatgccttctgcaaatata	vvi-miR169r|ctrl_perfect match probe
vvi|miR-1643|2mut|as|	gcatgtcttctgaaaatata	vvi-miR169r|ctrl_mismatch probe
vvi|miR-1644|nat|as|	tcgatggtgcctaatgccaa	vvi-miR169s|ctrl_perfect match probe
vvi|miR-1644|2mut|as|	tcgatggtgtctaatcccaa	vvi-miR169s|ctrl_mismatch probe
vvi|miR-1645|nat|as|	caagtccttccgcaaatata	vvi-miR169t|ctrl_perfect match probe
vvi|miR-1645|2mut|as|	caagtcctttcccaaatata	vvi-miR169t|ctrl_mismatch probe
vvi|miR-1646|nat|as|	cgtgcccttctgcaaatata	vvi-miR169u|ctrl_perfect match probe
vvi|miR-1646|2mut|as|	cgtgtccttctgaaaatata	vvi-miR169u|ctrl_mismatch probe
vvi|miR-1647|nat|as|	gacaaagctgcctactttca	vvi-miR169v|ctrl_perfect match probe
vvi|miR-1647|2mut|as|	gacaaagctctctactttca	vvi-miR169v|ctrl_mismatch probe
vvi|miR-1648|nat|as|	catgagtacaataaagggag	vvi-miR169w|ctrl_perfect match probe
vvi|miR-1648|2mut|as|	catgagtaaaataaacggag	vvi-miR169w|ctrl_mismatch probe
vvi|miR-1649|nat|as|	agtgttttgaaacctggtgg	vvi-miR169x|ctrl_perfect match probe
vvi|miR-1649|2mut|as|	agtgttttaaaacctcgtgg	vvi-miR169x|ctrl_mismatch probe
vvi|miR-1650|nat|as|	aaccaaagatctcaatggtt	vvi-miR171a|ctrl_perfect match probe
vvi|miR-1650|2mut|as|	aaccaaacatctaaatggtt	vvi-miR171a|ctrl_mismatch probe
vvi|miR-1651|nat|as|	cacatggctaatatctcttc	vvi-miR171b|ctrl_perfect match probe
vvi|miR-1651|2mut|as|	cacatgcctaatatcccttc	vvi-miR171b|ctrl_mismatch probe
vvi|miR-1652|nat|as|	aaaaccagaactcaaaaaga	vvi-miR171c|ctrl_perfect match probe
vvi|miR-1652|2mut|as|	aaaacaagaactaaaaaaga	vvi-miR171c|ctrl_mismatch probe

vvi|miR-1653|nat|as|	aacaacagagttcttaactt	vvi-miR171d|ctrl_perfect match probe
vvi|miR-1653|2mut|as|	aacaaaagagtttttaactt	vvi-miR171d|ctrl_mismatch probe
vvi|miR-1654|nat|as|	atcgttctttacggctcttc	vvi-miR171e|ctrl_perfect match probe
vvi|miR-1654|2mut|as|	atcgttttttacgcctcttc	vvi-miR171e|ctrl_mismatch probe
vvi|miR-1655|nat|as|	gagatcagtttttacattca	vvi-miR171f|ctrl_perfect match probe
vvi|miR-1655|2mut|as|	gagatccgtttttaaattca	vvi-miR171f|ctrl_mismatch probe
vvi|miR-1656|nat|as|	gttggtgtcccccaccgatg	vvi-miR171g|ctrl_perfect match probe
vvi|miR-1656|2mut|as|	gttggtgtctcccacagatg	vvi-miR171g|ctrl_mismatch probe
vvi|miR-1657|nat|as|	atcagagtcgatacattcat	vvi-miR171h|ctrl_perfect match probe
vvi|miR-1657|2mut|as|	atcagagtagataaattcat	vvi-miR171h|ctrl_mismatch probe
vvi|miR-1658|nat|as|	gattaaaggacccaaaaccc	vvi-miR171i|ctrl_perfect match probe
vvi|miR-1658|2mut|as|	gattaaagaactcaaaaccc	vvi-miR171i|ctrl_mismatch probe
vvi|miR-1659|nat|as|	gcaaatgcacggtggtgatg	vvi-miR172a|ctrl_perfect match probe
vvi|miR-1659|2mut|as|	gcaaatgaacagtggtgatg	vvi-miR172a|ctrl_mismatch probe
vvi|miR-1660|nat|as|	gcgaaagcccggcgcggcag	vvi-miR172b|ctrl_perfect match probe
vvi|miR-1660|2mut|as|	gcgaaagtccggcgaggcag	vvi-miR172b|ctrl_mismatch probe
vvi|miR-1661|nat|as|	atcaaagagccatcggagtg	vvi-miR172c|ctrl_perfect match probe
vvi|miR-1661|2mut|as|	atcaaagagtcatcagagtg	vvi-miR172c|ctrl_mismatch probe
vvi|miR-1662|nat|as|	ctcaaaatctttggcagaga	vvi-miR172d|ctrl_perfect match probe
vvi|miR-1662|2mut|as|	ctcaaaatttttgccagaga	vvi-miR172d|ctrl_mismatch probe
vvi|miR-1663|nat|as|	tgacagccataactgaacgc	vvi-miR319b|ctrl_perfect match probe
vvi|miR-1663|2mut|as|	tgacagtcataaccgaacgc	vvi-miR319b|ctrl_mismatch probe
vvi|miR-1664|nat|as|	agggagcttaagatccaatt	vvi-miR319c|ctrl_perfect match probe
vvi|miR-1664|2mut|as|	agggagtttaagatcaaatt	vvi-miR319c|ctrl_mismatch probe

vvi|miR-1665|nat|as| aggagcatgaagttcatcat vvi-miR319e|ctrl_perfect match probe
vvi|miR-1665|2mut|as| aggagaatgaagttaatcat vvi-miR319e|ctrl_mismatch probe
vvi|miR-1666|nat|as| agacaagaaaaggatgaaat vvi-miR319f|ctrl_perfect match probe
vvi|miR-1666|2mut|as| agacaacaaaagaatgaaat vvi-miR319f|ctrl_mismatch probe
vvi|miR-1667|nat|as| tgggcaaagcgtgcggagga vvi-miR319g|ctrl_perfect match probe
vvi|miR-1667|2mut|as| tgggcaaagagtgaggagga vvi-miR319g|ctrl_mismatch probe
vvi|miR-1668|nat|as| caatcattttcatcatggct vvi-miR390|ctrl_perfect match probe
vvi|miR-1668|2mut|as| caataattttcataatggct vvi-miR390|ctrl_mismatch probe
vvi|miR-1669|nat|as| aagagaggtggaagataacc vvi-miR393a|ctrl_perfect match probe
vvi|miR-1669|2mut|as| aagagaggtcaaagataacc vvi-miR393a|ctrl_mismatch probe
vvi|miR-1670|nat|as| gatccaaagacatggagagt vvi-miR393b|ctrl_perfect match probe
vvi|miR-1670|2mut|as| gatcaaaagacatcgagagt vvi-miR393b|ctrl_mismatch probe
vvi|miR-1671|nat|as| gaatatagacccatgatgtg vvi-miR394a|ctrl_perfect match probe
vvi|miR-1671|2mut|as| gaatatagattcatgatgtg vvi-miR394a|ctrl_mismatch probe
vvi|miR-1672|nat|as| aaaagagagattttcaagag vvi-miR394b|ctrl_perfect match probe
vvi|miR-1672|2mut|as| aaaagagcgattttaaagag vvi-miR394b|ctrl_mismatch probe
vvi|miR-1673|nat|as| aattcagaagaattggtata vvi-miR394c|ctrl_perfect match probe
vvi|miR-1673|2mut|as| aattccgaagaattcgtata vvi-miR394c|ctrl_mismatch probe
vvi|miR-1674|nat|as| gaagtcattaaaactagcga vvi-miR395a|ctrl_perfect match probe
vvi|miR-1674|2mut|as| gaagccattaaaattagcga vvi-miR395a|ctrl_mismatch probe
vvi|miR-1675|nat|as| gtaggcattcattagagaag vvi-miR395b|ctrl_perfect match probe
vvi|miR-1675|2mut|as| gtagccattcattagcgaag vvi-miR395b|ctrl_mismatch probe
vvi|miR-1676|nat|as| gaagtcattataactagaga vvi-miR395c|ctrl_perfect match probe
vvi|miR-1676|2mut|as| gaagccattataattagaga vvi-miR395c|ctrl_mismatch probe

<table>
<tr><td>vvi|miR-1677|nat|as|</td><td>gaagtcattagagaagatcc</td><td>vvi-miR395d|ctrl_perfect match probe</td></tr>
<tr><td>vvi|miR-1677|2mut|as|</td><td>gaagccattagcgaagatcc</td><td>vvi-miR395d|ctrl_mismatch probe</td></tr>
<tr><td>vvi|miR-1678|nat|as|</td><td>gaagtcattataattagaga</td><td>vvi-miR395e|ctrl_perfect match probe</td></tr>
<tr><td>vvi|miR-1678|2mut|as|</td><td>gaagccattataattcgaga</td><td>vvi-miR395e|ctrl_mismatch probe</td></tr>
<tr><td>vvi|miR-1679|nat|as|</td><td>gaagtcattagagaagatcc</td><td>vvi-miR395f|ctrl_perfect match probe</td></tr>
<tr><td>vvi|miR-1679|2mut|as|</td><td>gaagccattagcgaagatcc</td><td>vvi-miR395f|ctrl_mismatch probe</td></tr>
<tr><td>vvi|miR-1680|nat|as|</td><td>gaattgaaaactaaggatcc</td><td>vvi-miR395g|ctrl_perfect match probe</td></tr>
<tr><td>vvi|miR-1680|2mut|as|</td><td>gaattaaaaactaagaatcc</td><td>vvi-miR395g|ctrl_mismatch probe</td></tr>
<tr><td>vvi|miR-1681|nat|as|</td><td>gaagtcattataattagaga</td><td>vvi-miR395h|ctrl_perfect match probe</td></tr>
<tr><td>vvi|miR-1681|2mut|as|</td><td>gaagccattataattcgaga</td><td>vvi-miR395h|ctrl_mismatch probe</td></tr>
<tr><td>vvi|miR-1682|nat|as|</td><td>gaagtcattacagaagatcc</td><td>vvi-miR395i|ctrl_perfect match probe</td></tr>
<tr><td>vvi|miR-1682|2mut|as|</td><td>gaagccattaaagaagatcc</td><td>vvi-miR395i|ctrl_mismatch probe</td></tr>
<tr><td>vvi|miR-1683|nat|as|</td><td>gaagtcattaaagaagatcc</td><td>vvi-miR395j|ctrl_perfect match probe</td></tr>
<tr><td>vvi|miR-1683|2mut|as|</td><td>gaagccattaaagaacatcc</td><td>vvi-miR395j|ctrl_mismatch probe</td></tr>
<tr><td>vvi|miR-1684|nat|as|</td><td>gaagtcattataattagaga</td><td>vvi-miR395k|ctrl_perfect match probe</td></tr>
<tr><td>vvi|miR-1684|2mut|as|</td><td>gaagccattataattcgaga</td><td>vvi-miR395k|ctrl_mismatch probe</td></tr>
<tr><td>vvi|miR-1685|nat|as|</td><td>gaagtcattacagaagatcc</td><td>vvi-miR395l|ctrl_perfect match probe</td></tr>
<tr><td>vvi|miR-1685|2mut|as|</td><td>gaagccattaaagaagatcc</td><td>vvi-miR395l|ctrl_mismatch probe</td></tr>
<tr><td>vvi|miR-1686|nat|as|</td><td>gaagtcattataactagaga</td><td>vvi-miR395m|ctrl_perfect match probe</td></tr>
<tr><td>vvi|miR-1686|2mut|as|</td><td>gaagccattataattagaga</td><td>vvi-miR395m|ctrl_mismatch probe</td></tr>
<tr><td>vvi|miR-1687|nat|as|</td><td>aggaaatcaccctggaaata</td><td>vvi-miR395n|ctrl_perfect match probe</td></tr>
<tr><td>vvi|miR-1687|2mut|as|</td><td>aggaaatcattctggaaata</td><td>vvi-miR395n|ctrl_mismatch probe</td></tr>
<tr><td>vvi|miR-1688|nat|as|</td><td>tcttcataaggagtgtgaaa</td><td>vvi-miR396a|ctrl_perfect match probe</td></tr>
<tr><td>vvi|miR-1688|2mut|as|</td><td>tcttcataacaagtgtgaaa</td><td>vvi-miR396a|ctrl_mismatch probe</td></tr>
<tr><td>vvi|miR-1689|nat|as|</td><td>ctcctacgtttacccatcca</td><td>vvi-miR396b|ctrl_perfect match probe</td></tr>
</table>

vvi|miR-1689|2mut|as|	ctcctacctttactcatcca	vvi-miR396b|ctrl_mismatch probe
		vvi-miR396c|ctrl_perfect match
vvi|miR-1690|nat|as|	atggaagtggaaggcttcca	probe
vvi|miR-1690|2mut|as|	atggaagtcgaagccttcca	vvi-miR396c|ctrl_mismatch probe
		vvi-miR396d|ctrl_perfect match
vvi|miR-1691|nat|as|	acacaccagtagtctgaaaa	probe
vvi|miR-1691|2mut|as|	acacacaagtagcctgaaaa	vvi-miR396d|ctrl_mismatch probe
		vvi-miR397a|ctrl_perfect match
vvi|miR-1692|nat|as|	tgaaaaatggaatacttcag	probe
vvi|miR-1692|2mut|as|	tgaaaaatcgaatatttcag	vvi-miR397a|ctrl_mismatch probe
		vvi-miR397b|ctrl_perfect match
vvi|miR-1693|nat|as|	tgaaaaatggaatacttcag	probe
vvi|miR-1693|2mut|as|	tgaaaaatcgaatatttcag	vvi-miR397b|ctrl_mismatch probe
		vvi-miR398a|ctrl_perfect match
vvi|miR-1694|nat|as|	tgcattagctagcagagctt	probe
vvi|miR-1694|2mut|as|	tgcattagttagaagagctt	vvi-miR398a|ctrl_mismatch probe
		vvi-miR398b|ctrl_perfect match
vvi|miR-1695|nat|as|	gagcaaagatgggtggtgcg	probe
vvi|miR-1695|2mut|as|	gagcaaacatgtgtggtgcg	vvi-miR398b|ctrl_mismatch probe
		vvi-miR398c|ctrl_perfect match
vvi|miR-1696|nat|as|	gagcaaagatgggtgatgcg	probe
vvi|miR-1696|2mut|as|	gagcaaacatgtgtgatgcg	vvi-miR398c|ctrl_mismatch probe
		vvi-miR399a|ctrl_perfect match
vvi|miR-1697|nat|as|	cagctaccgccttctactcc	probe
vvi|miR-1697|2mut|as|	cagctacccctttctactcc	vvi-miR399a|ctrl_mismatch probe
		vvi-miR399b|ctrl_perfect match
vvi|miR-1698|nat|as|	cacatctttttactgcagct	probe
vvi|miR-1698|2mut|as|	cacatttttttactgaagct	vvi-miR399b|ctrl_mismatch probe
		vvi-miR399c|ctrl_perfect match
vvi|miR-1699|nat|as|	cattcacatctatgtcacct	probe
vvi|miR-1699|2mut|as|	cattcccatctatctcacct	vvi-miR399c|ctrl_mismatch probe
		vvi-miR399d|ctrl_perfect match
vvi|miR-1700|nat|as|	acactcaatgccctttgagc	probe
vvi|miR-1700|2mut|as|	acactcaatctcctttgagc	vvi-miR399d|ctrl_mismatch probe

vvi|miR-1701|nat|as|	ggcacactacaagaatgcat	vvi-miR399e|ctrl_perfect match probe
vvi|miR-1701|2mut|as|	ggcaccctacaacaatgcat	vvi-miR399e|ctrl_mismatch probe
vvi|miR-1702|nat|as|	agaagactgcagacacagac	vvi-miR399f|ctrl_perfect match probe
vvi|miR-1702|2mut|as|	agaagactgaagacccagac	vvi-miR399f|ctrl_mismatch probe
vvi|miR-1703|nat|as|	agcttagtctgctacttgtg	vvi-miR399g|ctrl_perfect match probe
vvi|miR-1703|2mut|as|	agcttagcctcctacttgtg	vvi-miR399g|ctrl_mismatch probe
vvi|miR-1704|nat|as|	cagaagtatgcatgtgcatg	vvi-miR399h|ctrl_perfect match probe
vvi|miR-1704|2mut|as|	cagaagtatccatgtccatg	vvi-miR399h|ctrl_mismatch probe
vvi|miR-1705|nat|as|	cattggaagttaatcaacac	vvi-miR399i|ctrl_perfect match probe
vvi|miR-1705|2mut|as|	cattcgaagttaataaacac	vvi-miR399i|ctrl_mismatch probe
vvi|miR-1706|nat|as|	cgtcgaatcgccccgtgatt	vvi-miR403a|ctrl_perfect match probe
vvi|miR-1706|2mut|as|	cgtcgaatcctcccgtgatt	vvi-miR403a|ctrl_mismatch probe
vvi|miR-1707|nat|as|	ccctttgaaagaagatcgag	vvi-miR403b|ctrl_perfect match probe
vvi|miR-1707|2mut|as|	ccctttaaaagaacatcgag	vvi-miR403b|ctrl_mismatch probe
vvi|miR-1708|nat|as|	cgtcgaatcgccccgtgatt	vvi-miR403c|ctrl_perfect match probe
vvi|miR-1708|2mut|as|	cgtcgaatcctcccgtgatt	vvi-miR403c|ctrl_mismatch probe
vvi|miR-1709|nat|as|	ccctttgaaagaagattgag	vvi-miR403d|ctrl_perfect match probe
vvi|miR-1709|2mut|as|	ccctttaaaagaacattgag	vvi-miR403d|ctrl_mismatch probe
vvi|miR-1710|nat|as|	cgtcgaatcgccccgtgatt	vvi-miR403e|ctrl_perfect match probe
vvi|miR-1710|2mut|as|	cgtcgaatcctcccgtgatt	vvi-miR403e|ctrl_mismatch probe
vvi|miR-1711|nat|as|	catagaaaaggtatcgcatc	vvi-miR403f|ctrl_perfect match probe
vvi|miR-1711|2mut|as|	catagaaaacgtatcccatc	vvi-miR403f|ctrl_mismatch probe
vvi|miR-1712|nat|as|	tagagcaaaaacagcttaac	vvi-miR408|ctrl_perfect match probe
vvi|miR-1712|2mut|as|	tagagaaaaaacagtttaac	vvi-miR408|ctrl_mismatch probe
vvi|miR-1713|nat|as|	accttgataatcagccagga	vvi-miR477a|ctrl_perfect match probe

vvi|miR-1713|2mut|as|	accttaataatcagtcagga	vvi-miR477a|ctrl_mismatch probe
vvi|miR-1714|nat|as|	gccgatgaaaatgaatgaaa	vvi-miR479|ctrl_perfect match probe
vvi|miR-1714|2mut|as|	gccgataaaaataaatgaaa	vvi-miR479|ctrl_mismatch probe
		vvi-miR482a|ctrl_perfect match
vvi|miR-1715|nat|as|	gactagctctagacccccaa	probe
vvi|miR-1715|2mut|as|	gactagttctagacctccaa	vvi-miR482a|ctrl_mismatch probe
		vvi-miR535a|ctrl_perfect match
vvi|miR-1716|nat|as|	tgttcctcgcatgaatgctg	probe
vvi|miR-1716|2mut|as|	tgttcctcccataaatgctg	vvi-miR535a|ctrl_mismatch probe
		vvi-miR535b|ctrl_perfect match
vvi|miR-1717|nat|as|	tgttcctcgcatgaatgctg	probe
vvi|miR-1717|2mut|as|	tgttcctcccataaatgctg	vvi-miR535b|ctrl_mismatch probe
		vvi-miR535c|ctrl_perfect match
vvi|miR-1718|nat|as|	tgttcctcgcatgaatgctg	probe
vvi|miR-1718|2mut|as|	tgttcctcccataaatgctg	vvi-miR535c|ctrl_mismatch probe
		vvi-miR535d|ctrl_perfect match
vvi|miR-1719|nat|as|	tgttcctcgcatgaatgcag	probe
vvi|miR-1719|2mut|as|	tgttcctcccataaatgcag	vvi-miR535d|ctrl_mismatch probe
		vvi-miR535e|ctrl_perfect match
vvi|miR-1720|nat|as|	tgttcctcgcatgaatgcag	probe
vvi|miR-1720|2mut|as|	tgttcctcccataaatgcag	vvi-miR535e|ctrl_mismatch probe
		vvi-miR828a|ctrl_perfect match
vvi|miR-1721|nat|as|	tcattgcttcacaagctgtt	probe
vvi|miR-1721|2mut|as|	tcattccttcacaagttgtt	vvi-miR828a|ctrl_mismatch probe
		vvi-miR828b|ctrl_perfect match
vvi|miR-1722|nat|as|	ggaaatgactatccaggttg	probe
vvi|miR-1722|2mut|as|	ggaaatgcctatcaaggttg	vvi-miR828b|ctrl_mismatch probe
		vvi-miR845a|ctrl_perfect match
vvi|miR-1723|nat|as|	ctcttgcttaagacttaatc	probe
vvi|miR-1723|2mut|as|	ctcttccttaagccttaatc	vvi-miR845a|ctrl_mismatch probe
		vvi-miR845b|ctrl_perfect match
vvi|miR-1724|nat|as|	ctcttgcttaagacttaatc	probe
vvi|miR-1724|2mut|as|	ctcttccttaagccttaatc	vvi-miR845b|ctrl_mismatch probe
vvi|miR-1725|nat|as|	gtttaatcattaagaaattg	vvi-miR845c|ctrl_perfect match

<table>
<tr><td></td><td></td><td>probe</td></tr>
<tr><td>vvi|miR-1725|2mut|as|</td><td>gtttaataattaacaaattg</td><td>vvi-miR845c|ctrl_mismatch probe</td></tr>
<tr><td></td><td></td><td>vvi-miR845d|ctrl_perfect match</td></tr>
<tr><td>vvi|miR-1726|nat|as|</td><td>gttagtaattgtgctagaac</td><td>probe</td></tr>
<tr><td>vvi|miR-1726|2mut|as|</td><td>gttactaattgtcctagaac</td><td>vvi-miR845d|ctrl_mismatch probe</td></tr>
<tr><td></td><td></td><td>vvi-miR845e|ctrl_perfect match</td></tr>
<tr><td>vvi|miR-1727|nat|as|</td><td>gttagtaatcgtgctagaac</td><td>probe</td></tr>
<tr><td>vvi|miR-1727|2mut|as|</td><td>gttagtaatagtcctagaac</td><td>vvi-miR845e|ctrl_mismatch probe</td></tr>
<tr><td></td><td></td><td>vvi-miR156a|compl_perfect match</td></tr>
<tr><td>vvi|miR-1728|nat|as|</td><td>tgacagaagagagggagcac</td><td>probe</td></tr>
<tr><td>vvi|miR-1728|2mut|as|</td><td>tgaccgaagagagtgagcac</td><td>vvi-miR156a|compl_mismatch probe</td></tr>
<tr><td></td><td></td><td>vvi-miR156b|compl_perfect match</td></tr>
<tr><td>vvi|miR-1729|nat|as|</td><td>tgacagaagagagtgagcac</td><td>probe</td></tr>
<tr><td>vvi|miR-1729|2mut|as|</td><td>tgaccgaagagagcgagcac</td><td>vvi-miR156b|compl_mismatch probe</td></tr>
<tr><td></td><td></td><td>vvi-miR156e|compl_perfect match</td></tr>
<tr><td>vvi|miR-1730|nat|as|</td><td>tgacagaggagagtgagcac</td><td>probe</td></tr>
<tr><td>vvi|miR-1730|2mut|as|</td><td>tgacagagaagagcgagcac</td><td>vvi-miR156e|compl_mismatch probe</td></tr>
<tr><td></td><td></td><td>vvi-miR156f|compl_perfect match</td></tr>
<tr><td>vvi|miR-1731|nat|as|</td><td>ttgacagaagatagagagcac</td><td>probe</td></tr>
<tr><td>vvi|miR-1731|2mut|as|</td><td>ttgaaagaagatagagcgcac</td><td>vvi-miR156f|compl_mismatch probe</td></tr>
<tr><td></td><td></td><td>vvi-miR156h|compl_perfect match</td></tr>
<tr><td>vvi|miR-1732|nat|as|</td><td>tgacagaagagagagagcat</td><td>probe</td></tr>
<tr><td>vvi|miR-1732|2mut|as|</td><td>tgaccgaagagagagcgcat</td><td>vvi-miR156h|compl_mismatch probe</td></tr>
<tr><td></td><td></td><td>vvi-miR156i|compl_perfect match</td></tr>
<tr><td>vvi|miR-1733|nat|as|</td><td>tgacagaagatagagagcac</td><td>probe</td></tr>
<tr><td>vvi|miR-1733|2mut|as|</td><td>tgaccgaagatagagcgcac</td><td>vvi-miR156i|compl_mismatch probe</td></tr>
<tr><td></td><td></td><td>vvi-miR159a|compl_perfect match</td></tr>
<tr><td>vvi|miR-1734|nat|as|</td><td>cttggagtgaagggagctctc</td><td>probe</td></tr>
<tr><td>vvi|miR-1734|2mut|as|</td><td>cttgaagtgaagtgagctctc</td><td>vvi-miR159a|compl_mismatch probe</td></tr>
<tr><td></td><td></td><td>vvi-miR159b|compl_perfect match</td></tr>
<tr><td>vvi|miR-1735|nat|as|</td><td>cttggagtgaagggagctctca</td><td>probe</td></tr>
<tr><td>vvi|miR-1735|2mut|as|</td><td>cttgaagtgaagtgagctctca</td><td>vvi-miR159b|compl_mismatch probe</td></tr>
<tr><td></td><td></td><td>vvi-miR159c|compl_perfect match</td></tr>
<tr><td>vvi|miR-1736|nat|as|</td><td>tttggattgaagggagctcta</td><td>probe</td></tr>
</table>

vvi|miR-1736|2mut|as| tttgaattgaagtgagctcta vvi-miR159c|compl_mismatch probe

vvi-miR160a|compl_perfect match probe

vvi|miR-1737|nat|as| tgcctggctccctgaatgcca

vvi|miR-1737|2mut|as| tgcctgcctccttgaatgcca vvi-miR160a|compl_mismatch probe

vvi-miR160c|compl_perfect match probe

vvi|miR-1738|nat|as| tgcctggctccctgtatgcca

vvi|miR-1738|2mut|as| tgcctgcctccttgtatgcca vvi-miR160c|compl_mismatch probe

vvi-miR162|compl_perfect match probe

vvi|miR-1739|nat|as| tcgataaacctctgcatccag

vvi|miR-1739|2mut|as| tcgataaatctctgaatccag vvi-miR162|compl_mismatch probe

vvi-miR164a|compl_perfect match probe

vvi|miR-1740|nat|as| tggagaagcagggcacgtgca

vvi|miR-1740|2mut|as| tggagaagaaggccacgtgca vvi-miR164a|compl_mismatch probe

vvi-miR164b|compl_perfect match probe

vvi|miR-1741|nat|as| tggagaagcagggcacatgct

vvi|miR-1741|2mut|as| tggagaagaaggccacatgct vvi-miR164b|compl_mismatch probe

vvi-miR166c|compl_perfect match probe

vvi|miR-1742|nat|as| tcggaccaggcttcattccccc

vvi|miR-1742|2mut|as| tcggaccagccttcatttcccc vvi-miR166c|compl_mismatch probe

vvi-miR166d|compl_perfect match probe

vvi|miR-1743|nat|as| tcggaccaggcttcattcccct

vvi|miR-1743|2mut|as| tcggaccagccttcatttccct vvi-miR166d|compl_mismatch probe

vvi-miR166a|compl_perfect match probe

vvi|miR-1744|nat|as| tcggaccaggcttcattcctg

vvi|miR-1744|2mut|as| tcggaccagccttaattcctg vvi-miR166a|compl_mismatch probe

vvi-miR166b|compl_perfect match probe

vvi|miR-1745|nat|as| tcggaccaggcttcattcctc

vvi|miR-1745|2mut|as| tcggaccagccttaattcctc vvi-miR166b|compl_mismatch probe

vvi-miR167a|compl_perfect match probe

vvi|miR-1746|nat|as| tgaagctgccagcatgatctg

vvi|miR-1746|2mut|as| tgaagctgtcagaatgatctg vvi-miR167a|compl_mismatch probe

vvi-miR167b|compl_perfect match probe

vvi|miR-1747|nat|as| tgaagctgccagcatgatcta

vvi|miR-1747|2mut|as| tgaagctgtcagaatgatcta vvi-miR167b|compl_mismatch probe

vvi|miR-1748|nat|as|	tgaagctgccagcatgatct	vvi-miR167c|compl_perfect match probe
vvi|miR-1748|2mut|as|	tgaagctgtcagaatgatct	vvi-miR167c|compl_mismatch probe
vvi|miR-1749|nat|as|	tcgcttggtgcaggtcgggaa	vvi-miR168|compl_perfect match probe
vvi|miR-1749|2mut|as|	tcgcttggtgaaggtcaggaa	vvi-miR168|compl_mismatch probe
vvi|miR-1750|nat|as|	cagccaaggatgacttgccgg	vvi-miR169a|compl_perfect match probe
vvi|miR-1750|2mut|as|	cagcaaaggatgacttcccgg	vvi-miR169a|compl_mismatch probe
vvi|miR-1751|nat|as|	tagcgaaggatgacttgccta	vvi-miR169y|compl_perfect match probe
vvi|miR-1751|2mut|as|	tagcaaaggatgacttcccta	vvi-miR169y|compl_mismatch probe
vvi|miR-1752|nat|as|	tgagccaaggatggcttgccg	vvi-miR169b|compl_perfect match probe
vvi|miR-1752|2mut|as|	tgagtcaaggatgccttgccg	vvi-miR169b|compl_mismatch probe
vvi|miR-1753|nat|as|	cagccaagaatgatttgccgg	vvi-miR169d|compl_perfect match probe
vvi|miR-1753|2mut|as|	cagcaaagaatgatttcccgg	vvi-miR169d|compl_mismatch probe
vvi|miR-1754|nat|as|	tagccaaggatgacttgcctg	vvi-miR169e|compl_perfect match probe
vvi|miR-1754|2mut|as|	tagcaaaggatgacttccctg	vvi-miR169e|compl_mismatch probe
vvi|miR-1755|nat|as|	cagccaaggatgacttgccga	vvi-miR169f|compl_perfect match probe
vvi|miR-1755|2mut|as|	cagcaaaggatgacttcccga	vvi-miR169f|compl_mismatch probe
vvi|miR-1756|nat|as|	cagccaaggatgacttgcc	vvi-miR169g|compl_perfect match probe
vvi|miR-1756|2mut|as|	cagcaaaggatgccttgcc	vvi-miR169g|compl_mismatch probe
vvi|miR-1757|nat|as|	gagccaaggatgactggccgt	vvi-miR169i|compl_perfect match probe
vvi|miR-1757|2mut|as|	gagcaaaggatgactgcccgt	vvi-miR169i|compl_mismatch probe
vvi|miR-1758|nat|as|	gagccaaggatgacttgccgt	vvi-miR169l|compl_perfect match probe
vvi|miR-1758|2mut|as|	gagcaaaggatgacttcccgt	vvi-miR169l|compl_mismatch probe
vvi|miR-1759|nat|as|	gagccaaggatgacttgccgg	vvi-miR169m|compl_perfect match

		probe
vvi|miR-1759|2mut|as|	gagcaaaggatgacttcccgg	vvi-miR169m|compl_mismatch probe
vvi|miR-1760|nat|as|	gagccaaggatgacttgccgc	vvi-miR169o|compl_perfect match probe
vvi|miR-1760|2mut|as|	gagcaaaggatgacttcccgc	vvi-miR169o|compl_mismatch probe
vvi|miR-1761|nat|as|	tgagtcaaggatgacttgccg	vvi-miR169r|compl_perfect match probe
vvi|miR-1761|2mut|as|	tgagtcaagaatgccttgccg	vvi-miR169r|compl_mismatch probe
vvi|miR-1762|nat|as|	cgagtcaaggatgacttgccg	vvi-miR169t|compl_perfect match probe
vvi|miR-1762|2mut|as|	cgagtcaagaatgccttgccg	vvi-miR169t|compl_mismatch probe
vvi|miR-1763|nat|as|	aagccaaggatgaattgccgg	vvi-miR169v|compl_perfect match probe
vvi|miR-1763|2mut|as|	aagcaaaggatgaattcccgg	vvi-miR169v|compl_mismatch probe
vvi|miR-1764|nat|as|	tagccaaggatgacttgccta	vvi-miR169x|compl_perfect match probe
vvi|miR-1764|2mut|as|	tagcaaaggatgacttcccta	vvi-miR169x|compl_mismatch probe
vvi|miR-1765|nat|as|	ttgagccgtcccaacatcacg	vvi-miR171a|compl_perfect match probe
vvi|miR-1765|2mut|as|	ttgagtcgtccaaacatcacg	vvi-miR171a|compl_mismatch probe
vvi|miR-1766|nat|as|	tgattgagccgcgtcaatatc	vvi-miR171b|compl_perfect match probe
vvi|miR-1766|2mut|as|	tgattgagcccagtcaatatc	vvi-miR171b|compl_mismatch probe
vvi|miR-1767|nat|as|	ttgagccgtgccaatatcacg	vvi-miR171c|compl_perfect match probe
vvi|miR-1767|2mut|as|	ttgagccgtgtaaatatcacg	vvi-miR171c|compl_mismatch probe
vvi|miR-1768|nat|as|	tgattgagccgcgccaatatc	vvi-miR171e|compl_perfect match probe
vvi|miR-1768|2mut|as|	tgattgagcccccccaatatc	vvi-miR171e|compl_mismatch probe
vvi|miR-1769|nat|as|	ttgagccgcgccaatatcact	vvi-miR171f|compl_perfect match probe
vvi|miR-1769|2mut|as|	ttgagccgcccaaatatcact	vvi-miR171f|compl_mismatch probe
vvi|miR-1770|nat|as|	ttgagccgaaccaatatcacc	vvi-miR171g|compl_perfect match probe

vvi|miR-1770|2mut|as| ttgagtcgaacaaatatcacc vvi-miR171g|compl_mismatch probe

vvi|miR-1771|nat|as| tggttgagccgcgccaatatc vvi-miR171h|compl_perfect match probe
vvi|miR-1771|2mut|as| tggttgagcccccccaatatc vvi-miR171h|compl_mismatch probe

vvi|miR-1772|nat|as| ttgagccgtgccaatatcatc vvi-miR171i|compl_perfect match probe
vvi|miR-1772|2mut|as| ttgagccgtgtaaatatcatc vvi-miR171i|compl_mismatch probe

vvi|miR-1773|nat|as| tgaatcttgatgatgctacat vvi-miR172a|compl_perfect match probe
vvi|miR-1773|2mut|as| tgaatcttaatgatcctacat vvi-miR172a|compl_mismatch probe

vvi|miR-1774|nat|as| tgaatcttgatgatgctacac vvi-miR172b|compl_perfect match probe
vvi|miR-1774|2mut|as| tgaatcttaatgatcctacac vvi-miR172b|compl_mismatch probe

vvi|miR-1775|nat|as| ggaatcttgatgatgctgcag vvi-miR172c|compl_perfect match probe
vvi|miR-1775|2mut|as| ggaatcttaatgatcctgcag vvi-miR172c|compl_mismatch probe

vvi|miR-1776|nat|as| agaatcttgatgatgctgcat vvi-miR172d|compl_perfect match probe
vvi|miR-1776|2mut|as| agaatcttaatgatcctgcat vvi-miR172d|compl_mismatch probe

vvi|miR-1777|nat|as| cttggactgaagggagctccc vvi-miR319b|compl_perfect match probe
vvi|miR-1777|2mut|as| cttgaactgaagtgagctccc vvi-miR319b|compl_mismatch probe

vvi|miR-1778|nat|as| tttggactgaagggagctcct vvi-miR319e|compl_perfect match probe
vvi|miR-1778|2mut|as| tttgaactgaagtgagctcct vvi-miR319e|compl_mismatch probe

vvi|miR-1779|nat|as| attggactgaagggagctccc vvi-miR319g|compl_perfect match probe
vvi|miR-1779|2mut|as| attgaactgaagtgagctccc vvi-miR319g|compl_mismatch probe

vvi|miR-1780|nat|as| aagctcaggagggatagcgcc vvi-miR390|compl_perfect match probe
vvi|miR-1780|2mut|as| aagctcagaagtgatagcgcc vvi-miR390|compl_mismatch probe

vvi|miR-1781|nat|as| tccaaagggatcgcattgatcc vvi-miR393a|compl_perfect match probe
vvi|miR-1781|2mut|as| tccaaagtgatcccattgatcc vvi-miR393a|compl_mismatch probe

vvi|miR-1782|nat|as|	ttggcattctgtccacctcc	vvi-miR394a|compl_perfect match probe
vvi|miR-1782|2mut|as|	ttggaattctgtcaacctcc	vvi-miR394a|compl_mismatch probe
vvi|miR-1783|nat|as|	ctgaagtgtttgggggaactc	vvi-miR395a|compl_perfect match probe
vvi|miR-1783|2mut|as|	ctgaagcgtttgggtgaactc	vvi-miR395a|compl_mismatch probe
vvi|miR-1784|nat|as|	ctgaagagtctggaggaactc	vvi-miR395n|compl_perfect match probe
vvi|miR-1784|2mut|as|	ctgaagagcctcgaggaactc	vvi-miR395n|compl_mismatch probe
vvi|miR-1785|nat|as|	ctcccacagctttcttgagctt	vvi-miR396a|compl_perfect match probe
vvi|miR-1785|2mut|as|	ctcccacagttttcttaagctt	vvi-miR396a|compl_mismatch probe
vvi|miR-1786|nat|as|	ttcccacagctttcttgaactt	vvi-miR396b|compl_perfect match probe
vvi|miR-1786|2mut|as|	ttcccacagttttcttaaactt	vvi-miR396b|compl_mismatch probe
vvi|miR-1787|nat|as|	ttccacagctttcttgaactg	vvi-miR396c|compl_perfect match probe
vvi|miR-1787|2mut|as|	ttccacagttttcttaaactg	vvi-miR396c|compl_mismatch probe
vvi|miR-1788|nat|as|	tcattgagtgcagcgttgatg	vvi-miR397a|compl_perfect match probe
vvi|miR-1788|2mut|as|	tcattgagtgaagagttgatg	vvi-miR397a|compl_mismatch probe
vvi|miR-1789|nat|as|	tgtgttctcaggtcacccctt	vvi-miR398a|compl_perfect match probe
vvi|miR-1789|2mut|as|	tgtgttctcacgtcactcctt	vvi-miR398a|compl_mismatch probe
vvi|miR-1790|nat|as|	tgtgttctcaggtcgcccctg	vvi-miR398b|compl_perfect match probe
vvi|miR-1790|2mut|as|	tgtgttctcacgtccccccctg	vvi-miR398b|compl_mismatch probe
vvi|miR-1791|nat|as|	tgccaaaggagaattgccctg	vvi-miR399a|compl_perfect match probe
vvi|miR-1791|2mut|as|	tgccaaagaagaattgtcctg	vvi-miR399a|compl_mismatch probe
vvi|miR-1792|nat|as|	tgccaaaggagagttgccctg	vvi-miR399b|compl_perfect match probe
vvi|miR-1792|2mut|as|	tgccaaagaagagttgtcctg	vvi-miR399b|compl_mismatch probe
vvi|miR-1793|nat|as|	tgccaaaggagatttgctcgt	vvi-miR399d|compl_perfect match

		probe
vvi|miR-1793|2mut|as|	tgccaaagaagatttcctcgt	vvi-miR399d|compl_mismatch probe
vvi|miR-1794|nat|as|	tgccaaaggagatttgcccgg	vvi-miR399e|compl_perfect match probe
vvi|miR-1794|2mut|as|	tgccaaagaagatttgtccgg	vvi-miR399e|compl_mismatch probe
vvi|miR-1795|nat|as|	tgccgaaggagatttgtcctg	vvi-miR399f|compl_perfect match probe
vvi|miR-1795|2mut|as|	tgccaaaggagatttctcctg	vvi-miR399f|compl_mismatch probe
vvi|miR-1796|nat|as|	tgccaaaggagatttgcccct	vvi-miR399g|compl_perfect match probe
vvi|miR-1796|2mut|as|	tgccaaagaagatttgtccct	vvi-miR399g|compl_mismatch probe
vvi|miR-1797|nat|as|	cgccaaaggagagttgccctg	vvi-miR399i|compl_perfect match probe
vvi|miR-1797|2mut|as|	cgccaaagaagagttgtcctg	vvi-miR399i|compl_mismatch probe
vvi|miR-1798|nat|as|	ttagattcacgcacaaactcg	vvi-miR403a|compl_perfect match probe
vvi|miR-1798|2mut|as|	ttagattcaccaacaaactcg	vvi-miR403a|compl_mismatch probe
vvi|miR-1799|nat|as|	atgcactgcctcttccctggc	vvi-miR408|compl_perfect match probe
vvi|miR-1799|2mut|as|	atgcactgtctcttctctggc	vvi-miR408|compl_mismatch probe
vvi|miR-1800|nat|as|	atctccctcaaaggcttccaa	vvi-miR477a|compl_perfect match probe
vvi|miR-1800|2mut|as|	atctctctcaaagccttccaa	vvi-miR477a|compl_mismatch probe
vvi|miR-1801|nat|as|	tgtggtattggttcggctcatc	vvi-miR479|compl_perfect match probe
vvi|miR-1801|2mut|as|	tgtggtattcgttcgcctcatc	vvi-miR479|compl_mismatch probe
vvi|miR-1802|nat|as|	cctactcctcccattcc	vvi-miR482a|compl_perfect match probe
vvi|miR-1802|2mut|as|	cctacttctctcattcc	vvi-miR482a|compl_mismatch probe
vvi|miR-1803|nat|as|	tgacaacgagagagagcacgct	vvi-miR535a|compl_perfect match probe
vvi|miR-1803|2mut|as|	tgacaaagagagagagaacgct	vvi-miR535a|compl_mismatch probe
vvi|miR-1804|nat|as|	tcttgctcaaatgagtattcca	vvi-miR828a|compl_perfect match probe

<table>
<tr><td>vvi|miR-1804|2mut|as|</td><td>tcttcctcaaataagtattcca</td><td>vvi-miR828a|compl_mismatch probe</td></tr>
<tr><td>vvi|miR-1805|nat|as|</td><td>tcttgctcaaatgagtgttcca</td><td>vvi-miR828b|compl_perfect match probe</td></tr>
<tr><td>vvi|miR-1805|2mut|as|</td><td>tcttcctcaaatgagcgttcca</td><td>vvi-miR828b|compl_mismatch probe</td></tr>
<tr><td>vvi|miR-1806|nat|as|</td><td>tagctctgataccaattgata</td><td>vvi-miR845a|compl_perfect match probe</td></tr>
<tr><td>vvi|miR-1806|2mut|as|</td><td>tagctccgatacaaattgata</td><td>vvi-miR845a|compl_mismatch probe</td></tr>
<tr><td>vvi|miR-1807|nat|as|</td><td>aggctctgataccaattgatg</td><td>vvi-miR845c|compl_perfect match probe</td></tr>
<tr><td>vvi|miR-1807|2mut|as|</td><td>aggctccgatacaaattgatg</td><td>vvi-miR845c|compl_mismatch probe</td></tr>
<tr><td>vvi|miR-1808|nat|as|</td><td>tggctctgataccaattgatg</td><td>vvi-miR845d|compl_perfect match probe</td></tr>
<tr><td>vvi|miR-1808|2mut|as|</td><td>tggctccgatacaaattgatg</td><td>vvi-miR845d|compl_mismatch probe</td></tr>
<tr><td>vvi|miR-1809|nat|as|</td><td>gtgctctctcttcttctgtca</td><td>vvi-miR156a|*compl_perfect match probe</td></tr>
<tr><td>vvi|miR-1809|2mut|as|</td><td>gtgctctcccttcttccgtca</td><td>vvi-miR156a|*compl_mismatch probe</td></tr>
<tr><td>vvi|miR-1810|nat|as|</td><td>gtgctcatttctctttctgtca</td><td>vvi-miR156b|*compl_perfect match probe</td></tr>
<tr><td>vvi|miR-1810|2mut|as|</td><td>gtgctaatttctctttccgtca</td><td>vvi-miR156b|*compl_mismatch probe</td></tr>
<tr><td>vvi|miR-1811|nat|as|</td><td>gtgctcactctctatctgtca</td><td>vvi-miR156c|*compl_perfect match probe</td></tr>
<tr><td>vvi|miR-1811|2mut|as|</td><td>gtgctccctctctatccgtca</td><td>vvi-miR156c|*compl_mismatch probe</td></tr>
<tr><td>vvi|miR-1812|nat|as|</td><td>gtgctcacctctctttctgtca</td><td>vvi-miR156d|*compl_perfect match probe</td></tr>
<tr><td>vvi|miR-1812|2mut|as|</td><td>gtgctcatctctctttccgtca</td><td>vvi-miR156d|*compl_mismatch probe</td></tr>
<tr><td>vvi|miR-1813|nat|as|</td><td>gtgcttactccctatctgtca</td><td>vvi-miR156e|*compl_perfect match probe</td></tr>
<tr><td>vvi|miR-1813|2mut|as|</td><td>gtgcttactctttatctgtca</td><td>vvi-miR156e|*compl_mismatch probe</td></tr>
<tr><td>vvi|miR-1814|nat|as|</td><td>gtcctctctcttctcctgtcaa</td><td>vvi-miR156f|*compl_perfect match</td></tr>
</table>

		probe
vvi|miR-1814|2mut|as|	gtcctctcccttcttctgtcaa	vvi-miR156f|*compl_mismatch probe
vvi|miR-1815|nat|as|	gtgctctctagacttctgtca	vvi-miR156g|*compl_perfect match probe
vvi|miR-1815|2mut|as|	gtgctccctagccttctgtca	vvi-miR156g|*compl_mismatch probe
vvi|miR-1816|nat|as|	gttctttctgcgcctttca	vvi-miR156h|*compl_perfect match probe
vvi|miR-1816|2mut|as|	gttctttctccccctttca	vvi-miR156h|*compl_mismatch probe
vvi|miR-1817|nat|as|	gtgctctctattcttctgtca	vvi-miR156i|*compl_perfect match probe
vvi|miR-1817|2mut|as|	gtgctccctattcttccgtca	vvi-miR156i|*compl_mismatch probe
vvi|miR-1818|nat|as|	gggagctcctttacgctccag	vvi-miR159a|*compl_perfect match probe
vvi|miR-1818|2mut|as|	gggagttcctttaccctccag	vvi-miR159a|*compl_mismatch probe
vvi|miR-1819|nat|as|	tgggagcttctttacactccag	vvi-miR159b|*compl_perfect match probe
vvi|miR-1819|2mut|as|	tgggagtttctttaccctccag	vvi-miR159b|*compl_mismatch probe
vvi|miR-1820|nat|as|	tggagctccttgaagtccaa	vvi-miR159c|*compl_perfect match probe
vvi|miR-1820|2mut|as|	tggagttccttgaagcccaa	vvi-miR159c|*compl_mismatch probe
vvi|miR-1821|nat|as|	tggcatcagaggagtcatgca	vvi-miR160a|*compl_perfect match probe
vvi|miR-1821|2mut|as|	tggcatcagacaagtcatgca	vvi-miR160a|*compl_mismatch probe
vvi|miR-1822|nat|as|	tggcatgaggggagtcaagca	vvi-miR160b|*compl_perfect match probe
vvi|miR-1822|2mut|as|	tggcatgaggtaagtcaagca	vvi-miR160b|*compl_mismatch probe
vvi|miR-1823|nat|as|	tggcgtgcgaggagccaagca	vvi-miR160c|*compl_perfect match

		probe
vvi|miR-1823|2mut|as|	tggcgtgagaggagtcaagca	vvi-miR160c|*compl_mismatch probe
vvi|miR-1824|nat|as|	tggcgtgcgaggagccatgca	vvi-miR160d|*compl_perfect match probe
vvi|miR-1824|2mut|as|	tggcgtgagaggagtcatgca	vvi-miR160d|*compl_mismatch probe
vvi|miR-1825|nat|as|	tggcatgaggggagtcaagca	vvi-miR160e|*compl_perfect match probe
vvi|miR-1825|2mut|as|	tggcatgaggtaagtcaagca	vvi-miR160e|*compl_mismatch probe
vvi|miR-1826|nat|as|	tggcgtatgaggagccatgca	vvi-miR160f|*compl_perfect match probe
vvi|miR-1826|2mut|as|	tggcctatgaggagtcatgca	vvi-miR160f|*compl_mismatch probe
vvi|miR-1827|nat|as|	ctggatgcagcggttcatcga	vvi-miR162|*compl_perfect match probe
vvi|miR-1827|2mut|as|	ctggatgcagaagttcatcga	vvi-miR162|*compl_mismatch probe
vvi|miR-1828|nat|as|	gcacgtgctccccttctcca	vvi-miR164a|*compl_perfect match probe
vvi|miR-1828|2mut|as|	gcacgtcctcctcttctcca	vvi-miR164a|*compl_mismatch probe
vvi|miR-1829|nat|as|	catgtgccctggctctcc	vvi-miR164b|*compl_perfect match probe
vvi|miR-1829|2mut|as|	catgtgtcctgcctctcc	vvi-miR164b|*compl_mismatch probe
vvi|miR-1830|nat|as|	tgcacgtgctccccttctcca	vvi-miR164d|*compl_perfect match probe
vvi|miR-1830|2mut|as|	tgcacgtcctcctcttctcca	vvi-miR164d|*compl_mismatch probe
vvi|miR-1831|nat|as|	ggggaatgttgtctggctcga	vvi-miR166c|*compl_perfect match probe
vvi|miR-1831|2mut|as|	ggggaatgttctctgcctcga	vvi-miR166c|*compl_mismatch probe
vvi|miR-1832|nat|as|	aggggattgttgtctggctcga	vvi-miR166d|*compl_perfect match

		probe
		vvi-miR166d|*compl_mismatch
vvi|miR-1832|2mut|as|	agggaattgttgtctgcctcga	probe
		vvi-miR166e|*compl_perfect match
vvi|miR-1833|nat|as|	ggggaatgttgtctggctcga	probe
		vvi-miR166e|*compl_mismatch
vvi|miR-1833|2mut|as|	ggggaatgttctctgcctcga	probe
		vvi-miR166f|*compl_perfect match
vvi|miR-1834|nat|as|	ggggaatgttggctggctcga	probe
vvi|miR-1834|2mut|as|	ggggaatgttcgctgcctcga	vvi-miR166f|*compl_mismatch probe
		vvi-miR166g|*compl_perfect match
vvi|miR-1835|nat|as|	aggggaatgttgtctggttcga	probe
		vvi-miR166g|*compl_mismatch
vvi|miR-1835|2mut|as|	agggaaatgttgtctcgttcga	probe
		vvi-miR166h|*compl_perfect match
vvi|miR-1836|nat|as|	ggggaacgctgtctggttcga	probe
		vvi-miR166h|*compl_mismatch
vvi|miR-1836|2mut|as|	ggggaaccctgtctcgttcga	probe
		vvi-miR166a|*compl_perfect match
vvi|miR-1837|nat|as|	ttggaatgaggtttgatccaa	probe
		vvi-miR166a|*compl_mismatch
vvi|miR-1837|2mut|as|	ttggaatgagctttaatccaa	probe
		vvi-miR166b|*compl_perfect match
vvi|miR-1838|nat|as|	ggggaatgttggctggctcga	probe
		vvi-miR166b|*compl_mismatch
vvi|miR-1838|2mut|as|	ggggaatgttcgctgcctcga	probe
		vvi-miR167a|*compl_perfect match
vvi|miR-1839|nat|as|	caggtcatcttgcagcttca	probe
		vvi-miR167a|*compl_mismatch
vvi|miR-1839|2mut|as|	caggccatcttgaagcttca	probe
		vvi-miR167b|*compl_perfect match
vvi|miR-1840|nat|as|	tagatcatgtggcagtttca	probe
		vvi-miR167b|*compl_mismatch
vvi|miR-1840|2mut|as|	tagatcatgcgccagtttca	probe

vvi|miR-1841|nat|as|	agatcatgtggtagcctca	vvi-miR167c|*compl_perfect match probe
vvi|miR-1841|2mut|as|	agatcatgtcgtagtctca	vvi-miR167c|*compl_mismatch probe
vvi|miR-1842|nat|as|	taggtcatgccctgacagcctca	vvi-miR167d|*compl_perfect match probe
vvi|miR-1842|2mut|as|	taggtcatgtcctgacagtctca	vvi-miR167d|*compl_mismatch probe
vvi|miR-1843|nat|as|	agatcatgtggcagtttca	vvi-miR167e|*compl_perfect match probe
vvi|miR-1843|2mut|as|	agatcatgtcccagtttca	vvi-miR167e|*compl_mismatch probe
vvi|miR-1844|nat|as|	tcccgccttgcatcaactga	vvi-miR168|*compl_perfect match probe
vvi|miR-1844|2mut|as|	tcccccccttgaatcaactga	vvi-miR168|*compl_mismatch probe
vvi|miR-1845|nat|as|	ctggcaagttgtccttggct	vvi-miR169a|*compl_perfect match probe
vvi|miR-1845|2mut|as|	ctggaaagttgttcttggct	vvi-miR169a|*compl_mismatch probe
vvi|miR-1846|nat|as|	ggcaagtctccttggca	vvi-miR169y|*compl_perfect match probe
vvi|miR-1846|2mut|as|	ggcaagccttcttggca	vvi-miR169y|*compl_mismatch probe
vvi|miR-1847|nat|as|	cggttagtcatccttggctc	vvi-miR169b|*compl_perfect match probe
vvi|miR-1847|2mut|as|	cggttagccatccttcgctc	vvi-miR169b|*compl_mismatch probe
vvi|miR-1848|nat|as|	ccggcaagttgtccttggct	vvi-miR169c|*compl_perfect match probe
vvi|miR-1848|2mut|as|	ccggaaagttgttcttggct	vvi-miR169c|*compl_mismatch probe
vvi|miR-1849|nat|as|	ctggcaagttgtccttggct	vvi-miR169d|*compl_perfect match probe
vvi|miR-1849|2mut|as|	ctggaaagttgttcttggct	vvi-miR169d|*compl_mismatch

		probe
		vvi-miR169e|*compl_perfect match
vvi|miR-1850|nat|as|	caggcagtcaccttggctaa	probe
		vvi-miR169e|*compl_mismatch
vvi|miR-1850|2mut|as|	caggaagtcaccttgcctaa	probe
		vvi-miR169f|*compl_perfect match
vvi|miR-1851|nat|as|	ggcaagttgtgtttggct	probe
vvi|miR-1851|2mut|as|	ggcaagttccgtttggct	vvi-miR169f|*compl_mismatch probe
		vvi-miR169g|*compl_perfect match
vvi|miR-1852|nat|as|	ggcaagttgtctttggct	probe
		vvi-miR169g|*compl_mismatch
vvi|miR-1852|2mut|as|	ggcaagttccctttggct	probe
		vvi-miR169h|*compl_perfect match
vvi|miR-1853|nat|as|	cggagggttatccttgactc	probe
		vvi-miR169h|*compl_mismatch
vvi|miR-1853|2mut|as|	cggagtgttattcttgactc	probe
		vvi-miR169i|*compl_perfect match
vvi|miR-1854|nat|as|	acggctggttatccctgtctc	probe
vvi|miR-1854|2mut|as|	acggttggttatctctgtctc	vvi-miR169i|*compl_mismatch probe
		vvi-miR169j|*compl_perfect match
vvi|miR-1855|nat|as|	ctggcaggttgtccttggct	probe
vvi|miR-1855|2mut|as|	ctggaaggttgttcttggct	vvi-miR169j|*compl_mismatch probe
		vvi-miR169k|*compl_perfect match
vvi|miR-1856|nat|as|	ctggcaggttgtccttggct	probe
		vvi-miR169k|*compl_mismatch
vvi|miR-1856|2mut|as|	ctggaaggttgttcttggct	probe
		vvi-miR169l|*compl_perfect match
vvi|miR-1857|nat|as|	acggctagttattcttggctc	probe
vvi|miR-1857|2mut|as|	acggttagttattcttcgctc	vvi-miR169l|*compl_mismatch probe
		vvi-miR169m|*compl_perfect match
vvi|miR-1858|nat|as|	ctggcaagcatccgaggctc	probe
		vvi-miR169m|*compl_mismatch
vvi|miR-1858|2mut|as|	ctggcaagaatcagaggctc	probe
vvi|miR-1859|nat|as|	cataactggcaagcatctgaggctc	vvi-miR169n|*compl_perfect match

		probe
vvi|miR-1859|2mut|as|	cataactgccaagaatctgaggctc	vvi-miR169n|*compl_mismatch probe
vvi|miR-1860|nat|as|	gacagcgagttattcttggctc	vvi-miR169o|*compl_perfect match probe
vvi|miR-1860|2mut|as|	gacagagagttattcttcgctc	vvi-miR169o|*compl_mismatch probe
vvi|miR-1861|nat|as|	ctggcaagcatccgaggctc	vvi-miR169p|*compl_perfect match probe
vvi|miR-1861|2mut|as|	ctggcaagaatcagaggctc	vvi-miR169p|*compl_mismatch probe
vvi|miR-1862|nat|as|	ccggcatgtaatcctggctc	vvi-miR169q|*compl_perfect match probe
vvi|miR-1862|2mut|as|	ccggaatgtaatcctcgctc	vvi-miR169q|*compl_mismatch probe
vvi|miR-1863|nat|as|	cggcaagttgacttgactca	vvi-miR169r|*compl_perfect match probe
vvi|miR-1863|2mut|as|	cggcaagttaacttgcctca	vvi-miR169r|*compl_mismatch probe
vvi|miR-1864|nat|as|	ccggcaagttgttcctggct	vvi-miR169s|*compl_perfect match probe
vvi|miR-1864|2mut|as|	ccggaaagttgtttctggct	vvi-miR169s|*compl_mismatch probe
vvi|miR-1865|nat|as|	cggcaagttgacttgactc	vvi-miR169t|*compl_perfect match probe
vvi|miR-1865|2mut|as|	cggcaagttaccttgactc	vvi-miR169t|*compl_mismatch probe
vvi|miR-1866|nat|as|	cggcaagttgacttgactc	vvi-miR169u|*compl_perfect match probe
vvi|miR-1866|2mut|as|	cggcaagttaccttgactc	vvi-miR169u|*compl_mismatch probe
vvi|miR-1867|nat|as|	tcggcaatttatttcttggct	vvi-miR169v|*compl_perfect match probe
vvi|miR-1867|2mut|as|	tcggaaatttattttttggct	vvi-miR169v|*compl_mismatch probe
vvi|miR-1868|nat|as|	tcggcgagtcattcttagct	vvi-miR169w|*compl_perfect match

		probe
vvi|miR-1868|2mut|as|	tcggagagtcatttttagct	vvi-miR169w|*compl_mismatch probe
vvi|miR-1869|nat|as|	taggcagtcattccttcggcta	vvi-miR169x|*compl_perfect match probe
vvi|miR-1869|2mut|as|	taggaagtcattccttcagcta	vvi-miR169x|*compl_mismatch probe
vvi|miR-1870|nat|as|	catgatattggcacggctcaa	vvi-miR171a|*compl_perfect match probe
vvi|miR-1870|2mut|as|	catgatattgccacgcctcaa	vvi-miR171a|*compl_mismatch probe
vvi|miR-1871|nat|as|	ggtattggcgtgcctcaatt	vvi-miR171b|*compl_perfect match probe
vvi|miR-1871|2mut|as|	ggtattggagtgtctcaatt	vvi-miR171b|*compl_mismatch probe
vvi|miR-1872|nat|as|	cgggatattggtgcggttcaa	vvi-miR171c|*compl_perfect match probe
vvi|miR-1872|2mut|as|	cgggatattcgtgaggttcaa	vvi-miR171c|*compl_mismatch probe
vvi|miR-1873|nat|as|	cgagatattgatacggttcaa	vvi-miR171d|*compl_perfect match probe
vvi|miR-1873|2mut|as|	cgagatattaatacagttcaa	vvi-miR171d|*compl_mismatch probe
vvi|miR-1874|nat|as|	gatgttggtgaggttcaatc	vvi-miR171e|*compl_perfect match probe
vvi|miR-1874|2mut|as|	gatgttcgtgagcttcaatc	vvi-miR171e|*compl_mismatch probe
vvi|miR-1875|nat|as|	agcgatgttggtgaggttcaa	vvi-miR171f|*compl_perfect match probe
vvi|miR-1875|2mut|as|	agcgatgttcgtgagcttcaa	vvi-miR171f|*compl_mismatch probe
vvi|miR-1876|nat|as|	atgttggttccatcgg	vvi-miR171g|*compl_perfect match probe
vvi|miR-1876|2mut|as|	atgttcgttcaatcgg	vvi-miR171g|*compl_mismatch probe

vvi|miR-1877|nat|as|	gatgttggtgcggttcaacc	vvi-miR171h|*compl_perfect match probe
vvi|miR-1877|2mut|as|	gatgttggtcaggttcaacc	vvi-miR171h|*compl_mismatch probe
vvi|miR-1878|nat|as|	ggggatgttggaatggctcaa	vvi-miR171i|*compl_perfect match probe
vvi|miR-1878|2mut|as|	ggggatgttcgaatgcctcaa	vvi-miR171i|*compl_mismatch probe
vvi|miR-1879|nat|as|	atgcagcatcatcaagattc	vvi-miR172a|*compl_perfect match probe
vvi|miR-1879|2mut|as|	atgcagaatcataaagattc	vvi-miR172a|*compl_mismatch probe
vvi|miR-1880|nat|as|	tgcagcatcatcaagattc	vvi-miR172b|*compl_perfect match probe
vvi|miR-1880|2mut|as|	tgcagaatcataaagattc	vvi-miR172b|*compl_mismatch probe
vvi|miR-1881|nat|as|	tggagcatcatcaagattc	vvi-miR172c|*compl_perfect match probe
vvi|miR-1881|2mut|as|	tggagaatcataaagattc	vvi-miR172c|*compl_mismatch probe
vvi|miR-1882|nat|as|	atgcagcatcatcaagattc	vvi-miR172d|*compl_perfect match probe
vvi|miR-1882|2mut|as|	atgcagaatcataaagattc	vvi-miR172d|*compl_mismatch probe
vvi|miR-1883|nat|as|	ggagctctctttagtccag	vvi-miR319b|*compl_perfect match probe
vvi|miR-1883|2mut|as|	ggagttctctttagcccag	vvi-miR319b|*compl_mismatch probe
vvi|miR-1884|nat|as|	gagagctttcttcagtcca	vvi-miR319c|*compl_perfect match probe
vvi|miR-1884|2mut|as|	gagagttttcttccgtcca	vvi-miR319c|*compl_mismatch probe
vvi|miR-1885|nat|as|	gggggttcctttgcagcccaaa	vvi-miR319e|*compl_perfect match probe
vvi|miR-1885|2mut|as|	ggggcttcctttgcagtccaaa	vvi-miR319e|*compl_mismatch

		probe
		vvi-miR319f|*compl_perfect match
vvi|miR-1886|nat|as|	gagagcttccttcagtcca	probe
vvi|miR-1886|2mut|as|	gagagtttccttccgtcca	vvi-miR319f|*compl_mismatch probe
		vvi-miR319g|*compl_perfect match
vvi|miR-1887|nat|as|	gagctccttttagtccaat	probe
		vvi-miR319g|*compl_mismatch
vvi|miR-1887|2mut|as|	gagcttcttttagttcaat	probe
		vvi-miR390|*compl_perfect match
vvi|miR-1888|nat|as|	ggcgctatctatcctgagttt	probe
vvi|miR-1888|2mut|as|	ggcgttatctattctgagttt	vvi-miR390|*compl_mismatch probe
		vvi-miR393a|*compl_perfect match
vvi|miR-1889|nat|as|	ggatcatgctatcccttagga	probe
		vvi-miR393a|*compl_mismatch
vvi|miR-1889|2mut|as|	ggatcatcctatctcttagga	probe
		vvi-miR393b|*compl_perfect match
vvi|miR-1890|nat|as|	ggatcatgctatccctttgga	probe
		vvi-miR393b|*compl_mismatch
vvi|miR-1890|2mut|as|	ggatcatcctatctctttgga	probe
		vvi-miR394a|*compl_perfect match
vvi|miR-1891|nat|as|	ggaggtggccagcatgccaa	probe
		vvi-miR394a|*compl_mismatch
vvi|miR-1891|2mut|as|	ggaggtggtcagcatcccaa	probe
		vvi-miR394b|*compl_perfect match
vvi|miR-1892|nat|as|	ggaggtgggcatactgccaa	probe
		vvi-miR394b|*compl_mismatch
vvi|miR-1892|2mut|as|	ggaggtggccatactcccaa	probe
		vvi-miR394c|*compl_perfect match
vvi|miR-1893|nat|as|	ggaggcggccaggatgccaa	probe
		vvi-miR394c|*compl_mismatch
vvi|miR-1893|2mut|as|	ggaggaggccaggatcccaa	probe
		vvi-miR395a|*compl_perfect match
vvi|miR-1894|nat|as|	gagttcccttgatcacttca	probe
vvi|miR-1894|2mut|as|	gagttctcttgatcccttca	vvi-miR395a|*compl_mismatch

		probe
vvi|miR-1895|nat|as|	gagttccccttaccacttca	vvi-miR395b|*compl_perfect match probe
vvi|miR-1895|2mut|as|	gagttcctcttacaacttca	vvi-miR395b|*compl_mismatch probe
vvi|miR-1896|nat|as|	gagttcccttgaccacttca	vvi-miR395c|*compl_perfect match probe
vvi|miR-1896|2mut|as|	gagttctcttgacaacttca	vvi-miR395c|*compl_mismatch probe
vvi|miR-1897|nat|as|	gagttcccctgaccacttca	vvi-miR395d|*compl_perfect match probe
vvi|miR-1897|2mut|as|	gagttcctctgacaacttca	vvi-miR395d|*compl_mismatch probe
vvi|miR-1898|nat|as|	gagttcccttgaccacttca	vvi-miR395e|*compl_perfect match probe
vvi|miR-1898|2mut|as|	gagttctcttgacaacttca	vvi-miR395e|*compl_mismatch probe
vvi|miR-1899|nat|as|	gagttcccctgaccacttca	vvi-miR395f|*compl_perfect match probe
vvi|miR-1899|2mut|as|	gagttcctctgacaacttca	vvi-miR395f|*compl_mismatch probe
vvi|miR-1900|nat|as|	gagttcccctgagcacttca	vvi-miR395g|*compl_perfect match probe
vvi|miR-1900|2mut|as|	gagttcctctgagaacttca	vvi-miR395g|*compl_mismatch probe
vvi|miR-1901|nat|as|	gagttcccttgaccacttca	vvi-miR395h|*compl_perfect match probe
vvi|miR-1901|2mut|as|	gagttctcttgacaacttca	vvi-miR395h|*compl_mismatch probe
vvi|miR-1902|nat|as|	gagttcccctgaccacttca	vvi-miR395i|*compl_perfect match probe
vvi|miR-1902|2mut|as|	gagttcctctgacaacttca	vvi-miR395i|*compl_mismatch probe
vvi|miR-1903|nat|as|	gagttcccctgaccacttca	vvi-miR395j|*compl_perfect match probe
vvi|miR-1903|2mut|as|	gagttcctctgacaacttca	vvi-miR395j|*compl_mismatch probe

vvi|miR-1904|nat|as|	gagttcccttgaccacttca	vvi-miR395k|*compl_perfect match probe
vvi|miR-1904|2mut|as|	gagttctcttgacaacttca	vvi-miR395k|*compl_mismatch probe
vvi|miR-1905|nat|as|	gagttcccctgaccacttca	vvi-miR395l|*compl_perfect match probe
vvi|miR-1905|2mut|as|	gagttcctctgacaacttca	vvi-miR395l|*compl_mismatch probe
vvi|miR-1906|nat|as|	gagttcccttgaacacttca	vvi-miR395m|*compl_perfect match probe
vvi|miR-1906|2mut|as|	gagttctcttgaacccttca	vvi-miR395m|*compl_mismatch probe
vvi|miR-1907|nat|as|	gagttcccccaacccctccag	vvi-miR395n|*compl_perfect match probe
vvi|miR-1907|2mut|as|	gagttccctcaacctctccag	vvi-miR395n|*compl_mismatch probe
vvi|miR-1908|nat|as|	agttcaagaaagctgtgg	vvi-miR396a|*compl_perfect match probe
vvi|miR-1908|2mut|as|	agttaaagaaagttgtgg	vvi-miR396a|*compl_mismatch probe
vvi|miR-1909|nat|as|	aagttcaagaaagctgtggaaa	vvi-miR396b|*compl_perfect match probe
vvi|miR-1909|2mut|as|	aagttaaagaaagttgtggaaa	vvi-miR396b|*compl_mismatch probe
vvi|miR-1910|nat|as|	cagctcaggaaagttgtggaa	vvi-miR396c|*compl_perfect match probe
vvi|miR-1910|2mut|as|	cagctcagaaaagttgcggaa	vvi-miR396c|*compl_mismatch probe
vvi|miR-1911|nat|as|	cggttcaataaagctgtggga	vvi-miR396d|*compl_perfect match probe
vvi|miR-1911|2mut|as|	cggttaaataaagttgtggga	vvi-miR396d|*compl_mismatch probe
vvi|miR-1912|nat|as|	cattggcgctgcactcaatca	vvi-miR397a|*compl_perfect match probe
vvi|miR-1912|2mut|as|	cattggccctgaactcaatca	vvi-miR397a|*compl_mismatch

		probe
vvi|miR-1913|nat|as|	cattggcgctgcactcaatca	vvi-miR397b|*compl_perfect match probe
vvi|miR-1913|2mut|as|	cattggccctgaactcaatca	vvi-miR397b|*compl_mismatch probe
vvi|miR-1914|nat|as|	agggagtggcacctgagaacac	vvi-miR398a|*compl_perfect match probe
vvi|miR-1914|2mut|as|	agggagtgccatctgagaacac	vvi-miR398a|*compl_mismatch probe
vvi|miR-1915|nat|as|	caggtgtgacctgagaatcaca	vvi-miR398b|*compl_perfect match probe
vvi|miR-1915|2mut|as|	caggtgtgatccgagaatcaca	vvi-miR398b|*compl_mismatch probe
vvi|miR-1916|nat|as|	caggagtgacctgagaatcaca	vvi-miR398c|*compl_perfect match probe
vvi|miR-1916|2mut|as|	caggagtgatccgagaatcaca	vvi-miR398c|*compl_mismatch probe
vvi|miR-1917|nat|as|	cagtgtgattctcctttggca	vvi-miR399a|*compl_perfect match probe
vvi|miR-1917|2mut|as|	cagtgcgattcttctttggca	vvi-miR399a|*compl_mismatch probe
vvi|miR-1918|nat|as|	tagggcacctctttcttggca	vvi-miR399b|*compl_perfect match probe
vvi|miR-1918|2mut|as|	taggccacctcttttttggca	vvi-miR399b|*compl_mismatch probe
vvi|miR-1919|nat|as|	cagggcctctttcacttggta	vvi-miR399c|*compl_perfect match probe
vvi|miR-1919|2mut|as|	cagggtctctttcccttggta	vvi-miR399c|*compl_mismatch probe
vvi|miR-1920|nat|as|	gagcagatttcttttggca	vvi-miR399d|*compl_perfect match probe
vvi|miR-1920|2mut|as|	gagccgattttttttggca	vvi-miR399d|*compl_mismatch probe
vvi|miR-1921|nat|as|	gggcaaattatcttttggca	vvi-miR399e|*compl_perfect match

		probe
vvi|miR-1921|2mut|as|	gggccaattattttttggca	vvi-miR399e|*compl_mismatch probe
vvi|miR-1922|nat|as|	cattagagggccaaatctgctttggca	vvi-miR399f|*compl_perfect match probe
vvi|miR-1922|2mut|as|	cattagagggtcaaatctcctttggca	vvi-miR399f|*compl_mismatch probe
vvi|miR-1923|nat|as|	gggcaatactccattggca	vvi-miR399g|*compl_perfect match probe
vvi|miR-1923|2mut|as|	gggcaatattcaattggca	vvi-miR399g|*compl_mismatch probe
vvi|miR-1924|nat|as|	cagtgcaatcctcctttggca	vvi-miR399h|*compl_perfect match probe
vvi|miR-1924|2mut|as|	cagtgaaatccttctttggca	vvi-miR399h|*compl_mismatch probe
vvi|miR-1925|nat|as|	tagggcttctctccttctggc	vvi-miR399i|*compl_perfect match probe
vvi|miR-1925|2mut|as|	taggccttctcttcttctggc	vvi-miR399i|*compl_mismatch probe
vvi|miR-1926|nat|as|	agtttgtgcgtgaatcaaa	vvi-miR403a|*compl_perfect match probe
vvi|miR-1926|2mut|as|	agtttgtgagcgaatcaaa	vvi-miR403a|*compl_mismatch probe
vvi|miR-1927|nat|as|	cgagtttgtgcgcgaatccaa	vvi-miR403b|*compl_perfect match probe
vvi|miR-1927|2mut|as|	cgagtttgtgagagaatccaa	vvi-miR403b|*compl_mismatch probe
vvi|miR-1928|nat|as|	caagtttgtgcgtgaatcaaa	vvi-miR403c|*compl_perfect match probe
vvi|miR-1928|2mut|as|	caagtttgtgactgaatcaaa	vvi-miR403c|*compl_mismatch probe
vvi|miR-1929|nat|as|	cgagtttgtgcgcgaatc	vvi-miR403d|*compl_perfect match probe
vvi|miR-1929|2mut|as|	cgagtttgcgcgagaatc	vvi-miR403d|*compl_mismatch probe
vvi|miR-1930|nat|as|	caagtttgtgcgtgaatcaaa	vvi-miR403e|*compl_perfect match

		probe
vvi|miR-1930|2mut|as|	caagtttgtgactgaatcaaa	vvi-miR403e|*compl_mismatch probe
vvi|miR-1931|nat|as|	gagtttgtgcgtgactctaa	vvi-miR403f|*compl_perfect match probe
vvi|miR-1931|2mut|as|	gagtttgtgactgactctaa	vvi-miR403f|*compl_mismatch probe
vvi|miR-1932|nat|as|	cggggacgaggtagtgcat	vvi-miR408|*compl_perfect match probe
vvi|miR-1932|2mut|as|	cggggaagagctagtgcat	vvi-miR408|*compl_mismatch probe
vvi|miR-1933|nat|as|	ttggaagacagtgggggacct	vvi-miR477a|*compl_perfect match probe
vvi|miR-1933|2mut|as|	ttggaagaaagtgggtgacct	vvi-miR477a|*compl_mismatch probe
vvi|miR-1934|nat|as|	gacgagccgaaccaatatcac	vvi-miR479|*compl_perfect match probe
vvi|miR-1934|2mut|as|	gacgagtcgaacaaatatcac	vvi-miR479|*compl_mismatch probe
vvi|miR-1935|nat|as|	ggaattggagagtagg	vvi-miR482a|*compl_perfect match probe
vvi|miR-1935|2mut|as|	ggaattcgagactagg	vvi-miR482a|*compl_mismatch probe
vvi|miR-1936|nat|as|	ggcgtgctctctctcgctgtca	vvi-miR535a|*compl_perfect match probe
vvi|miR-1936|2mut|as|	ggcgtcctctctctccctgtca	vvi-miR535a|*compl_mismatch probe
vvi|miR-1937|nat|as|	ggcgtgctctctctcgctgtca	vvi-miR535b|*compl_perfect match probe
vvi|miR-1937|2mut|as|	ggcgtcctctctctccctgtca	vvi-miR535b|*compl_mismatch probe
vvi|miR-1938|nat|as|	ggcgtgctctctctcgctgtca	vvi-miR535c|*compl_perfect match probe
vvi|miR-1938|2mut|as|	ggcgtcctctctctccctgtca	vvi-miR535c|*compl_mismatch probe
vvi|miR-1939|nat|as|	ggcgtgctctctgtcgctgtca	vvi-miR535d|*compl_perfect match probe

vvi|miR-1939|2mut|as|	ggcgtcctctctgtccctgtca	vvi-miR535d|*compl_mismatch probe
vvi|miR-1940|nat|as|	ggcgtgctctctgtcgctgtca	vvi-miR535e|*compl_perfect match probe
vvi|miR-1940|2mut|as|	ggcgtcctctctgtccctgtca	vvi-miR535e|*compl_mismatch probe
vvi|miR-1941|nat|as|	tgagatgctcatttgaggaag	vvi-miR828a|*compl_perfect match probe
vvi|miR-1941|2mut|as|	tgagatcctcatttgacgaag	vvi-miR828a|*compl_mismatch probe
vvi|miR-1942|nat|as|	tggatagtcatttcctgcaa	vvi-miR828b|*compl_perfect match probe
vvi|miR-1942|2mut|as|	tggatagccattttctgcaa	vvi-miR828b|*compl_mismatch probe
vvi|miR-1943|nat|as|	attagttccttcatgagtta	vvi-miR845a|*compl_perfect match probe
vvi|miR-1943|2mut|as|	attagtttcttcataagtta	vvi-miR845a|*compl_mismatch probe
vvi|miR-1944|nat|as|	attagttccttcatgagtt	vvi-miR845b|*compl_perfect match probe
vvi|miR-1944|2mut|as|	attagtttcttcataagtt	vvi-miR845b|*compl_mismatch probe
vvi|miR-1945|nat|as|	tgttttggttgtctgagttt	vvi-miR845c|*compl_perfect match probe
vvi|miR-1945|2mut|as|	tgttttcgttctctgagttt	vvi-miR845c|*compl_mismatch probe
vvi|miR-1946|nat|as|	cattaattatccctagggcca	vvi-miR845d|*compl_perfect match probe
vvi|miR-1946|2mut|as|	cattaattattcctagtgcca	vvi-miR845d|*compl_mismatch probe
vvi|miR-1947|nat|as|	cattaattatccctagggcca	vvi-miR845e|*compl_perfect match probe
vvi|miR-1947|2mut|as|	cattaattattcctagtgcca	vvi-miR845e|*compl_mismatch probe

Degradation and Negative control probes have been used to define background level. Mismatch probes have been designed by the Combimatrix Company for each perfect match probe, to asses the quality of each hybridization. For each miRNA precursor molecules many different probes have been designed. In particular there are probes designed on the predicted mature miRNA; probes designed on the predicted miRNA* (marked as |*); probes shifted of 5 or 10 nucleotide towards the 5’ (|+5 and |+10) or the 3’(|-5 and |-10)with respect to the start of the expected mature miRNA; probes shifted of 5 or 10 nucleotide towards the 5’ (|*+5 and |*+10) or the 3’(|*-5 and |*-10)with respect to the start of the expected mature miRNA*, probes of exactly 20 nucleotide designed on the predicted miRNA, when longer than 20nt (|0); probes of exactly 20 nucleotide designed on the predicted miRNA*, when longer than 20nt (|*0), probes designed on the precursor sequence, outside the miRNA or miRNA* region (|ctrl) and finally complementary probes to the miRNA probe (|compl) and to the miRNA* (|*compl).
